# Remote C−H functionalization using radical translocating arylating groups

**DOI:** 10.1038/s41467-018-05193-6

**Published:** 2018-07-18

**Authors:** Florian W. Friese, Christian Mück-Lichtenfeld, Armido Studer

**Affiliations:** 1 Organisch-Chemisches Institut, Westfälische Wilhelms-Universität, Corrensstraße 40, 48149 Münster, Germany; 2 Center for Multiscale Theory and Computation (CMTC), Corrensstraße 40, 48149 Münster, Germany

## Abstract

Site selective chemical functionalization at unactivated C(sp^3^)−H bonds is highly challenging and recent successful studies mostly focus on the use of transition metal catalysis in combination with directing groups. Radical chemistry offers a complementary approach with the Barton and the Hofmann-Löffler-Freytag reactions being landmark contributions in the area of remote C−H functionalization at unactivated aliphatic sites. Herein we introduce the concept of radical translocation arylation at unactivated secondary and tertiary C(sp^3^)−H bonds in various alcohols. The straightforward two-step sequence comprises an ionic alcohol sulfonylation with especially designed ortho-iodoaryl sulfonyl chlorides followed by a radical cascade reaction including aryl radical generation, remote radical translocation, aryl migration, reduction, and SO_2_ extrusion to give the corresponding γ-arylated alcohols. Moderate to good yields are obtained, remote C−H arylation occurs with excellent regioselectivity and for secondary C(sp^3^)−H bonds good to excellent stereoselectivity is achieved.

## Abstract (web summary)

Selective remote functionalization of aliphatic C(sp^3^)−H bonds is highly challenging and often requires transition metals and/or directing groups. Here, the authors show the γ-arylation of aliphatic alcohols via a two-step radical translocation and subsequent radical aryl migration.

## Introduction

**Fig. 1:**
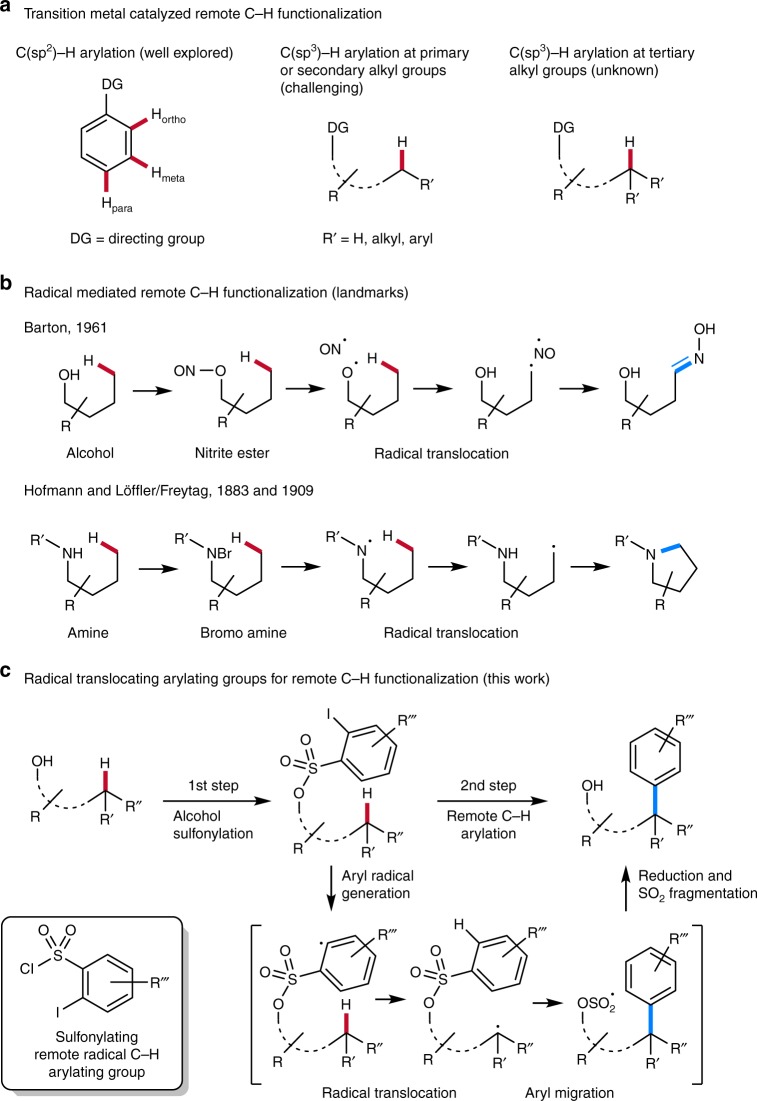
Remote C−H functionalization. **a** Different substrates for transition-metal catalyzed direct C(sp^2^)−H and C(sp^3^)-arylation containing a functional group to direct the C−H activation step. The directing group controls regiochemistry of the C−H activation step. **b** Remote C−H functionalization via radical H-atom transfer from C−H to activated N- and O-centered radicals. **c** Two step remote radical C−H arylation comprising radical translocation and subsequent radical aryl migration

Remote C−H functionalization of inherently unreactive σ-bonds, which offers the possibility for late stage chemical modification of various structural motifs, is emerging as a valuable route in retrosynthetic analysis^1^. Transition-metal catalysis has been successfully used along these lines and regioselectivity is generally achieved by installing directing groups^2–4^. As compared to the arene C(sp^2^)−H functionalization, directed C(sp^3^)−H modification is not as well investigated and only few reports on metal-catalyzed remote C(sp^3^)−H arylation have appeared (Fig. 1a)^2,4–8^. As an alternative to the transition metal mediated C−H activation, radical chemistry offers great potential for remote C−H functionalization. This has a long tradition with landmark papers being published several decades ago. Barton showed that nitrite esters, which are readily generated in situ from alcohols, can be homolyzed to the corresponding alkoxyl radicals that undergo 1,5-H-transfer (radical translocation) to give C-radicals. NO-trapping and tautomerization eventually provide the corresponding oximes (Fig. 1b)^9^. The Hofmann-Löffler-Freytag reaction, discovered more than a century ago, uses in situ generated bromo amines for remote radical C−H functionalization at unactivated positions (Fig. 1b)^10,11^. Homolysis of the N−Br bond leads to N-centered radicals that undergo radical translocation to give C-radicals that are brominated by the intermediate N−Br derivatives in a chain reaction. Ionic cyclization then affords valuable pyrrolidines. Guided by these pioneering studies, modern variants for radical translocation functionalization via N-radicals have appeared recently^12,13^.

Curran introduced the valuable concept of radical translocating groups where reactive aryl radicals are used for remote radical generation via H atom transfer reactions to C-radicals^14,15^. This approach was applied by Baran to the site selective alkane dehydrogenation in complex compounds mimicking natural dehydrogenases^16^. We herein introduce a method for radical translocation arylation of unactivated secondary and tertiary C(sp^3^)−H bonds using radical translocating arylating groups (Fig. 1c). In this straightforward two-step process, alcohols are first sulfonylated with ortho-iodoaryl sulfonyl chlorides that we have especially designed for this purpose. The second step is a radical cascade comprising aryl radical generation, radical translocation, radical aryl migration from sulfur to carbon^17,18^, reduction and ionic SO_2_ extrusion to eventually give the remote γ-C−H arylated alcohols. The method works for remote arylation of secondary and tertiary aliphatic C−H bonds and in case of prochiral secondary C-radicals, high selectivities can be achieved in the C−H functionalization step. Notably, this method allows constructing all-carbon quaternary C-centers which can currently not be achieved using transition-metal mediated C−H arylation documenting the power of radical chemistry^19^.

## Results

### Preparation of substrates and reaction optimization

**Fig. 2:**
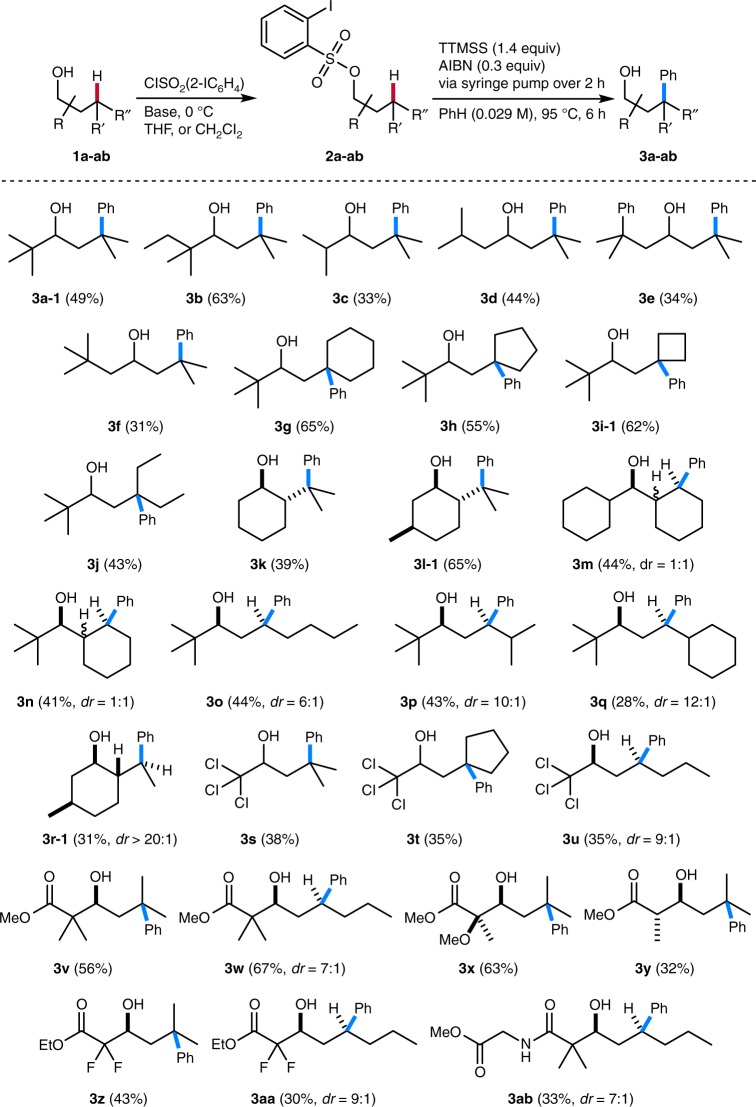
Remote C−H phenylation via radical generation, translocation, and phenyl migration. Sulfonylation conditions: ClSO_2_Aryl, Me_3_N·HCl, Et_3_N, ROH in CH_2_Cl_2_ at 0 °C or ClSO_2_Aryl, Me_3_N, ROLi in THF at 0 °C, see Supplementary Methods. Variation of the alcohol. Phenylation of tertiary and secondary C(sp^3^)−H bonds. For the latter, moderate to excellent stereoselectivities are obtained and only the major diastereoisomer is drawn, if applicable (Fig. 3)

ortho-Iodoarylsulfonyl chlorides, herein introduced as sulfonylating radical translocating arylating groups, were prepared from commercially available ortho-aminosulfonic acids, para-tolylsulfonyl chloride, and 2-fluoro-3-iodopyridine (see Supplementary Methods). Sulfonylation of the secondary alcohols **1a**–**ab** provided sulfonates **2a**–**ab** (Fig. 2), while tertiary alcohols could not efficiently be protected under these conditions. The key radical translocation arylation is best conducted using commercial tris(trimethylsilyl)silane (TTMSS)^20^ as a radical chain reducing reagent and AIBN (α,α’-azobisisobutyronitrile) as an initiator in benzene at elevated temperature (See Supplementary Table 1).

**Fig. 3:**
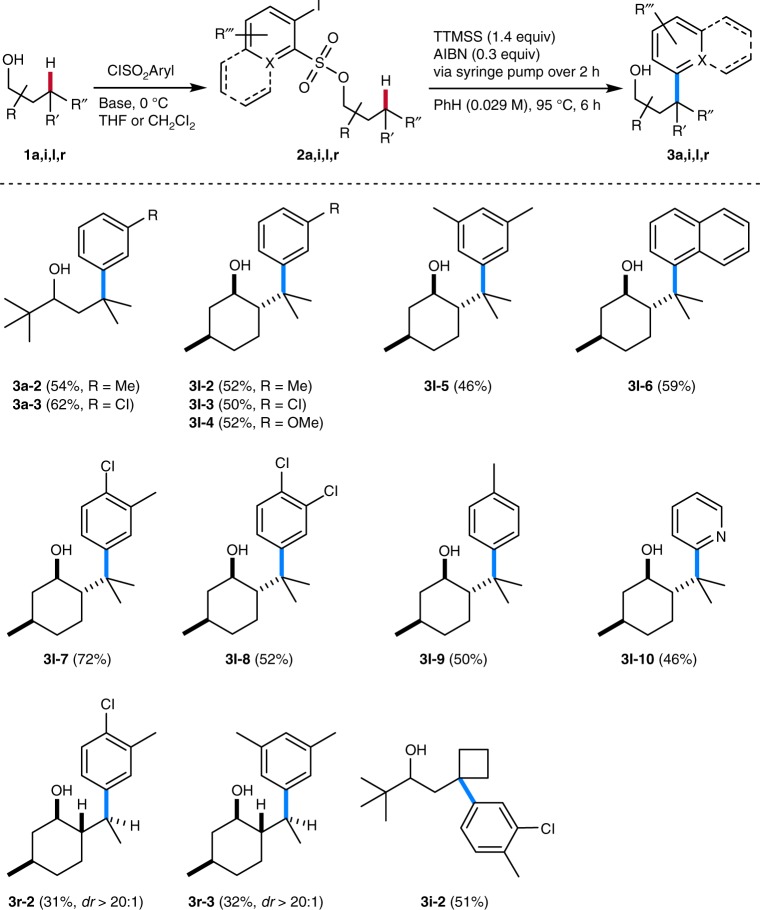
Variation of alcohol and arylating translocating group. Sulfonylation conditions: ClSO_2_Aryl, Me_3_N·HCl, Et_3_N, ROH in CH_2_Cl_2_ at 0 °C or ClSO_2_Aryl, Me_3_N, ROLi in THF at 0 °C, see Supplementary Methods

### Substrate scope

The sulfonates **2a**–**ab** reacted in moderate to good yields to the corresponding C−H phenylated alcohols **3a**–**ab** (Fig. 2). For substrates with a tertiary C−H bond at the γ-position, C−H functionalization occurred with high regiocontrol and other regioisomers could not be isolated. Notably, sulfonate **2g** has six CH_2_ and three CH_3_ entities along with the single tertiary C−H site where C−C bond forming functionalization occurred, documenting the high regioselectivity for the radical 1,7-H translocation. An impressive example is the transformation of natural menthol **1l**, where a perfect regioselectivity was achieved for the remote C−H phenylation. Menthol bears three tertiary C−H bonds, three CH_2_ moieties, three CH_3_ groups and the radical C−H activation occurred with complete regioselectivity to eventually give **3l**-**1**. As shown by the successful preparation of **3m**−**3r**-**1**, **3u**, **3w**, **3aa** and **3ab**, C−H functionalization is not restricted to tertiary C−H bonds. Regioselectivity for 1,7-radical translocation was very high also in these cases and phenylation of a CH_2_ group next to a thermodynamically more activated tertiary C−H site was possible (see **3m**, **3n**, **3p**, **3q** and **3r**-**1**). The excellent regioselectivity for the functionalization was further supported by DFT calculations addressing the regiochemistry determining 1,7-H-atom transfer step (see below). Remarkably, the methodology can also be applied to various readily accessible aldol motifs and the corresponding arylated aldol products **3v**-**ab** could be isolated in moderate to good yields. If applicable, the C−C bond forming phenyl migration occurred with good to excellent diastereocontrol (**3o**–**q**, **3r**-**1**, **3** **u**, **3w**, **3aa**, **3ab**). In the remote C−H phenylation of alcohols **1m** and **1n**, 2 products out of 4 possible diastereoisomers were formed. The initial 1,7-H transfer does not proceed stereoselectively in these cases but the proceeding phenyl group transfer occurs with excellent stereocontrol (see also theoretical studies below). In case of sulfonates derived from primary alcohols, remote C−H functionalization failed and only the corresponding hydrodeiodinated sulfonates were observed (see Supplementary Methods). Likely, both the 1,7-H-atom transfer and also the aryl migration are slowed down for these less rigid substrates. The hydrodeiodination compound was identified as the major side product also in the successful remote phenylations of the secondary aryl sulfonates. Another problem occasionally observed for some substrates explaining diminished yields is the ionic elimination of the ortho-iodoaryl sulfonic acid in the starting sulfonates.

**Fig. 4:**
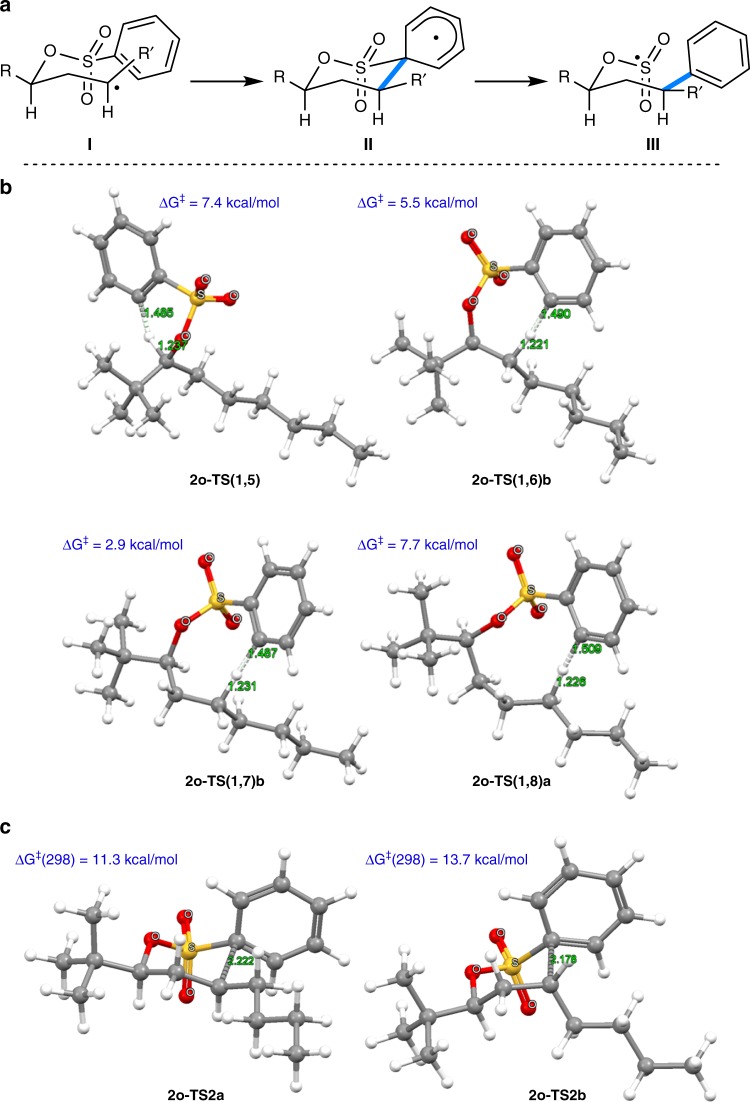
Regioselectivity and stereoselectivity of the remote C−H arylation. **a** Model to explain the stereochemical outcome of the radical phenyl migration reaction. **b** DFT studies on the regioselectivity in a non-cyclic system. Transition state structures with relative activation energies for the 1,5-, 1,6-, 1,7-, and 1,8-H translocation in the radical derived from **2o**. Only the more favorable transfer of two hydrogens at positions 6, 7, and 8 is shown. **c** Stereodetermining step in the arylation of secondary C−H bonds (**I** → **II**): transition structures leading to cyclohexadienyl radicals formed as intermediates in the aryl migration

Switching to other sulfonylating translocating groups will allow for straightforward variation of the aryl group in these remote alcohol functionalizations (Fig. 3). Along these lines, we reacted various arylsulfonates under the optimized conditions and isolated the targeted C−H arylated product alcohols in moderate to good yields (31–72%), documenting that this sequence is not restricted to the parent C−H phenylation (**3a**-**2**, **3a**-**3**, **3l**-**2**−**3l**-**9**, **3r**-**2**, **3r**-**3**, and **3i**-**2**). Alkyl-, halogen- and alkoxy-substituted aryl groups and also the naphthyl group could be introduced into various alcohols at unactivated aliphatic γ-C−H sites via this transformation. The naphthyl-conjugated menthol derivative **3l**-**6** and also the phenyl congener **3l**-**1** are highly efficient auxiliaries in asymmetric synthesis^21,22^ that are now accessible in enantiomerically pure form from natural menthol using this straightforward sequence, convincingly documenting the potential of this approach. Since the radical aryl migration occurs exclusively via ipso-substitution (see Fig. 4a), for substituted aryl groups, arylation proceeded with complete regioselectivity with respect to the migrating aryl moiety. Notably, heteroarylation is also possible as documented by the successful remote 2-pyridylation of menthol (**3l**-**10**).

### Proposed mechanism and DFT-calculations

Regarding arylation of secondary C−H bonds, selectivity for the aryl migration can be understood considering that reaction proceeds from radical intermediate **I** via the chair type cyclohexadienyl radical **II** with the substituents R and R’ in equatorial positions to **III**^23^ (Fig. 4a). This was further supported by DFT calculations (see Supplementary Methods). We addressed the regioselectivity issue and compared the activation energy of the 1,7-H-transfer with respect to the corresponding 1,5-, 1,6- and 1,8-H-translocation in a non-cyclic derivative (**2o**) having large conformational flexibility (Fig. 4b). The DFT studies revealed that the 1,7-H-transfer is favored over the other processes by at least 2.6 kcal/mol, explaining the excellent observed regioselectivity. Calculations also showed that the initial 1,7-H-transfer in the aryl radical generated from **2n** occurs without any diastereoselectivity. The subsequent phenyl migration, forming the cyclohexadienyl radical (**II**), proceeds highly diastereoselectively, explaining the ratio of stereoisomers of **3n** (see Supplementary Fig. 7 and 8). In this rigid bicyclic system, the difference between the two free energy barriers of aryl transfer is at least 1.5 kcal/mol. For the open-chain system (**2o**), a high diastereoselectivity was calculated for the rate-determining ipso-attack leading to cyclohexadienyl radical of type **II** (Fig. 4c). The difference of the free energy barriers (2.4 kcal/mol) explains the good diastereoselectivity by kinetic control of the C−C bond formation. These results validate the stereochemical model suggested in Fig. 4a. The subsequent C−S bond cleavage forming radicals of type **III** shows a low barrier (4.6 kcal/mol for the cleavage of the major isomer **II** to radical **III** for the system leading to **3o**, see Supplementary Fig. 2-4).

## Discussion

In summary, ortho-iodo(hetero)arylsulfonyl chlorides are introduced as readily accessible radical translocating arylating groups for remote C−H (hetero)arylation of various secondary alcohols. The sequence comprises initial ionic sulfonylation of the alcohol functionality followed by a radical cascade reaction. Aryl radical generation in the starting ortho-iodo(hetero)arylsulfonates can be achieved using established radical methodology with TTMSS and AIBN. The intermediate aryl radicals engage in highly selective 1,7 hydrogen abstractions leading to the corresponding remotely translocated C-radicals. These radicals in turn undergo diastereoselective intramolecular (hetero)aryl migrations from sulfur to carbon. Reduction and SO_2_ fragmentation eventually provide the target γ-(hetero)arylated product alcohols. Hence, the translocating arylating groups serve a dual role, they act as directing reagents for regioselective remote C−H activation and as aryl donors for the key C−H functionalization step. Notable features of this cascade are the broad substrate scope, excellent γ-regioselectivity and high diastereoselectivity generally obtained.

## Methods

### General procedure for the remote arylation reaction

To a solution of the 2-iodobenzene sulfonate **2a–ab** (1.0 equiv) in anhydrous benzene (0.032 M) was added a solution of AIBN (0.3 equiv) and TTMSS (1.4 equiv) in anhydrous benzene (0.41 M in respect of TTMSS) over a period of 2 h at 95 °C oil bath temperature under argon via syringe pump. The reaction was further refluxed for additional 4 h. After removal of the solvent in vacuo, the product alcohol was isolated by flash chromatography. In some cases, the addition of TBAF (tetrabutylammonium fluoride, 2.0 equiv, 1.0 M in THF) to the crude product and stirring for 12 h was beneficial for the isolation process.

### Data availability

Crystallographic data for the structures reported in this Article have been deposited at the Cambridge Crystallographic Data Center, under deposition nos. CCDC 1583763 (**3p**), CCDC 1583764 (**S-26**). Copies of the data can be obtained free of charge from www.ccdc.cam.ac.uk/structures/. All other data supporting the findings of this study are available within the article and its Supplementary Data or from the corresponding author upon request.

## Electronic supplementary material

Supplementary Information

Supplementary Data 1

## References

[1] Yamaguchi J, Yamaguchi AD, Itami K (2012). C−H bond functionalization emerging synthetic tools for natural products and pharmaceuticals. Angew. Chem. Int. Ed..

[2] He J, Wasa M, Chan KSL, Shao Q, Yu JQ (2017). Palladium-catalyzed alkyl C−H bond activation. Chem. Rev..

[3] Daugulis O, Roane J, Trane L (2015). Bidentate, monoanionic auxiliary-directed functionalization of carbon-hydrogen bonds. Acc. Chem. Res..

[4] Saint-Denis TG, Zhu RY, Chen G, Wu QF, Yu JQ (2018). Enantioselective C(sp^3^)−H bond activation by chiral transition metal catalysts. Science.

[5] Chen G (2016). Ligand-accelerated enantioselective methylene C(sp^3^)−H bond activation. Science.

[6] Topczewski JJ, Cabrera PJ, Saper NI, Sanford MS (2016). Palladium-catalysed transannular C−H functionalization of alicyclic amines. Nature.

[7] Liu Y, Ge H (2017). Site-selective C−H arylation of primary aliphatic amines enabled by a catalytic transient directing group. Nat. Chem..

[8] Wu QF (2017). Formation of α-chiral centers by asymmetric β-C(sp^3^)−H arylation, alkenylation, and alkynylation. Science.

[9] Barton DHR, Beaton JM, Geller LE, Pechet MM (1961). A new photochemical reaction. J. Am. Chem. Soc..

[10] Hofmann AW (1883). Über die Einwirkung des Broms in alkalischer Lösung auf Amine. Chem. Ber..

[11] Löffler K, Freytag C (1909). Über eine neue Bildungsweise von N-alkylierten Pyrrolidinen. Chem. Ber..

[12] Choi GJ, Zhu Q, Miller DC, Gu CJ, Knowles RR (2016). Catalytic alkylation of remote C−H bonds enabled by proton-coupled electron transfer. Nature.

[13] Chu JCK, Rovis T (2016). Amide-directed photoredox-catalysed C−C bond formation at unactivated sp^3^ C−H bonds. Nature.

[14] Curran DP, Kim D, Liu HT, Shen W (1988). Translocation of radical sites by intramolecular 1,5-hydrogen atom transfer. J. Am. Chem. Soc..

[15] Stateman LM, Nakafuku KM, Nagib DA (2018). Remote C–H functionalization via selective hydrogen atom transfer. Synthesis.

[16] Voica AF, Mendoza A, Gutekunst WR, Fraga JO, Baran PS (2012). Guided desaturation of unactivated aliphatics. Nat. Chem..

[17] Chen ZM, Zhang XM, Tu YQ (2015). Radical aryl migration reactions and synthetic applications. Chem. Soc. Rev..

[18] Li W, Xu W, Xie J, Yu S, Zhu C (2018). Distal radical migration strategy: an emerging synthetic means. Chem. Soc. Rev..

[19] Wu X (2018). Tertiary‐alcohol‐directed functionalization of remote C(sp^3^)−H bonds by sequential hydrogen atom and heteroaryl migrations. Angew. Chem. Int. Ed..

[20] Chatgilialoglu C (1995). Structural and chemical properties of silyl radicals. Chem. Rev..

[21] Corey EJ, Ensley HE (1975). Preparation of an optically active prostaglandin intermediate via asymmetric induction. J. Am. Chem. Soc..

[22] Crossley SWM, Martinez RM, Guevara-Zuluaga S, Shenvi RA (2016). Synthesis of the privileged 8-arylmenthol class by radical arylation of isopulegol. Org. Lett..

[23] Studer A, Bossart M (1998). Stereoselective radical aryl migrations from sulfur to carbon. Chem. Commun..

