# Supplementary material for: Remote C−H functionalization using radical translocating arylating groups
Source: Nat Commun. 2018 Jul 18;9:2808. doi: 10.1038/s41467-018-05193-6 (PMC6051993; doi:10.1038/s41467-018-05193-6)
Supplement: Supplementary file 1 — Supplementary Information [file 41467_2018_5193_MOESM1_ESM.pdf]

Supplementary Materials for

Remote C-H Functionalization Using Radical Translocating Arylating Groups

Studer *et al.*

correspondence to: studer@uni-muenster.de

This PDF file includes:

Supplementary Methods
Supplementary Figure 1 – 136
Supplementary Table 1 – 2
Supplementary References

Supplementary Methods

General Considerations: All reactions involving air or moisture sensitive reagents were carried out in flame-dried glass ware under argon atmosphere using standard *Schlenk* techniques. Solvents used in reactions were either freshly distilled or obtained in extra-dry grade from commercial sources. Dichloromethane (**CH_2Cl_2**) was freshly distilled from P_2O_5. Benzene (**PhH**) was freshly distilled from Na. Diethyl ether (**Et_2O**) was freshly distilled from K-Na-alloy. Tetrahydrofuran (**THF**) was freshly distilled from K. Dry dimethylformamide (**DMF**) was purchased from *Acros Organics*. Solvents for extraction and for flash chromatography were distilled. Tris(trimethylsilyl)silane (**TTMSS**, 97%) and azobisisobutyronitrile (**AIBN**, 98%) were obtained from *Sigma Aldrich*. All other chemicals were purchased from *ABCR*, *Acros Organics*, *Alfa Aesar*, *Fluka*, *Sigma Aldrich*, *Enamine* and *TCI* and were used as received. If not stated otherwise, flash chromatography was performed on *Merck* silica gel 60 (40-63 μm) with an excess argon pressure up to 0.5 bar. Specifically mentioned is the use of *MP BIOMEDICALS Ecochrom*™ *Alumina B* (deactivated to activity III by addition of 6% water) for flash column chromatography. *Merck* silica gel 60 F254 plates were used for thin layer chromatography (**TLC**) using UV light (254/366 nm) or oxidation with $KMnO_4$ (1.5 g in 200 mL H_2O, 5 g $NaHCO_3$) for detection. Melting points (**MP**) were determined with a *Stuart SMP10* and are uncorrected. Infrared spectra (**IR**) were measured on a *Digilab 3100 FT-IR Excalibur Series* spectrometer and the position of the absorption bands is given in wave numbers *v* (cm-1). **^{1}H NMR** (300 MHz, 400 MHz, 500 MHz and 600 MHz), **^{13}C NMR** (75 MHz, 101 MHz, 126 MHz and 151 MHz) and **^{19}F NMR** (282 MHz)) spectra were measured on a *Bruker DPX 300*, *Bruker AV 300*, *Bruker AV 400*, *Agilent DD2 500* or an *Agilent DD2 600* spectrometer. The multiplicity of all signals was described as s (singlet), d (doublet), t (triplet), q (quartet), m (multiplet) and br (broad) as well as its combinations. Chemical shifts (δ in ppm) were referenced on the residual peak of $CDCl_3$ (^{1}H NMR: δ = 7.26; ^{13}C NMR: δ = 77.16), D_2O (^{1}H NMR: δ = 4.79) or DMSO-d_6 (^{1}H NMR: δ = 2.50; ^{13}C NMR: δ = 39.52). **HRMS ESI** (m/z) measurements were performed on a *Bruker MicroTof* and **HRMS APCI** (m/z) on a *Thermo-Fisher Scientific LTQ XL Orbitrap* in toluene. Optical rotation (**OR**) was measured in chloroform using a *Perkin Elmer Polarimeter 341* (Na-D[a], 589 nm). Ozonolysis was carried out using a *FISCHER ozon-generator 500.* Elementary Analysis (**EA**) was performed using a *Elementar Analysensysteme Gmbh - Vario EL III*.

General Procedure for the Preparation of 2-Iodobenzenesulfonic Acids (GP1)

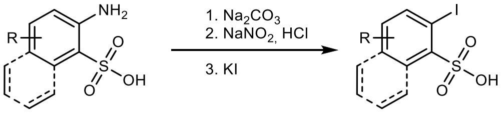

Various 2-iodobenzenesulfonic acids have been prepared following a slightly modified procedure by *Kice and coworkers* (*1*). Commercial aniline-2-sulfonic acids (1.0 equiv) were dissolved in water (0.6 M) and Na_2CO_3 (0.5 equiv) was added slowly until no further evolution of gas was observed. The solution was cooled to 0 °C and $NaNO_2$ (1.1 equiv) added over a period of 15 min. After stirring for 30 min, conc. HCl (2.0 equiv) was added dropwise. Stirring was continued at 0 °C for 30 min and then KI (1.2 – 1.5 equiv, 0.72 – 0.909 M in water) was added slowly. The solution was slowly heated to rt and then refluxed for 1 hr. After cooling to

rt, Na_2SO_3 was added to reduce residual iodine. Removal of the solvent and recrystallization from water gave the pure 2-iodobenzenesulfonic acids.

General Procedure for the Preparation of 2-Iodobenzenesulfonyl Chlorides (GP2)

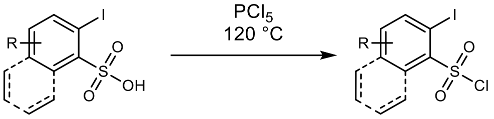

The 2-iodobenzenesulfonic acids were further transformed into the sulfonyl chlorides following a literature known procedure (*2*) by addition of two portions of PCl_5 (200 weight%) and heated to 120 °C for 1 hr. In some cases, the addition of toluene was beneficial to obtain a homogeneous reaction mixture. The mixture was poured on ice, the aqueous phase extracted with Et_2O three times and the combined organic layers concentrated *in vacuo*. Flash column chromatography gave the pure 2-iodobenzene sulfonyl chlorides.

General Procedure for the Preparation of the Sulfonates (GP3)

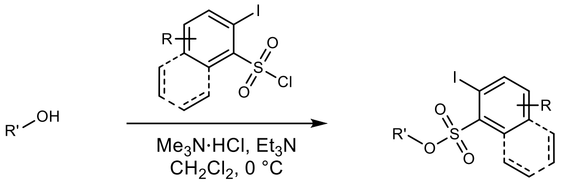

The sulfonates were prepared following a slightly modified procedure by *Tenabe and coworkers* (*3*). The alcohol (1.0 equiv) was dissolved in anhydrous CH_2Cl_2 followed by addition of $Me_3N{\cdot}HCl$ (1.0 equiv) and Et_3N (2.5 equiv). To this mixture, the 2-iodobenzene sulfonyl chloride (1.5 equiv) in anhydrous CH_2Cl_2 was added via a septum at 0 °C. The reaction progress was monitored by TLC and after the alcohol was completely consumed, imidazole was added until no remaining sulfonyl chloride was observed by TLC. The reaction mixture was transferred onto a column and directly purified by flash chromatography.

General Procedure for the Preparation of the Sulfonates (GP4)

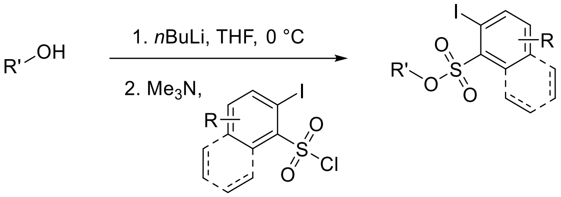

For sterically more hindered alcohols, a different sulfonylation method was used. To this end, a Schlenk flask was charged with the alcohol (1.0 equiv) in anhydrous THF under argon. *n*BuLi (1.2 equiv) was added slowly at 0 °C and stirring was continued for 30 min. At the same time, a second Schlenk flask was charged with the sulfonyl chloride (1.2 equiv) in anhydrous THF and treated with Me_3N (1.5 equiv, 2.0 M in THF) at 0 °C and stirring was continued for 30 min. The alcoholate solution was then transferred via a cannula to the sulfonyl chloride solution. The reaction progress was monitored by TLC and after the alcohol was completely consumed, imidazole was added until no remaining sulfonyl chloride was observed by TLC. The reaction mixture was transferred onto a column and directly purified by flash chromatography. Most compounds prepared by this procedure turned out to readily decompose upon removal of solvent. Therefore they were

stored in Et_2O. For the arylation reaction, the solvent was removed and the pure compound directly dissolved in benzene.

General Procedure for the Remote Arylation Reaction (GP5)

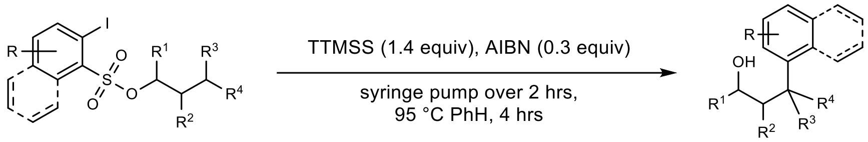

To a solution of the 2-iodobenzene sulfonate (1.0 equiv) in anhydrous benzene (0.032 M) was added a solution of AIBN (0.3 equiv) and TTMSS (1.4 equiv) in anhydrous benzene (0.41 M in respect of TTMSS) over a period of 2 hrs at 95 °C oil bath temperature under argon via syringe pump. The reaction was further refluxed for additional 4 hrs. After removal of the solvent *in vacuo*, the product was isolated by flash chromatography. In some cases, the addition of TBAF (tetrabutylammonium fluoride, 2.0 equiv, 1.0 M in THF) to the crude product and stirring for 12 hrs was beneficial for the isolation process.

Analytical Data of Compounds

2-Iodobenzenesulfonic acid (S-1)

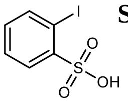

S-1 was prepared following **GP1** with aniline-2-sulfonic acid (1.0 equiv, 30.0 mmol, 5.20 g), Na_2CO_3 (0.5 equiv, 15.0 mmol, 1.59 g), $NaNO_2$ (1.1 equiv, 33.0 mmol, 2.28 g), conc. HCl (6.0 mL) and KI (1.2 equiv, 36.0 mmol, 5.98 g) in water (50 mL). Na_2SO_3 was added until residual iodine was reduced completely. Recrystallization from water gave **S-1** (6.67 g, 23.5 mmol, 78%) as an orange solid. **MP**: > 300 °C. **^{1}H NMR** (300 MHz, DMSO-d_6, 299 K) δ (ppm) = 7.92 (dd, J = 7.8 Hz, J = 1.7 Hz, 1H), 7.88 (dd, J = 7.8 Hz, J = 1.2 Hz, 1H), 7.34 (ddd, J = J = 7.6 Hz, J = 1.3 Hz, 1H), 6.99 (dd, J = J = 7.6 Hz, J = 1.8 Hz, 1H). **^{13}C NMR** (75 MHz, DMSO-d_6, 299 K) δ (ppm) = 149.7 (C), 140.8 (CH), 130.3 (CH), 128.1 (CH), 127.5 (CH), 93.3 (C). **HRMS** (ESI) *m/z* = 282.8931 calcd. for $C_6H_5IO_3S^-$ $[M-H]^-$, found: 282.8923. The analytical data are in accordance with those described in literature (*1*).

2-Iodonaphthalene-1-sulfonic acid (S-2)

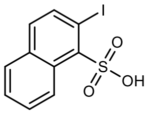

S-2 was prepared following **GP1** with 2-aminonaphthalene-1-sulfonic acid (1.0 equiv, 6.0 mmol, 1.34 g), Na_2CO_3 (0.5 equiv, 3.0 mmol, 318 mg), $NaNO_2$ (1.1 equiv, 6.6 mmol, 455 mg), conc. HCl (1.2 mL) and KI (1.2 equiv, 7.2 mmol, 1.20 g) in water (10 mL). The mixture wasn't refluxed but instead stirred at rt for 18 hrs. Recrystallization from water gave **S-2** (1.15 g, 3.31 mmol, 56%) as a red solid. **MP**: > 300 °C. **FT IR** (neat) ν (cm^{-1}): 1621, 1585, 1552, 1500, 1467, 1421, 1375, 1292, 1191, 1088, 1049, 992, 966, 863, 810, 770, 745, 685, 614. **^{1}H NMR** (300 MHz, D_2O, 299 K) δ (ppm) = 8.92 (d, J = 8.7 Hz, 1H), 8.07 (d, J = 8.7 Hz, 1H), 7.82 (dd, J = 7.4 Hz, J = 1.5 Hz, 1H), 7.68 – 7.52 (m, 2H), 7.50 (d, J = 8.7 Hz, 1H). **^{13}C NMR** (75 MHz, D_2O, 299 K) δ (ppm) = 140.8 (C), 139.1 (CH), 133.4 (C), 132.2 (CH), 130.4 (C), 128.7 (CH), 127.6 (CH), 126.8 (CH), 126.0 (CH), 92.1 (C). **HRMS** (ESI) *m/z* = 332.9088 calcd. for $C_{10}H_6IO_3S^-$ $[M-H]^-$, found: 332.9078.

2-Iodo-5-methylbenzenesulfonic acid (S-3)

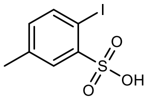

S-3 was prepared following **GP1** with 2-amino-5-methylbenzenesulfonic acid (1.0 equiv, 30.0 mmol, 5.62 g), Na_2CO_3 (0.5 equiv, 15.0 mmol, 1.59 g), $NaNO_2$ (1.1 equiv, 33.0 mmol,

2.28 g), conc. HCl (6.0 mL) and KI (1.2 equiv, 36.0 mmol, 5.98 g) in water (50 mL). Recrystallization from water gave **S-3** (7.06 g, 23.7 mmol, 79%) as a pale yellow solid. **MP**: > 300 °C. **^{1}H NMR** (300 MHz, DMSO-*d*$_6$, 299 K) δ (ppm) = 7.88 – 7.64 (m, 2H), 6.84 (ddd, *J* = 7.8 Hz, *J* = 2.3 Hz, *J* = 0.8 Hz, 1H), 2.25 (s, 3H, CH_3). **^{13}C NMR** (75 MHz, DMSO-*d*$_6$, 299 K) δ (ppm) = 149.7 (C), 140.5 (CH), 136.9 (C), 130.6 (CH), 128.8 (CH), 89.1 (C), 20.5 (CH_3). **HRMS** (ESI) *m/z* = 296.90878 calcd. for $C_7H_6IO_3S^-$ [M−H]$^-$, found: 296.90714. The analytical data are in accordance with those described in literature (*4*).

2-Iodo-5-methoxybenzenesulfonic acid (S-4)

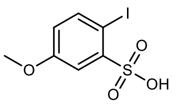

S-4 was prepared following **GP1** with 2-amino-5-methoxybenzenesulfonic acid (1.0 equiv, 30.0 mmol, 6.07 g), Na_2CO_3 (0.5 equiv, 15.0 mmol, 1.59 g), $NaNO_2$ (1.1 equiv, 33.0 mmol, 2.28 g), conc. HCl (6.0 mL) and KI (1.2 equiv, 36.0 mmol, 5.98 g) in water (50 mL). Na_2SO_3 was added until residual iodine was reduced completely. Recrystallization from water gave **S-4** (10.9 g, 28.3 mmol, 94%) as a brown solid. **MP**: decomposition > 180 °C. **FT IR** (neat) ν (cm^{-1}): 3439, 2960, 2837, 2582, 1459, 1439, 1381, 1288, 1262, 1227, 1189, 1139, 1101, 1060, 1031, 1000, 869, 820, 695, 627. **^{1}H NMR** (300 MHz, D_2O, 299 K) δ (ppm) = 7.98 (d, *J* = 8.7 Hz, 1H), 7.63 (d, *J* = 3.1 Hz, 1H), 6.85 (dd, *J* = 8.7 Hz, *J* = 3.1 Hz, 1H), 3.87 (s, 3H, CH_3). **^{13}C NMR** (75 MHz, D_2O, 299 K) δ (ppm) = 159.1 (C), 146.0 (C), 143.0 (CH), 118.2 (CH), 114.7 (CH), 79.6 (C), 55.8 (CH_3). **HRMS** (ESI) *m/z* = 312.9037 calcd. for $C_7H_6IO_4S^-$ [M−H]$^-$, found: 312.9033.

5-Chloro-2-iodo-benzenesulfonic acid (S-5)

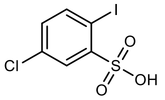

S-5 was prepared following **GP1** with 2-amino-5-chlorobenzenesulfonic acid (1.0 equiv, 24.1 mmol, 5.0 g), Na_2CO_3 (0.5 equiv, 12.1 mmol, 1.28 g), $NaNO_2$ (1.1 equiv, 26.5 mmol, 1.83 g), conc. HCl (4.8 mL) and KI (1.2 equiv, 28.9 mmol, 4.80 g) in water (40 mL). Na_2SO_3 was added until residual iodine was reduced completely. Recrystallization from water gave **S-5** (5.95 g, 18.7 mmol, 78%) as an orange solid. **MP**: Decomposition > 240 °C. **FT IR** (neat) ν (cm^{-1}): 3627, 3454, 1665, 1617, 1441, 1365, 1252, 1218, 1150, 1134, 1105, 1050, 1000, 886, 809, 673, 609, 588, 557. **^{1}H NMR** (300 MHz, D_2O, 299 K) δ (ppm) = 8.08 (d, *J* = 8.4 Hz, 1H), 8.05 (d, *J* = 2.6 Hz, 1H), 7.27 (dd, *J* = 8.4 Hz, *J* = 2.6 Hz, 1H). **^{13}C NMR** (75 MHz, D_2O, 299 K) δ (ppm) = 146.6 (C), 143.4 (CH), 134.3 (C), 132.2 (CH), 128.4 (CH), 88.6 (C). **HRMS** (ESI) *m/z* = 316.8542 calcd. for $C_6H_3ClIO_3S^-$ [M−H]$^-$, found: 316.8519.

2-Iodo-3,5-dimethylbenzenesulfonic acid (S-6)

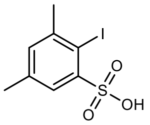

S-6 was prepared following **GP1** with 2-amino-3,5-dimethylbenzenesulfonic acid (1.0 equiv, 30.0 mmol, 6.04 g), Na_2CO_3 (0.5 equiv, 15.0 mmol, 1.59 g), $NaNO_2$ (1.1 equiv, 33.0 mmol, 2.28 g), conc. HCl (6.0 mL) and KI (1.5 equiv, 45.0 mmol, 7.47 g) in water (50 mL). The mixture wasn't refluxed but instead stirred at rt for 18 hrs. Na_2SO_3 was added until residual iodine was reduced completely. Recrystallization from water gave **S-6** (6.81 g, 21.8 mmol, 73%) as a brown solid. **MP**: > 300 °C. **FT IR** (neat) ν (cm^{-1}): 3618, 3445, 2918, 1668, 1623, 1451, 1409, 1381, 1239, 1210, 1160, 1057, 1050, 1001, 959, 897, 850, 697, 627, 590. **^{1}H NMR** (300 MHz, DMSO-*d*$_6$, 299 K) δ (ppm) = 7.61 (d, *J* = 2.3 Hz, 1H), 7.09 (d, *J* = 2.3, 1H), 2.39 (s, 3H, CH_3), 2.22 (s, 3H, CH_3). **^{13}C NMR** (75 MHz, DMSO-*d*$_6$, 299 K) δ (ppm) = 150.7 (C), 142.0 (C), 136.2 (C), 130.1 (CH), 126.5 (CH), 96.2 (C), 29.7 (CH_3), 20.3 (CH_3). **HRMS** (ESI) *m/z* = 310.9244 calcd. for $C_8H_8IO_3S^-$ [M−H]$^-$, found: 310.9239.

4-Chloro-2-iodo-5-methylbenzenesulfonic acid (S-7)

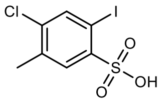

S-7 was prepared following **GP1** with 2-amino-4-chloro-5-methylbenzenesulfonic acid (1.0 equiv, 30.0 mmol, 6.65 g), Na_2CO_3 (0.5 equiv, 15.0 mmol, 1.59 g), $NaNO_2$ (1.1 equiv, 33.0 mmol, 2.28 g), conc. HCl (6.0 mL) and KI (1.5 equiv, 45.0 mmol, 7.47 g) in water (50 mL). Na_2SO_3 was added until residual iodine was reduced completely. Recrystallization from water gave **S-7** (8.16 g, 24.5 mmol, 82%) as a beige solid. **MP**: > 300 °C. **FT IR** (neat) ν cm^{-1}:3616, 3473, 1668, 1621, 1574, 1542, 1449, 1380, 1334, 1210, 1126, 1069, 1033, 890, 716, 655, 609. **^{1}H NMR** (400 MHz, DMSO-d_6, 299 K) δ (ppm) = 7.88 – 7.82 (m, 2H), 2.27 (s, 3H, CH_3). **^{13}C NMR** (101 MHz, DMSO-d_6, 299 K) δ (ppm) = 149.0 (C), 139.6 (CH), 134.6 (C), 133.5 (C), 130.2 (CH), 90.1 (C), 19.2 (CH_3). **HRMS** (ESI) *m/z* = 330.8698 calcd. for $C_7H_5ClIO_3S^-$ $[M-H]^-$, found: 330.8695.

4,5-Dichloro-2-iodobenzenesulfonic acid (S-8)

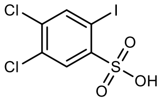

S-8 was prepared following **GP1** with 2-amino-4,5-dichlorobenzenesulfonic acid (1.0 equiv, 30.0 mmol, 7.20 g), Na_2CO_3 (0.5 equiv, 15.0 mmol, 1.59 g), $NaNO_2$ (1.1 equiv, 33.0 mmol, 2.28 g), conc. HCl (6.0 mL) and KI (1.5 equiv, 45.0 mmol, 7.47 g) in water (50 mL). The mixture wasn't refluxed but instead stirred at rt for 18 hrs. Na_2SO_3 was added until residual iodine was reduced completely. Recrystallization from water gave **S-8** (10.3 g, 29.1 mmol, 97%) as a beige solid. **MP**: > 300 °C. **FT IR** (neat) ν cm^{-1}:3632, 3455, 3081, 1668, 1618, 1558, 1530, 1435, 1316, 1249, 1216, 1143, 1119, 1060, 1024, 892, 867, 849, 682, 653, 617, 579. **^{1}H NMR** (300 MHz, DMSO-d_6, 299 K) δ (ppm) = 8.12 (s, 1H), 7.97 (s, 1H). **^{13}C NMR** (75 MHz, DMSO-d_6, 299 K) δ (ppm) = 150.8 (C), 141.5 (CH), 131.5 (C), 130.4 (C), 128.9 (CH), 92.2 (C). **HRMS** (ESI) *m/z* = 350.8152 calcd. for $C_6H_2Cl_2IO_3S^-$ $[M-H]^-$, found: 350.8146.

Isopropyl 4-methylbenzenesulfonate (S-9)

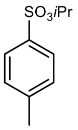

p-Toluenesulfonyl chloride (1.00 equiv, 25.7 mmol, 4.90 g) and $Me_3N\cdot HCl$ (0.05 equiv, 1.29 mmol, 123 mg) was dissolved in anhydrous CH_2Cl_2 (10 mL). *i*PrOH (10.0 equiv, 257 mmol, 19.8 mL) and Et_3N (2.50 equiv, 64.2 mmol, 9.03 mL) was added and the solution was stirred for 24 hrs. After the reaction was quenched by addition of H_2O (40 mL), the aqueous layer was extracted with Et_2O (4 x 50 mL). The combined organic layers were washed with H_2O and brine (10 mL each) and dried over $MgSO_4$. Removal of the solvent *in vacuo* yielded **S-9** (5.01 g, 23.4 mmol, 91%) as a slightly orange liquid. **^{1}H NMR** (300 MHz, $CDCl_3$, 299 K) δ (ppm) = 7.83 – 7.73 (m, 2H), 7.38 – 7.28 (m, 2H), 4.72 (hept, *J* = 6.3 Hz, 1H), 2.43 (s, 3H), 1.26 (d, *J* = 6. 3 Hz, 6H). **^{13}C NMR** (75 MHz, $CDCl_3$, 299 K) δ (ppm) = 144.5 (C), 134.7 (C), 129.8 (CH), 127.7 (CH), 77.1 (CH), 22.9 (CH_3), 21.7 (CH_3). **HRMS** (ESI) *m/z* = 237.0556 calcd. for $C_{10}H_{14}NaO_3S^+$ $[M+Na]^+$, found: 237.0553. The analytical data are in accordance to those reported in the literature (*5*).

Sodium 2-iodo-4-methylbenzenesulfonate (S-10)

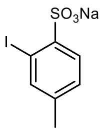

S-10 was prepared following a modified procedure by *Bonfiglio* (*6*). **S-9** (1.0 equiv, 10.9 mmol, 2.34 g) was dissolved in anhydrous THF (100 mL) under argon. *n*BuLi (1.6 M in hexane, 1.1 equiv, 12.0 mmol, 7.51 mL) was added over a period of 1 hr at −78 °C. The solution was stirred for 5 hrs and I_2 (1.2 equiv, 13.1 mmol, 3.33 g, dissolved 5 mL in THF) was added over a period of 15

min until the brown colour persisted. The solution was diluted with Et_2O (50 mL), was allowed to warm to room temperature and was then filtered through a plug of silica. After the solvent was removed *in vacuo*, the crude product was dissolved in MeCN (55 mL) and NaI (1.1 equiv, 12.0 mmol,1.79 g) and the mixture was heated to 75 °C for 2 hrs. The solvent was removed *in vacuo* and the resulting solid was recrystallized from H_2O yielding **S-10** (2.81 g, 8.78 mmol, 81%) as a colorless solid. **MP**.: > 300 °C. **FT IR** (neat) ν (cm^{-1}): 3653, 3463, 1623, 1591, 1551, 1464, 1441, 1372, 1213, 1146, 1122, 1057, 1031, 1015, 876, 831, 674. ^{1}**H NMR** (300 MHz, D_2O, 299 K) δ 7.98 – 7.94 (m, 1H), 7.88 (d, *J* = 8.0 Hz, 1H), 7.36 – 7.24 (m, 1H), 2.29 (s, 3H). ^{13}**C NMR** (75 MHz, D_2O, 299 K) δ (ppm) = 143.6 (C), 142.4 (CH), 142.1 (C), 129.0 (CH), 128.2 (CH), 91.0 (C), 19.9 (CH_3). **HRMS** (ESI) *m/z* = 296.9088 calcd. for $C_7H_6IO_3S^-$ $[M-Na]^-$, found: 296.9093.

2-Iodobenzenesulfonyl chloride (S-11)

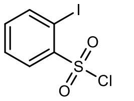

S-11 was prepared following **GP2** with 2-iodobenzenesulfonic acid (1.00 equiv, 14.3 mmol, 4.20 g), PCl_5 (2.82 equiv, 40.3 mmol, 8.40 g) and $POCl_3$ (1.55 equiv, 22.2 mmol, 2.0 mL). After addition to ice, the aqueous layer was extracted with Et_2O (3 x 10 mL). The solvent was removed *in vacuo* and the pure sulfonyl chloride isolated by flash column chromatography (pentane/Et_2O 9:1) as a slightly yellow solid (3.59 g, 11.9 mmol, 80%). **MP**.: 50-51 °C. ^{1}**H NMR** (300 MHz, $CDCl_3$, 299 K) δ (ppm) = 8.23 (ddd, *J* = *J* = 7.9 Hz, *J* = 1.2 Hz, 2H), 7.59 (ddd, *J* = *J* = 7.8 Hz, *J* = 1.3 Hz, 1H), 7.35 (ddd, *J* = *J* = 7.7 Hz, *J* = 1.6 Hz, 1H). ^{13}**C NMR** (75 MHz, $CDCl_3$, 299 K) δ (ppm) = 146.53 (C), 143.93 (CH), 135.57 (CH), 130.58 (CH), 128.98 (CH), 92.27 (C). **EA**.: (calcd): C 23.83 (23.79)/H 1.33 (1.32). The analytical data are in accordance with those described in literature (*1*).

2-Iodonaphthalene-1-sulfonyl chloride (S-12)

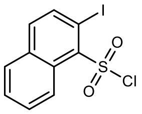

S-12 was prepared by addition of 2-iodonaphthalenesulfonic acid (1.0 equiv, 2.10 mmol, 700 mg) to a mixture of PCl_5 (2.7 equiv, 5.67 mmol, 1.18 g) and $POCl_3$ (1.5 equiv, 3.15 mmol, 0.29 mL). After stirring at 95 °C for 2 hrs, the reaction mixture was quenched by addition to ice. The aqueous layer was extracted with Et_2O (3 x 10 mL). The solvent was removed *in vacuo* and the pure sulfonyl chloride isolated by flash column chromatography (pentane/Et_2O 9:1) as a red solid (560 mg, 1.59 mmol, 76%). **MP**.: 110 °C. **FT IR** (neat) ν (cm^{-1}): 1616, 1581, 1547, 1499, 1446, 1423, 1375, 1338, 1263, 1178, 117, 1157, 1095, 961, 866, 816, 766, 744, 677, 636, 590, 568. ^{1}**H NMR** (300 MHz, $CDCl_3$, 299 K) δ (ppm) = 9.06 – 9.00 (m, 1H), 8.27 (d, *J* = 8.6 Hz, 1H), 7.93 (dd, *J* = 8.0 Hz, *J* = 1.6 Hz, 1H), 7.81 – 7.61 (m, 3H). ^{13}**C NMR** (75 MHz, $CDCl_3$, 299 K) δ (ppm) = 142.5 (C), 139.8 (CH), 136.0 (CH), 133.9 (C), 130.6 (C), 129.6 (CH), 129.2 (CH), 128.0 (CH), 125.2 (CH), 95.8 (C). **EA**.: (calcd): C 34.29 (34.07)/H 1.65 (1.72).

2-Iodo-5-methylbenzenesulfonyl chloride (S-13)

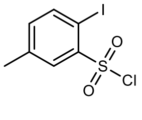

S-13 was prepared following **GP2** with 2-iodo-5-methylbenzenesulfonic acid (1.00 equiv, 13.4 mmol, 4.00 g) and PCl_5 (2.87 equiv, 38.4 mmol, 8.00 g). After quenching with ice, the aqueous layer was extracted with Et_2O (3 x 20 mL). The solvent was removed *in vacuo* and the pure sulfonyl chloride isolated by flash column chromatography (pentane/Et_2O 9:1) as a slightly yellow solid (3.13 g, 9.89 mmol, 74%). **MP**.: 69-71 °C. **FT IR** (neat) ν (cm^{-1}): 2925, 1459, 1382, 1365, 1278, 1260, 1220, 1170, 1098, 1041, 1011, 863, 823, 689, 583. ^{1}**H NMR** (300 MHz, $CDCl_3$, 299 K) δ (ppm) = 8.25 –

7.90 (m, 2H), 7.15 (ddt, J = 7.3 Hz, J = 2.1 Hz, J = 0.8 Hz, 1H), 2.42 (s, 3H, CH_3). **^{13}C NMR** (75 MHz, $CDCl_3$, 299 K) δ (ppm) = 146.3 (C), 143.6 (CH), 139.8 (C), 136.5 (CH), 131.1 (CH), 88.0 (CH), 21.1 (CH_3). **EA**.: (calcd): C 26.70 (26.56)/H 1.76 (1.91).

2-Iodo-5-methoxybenzenesulfonyl chloride (S-14)

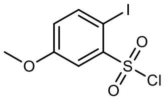

S-14 was prepared by addition of 2-iodo-5-methoxybenzene sulfonic acid (1.0 equiv, 2.51 mmol, 800 mg) to a mixture of PCl_5 (2.7 equiv, 6.78 mmol, 1.41 g) and $POCl_3$ (1.5 equiv, 3.77 mmol, 0.35 mL). After stirring at 95 °C for 2 hrs, the reaction mixture was quenched by addition to ice. The aqueous layer was extracted with Et_2O (3 x 10 mL). The solvent was removed *in vacuo* and the pure sulfonyl chloride isolated by flash column chromatography (pentane/Et_2O 4:1) as a slightly yellow solid (499 mg, 1.50 mmol, 60%). **MP**.: 89 °C. **FT IR** (neat) ν cm^{-1}:3095, 2940, 2840, 1587, 1464, 1437, 1370, 1295, 1263, 1235, 1178, 1033, 1006, 858, 826, 683, 607. **^{1}H NMR** (300 MHz, $CDCl_3$, 299 K) δ (ppm) = 8.03 (d, J = 8.7 Hz, 1H), 7.76 (d, J=3.0 Hz, 1H), 6.91 (dd, J = 8.7 Hz, J = 3.0 Hz, 1H), 3.88 (s, 3H, CH_3). **^{13}C NMR** (75 MHz, $CDCl_3$, 299 K) δ (ppm) = 159.9 (C), 146.9 (C), 144.2 (CH), 122.2 (CH), 115.8 (CH), 79.9 (C), 56.0 (CH_3). **EA**.: (calcd): C 25.34 (25.28)/H 1.65 (1.82).

5-Chloro-2-iodobenzenesulfonyl chloride (S-15)

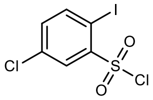

S-15 was prepared by addition of 5-chloro-2-iodobenzenesulfonic acid (1.0 equiv, 9.42 mmol, 3.00 g) to a mixture of PCl_5 (2.7 equiv, 25.4 mmol, 5.30 g) and $POCl_3$ (1.5 equiv, 14.1 mmol, 1.32 mL). After stirring at 95 °C for 1 hr, the reaction mixture was quenched by addition to ice. The aqueous layer was extracted with Et_2O (3 x 20 mL). The solvent was removed *in vacuo* and the pure sulfonyl chloride isolated by flash column chromatography (pentane/Et_2O 9:1) as a yellow solid (2.33 g, 6.62 mmol, 70%). **MP**.: 69-71 °C. **FT IR** (neat) ν (cm^{-1}): 3083, 3057, 1445, 1372, 1360, 1267, 1249, 1172, 1144, 1105, 1087, 1058, 1008, 887, 829, 805, 682, 667, 584. **^{1}H NMR** (300 MHz, $CDCl_3$, 299 K) δ (ppm) = 8.21 (d, J = 2.4 Hz, 1H), 8.12 (d, J = 8.4 Hz, 1H), 7.33 (dd, J = 8.4 Hz, J = 2.5 Hz, 1H). **^{13}C NMR** (75 MHz, $CDCl_3$, 299 K) δ (ppm) =147.5 (C), 144.8 (CH), 135.7 (C), 135.6 (CH) 130.5 (CH), 89.4 (C). **EA**.: (calcd): C 21.42 (21.39)/H 0.66 (0.90).

2-Iodo-3,5-dimethylbenzenesulfonyl chloride (S-16)

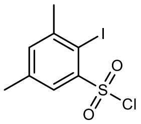

S-16 was prepared by addition of 2-Iodo-3,5-dimethylbenzenesulfonic acid (1.0 equiv, 19.2 mmol, 6.00 g) to a mixture of PCl_5 (2.7 equiv, 51.9 mmol, 10.8 g) and $POCl_3$ (1.5 equiv, 28.8 mmol, 2.65 mL). After stirring at 120 °C for 1 hr, the reaction mixture was quenched by addition to ice. The aqueous layer was extracted with Et_2O (3 x 50 mL). The solvent was removed *in vacuo* and the pure sulfonyl chloride isolated by flash column chromatography (pentane/Et_2O 99:1) as a colorless solid (2.94 g, 8.89 mmol, 46%). **MP**.: 95 °C. **FT IR** (neat) ν (cm^{-1}): 3068, 2987, 2924, 1779, 1593, 1548, 1454, 1438, 1413, 1379, 1360, 1280, 1264, 1264, 1219, 1172, 1142, 1042, 1012, 960, 913, 871, 843, 689, 588. **^{1}H NMR** (300 MHz, $CDCl_3$, 299 K) δ (ppm) = 7.89 (d, J = 2.1 Hz, 1H), 7.38 (d, J = 2.1 Hz, 1H), 2.59 (s, 3H, CH_3), 2.38 (s, 3H, CH_3). **^{13}C NMR** (75 MHz, $CDCl_3$, 299 K) δ (ppm) = 147.1 (C), 146.6 (C), 139.1 (C), 136.2 (CH), 129.1 (CH), 95.1 (C), 30.4 (CH_3), 20.9 (CH_3). **EA**.: (calcd): C 29.06 (29.07)/H 2.41 (2.44).

4-Chloro-2-iodo-5-methylbenzenesulfonyl chloride (S-17)

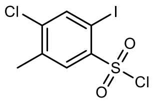

S-17 was prepared by addition of 4-chloro-2-iodo-5-methylbenzene sulfonic acid (1.0 equiv, 9.42 mmol, 3.13 g) to a mixture of PCl_5 (2.7 equiv, 25.4 mmol, 5.30 g) and $POCl_3$ (1.5 equiv, 14.1 mmol, 1.32 mL). After stirring at 95 °C for 1 hr, the reaction mixture was quenched by addition to ice. The aqueous layer was extracted with Et_2O (3 x 20 mL). The solvent was removed *in vacuo* and the pure sulfonyl chloride isolated by flash column chromatography (pentane/Et_2O 1:0 to 99:1) as a colorless solid (2.54 g, 7.24 mmol, 77%). **MP.**: 94 °C. **FT IR** (neat) ν (cm^{-1}): 3086, 1570, 1530, 1449, 1382, 1368, 1327, 1262, 1206, 1172, 1105, 1066, 1037, 893. **^{1}H NMR** (300 MHz, $CDCl_3$, 299 K) δ (ppm) = 8.16 (s, 1H), 8.08 (d, *J* = 0.8 Hz, 1H), 2.43 (s, 3H, CH_3). **^{13}C NMR** (75 MHz, $CDCl_3$, 299 K) δ (ppm) = 144.6 (C), 143.2 (CH), 142.0 (C), 138.0 (C), 132.2 (CH), 88.6 (C), 20.1 (CH_3). **EA.**: (calcd): C 24.01 (23.95)/H 1.33 (1.44).

4,5-Dichloro-2-iodobenzenesulfonyl chloride (S-18)

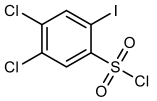

S-18 was prepared by addition of 4,5-dichloro-2-iodobenzene sulfonic acid (1.0 equiv, 9.42 mmol, 3.32 g) to a mixture of PCl_5 (2.7 equiv, 25.4 mmol, 5.30 g) and $POCl_3$ (1.5 equiv, 14.1 mmol, 1.32 mL). After stirring at 95 °C for 2 hrs, the reaction mixture was quenched by addition to ice. The aqueous layer was extracted with Et_2O (3 x 20 mL). The solvent was removed *in vacuo* and the pure sulfonyl chloride isolated by flash column chromatography (pentane/Et_2O 99:1) as a slightly yellow solid (2.58 g, 6.95 mmol, 74%). **MP.**: 99 °C. **FT IR** (neat) ν (cm^{-1}): 3091, 1554, 1525, 1439, 1370, 1308, 1256, 1175, 1156, 1145, 1101, 1047, 901, 878, 846, 783, 683, 621, 604. **^{1}H NMR** (400 MHz, $CDCl_3$, 299 K) δ (ppm) = 8.33 – 8.21 (m, 2H). **^{13}C NMR** (101 MHz, $CDCl_3$, 299 K) δ (ppm) = 145.4 (C), 144.4 (CH), 140.3 (C), 134.1 (C), 131.6 (CH), 89.5 (C). **EA.**: (calcd): C 19.40 (19.46)/H 0.54 (0.49).

2-Iodo-4-methylbenzenesulfonyl chloride (S-19)

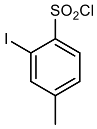

S-19 was prepared by addition of sodium 2-iodo-4-methylbenzenesulfonate (1.0 equiv, 4.06 mmol, 1.30 g) to a mixture of PCl_5 (2.7 equiv, 11.0 mmol, 2.28 g) and $POCl_3$ (1.5 equiv, 6.09 mmol, 0.57 mL). After stirring at 95 °C for 2 hrs, the reaction mixture was quenched by addition to ice. The aqueous layer was extracted with Et_2O (3 x 50 mL). The solvent was removed *in vacuo* and the pure sulfonyl chloride isolated by flash column chromatography (pentane/Et_2O 19:1) as a colorless solid (1.11 g, 3.51 mmol, 87%). **MP.**: 86 °C. **FT IR** (neat) ν (cm^{-1}): 3091, 2928, 1581, 1457, 1378, 1365, 1283, 1268, 1214, 1170, 1153, 1100, 1037, 1020, 963, 900, 840, 816, 748, 691, 661, 645. **^{1}H NMR** (300 MHz, $CDCl_3$, 299 K) δ (ppm) = 8.09 (d, *J* = 8.2 Hz, 1H), 8.03 (dd, *J* = 1.7 Hz, *J* = 0.8 Hz, 1H), 7.35 (ddd, *J* = 8.2 Hz, *J* = 1.7 Hz, *J* = 0.8 Hz, 1H), 2.42 (d, *J* = 0.8 Hz, 3H). **^{13}C NMR** (75 MHz, $CDCl_3$, 299 K) δ (ppm) = 147.4 (C), 144.3(CH), 143.9(C), 130.5(CH), 129.6(CH), 92.3(C), 21.2(CH_3), **HRMS** (ESI) *m/z* = 338.8714 calcd. for $C_7H_6ClINaO_2S^+$ $[M+Na]^+$, found: 338.8728.

2,2,5-Trimethylhexan-3-ol (1a)

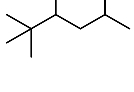

Pivaldehyde (1.0 equiv, 15.0 mmol, 1.63 mL) was dissolved in anhydrous THF (20 mL) under argon atmosphere and *i*BuLi (1.7 M in hexane, 1.1 equiv, 16.5 mmol, 9.71 mL) was added over a period of 1 hr at −78 °C. The reaction mixture was allowed to warm to rt and stirring

was continued for 2 hrs. After quenching by addition of saturated NH_4Cl solution (10 mL), the aqueous layer was extracted with Et_2O (5 x 10 mL). The combined organic layers were dried over $MgSO_4$ and concentrated *in vacuo* to obtain the desired alcohol (1.87 g, 13.0 mmol, 87%) which was used without further purification. **FT IR** (neat) ν (cm^{-1}): 3374, 2955, 2935, 2870, 1468, 1385, 1367, 1307, 1279, 1229, 1192, 1169, 1128, 1114, 1072, 1011, 986, 949, 900, 842, 764, 606. **^{1}H NMR** (300 MHz, $CDCl_3$, 299 K) δ (ppm) = 3.33 – 3.20 (m, 1H), 1.89 – 1.68 (m, 1H), 1.33 – 1.15 (m, 2H), 0.99 – 0.83 (m, 15H). **^{13}C NMR** (75 MHz, $CDCl_3$, 299 K) δ (ppm) = 77.7 (CH), 40.9 (CH_2), 34.9 (C), 25.8 (CH), 25.1 (CH_3), 24.4 (CH_3), 21.5 (CH_3). **HRMS** (ESI) *m/z* = 167.1406 calcd. for $C_9H_{20}NaO^+$ $[M+Na]^+$, found: 167.1397.

2,5,5-Trimethylheptan-4-ol (1b)

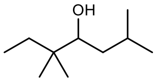

Isovaleraldehyde (1.0 equiv, 15.0 mmol, 1.52 mL) was dissolved in anhydrous THF (20 mL) under argon atmosphere and 1,1-dimethylpropylmagnesium chloride (1.0 M in Et_2O, 1.07 equiv, 16.0 mmol, 16.0 mL) was added over a period of 2 hr at 0 °C. The reaction mixture was allowed to warm to rt and stirring was continued for 2 hrs. After quenching by addition of saturated NH_4Cl solution (10 mL), the aqueous layer was extracted with Et_2O (3 x 50 mL). The combined organic layers were washed with brine (10 mL), dried over $MgSO_4$ and concentrated *in vacuo*. The alcohol was then isolated by flash column chromatography (pentane/Et_2O 9:1) as a colorless liquid (1.00 g, 6.32 mmol, 42%). **FT IR** (neat) ν (cm^{-1}): 3393, 2958, 2935, 2869, 1467, 1385, 1367, 1221, 1171, 1128, 1114, 1078, 1040, 981,951,935,900,875,841,782. **^{1}H NMR** (400 MHz, $CDCl_3$, 299 K) δ (ppm) = 3.35 (dd, *J* = 10.2 Hz, *J* = 2.0 Hz, 1H), 1.87 – 1.70 (m, 1H), 1.44 – 1.13 (m, 5H), 0.94 (d, *J* = 6.7 Hz, 3H), 0.89 (d, *J* = 6.6 Hz, 3H), 0.86 – 0.78 (m, 9H). **^{13}C NMR** (101 MHz, $CDCl_3$, 299 K) δ (ppm) = 76.2 (CH), 40.6 (CH_2), 37.3 (C), 31.2 (CH_2), 25.1 (CH), 24.4 (CH_3), 22.5 (CH_3), 22.3 (CH_3), 21.5 (CH_2), 8.2 (CH_2). **HRMS** (ESI) *m/z* = 181.1563 calcd. for $C_{10}H_{22}NaO^+$ $[M+Na]^+$, found: 181.1559.

2,6-Dimethylheptan-4-ol (1d)

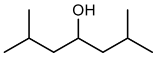

Isovaleraldehyde (1.0 equiv, 15.0 mmol, 1.62 mL) was dissolved in anhydrous THF (20 mL) under argon atmosphere and *i*BuLi (1.7 M in hexane, 1.1 equiv, 16.5 mmol, 9.71 mL) was added over a period of 1 hr at −78 °C. The reaction mixture was allowed to warm to rt and stirring was continued for 2 hrs. After quenching by addition of saturated NH_4Cl solution (10 mL), the aqueous layer was extracted with Et_2O (5 x 10 mL). The combined organic layers were dried over $MgSO_4$ and concentrated *in vacuo* to obtain the desired alcohol (1.91 g, 13.2 mmol, 88%) which was used without further purification. **FT IR** (neat) ν (cm^{-1}): 3343, 2955, 2926, 2870, 1711, 1711, 1468, 1385, 1367, 1316, 1151, 1127, 1055, 1025, 972, 959, 920, 866, 844, 828. **^{1}H NMR** (300 MHz, $CDCl_3$, 299 K) δ (ppm) = 3.74 – 3.62 (m, 1H), 1.81 – 1.54 (m, 3H), 1.40 – 1.08 (m, 4H), 0.94 – 0.76 (m, 12H). **^{13}C NMR** (75 MHz, $CDCl_3$, 299 K) δ (ppm) = 68.2 (CH), 47.6 (CH_2), 24.8 (CH), 23.6 (CH_3), 22.3 (CH_3). **HRMS APCI** *m/z* = 143.14304 calcd. for $C_9H_{19}O^+$ $[M-H]^+$, found: 143.14276.

2,6-Dimethyl-2-phenylheptan-4-ol (1e)

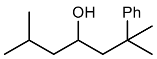

To a slurry of magnesium turnings (1.3 equiv, 14.3 mmol, 348 mg) in anhydrous THF (2.0 mL) was added a drop of 1,2-dibromoethane under an atmosphere of argon followed by a solution of (1-chloro-2-methylpropan-2-yl)benzene (1.0 equiv, 11.0 mmol, 1.86 g) in anhydrous THF

(2.0 mL) while maintaining constant self reflux of the reaction mixture. After 2 hrs, the solution was diluted with anhydrous THF (10 mL) and a solution of isovaleraldehyde (1.0 equiv, 11.0 mmol, 1.18 mL) in anhydrous THF (5.0 mL) was added over a period of 1 hr at 0 °C. The reaction mixture was allowed to warm to rt and then quenched by addition of saturated NH_4Cl solution (10 mL). The aqueous layer was extracted with Et_2O (3 x 50 mL) and the combined organic layers washed with brine (5.0 mL) and dried over $MgSO_4$. Upon removal of the solvent *in vacuo*, flash column chromatography (pentane/Et_2O 9:1) afforded the product as a colorless liquid (1.33 g, 6.04 mmol, 55%). **FT IR** (neat) ν (cm^{-1}): 3380, 2956, 2930, 2870, 1602, 1496, 1467, 1445, 1386, 1367, 1280, 1137, 1079, 1056, 1031, 1003, 928, 887, 842, 763, 698. **^{1}H NMR** (300 MHz, $CDCl_3$, 299 K) δ (ppm) = 7.46 – 7.33 (m, 2H), 7.36 – 7.29 (m, 2H), 7.23 – 7.16 (m, 1H), 3.66 (tdd, *J* = 8.4 Hz, *J* = 4.8 Hz, *J* = 2.4 Hz, 1H), 1.91 – 1.79 (m, 1H), 1.77 – 1.69 (m, 1H), 1.68 – 1.55 (m, 1H), 1.40 (s, 3H), 1.39 (s, 3H), 1.38 – 1.23 (m, 1H), 1.15 – 0.90 (m, 1H), 0.81 (dd, *J* = 6.6 Hz, *J* = 2.3 Hz, 6H). **^{13}C NMR** (75 MHz, $CDCl_3$, 299 K) δ (ppm) = 148.9 (C), 128.6 (CH), 126.1 (CH), 126.1 (CH), 67.6 (CH), 52.7 (CH_2), 48.2 (CH_2), 37.3 (C), 30.7 (CH_3), 28.5 (CH_3), 24.6 (CH), 23.4 (CH_3), 22.2 (CH_3). **HRMS** (ESI) *m/z* = 243.1719 calcd. for $C_{15}H_{24}NaO^+$ $[M+Na]^+$, found: 243.1723.

2,2,6-Trimethylheptan-4-ol (1f)

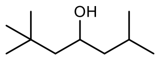

*i*BuLi (1.7 M in hexane, 1.07 equiv, 16.0 mmol, 9.41 mL) was dissolved in anhydrous THF (20 mL) under an atmosphere of argon and 3,3-dimethylbutyraldehyde (1.0 equiv, 15.0 mmol, 1.88 mL) was added over period of 1 hr at −78 °C. The mixture was stirred for 1 hr and was then allowed to warm to rt. The reaction was quenched by addition of water (30 mL). The aqueous layer was extracted with Et_2O (3 x 40 mL) and the combined organic layers were washed with brine (10 mL) and dried over $MgSO_4$. Upon removal of the solvent *in vacuo*, the desired alcohol was obtained as a colorless liquid (2.30 g, 14.5 mmol, 97%). **FT IR** (neat) ν (cm^{-1}): 3357, 2954, 2935, 2869, 1468, 1385, 1365, 1323, 1249, 1201, 1169, 1138, 1116, 1070, 1050, 1014, 967, 917, 862, 841. **^{1}H NMR** (300 MHz, $CDCl_3$, 299 K) δ (ppm) = 3.86 – 3.75 (m, 1H), 1.72 (dddd, *J* = 13.1 Hz, *J* = 12.1 Hz, *J* = 8.5 Hz, *J* = 6.5 Hz, 1H), 1.44 – 1.29 (m, 4H), 1.26 – 1.10 (m, 1H), 1.03 – 0.85 (m, 15H). **^{13}C NMR** (75 MHz, $CDCl_3$, 299 K) δ (ppm) = 67.8 (CH), 52.0 (CH_2), 49.1 (CH_2), 30.4 (C), 30.3 (CH_3), 24.8 (CH), 23.5 (CH_3), 22.3 (CH_3). **HRMS APCI** *m/z* = 157.15869 calcd. for $C_{10}H_{21}O^+$ $[M-H]^+$, found: 157.15879.

1-Cyclohexyl-3,3-dimethylbutan-2-ol (1g)

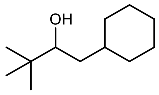

To a solution of bromomethylcyclohexane (0.9 equiv, 6.67 mmol, 0.93 mL) in anhydrous THF (10 mL) was added *t*BuLi (1.9 M in pentane, 1.8 equiv, 13.3 mmol, 7.02 mL) under an argon atmosphere over a period of 30 min at −78 °C. The solution was allowed to warm to rt and stirring was continued for 15 min. Upon cooling to −78 °C, pivaldehyde (1.0 equiv, 7.41 mmol, 0.82 mL) was added dropwise. The reaction mixture was allowed to warm to rt overnight and was then quenched by addition of saturated NH_4Cl solution (10 mL). The mixture was diluted with water (10 mL) and the aqueous layer extracted with Et_2O (5 x 30 mL). The combined organic layers were washed with brine (10 mL) and dried over $MgSO_4$. Upon removal of the solvent *in vacuo*, flash column chromatography (pentane/Et_2O 9:1) afforded the product as a colorless solid (423 mg, 2.30 mmol, 34%). **MP**.: 75 °C. **FT IR** (neat) ν (cm^{-1}): 3355, 2924, 2852, 1716, 1478, 1447, 1390, 1362, 1314, 1302, 1246, 1198, 1165, 1131, 1070,

1054, 1010, 992, 960, 932, 922, 908, 894, 879, 845, 831, 662. **^{1}H NMR** (400 MHz, $CDCl_3$, 299 K) δ (ppm) = 3.31 (dd, *J* = 10.3 Hz, *J* = 2.0 Hz, 1H), 1.91 – 1.80 (m, 1H), 1.75 – 1.61 (m, 4H), 1.52 – 1.40 (m, 1H), 1.36 – 1.08 (m, 6H), 1.04 – 0.92 (m, 1H), 0.90 – 0.71 (m, 10H). **^{13}C NMR** (101 MHz, $CDCl_3$, 299 K) δ (ppm) = 77.0 (CH), 39.6 (CH_2), 35.1 (C), 34.9 (CH_2), 34.6 (CH), 32.5 (CH_2), 26.8 (CH_2), 26.7 (CH_2), 26.3 (CH_2), 25.8 (CH_3). **HRMS** (ESI) *m/z* = 207.1719 calcd. for $C_{12}H_{24}NaO^+$ $[M+Na]^+$, found: 207.1723.

1-Cyclopentyl-3,3-dimethylbutan-2-ol (1h)

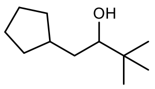

To a solution of cyclopentylacetaldehyde (1.0 equiv, 4.50 mmol, 505 mg) in anhydrous THF (10 mL) was added *t*BuLi (1.7 M in pentane, 0.995 equiv, 4.48 mmol, 2.63 mL) at −78 °C under an atmosphere of argon over a period of 30 min. Upon stirring at that temperature for 1 hr, the reaction mixture was allowed to warm to rt. Stirring was continued for 1 hr and the reaction was then quenched by addition of saturated NH_4Cl solution (10 mL). The aqueous layer was extracted with Et_2O (3 x 20 mL) and the combined organic layers washed with brine (10 mL) and dried over $MgSO_4$. Upon removal of the solvent *in vacuo*, flash column chromatography (pentane/Et_2O 20:1 → 9:1) afforded the product as a colorless solid (439 mg, 2.58 mmol, 57%). **MP**.: 55 °C. **FT IR** (neat) ν (cm^{-1}): 3319, 2948, 2937, 2915, 2867, 1478, 1451, 1390, 1362, 1325, 1309, 1189, 1147, 1095, 1069, 1009, 943, 891, 764. **^{1}H NMR** (300 MHz, $CDCl_3$, 299 K) δ (ppm) = 3.29 – 3.20 (m, 1H), 2.11 – 1.89 (m, 1H), 1.89 – 1.75 (m, 2H), 1.69 – 1.47 (m, 4H), 1.43 – 1.36 (m, 2H), 1.34 – 1.25 (brs, 1H), 1.21 – 0.98 (m, 2H), 0.89 (s, 9H). **^{13}C NMR** (75 MHz, $CDCl_3$, 299 K) δ (ppm) = 79.3 (CH), 38.1 (CH_2), 37.5 (CH), 35.0 (C), 33.9 (CH_2), 32.1 (CH_2), 25.8 (CH_3), 25.3 (CH_2), 25.2 (CH_2). **HRMS** (ESI) *m/z* = 193.1563 calcd. for $C_{11}H_{22}NaO^+$ $[M+Na]^+$, found: 193.1576. The analytical data are in accordance to those reported in the literature (*18*).

1-Cyclobutyl-3,3-dimethylbutan-2-ol (1i)

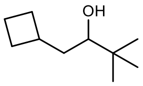

To a slurry of magnesium turnings (1.09 equiv, 12.0 mmol, 292 mg) in anhydrous THF (7.0 mL) was added a drop of 1,2-dibromoethane under an atmosphere of argon followed by (bromomethyl)cyclobutane (1.0 equiv, 11.0 mmol, 1.24 mL) while maintaining constant self reflux of the reaction mixture. After complete addition, the mixture was heated to 70 °C for 1 hr. Pivaldehyde (1.0 equiv, 11.0 mmol, 1.20 mL) in anhydrous THF (4.0 mL) was added over a period of 30 min at 0 °C. The reaction mixture was allowed to warm to rt and heated at 60 °C for 1 hr. Upon cooling to rt, the reaction was quenched by addition of saturated NH_4Cl solution (10 mL). The aqueous layer was extracted with Et_2O (3 x 50 mL) and the combined organic layers washed with brine (10 mL) and dried over $MgSO_4$. Upon removal of the solvent *in vacuo*, flash column chromatography (pentane/Et_2O 20:1) afforded the product as a colorless solid (650 mg, 4.16 mmol, 38%). **MP**.: 40 °C. **FT IR** (neat) ν (cm^{-1}): 3382, 2954, 2935, 2912, 2867, 1480, 1468, 1443, 1393, 1363, 1308, 1285, 1238, 1187, 1127, 1079, 1046, 1006, 987, 932, 914, 890, 842, 751. **^{1}H NMR** (300 MHz, $CDCl_3$, 299 K) δ (ppm) = 3.12 (dd, *J* = 10.3 Hz, *J* = 1.9 Hz, 1H), 2.61 – 2.38 (m, 1H), 2.18 – 1.99 (m, 2H), 1.98 – 1.73 (m, 2H), 1.73 – 1.48 (m, 3H), 1.46 – 1.30 (m, 2H), 0.93 – 0.83 (m, 9H). **^{13}C NMR** (75 MHz, $CDCl_3$, 299 K) δ (ppm) = 78.4 (CH), 38.9 (CH_2), 34.8 (C), 33.8 (CH), 29.1 (CH_2), 28.2 (CH_2), 25.8 (CH_3), 18.8 (CH_2). **HRMS** (ESI) *m/z* = 179.1406 calcd. for $C_{10}H_{20}NaO^+$ $[M+Na]^+$, found: 179.1415.

5-Ethyl-2,2-dimethylheptan-3-ol (1j)

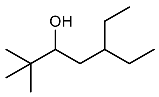

To a slurry of magnesium turnings (1.3 equiv, 14.3 mmol, 348 mg) in anhydrous THF (2.0 mL) was added a drop of 1,2-dibromoethane under an atmosphere of argon followed by a solution of 2-bromo-2-ethylpentane (1.2 equiv, 13.2 mmol, 1.85 mL) in anhydrous THF (5.0 mL) while maintaining constant self reflux of the reaction mixture. After 2 hrs, a solution of pivaldehyde (1.0 equiv, 11.0 mmol, 1.20 mL) in anhydrous THF (10 mL) was added over a period of 1 hr at 0 °C. The reaction mixture was allowed to warm to rt over night and then quenched by addition of saturated NH_4Cl solution (10 mL). The aqueous layer was extracted with Et_2O (3 x 50 mL) and the combined organic layers washed with brine (10 mL) and dried over $MgSO_4$. Upon removal of the solvent *in vacuo*, flash column chromatography (pentane/Et_2O 20:1) afforded the product as a colorless liquid (243 mg, 1.41 mmol, 13%). **FT IR** (neat) ν (cm^{-1}): 3428, 2961, 2940, 2920, 2875, 1479, 1461, 1381, 1364, 1299, 1245, 1190, 1108, 1073, 999, 961, 920, 888, 863, 814, 771. **^{1}H NMR** (400 MHz, $CDCl_3$, 299 K) δ (ppm) = 3.27 (dd, *J* = 10.3 Hz, *J* = 1.8 Hz, 1H), 1.53 – 1.17 (m, 8H), 0.95 – 0.79 (m, 15H). **^{13}C NMR** (101 MHz, $CDCl_3$, 299 K) δ (ppm) = 77.7 (CH), 37.2 (CH), 35.3 (CH_2), 35.1 (C), 26.6 (CH_2), 25.8 (CH_3), 24.4 (CH_2), 11.3 (CH_3), 10.2 (CH_3). **HRMS** (ESI) *m/z* = 195.1719 calcd. for $C_{11}H_{24}NaO^+$ $[M+Na]^+$, found: 195.1726.

***trans*-2-Isopropylcyclohexan-1-ol (1k)**

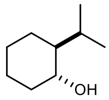

Following a procedure by *Linstrumelle and coworkers* (*7*), CuI (0.15 equiv, 3.00 mmol, 571 mg) in anhydrous THF (25 mL) was treated with *i*PrMgCl (2.0 M in THF, 1.5 equiv, 30.0 mmol, 15,0 mL) at −30 °C under an atmosphere of argon. After 5 min, cyclohexene oxide (1.0 equiv, 20.0 mmol, 2.02 mL) in anhydrous THF (5.0 mL) was added over a period of 30 min. The mixture was stirred for 1 hr and was then allowed to warm to rt and stirring was continued for 18 hrs. After quenching by addition of saturated NH_4Cl solution (30 mL), the aqueous layer was extracted with Et_2O (4 x 50 mL). The combined organic layers were washed with brine (10 mL) and dried over $MgSO_4$. Fractional distillation afforded the alcohol (1.36 g) in 91% purity (by GC). Therefore, this mixture was used without any further purification.

Dicyclohexylmethanol (1m)

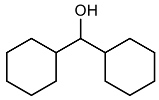

Cyclohexylmagnesium chloride (1.3 M in THF/toluene 1:1, 1.2 equiv, 18.0 mmol, 13.9 mL) was dissolved in anhydrous THF (14 mL) under an atmosphere of argon and cyclohexyl carboxaldehyde (1.0 equiv, 15.0 mmol, 1.82 mL) was added over period of 30 min. The mixture was stirred at rt for 2 hrs and stirring was continued at 50 °C for 4 hrs. Upon cooling to rt, the reaction was quenched by addition of saturated NH_4Cl solution (20 mL). The aqueous layer was extracted with Et_2O (4 x 30 mL) and the combined organic layers were washed with saturated $NaHCO_3$ solution and brine (10 mL each) and dried over $MgSO_4$. Upon removal of the solvent *in vacuo*, the desired alcohol was obtained by flash column chromatography (pentane/Et_2O 20:1 → 9:1) as a colorless solid (2.36 g, 12.0 mmol, 80%). **MP**.: 66 °C. **FT IR** (neat) ν (cm^{-1}): 3318, 2920, 2850, 1447, 1401, 1333, 1313, 1297, 1260, 1215, 1195, 11701149, 1127, 1104, 1087, 1072, 1051, 986, 936, 890, 847, 869, 690, 661, 628. **^{1}H NMR** (300 MHz, $CDCl_3$, 299 K) δ (ppm) = 3.05 (t, *J* = 5.7 Hz, 1H), 1.92 – 1.36 (m, 12H), 1.36 – 0.93 (m, 10H). **^{13}C NMR** (75 MHz, $CDCl_3$, 299 K) δ (ppm) = 80.6 (CH), 40.1 (CH), 30.2 (CH_2), 27.5 (CH_2), 26.7 (CH_2), 26.7 (CH_2), 26.4 (CH_2). **HRMS** (ESI) *m/z* = 219.1719 calcd. for $C_{13}H_{24}NaO^+$ $[M+Na]^+$, found: 219.1729.

1-Cyclohexyl-2,2-dimethylpropan-1-ol (1n)

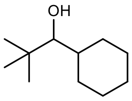

Cyclohexylmagnesium chloride (1.3 M in THF/toluene 1:1, 1.1 equiv, 12.1 mmol, 9.31 mL) was dissolved in anhydrous Et_2O (20 mL) under an atmosphere of argon and pivaldehyde (1.0 equiv, 11.0 mmol, 1.20 mL) was added over period of 15 min. After stirring for 4 hrs, the reaction was quenched by addition of saturated NH_4Cl solution (10 mL). The aqueous layer was extracted with Et_2O (3 x 50 mL) and the combined organic layers were washed with brine (10 mL) followed by drying over $MgSO_4$. Removal of the solvent *in vacuo* followed by flash column chromatography (pentane/Et_2O 9:1) afforded the desired alcohol as a colorless liquid (590 mg, 3.47 mmol, 32%). **FT IR** (neat) ν (cm^{-1}): 3454, 2954, 2921, 2852, 1479, 1449, 1395, 1363, 1309, 1263, 1195, 1098, 1085, 1052, 983, 896, 843, 759. **^{1}H NMR** (400 MHz, $CDCl_3$, 299 K) δ (ppm) = 3.02 (d, *J* = 2.3 Hz, 1H), 1.82 – 1.69 (m, 3H), 1.69 – 1.49 (m, 3H), 1.49 – 1.07 (m, 6H), 0.93 (s, 9H). **^{13}C NMR** (101 MHz, $CDCl_3$, 299 K) δ (ppm) = 84.0 (CH), 39.5 (CH), 36.1 (C), 34.0 (CH_2), 27.4 (CH_2), 27.1 (CH_2), 26.8 (CH_3), 26.6 (CH_2), 26.5 (CH_2). **HRMS** (ESI) *m/z* = 193.1563 calcd. for $C_{13}H_{24}NaO^+$ $[M+Na]^+$, found: 193.1585.

2,2-Dimethylnonan-3-ol (1o)

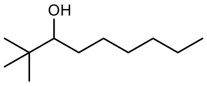

To a solution of pivaldehyde (1.0 equiv, 15.0 mmol, 1.63 mL) in anhydrous THF (20 mL) was added *n*Hexyllithium (2.5 M in hexane, 1.1 equiv, 16.5 mmol, 6.60 mL) over a period of 1 hr at −78 °C under an atmosphere of argon. Stirring was continued for 1 hr. The reaction mixture was then allowed to warm to rt and stirring was continued for 2 hrs. The reaction was quenched by addition of saturated NH_4Cl solution (10 mL). The aqueous layer was extracted with Et_2O (5 x 10 mL) and the combined organic layers were washed with brine (10 mL) followed by drying over $MgSO_4$. Removal of the solvent *in vacuo* followed by flash column chromatography (pentane/Et_2O 9:1) afforded the desired alcohol as a colorless liquid (2.20 g, 12.8 mmol, 85%). **FT IR** (neat) ν (cm^{-1}): 3391, 2955, 2926, 2858, 1467, 1393, 1363, 1313, 1286, 1248, 1190, 1119, 1074, 1040, 1009, 956, 932, 894, 860, 764, 723. **^{1}H NMR** (300 MHz, $CDCl_3$, 299 K) δ (ppm) = 3.18 (dd, *J* = 9.8 Hz, *J* = 1.6 Hz, 1H), 1.64 – 1.44 (m, 2H), 1.44 – 1.19 (m, 9H), 0.96 – 0.83 (m, 12H). **^{13}C NMR** (75 MHz, $CDCl_3$, 299 K) δ (ppm) = 80.2 (CH), 35.1 (C), 32.1 (CH_2), 31.7 (CH_2), 29.6 (CH_2), 27.2 (CH_2), 25.9 (CH_3), 22.8 (CH_2), 14.2 (CH_3). **HRMS** (ESI) *m/z* = 195.1719 calcd. for $C_{11}H_{24}NaO^+$ $[M+Na]^+$, found: 195.1716. The analytical data are in accordance to those reported in the literature (*8*).

2,2,6-Trimethylheptan-3-ol (1p)

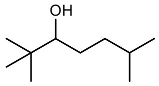

To a slurry of magnesium turnings (1.3 equiv, 14.3 mmol, 348 mg) in anhydrous THF (2.0 mL) was added a drop of 1,2-dibromoethane under an atmosphere of argon followed by a solution of 1-bromo-3-methylbutane (1.2 equiv, 13.2 mmol, 1.58 mL) in anhydrous THF (5.0 mL) while maintaining constant self reflux of the reaction mixture. After 2 hrs, a solution of pivaldehyde (1.0 equiv, 11.0 mmol, 1.20 mL) in anhydrous THF (10 mL) was added over a period of 1 hr at 0 °C. The reaction mixture was allowed to warm to rt over night and then quenched by addition of saturated NH_4Cl solution (10 mL). The aqueous layer was extracted with Et_2O (3 x 50 mL) and the combined organic layers washed with brine (10 mL) and dried over $MgSO_4$. Upon removal of the solvent *in vacuo*, flash column chromatography (pentane/Et_2O 20:1) afforded the product as a colorless liquid (659 mg, 14.16 mmol, 38%). **FT IR** (neat) ν (cm^{-1}): 3405, 2953, 2906, 2870, 1468, 1385, 1366, 1312, 1280, 1245, 1190, 1122, 1072, 1042, 1007, 956, 916, 877, 769. **^{1}H NMR** (300 MHz, $CDCl_3$, 299 K) δ (ppm) = 3.20 – 3.09 (m, 1H), 1.65 – 1.35 (m, 4H), 1.30 – 1.09 (m, 2H), 0.99 – 0.84 (m, 15H). **^{13}C NMR** (75 MHz, $CDCl_3$, 299 K) δ (ppm) = 80.5 (CH), 36.6 (CH_2), 35.2 (C), 29.5 (CH_2), 28.3 (CH), 25.9 (CH_3), 23.1 (CH_3), 22.6 (CH_3). **HRMS** (ESI) *m/z* = 181.1563 calcd. for $C_{10}H_{22}NaO^+$ $[M+Na]^+$, found: 181.1565.

1-Cyclohexyl-4,4-dimethylpentan-3-ol (1q)

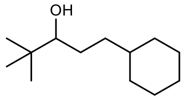

To a slurry of magnesium turnings (1.3 equiv, 5.67 mmol, 138 mg) in anhydrous THF (2.0 mL) was added a drop of 1,2-dibromoethane under an atmosphere of argon followed by a solution of (2-bromoethyl)cyclohexane (1.2 equiv, 5.23 mmol, 1.0 g) in anhydrous THF (5.0 mL) while maintaining constant self reflux of the reaction mixture. After 2 hrs, a solution of pivaldehyde (1.0 equiv, 4.36 mmol, 0.50 mL) in anhydrous THF (10 mL) was added over a period of 1 hr at 0 °C. The reaction mixture was allowed to warm to rt over night and then quenched by addition of saturated NH_4Cl solution (10 mL). The aqueous layer was extracted with Et_2O (3 x 50 mL) and the combined organic layers washed with brine (10 mL) and dried over $MgSO_4$. Upon removal of the solvent *in vacuo*, flash column chromatography (pentane/Et_2O 9:1) afforded the product as a colorless solid (404 mg, 2.04 mmol, 47%). **MP**.: 43 °C. **FT IR** (neat) ν (cm^{-1}): 3324, 2918, 2849, 1478, 1448, 1390, 1363, 1329, 1309, 1261, 1199, 1171, 1131, 1074, 1009, 942, 931, 889, 755. **^{1}H NMR** (300 MHz, $CDCl_3$, 299 K) δ (ppm) = 3.20 – 3.08 (m, 1H), 1.80 – 1.34 (m, 8H), 1.32 – 1.07 (m, 6H), 0.98 – 0.81 (m, 11H). **^{13}C NMR** (75 MHz, $CDCl_3$, 299 K) δ (ppm) = 80.5 (CH), 38.0 (CH), 35.2 (C), 35.0 (CH_2), 33.9 (CH_2), 33.4 (CH_2), 29.0 (CH_2), 26.9 (CH_2), 26.6 (CH_2), 26.6 (CH_2), 25.9 (CH_3). **HRMS** (ESI) *m/z* = 221.1876 calcd. for $C_{13}H_{26}NaO^+$ $[M+Na]^+$, found: 221.1869.

1-((1*R*,2*R*,4*R*)-2-Hydroxy-4-methylcyclohexyl)ethan-1-one (1r)
1-((1*R*,2*R*,4*R*)-2-Hydroxy-4-methylcyclohexyl)ethan-1-one (S-20)

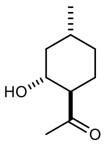

Ketone **S-20** was prepared following a slightly modified procedure by *Thomas and coworkers* (*9*). Ozone was bubbled through a stirred solution of (−)-isopulegol (1.0 equiv, 20.0 mmol, 3.4 mL) in MeOH (200 mL) at −78 °C, until the blueish color of ozone in MeOH persisted. Excess of ozone was discharged by bubbling oxygen through the solution for 10 min. Afterwards, Me_2S (5.0 equiv, 0.10 mol, 7.3 mL) was added and the solution was allowed to warm to rt. The

solution was concentrated *in vacuo* and the product was isolated by flash column chromatography (pentane/Et_2O 4:1 → 1:1) as a colorless solid (1.82 g, 11.7 mmol, 58%). **MP.**: 43 °C. **^{1}H NMR** (300 MHz, $CDCl_3$, 299 K) δ (ppm) = 3.82 (ddd, *J* = 11.3 Hz, *J* = 9.6 Hz, *J* = 4.3 Hz, 1H), 2.50 (brs, 1H), 2.29 (ddd, *J* = 13.1 Hz, *J* = 9.7 Hz, *J* = 3.7 Hz, 1H), 2.23 – 2.11 (m, 3H), 2.06 – 1.90 (m, 2H), 1.78 – 1.68 (m, 1H), 1.47 (tdq, *J* = 12.7 Hz, *J* = 6.5 Hz, *J* = 3.3 Hz, 1H), 1.26 (qd, *J* = 13.0 Hz, *J* = 3.5 Hz, 1H), 1.09 – 0.86 (m, 5H). **^{13}C NMR** (75 MHz, $CDCl_3$, 299 K) δ (ppm) = 213.0 (C), 70.6 (CH), 58.6 (CH), 42.4 (CH_2), 34.1 (CH_2), 31.2 (CH), 29.3 (CH_3), 27.7 (CH_2), 22.1 (CH_3). **HRMS** (ESI) *m/z* = 179.1043 calcd. for $C_9H_{16}NaO^+$ $[M+Na]^+$, found: 179.1050. **OR.**: $[\alpha]_D^{20}$ = −24.00 (c = 0.56 in $CHCl_3$). The analytical data are in accordance with those reported in the literature (*10*).

1-((1*R*,2*R*,4*R*)-2-Hydroxy-4-methylcyclohexyl)ethan-1-one (1r)

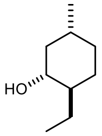

Alcohol **1q** was prepared following a modified literature known procedure (*2*). **S-20** (1.0 equiv, 5.40 mmol, 844 mg) was mixed with hydrazine mono hydrate (3.0 equiv, 16.2 mmol, 0.79 mL) and KOH powder (4.0 equiv, 21.6 mmol, 1.21 g) and heated in triethylene glycol (15 mL) at 195 °C for 6 hrs. After cooling to rt, water (15 mL) was added and the aqueous layer was extracted with Et_2O (3 x 20 mL). After removal of the solvent *in vacuo*, the desired alcohol was obtained by flash column chromatography (pentane/Et_2O 9:1) as a colorless liquid (352 mg, 2.48 mmol, 50%). **FT IR** (neat) ν (cm^{-1}): 3331, 2951, 2916, 2868, 1706, 1456, 1447, 1375, 1351, 1307, 1270, 1258, 1222, 1175, 1108, 1072, 1061, 1039, 1015, 982, 947, 920, 904, 845, 757. **^{1}H NMR** (300 MHz, $CDCl_3$, 299 K) δ (ppm) = 3.23 (ddd, *J* = 11.0 Hz, *J* = 9.2 Hz, *J* = 4.3 Hz, 1H), 1.93 (dtd, *J* = 12.2 Hz, *J* = 3.9 Hz, *J* = 2.1 Hz, 1H), 1.87 – 1.75 (m, 2H), 1.67 – 1.58 (m, 1H), 1.53 – 1.35 (m, 2H), 1.23 – 1.01 (m, 2H), 0.98 – 0.77 (m, 9H). **^{13}C NMR** (75 MHz, $CDCl_3$, 299 K) δ (ppm) = 74.3 (CH), 46.3 (CH), 44.8 (CH_2), 34.6 (CH_2), 31.8 (CH), 29.4 (CH_2), 24.7 (CH_3), 22.3 (CH_3), 11.0 (CH_3). **HRMS** (ESI) *m/z* = 165.1050 calcd. for $C_9H_{18}NaO^+$ $[M+Na]^+$, found: 165.1237. **OR.**: $[\alpha]_D^{20}$ = −24.50 (c = 1.5 in $CHCl_3$).

1,1,1-Trichloro-4-methylpentan-2-ol (1s)

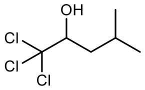

Following a procedure by *Confalone and coworkers* (*11*), trichloroacetic acid (1.5 equiv, 15.0 mmol, 2.45 g) and sodium trichloroacetate (1.5 equiv, 15.0 mmol, 2.78 g) were dissolved in anhydrous DMF (7.0 mL) and isovaleraldehyde (1.0 equiv, 10.0 mmol, 1.08 mL) was added. After stirring for 4 hrs at rt, water (70 mL) was added and the aqueous layer was extracted with pentane (4 x 200 mL). The combined organic layers were washed with water and brine (20 mL each), dried over $MgSO_4$ and concentrated *in vacuo*. The residue was purified by flash column chromatography (pentane/Et_2O 9:1) as a slightly yellow liquid (1.10 g, 5.37 mmol, 54%). **FT IR** (neat) ν (cm^{-1}): 3406, 2960, 2931, 2873, 1711, 1468, 1388, 1370, 1297, 1219, 1170, 1142, 1087, 1025, 978, 952, 858, 836, 806, 764, 632. **^{1}H NMR** (300 MHz, $CDCl_3$, 299 K) δ (ppm) = 4.07 (dd, *J* = 9.6 Hz, *J* = 2.0 Hz, 1H), 1.92 (dddd, *J* = 13.0 Hz, *J* = 10.4, *J* = 6.6 Hz, *J* = 4.0 Hz, 1H), 1.78 (ddd, *J* = 13.8 Hz, *J* = 9.8 Hz, *J* = 2.0 Hz, 1H), 1.63 (ddd, *J* = 13.8 Hz, *J* = 9.6 Hz, *J* = 4.1 Hz, 1H), 0.99 (dd, *J* = 10.3 Hz, *J* = 6.6 Hz, 6H). **^{13}C NMR** (75 MHz, $CDCl_3$, 299 K) δ (ppm) = 104.6 (C), 81.4 (CH), 40.4 (CH_2), 25.0 (CH), 23.6 (CH_3), 21.4 (CH_3). **HRMS** (ESI) *m/z* = 248.9858 calcd. for $C_7H_{12}Cl_3O_3$ $[M+HCO_2^-]^-$, found: 248.9866. The analytical data are in accordance with those reported in the literature (*12*).

1,1,1-Trichloro-3-cyclopentylpropan-2-ol (1t)

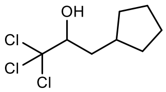

Following a procedure by *Confalone and coworkers* (*11*), trichloroacetic acid (1.5 equiv, 5.22 mmol, 852 mg) and sodium trichloroacetate (1.5 equiv, 5.22 mmol, 967 mg) were dissolved in anhydrous DMF (2.5 mL) and 2-cyclopentylacetaldehyde (1.0 equiv, 3.48 mmol, 390 mg) was added. After stirring for 4 hrs at rt, water (20 mL) was added and the aqueous layer was extracted with pentane (5 x 70 mL). The combined organic layers were washed with water and brine (10 mL each), dried over $MgSO_4$ and concentrated *in vacuo*. The residue was purified by flash column chromatography (pentane/Et_2O 19:1) as a colorless solid (613 mg, 2.65 mmol, 76%). **MP.**: 50 °C. **FT IR** (neat) ν (cm^{-1}): 3305, 2953, 2858, 1568, 1450, 1395, 11367, 1330, 1306, 1255, 1179, 1154, 1087, 1036, 1018, 986, 928, 865, 807, 764, 644, 592. **^{1}H NMR** (300 MHz, $CDCl_3$, 299 K) δ (ppm) = 4.10 – 3.99 (m, 1H), 2.69 (d, J = 5.3 Hz, 1H), 2.25 – 2.02 (m, 1H), 2.02 – 1.46 (m, 8H), 1.32 – 1.05 (m, 2H). **^{13}C NMR** (75 MHz, $CDCl_3$, 299 K) δ (ppm) = 104.7 (C), 82.7 (CH), 37.9 (CH_2), 36.9 (CH), 33.5 (CH_2), 32.1 (CH_2), 25.2 (CH_2), 25.1 (CH_2). **HRMS** (ESI) *m/z* = 275.0014 calcd. for $C_9H_{14}Cl_3O_3$ $[M+HCO_2^-]^-$, found: 275.0010.

1,1,1-Trichloroheptan-2-ol (1u)

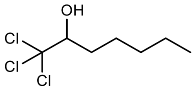

Following a procedure by *Confalone and coworkers* (*11*), trichloroacetic acid (1.5 equiv, 15.0 mmol, 2.45 g) and sodium trichloroacetate (1.5 equiv, 15.0 mmol, 2.78 g) were dissolved in anhydrous DMF (7.0 mL) and hexanal (1.0 equiv, 10.0 mmol, 1.24 mL) was added. After stirring for 4 hrs at rt, water (70 mL) was added and the aqueous layer was extracted with pentane (4 x 200 mL). The combined organic layers were washed with water and brine (20 mL each), dried over $MgSO_4$ and concentrated *in vacuo*. The residue was purified by flash column chromatography (pentane/Et_2O 9:1) as a slightly yellow liquid (1.43 g, 6.52 mmol, 65%). **FT IR** (neat) ν (cm^{-1}): 3390, 2958, 2930, 2862, 1467, 1380, 1297, 1258, 1188, 1131, 1086, 1040, 1000, 928, 808, 781, 727, 631. **^{1}H NMR** (300 MHz, $CDCl_3$, 299 K) δ (ppm) = 3.99 (dd, J = 9.4 Hz, J = 2.0 Hz, 1H), 2.78 (brs, 1H), 2.11 – 1.93 (m, 1H), 1.73 – 1.54 (m, 2H), 1.53 – 1.21 (m, 5H), 1.00 – 0.82 (m, 3H). **^{13}C NMR** (75 MHz, $CDCl_3$, 299 K) δ (ppm) = 104.4 (C), 83.0 (CH), 31.5 (CH_2), 31.5 (CH_2), 25.8 (CH_2), 22.5 (CH_2), 14.0 (CH_3). **HRMS** (ESI) *m/z* = 275.0014 calcd. for $C_8H_{14}Cl_3O_3$ $[M+HCO_2^-]^-$, found: 263.0027. The analytical data are in accordance with those reported in the literature (*12*).

Methyl 3-hydroxy-2,2,5-trimethylhexanoate (1v)

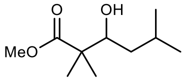

1v was prepared by a modified procedure by *Newcomb and coworkers* (*13*). Diisopropylamine (1.25 equiv, 18.8 mmol, 2.65) was dissolved in anhydrous THF (60 mL) under argon and *n*BuLi (1.6 M in hexane, 1.20 equiv, 18.0 mmol, 11.3 mL) was added over a period of 10 min at −78 °C. The solution was stirred for 30 min and methyl isobutyrate (1.00 equiv, 15.0 mmol, 1.72 mL, dissolved in 4 mL THF) was added over 10 min. Stirring was continued for 30 min followed by addition of isobutyraldehyde (1.10 equiv, 16.5 mmol, 1.78 mL, dissolved in 4 mL THF) over a period of 10 min. The solution was allowed to warm to room temperature and stirring was continued for 12 hrs. The reaction was quenched by addition of sat. NH_4Cl solution (10 mL) at 0 °C. After the aqueous layer was extracted with Et_2O (3 x 50 mL), the combined organic layers were washed with brine (10 mL) and dried over $MgSO_4$. The removal of solvent *in vacuo* yielded the pure aldol (2.40 g, 12.8 mmol, 85%) as a colorless

liquid which was used without any further purification. **FT IR** (neat) ν (cm^{-1}): 3501, 2955, 2872, 1718, 1468, 1435, 1387, 1368, 1261, 1192, 1138, 1109, 1072, 1015, 993, 952, 870, 844, 794, 769. **^{1}H NMR** (300 MHz, $CDCl_3$, 299 K) δ (ppm) = 3.68 (s, 3H), 2.37 (brs, 1H), 1.95 – 1.72 (m, 1H), 1.40 – 1.20 (m, 1H), 1.21 – 1.03 (m, 6H), 1.01 – 0.80 (m, 6H). **^{13}C NMR** (75 MHz, $CDCl_3$, 299 K) δ (ppm) = 178.4 (C), 74.8 (CH), 52.0 (CH_3), 47.3 (C), 41.1 (CH_2), 24.9 (CH), 24.2 (CH_3), 22.3 (CH_3), 21.5 (CH_3), 20.5 (CH_3). **HRMS** (ESI) *m/z* = 211.1305 calcd. for $C_{10}H_{20}NaO_3^+$ [M+Na]$^+$, found: 211.1305.

Methyl 3-hydroxy-2,2-dimethyloctanoate (1w)

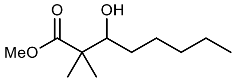

1w was prepared by a modified procedure by *Newcomb and coworkers* (*13*). Diisopropylamine (1.25 equiv, 18.8 mmol, 2.65) was dissolved in anhydrous THF (60 mL) under argon and *n*BuLi (1.6 M in hexane, 1.20 equiv, 18.0 mmol, 11.3 mL) was added over a period of 10 min at −78 °C. The solution was stirred for 30 min and methyl isobutyrate (1.00 equiv, 15.0 mmol, 1.72 mL, dissolved in 4 mL THF) was added over 10 min. Stirring was continued for 30 min followed by addition of hexanal (1.10 equiv, 16.5 mmol, 2.04 mL, dissolved in 4 mL THF) over a period of 10 min. The solution was allowed to warm to room temperature and stirring was continued for 12 hrs. The reaction was quenched by addition of sat. NH_4Cl solution (10 mL) at 0 °C. After the aqueous layer was extracted with Et_2O (3 x 50 mL), the combined organic layers were washed with brine (10 mL) and dried over $MgSO_4$. The removal of solvent *in vacuo* yielded the pure aldol (2.95 g, 14.6 mmol, 97%) as a colorless liquid which was used without any further purification. **FT IR** (neat) ν (cm^{-1}): 3483, 2952, 2932, 2860, 1715, 1468, 1435, 1389, 1366, 1264, 1192, 1140, 1117, 1076, 1008, 995, 945, 928, 862, 798,772, 727, 680, 609. **^{1}H NMR** (300 MHz, $CDCl_3$, 299 K) δ (ppm) = 3.69 – 3.68 (m, 3H), 3.64 – 3.54 (m, 1H), 2.37 (brd, *J* = 6.8 Hz, 1H), 1.65 – 1.51 (m, 1H), 1.47 – 1.12 (m, 13H), 0.95 – 0.84 (m, 3H). **^{13}C NMR** (75 MHz, $CDCl_3$, 299 K) δ (ppm) = 178.4 (C), 76.9 (CH), 52.0 (CH_3), 47.4 (C), 31.9 (CH_2), 31.8 (CH_2), 26.5 (CH_2), 22.8 (CH_2), 22.4 (CH_3), 20.5 (CH_3), 14.2 (CH_3). **HRMS** (ESI) *m/z* = 225.1461 calcd. for $C_{11}H_{22}NaO_3^+$ [M+Na]$^+$, found: 225.1481.

***rac* Methyl (2*R*,3*S*)-3-hydroxy-2-methoxy-2,5-dimethylhexanoate (1x)**

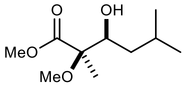

1x was prepared by a modified procedure by *Newcomb and coworkers* (*13*). Diisopropylamine (1.25 equiv, 18.8 mmol, 2.65) was dissolved in anhydrous THF (60 mL) under argon and *n*BuLi (1.6 M in hexane, 1.20 equiv, 18.0 mmol, 11.3 mL) was added over a period of 10 min at −78 °C. The solution was stirred for 30 min and methyl-2-methoxypropionate (1.00 equiv, 15.0 mmol, 1.77 mL, dissolved in 4 mL THF) was added over 10 min. Stirring was continued for 30 min followed by addition of isobutyraldehyde (1.10 equiv, 16.5 mmol, 1.78 mL, dissolved in 4 mL THF) over a period of 10 min. The solution was allowed to warm to room temperature and stirring was continued for 12 hrs. The reaction was quenched by addition of sat. NH_4Cl solution (10 mL) at 0 °C. After the aqueous layer was extracted with EtOAc (3 x 50 mL), the combined organic layers were washed with brine (10 mL) and dried over $MgSO_4$. After the removal of the solvent *in vacuo*, flash column chromatography (pentane/Et_2O 4:1) yielded the pure aldol (2.48 g, 12.1 mmol, 81%, *dr*: 7:1) as a colorless liquid. **FT IR** (neat) ν (cm^{-1}): 3484, 2953, 2870, 2834, 1735, 1457, 1369, 1257, 1221, 1185, 1170, 1133, 1097, 1076, 1048, 987, 918, 880, 848, 803, 772, 706, 663. **^{1}H NMR** (300 MHz, $CDCl_3$, 299 K) δ (ppm) = 3.86 – 3.71 (m, 4H), 3.37 – 3.28 (m,

3H), 2.39 (brs, 1H), 1.92 – 1.72 (m, 1H), 1.45 – 1.30 (m, 4H), 1.20 – 0.99 (m, 1H), 0.97 – 0.84 (m, 6H). **^{13}C NMR** (75 MHz, $CDCl_3$, 299 K, major isomer) δ (ppm) = 173.7 (C), 83.0 (C), 74.0 (CH), 52.5 (CH_3), 52.1 (CH_3), 40.3 (CH_2), 24.8 (CH), 24.1 (CH_3), 21.5 (CH_3), 16.9 (CH_3). **HRMS** (ESI) *m/z* = 227.1254 calcd. for $C_{10}H_{20}NaO_4^+$ $[M+Na]^+$, found: 227.1267.

rac **Methyl (2*R*,3*S*)-3-hydroxy-2,5-dimethylhexanoate (1y)**

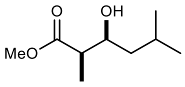

Aldol **1y** was prepared by a modified procedure by *Newcomb and coworkers* (*13*). Diisopropylamine (1.25 equiv, 18.8 mmol, 2.65) was dissolved in anhydrous THF (60 mL) under argon and *n*BuLi (1.6 M in hexane, 1.20 equiv, 18.0 mmol, 11.3 mL) was added over a period of 10 min at –78 °C. The solution was stirred for 30 min and methylpropionate (1.00 equiv, 15.0 mmol, 1.45 mL, dissolved in 4 mL THF) was added over 10 min. Stirring was continued for 30 min followed by addition of isobutyraldehyde (1.10 equiv, 16.5 mmol, 1.78 mL, dissolved in 4 mL THF) over a period of 10 min. The solution was allowed to warm to room temperature and stirring was continued for 12 hrs. The reaction was quenched by addition of sat. NH_4Cl solution (10 mL) at 0 °C. After the aqueous layer was extracted with Et_2O (3 x 50 mL), the combined organic layers were washed with brine (10 mL) and dried over $MgSO_4$. Removal of the solvent *in vacuo* yielded a mixture of two diasteromers (2.28 g, 13.1 mmol, 87%, *dr*: 3:2) as a colorless liquid that was used without any further purification. **^{1}H NMR** (300 MHz, $CDCl_3$, 299 K, major isomer) δ (ppm) = 3.96 (dt, *J* = 9.5 Hz, *J* = 3.6 Hz, 1H), 3.75 – 3.63 (m, 6H), 2.54 – 2.42 (m, 3H), 1.90 – 1.68 (m, 2H), 1.49 – 1.29 (m, 2H), 1.25 – 1.04 (m, 7H), 0.96 – 0.86 (m, 13H). **^{13}C NMR** (75 MHz, $CDCl_3$, 299 K, major isomer) δ (ppm) = 176.7 (C), 69.9 (CH), 51.8 (C), 44.8 (CH), 43.0 (CH_2), 24.8 (CH), 23.6 (CH_3), 22.0 (CH_3), 10.8 (CH_3). **HRMS** (ESI) *m/z* = 197.1148 calcd. for $C_9H_{18}NaO_3^+$ $[M+Na]^+$, found: 197.1150. The analytical data are in accordance with those reported in the literature (*14*).

Ethyl 2,2-difluoro-3-hydroxy-5-methylhexanoate (1z)

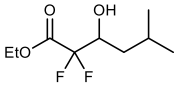

1z was prepared following a modified procedure by *Sorensen and coworkers* (*15*). Under argon, anhydrous THF (70 mL) was heated to 70 °C and activated zinc dust (2.0 equiv, 15.0 mmol, 981 mg), isovaleraldehyde (1.0 equiv, 7.5 mmol, 0.81 mL) and ethyl bromodifluoroacetate (2.0 equiv, 15.0 mmol, 1.92 mL) was added. After the strongly exothermic reaction occurred, the mixture was refluxed for 1 hr and was then allowed to cool down to room temperature overnight. The reaction was quenched by the addition of sat. NH_4Cl solution (10 mL). The aqueous layer was extracted with Et_2O (3 x 50 mL) and the combined organic layers washed with brine (10 mL) followed by drying over $MgSO_4$. Upon removal of the solvent *in vacuo*, the desired aldol was obtained by flash column chromatography (pentane/Et_2O 9:1) as a colorless liquid (1.01 g, 4.81 mmol, 64%). **FT IR** (neat) ν (cm^{-1}): 3483, 2961, 2876, 1757, 1470, 1373, 1312, 1272, 1213, 1173, 1128, 1062, 1011, 981, 952, 857, 832, 781, 745, 714. **^{1}H NMR** (300 MHz, $CDCl_3$, 299 K) δ (ppm) = 4.35 (qd, *J* = 7.2 Hz, *J* = 1.5 Hz, 2H), 4.09 (dddd, *J* = 14.7 Hz, *J* = 10.5 Hz, *J* = 7.9 Hz, *J* = 2.7 Hz, 1H), 2.14 (brs, 1H), 1.97 – 1.77 (m, 1H), 1.53 (ddd, *J* = 14.7 Hz, *J* = 10.4 Hz, *J* = 4.6 Hz, 1H), 1.45 – 1.30 (m, 4H), 0.98 (d, *J* = 6.7 Hz, 3H), 0.93 (d, *J* = 6.6 Hz, 3H). **^{13}C NMR** (75 MHz, $CDCl_3$, 299 K) δ (ppm) = 163.9 (dd, *J* = 32.7 Hz, *J* = 31.3 Hz, C), 114.9 (dd, *J* = 256.5 Hz, *J* = 254.4 Hz, C), 70.3 (dd, *J* = 27.1 Hz, *J* = 24.9 Hz, CH), 63.2 (CH_2), 38.1 (dd, *J* = *J* = 2.0 Hz, CH_2), 24.2 (CH), 23.6 (CH_3), 21.4 (CH_3), 14.1 (CH_3). **^{19}F{^{1}H} NMR** (282 MHz, $CDCl_3$, 299 K) δ (ppm) = –115.28 (d, *J* = 263.7 Hz, 1F),

−122.37 (d, J = 263.7 Hz, 1F). **HRMS** (ESI) m/z = 233.0960 calcd. for $C_9H_{16}F_2NaO_3^+$ [M+Na]$^+$, found: 233.0966.

Ethyl 2,2-difluoro-3-hydroxyoctanoate (1aa)

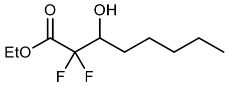

1aa was prepared following a modified procedure by *Sorensen and coworkers* (*15*). Under argon, anhydrous THF (70 mL) was heated to 70 °C and activated zinc dust (2.0 equiv, 15.0 mmol, 981 mg), hexanal (1.0 equiv, 7.5 mmol, 0.93 mL) and ethyl bromodifluoroacetate (2.0 equiv, 15.0 mmol, 1.92 mL) was added. After the strongly exothermic reaction occurred, the mixture was refluxed for 1 hr and was then allowed to cool down to room temperature overnight. The reaction was quenched by the addition of sat. NH_4Cl solution (10 mL). The aqueous layer was extracted with Et_2O (3 x 50 mL) and the combined organic layers washed with brine (10 mL) followed by drying over $MgSO_4$. Upon removal of the solvent *in vacuo*, the desired aldol was obtained by flash column chromatography (pentane/Et_2O 9:1) as a colorless liquid (1.02 g, 4.53 mmol, 60%). **FT IR** (neat) ν (cm^{-1}): 3448, 2958, 2934, 2864, 1758, 1717, 1399, 1375, 1314, 1234, 1212, 1115, 1061, 1013, 930,855, 784,725. **^{1}H NMR** (300 MHz, $CDCl_3$, 299 K) δ (ppm) = 4.35 (q, J = 7.1 Hz, 2H), 4.01 (dddd, J = 14.9 Hz, J = 9.5 Hz, J = 7.7 Hz, J = 2.8 Hz, 1H), 2.39 (s, 1H), 1.76 – 1.47 (m, 3H), 1.45 – 1.22 (m, 8H), 0.97 – 0.86 (m, 3H). **^{13}C NMR** (75 MHz, $CDCl_3$, 299 K) δ (ppm) = 163.9 (dd, J = 32.6 Hz, J = 31.4 Hz, C), 114.8 (dd, J = 256.6 Hz, J = 254.3 Hz, C), 71.95 (dd, J = 27.2, 25.0 Hz, CH), 63.2 (CH_2), 31.6 (CH_2), 29.3 (dd, J = J = 2.3 Hz, CH_2), 25.0 (CH_2), 22.6 (CH_2), 14.1 (2xCH_3). **^{19}F{^{1}H} NMR** (282 MHz, $CDCl_3$, 299 K) δ (ppm) = –114.96 (d, J = 264.1 Hz), –122.38 (d, J = 264.1 Hz). **HRMS** (ESI) m/z = 247.1116 calcd. for $C_{10}H_{18}F_2NaO_3^+$ [M+Na]$^+$, found: 247.1115.

3-Hydroxy-2,2-dimethyloctanoic acid (S-21)

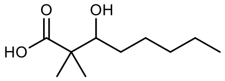

Methyl 3-hydroxy-2,2-dimethyloctanoate (**1w**, 1.0 equiv, 7.42 mmol, 1.50 g) was dissolved in a mixture of MeOH and water (4:1, 70 mL) and LiOH·H_2O (6.0 equiv, 44.5 mmol, 1.91 g) was added. Upon stirring at 75 °C for 12 hrs, the solvent was removed *in vacuo* and the residue was taken up in aqueous HCl (2 M, 10 mL). The aqueous layer was extracted with EtOAc (3 x 100 mL) and the combined organic layers were washed with brine (10 mL) and dried over $MgSO_4$. The acid was obtained upon removal of the solvent *in vacuo* as a colourless oil (1.13 g, 6.00 mmol, 81%). **FT IR** (neat) ν (cm^{-1}): 3401, 2958, 2933, 286, 1700, 1468, 1399, 1378, 1261, 1170, 1147, 1117, 1073, 1007, 926, 860, 728, 655. **^{1}H NMR** (300 MHz, $CDCl_3$, 299 K) δ (ppm) = 3.71 – 3.56 (m, 1H), 1.70 – 1.14 (m, 14H), 0.97 – 0.78 (m, 3H). **^{13}C NMR** (75 MHz, $CDCl_3$, 299 K) δ (ppm) = 183.2 (C), 76.8 (CH), 47.2 (C), 31.9 (CH_2), 31.7 (CH_2), 26.4 (CH_2), 22.8 (CH_2), 22.6 (CH_3), 20.3 (CH_3), 14.2 (CH_3). **HRMS** (ESI) m/z = 187.1342 calcd. for $C_{10}H_{19}O_3^-$ [M–H]$^-$, found: 187.1340.

Methyl (3-hydroxy-2,2-dimethyloctanoyl)glycinate (1ab)

Following a modified procedure by *Nicolaou and coworkers* (*16*), methyl (3-hydroxy-2,2-dimethyloctanoyl)glycinate (**S-21**, 1.0 equiv, 2.00 mmol, 376 mg), HOAt (4.0 equiv, 8.0 mmol, 1.09 g), EDC·HCl (5.0 equiv, 10.0 mmol, 1.92 g) and glycine methyl ester hydrochloride (4.0 equiv, 8.0 mmol, 1.00 mg) were dissolved in anhydrous DMF (20 mL) and $i$$Pr_2$EtN (10.0 equiv, 20.0 mmol, 3.40 mL) was added slowly. After the solution was stirred for 18 hrs, aqueous HCl (1 M, 50 mL) was added and the aqueous layer

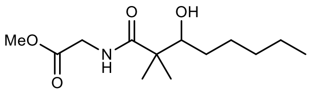

was extracted with Et_2O (3 x 100 mL). The combined organic layers were washed with brine (10 mL), dried over $MgSO_4$ and the solvent was removed *in vacuo*. Pure **1ab** was obtained by flash column chromatography (pentane/acetone 4:1) as a colourless oil (476 mg, 1.84 mmol, 92%). **FT IR** (neat) ν (cm^{-1}): 3347, 2954, 2933, 2859, 1745, 1645, 1527, 1460, 1437, 1367, 1207, 1178, 1120, 1068, 1024, 984, 941, 735. **^{1}H NMR** (400 MHz, $CDCl_3$, 299 K) δ (ppm) = 6.72 (brt, *J* = 5.6 Hz, 1H), 4.09 – 3.94 (m, 2H), 3.74 (s, 3H), 3.53 – 3.42 (m, 1H), 3.05 (brs, 1H), 1.66 – 1.42 (m, 2H), 1.37 – 1.19 (m, 10H), 1.15 (s, 3H), 0.93 – 0.81 (m, 2H). **^{13}C NMR** (101 MHz, $CDCl_3$, 299 K) δ (ppm) = 178.3 (C), 171.0 (C), 77.9 (CH), 52.5 (CH_3), 46.4 (C), 41.3 (CH_2), 31.9 (CH_2), 31.6 (CH_2), 26.5 (CH_2), 24.0 (CH_3), 22.8 (CH_2), 20.8 (CH_3), 14.2 (CH_3). **HRMS** (ESI) *m/z* = 282.1676 calcd. for $C_{13}H_{25}NNaO_4^+$ $[M+Na]^+$, found: 282.1681.

2-Cyclopentylethyl 2-iodobenzenesulfonate (S-22)

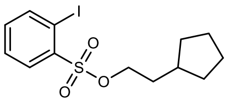

S-22 was prepared following a slightly modified procedure by *Tenabe and coworkers* (*3*). 2-Cyclopentylethanol (1.0 equiv, 3.00 mmol, 0.372 mL) was dissolved in anhydrous CH_2Cl_2 (3 mL) followed by addition of $Me_3N \cdot HCl$ (0.1 equiv, 0.30 mmol, 29 mg) and Et_3N (2.5 equiv, 7.50 mmol, 1.04 mL). To this mixture, 2-iodobenzene sulfonyl chloride **S-11** (1.2 equiv, 3.60 mmol, 1.09 g) in anhydrous CH_2Cl_2 (3 mL) was added via a septum at 0 °C. After 2 hrs, the reaction mixture was transferred onto a column and directly purified by flash chromatography (pentane/Et_2O 9:1) yielding **S-11** as a colorless oil (1.09 g, 2.87 mmol, 96%). **FT IR** (neat) ν (cm^{-1}): 2945, 2913, 2864, 1570, 1450, 1430, 1421, 1358, 1276, 1256, 1180, 1124, 1098, 1037, 1015, 953, 922, 893, 832, 756, 723, 703, 641, 615, 584. **^{1}H NMR** (300 MHz, $CDCl_3$, 299 K) δ (ppm) = 8.17 – 8.07 (m, 2H), 7.52 (ddd, *J* = *J* = 7.7 Hz, *J* = 1.2 Hz, 1H), 7.26 (ddd, *J* = *J* = 7.7 Hz, *J* = 1.7 Hz, 1H), 4.10 (t, *J* = 6.7 Hz, 2H), 2.01 – 1.82 (m, 1H), 1.79 – 1.66 (m, 4H), 1.64 – 1.43 (m, 4H), 1.14 – 0.93 (m, 2H). **^{13}C NMR** (75 MHz, $CDCl_3$, 299 K) δ (ppm) = 143.0 (CH), 139.6 (C), 134.3 (CH), 131.8 (CH), 128.4 (CH), 92.5 (CH), 71.1 (CH_2), 36.3 (CH), 35.0 (CH_2), 32.5 (CH_2), 25.1 (CH_2). **HRMS** (ESI) *m/z* = 402.9835 calcd. for $C_{13}H_{17}INaO_3S^+$ $[M+Na]^+$, found: 402.9839.

2,2,5-Trimethylhexan-3-yl 2-iodobenzenesulfonate (2a-1)

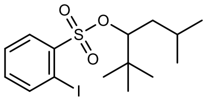

Sulfonate **2a-1** was prepared following **GP4** with **1a** (1.0 equiv, 1.09 mmol, 157 mg), **S-11** (1.2 equiv, 1.31 mmol, 396 mg), Me_3N (2.0 M in THF, 1.5 equiv, 1.63 mmol, 0.82 mL) and *n*BuLi (1.6 M in hexane, 1.2 equiv, 1.31 mmol, 0.82 mL) in anhydrous THF (4.1 mL each). After a reaction time of 2 hrs, the desired sulfonate was obtained by flash column chromatography (pentane/Et_2O 9:1) as a slightly yellow solid (449 mg, 1.07 mmol, 98%). **MP**.: 62 °C. **FT IR** (neat) ν (cm^{-1}): 3095, 3060, 2958, 2869, 1572, 1480, 1448, 1425, 1399, 1354, 1341, 1276, 1258, 1178, 1123, 110, 1056, 1018, 886, 761, 726, 705, 669, 642, 601, 570. **^{1}H NMR** (400 MHz, $CDCl_3$, 299 K) δ (ppm) = 8.09 (dd, *J* = 7.8 Hz, *J* =1.2 Hz, 1H), 8.05 (dd, *J* = 7.9 Hz, *J* =1.6 Hz, 1H), 7.47 (ddd, *J* = *J* = 7.6 Hz, *J* = 1.2 Hz, 1H), 7.21 (ddd, *J* = *J* = 7.6 Hz, *J* = 1.6 Hz, 1H), 4.63 (dd, *J* = 9.7 Hz, *J* = 1.8 Hz, 1H), 1.67 (ddd, *J* = 14.8 Hz, *J* = 9.7 Hz, *J* = 3.0 Hz, 1H), 1.41 (dqd, *J* = 15.9 Hz, *J* = 6.5 Hz, *J* = 3.1 Hz, 1H), 1.26 (ddd, *J* = 14.7 Hz, *J* = 10.7 Hz, *J* = 1.8 Hz, 1H), 0.95 – 0.70 (m, 15H). **^{13}C NMR** (101 MHz, $CDCl_3$, 299 K) δ (ppm) = 142.7 (CH), 142.4 (C), 133.6 (CH), 130.2 (CH), 128.1 (CH), 93.8 (CH), 92.3 (C), 39.9 (CH_2), 35.4 (C), 26.4 (CH_3), 24.6

(CH), 24.0 (CH_3), 21.2 (CH_3). **HRMS** (ESI) *m/z* = 433.0305 calcd. for $C_{15}H_{23}INaO_3S^+$ [M+Na]$^+$, found: 433.0294.

2,2,5-Trimethylhexan-3-yl 2-iodo-5-methylbenzenesulfonate (2a-2)

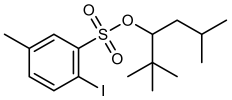

Sulfonate **2a-2** was prepared following **GP4** with **1a** (1.0 equiv, 1.0 mmol, 144 mg), **S-11** (1.2 equiv, 1.2 mmol, 380 mg), Me_3N (2.0 M in THF, 1.5 equiv, 1.5 mmol, 0.75 mL) and *n*BuLi (1.6 M in hexane, 1.2 equiv, 1.2 mmol, 0.75 mL) in anhydrous THF (3.75 mL each). After a reaction time of 2 hrs, the desired sulfonate was obtained by flash column chromatography (pentane/Et_2O 99:1) as a colorless solid (254 mg, 0.599 mmol, 60%). **MP**.: decomposition > 110 °C. **FT IR** (neat) ν (cm^{-1}): 2960, 2870, 1459, 1380, 1357, 1260, 1221, 1176, 1104, 1015, 895, 863, 824, 763, 694, 610, 581. **^{1}H NMR** (300 MHz, $CDCl_3$, 299 K) δ (ppm) = 7.93 (d, *J* = 8.0 Hz, 1H), 7.87 (d, *J* = 2.1 Hz, 1H), 7.05 – 6.98 (m, 1H), 4.61 (dd, *J* = 9.5 Hz, *J* = 1.9 Hz, 1H), 2.36 (s, 3H), 1.72 – 1.61 (m, 1H), 1.51 – 1.38 (m, 1H), 1.26 (ddd, *J* = 14.7 Hz, *J* = 10.6, *J* = 1.9 Hz, 1H), 0.91 (s, 9H), 0.83 (d, *J* = 6.4 Hz, 3H), 0.79 (d, *J* = 6.6 Hz, 3H). **^{13}C NMR** (75 MHz, $CDCl_3$, 299 K) δ (ppm) = 142.5 (CH), 142.1 (C), 138.7 (C), 134.5 (CH), 130.9 (CH), 93.6 (CH), 88.0 (C), 39.9 (CH_2), 35.5 (C), 26.4 (CH_3), 24.6 (CH), 23.9 (CH_3), 21.3 (CH_3), 21.0 (CH_3). **HRMS** (ESI) *m/z* = 447.0461 calcd. for $C_{16}H_{25}INaO_3S^+$ [M+Na]$^+$, found: 447.0454.

2,2,5-Trimethylhexan-3-yl 5-chloro-2-iodobenzenesulfonate (2a-3)

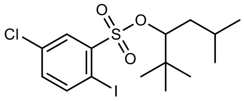

Sulfonate **2a-3** was prepared following **GP4** with **1a** (1.0 equiv, 1.09 mmol, 157 mg), **S-15** (1.2 equiv, 1.31 mmol, 441 mg), Me_3N (2.0 M in THF, 1.5 equiv, 1.63 mmol, 0.82 mL) and *n*BuLi (1.6 M in hexane, 1.2 equiv, 1.31 mmol, 0.82 mL) in anhydrous THF (4.1 mL each). After a reaction time of 2 hrs, the desired sulfonate was obtained by flash column chromatography (pentane/Et_2O 99:1) as a colorless solid (336 mg, 0.756 mmol, 69%). **MP**.: decomposition upon removal of solvent. **FT IR** (neat) ν (cm^{-1}): 2959, 2870, 1469, 1446, 1369, 1356, 1249, 1181, 1106, 1059, 1015, 893, 826, 760, 694, 674, 608, 583. **^{1}H NMR** (300 MHz, $CDCl_3$, 299 K) δ (ppm) = 8.07 – 7.93 (m, 2H), 7.19 (dd, *J* = 8.4 Hz, *J* = 2.5 Hz, 1H), 4.65 (dd, *J* = 9.7 Hz, *J* = 1.8 Hz, 1H), 1.68 (ddd, *J* = 14.6 Hz, *J* = 9.7 Hz, *J* = 3.0 Hz, 1H), 1.43 (dqd, *J* = 15.7 Hz, *J* = 6.5 Hz, *J* = 2.9 Hz, 1H), 1.34 – 1.22 (m, 1H), 0.92 (s, 9H), 0.85 (d, *J* = 6.4 Hz, 3H), 0.81 (d, *J* = 6.5 Hz, 3H). **^{13}C NMR** (75 MHz, $CDCl_3$, 299 K) δ (ppm) = 143.8 (C), 143.6 (CH), 134.8 (C), 133.5 (CH), 130.1 (CH), 94.5 (CH), 89.2 (C), 39.7 (CH_2), 35.3 (C), 26.2 (CH_3), 24.5 (CH), 23.8 (CH_3), 21.0 (CH_3). **HRMS** (ESI) *m/z* = 910.9938 calcd. for $C_{30}H_{44}Cl_2I_2NaO_6S_2^+$ [2M+Na]$^+$, found: 910.9941.

2,5,5-Trimethylheptan-4-yl 2-iodobenzenesulfonate (2b)

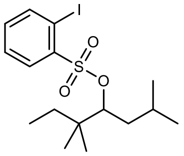

Sulfonate **2b** was prepared following **GP4** with **1b** (1.0 equiv, 1.00 mmol, 158 mg), **S-11** (1.2 equiv, 1.20 mmol, 363 mg), Me_3N (2.0 M in THF, 1.5 equiv, 1.50 mmol, 0.75 mL) and *n*BuLi (1.6 M in hexane, 1.2 equiv, 1.20 mmol, 0.75 mL) in anhydrous THF (3.8 mL each). After a reaction time of 2 hrs, the desired sulfonate was obtained by flash column chromatography (pentane/Et_2O 9:1) as a colorless oil (336 mg, 0.792 mmol, 79%). The neat compound is unstable and was stored in Et_2O. **FT IR** (neat) ν (cm^{-1}): 2960, 2870, 2362, 1571, 1466 ,1449 ,1431, 1358, 1276, 1257, 1179, 1124, 1099, 1069, 1038, 1015, 888, 758, 728, 703,642, 602. **^{1}H NMR** (300 MHz, $CDCl_3$, 299 K) δ (ppm) = 8.09 (dd, *J*=7.9, 1.2, 1H), 8.04 (dd, *J* = 7.9 Hz, *J* = 1.6 Hz, 1H), 7.47 (ddd, *J* = *J* = 7.7 Hz,

J = 1.2 Hz, 1H), 7.20 (ddd, *J* = *J* = 7.7 Hz, *J* = 1.7 Hz, 1H), 4.73 (dd, *J* = 9.8 Hz, *J* = 1.7 Hz, 1H), 1.68 (ddd, *J* = 14.8 Hz, *J* = 9.8 Hz, *J* = 2.9 Hz, 1H), 1.52 – 1.37 (m, 1H), 1.36 – 1.18 (m, 3H), 0.93 – 0.72 (m, 15H). **^{13}C NMR** (75 MHz, $CDCl_3$, 299 K) δ (ppm) = 142.7 (CH), 142.5 (C), 133.6 (CH), 130.2 (CH), 128.1 (CH), 93.1 (CH), 92.2 (C), 39.6 (CH_2), 38.0 (C), 31.4 (CH_2), 24.6 (CH), 24.0 (CH_3), 23.6 (CH_3), 22.9 (CH_3), 21.3 (CH_3), 8.2 (CH_3). **HRMS** (ESI) *m/z* = 447.0461 calcd. for $C_{16}H_{25}INaO_3S^+$ $[M+Na]^+$, found: 447.0448.

2,5-Dimethylhexan-3-yl 2-iodobenzenesulfonate (2c)

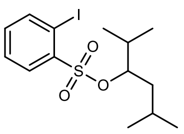

Sulfonate **2c** was prepared following **GP4** with 2,5-Dimethyl-3-hexanol (1.0 equiv, 2.0 mmol, 0.32 mL), **S-11** (1.2 equiv, 2.4 mmol, 726 mg), Me_3N (2.0 M in THF, 1.5 equiv, 3.0 mmol, 1.5 mL) and *n*BuLi (1.6 M in hexane, 1.2 equiv, 2.4 mmol, 1.5 mL) in anhydrous THF (7.5 mL each). After a reaction time of 2 hrs, the desired sulfonate was obtained by flash column chromatography (pentane/Et_2O 9:1) as a colorless oil (780 mg, 1.97 mmol, 98%). **FT IR** (neat) ν (cm^{-1}): 2961, 2935, 2872, 1571, 1467, 1448, 1362, 1257, 1182, 1124, 1099, 1037, 1016, 959, 892, 822, 760, 728, 704, 604, 582. **^{1}H NMR** (400 MHz, $CDCl_3$, 299 K) δ (ppm) = 8.10 (ddd, *J* = 7.9 Hz, *J* = *J* = 1.5 Hz, 2H), 7.49 (ddd, *J* = *J* = 7.7 Hz, *J* = 1.2 Hz, 1H), 7.23 (ddd, *J* = *J* = 7.7 Hz, *J* = 1.6 Hz, 1H), 4.69 – 4.64 (m, 1H), 1.98 – 1.89 (m, 1H), 1.67 – 1.49 (m, 2H), 1.36 – 1.22 (m, 1H), 0.90 (d, *J* = 6.8 Hz, 3H), 0.88 (d, *J* = 7.0 Hz, 3H), 0.83 (d, *J* = 6.5 Hz, 3H), 0.79 (d, *J* = 6.5 Hz, 3H). **^{13}C NMR** (101 MHz, $CDCl_3$, 299 K) δ (ppm) = 142.9 (CH), 141.5 (C), 133.9 (CH), 130.8 (CH), 128.3 (CH), 92.6 (C), 89.4 (CH), 39.5 (CH_2), 31.9 (CH), 24.4 (CH), 23.2 (CH_3), 21.9 (CH_3), 17.8 (CH_3), 17.4 (CH_3). **HRMS** (ESI) *m/z* = 419.0148 calcd. for $C_{14}H_{21}INaO_3S^+$ $[M+Na]^+$, found: 419.0135.

2,6-Dimethylheptan-4-yl 2-iodobenzenesulfonate (2d)

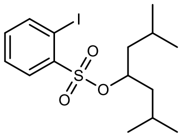

Sulfonate **2d** was prepared following **GP4** with **1d** (1.0 equiv, 1.0 mmol, 144 mg), **S-11** (1.2 equiv, 1.2 mmol, 363 mg), Me_3N (2.0 M in THF, 1.5 equiv, 1.5 mmol, 0.75 mL) and *n*BuLi (1.6 M in hexane, 1.2 equiv, 1.2 mmol, 0.75 mL) in anhydrous THF (3.75 mL each). After a reaction time of 2 hrs, the desired sulfonate was obtained by filtration over a plug of silica followed by flash column chromatography (pentane/Et_2O 99:1) as a colorless oil (248 mg, 0.604 mmol, 60%). The neat compound is unstable and was stored in Et_2O. **FT IR** (neat) ν (cm^{-1}): 2958, 2930, 2870, 1571, 1468, 1450, 1430, 1361, 1255, 1183, 1123, 1099, 1038, 1016, 893, 759, 725, 704, 642, 591. **^{1}H NMR** (300 MHz, $CDCl_3$, 299 K) δ (ppm) = 8.15 – 8.10 (m, 2H), 7.50 (ddd, *J* = *J* = 7.7 Hz, *J* = 1.2 Hz, 1H), 7.27 – 7.20 (m, 1H), 4.81 – 4.70 (m, 1H), 1.68 – 1.54 (m, 4H), 1.49 – 1.34 (m, 2H), 0.87 – 0.75 (m, 12H). **^{13}C NMR** (75 MHz, $CDCl_3$, 299 K) δ (ppm) = 143.0 (CH), 141.3 (C), 133.9 (CH), 131.1 (CH), 128.3 (CH), 92.8 (C), 83.9 (CH), 44.1 (CH_2), 24.6 (CH), 22.8 (CH_3), 22.4 (CH_3). **HRMS** (ESI) *m/z* = 433.0305 calcd. for $C_{15}H_{23}INaO_3S^+$ $[M+Na]^+$, found: 433.0295.

2,6-Dimethyl-2-phenylheptan-4-yl 2-iodobenzenesulfonate (2e)

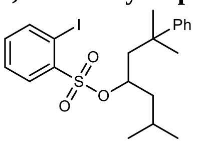

Sulfonate **2e** was prepared following **GP3** using **1e** (1.0 equiv, 1.0 mmol, 220 mg), **S-11** (1.5 equiv, 1.5 mmol, 454 mg), $Me_3N\cdot HCl$ (1.0 equiv, 1.0 mmol, 96 mg), Et_3N (2.5 equiv, 2.5 mmol, 0.35 mL) in anhydrous CH_2Cl_2 (0.55 mL each). After 1 hr, imidazole (100 mg) was added and the product was isolated by flash chromatography (pentane/Et_2O 9:1) as a colorless oil (431 mg, 0.886 mmol, 89%). **FT IR** (neat) ν (cm^{-1}): 3059, 2957, 2871,

16021570, 1496, 1466, 1445, 1364, 1254, 1180, 1124, 1098, 1053, 1031, 1016, 1000, 884, 758, 726, 699, 642, 594, 577. **^{1}H NMR** (400 MHz, $CDCl_3$, 299 K) δ (ppm) = 8.09 (dd, *J* = 7.9 Hz, *J* = 1.2 Hz, 1H), 8.02 (dd, *J* = 8.0 Hz, *J* = 1.6 Hz, 1H), 7.48 (ddd, *J* = *J* = 7.7 Hz, *J* = 1.2 Hz, 1H), 7.30 – 7.20 (m, 5H), 7.19 – 7.13 (m, 1H), 4.60 (tt, *J* = 6.4 Hz, *J* = 5.1 Hz, 1H), 2.08 (dd, *J* = 14.7 Hz, *J* = 4.9 Hz, 1H), 1.98 (dd, *J* = 14.7 Hz, 6.3, 1H), 1.48 – 1.36 (m, 1H), 1.34 – 1.21 (m, 7H), 0.95 (ddd, *J* = 14.4 Hz, *J* = 8.0 Hz, *J* = 5.3 Hz, 1H), 0.57 – 0.54 (m, 6H). **^{13}C NMR** (101 MHz, $CDCl_3$, 299 K) δ (ppm) = 147.8 (C), 142.9 (CH), 141.5 (C), 133.9 (CH), 131.0 (CH), 128.3 (CH), 128.4 (CH), 126.1 (CH), 126.0 (CH), 92.7 (C), 83.4 (CH), 49.2 (CH_2), 44.9 (CH_2), 36.9 (C), 31.0 (CH_3), 27.4 (CH_3), 24.3 (CH), 22.6 (CH_3), 22.0 (CH_3). **HRMS** (ESI) *m/z* = 509.0618 calcd. for $C_{21}H_{27}INaO_3S^+$ $[M+Na]^+$, found: 509.0630.

2,2,6-Trimethylheptan-4-yl 2-iodobenzenesulfonate (2f)

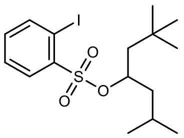

Sulfonate **2f** was prepared following **GP4** with **1f** (1.0 equiv, 1.0 mmol, 158 mg), **S-11** (1.2 equiv, 1.2 mmol, 363 mg), Me_3N (2.0 M in THF, 1.5 equiv, 1.5 mmol, 0.75 mL) and *n*BuLi (1.6 M in hexane, 1.2 equiv, 1.2 mmol, 0.75 mL) in anhydrous THF (3.75 mL each). After a reaction time of 2 hrs, the desired sulfonate was obtained by flash column chromatography (pentane/Et_2O 9:1) as a colorless oil (215 mg, 0.506 mmol, 51%). **FT IR** (neat) ν (cm^{-1}): 2958, 2870, 1571, 1469, 1450, 1364, 1254, 1179, 1124, 1099, 1039, 1016, 884, 758, 723, 703, 642, 616, 590. **^{1}H NMR** (300 MHz, $CDCl_3$, 299 K) δ (ppm) = 8.13 – 8.08 (m, 2H), 7.49 (ddd, *J* = *J* = 7.7 Hz, *J* = 1.2 Hz, 1H), 7.22 (ddd, *J* = *J* = 7.7 Hz, *J* = 1.7 Hz, 1H), 4.91 – 4.81 (m, 1H), 1.74 – 1.36 (m, 5H), 0.91 – 0.77 (m, 15H). **^{13}C NMR** (75 MHz, $CDCl_3$, 299 K) δ (ppm) = 142.9 (CH), 141.9 (C), 133.9 (CH), 130.9 (CH), 128.3 (CH), 92.7 (C), 83.4 (CH), 48.3 (CH_2), 45.8 (CH_2), 30.1 (C), 30.0 (CH_3), 24.7 (CH), 22.8 (CH_3), 22.4 (CH_3). **HRMS** (ESI) *m/z* = 447.0461 calcd. for $C_{16}H_{25}INaO_3S^+$ $[M+Na]^+$, found: 447.0461.

1-Cyclohexyl-3,3-dimethylbutan-2-yl 2-iodobenzenesulfonate (2g)

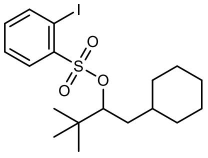

Sulfonate **2g** was prepared following **GP4** with **1g** (1.0 equiv, 0.50 mmol, 91 mg), **S-11** (1.2 equiv, 0.60 mmol, 182 mg), Me_3N (2.0 M in THF, 1.5 equiv, 0.75 mmol, 0.38 mL) and *n*BuLi (1.6 M in hexane, 1.2 equiv, 0.60 mmol, 0.38 mL) in anhydrous THF (1.9 mL each). After a reaction time of 2 hrs, the desired sulfonate was obtained by flash column chromatography (pentane/Et_2O 9:1) as a colorless solid (208 mg, 0.460 mmol, 92%). The neat compound is unstable and was stored in Et_2O. **FT IR** (neat) ν (cm^{-1}): 2962, 2922, 2850, 1571, 1480, 1449, 1430, 1399, 1361, 1276, 1256, 1180, 1125, 1099, 1064, 1037, 1016, 944, 894, 758, 728, 704, 669, 642, 604, 583, 565. **^{1}H NMR** (300 MHz, $CDCl_3$, 299 K) δ (ppm) = 8.13 – 8.03 (m, 2H), 7.49 (ddd, *J* = *J* = 7.7 Hz, *J* = 1.2 Hz, 1H), 7.22 (ddd, *J* = *J* = 7.7 Hz, *J* = 1.7 Hz, 1H), 4.64 (dd, *J* = 9.7 Hz, *J* = 1.8 Hz, 1H), 1.88 – 1.46 (m, 5H), 1.43 – 1.21 (m, 2H), 1.12 – 0.55 (m, 15H). **^{13}C NMR** (75 MHz, $CDCl_3$, 299 K) δ (ppm) = 142.8 (CH), 142.7 (C), 133.6 (CH), 130.3 (CH), 128.2 (CH), 93.4 (CH), 92.4 (C), 38.3 (CH_2), 35.5 (C), 34.5 (CH_2), 34.0 (CH), 32.0 (CH_2), 26.5 (CH_2), 26.4 (CH_2), 26.3 (CH_3), 25.9 (CH_2). **HRMS** (ESI) *m/z* = 473.0618 calcd. for $C_{18}H_{27}INaO_3S^+$ $[M+Na]^+$, found: 473.0611.

1-Cyclopentyl-3,3-dimethylbutan-2-yl 2-iodobenzenesulfonate (2h)

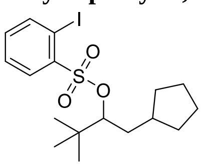

Sulfonate **2h** was prepared following **GP4** with **1h** (1.0 equiv, 0.50 mmol, 85 mg), **S-11** (1.2 equiv, 0.60 mmol, 182 mg), Me_3N (2.0 M in THF, 1.5 equiv, 0.75 mmol, 0.38 mL) and *n*BuLi (1.57 M in hexane, 1.2 equiv, 0.60 mmol, 0.38 mL) in anhydrous THF (1.9 mL each). After a reaction time of 2 hrs, imidazole (50 mg) was added and the desired sulfonate was obtained by flash column chromatography (pentane/Et_2O 20:1) as a colorless solid (199 mg, 0.456 mmol, 91%). **MP**.: decomposition > 90 °C. **FT IR** (neat) ν (cm^{-1}): 2957, 2921, 2870, 1571, 1480, 1450, 1428, 1398, 1362, 1256, 1181, 1125, 1100, 1016, 895, 758, 729, 642, 603. **^{1}H NMR** (300 MHz, $CDCl_3$, 299 K) δ (ppm) = 8.13 – 8.00 (m, 2H), 7.47 (ddd, *J* = *J* = 7.7 Hz, *J* = 1.2 Hz, 1H), 7.20 (ddd, *J* = *J* = 7.7 Hz, *J* = 1.7 Hz, 1H), 4.59 (dd, *J* = 9.8 Hz, *J* = 1.8 Hz, 1H), 1.92 – 1.71 (m, 2H), 1.70 – 1.23 (m, 8H), 1.01 – 0.87 (m, 10H). **^{13}C NMR** (75 MHz, $CDCl_3$, 299 K) δ (ppm) = 142.7 (CH), 142.5 (C), 133.5 (CH), 130.2 (CH), 128.1 (CH), 95.2 (CH), 92.4 (C), 36.9 (CH_2), 36.6 (CH), 35.5 (C), 33.5 (CH_2), 31.9 (CH_2), 26.4 (CH_3), 25.1 (CH_2), 25.0 (CH_2). **HRMS** (ESI) *m/z* = 459.0461 calcd. for $C_{17}H_{25}INaO_3S^+$ $[M+Na]^+$, found: 459.0478.

1-Cyclobutyl-3,3-dimethylbutan-2-yl 2-iodobenzenesulfonate (2i-1)

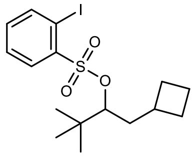

Sulfonate **2i-1** was prepared following **GP4** with **1i** (1.0 equiv, 1.0 mmol, 156 mg), **S-11** (1.2 equiv, 1.2 mmol, 363 mg), Me_3N (2.0 M in THF, 1.5 equiv, 1.5 mmol, 0.75 mL) and *n*BuLi (1.57 M in hexane, 1.2 equiv, 1.2 mmol, 0.75 mL) in anhydrous THF (3.75 mL each). After a reaction time of 2 hrs, imidazole (50 mg) was added and the desired sulfonate was obtained by flash column chromatography (pentane/Et_2O 9:1) as a colorless solid (416 mg, 0.985 mmol, 99%). The neat compound is unstable and was stored in Et_2O. **FT IR** (neat) ν (cm^{-1}): 2968, 2869, 1571, 1448, 1430, 1399, 1361, 1256, 1180, 1124, 1099, 1068, 1016, 887, 758, 729, 703, 642, 598, 568. **^{1}H NMR** (300 MHz, $CDCl_3$, 299 K) δ (ppm) = 8.10 (dd, *J* = 7.8 Hz, *J* = 1.2 Hz, 1H), 8.05 (dd, *J* = 7.9 Hz, *J* = 1.6 Hz, 1H), 7.48 (ddd, *J* = *J* = 7.7 Hz, *J* = 1.2 Hz, 1H), 7.22 (ddd, *J* = *J* = 7.7 Hz, *J* = 1.7 Hz, 1H), 4.45 (dd, *J* = 9.7 Hz, *J* = 2.2 Hz, 1H), 2.27 – 2.12 (m, 1H), 2.12 – 1.87 (m, 2H), 1.87 – 1.40 (m, 6H), 0.88 (s, 9H). **^{13}C NMR** (75 MHz, $CDCl_3$, 299 K) δ (ppm) = 142.7 (CH), 142.3 (C), 133.7 (CH), 130.2 (CH), 128.1 (CH), 93.5 (CH), 92.4 (C), 37.8 (CH_2), 35.2 (C), 32.4 (CH), 28.3 (CH_2), 28.0 (CH_2), 26.4 (CH_3), 18.2 (CH_2). **HRMS** (ESI) *m/z* = 445.0305 calcd. for $C_{16}H_{23}INaO_3S^+$ $[M+Na]^+$, found: 445.0327.

1-Cyclobutyl-3,3-dimethylbutan-2-yl 5-chloro-2-iodo-4-methylbenzenesulfonate (2i-2)

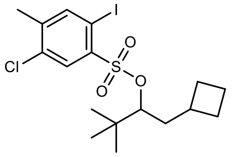

Sulfonate **2i-2** was prepared following **GP4** with **1i** (1.0 equiv, 0.50 mmol, 78 mg), **S-17** (1.2 equiv, 0.60 mmol, 211 mg), Me_3N (2.0 M in THF, 1.5 equiv, 0.75 mmol, 0.38 mL) and *n*BuLi (1.6 M in hexane, 1.2 equiv, 0.60 mmol, 0.38 mL) in anhydrous THF (1.9 mL each). After a reaction time of 2 hrs, imidazole (50 mg) was added and the desired sulfonate was obtained by flash column chromatography (pentane/Et_2O 20:1) as a colorless liquid (183 mg, 0.389 mmol, 78%). The neat compound is unstable and was stored in Et_2O. **FT IR** (neat) ν (cm^{-1}): 2965, 2871, 1574, 1538, 1450, 1364, 1249, 1210, 1179, 1115, 1065, 1005, 906, 878, 765, 715, 641, 613, 601, 554. **^{1}H NMR** (300 MHz, $CDCl_3$, 299 K) δ (ppm) = 8.05 (s, 1H), 7.89 (s, 1H), 4.45 (dd, *J* = 9.5 Hz, *J* = 2.3 Hz, 1H), 2.39 (s, 3H), 2.33 – 2.16 (m, 1H), 2.15 – 1.92 (m, 2H), 1.90 – 1.70 (m, 3H), 1.68 – 1.40 (m, 4H), 0.89 (s, 9H). **^{13}C NMR** (75 MHz, $CDCl_3$, 299 K) δ (ppm) = 142.0 (CH), 140.5 (C), 139.3 (C), 136.6 (C), 131.8 (CH), 93.6 (CH), 88.4 (C), 37.7 (CH_2), 35.1 (C), 32.4 (CH), 28.2 (CH_2), 27.9 (CH_2), 26.3 (CH_3), 19.7 (CH_3), 18.1 (CH_2). **HRMS** (ESI) *m/z* = 493.0072 calcd. for $C_{17}H_{24}ClINaO_3S^+$ $[M+Na]^+$, found: 493.0061.

5-Ethyl-2,2-dimethylheptan-3-yl 2-iodobenzenesulfonate (2j)

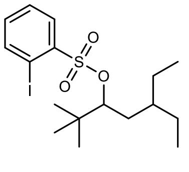

Sulfonate **2j** was prepared following **GP4** with **1j** (1.0 equiv, 0.7 mmol, 121 mg), **S-11** (1.2 equiv, 0.84 mmol, 254 mg), Me_3N (2.0 M in THF, 1.5 equiv, 1.05 mmol, 0.53 mL) and *n*BuLi (1.57 M in hexane, 1.2 equiv, 0.6 mmol, 0.54 mL) in anhydrous THF (2.7 mL each). After a reaction time of 2 hrs, imidazole (80 mg) was added and the desired sulfonate was obtained by flash column chromatography (pentane/Et_2O 9:1) as a colorless oil (312 mg, 0.689 mmol, 98%). The neat compound is unstable and was stored in Et_2O. **FT IR** (neat) ν (cm^{-1}): 2963, 2934, 2874, 1571, 1462, 1399, 1361, 1257, 1181, 1124, 1098, 1067, 1016, 940, 897, 759, 729, 642, 604. **^{1}H NMR** (400 MHz, $CDCl_3$, 299 K) δ (ppm) = 8.09 (dd, *J* = 7.8 Hz, *J* = 1.2 Hz, 1H), 8.04 (dd, *J* = 7.9 Hz, *J* = 1.6 Hz, 1H), 7.46 (ddd, *J* = *J* = 7.7 Hz, *J* = 1.2 Hz, 1H), 7.20 (ddd, *J* = *J* = 7.7 Hz, *J* = 1.6 Hz, 1H), 4.66 (dd, *J* = 9.9 Hz, *J* = 1.5 Hz, 1H), 1.62 (ddd, *J* = 15.1 Hz, *J* = 10.0 Hz, *J* = 2.8 Hz, 1H), 1.37 – 1.27 (m, 2H), 1.24 – 1.05 (m, 2H), 1.04 – 0.90 (m, 10H), 0.89 – 0.77 (m, 1H), 0.71 (t, *J* = 7.4 Hz, 3H), 0.61 (t, *J* = 7.4 Hz, 3H). **^{13}C NMR** (101 MHz, $CDCl_3$, 299 K) δ (ppm) = 142.7 (CH), 142.6 (C), 133.6 (CH), 130.1 (CH), 128.1 (CH), 94.1 (CH), 92.4 (C), 36.4 (CH), 35.6 (C), 34.2 (CH_2), 26.4 (CH_3), 25.9 (CH_2), 24.2 (CH_2), 11.0 (CH_3), 9.8 (CH_3). **HRMS** (ESI) *m/z* = 461.0618 calcd. for $C_{17}H_{27}INaO_3S^+$ $[M+Na]^+$, found: 461.0613.

***trans*-2-Isopropylcyclohexyl 2-iodobenzenesulfonate (2k)**

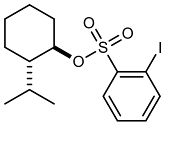

Sulfonate **2k** was prepared following **GP3** with **1k** (1.0 equiv, 0.50 mmol, 71 mg), $Me_3N·HCl$ (1.0 equiv, 0.50 mmol, 48 mg), Et_3N (2.5 equiv, 1.25 mmol, 0.17 mL) and **S-11** (1.5 equiv, 0.75 mmol, 227 mg) in anhydrous CH_2Cl_2 (0.50 mL each). After 1 hr, imidazole (50 mg) was added and the product was isolated by flash chromatography (pentane/Et_2O 9:1) as a colorless oil (201 mg, 0.492 mmol, 98%). **FT IR** (neat) ν (cm^{-1}): 2955, 2936, 2867, 1711, 1570, 1450, 1450, 1424, 1362, 1276, 1255, 1180, 1124, 1099, 1035, 1016, 948, 905, 894, 878, 839, 801, 758, 728, 703, 641, 616, 592, 569. **^{1}H NMR** (300 MHz, $CDCl_3$, 299 K) δ (ppm) = 8.16 – 8.07 (m, 2H), 7.50 (ddd, *J* = *J* = 7.7 Hz, *J* = 1.2 Hz, 1H), 7.27 – 7.17 (m, 1H), 4.51 (ddd, *J* = *J* = 9.9 Hz, *J* = 4.4 Hz, 1H),

2.11 – 1.89 (m, 2H), 1.80 – 1.44 (m, 5H), 1.31 – 0.94 (m, 3H), 0.86 (d, *J* = 7.0 Hz, 3H), 0.63 (d, *J* = 6.9 Hz, 3H). **^{13}C NMR** (101 MHz, $CDCl_3$, 299 K) δ (ppm) = 142.8 (CH), 141.3 (C), 133.8 (CH), 130.9 (CH), 128.2 (CH), 92.5 (C), 85.7 (CH), 47.7 (CH), 32.5 (CH_2), 25.7 (CH), 24.4 (CH_2), 24.3 (CH_2), 23.6 (CH_2), 20.7 (CH_3), 16.0 (CH_3). **HRMS** (ESI) *m/z* = 431.0148 calcd. for $C_{15}H_{21}INaO_3S^+$ [M+Na]$^+$, found: 431.0166.

(1*R*,2*S*,5*R*)-2-Isopropyl-5-methylcyclohexyl 2-iodobenzenesulfonate (2k-1)

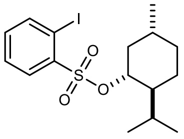

2k-1 was prepared following **GP3** with *L*-menthol (1.0 equiv, 1.10 mmol, 172 mg), $Me_3N \cdot HCl$ (1.0 equiv, 1.10 mmol, 105 mg), Et_3N (2.5 equiv, 2.75 mmol, 0.38 mL) and **S-11** (1.5 equiv, 1.65 mmol, 500 mg) in anhydrous CH_2Cl_2 (1.1 mL each). After 1 hr, the product was isolated by flash chromatography (pentane/CH_2Cl_2 2:1) as a colorless solid (463 mg, 1.10 mmol, > 99%). **MP**.: decomposition > 98 °C. **FT IR** (neat) ν (cm^{-1}): 2954, 2927, 2870, 1570, 1452, 1422, 1357, 1275, 1256, 1180, 1125, 1099, 1016, 938, 900, 869, 822, 805, 758, 728, 703, 641641, 617, 590. **^{1}H NMR** (300 MHz, $CDCl_3$, 299 K) δ (ppm) = 8.17 – 8.07 (m, 2H), 7.50 (ddd, *J* = *J* = 7.8 Hz, *J* = 1.2 Hz, 1H), 7.26 – 7.19 (m, 1H), 4.49 (td, *J* = 10.7 Hz, *J* = 4.6 Hz, 1H), 2.12 – 1.96 (m, 2H), 1.74 – 1.58 (m, 2H), 1.54 – 1.20 (m, 3H), 1.10 – 0.75 (m, 8H), 0.56 (d, *J* = 6.9 Hz, 3H). **^{13}C NMR** (75 MHz, $CDCl_3$, 299 K) δ (ppm) = 143.0 (CH), 141.4 (C), 133.9 (CH), 131.0 (CH), 128.4 (CH), 92.6 (C), 85.7 (CH), 47.7 (CH), 41.8 (CH_2), 33.8 (CH_2), 31.8 (CH), 25.7 (CH), 23.1 (CH_2), 22.0 (CH_3), 20.9 (CH_3), 15.4 (CH_3). **HRMS** (ESI) *m/z* = 445.0305 calcd. for $C_{16}H_{23}INaO_3S^+$ [M+Na]$^+$, found: 445.0313. **OR**.: $[\alpha]_D^{20}$ = −51.49 (c = 1.2 in $CHCl_3$).

(1*R*,2*S*,5*R*)-2-Isopropyl-5-methylcyclohexyl 2-iodo-5-methylbenzenesulfonate (2l-2)

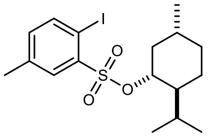

2l-2 was prepared following **GP3** with *L*-menthol (1.0 equiv, 1.10 mmol, 172 mg), $Me_3N \cdot HCl$ (1.0 equiv, 1.10 mmol, 105 mg), Et_3N (2.5 equiv, 2.75 mmol, 0.38 mL) and **S-13** (1.5 equiv, 1.65 mmol, 522 mg) in anhydrous CH_2Cl_2 (1.1 mL each). After 1 hr, imidazole (100 mg) was added and the product was isolated by flash chromatography (pentane/Et_2O 9:1) as a colorless solid (451 mg, 1.03 mmol, 94%). **MP**.: 61 °C. **FT IR** (neat) ν (cm^{-1}): 2956, 2927, 2871, 1457, 1382, 1356, 1260, 1220, 1177, 1104, 1014, 939, 905, 887, 860, 818, 802, 693, 626, 603, 585. **^{1}H NMR** (300 MHz, $CDCl_3$, 299 K) δ (ppm) = 7.98 – 7.91 (m, 2H), 7.04 (ddd, *J* = 8.0 Hz, *J* = 2.2 Hz, *J* = 0.8 Hz, 1H), 4.48 (td, *J* = 10.7 Hz, *J* = 4.6 Hz, 1H), 2.38 (s, 3H), 2.13 – 1.98 (m, 2H), 1.75 – 1.53 (m, 2H), 1.52 – 1.18 (m, 3H), 1.09 – 0.74 (m, 8H), 0.56 (d, *J* = 6.9 Hz, 3H). **^{13}C NMR** (75 MHz, $CDCl_3$, 299 K) δ (ppm) = 142.7 (CH), 141.0 (C), 138.9 (C), 134.9 (CH), 131.7 (CH), 88.4 (C), 85.5 (CH), 47.8 (CH), 41.9 (CH_2), 33.8 (CH_2), 31.9 (CH), 25.7 (CH), 23.2 (CH_2), 22.0 (CH_3), 21.0 (CH_3), 21.0 (CH_3), 15.4 (CH_3). **HRMS** (ESI) *m/z* = 459.0461 calcd. for $C_{17}H_{25}INaO_3S^+$ [M+Na]$^+$, found: 459.0464. **OR**.: −49.09 (c = 1.0 in $CHCl_3$).

(1*R*,2*S*,5*R*)-2-Isopropyl-5-methylcyclohexyl 5-chloro-2-iodobenzenesulfonate (2l-3)

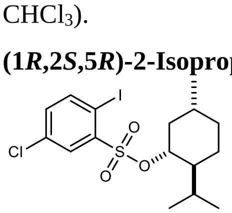

2l-3 was prepared following **GP3** with *L*-menthol (1.0 equiv, 1.10 mmol, 172 mg), $Me_3N \cdot HCl$ (1.0 equiv, 1.10 mmol, 105 mg), Et_3N (2.5 equiv, 2.75 mmol, 0.38 mL) and **S-15** (1.5 equiv, 1.65 mmol, 556 mg) in anhydrous CH_2Cl_2 (1.3 mL each). After 1 hr, imidazole (100 mg) was added and the product was isolated by flash chromatography (pentane/Et_2O 9:1) as a yellow solid (500 mg, 1.10 mmol, > 99%). **MP**.: 65 °C. **FT IR** (neat) ν (cm^{-1}): 2928, 2855, 2363, 2335, 1623, 1597, 1570, 1487, 1461, 1428, 1337, 1255, 1114, 1032, 968, 838,

777, 753, 704, 637. **^{1}H NMR** (300 MHz, $CDCl_3$, 299 K) δ (ppm) = 8.10 (d, J = 2.5 Hz, 1H), 8.01 (dd, J = 8.4 Hz, 1.3, 1H), 7.22 (dd, J = 8.4 Hz, 2.5, 1H), 4.54 (td, J = 10.8 Hz, J = 10.7 Hz, J = 4.6 Hz, 1H), 2.14 – 1.94 (m, 2H), 1.74 – 1.60 (m, 2H), 1.53 – 1.20 (m, 3H), 1.10 – 0.74 (m, 8H), 0.60 (d, J = 6.9 Hz, 3H). **^{13}C NMR** (75 MHz, $CDCl_3$, 299 K) δ (ppm) = 144.0 (CH), 143.0 (C), 135.1 (C), 134.0 (CH), 131.0 (CH), 89.8 (C), 86.5 (CH), 47.8 (CH), 41.9 (CH_2), 33.8 (CH_2), 31.9 (CH), 25.8 (CH), 23.2 (CH_2), 22.0 (CH_3), 21.0 (CH_3), 15.5 (CH_3). **HRMS** (ESI) *m/z* = 478.9915 calcd. for $C_{16}H_{22}ClINaO_3S^+$ $[M+Na]^+$, found: 478.9907. **OR**.: $[\alpha]_D^{20}$ = −41.87 (c = 1.6 in $CHCl_3$).

(1*R*,2*S*,5*R*)-2-Isopropyl-5-methylcyclohexyl 2-iodo-5-methoxybenzenesulfonate (2l-4)

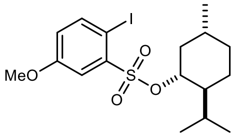

2l-4 was prepared following **GP3** with *L*-menthol (1.0 equiv, 1.10 mmol, 172 mg), $Me_3N·HCl$ (1.0 equiv, 1.10 mmol, 105 mg), Et_3N (2.5 equiv, 2.75 mmol, 0.38 mL) and **S-14** (1.5 equiv, 1.65 mmol, 499 mg) in anhydrous CH_2Cl_2 (1.3 mL each). After 1 hr, imidazole (100 mg) was added and the product was isolated by flash chromatography (pentane/Et_2O 9:1) as a colorless solid (453 mg, 1.07 mmol, > 99%). **MP**.: decomposition > 82 °C. **FT IR** (neat) ν (cm^{-1}): 2956, 2934, 2871, 1587, 1562, 1462, 1438, 1385, 1357, 1292, 1262, 1233, 1178, 1094, 1038, 1009, 939, 906, 862, 819, 803, 694, 626, 598. **^{1}H NMR** (300 MHz, $CDCl_3$, 299 K) δ (ppm) = 7.94 (d, J = 8.6 Hz, 1H), 7.67 (d, J = 3.0 Hz, 1H), 6.80 (dd, J = 8.7 Hz, J = 3.1 Hz, 1H), 4.50 (td, J = 10.7 Hz, J = 4.6 Hz, 1H), 3.85 (s, 3H), 2.19 – 2.00 (m, 2H), 1.80 – 1.16 (m, 5H), 1.11 – 0.68 (m, 8H), 0.59 (d, J = 6.9 Hz, 3H). **^{13}C NMR** (75 MHz, $CDCl_3$, 299 K) δ (ppm) = 159.8 (C), 143.6 (CH), 142.2 (C), 120.4 (CH), 116.8 (CH), 85.8 (CH), 80.6 (C), 56.0 (CH_3), 47.8 (CH), 41.8 (CH_2), 33.8 (CH_2), 31.9 (CH), 25.7 (CH), 23.2 (CH_2), 22.0 (CH_3), 21.0 (CH_3), 15.5 (CH_3). **HRMS** (ESI) *m/z* = 475.0410 calcd. for $C_{17}H_{25}INaO_4S^+$ $[M+Na]^+$, found: 475.0395. **OR**.: $[\alpha]_D^{20}$ = −43.21 (c = 1.2 in $CHCl_3$).

(1*R*,2*S*,5*R*)-2-Isopropyl-5-methylcyclohexyl 2-iodo-3,5-dimethylbenzenesulfonate (2l-5)

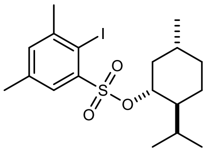

2l-5 was prepared following **GP3** with *L*-menthol (1.0 equiv, 1.10 mmol, 172 mg), $Me_3N·HCl$ (1.0 equiv, 1.10 mmol, 105 mg), Et_3N (2.5 equiv, 2.75 mmol, 0.38 mL) and **S-16** (1.5 equiv, 1.65 mmol, 545 mg) in anhydrous CH_2Cl_2 (1.1 mL each). After 1 hr, imidazole (100 mg) was added and the product was isolated by flash chromatography (pentane/Et_2O 9:1) as a colorless solid (495 mg, 1.10 mmol, > 99%). **MP**.: 94 °C. **FT IR** (neat) ν (cm^{-1}): 2957, 2926, 2870, 1454, 1412, 1357, 1224, 1180, 1148, 1012, 939, 905, 875, 800, 698, 643, 613, 586. **^{1}H NMR** (300 MHz, $CDCl_3$, 299 K) δ (ppm) = 7.75 (d, J = 2.2 Hz, 1H), 7.25 – 7.20 (m, 1H), 4.47 (td, J = 10.7 Hz, J = 10.6 Hz, 4.6, 1H), 2.50 (d, J = 2.4 Hz, 3H), 2.30 (d, J = 2.4 Hz, 3H), 2.12 – 1.96 (m, 2H), 1.70 – 1.53 (m, 3H), 1.49 – 1.14 (m, 3H), 1.06 – 0.70 (m, 8H), 0.60 – 0.50 (m, 3H). **^{13}C NMR** (75 MHz, $CDCl_3$, 299 K) δ (ppm) = 145.4 (C), 141.9 (C), 138.4 (C), 134.6 (CH), 129.5 (CH), 95.5 (C), 85.4 (CH), 47.8 (CH), 41.9 (CH_2), 33.9 (CH_2), 31.9 (CH), 30.4 (CH_3), 25.7 (CH), 23.2 (CH_2), 22.0 (CH_3), 21.0 (CH_3), 20.9 (CH_3), 15.5 (CH_3). **HRMS** (ESI) *m/z* = 473.0618 calcd. for $C_{18}H_{27}INaO_3S^+$ $[M+Na]^+$, found: 473.0605. **OR**.: $[\alpha]_D^{20}$ = −47.63 (c = 0.99 in $CHCl_3$).

(1*R*,2*S*,5*R*)-2-Isopropyl-5-methylcyclohexyl 2-iodonaphthalene-1-sulfonate (2l-6)

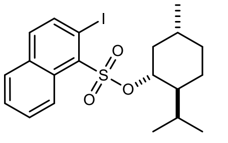

2l-6 was prepared following **GP3** with *L*-menthol (1.0 equiv, 0.96 mmol, 150 mg), $Me_3N{\cdot}HCl$ (1.0 equiv, 0.92 mmol, 96 mg), Et_3N (2.5 equiv, 2.40 mmol, 0.33 mL) and **S-12** (1.5 equiv, 1.65 mmol, 508 mg) in anhydrous CH_2Cl_2 (1.0 mL each). After 1 hr, the product was isolated by flash chromatography (pentane/Et_2O 9:1) as an orange solid (0.950 mmol, 99%). **MP**.: 89 °C. **FT IR** (neat) ν (cm^{-1}): 3066, 2954, 2869, 1584, 1550, 1500, 1453, 1364, 1266, 1180, 1092, 972, 898, 865, 814, 770, 681, 594. **^{1}H NMR** (300 MHz, $CDCl_3$, 299 K) δ (ppm) = 8.93 – 8.86 (m, 1H), 8.18 (d, *J* = 8.7 Hz, 1H), 7.87 – 7.78 (m, 1H), 7.69 – 7.53 (m, 3H), 4.59 (td, *J* = 10.9 Hz, *J* = 4.5 Hz, 1H), 2.09 – 2.01 (m, 1H), 1.89 – 1.72 (m, 1H), 1.68 – 1.55 (m, 2H), 1.46 – 1.29 (m, 2H), 1.22 – 0.69 (m, 9H), 0.45 (d, *J* = 6.9 Hz, 3H). **^{13}C NMR** (75 MHz, $CDCl_3$, 299 K) δ (ppm) = 139.7 (CH), 136.9 (C), 134.2 (CH), 133.7 (C), 131.4 (C), 128.7 (CH), 128.4 (CH), 127.4 (CH), 126.6 (CH), 95.0 (C), 85.8 (CH), 47.7 (CH), 41.8 (CH_2), 33.8 (CH_2), 31.8 (CH), 25.6 (CH), 23.1 (CH_2), 22.0 (CH_3), 20.8 (CH_3), 15.4 (CH_3). **HRMS** (ESI) *m/z* = 495.0461 calcd. for $C_{20}H_{25}INaO_3S^+$ $[M+Na]^+$, found: 495.0456. **OR**.: $[\alpha]_D^{20}$ = −45.44 (c = 0.87 in $CHCl_3$).

(1*R*,2*S*,5*R*)-2-Isopropyl-5-methylcyclohexyl 4-chloro-2-iodo-5-methylbenzenesulfonate (2l-7)

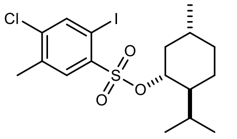

2l-5 was prepared following **GP3** with *L*-menthol (0.96 equiv, 1.10 mmol, 150 mg), $Me_3N{\cdot}HCl$ (1.0 equiv, 0.96 mmol, 92 mg), Et_3N (2.5 equiv, 2.40 mmol, 0.33 mL) and **S-17** (1.5 equiv, 1.44 mmol, 505 mg) in anhydrous CH_2Cl_2 (1.1 mL each). After 1 hr, imidazole (50 mg) was added and the product was isolated by flash chromatography (pentane/Et_2O 9:1) as a colorless solid (452 mg, 0.96 mmol, > 99%). **MP**.: decomposition > 80 °C. **FT IR** (neat) ν (cm^{-1}): 2959, 2929, 2867, 1577, 1540, 1450, 1359, 1211, 1178, 1117, 1178, 1117, 1066, 1037, 919, 905, 875, 820, 805, 715, 637, 599. **^{1}H NMR** (300 MHz, $CDCl_3$, 299 K) δ (ppm) = 8.06 (s, 1H), 7.97 – 7.96 (m, 1H), 4.51 (td, *J* = 10.7 Hz, *J* = 10.6 Hz, 4.6, 1H), 2.40 (s, 3H), 2.15 – 1.93 (m, 2H), 1.76 – 1.57 (m, 2H), 1.53 – 1.19 (m, 3H), 1.07 – 0.76 (m, 8H), 0.60 (d, *J* = 6.9 Hz, 3H). **^{13}C NMR** (75 MHz, $CDCl_3$, 299 K) δ (ppm) = 142.3 (CH), 139.9 (C), 139.6 (C), 137.1 (C), 132.7 (CH), 88.9 (C), 85.9 (CH), 47.7 (CH), 41.8 (CH_2), 33.8 (CH_2), 31.9 (CH), 25.7 (CH), 23.1 (CH_2), 22.0 (CH_3), 21.0 (CH_3), 19.9 (CH_3), 15.4 (CH_3). **HRMS** (ESI) *m/z* = 493.0072 calcd. for $C_{17}H_{24}ClINaO_3S^+$ $[M+Na]^+$, found: 493.0079. **OR**.: $[\alpha]_D^{20}$ = −49.59 (c = 0.71 in $CHCl_3$).

(1*R*,2*S*,5*R*)-2-Isopropyl-5-methylcyclohexyl 4,5-dichloro-2-iodobenzenesulfonate (2l-8)

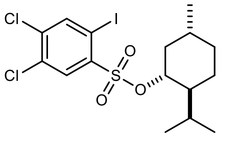

2l-8 was prepared following **GP3** with *L*-menthol (1.0 equiv, 1.10 mmol, 172 mg), $Me_3N{\cdot}HCl$ (1.0 equiv, 1.10 mmol, 105 mg), Et_3N (2.5 equiv, 2.75 mmol, 0.38 mL) and **S-18** (1.5 equiv, 1.65 mmol, 613 mg) in anhydrous CH_2Cl_2 (1.1 mL each). After 1 hr, imidazole (100 mg) was added and the product was isolated by flash chromatography (pentane/Et_2O 9:1) as a colorless solid (433 mg, 0.88 mmol, 80%). **MP**.: decomposition > 95 °C. **FT IR** (neat) ν (cm^{-1}): 3099, 2963, 2928, 2868, 2849, 1559, 1532, 1457, 1439, 1363, 1325, 1309, 1250, 1181, 1146, 1112, 1048, 1005, 984, 935, 917, 900, 879, 848, 803, 683, 633, 618, 603, 569. **^{1}H NMR** (300 MHz, $CDCl_3$, 299 K) δ (ppm) = 8.23 – 8.08 (m, 2H), 4.56 (td, *J* = 10.8 Hz, *J* = 4.6 Hz, 1H), 2.13 – 1.92 (m, 2H), 1.76 – 1.60 (m, 2H), 1.55 – 1.18 (m, 3H), 1.11 – 0.75 (m, 8H), 0.63 (d, *J* = 6.9 Hz, 3H). **^{13}C NMR**

(75 MHz, $CDCl_3$, 299 K) δ (ppm) = 143.6 (CH), 141.2 (C), 138.1 (C), 133.5 (C), 132.0 (CH), 89.8 (C), 86.7 (CH), 47.7 (CH), 41.8 (CH_2), 33.7 (CH_2), 31.9 (CH), 25.8 (CH), 23.1 (CH_2), 22.0 (CH_3), 20.9 (CH_3), 15.5 (CH_3). **HRMS** (ESI) *m/z* = 512.9525 calcd. for $C_{16}H_{21}Cl_2INaO_3S^+$ $[M+Na]^+$, found: 512.9526. **OR**.: $[\alpha]_D^{20}$ = −36.95 (c = 1.0 in $CHCl_3$).

(1*R*,2*S*,5*R*)-2-Isopropyl-5-methylcyclohexyl 2-iodo-4-methylbenzenesulfonate (2l-9)

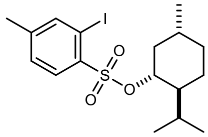

2l-9 was prepared following **GP3** with *L*-menthol (1.0 equiv, 1.10 mmol, 172 mg), $Me_3N·HCl$ (1.0 equiv, 1.10 mmol, 105 mg), Et_3N (2.5 equiv, 2.75 mmol, 0.38 mL) and **S-19** (1.5 equiv, 1.65 mmol, 522 mg) in anhydrous CH_2Cl_2 (1.1 mL each). After 24 hr, imidazole (50 mg) was added and the product was isolated by flash chromatography (pentane/Et_2O 9:1) as a slightly yellow oil (471 mg, 1.08 mmol, 98%). **FT IR** (neat) ν (cm^{-1}): 2953, 2927, 2869, 1588, 1555, 1455, 1356, 1262, 1213, 1177, 1110, 1027, 938, 902, 870, 843, 816, 802, 685, 667, 655. **^{1}H NMR** (300 MHz, $CDCl_3$, 299 K) δ (ppm) = 7.98 (d, *J* = 8.1 Hz, 1H), 7.94 (dd, *J* = 1.7 Hz, *J* = 0.8 Hz, 1H), 7.30 – 7.25 (m, 1H), 4.46 (td, *J* = 10.7 Hz, *J* = 4.6 Hz, 1H), 2.37 (s, 3H), 2.11 – 2.00 (m, 2H), 1.72 – 1.59 (m, 2H), 1.52 – 1.17 (m, 3H), 1.07 – 0.75 (m, 8H), 0.56 (d, *J* = 6.9 Hz, 3H). **^{13}C NMR** (75 MHz, $CDCl_3$, 299 K) δ (ppm) = 145.1 (C), 143.5 (CH), 138.5 (C), 130.9 (CH), 129.0 (CH), 92.6 (C), 85.3 (CH), 47.7 (CH), 41.8 (CH_2), 33.9 (CH_2), 31.9 (CH), 25.7 (CH), 23.2 (CH), 22.0 (CH_3), 21.9 (CH_3), 21.0 (CH_3), 20.9 (CH_3), 15.4 (CH_3). **HRMS** (ESI) *m/z* = 459.0461 calcd. for $C_{17}H_{25}INaO_3S^+$ $[M+Na]^+$, found: 459.0477. $[\alpha]_D^{20}$ = −44.6 (c = 2.1 in $CHCl_3$).

2-(Benzylthio)-3-iodopyridine (S-23)

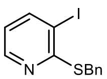

S-23 was prepared following a literature known procedure (*17*). 2-Fluoro-3-iodopyridine (1.0 equiv, 22.4 mmol, 5.00 g), benzylmercaptan (1.0 equiv, 22.4 mmol, 2.95 mL) and K_2CO_3 (1.1 equiv, 24.6 mmol, 3.41 g) were stirred at 90 °C in MeCN (30 mL) for 18 hrs. After the mixture was allowed to cool to room temperature, the solvent was removed *in vacuo* and the residue was taken up in CH_2Cl_2 (200 mL) and H_2O (20 mL). The layers were separated and the aqueous layer was extracted with CH_2Cl_2 (100 mL). The combined organic layers were washed with brine (10 mL), dried over $MgSO_4$ and the solvent was removed *in vacuo*. Pure **S-23** was obtained by flash column chromatography (pentane/Et_2O 1:0 → 99:1) as a colorless solid (5.00 g, 15.3 mmol, 68%). **MP**.: 55 °C. **FT IR** (neat) ν (cm^{-1}): 3086, 3040, 2931, 1598, 1557, 1533, 1494, 1450, 1425, 1378, 1243, 1226, 1193, 1142, 1116, 1060, 1030, 998, 970, 913, 845, 788, 777, 744, 726, 712, 693, 638. **^{1}H NMR** (300 MHz, $CDCl_3$, 299 K) δ (ppm) = 8.40 (dd, *J* = 4.7 Hz, *J* = 1.6 Hz, 1H), 7.87 (dd, *J* = 7.8 Hz, *J* = 1.6 Hz, 1H), 7.46 – 7.37 (m, 2H), 7.33 – 7.18 (m, 3H), 6.68 (dd, *J* = 7.7 Hz, *J* = 4.7 Hz, 1H), 4.39 (s, 2H). **^{13}C NMR** (75 MHz, $CDCl_3$, 299 K) δ (ppm) = 161.6 (C), 148.2 (CH), 145.9 (CH), 137.5 (C), 129.3 (CH), 128.5 (CH), 127.2 (CH), 120.3 (CH), 93.2 (C), 37.3 (CH_2). **HRMS** (ESI) *m/z* = 359.9471 calcd. for $C_{12}H_{10}INNaS^+$ $[M+Na]^+$, found: 349.9470.

(1*R*,2*S*,5*R*)-2-Isopropyl-5-methylcyclohexyl 3-iodopyridine-2-sulfonate (2l-10)

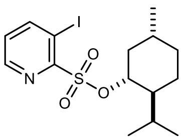

S-23 (1.0 equiv, 1.53 mmol, 500 mg) was dissolved in a mixture of conc. HCl (0.75 mL), H_2O (1.2 mL) and CH_2Cl_2 (5.0 mL). At 0 °C, NaOCl (13% in H_2O, 4.4 equiv, 6.73 mmol, 3.2 mL, diluted with additional 3 mL H_2O) was added over a period of 10 min. The mixture was stirred for 20 min and the aqueous layer extracted with CH_2Cl_2 (3 x 50 mL).

The combined organic layers were washed with diluted $NaHCO_3$ solution and diluted Na_2SO_3 solution (10 mL each) and dried over Na_2SO_4. To the crude sulfonyl chloride, obtained by removal of the solvent *in vacuo*, *L*-menthol (0.67 equiv, 1.02 mmol, 155 mg) was added at 0 °C, followed by addition Et_3N (2.5 equiv, 3.76 mmol, 0.50 mL) and CH_2Cl_2 (1.0 mL). The slurry was stirred for 4 hrs and was the transferred onto a column. The pure sulfonate was obtained by flash column chromatography (pentane/Et_2O 4:1) as a colorless solid (280 mg, 0.661 mmol, 99% relative to *L*-menthol). **MP**.: Decomposition > 50 °C. **FT IR** (neat) ν (cm^{-1}): 2953, 2925, 2866, 1545, 1450, 1420, 1356, 1273, 1221, 1179, 1126, 1053, 1013, 907, 874, 826, 809, 749, 738, 634, 594. **^{1}H NMR** (300 MHz, $CDCl_3$, 299 K) δ (ppm) = 8.69 (dd, *J* = 4.5 Hz, *J* = 1.5 Hz, 1H), 8.44 (dd, *J* = 8.0 Hz, *J* = 1.5 Hz, 1H), 7.21 (dd, *J* = 8.0 Hz, *J* = 4.5 Hz, 1H), 4.66 (td, *J* = 10.8 Hz, *J* = 4.6 Hz, 1H), 2.16 – 1.97 (m, 2H), 1.73 – 1.58 (m, 2H), 1.56 – 1.34 (m, 2H), 1.32 – 1.16 (m, 1H), 1.10 – 0.75 (m, 8H), 0.58 (d, *J* = 6.9 Hz, 3H). **^{13}C NMR** (75 MHz, $CDCl_3$, 299 K) δ (ppm) = 156.5 (C), 151.2 (CH), 147.9 (CH), 127.6 (CH), 89.8 (C), 87.0 (CH), 47.8 (CH), 41.9 (CH_2), 33.8 (CH_2), 31.8 (CH), 25.8 (CH), 23.2 (CH_2), 22.0 (CH_3), 21.0 (CH_3), 15.5 (CH_3). **HRMS** (ESI) *m/z* = 869.0622 calcd. for $C_{30}H_{44}I_2N_2NaO_6S_2^+$ $[2M+Na]^+$, found: 869.0655. $[\alpha]_D^{20}$ = −55.7 (c = 1.0 in $CHCl_3$).

Dicyclohexylmethyl 2-iodobenzenesulfonate (2m)

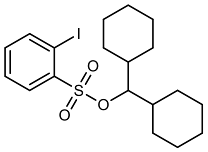

Sulfonate **2m** was prepared following **GP4** with **1m** (1.0 equiv, 0.50 mmol, 98 mg), **S-11** (1.2 equiv, 0.60 mmol, 182 mg), Me_3N (2.0 M in THF, 1.5 equiv, 0.75 mmol, 0.38 mL) and *n*BuLi (1.6 M in hexane, 1.2 equiv, 0.60 mmol, 0.38 mL) in anhydrous THF (1.9 mL each). After a reaction time of 2 hrs, imidazole (50 mg) was added and the desired sulfonate was obtained by flash column chromatography (pentane/Et_2O 20:1) as a colorless oil (186 mg, 0.403 mmol, 81%). The neat compound is unstable and was stored in Et_2O. **FT IR** (neat) ν (cm^{-1}): 2927, 2852, 1570, 1448, 1362, 1258, 1182, 1125, 1100, 1016, 981, 893, 851, 759, 733, 704, 658, 607, 592. **^{1}H NMR** (300 MHz, $CDCl_3$, 299 K) δ (ppm) = 8.10 (dd, *J* = 7.9 Hz, *J* = 1.2 Hz, 1H), 8.04 (dd, *J* = 7.9 Hz, *J* = 1.7 Hz, 1H), 7.47 (ddd, *J* = *J* = 7.7 Hz, *J* = 1.2 Hz, 1H), 7.20 (ddd, *J* = *J* = 7.7 Hz, *J* = 1.7 Hz, 1H), 4.45 (t, *J* = 5.4 Hz, 1H), 1.86 – 1.43 (m, 12H), 1.31 – 0.85 (m, 10H). **^{13}C NMR** (75 MHz, $CDCl_3$, 299 K) δ (ppm) = 142.7 (CH), 142.2 (CH), 133.6 (CH), 130.5 (CH), 128.1 (CH), 96.1 (CH), 92.4 (C), 39.4 (CH), 30.4 (CH_2), 28.1 (CH_2), 26.4 (CH_2), 26.3 (CH_2), 26.2 (CH_2). **HRMS** (ESI) *m/z* = 485.0618 calcd. for $C_{19}H_{27}INaO_3S^+$ $[M+Na]^+$, found: 485.0633.

1-Cyclohexyl-2,2-dimethylpropyl 2-iodobenzenesulfonate (2n)

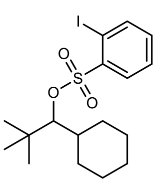

Sulfonate **2n** was prepared following **GP4** with **1n** (1.0 equiv, 1.0 mmol, 170 mg), **S-11** (1.2 equiv, 1.2 mmol, 363 mg), Me_3N (2.0 M in THF, 1.5 equiv, 1.5 mmol, 0.75 mL) and *n*BuLi (1.57 M in hexane, 1.2 equiv, 1.2 mmol, 0.75 mL) in anhydrous THF (3.75 mL each). After a reaction time of 2 hrs, the desired sulfonate was obtained by flash column chromatography (pentane/Et_2O 9:1) as a colorless oil (382 mg, 0.875 mmol, 88%). The neat compound is unstable and was stored in Et_2O. **FT IR** (neat) ν (cm^{-1}): 2926, 2853, 1571, 1480, 1450, 1399, 1358, 1341, 1256, 1180, 1125, 1099, 1016, 930, 902, 890, 848, 758, 732, 704, 642, 595. **^{1}H NMR** (400 MHz, $CDCl_3$, 299 K) δ (ppm) = 8.10 (dd, *J* = 7.9 Hz, *J* = 1.2 Hz, 1H), 8.04 (dd, *J* = 7.9 Hz, *J* = 1.6 Hz, 1H), 7.48 (ddd, *J* = *J* = 7.7 Hz, *J* = 1.2 Hz, 1H), 7.21 (ddd, *J* = *J* = 7.7 Hz, *J* = 1.7 Hz, 1H), 4.33 (d, *J* = 2.4 Hz, 1H),

1.85 – 1.48 (m, 6H), 1.36 – 1.08 (m, 4H), 1.05 – 0.95 (m, 1H), 0.89 (s, 9H). **^{13}C NMR** (101 MHz, $CDCl_3$, 299 K) δ (ppm) = 142.6 (CH), 142.0 (C), 133.5 (CH), 130.2 (CH), 128.0 (CH), 98.3 (CH), 92.3 (C), 39.2 (CH), 36.3 (C), 33.4 (CH_2), 29.0 (CH_2), 26.7 (CH_3), 26.6 (CH_2), 26.4 (CH_2), 26.0 (CH_2). **HRMS** (ESI) *m/z* = 459.0461 calcd. for $C_{17}H_{25}INaO_3S^+$ [M+Na]$^+$, found: 459.0466.

2,2-Dimethylnonan-3-yl 2-iodobenzenesulfonate (2o)

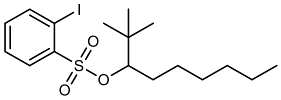

Sulfonate **2o** was prepared following **GP4** with **1o** (1.0 equiv, 2.0 mmol, 345 mg), **S-11** (1.2 equiv, 2.4 mmol, 726 mg), Me_3N (2.0 M in THF, 1.5 equiv, 3.0 mmol, 1.5 mL) and *n*BuLi (1.6 M in hexane, 1.2 equiv, 2.4 mmol, 1.5 mL) in anhydrous THF (7.5 mL each). After a reaction time of 2 hrs, the desired sulfonate was obtained by flash column chromatography (pentane/Et_2O 9:1) as a colorless oil (791 mg, 1.80 mmol, 90%). **FT IR** (neat) ν (cm^{-1}): 2957, 2929, 2871, 2859, 1571, 1480, 1467, 1449, 1399, 1361, 1337, 1276, 1256, 1181, 1125, 1099, 1016, 892, 760, 730, 703, 642, 601, 572. **^{1}H NMR** (300 MHz, $CDCl_3$, 299 K) δ (ppm) = 8.09 (dd, *J* = 7.9 Hz, *J* = 1.2 Hz, 1H), 8.05 (dd, *J* = 7.9 Hz, *J* = 1.6 Hz, 1H), 7.47 (ddd, *J* = *J* = 7.7 Hz, *J* = 1.2 Hz, 1H), 7.20 (ddd, *J* = *J* = 7.7 Hz, *J* = 1.7 Hz, 1H), 4.53 (dd, *J* = 8.6 Hz, *J* = 3.1 Hz, 2H), 1.73 – 1.47 (m, 2H), 1.33 – 1.03 (m, 7H), 0.92 (s, 9H), 0.83 (t, *J* = 7.0 Hz, 3H). **^{13}C NMR** (75 MHz, $CDCl_3$, 299 K) δ (ppm) = 142.8 (CH), 142.4 (C), 133.6 (CH), 130.3 (CH), 128.1 (CH), 95.6 (CH), 92.4 (C), 35.6 (CH), 31.7 (CH_2), 30.7 (CH_2), 29.1 (CH_2), 26.8 (CH_2), 26.4 (CH_3), 22.6 (CH_2), 14.2 (CH_3). **HRMS** (ESI) *m/z* = 461.0618 calcd. for $C_{17}H_{27}INaO_3S^+$ [M+Na]$^+$, found: 461.0601.

2,2,6-Trimethylheptan-3-yl 2-iodobenzenesulfonate (2p)

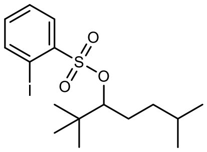

Sulfonate **2p** was prepared following **GP4** with **1p** (1.0 equiv, 1.0 mmol, 158 mg), **S-11** (1.2 equiv, 1.2 mmol, 363 mg), Me_3N (2.0 M in THF, 1.5 equiv, 1.5 mmol, 0.75 mL) and *n*BuLi (1.57 M in hexane, 1.2 equiv, 1.2 mmol, 0.76 mL) in anhydrous THF (3.75 mL each). After a reaction time of 2 hrs, the desired sulfonate was obtained by flash column chromatography (pentane/Et_2O 9:1) as a colorless solid (414 mg, 0.976 mmol, 98%). The neat compound is unstable and was stored in Et_2O. **FT IR** (neat) ν (cm^{-1}): 2957, 2871, 1570, 1467, 1449, 1399, 1360, 1336, 1256, 1218, 1179, 1125, 1099, 1039, 1015, 975, 948, 888, 813, 758, 737, 703, 642, 600. **^{1}H NMR** (400 MHz, $CDCl_3$, 299 K) δ (ppm) = 8.09 (dd, *J* = 7.9 Hz, *J* = 1.2 Hz, 1H), 8.05 (dd, *J* = 7.9 Hz, *J* = 1.7 Hz, 1H), 7.47 (ddd, *J* = *J* = 7.6 Hz, *J* = 1.2 Hz, 1H), 7.21 (ddd, *J* = *J* = 7.7 Hz, *J* = 1.7, 1H), 4.53 – 4.45 (m, 1H), 1.64 – 1.53 (m, 2H), 1.44 – 1.29 (m, 1H), 1.04 – 0.88 (m, 11H), 0.75 – 0.67 (m, 6H). **^{13}C NMR** (101 MHz, $CDCl_3$, 299 K) δ (ppm) = 142.7 (CH), 142.3 (C), 133.6 (CH), 130.4 (CH), 128.1 (CH), 96.0 (CH), 92.4 (C), 35.9 (C), 35.6 (CH_2), 28.4 (CH_2), 27.8 (CH), 26.4 (CH_3), 22.8 (CH_3), 22.1 (CH_3). **HRMS** (ESI) *m/z* = 447.0461 calcd. for $C_{16}H_{25}INaO_3S^+$ [M+Na]$^+$, found: 447.0450.

1-Cyclohexyl-4,4-dimethylpentan-3-yl 2-iodobenzenesulfonate (2q)

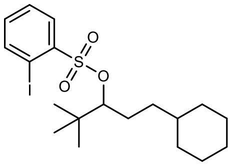

Sulfonate **2q** was prepared following **GP4** with **1q** (1.0 equiv, 0.70 mmol, 139 mg), **S-11** (1.2 equiv, 0.84 mmol, 254 mg), Me_3N (2.0 M in THF, 1.5 equiv, 1.05 mmol, 0.53 mL) and *n*BuLi (1.57 M in hexane, 1.2 equiv, 0.60 mmol, 0.54 mL) in anhydrous THF (2.7 mL each). After a reaction time of 2 hrs the desired sulfonate was obtained by flash column chromatography (pentane/Et_2O 20:1) as a colorless oil (275 mg, 0.592 mmol,

85%). The neat compound is unstable and was stored in Et_2O. **FT IR** (neat) ν (cm^{-1}): 2960, 2922, 2852, 1571, 1449, 1363, 1338, 1181, 1015, 896, 760. **^{1}H NMR** (300 MHz, $CDCl_3$, 299 K) δ (ppm) = 8.10 (dd, *J* = 7.9 Hz, *J* = 1.2, 1H), 8.05 (dd, *J* = 7.9 Hz, *J* = 1.7 Hz, 1H), 7.47 (ddd, *J* = *J* = 7.7 Hz, *J* = 1.2 Hz, 1H), 7.21 (ddd, *J* = *J* = 7.6 Hz, *J* = 1.7 Hz, 1H), 4.49 (dd, *J* = 6.3 Hz, *J* = 5.4 Hz, 1H), 1.69 – 1.40 (m, 7H), 1.20 – 0.89 (m, 15H), 0.81 – 0.57 (m, 2H). **^{13}C NMR** (75 MHz, $CDCl_3$, 299 K) δ (ppm) = 142.8 (CH), 142.5 (C), 133.6 (CH), 130.4 (CH), 128.1 (CH), 96.0 (CH), 92.4 (C), 37.5 (CH), 35.7 (C), 34.5 (CH_2), 33.5 (CH_2), 32.8 (CH_2), 28.0 (CH_2), 26.7 (CH_2), 26.4 (CH_3), 26.4 (CH_2), 26.3 (CH_2). **HRMS** (ESI) *m/z* = 951.1656 calcd. for $C_{38}H_{58}I_2NaO_6S_2^+$ $[2M+Na]^+$, found: 951.1653.

(1*R*,2*R*,5*R*)-2-Ethyl-5-methylcyclohexyl 2-iodobenzenesulfonate (2r-1)

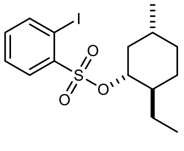

Sulfonate **2r-1** was prepared following **GP3** with **1r** (1.0 equiv, 0.56 mmol, 80 mg), $Me_3N·HCl$ (1.0 equiv, 0.56 mmol, 54 mg), Et_3N (2.5 equiv, 1.4 mmol, 0.19 mL) and **S-11** (1.5 equiv, 0.84 mmol, 255 mg) in anhydrous CH_2Cl_2 (0.50 mL each). After 2 hrs, imidazole (50 mg) was added and the product was isolated by flash chromatography (pentane/Et_2O 9:1) as a slightly yellow solid (181 mg, 0.443 mmol, 79%). **MP**.: 86 °C. **FT IR** (neat) ν (cm^{-1}): 2952, 2930, 2871, 1570, 1450, 1423, 1364, 1275, 1254, 1182, 1125, 1099, 1038, 1016, 960, 929, 900, 873, 817, 760, 729, 703, 641, 593, 569. **^{1}H NMR** (400 MHz, $CDCl_3$, 299 K) δ (ppm) = 8.15 – 8.07 (m, 2H), 7.50 (ddd, *J* = *J* = 7.6 Hz, *J* = 1.2 Hz, 1H), 7.23 (ddd, *J* = *J* = 7.7 Hz, *J* = 1.7 Hz, 1H), 4.36 (td, *J* = 10.8 Hz, *J* = 4.5 Hz, 1H), 1.96 (dddd, *J* = 12.1 Hz, *J* = 5.0 Hz, *J* = 3.2 Hz, *J* = 2.0 Hz, 1H), 1.91 – 1.84 (m, 1H), 1.76 – 1.65 (m, 1H), 1.64 – 1.56 (m, 1H), 1.48 – 1.34 (m, 2H), 1.25 (td, *J* = 12.1 Hz, *J* = 11.1 Hz, 1H), 1.10 – 0.98 (m, 1H), 0.97 – 0.91 (m, 1H), 0.91 – 0.83 (m, 4H), 0.78 (t, *J* = 7.5 Hz, 3H). **^{13}C NMR** (101 MHz, $CDCl_3$, 299 K) δ (ppm) = 142.9 (CH), 141.3 (C), 134.0 (CH), 131.0 (CH), 128.4 (CH), 92.7 (C), 87.9 (CH), 43.6 (CH), 41.4 (CH_2), 33.8 (CH_2), 31.8 (CH), 29.3 (CH_2), 24.5 (CH_2), 22.0 (CH_3), 10.5 (CH_3). **HRMS** (ESI) *m/z* = 431.0148 calcd. for $C_{15}H_{21}INaO_3S^+$ $[M+Na]^+$, found: 431.0142. **OR**.: $[\alpha]_D^{20}$ = −34.40 (c = 0.80 in $CHCl_3$).

(1*R*,2*R*,5*R*)-2-Ethyl-5-methylcyclohexyl 4-chloro-2-iodo-5-methylbenzenesulfonate (2r-2)

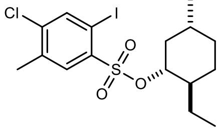

Sulfonate **2r-2** was prepared following **GP3** with **1r** (1.0 equiv, 0.56 mmol, 80 mg), $Me_3N·HCl$ (1.0 equiv, 0.56 mmol, 54 mg), Et_3N (2.5 equiv, 1.4 mmol, 0.19 mL) and **S-17** (1.5 equiv, 0.84 mmol, 294 mg) in anhydrous CH_2Cl_2 (0.50 mL each). After 2 hrs, imidazole (50 mg) was added and the product was isolated by flash chromatography (pentane/Et_2O 9:1) as a slightly yellow solid (255 mg, 0.560 mmol, > 99%). **MP**.: decomposition > 87 °C. **FT IR** (neat) ν (cm^{-1}): 2961, 2924, 2869, 2843, 1577, 1540, 1448, 1374, 1360, 1344, 1330, 1280, 1254, 1211, 1178, 1154, 1115, 1066, 963, 934, 905, 896, 870, 814, 760, 715, 697, 659, 637, 600, 590. **^{1}H NMR** (300 MHz, $CDCl_3$, 299 K) δ (ppm) = 8.06 (s, 1H), 7.96 – 7.95 (m, 1H), 4.38 (td, *J* = 10.7 Hz, *J* = 4.5 Hz, 1H), 2.40 (s, 3H), 2.07 – 1.97 (m, 1H), 1.95 – 1.85 (m, 1H), 1.79 – 1.57 (m, 2H), 1.51 – 1.35 (m, 2H), 1.34 – 1.20 (m, 1H), 1.14 – 0.76 (m, 9H). **^{13}C NMR** (75 MHz, $CDCl_3$, 299 K) δ (ppm) = 142.4 (CH), 139.9 (C), 139.7 (C), 137.1 (C), 132.7 (CH), 88.9 (CH), 88.1 (C), 43.7 (CH), 41.6 (CH_2), 33.8 (CH_2), 31.9 (CH), 29.3 (CH_2), 24.5 (CH_2), 22.0 (CH_3), 19.9 (CH_3), 10.6 (CH_3). **HRMS** (ESI) *m/z* = 478.9915 calcd. for $C_{16}H_{22}ClINaO_3S^+$ $[M+Na]^+$, found: 478.9911. **OR**.: $[\alpha]_D^{20}$ = −32.26 ° (c = 1.1 in $CHCl_3$).

(1*R*,2*R*,5*R*)-2-Ethyl-5-methylcyclohexyl 2-iodo-3,5-dimethylbenzenesulfonate (2r-3)

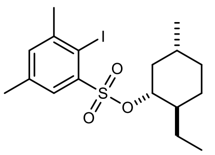

Sulfonate **2r-3** was prepared following **GP3** with **1r** (1.0 equiv, 0.56 mmol, 80 mg), $Me_3N{\cdot}HCl$ (1.0 equiv, 0.56 mmol, 54 mg), Et_3N (2.5 equiv, 1.4 mmol, 0.19 mL) and **S-16** (1.5 equiv, 0.84 mmol, 278 mg) in anhydrous CH_2Cl_2 (0.50 mL each). After 2 hrs, imidazole (50 mg) was added and the product was isolated by flash chromatography (pentane/Et_2O 9:1) as a colorless solid (217 mg, 0.497 mmol, 89%). **MP**.: 94 °C. **FT IR** (neat) ν (cm^{-1}): 2956, 2924, 2860, 1443, 1412, 1380, 1327, 1281, 1261, 1225, 1178, 1148, 1105, 1013, 960, 925, 886, 870, 846, 812, 759, 697, 642, 611, 588, 560. **^{1}H NMR** (300 MHz, $CDCl_3$, 299 K) δ (ppm) = 7.78 (d, *J* = 2.2 Hz, 1H), 7.29 – 7.25 (m, 1H), 4.38 (td, *J* = 10.7 Hz, *J* = 4.5 Hz, 1H), 2.54 (s, 3H), 2.34 (s, 3H), 2.08 – 1.97 (m, 1H), 1.93 – 1.84 (m, 1H), 1.80 – 1.65 (m, 1H), 1.65 – 1.54 (m, 1H), 1.50 – 1.33 (m, 2H), 1.26 (td, *J* = 12.0 Hz, *J* = 11.0 Hz, 1H), 1.15 – 0.74 (m, 9H). **^{13}C NMR** (75 MHz, $CDCl_3$, 299 K) δ (ppm) = 145.4 (C), 141.9 (C), 138.3 (C), 134.7 (CH), 129.5 (CH), 95.5 (C), 87.6 (CH), 43.7 (CH), 41.5 (CH_2), 33.9 (CH_2), 31.9 (CH), 30.4 (CH_3), 29.3 (CH_2), 24.5 (CH_2), 22.0 (CH_3), 20.9 (CH_3), 10.6 (CH_3). **HRMS** (ESI) *m/z* = 459.0461 calcd. for $C_{17}H_{25}INaO_3S^+$ $[M+Na]^+$, found: 459.0501. **OR**.: $[\alpha]_D^{20} = -35.79$ ° (c = 1.2 in $CHCl_3$).

1,1,1-Trichloro-4-methylpentan-2-yl 2-iodobenzenesulfonate (2s)

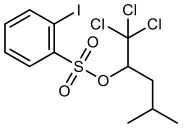

Sulfonate **2s** was prepared following **GP3** using **1s** (1.0 equiv, 1.0 mmol, 206 mg), **S-11** (1.5 equiv, 1.5 mmol, 454 mg), $Me_3N{\cdot}HCl$ (1.0 equiv, 1.0 mmol, 96 mg), Et_3N (2.5 equiv, 2.5 mmol, 0.35 mL) in anhydrous CH_2Cl_2 (0.55 mL each). After 2 hrs, imidazole (100 mg) was added and the product was isolated by flash chromatography (pentane/Et_2O 19:1) as a colorless liquid (335 mg, 0.710 mmol, 71%). **FT IR** (neat) ν (cm^{-1}): 2961, 2931, 2870, 1570, 1448, 1369, 1327, 1257, 1186, 1125, 1100, 1061, 1016, 969, 949, 893, 849, 812, 754, 725, 701, 647, 597, 587. **^{1}H NMR** (300 MHz, $CDCl_3$, 299 K) δ (ppm) = 8.12 (dd, *J* = 7.9 Hz, *J* = 1.2 Hz, 1H), 8.06 (dd, *J* = 8.0 Hz, *J* = 1.6 Hz, 1H), 7.48 (ddd, *J* = *J* = 7.7 Hz, *J* = 1.2 Hz, 1H), 7.24 (ddd, *J* = *J* = 7.7 Hz, *J* = 1.7 Hz, 1H), 5.19 (dd, *J* = 9.4 Hz, *J* = 1.9 Hz, 1H), 2.10 – 1.99 (m, 1H), 1.95 – 1.82 (m, 1H), 1.81 – 1.67 (m, 1H), 1.03 – 0.90 (m, 6H). **^{13}C NMR** (75 MHz, $CDCl_3$, 299 K) δ (ppm) = 143.0 (CH), 141.5 (C), 134.2 (CH), 130.6 (CH), 128.1 (CH), 99.1 (C), 92.4 (C), 89.4 (CH), 40.6 (CH_2), 24.3 (CH), 23.8 (CH_3), 21.2 (CH_3). **HRMS** (ESI) *m/z* = 492.8666 calcd. for $C_{12}H_{14}Cl_3INaO_3S^+$ $[M+Na]^+$, found: 492.8640.

1,1,1-Trichloro-3-cyclopentylpropan-2-yl 2-iodobenzenesulfonate (2t)

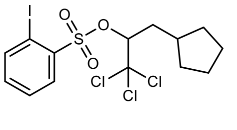

Sulfonate **2t** was prepared following **GP3** using **1t** (1.0 equiv, 1.0 mmol, 232 mg), **S-11** (1.5 equiv, 1.5 mmol, 454 mg), $Me_3N{\cdot}HCl$ (1.0 equiv, 1.0 mmol, 96 mg), Et_3N (2.5 equiv, 2.5 mmol, 0.35 mL) in anhydrous CH_2Cl_2 (0.55 mL each). After 2 hrs, imidazole (100 mg) was added and the product was isolated by flash chromatography (pentane/Et_2O 99:1) as a colorless solid (288 mg, 0.579 mmol, 58%). **MP**.: 122 °C. **FT IR** (neat) ν (cm^{-1}): 3062, 2951, 2865, 1611, 1570, 1480, 1449, 1422, 1364, 1335, 1278, 1256, 1181, 1127, 1032, 1019, 985, 950, 928, 889, 809, 788, 757, 723, 703, 655, 642, 595, 564. **^{1}H NMR** (300 MHz, $CDCl_3$, 299 K) δ (ppm) = 8.11 (dd, *J* = 7.9 Hz, *J* = 1.2 Hz, 1H), 8.06 (dd, *J* = 8.0 Hz, *J* = 1.6 Hz, 1H), 7.48 (ddd, *J* = *J* = 7.7 Hz, *J* = 1.2 Hz, 1H) 7.24 (ddd, *J* = *J* = 7.7 Hz, *J* = 1.6 Hz, 1H), 5.15 (dd, *J* = 9.6 Hz, *J* = 1.8 Hz, 1H), 2.20 (ddd, *J* = 14.3 Hz, *J* = 9.6 Hz, *J* = 3.1 Hz, 1H), 2.07 – 1.71 (m, 4H), 1.69 – 1.41 (m, 5H), 1.22 – 1.05 (m, 2H). **^{13}C NMR**

(75 MHz, $CDCl_3$, 299 K) δ (ppm) = 143.0 (CH), 141.6 (C), 134.2 (CH), 130.6 (CH), 128.0 (CH), 99.0 (C), 92.5 (C), 90.5 (CH), 37.8 (CH_2), 35.7 (CH), 33.4 (CH_2), 31.7 (CH_2), 25.2 (CH_2), 25.1 (CH_2). **HRMS** (ESI) *m/z* = 518.8823 calcd. for $C_{14}H_{16}Cl_3INaO_3S^+$ $[M+Na]^+$, found: 518.8794.

1,1,1-Trichloroheptan-2-yl 2-iodobenzenesulfonate (2u)

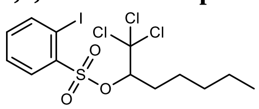

Sulfonate **2u** was prepared following **GP3** using **1u** (1.0 equiv, 1.0 mmol, 220 mg), **S-11** (1.5 equiv, 1.5 mmol, 454 mg), $Me_3N{\cdot}HCl$ (1.0 equiv, 1.0 mmol, 96 mg), Et_3N (2.5 equiv, 2.5 mmol, 0.35 mL) in anhydrous CH_2Cl_2 (0.55 mL each). After 2 hrs, imidazole (100 mg) was added and the product was isolated by flash chromatography (pentane/Et_2O 99:1) as a colorless solid (413 mg, 0.851 mmol, 85%). **MP**.: 91 °C. **FT IR** (neat) ν (cm^{-1}): 3086, 2929, 2860, 1568, 1460, 1433, 1421, 1366, 1332, 1278, 1254, 1236, 1179, 1126, 1098, 1067, 1035, 1017, 986, 970, 926, 876, 816, 798, 774, 756, 736, 718, 700, 643, 587. **^{1}H NMR** (300 MHz, $CDCl_3$, 299 K) δ (ppm) = 8.12 (dd, *J* = 7.9 Hz, *J* = 1.2 Hz, 1H), 8.06 (dd, *J* = 8.0 Hz, *J* = 1.6, 1H), 7.49 (ddd, *J* = *J* = 7.7 Hz, *J* = 1.3 Hz, 1H), 7.24 (ddd, *J* = *J* = 7.7 Hz, *J* = 1.7 Hz, 1H), 5.12 (dd, *J* = 9.2 Hz, *J* = 2.5 Hz, 1H), 2.23 – 1.93 (m, 2H), 1.54 – 1.14 (m, 6H), 0.92 – 0.81 (m, 3H). **^{13}C NMR** (75 MHz, $CDCl_3$, 299 K) δ (ppm) = 143.0 (CH), 141.5 (C), 134.2 (CH), 130.7 (CH), 128.1 (CH), 98.9 (C), 92.5 (C), 90.8 (CH), 31.6 (CH_2), 31.3 (CH_2), 25.3 (CH_2), 22.4 (CH_2), 14.0 (CH_3). **HRMS** (ESI) *m/z* = 506.8823 calcd. for $C_{13}H_{16}Cl_3INaO_3S^+$ $[M+Na]^+$, found: 506.8828.

Methyl 3-(((2-iodophenyl)sulfonyl)oxy)-2,2,5-trimethylhexanoate (2v)

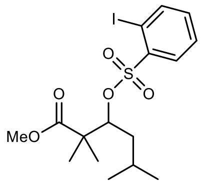

Sulfonate **2v** was prepared following **GP4** with **1v** (1.0 equiv, 0.50 mmol, 94 mg), **S-11** (1.2 equiv, 0.60 mmol, 182 mg), Me_3N (2.0 M in THF, 1.5 equiv, 0.75 mmol, 0.38 mL) and *n*BuLi (1.6 M in hexane, 1.2 equiv, 0.60 mmol, 0.38 mL) in anhydrous THF (1.9 mL each). After a reaction time of 2 hrs, imidazole (50 mg) was added and the desired sulfonate was obtained by flash column chromatography (pentane/Et_2O 9:1) as a colorless oil (129 mg, 0.284 mmol, 57%). **FT IR** (neat) ν (cm^{-1}): 2983, 2955, 2871, 1731, 1570, 1467, 1447, 1391, 1361, 1262, 1181, 1144, 1124, 1059, 1037, 1016, 994, 916, 888, 869, 805, 757, 730, 703, 642, 597, 566. **^{1}H NMR** (300 MHz, $CDCl_3$, 299 K) δ (ppm) = 8.14 – 8.00 (m, 2H), 7.48 (ddd, *J* = 7.7 Hz, *J* = 1.2 Hz, 1H), 7.22 (ddd, *J* = 7.7 Hz, *J* = 1.7 Hz, 1H), 5.15 (dd, *J* = 9.6 Hz, *J* = 1.8 Hz, 1H), 3.59 (s, 3H), 1.71 (ddd, *J* = 14.8 Hz, *J* = 9.6 Hz, *J* = 3.3 Hz, 1H), 1.53 – 1.36 (m, 1H), 1.23 – 1.14 (m, 6H), 1.07 (ddd, *J* = 14.9 Hz, *J* = 10.6 Hz, *J* = 1.8 Hz, 1H), 0.85 (d, *J* = 6.4 Hz, 3H), 0.78 (d, *J* = 6.6 Hz, 3H). **^{13}C NMR** (75 MHz, $CDCl_3$, 299 K) δ (ppm) = 175.5 (C), 142.8 (CH), 142.1 (C), 133.8 (CH), 130.4 (CH), 128.2 (CH), 92.2 (C), 88.2 (CH), 52.2 (CH_3), 47.6 (C), 40.9 (CH_2), 24.4 (CH), 23.8 (CH_3), 23.1 (CH_3), 21.2 (CH_3), 19.8 (CH_3). **HRMS** (ESI) *m/z* = 477.0203 calcd. for $C_{16}H_{23}INaO_5S^+$ $[M+Na]^+$, found: 477.0164.

Methyl 3-(((2-iodophenyl)sulfonyl)oxy)-2,2-dimethyloctanoate (2w)

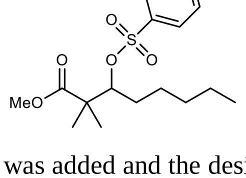

Sulfonate **2w** was prepared following **GP4** with **1w** (1.0 equiv, 1.00 mmol, 202 mg), **S-11** (1.2 equiv, 1.20 mmol, 363 mg), Me_3N (2.0 M in THF, 1.5 equiv, 1.50 mmol, 0.75 mL) and *n*BuLi (1.6 M in hexane, 1.2 equiv, 1.20 mmol, 0.75 mL) in anhydrous THF (3.8 mL each). After a reaction time of 2 hrs, imidazole (100 mg) was added and the desired sulfonate was obtained by flash column chromatography (pentane/Et_2O 9:1) as a

colorless oil (233 mg, 0.497 mmol, 50%). **FT IR** (neat) ν (cm^{-1}): 2954, 2932, 2871, 2861, 1731, 1570, 1434, 1364, 1264, 1181, 1143, 1120, 1016, 981, 902, 810, 794, 760, 732, 703, 668, 642, 594. **^{1}H NMR** (300 MHz, $CDCl_3$, 299 K) δ (ppm) = 8.14 – 7.99 (m, 2H), 7.48 (ddd, *J* = *J* = 7.7 Hz, *J* = 1.2 Hz, 1H), 7.22 (ddd, *J* = *J* = 7.6 Hz, *J* = 1.7 Hz, 1H), 5.06 (dd, *J* = 9.1 Hz, *J* = 2.8 Hz, 1H), 3.58 (s, 3H), 1.77 – 1.59 (m, 1H), 1.50 – 1.34 (m, 1H), 1.28 – 1.06 (m, 11H), 0.87 – 0.73 (m, 3H). **^{13}C NMR** (75 MHz, $CDCl_3$, 299 K) δ (ppm) = 175.6 (C), 142.8 (CH), 142.0 (C), 133.8 (CH), 130.5 (CH), 128.2 (CH), 92.3 (C), 89.9 (CH), 52.2 (CH_3), 47.5 (CH_2), 31.5 (CH_2), 31.4 (CH_2), 26.0 (CH_2), 23.1 (CH_3), 22.4 (CH_2), 19.9 (CH_3), 14.0 (CH_3). **HRMS** (ESI) *m/z* = 491.0360 calcd. for $C_{17}H_{25}INaO_5S^+$ $[M+Na]^+$, found: 491.0407.

***rac* Methyl (2*R*,3*S*)-3-(((2-iodophenyl)sulfonyl)oxy)-2-methoxy-2,5-dimethylhexanoate (2x)**

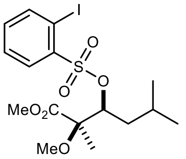

Sulfonate **2x** was prepared following **GP3** with **1x** (1.0 equiv, 0.50 mmol, 102 mg), $Me_3N\cdot HCl$ (1.0 equiv, 0.50 mmol, 48 mg), Et_3N (2.5 equiv, 1.25 mmol, 0.17 mL) and **S-11** (1.5 equiv, 0.75 mmol, 227 mg) in anhydrous CH_2Cl_2 (0.50 mL each). After 12 hrs, the product was isolated by flash chromatography (pentane/Et_2O 4:1) as a colorless oil (177 mg, 0.376 mmol, 75%, *dr*: 7:1). **FT IR** (neat) ν (cm^{-1}): 2956, 2872, 1739, 1570, 1448, 1432, 1363, 1257, 1182, 1137, 1116, 1099, 1059, 1038, 1016, 939, 926, 895, 808, 758, 734, 703, 642. **^{1}H NMR** (300 MHz, $CDCl_3$, 299 K, major isomer) δ (ppm) = 8.15 – 8.02 (m, 2H), 7.55 – 7.42 (m, 1H), 7.28 – 7.15 (m, 1H), 5.06 (dd, *J* = 9.7 Hz, *J* = 2.3 Hz, 1H), 3.66 (s, 3H), 3.21 (s, 3H), 1.74 (ddd, *J* = 14.4 Hz, *J* = 9.7 Hz, *J* = 3.1 Hz, 1H), 1.55 – 1.28 (m, 5H), 0.83 (dd, *J* = 6.4 Hz, *J* = 4.6 Hz, 6H). **^{13}C NMR** (101 MHz, $CDCl_3$, 299 K, major isomer) δ (ppm) = 172.0 (C), 142.7 (CH), 141.7 (C), 133.9 (CH), 130.6 (CH), 128.1 (CH), 92.4 (C), 86.1 (CH), 81.9 (C), 52.6 (CH_3), 52.3 (CH_3), 39.1 (CH_2), 24.2 (CH), 23.7 (CH_3), 21.2 (CH_3), 16.6 (CH_3). **HRMS** (ESI) *m/z* = 493.0152 calcd. for $C_{16}H_{23}INaO_6S^+$ $[M+Na]^+$, found: 493.0146.

***rac* Methyl (2*R*,3*S*)-3-(((2-iodophenyl)sulfonyl)oxy)-2,5-dimethylhexanoate (2y)**

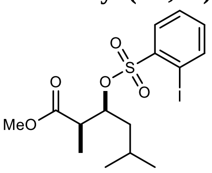

Sulfonate **2y** was prepared following **GP3** with **2y** (1.0 equiv, 0.50 mmol, 87 mg), $Me_3N\cdot HCl$ (1.0 equiv, 0.50 mmol, 48 mg), Et_3N (2.5 equiv, 1.25 mmol, 0.17 mL) and **S-11** (1.5 equiv, 0.75 mmol, 227 mg) in anhydrous CH_2Cl_2 (0.50 mL each). After 2 hrs, the product was isolated by flash chromatography (pentane/CH_2Cl_2 1:1) as a colorless oil (156 mg, 0.354 mmol, 71%, *dr*: 2:1). **FT IR** (neat) ν (cm^{-1}): 2958, 2872, 1735, 1570, 1449, 1435, 1365, 1256, 1182, 1120,1099, 1076, 1038, 1016,955, 888, 754, 727, 704, 686, 642. **^{1}H NMR** (300 MHz, $CDCl_3$, 299 K, mixture of isomers isomers) δ (ppm) = 8.19 – 8.06 (m, 2H), 7.57 – 7.46 (m, 1H), 7.31 – 7.15 (m, 1H), 5.13 – 5.03 (m, 1H), 3.65 – 3.53 (m, 3H), 3.00 (qd, *J* = 7.1 Hz, *J* = 4.4 Hz, 1H, minor isomer), 2.71 (qd, *J* = 7.1 Hz, *J* = 4.1 Hz, 1H, major isomer), 1.79 – 1.40 (m, 3H), 1.24 – 1.16 (m, 3H), 0.92 – 0.71 (m, 6H). **^{13}C NMR** (126 MHz, $CDCl_3$, 299 K, major isomer) δ (ppm) = 173.2 (C), 142.9 (CH), 141.2 (C), 134.0 (CH), 131.0 (CH), 128.3 (CH), 92.6 (C), 84.0 (CH), 52.1 (CH_3), 43.4 (CH), 41.6 (CH_2), 24.6 (CH), 22.7 (CH_3), 22.2 (CH_3), 11.5 (CH_3). **HRMS** (ESI) *m/z* = 463.0047 calcd. for $C_{15}H_{21}INaO_5S^+$ $[M+Na]^+$, found: 463.0040.

Methyl 2,2-difluoro-3-(((2-iodophenyl)sulfonyl)oxy)-5-methylhexanoate (2z)

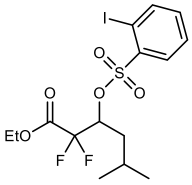

Sulfonate **2z** was prepared following **GP3** with **1z** (1.0 equiv, 0.50 mmol, 105 mg), $Me_3N{\cdot}HCl$ (1.0 equiv, 0.50 mmol, 48 mg), Et_3N (2.5 equiv, 1.25 mmol, 0.17 mL) and **S-11** (1.5 equiv, 0.75 mmol, 227 mg) in anhydrous CH_2Cl_2 (0.50 mL each). After 12 hrs, the product was isolated by flash chromatography (pentane/Et_2O 19:1) as a colorless oil (199 mg, 0.418 mmol, 84%). **FT IR** (neat) ν (cm^{-1}): 2966, 2936, 2873, 1776, 1570, 1469, 1445, 1371, 1312, 1272, 1257, 1223, 1185, 1121, 1096, 1074, 1051, 1040, 1016, 967, 953, 886, 857, 837, 758, 727, 702, 642, 596. **^{1}H NMR** (300 MHz, $CDCl_3$, 299 K) δ (ppm) = 8.12 (dd, *J* = 7.9 Hz, *J* = 1.2 Hz, 1H), 8.06 (dd, *J* = 8.0 Hz, *J* = 1.6 Hz, 1H), 7.50 (ddd, *J* = *J* = 7.7 Hz, *J* = 1.2 Hz, 1H), 7.25 (ddd, *J* = *J* = 7.7 Hz, *J* = 1.6 Hz, 1H), 5.13 (qd, *J* = 10.2 Hz, *J* = 3.0 Hz, 1H), 4.37 – 4.17 (m, 2H), 1.83 (ddd, *J* = 14.2 Hz, *J* = 9.8 Hz, *J* = 4.0 Hz, 1H), 1.77 – 1.65 (m, 1H), 1.51 – 1.40 (m, 1H), 1.33 (t, *J* = 7.2 Hz, 3H), 0.95 – 0.88 (m, 6H). **^{13}C NMR** (75 MHz, $CDCl_3$, 299 K) δ (ppm) = 162.1 (dd, *J* = *J* = 31.3 Hz, C), 143.0 (CH), 140.8 (C), 134.4 (CH), 130.9 (CH), 128.3 (CH), 112.8 (dd, *J* = *J* = 257.4 Hz, C), 92.4 (C), 78.9 (dd, *J* = *J* = 26.8 Hz, CH), 63.7 (CH_2), 37.4 (dd, *J* = *J* = 2.1 Hz, CH_2), 23.8 (CH), 23.4 (CH_3), 21.2 (CH_3), 14.0 (CH_3). **^{19}F{^{1}H} NMR** (282 MHz, $CDCl_3$, 299 K) δ (ppm) = –114.28 (brs, 2F). **HRMS** (ESI) *m/z* = 498.9858 calcd. for $C_{15}H_{19}F_2INaO_5S^+$ [M+Na]$^+$, found: 498.9843.

Ethyl 2,2-difluoro-3-(((2-iodophenyl)sulfonyl)oxy)octanoate (2aa)

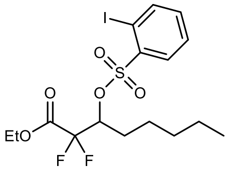

Sulfonate **2aa** was prepared following **GP3** with **1aa** (1.0 equiv, 0.50 mmol, 112 mg), $Me_3N{\cdot}HCl$ (1.0 equiv, 0.50 mmol, 48 mg), Et_3N (2.5 equiv, 1.25 mmol, 0.17 mL) and **S-11** (1.5 equiv, 0.75 mmol, 227 mg) in anhydrous CH_2Cl_2 (0.50 mL each). After 12 hrs, the product was isolated by flash chromatography (pentane/Et_2O 9:1) as a colorless oil (195 mg, 0.400 mmol, 80%). **FT IR** (neat) ν (cm^{-1}): 2959, 2932, 2872, 2864, 1761, 1570, 1448, 1423, 1373, 1312, 1220, 1185, 1126, 1096, 1071, 1053, 1016, 932, 853, 786, 758, 729. **^{1}H NMR** (300 MHz, $CDCl_3$, 299 K) δ (ppm) = 8.12 (dd, *J* = 7.9 Hz, *J* = 1.2 Hz, 1H), 8.07 (dd, *J* = 8.0 Hz, *J* = 1.6 Hz, 1H), 7.51 (ddd, *J* = *J* = 7.7 Hz, *J* = 1.2 Hz, 1H), 7.30 – 7.22 (m, 1H), 5.05 (dddd, *J* = 11.2 Hz, *J* = 9.7 Hz, *J* = 8.7 Hz, *J* = 4.0 Hz, 1H), 4.26 (qd, *J* = 7.1 Hz, *J* = 1.8 Hz, 2H), 1.92 – 1.64 (m, 2H), 1.39 – 1.17 (m, 9H), 0.90 – 0.79 (m, 3H). **^{13}C NMR** (75 MHz, $CDCl_3$, 299 K) δ (ppm) = 162.1 (dd, *J* = *J* = 31.3 Hz, C), 143.0 (CH), 140.7 (C), 134.4 (CH), 134.0 (CH), 128.3 (CH), 112.7 (dd, *J* = 258.4 Hz, *J* = 256.3 Hz, C), 92.4 (C), 80.2 (dd, *J* = 28.3 Hz, *J* = 25.7 Hz, CH), 63.7 (CH_2), 31.3 (CH_2), 28.4 (dd, *J* = *J* = 2.3 Hz, CH_2), 24.4 (CH_2), 22.3 (CH_2), 14.0 (2 x CH_3). **^{19}F{^{1}H} NMR** (282 MHz, $CDCl_3$, 299 K) δ (ppm) = −113.39 (d, *J* = 266.3 Hz, 1F), −115.22 (d, *J* = 266.3 Hz, 1F). **HRMS** (ESI) *m/z* = 513.0015 calcd. for $C_{16}H_{21}F_2INaO_5S^+$ [M+Na]$^+$, found: 513.0010.

Methyl (3-(((2-iodophenyl)sulfonyl)oxy)-2,2-dimethyloctanoyl)glycinate (2ab)

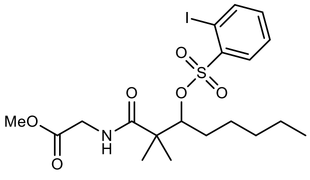

Methyl (3-hydroxy-2,2-dimethyloctanoyl)glycinate (1.0 equiv, 0.887 mmol, 230 mg), sulfonyl chloride **S-11** (2.0 equiv, 1.77 mmol, 537 mg) and $Me_3N{\cdot}HCl$ (2.0 equiv, 1.77 mmol, 170 mg) and Et_3N (3.0 equiv, 2.66 mmol, 0.37 mL) were combined in anhydrous CH_2Cl_2 (1.8 mL) and the mixture was stirred for 18 hrs. The reaction mixture was directly transferred onto a column

and the product was isolated by flash chromatography (pentane/EtOAc 7:3) as a colorless solid (186 mg, 0.354 mmol, 40%). Starting material was recovered in 59% yield (135 mg, 0.521 mmol). **MP**.: 120 °C. **FT IR** (neat) ν (cm^{-1}): 3392, 2956, 2931, 2861, 2364, 1735, 1653, 1526, 1457, 1437, 1364, 1241, 1209, 1180, 1124, 1099, 1045, 1016, 983, 901, 761, 733, 704. **^{1}H NMR** (400 MHz, $CDCl_3$, 299 K) δ (ppm) = 8.11 – 8.05 (m, 2H), 7.51 (ddd, *J* = *J* = 7.7 Hz, *J* = 1.2 Hz, 1H), 7.25 – 7.20 (m, 1H), 6.38 (t, *J* = 4.7 Hz, 1H), 4.96 (dd, *J* = 9.6 Hz, *J* = 2.5 Hz, 1H), 4.02 – 3.88 (m, 1H), 3.84 – 3.69 (m, 4H), 1.76 – 1.61 (m, 2H), 1.57 – 1.46 (m, 1H), 1.32 – 1.04 (m, 11H), 0.83 – 0.72 (m, 3H). **^{13}C NMR** (101 MHz, $CDCl_3$, 299 K) δ (ppm) = 174.9 (C), 170.3 (C), 142.7 (CH), 141.8 (C), 134.0 (CH), 130.8 (CH), 128.4 (CH), 92.3 (C), 91.6 (CH), 52.5 (CH_3), 47.0 (C), 41.4 (CH_2), 31.5 (CH_2), 31.1 (CH_2), 26.1 (CH_2), 22.9 (CH_3), 22.5 (CH_2), 22.2 (CH_3), 14.0 (CH_3). **HRMS** (ESI) *m/z* = 548.0574 calcd. for $C_{19}H_{28}INNaO_6S^+$ $[M+Na]^+$, found: 548.0562.

2-Cyclopentylethyl benzenesulfonate (S-24)

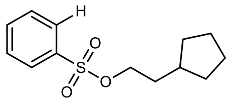

Sulfonate **S-24** was prepared following **GP5** using **S-22** (1.0 equiv, 0.290 mmol, 110 mg), TTMSS (1.4 equiv, 0.406 mmol, 125 µL) and AIBN (0.3 equiv, 87.1 µmol, 14.3 mg) in benzene (10.0 mL). Upon removal of the solvent *in vacuo*, the compound was obtained by flash column chromatography (pentane/CH_2Cl_2 3:2) as a colorless oil (44 mg, 17 µmol, 60%). **FT IR** (neat) ν (cm^{-1}): 2951, 2868, 1468, 1448, 1358, 1312, 1292, 1185, 1097, 958, 926, 837, 775, 752, 715, 688, 617, 584. **^{1}H NMR** (300 MHz, $CDCl_3$, 299 K) δ (ppm) = 7.97 – 7.85 (m, 2H), 7.71 – 7.59 (m, 1H), 7.62 – 7.49 (m, 2H), 4.07 (t, *J* = 6.6 Hz, 2H), 1.88 – 1.38 (m, 9H), 1.12 – 0.93 (m, 2H). **^{13}C NMR** (75 MHz, $CDCl_3$, 299 K) δ (ppm) = 136.5 (C), 133.7 (CH), 129.3 (CH), 128.0 (CH), 70.6 (CH_2), 36.4 (CH), 35.1 (CH_2), 32.5 (CH_2), 25.1 (CH_2). **HRMS** (ESI) *m/z* = 277.0869 calcd. for $C_{13}H_{18}NaO_3S^+$ $[M+Na]^+$, found: 277.0877.

2,2,5-Trimethyl-5-phenylhexan-3-ol (3a-1)

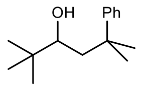

Alcohol **3a-1** was prepared following **GP5** using **2a-1** (1.0 equiv, 0.329 mmol, 135 mg), TTMSS (1.4 equiv, 0.461 mmol, 142 µL) and AIBN (0.3 equiv, 98.7 µmol, 16.2 mg) in benzene (11.4 mL). Upon removal of the solvent *in vacuo*, the desired compound was obtained by flash column chromatography over basic Al_2O_3 (pentane/CH_2Cl_2 7:3) as a colorless solid (37 mg, 0.13 mmol, 49%). **MP**.: 49 °C. **FT IR** (neat) ν cm^{-1}:3602, 3089, 3058, 2960, 2870, 1601, 1497, 1478, 1444, 1387, 1365, 1306, 1263, 1240, 1176, 1144, 1104, 1080, 1067, 1032, 995, 931, 878, 837, 760, 699, 563. **^{1}H NMR** (300 MHz, $CDCl_3$, 299 K) δ (ppm) = 7.43 – 7.38 (m, 2H), 7.36 – 7.30 (m, 2H), 7.20 (ddt, *J* = 7.8 Hz, *J* = 6.4 Hz, *J* = 1.4 Hz, 1H), 3.15 (dd, *J* = 8.9 Hz, *J* = 1.2 Hz, 1H), 1.87 (dd, *J* = 14.6 Hz, *J* = 1.2 Hz, 1H), 1.65 (dd, *J* = 14.6 Hz, *J* = 8.8 Hz, 1H), 1.41 (s, 3H), 1.39 (s, 3H), 1.02 (brs, 1H), 0.80 (s, 9H). **^{13}C NMR** (75 MHz, $CDCl_3$, 299 K) δ (ppm) = 149.0 (CH), 128.5 (CH), 126.2 (CH), 126.0 (C), 76.9 (CH), 47.0 (CH_2), 37.2 (C), 35.1 (C), 30.4 (CH_3), 28.6 (CH_3), 25.7 (CH_3). **HRMS** (ESI) *m/z* = 243.1719 calcd. for $C_{15}H_{24}NaO^+$ $[M+Na]^+$, found: 243.1721.

2,2,5-Trimethyl-5-(m-tolyl)hexan-3-ol (3a-2)

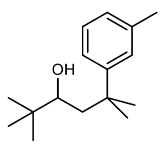

Alcohol **3a-2** was prepared following **GP5** using **2a-2** (1.0 equiv, 0.448 mmol, 190 mg), TTMSS (1.4 equiv, 0.627 mmol, 193 µL) and AIBN (0.3 equiv, 0.134 mmol, 22.1 mg) in benzene (15.5 mL). Upon removal of the solvent *in vacuo*, the desired compound was

obtained by flash column chromatography over basic Al_2O_3 (pentane/CH_2Cl_2 7:3) as a colorless oil (57 mg, 0.24 mmol, 54%). **FT IR** (neat) ν (cm^{-1}): 3593, 2959, 2870, 1605, 1478, 1386, 1364, 1305, 1238, 1174, 1143, 1091, 1067, 1036, 995, 931, 890, 860, 784, 706. **^{1}H NMR** (300 MHz, $CDCl_3$, 299 K) δ (ppm) = 7.28 – 7.17 (m, 3H), 7.05 – 6.99 (m, 1H), 3.17 (dd, *J* = 8.9 Hz, *J* = 1.2 Hz, 1H), 2.40 – 2.32 (m, 3H), 1.86 (dd, *J* = 14.6 Hz, *J* = 1.2 Hz, 1H), 1.66 (dd, *J* = 14.6 Hz, *J* = 8.8 Hz, 1H), 1.39 (s, 3H), 1.38 (s, 3H), 1.00 (brs, 1H), 0.82 (s, 9H). **^{13}C NMR** (75 MHz, $CDCl_3$, 299 K) δ (ppm) = 148.9 (C), 138.0 (C), 128.4 (CH), 126.9 (CH), 126.8 (CH), 123.2 (CH), 76.9 (CH), 47.0 (CH_2), 37.0 (C), 35.1 (C), 30.6 (CH_3), 28.5 (CH_3), 25.8 (CH_3), 22.0 (CH_3). **HRMS** (ESI) *m/z* = 257.1876 calcd. for $C_{16}H_{26}NaO^+$ $[M+Na]^+$, found: 257.1881.

5-(3-Chlorophenyl)-2,2,5-trimethylhexan-3-ol (3a-3)

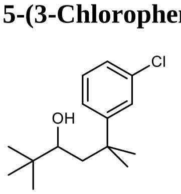

Alcohol **3a-3** was prepared following **GP5** using **2a-3** (1.0 equiv, 0.463 mmol, 206 mg), TTMSS (1.4 equiv, 0.649 mmol, 200 µL) and AIBN (0.3 equiv, 0.139 mmol, 22.8 mg) in benzene (16.0 mL). Upon removal of the solvent *in vacuo*, the desired compound was obtained by flash column chromatography over basic Al_2O_3 (pentane/CH_2Cl_2 4:1) as a colorless oil (74 mg, 0.29 mmol, 63%). **FT IR** (neat) ν (cm^{-1}): 3603, 3475, 2958, 2870, 1595, 1567, 1467, 1412, 1389, 1365, 1302, 1263, 1205, 1175, 1142, 1115, 1097, 1080, 1033, 998, 930, 879, 845, 780, 759, 698, 671. **^{1}H NMR** (300 MHz, $CDCl_3$, 299 K) δ (ppm) = 7.37 – 7.33 (m, 1H), 7.30 – 7.21 (m, 2H), 7.21 – 7.14 (m, 1H), 3.10 (dd, *J* = 8.8 Hz, *J* = 1.3 Hz, 1H), 1.86 (dd, *J* = 14.7 Hz, *J* = 1.3 Hz, 1H), 1.60 (dd, *J* = 14.7 Hz, *J* = 8.8 Hz, 1H), 1.39 (s, 3H), 1.36 (s, 3H), 1.02 (brs, 1H), 0.80 (s, 9H). **^{13}C NMR** (75 MHz, $CDCl_3$, 299 K) δ (ppm) = 151.7 (C), 134.4 (C), 129.6 (CH), 126.6 (CH), 126.1 (CH), 124.5 (CH), 77.0 (CH), 46.7 (CH_2), 37.5 (C), 35.3 (C), 29.6 (CH_3), 29.3 (CH_3), 25.6 (CH_3). **HRMS** (ESI) *m/z* = 277.1330 calcd. for $C_{15}H_{23}ClNaO^+$ $[M+Na]^+$, found: 277.1334.

2,5,5-Trimethyl-2-phenylheptan-4-ol (3b)

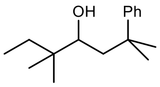

Alcohol **3b** was prepared following **GP5** using **2b** (1.0 equiv, 0.273 mmol, 116 mg), TTMSS (1.4 equiv, 0.382 mmol, 118 µL) and AIBN (0.3 equiv, 81.9 µmol, 13.5 mg) in benzene (9.4 mL). After allowing the reaction mixture to cool to rt, TBAF (1.0 M in THF, 2.0 equiv, 0.55 mmol, 0.55 mL) was added and stirring was continued for 2 hrs. Upon removal of the solvent *in vacuo*, the desired compound was obtained by flash column chromatography (pentane/Et_2O 19:1) as a colorless oil (40 mg, 0.17 mmol, 63%). **FT IR** (neat) ν cm^{-1}: 3061, 2962, 2877, 1601, 1497, 1465, 1444, 1387, 1366, 1306, 1254, 1183, 1103, 1080, 1031, 988, 890, 838, 765, 699. **^{1}H NMR** (300 MHz, $CDCl_3$, 299 K) δ (ppm) = 7.45 – 7.28 (m, 4H), 7.23 – 7.15 (m, 1H), 3.25 (dd, *J* = 8.9 Hz, *J* = 1.3 Hz, 1H), 1.86 (dd, *J* = 14.7 Hz, *J* = 1.3 Hz, 1H), 1.66 (dd, *J* = 14.6 Hz, *J* = 8.9 Hz, 1H), 1.43 – 1.37 (m, 6H), 1.35 – 1.08 (m, 2H), 0.83 – 0.71 (m, 10H). **^{13}C NMR** (75 MHz, $CDCl_3$, 299 K) δ (ppm) = 149.1 (C), 128.5 (CH), 126.2 (CH), 126.0 (CH), 75.5 (CH), 46.8 (CH_2), 37.6 (C), 37.3 (C), 30.8 (CH_2), 30.4 (CH_3), 28.7 (CH_3), 22.3 (CH_3), 22.3 (CH_3), 8.2 (CH_3). **HRMS** (ESI) *m/z* = 257.1876 calcd. for $C_{16}H_{26}NaO^+$ $[M+Na]^+$, found: 257.1883.

2,5-Dimethyl-5-phenylhexan-3-ol (3c)

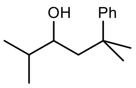

Alcohol **3c** was prepared following **GP5** using **2c** (1.0 equiv, 0.290 mmol, 115 mg), TTMSS (1.4 equiv, 0.406 mmol, 125 µL) and AIBN (0.3 equiv, 87.1 µmol, 14.3 mg) in benzene (10.0 mL). Upon removal of the solvent *in vacuo*, the desired compound was obtained by

flash column chromatography over basic Al_2O_3 (pentane/CH_2Cl_2 1:1) as a colorless oil (20 mg, 97 µmol, 33%). **FT IR** (neat) ν (cm^{-1}): 3581, 3462, 3088, 3058, 2959, 2874, 1602, 1497, 1466, 1444, 1386, 1367, 1337, 1279, 1244, 1183, 1154, 1122, 1096, 1076, 1031, 993, 953, 906, 889, 839. **^{1}H NMR** (300 MHz, $CDCl_3$, 299 K) δ (ppm) = 7.44 – 7.37 (m, 2H), 7.36 – 7.28 (m, 2H), 7.24 – 7.16 (m, 1H), 3.34 (ddd, *J* = 6.8 Hz, *J* = 4.8 Hz, *J* = 3.7 Hz, 1H), 1.79 – 1.72 (m, 2H), 1.60 – 1.45 (m, 1H), 1.40 (s, 3H), 1.38 (s, 3H), 0.86 – 0.76 (m, 6H). **^{13}C NMR** (75 MHz, $CDCl_3$, 299 K) δ (ppm) = 149.0 (CH), 128.5 (CH), 126.1 (CH), 126.0 (C), 73.9 (CH), 49.0 (CH_2), 37.2 (C), 34.7 (CH), 30.4 (CH_3), 28.7 (CH_3), 18.5 (CH_3), 17.2 (CH_3). **HRMS** (ESI) *m/z* = 229.1563 calcd. for $C_{14}H_{22}NaO^+$ $[M+Na]^+$, found: 229.1568.

2,6-Dimethyl-2-phenylheptan-4-ol (3d)

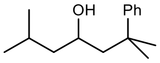

Alcohol **3d** was prepared following **GP5** using **2d** (1.0 equiv, 0.290 mmol, 119 mg), TTMSS (1.4 equiv, 0.406 mmol, 125 µL) and AIBN (0.3 equiv, 87.1 µmol, 14.3 mg) in benzene (10.0 mL). Upon removal of the solvent *in vacuo*, the desired compound was obtained by flash column chromatography over basic Al_2O_3 (pentane/CH_2Cl_2 4:1) as a colorless oil (28 mg, 0.13 mmol, 44%). **FT IR** (neat) ν (cm^{-1}): 3374, 2956, 2927, 2869, 1602, 1496, 1468, 1445, 1385, 1367, 1280, 1135, 1031, 1003, 842, 764, 699. **^{1}H NMR** (300 MHz, $CDCl_3$, 299 K) δ (ppm) = 7.44 – 7.37 (m, 2H), 7.36 – 7.29 (m, 2H), 7.23 – 7.15 (m, 1H), 3.66 (tdd, *J* = 8.4 Hz, *J* = 4.8 Hz, *J* = 2.5 Hz, 1H), 1.84 (dd, *J* = 14.6 Hz, *J* = 8.3 Hz, 1H), 1.73 (dd, *J* = 14.6 Hz, *J* = 2.5 Hz, 1H), 1.69 – 1.55 (m, 1H), 1.40 (s, 3H), 1.39 (s, 3H), 1.32 (ddd, *J* = 14.0 Hz, *J* = 8.5 Hz, *J* = 5.7 Hz, 1H), 1.06 (ddd, *J* = 13.4 Hz, *J* = 8.3 Hz, *J* = 4.8 Hz, 1H), 0.84 – 0.78 (m, 6H). **^{13}C NMR** (101 MHz, $CDCl_3$, 299 K) δ (ppm) = 148.8 (C), 128.4 (CH), 125.9 (CH), 67.5 (CH), 52.6 (CH_2), 48.1 (CH_2), 37.2 (C), 30.5 (CH_3), 28.5 (CH_3), 24.4 (CH), 23.2 (CH_3), 22.2 (CH_3). **HRMS** (ESI) *m/z* = 243.1719 calcd. for $C_{15}H_{24}NaO^+$ $[M+Na]^+$, found: 243.1728.

2,6-Dimethyl-2,6-diphenylheptan-4-ol (3e)

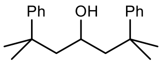

Alcohol **3e** was prepared following **GP5** using **2e** (1.0 equiv, 0.290 mmol, 141 mg), TTMSS (1.4 equiv, 0.406 mmol, 125 µL) and AIBN (0.3 equiv, 87.1 µmol, 14.3 mg) in benzene (10.0 mL). After cooling to rt, TBAF was added (1.0 M solution in THF, 2.0 equiv, 0.58 mL) and the solution was stirred overnight. Upon removal of the solvent *in vacuo*, the desired compound was obtained by flash column chromatography (pentane/Et_2O 50:1 → 9:1) as a colorless oil (29 mg, 98 µmol, 34%). **FT IR** (neat) ν (cm^{-1}): 3059, 2962, 2927, 2871, 1726, 1601, 1496, 1463, 1444, 1386, 1261, 1190, 1106, 1080, 1031, 1001, 763, 698, 580. **^{1}H NMR** (300 MHz, $CDCl_3$, 299 K) δ (ppm) = 7.34 – 7.25 (m, 8H), 7.24 – 7.13 (m, 2H), 3.54 (tt, *J* = 8.1 Hz, *J* = 2.9 Hz, 1H), 1.79 (dd, *J* = 14.5 Hz, *J* = 8.1 Hz, 2H), 1.57 (dd, *J* = 14.5 Hz, *J* = 2.9 Hz, 2H), 1.28 (s, 7H), 1.23 (s, 6H). **^{13}C NMR** (75 MHz, $CDCl_3$, 299 K) δ (ppm) = 149.1 (C), 128.4 (CH), 126.0 (CH), 125.9 (CH), 67.6 (CH), 53.5 (CH_2), 37.3 (C), 29.8 (CH_3), 29.2 (CH_3). **HRMS** (ESI) *m/z* = 319.2032 calcd. for $C_{21}H_{28}NaO^+$ $[M+Na]^+$, found: 319.2028.

2,2,6-Trimethyl-6-phenylheptan-4-ol (3f)

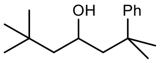

Alcohol **3f** was prepared following **GP5** using **2f** (1.0 equiv, 0.587 mmol, 249 mg), TTMSS (1.4 equiv, 0.822 mmol, 253 µL) and AIBN (0.3 equiv, 0.176 mmol, 28.9 mg) in benzene (20 mL). Upon removal of the solvent *in vacuo*, the desired compound was obtained by flash column chromatography over basic Al_2O_3 (pentane/CH_2Cl_2 19:1 → 9:1) as a colorless liquid (42 mg, 0.18 mmol,

31%). **FT IR** (neat) ν (cm^{-1}): 2951, 2928, 2867, 1601, 1496, 1467, 1446, 1386, 1364, 1249, 1200, 1104, 1081, 1057, 1032, 846, 765, 699. **^{1}H NMR** (300 MHz, $CDCl_3$, 299 K) δ (ppm) = 7.43 – 7.37 (m, 2H), 7.36 – 7.27 (m, 2H), 7.23 – 7.16 (m, 1H), 3.77 (tt, *J* = 8.4 Hz, *J* = 2.7 Hz, 1H), 1.93 (dd, *J* = 14.5 Hz, *J* = 8.6 Hz, 1H), 1.70 (dd, *J* = 14.5 Hz, *J* = 2.6 Hz, 1H), 1.40 (s, 3H), 1.38 (s, 3H), 1.26 (s, 1H), 1.17 (dd, *J* = 14.5 Hz, *J* = 2.8 Hz, 1H), 0.85 (s, 9H). **^{13}C NMR** (75 MHz, $CDCl_3$, 299 K) δ (ppm) = 148.9 (C), 128.6 (CH), 126.1 (CH), 126.1 (CH), 67.6 (CH), 54.4 (CH_2), 52.8 (CH_2), 37.4 (C), 30.9 (CH_3), 30.5 (C), 30.2 (CH_3), 28.5 (CH_3). **HRMS** (ESI) *m/z* = 257.1876 calcd. for $C_{26}H_{26}NaO^+$ $[M+Na]^+$, found: 257.1870.

3,3-Dimethyl-1-(1-phenylcyclohexyl)butan-2-ol (3g)

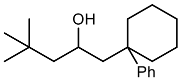

Alcohol **3g** was prepared following **GP5** using **2g** (1.0 equiv, 0.290 mmol, 131 mg), TTMSS (1.4 equiv, 0.406 mmol, 125 µL) and AIBN (0.3 equiv, 87.1 µmol, 14.3 mg) in benzene (10.0 mL). Upon removal of the solvent *in vacuo*, the desired compound was obtained by flash column chromatography over basic Al_2O_3 (pentane/CH_2Cl_2 4:1) as a colorless liquid (49 mg, 0.19 mmol, 65%). **FT IR** (neat) ν (cm^{-1}): 2932, 2859, 1496, 1479, 1452, 1395, 1362, 1276, 1184, 1167, 1072, 1054, 1033, 1006, 957, 924, 912, 758, 733, 627. **^{1}H NMR** (400 MHz, $CDCl_3$, 299 K) δ (ppm) = 7.41 – 7.33 (m, 4H), 7.23 – 7.16 (m, 1H), 3.12 (dd, *J* = 8.7 Hz, *J* = 1.0 Hz, 1H), 2.29 – 2.14 (m, 2H), 1.81 (dd, *J* = 14.7 Hz, *J* = 1.0 Hz, 1H), 1.66 (ddd, *J* = 13.6 Hz, *J* = 10.3 Hz, *J* = 3.4 Hz, 1H), 1.60 – 1.28 (m, 8H), 0.75 (s, 9H). **^{13}C NMR** (101 MHz, $CDCl_3$, 299 K) δ (ppm) = 146.3 (C), 128.8 (CH), 127.2 (CH), 125.9 (CH), 75.8 (CH), 47.5 (CH_2), 40.7 (C), 37.8 (CH_2), 35.9 (CH_2), 35.0 (C), 26.7 (CH_2), 25.7 (CH_3), 22.5 (CH_2), 22.4 (CH_2). **HRMS** (ESI) *m/z* = 283.2032 calcd. for $C_{18}H_{28}NaO^+$ $[M+Na]^+$, found: 283.2056.

3,3-Dimethyl-1-(1-phenylcyclopentyl)butan-2-ol (3h)

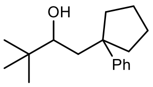

Alcohol **3h** was prepared following **GP5** using **2h** (1.0 equiv, 0.424 mmol, 185 mg), TTMSS (1.4 equiv, 0.594 mmol, 183 µL) and AIBN (0.3 equiv, 0.127 mmol, 20.9 mg) in benzene (14.6 mL). Upon removal of the solvent *in vacuo*, the desired compound was obtained by flash column chromatography over basic Al_2O_3 (pentane/CH_2Cl_2 4:1) as a colorless oil (57 mg, 0.23 mmol, 55%). **FT IR** (neat) ν (cm^{-1}): 3596, 2951, 2870, 1600, 1495, 1478, 1395, 1363, 1237, 1175, 1076, 1058, 1031, 1011, 947, 907, 842, 759, 700. **^{1}H NMR** (300 MHz, $CDCl_3$, 299 K) δ (ppm) = 7.40 – 7.28 (m, 4H), 7.23 – 7.15 (m, 1H), 3.16 – 3.02 (m, 1H), 2.09 – 1.51 (m, 10H), 0.84 – 0.70 (m, 10H). **^{13}C NMR** (75 MHz, $CDCl_3$, 299 K) δ (ppm) = 148.4 (C), 128.5 (CH), 127.0 (CH), 126.0 (CH), 77.4 (CH), 50.4 (C), 44.2 (CH_2), 39.1 (CH_2), 37.2 (CH_2), 34.9 (C), 25.7 (CH_3), 23.2 (CH_2), 22.9 (CH_2). **HRMS** (ESI) *m/z* = 269.1876 calcd. for $C_{17}H_{26}NaO^+$ $[M+Na]^+$, found: 269.1873. The analytical data are in accordance to those reported in the literature (*18*).

3,3-Dimethyl-1-(1-phenylcyclobutyl)butan-2-ol (3i-1)

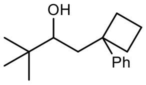

Alcohol **3i-1** was prepared following **GP5** using **2i-1** (1.0 equiv, 0.521 mmol, 220 mg), TTMSS (1.4 equiv, 0.781 mmol, 226 µL) and AIBN (0.3 equiv, 0.156 mmol, 25.7 mg) in benzene (17.9 mL). Upon removal of the solvent *in vacuo*, the desired compound was obtained by flash column chromatography over basic Al_2O_3 (pentane/CH_2Cl_2 4:1) as a colorless liquid (75 mg, 0.32 mmol, 62%). **FT IR** (neat) ν (cm^{-1}): 3596, 2955, 2868, 1601, 1494, 1479, 1465, 1445, 1395, 1363, 1276, 1241, 1175, 1076, 1029, 1005, 991, 891, 758, 701. **^{1}H NMR** (400 MHz, $CDCl_3$, 299 K) δ (ppm)

= 7.36 – 7.31 (m, 2H), 7.24 – 7.16 (m, 3H), 3.08 (dd, *J* = 9.0 Hz, *J* = 1.1 Hz, 1H), 2.49 – 2.35 (m, 2H), 2.35 – 2.26 (m, 1H), 2.20 – 2.00 (m, 3H), 1.92 – 1.81 (m, 1H), 1.80 – 1.72 (m, 1H), 0.92 (brs, 1H), 0.82 (s, 9H). ^{13}**C NMR** (101 MHz, $CDCl_3$, 299 K) δ (ppm) = 150.1 (C), 128.6 (CH), 125.8 (CH), 125.8 (CH), 77.2 (CH), 45.8 (C), 44.9 (CH_2), 34.9 (C), 34.5 (CH_2), 32.7 (CH_2), 25.7 (CH_3), 16.2 (CH_2). **HRMS** (ESI) *m/z* = 255.1719 calcd. for $C_{16}H_{24}NaO^+$ $[M+Na]^+$, found: 255.1722.

1-(1-(4-Chloro-3-methylphenyl)cyclobutyl)-3,3-dimethylbutan-2-ol (3i-2)

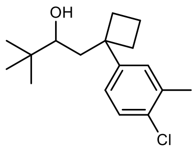

Alcohol **3i-2** was prepared following **GP5** using **2i-2** (1.0 equiv, 0.259 mmol, 122 mg), TTMSS (1.4 equiv, 0.363 mmol, 112 µL) and AIBN (0.3 equiv, 77.9 µmol, 12.8 mg) in benzene (8.9 mL). Upon removal of the solvent *in vacuo*, the desired compound was obtained by flash column chromatography over basic Al_2O_3 (pentane/CH_2Cl_2 1:1) as a colorless oil (37 mg, 0.13 mmol, 51%). **FT IR** (neat) ν (cm^{-1}): 3601, 2955, 2869, 1479, 1446, 1395, 1363, 1324, 1269, 1243, 1209, 1177, 1143, 1083, 1046, 999, 951, 908, 885, 820, 765, 734, 721, 705, 608. ^{1}**H NMR** (300 MHz, $CDCl_3$, 299 K) δ (ppm) = 7.30 – 7.25 (m, 1H), 7.04 (d, *J* = 2.3 Hz, 1H), 6.96 (dd, *J* = 8.2, *J* = 2.3 Hz, 1H), 3.04 (dd, *J* = 9.1 Hz, *J* = 1.2 Hz, 1H), 2.42 – 2.22 (m, 6H), 2.16 – 1.94 (m, 3H), 1.92 – 1.70 (m, 2H), 0.98 (brs, 1H), 0.81 (s, 9H). ^{13}**C NMR** (75 MHz, $CDCl_3$, 299 K) δ (ppm) = 148.7 (C), 136.0 (C), 131.7 (C), 129.1 (CH), 128.5 (CH), 124.8 (CH), 77.3 (CH), 45.6 (C), 44.7 (CH_2), 35.0 (C), 34.4 (CH_2), 33.2 (CH_2), 25.7 (CH_3), 20.4 (CH_3), 16.1 (CH_2). **HRMS** (ESI) *m/z* = 303.1486 calcd. for $C_{17}H_{25}ClNaO^+$ $[M+Na]^+$, found: 303.1489.

5-Ethyl-2,2-dimethyl-5-phenylheptan-3-ol (3j)

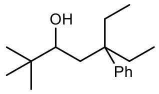

Alcohol **3j** was prepared following **GP5** using **2j** (1.0 equiv, 0.433 mmol, 190 mg), TTMSS (1.4 equiv, 0.607 mmol, 187 µL) and AIBN (0.3 equiv, 0.130 mmol, 21.4 mg) in benzene (14.9 mL). Upon removal of the solvent *in vacuo*, the desired compound was obtained by flash column chromatography over basic Al_2O_3 (pentane/CH_2Cl_2 9:1) as a colorless oil (46 mg, 0.19 mmol, 43%). **FT IR** (neat) ν (cm^{-1}): 3595, 2962, 2878, 1599, 1497, 1479, 1465, 1395, 1378, 1363, 1273, 1235, 1159, 1082, 1060, 1005, 967, 877, 831, 785, 756, 699. ^{1}**H NMR** (300 MHz, $CDCl_3$, 299 K) δ (ppm) = 7.41 – 7.29 (m, 4H), 7.22 – 7.15 (m, 1H), 3.13 (dd, *J* = 9.2 Hz, *J* = 1.2 Hz, 1H), 1.92 – 1.73 (m, 5H), 1.62 (dd, *J* = 14.8 Hz, *J* = 9.3 Hz, 1H), 0.83 – 0.75 (m, 12H), 0.68 (t, *J* = 7.3 Hz, 3H). ^{13}**C NMR** (75 MHz, $CDCl_3$, 299 K) δ (ppm) = 147.2 (C), 128.5 (CH), 127.0 (CH), 125.9 (CH), 76.4 (CH), 42.9 (C), 41.4 (CH_2), 35.3 (C), 29.4 (CH_2), 26.8 (CH_2), 25.8 (CH_3), 8.0 (CH_3), 7.9 (CH_3). **HRMS** (ESI) *m/z* = 271.2032 calcd. for $C_{17}H_{28}NaO^+$ $[M+Na]^+$, found: 271.2039.

***trans*-2-(2-Phenylpropan-2-yl)cyclohexan-1-ol (3k)**

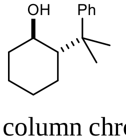

Alcohol **3k** was prepared following **GP5** using **2k** (1.0 equiv, 0.473 mmol, 193 mg), TTMSS (1.4 equiv, 0.662 mmol, 204 µL) and AIBN (0.3 equiv, 0.142 mmol, 20.7 mg) in benzene (16.3 mL). Upon removal of the solvent *in vacuo*, the desired compound was obtained by flash column chromatography over basic Al_2O_3 (pentane/CH_2Cl_2 4:1) as a colorless oil (40 mg, 0.18 mmol, 39%). **FT IR** (neat) ν (cm^{-1}): 3412, 3057, 3025, 2926, 2856, 1599, 1496, 1447, 1386, 1367, 1257, 1233, 1185, 1120, 1051, 988, 955, 905, 874, 849, 761, 780, 761, 699. ^{1}**H NMR** (400 MHz, $CDCl_3$, 299 K) δ (ppm) = 7.43 – 7.39 (m, 2H), 7.36 – 7.30 (m, 2H), 7.22 – 7.16 (m, 1H), 3.54 – 3.46 (m, 1H), 1.93 – 1.82 (m, 1H), 1.82 – 1.62

(m, 4H), 1.43 (s, 3H), 1.36 (brs, 1H), 1.29 (s, 3H), 1.25 – 1.10 (m, 3H), 1.06 – 0.94 (m, 1H). **^{13}C NMR** (101 MHz, $CDCl_3$, 299 K) δ (ppm) = 151.5 (C), 128.6 (CH), 125.9 (CH), 125.9 (CH), 73.6 (CH), 54.8 (CH), 40.1 (C), 36.8 (CH_2), 28.9 (CH_3), 27.0 (CH_2), 26.4 (CH_2), 25.2 (CH_2), 24.3 (CH_3). **HRMS** (ESI) *m/z* = 241.1563 calcd. for $C_{15}H_{22}NaO^+$ $[M+Na]^+$, found: 241.1575.

(1*R*,2*S*,5*R*)-5-Methyl-2-(2-phenylpropan-2-yl)cyclohexan-1-ol (3l-1)

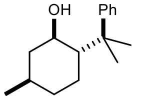

Alcohol **3l-1** was prepared following **GP5** using **2l-1** (1.0 equiv, 0.290 mmol, 123 mg), TTMSS (1.4 equiv, 0.406 mmol, 125 µL) and AIBN (0.3 equiv, 87.1 µmol, 14.3 mg) in benzene (10.0 mL). After cooling to rt, TBAF (1.0 M in THF, 2.0 equiv, 0.58 mmol, 0.58 mL) was added and the solution was stirred for 1 hr. Upon removal of the solvent *in vacuo*, the desired compound was obtained by flash column chromatography (pentane/Et_2O 9:1) as a colorless oil (44 mg, 0.19 mmol, 65%). **^{1}H NMR** (300 MHz, $CDCl_3$, 299 K) δ (ppm) = 7.44 – 7.37 (m, 2H), 7.37 – 7.28 (m, 2H), 7.24 – 7.14 (m, 1H), 3.54 (ddd, *J* = 10.7 Hz, *J* = 9.6 Hz, *J* = 4.3 Hz, 1H), 1.91 – 1.79 (m, 1H), 1.78 – 1.60 (m, 3H), 1.50 – 1.25 (m, 7H), 1.15 – 0.81 (m, 7H). **^{13}C NMR** (75 MHz, $CDCl_3$, 299 K) δ (ppm) = 151.5 (C), 128.6 (CH), 125.9 (CH), 73.1 (CH), 54.4 (CH), 45.6 (CH_2), 40.0 (C), 35.1 (CH_2), 31.7 (CH), 28.8 (CH_3), 26.7 (CH_2), 24.5 (CH_3), 22.1 (CH_3). **HRMS** (ESI) *m/z* = 255.17194 calcd. for $C_{16}H_{24}NaO^+$ $[M+Na]^+$, found: 255.17176. **OR.**: $[\alpha]_D^{20} = -14.27$ (c = 1.5 in $CHCl_3$). The spectroscopic data are in accordance to those reported in the literature (*19*).

(1*R*,2*S*,5*R*)-5-Methyl-2-(2-(m-tolyl)propan-2-yl)cyclohexan-1-ol (3l-2)

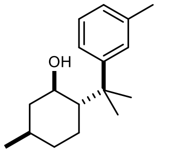

Alcohol **3l-2** was prepared following **GP5** using **2l-2** (1.0 equiv, 0.290 mmol, 127 mg), TTMSS (1.4 equiv, 0.406 mmol, 125 µL) and AIBN (0.3 equiv, 87.1 µmol, 14.3 mg) in benzene (10.0 mL). Upon removal of the solvent *in vacuo*, the desired compound was obtained by flash column chromatography over basic Al_2O_3 (pentane/CH_2Cl_2 7:3) as a colorless oil (37 mg, 0.15 mmol, 52%). **^{1}H NMR** (300 MHz, $CDCl_3$, 299 K) δ (ppm) = 7.24 – 7.17 (m, 3H), 7.05 – 6.97 (m, 1H), 3.54 (ddd, *J* = 10.6 Hz, *J* = 9.5 Hz, *J* = 4.3 Hz, 1H), 2.35 (s, 3H), 1.84 (dtd, *J* = 12.5 Hz, *J* = 4.0 Hz, *J* = 2.4 Hz, 1H), 1.79 – 1.60 (m, 3H), 1.49 – 1.20 (m, 7H), 1.16 – 0.79 (m, 7H). **^{3}C NMR** (75 MHz, $CDCl_3$, 299 K) δ (ppm) = 151.4 (C), 138.0 (C), 128.5 (CH), 126.8 (CH), 126.7 (CH), 123.0 (CH), 73.1 (CH), 54.3 (CH), 45.4 (CH_2), 39.8 (C), 35.1 (CH_2), 31.6 (CH), 29.2 (CH_3), 26.6 (CH_2), 24.0 (CH_3), 22.2 (CH_3), 21.9 (CH_3). **HRMS** (ESI) *m/z* = 269.1876 calcd. for $C_{17}H_{26}NaO^+$ $[M+Na]^+$, found: 269.1886. **OR.**: $[\alpha]_D^{20} = -31.7$ (c = 1.8 in $CHCl_3$). The spectroscopic data are in accordance to those reported in the literature (*19*).

(1*R*,2*S*,5*R*)-2-(2-(3-Chlorophenyl)propan-2-yl)-5-methylcyclohexan-1-ol (3l-3)

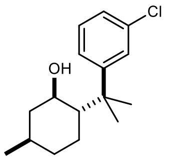

Alcohol 3**l-3** was prepared following **GP5** using **2l-3** (1.0 equiv, 0.290 mmol, 133 mg), TTMSS (1.4 equiv, 0.406 mmol, 125 µL) and AIBN (0.3 equiv, 87.1 µmol, 14.3 mg) in benzene (10.0 mL). Upon removal of the solvent *in vacuo*, the desired compound was obtained by flash column chromatography over basic Al_2O_3 (pentane/CH_2Cl_2 4:1) as a colorless oil (39 mg, 0.15 mmol, 50%). **FT IR** (neat) ν (cm^{-1}): 3600, 3397, 2950, 2918, 2869, 1567, 1594, 1475, 1456, 1411, 1387, 1368, 1257, 1226, 1179, 1092, 1055, 1026, 1010, 998, 965, 937, 891, 878, 838, 781, 764, 701, 682. **^{1}H NMR** (300 MHz, $CDCl_3$, 299 K) δ (ppm) = 7.36 – 7.32 (m, 1H), 7.29 – 7.21 (m, 2H), 7.18 – 7.12 (m, 1H), 3.51 (ddd, *J* = 10.9 Hz, *J* = 9.7 Hz, *J* = 4.2 Hz, 1H), 1.92 – 1.81 (m, 1H), 1.70 – 1.55 (m,

3H), 1.50 – 1.22 (m, 7H), 1.12 – 0.78 (m, 7H). **^{13}C NMR** (75 MHz, $CDCl_3$, 299 K) δ (ppm) = 153.9 (C), 134.3 (C), 129.5 (CH), 126.4 (CH), 125.8 (CH), 124.2 (CH), 73.1 (CH), 54.3 (CH), 46.0 (CH_2), 40.4 (C), 34.9 (CH_2), 31.7 (CH), 27.8 (CH_3), 26.8 (CH_2), 25.7 (CH_3), 22.1 (CH_3). **HRMS** (ESI) *m/z* = 289.1330 calcd. for $C_{16}H_{23}ClNaO^+$ $[M+Na]^+$, found: 289.1335. **OR**.: $[\alpha]_D^{20}$ = −28.26 (c = 1.5 in $CHCl_3$).

(1*R*,2*S*,5*R*)-2-(2-(3-Methoxyphenyl)propan-2-yl)-5-methylcyclohexan-1-ol (3l-4)

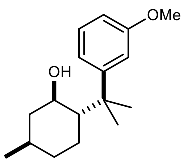

Alcohol **3l-4** was prepared following **GP5** using **2l-4** (1.0 equiv, 0.290 mmol, 131 mg), TTMSS (1.4 equiv, 0.406 mmol, 125 µL) and AIBN (0.3 equiv, 87.1 µmol, 14.3 mg) in benzene (10.0 mL). The solution was allowed to cool to rt and KF (3.0 equiv, 0.87 mmol, 51 mg) was added and the mixture was stirred overnight. Upon removal of the solvent *in vacuo*, the desired compound was obtained by flash column chromatography over basic Al_2O_3 (pentane/CH_2Cl_2 7:3) as a colorless oil (40 mg, 0.15 mmol, 52%). **FT IR** (neat) ν (cm^{-1}): 3436, 2949, 2918, 2869, 2846, 2361, 2336, 1599, 1581, 1488, 1457, 1430, 1387, 1368, 1318, 1291, 1260, 1245, 1214, 1175, 1091, 1052, 1027, 1010, 999, 966, 938, 874, 779, 705, 668, 567. **^{1}H NMR** (300 MHz, $CDCl_3$, 299 K) δ (ppm) = 7.31 – 7.19 (m, 1H), 7.02 – 6.96 (m, 1H), 6.96 – 6.92 (m, 1H), 6.75 – 6.70 (m, 1H), 3.80 (s, 3H), 3.53 (ddd, *J* = 10.8 Hz, *J* = 9.6 Hz, *J* = 4.3 Hz, 1H), 1.85 (dtd, *J* = 12.4 Hz, *J* = 3.9 Hz, *J* = 2.2 Hz, 1H), 1.77 – 1.57 (m, 3H), 1.48 – 1.25 (m, 7H), 1.22 (brs, 1H), 1.11 – 0.76 (m, 6H). **^{13}C NMR** (75 MHz, $CDCl_3$, 299 K) δ (ppm) = 159.8 (C), 153.4 (C), 129.5 (CH), 118.4 (CH), 112.8 (CH), 110.4 (CH), 73.1 (CH), 55.3 (CH_3), 54.3 (CH), 45.5 (CH_2), 40.0 (C), 35.1 (CH_2), 31.7 (CH), 28.8 (CH_3), 26.6 (CH_2), 24.5 (CH_3), 22.1 (CH_3). **HRMS** (ESI) *m/z* = 285.1825 calcd. for $C_{17}H_{26}NaO_2^+$ $[M+Na]^+$, found: 285.1835. **OR**.: $[\alpha]_D^{20}$ = −30.98 (c = 1.7 in $CHCl_3$).

(1*R*,2*S*,5*R*)-2-(2-(3,5-Dimethylphenyl)propan-2-yl)-5-methylcyclohexan-1-ol (3l-5)

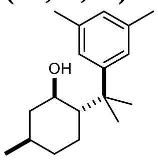

Alcohol **3l-5** was prepared following **GP5** using **2l-5** (1.0 equiv, 0.290 mmol, 131 mg), TTMSS (1.4 equiv, 0.406 mmol, 125 µL) and AIBN (0.3 equiv, 87.1 µmol, 14.3 mg) in benzene (10.0 mL). Upon removal of the solvent *in vacuo*, the desired compound was obtained by flash column chromatography (pentane/CH_2Cl_2 4:1) followed by a second flash column chromatography over basic Al_2O_3 (pentane/CH_2Cl_2 4:1) as a colorless oil (35 mg, 0.13 mmol, 46%). **FT IR** (neat) ν (cm^{-1}): 3544, 3367, 2949, 2916, 2868, 1599, 1456, 1383, 1366, 12192, 1260, 1208, 1192, 1053, 1026, 999, 972, 946, 869, 846, 803, 755, 711, 693, 646. **^{1}H NMR** (300 MHz, $CDCl_3$, 299 K) δ (ppm) = 7.00 (s, 2H), 6.83 (s, 1H), 3.62 – 3.46 (m, 1H), 2.30 (s, 6H), 1.90 – 1.58 (m, 4H), 1.49 – 1.17 (m, 8H), 1.15 – 0.79 (m, 6H). **^{13}C NMR** (75 MHz, $CDCl_3$, 299 K) δ (ppm) = 151.4 (C), 138.0 (C), 127.8 (CH), 123.8 (CH), 73.1 (CH), 54.3 (CH), 45.3 (CH_2), 39.6 (C), 35.1 (CH_2), 31.6 (CH), 29.6 (CH_3), 26.5 (CH_2), 23.5 (CH_3), 22.2 (CH_3), 21.8 (CH_3). **HRMS** (ESI) *m/z* = 283.2032 calcd. for $C_{18}H_{28}NaO^+$ $[M+Na]^+$, found: 283.2050. **OR**.: $[\alpha]_D^{20}$ = −32.17 (c = 0.52 in $CHCl_3$).

(1*R*,2*S*,5*R*)-5-Methyl-2-(2-(naphthalen-1-yl)propan-2-yl)cyclohexan-1-ol (3l-6)

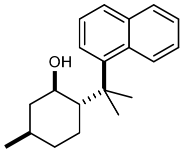

Alcohol **3l-6** was prepared following **GP5** using **2l-6** (1.0 equiv, 0.290 mmol, 137 mg), TTMSS (1.4 equiv, 0.406 mmol, 125 µL) and AIBN (0.3 equiv, 87.1 µmol, 14.3 mg) in benzene (10.0 mL). Upon removal of the solvent *in vacuo*, the desired compound was obtained by flash column chromatography over basic Al_2O_3 (pentane/CH_2Cl_2 1:1) as a

slightly yellow oil (48 mg, 0.17 mmol, 59%). **FT IR** (neat) ν (cm^{-1}): 3550, 3435, 3099, 3047, 2948, 2922, 1600, 1574, 1511, 1455, 1388, 1369, 1333, 1245, 1197, 1100, 1055, 1026, 1026, 1001, 966, 837, 803, 777, 736, 691. **^{1}H NMR** (400 MHz, $CDCl_3$, 299 K) δ (ppm) = 8.46 – 8.41 (m, 1H), 7.90 – 7.85 (m, 1H), 7.75 – 7.70 (m, 1H), 7.58 – 7.54 (m, 1H), 7.53 – 7.43 (m, 2H), 7.43 – 7.38 (m, 1H), 3.78 – 3.67 (m, 1H), 2.59 (ddd, *J* = 12.8 Hz, *J* = 9.6 Hz, *J* = 3.4 Hz, 1H), 1.85 (dtd, *J* = 12.4 Hz, *J* = 3.9 Hz, *J* = 2.3 Hz, 1H), 1.76 – 1.53 (m, 7H), 1.43 (tdq, *J* = 12.9 Hz, *J* = 6.6 Hz, *J* = 3.3 Hz, 1H), 1.32 – 1.08 (m, 2H), 0.97 – 0.80 (m, 6H). **^{13}C NMR** (101 MHz, $CDCl_3$, 299 K) δ (ppm) = 147.3 (C), 135.5 (C), 131.5 (C), 130.0 (CH), 128.0 (CH), 126.9 (CH), 125.4 (CH), 125.0 (CH), 124.9 (CH), 123.5 (CH), 73.4 (CH), 51.1 (CH), 45.4 (CH_2), 41.9 (C), 35.1 (CH_2), 31.6 (CH), 29.8 (CH_3), 28.1 (CH_3), 26.9 (CH_2), 22.1 (CH_3). **HRMS** (ESI) *m/z* = 305.1876 calcd. for $C_{20}H_{26}NaO^+$ $[M+Na]^+$, found: 305.1880. **OR.**: $[\alpha]_D^{20}$ = −34.5 (c = 2.4 in $CHCl_3$). The spectroscopic data are in accordance to those reported in the literature (*19*).

(1*R*,2*S*,5*R*)-2-(2-(4-Chloro-3-methylphenyl)propan-2-yl)-5-methylcyclohexan-1-ol (3l-7)

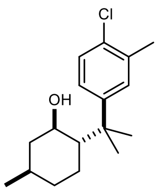

Alcohol **3l-7** was prepared following **GP5** using **2l-7** (1.0 equiv, 0.290 mmol, 137 mg), TTMSS (1.4 equiv, 0.406 mmol, 125 µL) and AIBN (0.3 equiv, 87.1 µmol, 14.3 mg) in benzene (10.0 mL). Upon removal of the solvent *in vacuo*, the desired compound was obtained by flash column chromatography over basic Al_2O_3 (pentane/CH_2Cl_2 4:1) as a colorless oil (59 mg, 0.21 mmol, 72%). **FT IR** (neat) ν (cm^{-1}): 3570, 3423, 2950, 2917, 2868, 1719, 1607, 1481, 1455, 1391, 1367, 1338, 1272, 1193, 1175, 1152, 1122, 1099, 1046, 1026, 1011, 965, 938, 886, 848, 818, 770, 714, 697, 657, 606, 566. **^{1}H NMR** (400 MHz, $CDCl_3$, 299 K) δ (ppm) = 7.30 – 7.24 (m, 1H), 7.23 – 7.21 (m, 1H), 7.14 (dd, *J* = 8.3 Hz, *J* = 2.4 Hz, 1H), 3.51 (ddd, *J* = 10.8 Hz, *J* = 9.6 Hz, *J* = 4.3 Hz, 1H), 2.36 (s, 3H), 1.85 (dtd, *J* = 12.3 Hz, *J* = 3.9 Hz, *J* = 2.2 Hz, 1H), 1.72 – 1.58 (m, 3H), 1.45 – 1.32 (m, 4H), 1.26 (s, 3H), 1.15 (brs, 1H), 1.08 – 0.78 (m, 6H). **^{13}C NMR** (101 MHz, $CDCl_3$, 299 K) δ (ppm) = 150.2 (C), 135.8 (C), 131.8 (C), 129.0 (CH), 128.6 (CH), 124.8 (CH), 73.2 (CH), 54.2 (CH), 45.6 (CH_2), 39.7 (C), 35.0 (CH_2), 31.7 (CH), 28.6 (CH_3), 26.6 (CH_2), 24.8 (CH_3), 22.1 (CH_3), 20.6 (CH_3). **HRMS** (ESI) *m/z* = 303.1486 calcd. for $C_{17}H_{25}ClNaO^+$ $[M+Na]^+$, found: 303.1496. **OR.**: $[\alpha]_D^{20}$ = −24.06 (c = 2.1 in $CHCl_3$).

(1*R*,2*S*,5*R*)-2-(2-(3,4-Dichlorophenyl)propan-2-yl)-5-methylcyclohexan-1-ol (3l-8)

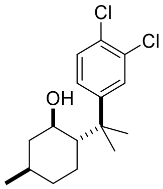

Alcohol **3l-8** was prepared following **GP5** using **2l-8** (1.0 equiv, 0.290 mmol, 143 mg), TTMSS (1.4 equiv, 0.406 mmol, 125 µL) and AIBN (0.3 equiv, 87.1 µmol, 14.3 mg) in benzene (10.0 mL). Upon removal of the solvent *in vacuo*, the desired compound was obtained by flash column chromatography over basic Al_2O_3 (pentane/CH_2Cl_2 4:1) as a colorless oil (45 mg, 0.15 mmol, 52%). **FT IR** (neat) ν (cm^{-1}): 3400, 2950, 2917, 2869, 1588, 1554, 1472, 1456, 1380, 1272, 1255, 1225, 1141, 1097, 1055, 1027, 1012, 999, 965, 879, 846, 820, 796, 767, 735, 711, 684, 661, 641, 613, 558. **^{1}H NMR** (300 MHz, $CDCl_3$, 299 K) δ (ppm) = 7.42 (d, *J* = 2.3 Hz, 1H), 7.35 (d, *J* = 8.5 Hz, 1H), 7.20 (dd, *J* = 8.5 Hz, *J* = 2.3 Hz, 1H), 3.48 (ddd, *J* = 10.8 Hz, *J* = 9.7 Hz, *J* = 4.2 Hz, 1H), 1.90 – 1.80 (m, 1H), 1.67 – 1.50 (m, 3H), 1.48 – 1.30 (m, 4H), 1.28 (s, 3H), 1.04 – 0.74 (m, 7H). **^{13}C NMR** (75 MHz, $CDCl_3$, 299 K) δ (ppm) = 152.3 (C), 132.2 (C), 130.0 (CH), 129.4 (C), 128.2 (CH), 125.7 (CH), 73.1 (CH), 54.2 (CH), 46.1 (CH_2), 40.2 (C), 34.8 (CH_2), 31.7 (CH), 27.6 (CH_3),

26.7 (CH_2), 26.0 (CH_3), 22.1 (CH_3). **HRMS** (ESI) *m/z* = 323.0940 calcd. for $C_{16}H_{22}Cl_2NaO^+$ $[M+Na]^+$, found: 323.0947. **OR.**: $[\alpha]_D^{20}$ = −23.14 ° (c = 2.3 in $CHCl_3$).

(1*R*,2*S*,5*R*)-5-Methyl-2-(2-(*p*-tolyl)propan-2-yl)cyclohexan-1-ol (3l-9)

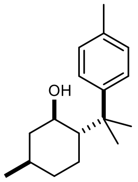

Alcohol **3l-9** was prepared following **GP5** using **2l-9** (1.0 equiv, 0.290 mmol, 127 mg), TTMSS (1.4 equiv, 0.406 mmol, 125 µL) and AIBN (0.3 equiv, 87.0 µmol, 14.3 mg) in benzene (10.0 mL). After allowing the reaction mixture to cool to rt, TBAF (1.0 M in THF, 2.0 equiv, 0.58 mmol, 0.58 mL) was added and stirring was continued for 2 hrs. Upon removal of the solvent *in vacuo*, the desired compound was obtained by flash column chromatography (pentane/Et_2O 9:1) as a colorless oil (36 mg, 0.15 mmol, 50%). **^{1}H NMR** (300 MHz, $CDCl_3$, 299 K) δ (ppm) = 7.33 – 7.27 (m, 2H), 7.19 – 7.09 (m, 2H), 3.60 – 3.46 (m, 1H), 2.31 (s, 3H), 1.84 (dtd, *J* = 12.5 Hz, *J* = 4.0 Hz, *J* = 2.3 Hz, 1H), 1.79 – 1.59 (m, 3H), 1.51 – 1.19 (m, 7H), 1.15 – 0.76 (m, 7H). **^{13}C NMR** (75 MHz, $CDCl_3$, 299 K) δ (ppm) = 148.4 (C), 135.5 (C), 129.3 (CH), 125.8 (CH), 73.1 (CH), 54.3 (CH), 45.4 (CH_2), 39.5, 35.1 (CH_2), 31.6 (CH), 29.3 (CH_3), 26.6 (CH_2), 24.0 (CH_3), 22.2 (CH_3), 21.0 (CH_3). **HRMS** (ESI) *m/z* = 269.1876 calcd. for $C_{17}H_{26}NaO^+$ $[M+Na]^+$, found: 269.1874. $[\alpha]_D^{20}$ = −32.6 (c = 1.7 in $CHCl_3$). The spectroscopic data are in accordance to those reported in the literature (*19*).

(1*R*,2*S*,5*R*)-5-Methyl-2-(2-(pyridin-2-yl)propan-2-yl)cyclohexan-1-ol) (3l-10)

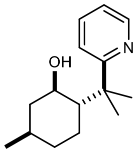

Alcohol **3l-10** was prepared following **GP5** using **2l-10** (1.0 equiv, 0.290 mmol, 123 mg), TTMSS (1.4 equiv, 0.406 mmol, 125 µL) and AIBN (0.3 equiv, 87.0 µmol, 14.3 mg) in benzene (10.0 mL). Upon removal of the solvent *in vacuo*, the desired compound was obtained by flash column chromatography (pentane/Et_2O 3:2) as a colorless oil (31 mg, 0.13 mmol, 46%). **^{1}H NMR** (300 MHz, $CDCl_3$, 299 K) δ (ppm) = 8.51 (ddd, *J* = 4.9 Hz, *J* = 2.0 Hz, *J* = 0.9 Hz, 1H), 7.63 (td, *J* = 7.8 Hz, *J* = 1.9 Hz, 1H), 7.34 (dt, *J* = 8.1 Hz, *J* = 1.1 Hz, 1H), 7.09 (ddd, *J* = 7.5 Hz, *J* = 4.9 Hz, *J* = 1.1 Hz, 1H), 3.44 (brs, 1H), 3.32 – 3.17 (m, 1H), 1.92 – 1.75 (m, 3H), 1.70 – 1.57 (m, 1H), 1.47 – 1.27 (m, 7H), 1.07 – 0.78 (m, 6H). **^{13}C NMR** (75 MHz, CDCl3) δ (ppm) = 169.7 (C), 147.9 (CH), 136.9 (CH), 120.9 (CH), 120.7 (CH), 73.3 (CH), 53.2 (CH), 45.1 (CH_2), 42.7 (C), 35.1 (CH_2), 31.8 (CH), 29.8 (CH_3), 26.5 (CH_2), 23.9 (CH_3), 22.1 (CH_3). **HRMS** (ESI) *m/z* = 234.1852 calcd. for $C_{15}H_{24}NO^+$ $[M+H]^+$, found: 234.1855. $[\alpha]_D^{20}$ = −22.7 (c = 1.0 in $CHCl_3$). The spectroscopic data are in accordance to those reported in the literature (*19*).

Cyclohexyl(2-phenylcyclohexyl)methanol (3m)

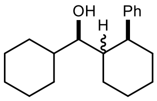

Alcohol **3m** was prepared following **GP5** using **2m** (1.0 equiv, 0.371 mmol, 172 mg), TTMSS (1.4 equiv, 0.519 mmol, 160 µL) and AIBN (0.3 equiv, 0.111 mmol, 18.3 mg) in benzene (12.8 mL). Two diastereomers were formed in a *dr* of 1:1 (determined by ^{1}H NMR spectroscopy of the crude product). Upon removal of the solvent *in vacuo*, the two observed diastereomers were isolated by flash column chromatography over basic Al_2O_3 (pentane/CH_2Cl_2 4:1) as a colorless liquid (44 mg, 0.162 mmol, 44%, *dr*: 1.5:1.0). **FT IR** (neat) ν (cm^{-1}): 3586, 3460, 3027, 2921, 2850, 1601, 1492, 1448, 1384, 1259, 1183, 1082, 1031, 907, 841, 756, 732, 699, 649. ^{1}**H NMR** (300 MHz, $CDCl_3$, 299 K) δ (ppm) = 7.36 – 7.15 (m, 13H, both isomers), 3.16 (t, *J* = 5.3 Hz, 1H, minor isomer), 3.10 (t, *J* = 4.1 Hz, 1H, major isomer), 2.96 (dt, *J* = 7.8 Hz, *J* = 4.3 Hz, 1H, minor isomer), 2.48 (td, *J* = 11.3 Hz, *J* = 3.3 Hz, 2H,

major isomer), 2.09 – 0.77 (m, 60H, both isomers). **^{13}C NMR** (151 MHz, $CDCl_3$, 299 K) δ (ppm) = 147.0 (C), 145.4 (C), 128.9 (CH), 128.8 (CH), 128.3 (CH), 127.7 (CH), 126.4 (CH), 126.0 (CH), 80.3 (CH), 77.2 (CH), 48.5 (CH), 46.5 (CH), 44.9 (CH), 41.5 (CH), 40.9 (CH), 40.1 (CH), 36.8 (CH_2), 30.8 (CH_2), 30.4 (CH_2), 30.2 (CH_2), 30.0 (CH_2), 27.4 (CH_2), 27.3 (CH_2), 26.8 (CH_2), 26.6 (CH_2), 26.6 (CH_2), 26.6 (CH_2), 26.6 (CH_2), 26.3 (CH_2), 26.3 (CH_2), 25.5 (CH_2), 24.8 (CH_2), 23.9 (CH_2). **HRMS** (ESI) *m/z* = 295.2032 calcd. for $C_{19}H_{28}NaO^+$ $[M+Na]^+$, found: 295.2036. The determination of the relative configuration was carried out in different experiments, see chapter 0.

2,2-Dimethyl-1-(2-phenylcyclohexyl)propan-1-ol (3n)

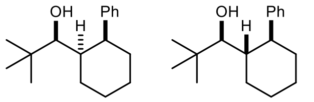

fraction I **(3m-a)** fraction II **(3m-b)**

Alcohol **3n** was prepared following **GP5** using **2n** (1.0 equiv, 0.481 mmol, 210 mg), TTMSS (1.4 equiv, 0.673 mmol, 207 µL) and AIBN (0.3 equiv, 0.144 mmol, 23.7 mg) in benzene (16.6 mL). Two diastereomers were formed in a *dr* of 1:1 (determined by ^{1}H NMR spectroscopy of the crude product). Upon removal of the solvent *in vacuo*, the two diastereomers were isolated by flash column chromatography over basic Al_2O_3 (pentane/CH_2Cl_2 9:1) as colorless liquids (**3n-a**: 23 mg, 93 µmol, 19%; **3n-b**: 25 mg, 0.10 mmol, 21%). **HRMS** (ESI) *m/z* = 269.1876 calcd. for $C_{17}H_{26}NaO^+$ $[M+Na]^+$, found: 269.1876. **3n-a**: **FT IR** (neat) ν (cm^{-1}): 3529, 3025, 2953, 2926, 2867, 1602, 1494, 1478, 1448, 1396, 1362, 1297, 1186, 1079, 1041, 1001, 963, 908, 888, 858, 843, 768, 732, 700, 647. **^{1}H NMR** (300 MHz, $CDCl_3$, 299 K) δ (ppm) = 7.39 – 7.26 (m, 4H), 7.24 – 7.15 (m, 1H), 3.17 (d, *J* = 1.7 Hz, 1H), 2.87 (dt, *J* = 8.0 Hz, *J* = 4.1 Hz, 1H), 2.13 – 2.05 (m, 1H), 2.03 – 1.59 (m, 6H), 1.58 – 1.41 (m, 2H), 1.27 (brs, 1H), 0.72 (s, 9H). **^{13}C NMR** (75 MHz, $CDCl_3$, 299 K) δ (ppm) = 145.5 (C), 129.4 (CH), 128.2 (CH), 126.1 (CH), 79.3 (CH), 47.6 (CH), 40.3 (CH), 36.2 (C), 30.1 (CH_2), 26.1 (CH_3), 25.9 (CH_2), 25.1 (CH_2), 24.3 (CH_2). **3n-b**.: **FT IR** (neat) ν (cm^{-1}): 3587, 3028, 2923, 2853, 1601, 1479, 1449, 1395, 1363, 1179, 1081, 1054, 1031, 994, 955, 908, 881, 848, 757, 731, 701, 647. **^{1}H NMR** (300 MHz, $CDCl_3$, 299 K) δ (ppm) = 7.33 – 7.22 (m, 4H), 7.21 – 7.12 (m, 1H), 3.07 (d, *J* = 4.6 Hz, 1H), 2.70 (td, *J* = 11.0 Hz, *J* = 3.6 Hz, 1H), 2.03 – 1.69 (m, 5H), 1.50 – 1.00 (m, 5H), 0.75 (s, 9H). **^{13}C NMR** (75 MHz, $CDCl_3$, 299 K) δ (ppm) = 148.0 (C), 128.7 (CH), 128.2 (CH), 126.2 (CH), 84.4 (CH), 47.6 (CH), 45.3 (CH), 38.2 (CH_2), 36.2 (C), 34.9 (CH_2), 26.8 (CH_3), 26.6 (CH_2). The determination of the relative configuration was carried out in different experiments, see chapter 0.

2,2-Dimethyl-5-phenylnonan-3-ol (3o)

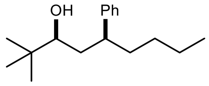

Alcohol **3o** was prepared following **GP5** using **2o** (1.0 equiv, 0.456 mmol, 200 mg), TTMSS (1.4 equiv, 0.638 mmol, 198 µL) and AIBN (0.3 equiv, 0.137 mmol, 22.5 mg) in benzene (15.7 mL). Two diastereomers were formed in a *dr* of 6:1 (determined by ^{1}H NMR spectroscopy of the crude product). Upon removal of the solvent *in vacuo*, the product was isolated by flash column chromatography over basic Al_2O_3 (pentane/CH_2Cl_2 1:1) as a colorless liquid (50 mg, 0.201 mmol, 44%, *dr*: 8:1). **FT IR** (neat) ν (cm^{-1}): 3474, 3026, 2955, 2928, 1602, 1494, 1479, 1467, 1453, 1364, 1241, 1175, 1066, 1008, 957, 899, 850, 760, 726, 699. **^{1}H NMR** (300 MHz, $CDCl_3$, 300 K, major isomer) δ (ppm) = 7.35 – 7.27 (m, 2H), 7.24 – 7.14 (m, 3H), 3.36 (dd, *J* = 10.0 Hz, *J* = 1.8 Hz, 1H), 2.79 – 2.65 (m, 1H), 1.86 (ddd, *J* = 14.2 Hz, *J* = 8.1 Hz, *J* = 1.8 Hz, 1H), 1.81 – 1.66 (m, 1H), 1.65 – 1.44 (m, 2H), 1.39 – 0.98 (m, 5H), 0.89 (s, 9H), 0.87 – 0.77 (m, 3H). **^{13}C NMR** (75 MHz, $CDCl_3$, 300 K, major isomer) δ

(ppm) = 146.5 (C), 128.5 (CH), 127.65 (CH), 126.15 (CH), 78.65 (CH), 43.85 (CH), 39.7 (CH_2), 35.5 (CH_2), 35.0 (C), 29.6 (CH_2), 25.7 (CH_3), 22.8 (CH_2), 14.0 (CH_3). **HRMS** (ESI) *m/z* = 271.2032 calcd. for $C_{17}H_{28}NaO^+$ [M+Na]$^+$, found: 271.2030.

2,2,6-Trimethyl-5-phenylheptan-3-ol (3p)

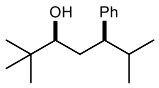

Alcohol **3p** was prepared following **GP5** using **2p** (1.0 equiv, 0.330 mmol, 140 mg), TTMSS (1.4 equiv, 0.462 mmol, 142 µL) and AIBN (0.3 equiv, 99.3 µmol, 16.3 mg) in benzene (15.7 mL). Two diastereomers were formed in a *dr* of 10:1 (determined by ^{1}H NMR spectroscopy of the crude product). Upon removal of the solvent *in vacuo*, the product was obtained by flash column chromatography over basic Al_2O_3 (pentane/CH_2Cl_2 9:1) followed by a second flash column chromatography (pentane/Et_2O 9:1) as a colorless solid (29 mg, 0.124 mmol, 43%, single isomer). Crystals suitable for X-ray diffraction analysis were obtained by slow evaporation of a solution **3o** in toluene, resulting in the assignment of the relative configuration of the compound (CCDC 1583763). For further information see chapter **Fehler! Verweisquelle konnte nicht gefunden werden.**. **MP**.: 55 °C. **FT IR** (neat) ν (cm^{-1}): 3586, 3469, 3026, 2956, 2931, 2869, 1301, 1494, 1479, 1466, 1452, 1365, 1178, 1069, 1032, 1010, 909, 759, 701. ^{1}**H NMR** (400 MHz, $CDCl_3$, 299 K) δ (ppm) = 7.33 – 7.27 (m, 2H), 7.23 – 7.17 (m, 3H), 3.28 (dd, *J* = 9.4 Hz, *J* = 1.9 Hz, 1H), 2.49 (dt, *J* = 8.2 Hz, *J* = 6.2 Hz, 1H), 2.13 (ddd, *J* = 14.4 Hz, *J* = 5.9 Hz, *J* = 1.9 Hz, 1H), 1.97 – 1.82 (m, 1H), 1.61 – 1.49 (m, 1H), 1.24 (brs, 1H), 0.97 – 0.86 (m, 12H), 0.72 (d, *J* = 6.7 Hz, 3H). ^{13}**C NMR** (101 MHz, $CDCl_3$, 299 K) δ (ppm) = 144.4 (C), 128.8 (CH), 128.3 (CH), 126.3 (CH), 79.9 (CH), 51.4 (CH), 35.8 (CH_2), 35.2 (C), 32.7 (CH), 25.7 (CH_3), 21.4 (CH_3), 19.4 (CH_3). **HRMS** (ESI) *m/z* = 257.1876 calcd. for $C_{16}H_{26}NaO^+$ [M+Na]$^+$, found: 257.1878.

1-Cyclohexyl-4,4-dimethyl-1-phenylpentan-3-ol (3q)

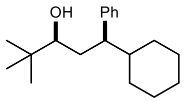

Alcohol **3q** was prepared following **GP5** using **2q** (1.0 equiv, 0.411 mmol, 191 mg), TTMSS (1.4 equiv, 0.575 mmol, 177 µL) and AIBN (0.3 equiv, 0.123 mmol, 20.3 mg) in benzene (14.2 mL). Two diastereomers were formed in a *dr* of 12:1 (determined by ^{1}H NMR spectroscopy of the crude product). Upon removal of the solvent *in vacuo*, the product was isolated by flash column chromatography over basic Al_2O_3 (pentane/CH_2Cl_2 4:1) followed by a second flash column chromatography (pentane/Et_2O 20:1) as a colorless oil (28 mg, 0.113 mmol, 28%, single isomer). **FT IR** (neat) ν (cm^{-1}): 3476, 3026, 3026, 2923, 2852, 1602, 1494, 1479, 1450, 1394, 1363, 1264, 1185, 1059, 1009, 945, 909, 945, 842, 759, 734, 701, 645. ^{1}**H NMR** (300 MHz, $CDCl_3$, 299 K) δ (ppm) = 7.33 – 7.26 (m, 2H), 7.23 – 7.14 (m, 3H), 3.26 (dd, *J* = 9.3 Hz, *J* = 1.9 Hz, 1H), 2.50 (dt, *J* = 8.3 Hz, *J* = 6.2 Hz, 1H), 2.16 (ddd, *J* = 14.4 Hz, *J* = 5.8 Hz, *J* = 1.9 Hz, 1H), 1.93 – 1.79 (m, 1H), 1.78 – 1.39 (m, 5H), 1.37 – 0.66 (m, 16H). ^{13}**C NMR** (75 MHz, $CDCl_3$, 299 K) δ (ppm) = 144.7 (C), 128.7 (CH), 128.3 (CH), 126.2 (CH), 80.0 (CH), 50.9 (CH), 42.8 (CH), 35.5 (CH_2), 35.2 (C), 31.8 (CH_2), 30.0 (CH_2), 26.7 (CH_2), 26.6 (CH_2), 26.5 (CH_2), 25.7 (CH_3). **HRMS** (ESI) *m/z* = 297.2189 calcd. for $C_{19}H_{30}NaO^+$ [M+Na]$^+$, found: 297.2183.

(1*R*,2*S*,5*R*)-5-Methyl-2-(*S*)-1-phenylethyl)cyclohexan-1-ol (3r-1)

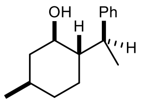

Alcohol **3r-1** was prepared following **GP5** using **2r-1** (1.0 equiv, 0.290 mmol, 118 mg), TTMSS (1.4 equiv, 0.406 mmol, 125 µL) and AIBN (0.3 equiv, 87.1 µmol, 14.3 mg) in benzene (10.0 mL). Only one diastereomer was observed in the crude ^{1}H NMR spectrum.

Upon removal of the solvent *in vacuo*, the desired product was isolated by flash column chromatography over basic Al_2O_3 (pentane/CH_2Cl_2 7:3) followed by a second flash column chromatography (pentane/CH_2Cl_2 1:1) as a colorless solid (23 mg, 0.105 mmol, 36%, *dr* > 20:1 by ^{1}H NMR spectroscopy). **MP**.:63-65 °C. **FT IR** (neat) ν (cm^{-1}): 3282, 3093, 3026, 2925, 2850, 1720, 1603, 1556, 1528, 1491, 1448, 1370, 1353, 1337, 1310, 1270, 1227, 1174, 1147, 1104, 1089, 1070, 1048, 1034, 1017, 991, 948, 902, 880, 847, 777, 752, 698, 684, 622, 601. ^{1}**H NMR** (300 MHz, $CDCl_3$, 299 K) δ (ppm) = 7.36 – 7.16 (m, 5H), 3.51 (ddd, *J* = 10.8 Hz, *J* = 9.7, *J* = 4.4 Hz, 1H), 3.34 (qd, *J* = 7.2 Hz, *J* = 3.7 Hz, 1H), 2.02 – 1.92 (m, 1H), 1.62 – 1.52 (m, 1H), 1.50 – 1.35 (m, 4H), 1.24 (d, *J* = 7.3 Hz, 3H), 1.15 – 0.93 (m, 2H), 0.89 (d, *J* = 6.6 Hz, 3H), 0.72 (tdd, *J* = 12.9 Hz, *J* = 11.6 Hz, *J* = 3.5 Hz, 1H). ^{13}**C NMR** (75 MHz, $CDCl_3$, 299 K) δ (ppm) = 146.6 (C), 128.3 (CH), 128.0 (CH), 125.9 (CH), 72.3 (CH), 51.4 (CH), 45.1 (CH_2), 38.4 (CH), 34.5 (CH_2), 31.8 (CH), 24.4 (CH_2), 22.3 (CH_3), 13.6 (CH_3). **HRMS** (ESI) *m/z* = 241.1563 calcd. for $C_{15}H_{22}NaO^+$ [M+Na]$^+$, found: 241.1560. **OR**.: $[\alpha]_D^{20}$ = −9.389 (c = 0.66 in $CHCl_3$).

(1*R*,2*S*,5*R*)-2-(*S*)-1-(4-Chloro-3-methylphenyl)ethyl)-5-methylcyclohexan-1-ol (3r-2)

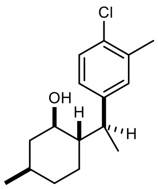

Alcohol **3r-2** was prepared following **GP5** using **2r-2** (1.0 equiv, 0.290 mmol, 132 mg), TTMSS (1.4 equiv, 0.406 mmol, 125 µL) and AIBN (0.3 equiv, 87.1 µmol, 14.3 mg) in benzene (10.0 mL). Only one diastereomer was observed in the crude ^{1}H NMR spectrum. Upon removal of the solvent *in vacuo*, the desired product was isolated by flash column chromatography over basic Al_2O_3 (pentane/CH_2Cl_2 1:1) followed by a second flash column chromatography (pentane/CH_2Cl_2 3:2) as a colorless oil (23.7 mg, 88.8 µmol, 31%, *dr* > 20:1 by ^{1}H NMR spectroscopy). **FT IR** (neat) ν (cm^{-1}): 3351, 2948, 2922, 2868, 1482, 1454, 1408, 1375, 1347, 1275, 1222, 1164, 1147, 1105, 1082, 1049, 1024, 988, 953, 909, 883, 847, 818, 771, 733, 659. ^{1}**H NMR** (300 MHz, $CDCl_3$, 299 K) δ (ppm) = 7.25 (d, *J* = 8.0 Hz, 1H), 7.09 (d, *J* = 2.2 Hz, 1H), 7.03 – 6.97 (m, 1H), 3.56 – 3.42 (m, 1H), 3.31 (qd, *J*=7.3 Hz, *J* = 3.5 Hz, 1H), 2.36 (s, 3H), 2.04 – 1.90 (m, 1H), 1.64 – 1.53 (m, 1H), 1.52 – 1.30 (m, 4H), 1.20 (d, *J* = 7.2 Hz, 3H), 1.14 – 0.84 (m, 5H), 0.81 – 0.62 (m, 1H). ^{13}**C NMR** (75 MHz, $CDCl_3$, 299 K) δ (ppm) = 145.2 (C), 135.6 (C), 131.7 (C), 130.7 (CH), 128.7 (CH), 126.7 (CH), 72.1 (CH), 51.4 (CH), 45.3 (CH_2), 37.7 (CH), 34.5 (CH_2), 31.8 (CH), 24.3 (CH_2), 22.2 (CH_3), 20.3 (CH_3), 13.5 (CH_3). **HRMS** (ESI) *m/z* = 289.1330 calcd. for $C_{16}H_{23}ClNaO^+$ [M+Na]$^+$, found: 289.1318. **OR**.: $[\alpha]_D^{20}$ = −1.88 ° (c = 1.2 in $CHCl_3$).

(1*R*,2*S*,5*R*)-2-(*S*)-1-(3,5-Dimethylphenyl)ethyl)-5-methylcyclohexan-1-ol (3r-3)

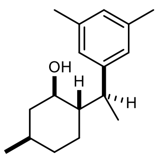

Alcohol **3r-3** was prepared following **GP5** using **2r-3** (1.0 equiv, 0.290 mmol, 127 mg), TTMSS (1.4 equiv, 0.406 mmol, 125 µL) and AIBN (0.3 equiv, 87.1 µmol, 14.3 mg) in benzene (10.0 mL). Only one diastereomer was observed in the crude ^{1}H NMR spectrum. Upon removal of the solvent *in vacuo*, the desired product was isolated by flash column chromatography over basic Al_2O_3 (pentane/CH_2Cl_2 1:1) followed by a second flash column chromatography (pentane/Et_2O 9:1) as a colorless oil (23.0 mg, 93.3 µmol, 32%, *dr* > 20:1 by ^{1}H NMR spectroscopy). **FT IR** (neat) ν cm^{-1}:3362, 2947, 2919, 2868, 1711, 1602, 1454, 1375, 1330, 1272, 1220, 1173, 1145, 1084, 1032, 1016, 989, 954, 883, 850, 771, 711, 650. ^{1}**H NMR** (300 MHz, $CDCl_3$, 299 K) δ (ppm) = 6.87 – 6.81 (m, 3H), 3.49 (td, *J* = 10.3 Hz, *J* = 4.4 Hz, 1H), 3.21 (qd, *J* = 7.2 Hz, *J* = 4.1 Hz, 1H), 2.30 (s, 6H), 2.01 – 1.90 (m,

1H), 1.64 – 1.34 (m, 5H), 1.20 (d, *J* = 7.2 Hz, 3H), 1.16 – 0.85 (m, 5H), 0.74 (qd, *J* = 12.6 Hz, *J* = 3.4 Hz, 1H). **^{13}C NMR** (75 MHz, $CDCl_3$, 299 K) δ (ppm) = 146.7 (C), 137.7 (C), 127.7 (CH), 125.8 (CH), 72.6 (CH), 51.4 (CH), 45.1 (CH_2), 38.7 (CH), 34.6 (CH_2), 31.8 (CH), 24.9 (CH_2), 22.3 (CH_3), 21.5 (CH_3), 14.2 (CH_3). **HRMS** (ESI) *m/z* = 269.1876 calcd. for $C_{17}H_{26}NaO^+$ $[M+Na]^+$, found: 269.1861. **OR**.: $[\alpha]_D^{20} = -1.88$ ° (c = 1.2 in $CHCl_3$).

1,1,1-Trichloro-4-methyl-4-phenylpentan-2-ol (3s)

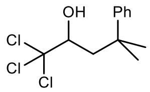

Alcohol **3s** was prepared following **GP5** using **2s** (1.0 equiv, 0.293 mmol, 138 mg), TTMSS (1.4 equiv, 0.410 mmol, 126 µL) and AIBN (0.3 equiv, 88.3 µmol, 14.5 mg) in benzene (10.1 mL). Upon removal of the solvent *in vacuo*, the desired compound was obtained by flash column chromatography (pentane/CH_2Cl_2 4:1) as a colorless liquid (31 mg, 0.11 mmol, 38%). **FT IR** (neat) ν (cm^{-1}): 3482, 3061, 2964, 2929, 1709, 1602, 1497, 1444, 1390, 1369, 1294, 1259, 1200, 1154, 1107, 1082, 1031, 1010, 850, 809, 780, 762, 698, 637. **^{1}H NMR** (300 MHz, $CDCl_3$, 299 K) δ (ppm) = 7.45 – 7.38 (m, 2H), 7.38 – 7.31 (m, 2H), 7.25 – 7.18 (m, 1H), 3.82 (dd, *J* = 8.6 Hz, *J* = 1.2 Hz, 1H), 2.43 (dd, *J* = 14.6 Hz, *J* = 1.2 Hz, 1H), 2.16 (brs, 1H), 1.95 (dd, *J* = 14.6 Hz, *J* = 8.6 Hz, 1H), 1.49 (s, 3H), 1.44 (d, *J* = 1.2 Hz, 3H). **^{13}C NMR** (101 MHz, $CDCl_3$, 299 K) δ (ppm) = 147.8 (C), 128.4 (CH), 126.2 (CH), 126.0 (CH), 80.8 (CH), 77.2 (C), 45.7 (CH_2), 37.2 (C), 30.2 (CH_3), 28.6 (CH_3). **HRMS** (ESI) *m/z* = 303.0081 calcd. for $C_{12}H_{15}Cl_3NaO^+$ $[M+Na]^+$, found: 303.0071.

1,1,1-Trichloro-3-(1-phenylcyclopentyl)propan-2-ol (3t)

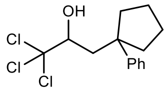

Alcohol **3t** was prepared following **GP5** using **2t** (1.0 equiv, 0.290 mmol, 144 mg), TTMSS (1.4 equiv, 0.406 mmol, 125 µL) and AIBN (0.3 equiv, 87.1 µmol, 14.3 mg) in benzene (10.0 mL). After cooling to rt, TBAF was added (1.0 M solution in THF, 2.0 equiv, 0.58 mL) and stirring was continued overnight. Upon removal of the solvent *in vacuo*, the desired compound was obtained by flash column chromatography (pentane/Et_2O 20:1) as a colorless liquid (31 mg, 0.10 mmol, 35%). **FT IR** (neat) ν (cm^{-1}): 3488, 3029, 2957, 2872, 1601, 1495, 1446, 1386, 1294, 1256, 1083, 1032, 908, 853, 8205, 761, 731, 700, 649, 624, 588. **^{1}H NMR** (300 MHz, $CDCl_3$, 299 K) δ (ppm) = 7.39 – 7.29 (m, 4H), 7.25 – 7.17 (m, 1H), 3.78 – 3.66 (m, 1H), 2.42 (dd, *J* = 14.5 Hz, *J* = 1.2 Hz, 1H), 2.30 (brs, 1H), 2.20 – 1.50 (m, 9H). **^{13}C NMR** (101 MHz, $CDCl_3$, 299 K) δ (ppm) = 146.8 (C), 128.4 (CH), 126.9 (CH), 126.2 (CH), 104.3 (C), 81.1 (CH), 50.0 (C), 43.2 (CH_2), 39.2 (CH_2), 37.1 (CH_2), 23.1 (CH_2), 22.5 (CH_2). **HRMS** (ESI) *m/z* = 329.0237 calcd. for $C_{14}H_{17}Cl_3NaO^+$ $[M+Na]^+$, found: 329.0237.

1,1,1-Trichloro-4-phenylheptan-2-ol (3u)

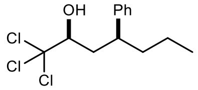

Alcohol **3u** was prepared following **GP5** using **2u** (1.0 equiv, 0.290 mmol, 141 mg), TTMSS (1.4 equiv, 0.406 mmol, 125 µL) and AIBN (0.3 equiv, 87.1 µmol, 14.3 mg) in benzene (10.0 mL). After cooling to rt, TBAF was added (1.0 M solution in THF, 2.0 equiv, 0.58 mL) and stirring was continued overnight. Two diastereomers were formed in a *dr* of 9:1 (determined by ^{1}H NMR spectroscopy of the crude product). Upon removal of the solvent *in vacuo*, the desired compound was obtained by flash column chromatography (pentane/Et_2O 19:1) as a colorless liquid (30 mg, 0.102 mmol, 35%, *dr*: 5:1). **FT IR** (neat) ν (cm^{-1}): 3445, 3028, 2958, 2931, 2872, 1711, 1602, 1495, 1453, 1380, 1301, 1251, 1183, 1074, 1030, 1006, 908, 792, 757, 732, 699, 644, 557. **^{1}H NMR** (300 MHz,

$CDCl_3$, 299 K) δ (ppm) = 7.37 – 7.29 (m, 2H), 7.28 – 7.19 (m, 3H), 4.16 (d, *J* = 9.3 Hz, 1H), 2.91 (ddt, *J* = 14.5 Hz, *J* = 9.8 Hz, *J* = 5.0 Hz, 1H), 2.55 (s, 1H), 2.38 (ddd, *J* = 14.3 Hz, *J* = 8.8 Hz, *J* = 2.1 Hz, 1H), 1.93 (ddd, *J* = 14.5 Hz, *J* = 9.2 Hz, *J* = 5.6 Hz, 1H), 1.82 – 1.52 (m, 2H), 1.19 (q, *J* = 7.5 Hz, 2H), 0.88 (t, *J* = 7.3 Hz, 3H). **^{13}C NMR** (75 MHz, $CDCl_3$, 299 K) δ (ppm) = 145.2 (C), 128.7 (CH), 127.8 (CH), 126.6 (CH), 104.4 (C), 81.8 (CH), 42.6 (CH), 39.4 (CH_2), 37.8 (CH_2), 20.6 (CH_2), 14.2 (CH_3). **HRMS** (ESI) *m/z* = 317.0237 calcd. for $C_{13}H_{17}Cl_3NaO^+$ $[M+Na]^+$, found: 317.0236.

Methyl 3-hydroxy-2,2,5-trimethyl-5-phenylhexanoate (3v)

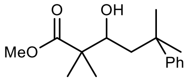

Alcohol **3v** was prepared following **GP5** using **2v** (1.0 equiv, 0.222 mmol, 101 mg), TTMSS (1.4 equiv, 0.31 mmol, 96 µL) and AIBN (0.3 equiv, 66.7 µmol, 11.0 mg) in benzene (7.7 mL). Upon absorbing the crude product on silica containing 10% KF, the compound was obtained by flash column chromatography (pentane/Et_2O 4:1) as a colorless oil (33 mg, 0.13 mmol, 56%). **FT IR** (neat) ν (cm^{-1}): 3532, 2961, 1718, 1497, 1471, 1388, 1367, 1311, 1267, 1193, 1148, 1131, 1104, 1080, 1070, 1032, 996, 908, 865, 765, 728, 700, 648. **^{1}H NMR** (300 MHz, $CDCl_3$, 299 K) δ (ppm) = 7.42 – 7.28 (m, 4H), 7.23 – 7.13 (m, 1H), 3.64 (s, 3H), 3.57 (dd, *J* = 8.1 Hz, *J* = 2.1 Hz, 1H), 1.84 – 1.58 (m, 3H), 1.44 – 1.37 (m, 6H), 1.10 – 1.03 (m, 6H). **^{13}C NMR** (75 MHz, $CDCl_3$, 299 K) δ (ppm) = 178.0 (C), 148.8 (C), 128.4 (CH), 126.1 (CH), 126.0 (CH), 74.0 (CH), 51.9 (CH_3), 47.7 (C), 47.1 (CH_2), 37.3 (C), 29.4 (CH_3), 29.2 (CH_3), 21.6 (CH_3), 20.6 (CH_3). **HRMS** (ESI) *m/z* = 287.1618 calcd. for $C_{16}H_{24}NaO_3^+$ $[M+Na]^+$, found: 287.1621.

Methyl 2,2,5-trimethyl-3-((phenylsulfonyl)oxy)hexanoate (S-25)

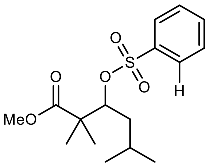

Sulfonate **S-25** was received as a side product of the aryl migration of **3v** following **GP5** using **3v** (1.0 equiv, 0.222 mmol, 101 mg), TTMSS (1.4 equiv, 0.31 mmol, 96 µL) and AIBN (0.3 equiv, 66.7 µmol, 11.0 mg) in benzene (7.7 mL). Upon absorbing the crude product on silica containing 10% KF, the compound was obtained by flash column chromatography (pentane/Et_2O 4:1) as a colorless oil (15 mg, 46 µmol, 21%). **FT IR** (neat) ν (cm^{-1}): 2957, 2872, 1737, 1468, 1449, 1391, 1361, 1346, 1290, 1263, 1185, 1145, 1096, 1056, 1017, 1000, 918, 886, 868, 840, 804, 778, 750, 731, 688, 670, 598. **^{1}H NMR** (300 MHz, $CDCl_3$, 299 K) δ (ppm) = 7.95 – 7.85 (m, 2H), 7.68 – 7.56 (m, 1H), 7.59 – 7.46 (m, 2H), 5.12 (dd, *J* = 9.4 Hz, *J* = 2.0 Hz, 1H), 3.62 (s, 3H), 1.71 – 1.47 (m, 2H), 1.28 – 1.15 (m, 4H), 1.12 (s, 3H), 0.89 (d, *J* = 6.4 Hz, 3H), 0.81 (d, *J* = 6.6 Hz, 3H). **^{13}C NMR** (75 MHz, $CDCl_3$, 299 K) δ (ppm) = 175.7 (C), 138.2 (C), 133.5 (CH), 129.1 (CH), 127.5 (CH), 86.3 (CH), 52.2 (CH_3), 47.5 (C), 40.9 (CH_2), 24.3 (CH), 23.8 (CH_3), 22.9 (CH_3), 21.3 (CH_3), 19.7 (CH_3). **HRMS** (ESI) *m/z* = 351.1237 calcd. for $C_{16}H_{24}NaO_5S^+$ $[M+Na]^+$, found: 351.1235.

rac **Methyl (3*S*,5*S*)-3-hydroxy-2,2-dimethyl-5-phenyloctanoate (3w)**

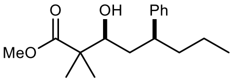

Alcohol **3w** was prepared following **GP5** using **2w** (1.0 equiv, 0.367 mmol, 172 mg), TTMSS (1.4 equiv, 0.514 mmol, 158 µL) and AIBN (0.3 equiv, 0.110 mmol, 18.1 mg) in benzene (12.7 mL). Two diastereomers were formed in a *dr* of 7:1 (determined by ^{1}H NMR spectroscopy of the crude product). Upon absorbing the crude product on silica containing 10% KF, the compound was obtained by flash column chromatography (pentane/Et_2O 9:1) as a colorless oil (69 mg, 0.25 mmol, 67%, *dr*: 6:1). **FT IR** (neat) ν (cm^{-1}):3491, 3028, 2955, 2932, 2872,

2364, 2337, 1721, 1467, 1389,1267, 1190 1136, 1067, 1028, 993, 906, 865, 762,732,700. **^{1}H NMR** (400 MHz, $CDCl_3$, 299 K, major isomer) δ (ppm) = 7.32 – 7.26 (m, 2H), 7.22 – 7.13 (m, 3H), 3.81 (dd, *J* = 10.3 Hz, *J* = 1.9 Hz, 1H), 3.68 (s, 3H), 2.86 – 2.76 (m, 1H), 2.16 (s, 1H), 1.77 – 1.66 (m, 2H), 1.61 – 1.43 (m, 2H), 1.22 – 1.04 (m, 8H), 0.84 (t, *J* = 7.3 Hz, 3H). **^{13}C NMR** (101 MHz, $CDCl_3$, 299 K, major isomer) δ (ppm) = 178.2 (C), 146.4 (C), 128.6 (CH), 127.7 (CH), 126.2 (CH), 75.3 (CH), 52.0 (CH_3), 47.4 (C), 42.8 (CH), 39.9 (CH_2), 37.6 (CH_2), 22.2 (CH_3), 20.7 (CH_3), 20.6 (CH_2), 14.3 (CH_3). **HRMS** (ESI) *m/z* = 301.1774 calcd. for $C_{17}H_{26}NaO_3^+$ [M+Na]$^+$, found: 301.1779.

***rac* Methyl (2*R*,3*S*)-3-hydroxy-2-methoxy-2,5-dimethyl-5-phenylhexanoate (3x)**

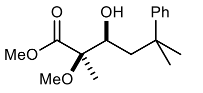

Alcohol **3x** was prepared following **GP5** using **2x** (1.0 equiv, 0.345 mmol, 162 mg), TTMSS (1.4 equiv, 0.483 mmol, 149 µL) and AIBN (0.3 equiv, 0.105 mmol, 17.0 mg) in benzene (11.9 mL). Two diastereomers were formed in a *dr* of 7:1 (determined by ^{1}H NMR spectroscopy of the crude product). Upon absorbing the crude product on silica containing 10% KF, the compound was obtained by flash column chromatography (pentane/Et_2O 9:1 → 4:1) as a colorless oil (61 mg, 0.22 mmol, 63%, *dr*: 7:1). **FT IR** (neat) ν (cm^{-1}): 3515, 2955, 1736, 1497, 144, 1259, 1180, 112, 1081, 1054, 1031, 980, 910, 841, 765, 730, 700. **^{1}H NMR** (400 MHz, $CDCl_3$, 299 K, major isomer) δ (ppm) = 7.40 – 7.34 (m, 2H), 7.33 – 7.27 (m, 2H), 7.20 – 7.14 (m, 1H), 3.68 (s, 3H), 3.62 – 3.58 (m, 1H), 3.19 (s, 3H), 1.91 (brs, 1H), 1.86 (dd, *J* = 14.7 Hz, *J* = 1.2 Hz, 1H), 1.76 – 1.65 (m, 1H), 1.44 – 1.39 (m, 3H), 1.38 – 1.34 (m, 3H), 1.29 – 1.25 (m, 3H). **^{13}C NMR** (101 MHz, $CDCl_3$, 299 K, major isomer) δ (ppm) = 173.7 (C), 149.0 (C), 128.3 (CH), 126.2 (CH), 125.9 (CH), 83.0 (C), 73.2 (CH), 52.4 (CH_3), 52.1 (CH_3), 45.9 (CH_2), 37.2 (C), 29.9 (CH_3), 28.8 (CH_3), 16.4 (CH_3). **HRMS** (ESI) *m/z* = 303.1567 calcd. for $C_{16}H_{24}NaO_4^+$ [M+Na]$^+$, found: 303.1576.

***rac* Methyl (2*R*,3*S*)-3-hydroxy-2,5-dimethyl-5-phenylhexanoate (3y)**

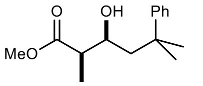

Alcohol **3y** was prepared following **GP5** using **2y** (1.0 equiv, 0.302 mmol, 133 mg), TTMSS (1.4 equiv, 0.423 mmol, 130 µL) and AIBN (0.3 equiv, 90.6 µmol, 14.9 mg) in benzene (10.4 mL). Upon absorbing the crude product on silica containing 10% KF, the compound was obtained by flash column chromatography (pentane/Et_2O 9:1) as a colorless oil (24 mg, 0.096 mmol, 32%, *dr*: 2:1). **FT IR** (neat) ν (cm^{-1}): 3503, 2959, 1723, 1601, 1497, 1461 1367, 1345, 1262, 1200, 1169, 1103, 1032, 990, 909, 840, 765, 730. **^{1}H NMR** (400 MHz, $CDCl_3$, 299 K, major isomer) δ (ppm) = 7.40 – 7.28 (m, 4H), 7.23 – 7.15 (m, 1H), 3.78 (ddd, *J* = 8.5 Hz, *J* = 4.1 Hz, *J* = 2.2 Hz, 1H), 3.67 – 3.64 (m, 3H), 2.43 – 2.28 (m, 1H), 1.94 (s, 1H), 1.86 – 1.67 (m, 2H), 1.42 (s, 3H), 1.38 (s, 3H), 1.11 – 1.06 (m, 3H). **^{13}C NMR** (101 MHz, $CDCl_3$, 299 K, major isomer) δ (ppm) = 176.3 (C), 148.8 (C), 128.5 (CH), 126.1 (CH), 126.0 (CH), 69.7 (CH), 51.8 (CH_3), 48.8 (CH_2), 45.8 (CH), 37.4 (C), 29.7 (CH_3), 29.2 (CH_3), 11.3 (CH_3). **HRMS** (ESI) *m/z* = 273.1461 calcd. for $C_{15}H_{22}NaO_3^+$ [M+Na]$^+$, found: 273.1473.

Ethyl 2,2-difluoro-3-hydroxy-5-methyl-5-phenylhexanoate (3z)

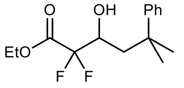

Alcohol **3z** was prepared following **GP5** using **2z** (1.0 equiv, 0.309 mmol, 147 mg), TTMSS (1.4 equiv, 0.432 mmol, 133 µL) and AIBN (0.3 equiv, 92.6 µmol, 15.2 mg) in benzene (10.6 mL). Upon absorbing the crude product on silica containing 10% KF, the compound was obtained by flash column chromatography (pentane/Et_2O 19:1) as a colorless oil (38 mg, 0.13 mmol, 43%).

FT IR (neat) ν (cm^{-1}): 3503, 2963, 2941, 2876, 1758, 1602, 1497, 1471, 1445, 1372, 1321, 1298, 1185, 1065, 1031, 855, 791, 765, 744, 700, 655. **^{1}H NMR** (400 MHz, $CDCl_3$, 299 K) δ (ppm) = 7.41 – 7.31 (m, 4H), 7.24 – 7.19 (m, 1H), 4.28 (q, *J* = 7.1 Hz, 2H), 3.94 (dddd, *J* = 15.6 Hz, *J* = 9.5 Hz, *J* = 7.9 Hz, *J* = 1.6 Hz, 1H), 2.09 – 2.03 (m, 1H), 1.92 (dd, *J* = 14.9 Hz, *J* = 9.6 Hz, 1H), 1.61 (brs, 1H), 1.45 (s, 3H), 1.42 (s, 3H), 1.30 (t, *J* = 7.2 Hz, 3H). **^{13}C NMR** (101 MHz, $CDCl_3$, 299 K) δ (ppm) = 163.7 (dd, *J* = 32.6 Hz, *J* = 31.3 Hz, C), 148.0 (C), 128.7 (CH), 126.4 (CH), 126.0 (CH), 114.7 (dd, *J* = 257.7 Hz, *J* = 253.4 Hz, C), 70.1 (dd, *J* = 27.7 Hz, *J* = 24.4 Hz, CH), 63.1 (CH_2), 44.2 – 40.6 (m, CH_2), 36.9 (C), 29.7 (CH_3), 29.3 (CH_3), 14.0 (CH_3). **^{19}F{^{1}H} NMR** (282 MHz, $CDCl_3$, 299 K) δ (ppm) = –114.66 (d, *J* = 261.8 Hz, 1F), –123.08 (d, *J* = 261.8 Hz, 1F). **HRMS** (ESI) *m/z* = 309.1273 calcd. for $C_{15}H_{20}F_2NaO_3^+$ [M+Na]$^+$, found: 309.1279.

***rac* Ethyl (3*S*,5*S*)-2,2-difluoro-3-hydroxy-5-phenyloctanoate(3aa)**

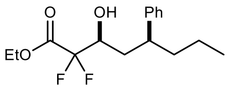

Alcohol **3aa** was prepared following **GP5** using **2aa** (1.0 equiv, 0.341 mmol, 167 mg), TTMSS (1.4 equiv, 0.477 mmol, 147 µL) and AIBN (0.3 equiv, 0.102 mmol, 16.8 mg) in benzene (11.8 mL). Two diastereomers were formed in a *dr* of 9:1 (determined by ^{19}F{^{1}H} NMR spectroscopy of the crude product). Upon absorbing the crude product on silica containing 10% KF, the compound was obtained by flash column chromatography (pentane/CH_2Cl_2 7:3) as a colorless oil (31 mg, 0.10 mmol, 30%, *dr*: 11:1). **FT IR** (neat) ν (cm^{-1}): 3434, 2957, 2932, 2874, 1759, 1454, 1375, 1315, 1210, 1186, 1097, 1068, 1020, 909, 857, 764, 731, 701. **^{1}H NMR** (300 MHz, $CDCl_3$, 299 K, major isomer) δ (ppm) = 7.37 – 7.25 (m, 2H), 7.25 – 7.14 (m, 3H), 4.40 – 4.26 (m, 2H), 4.12 (dddd, *J* = 16.0 Hz, *J* = 9.8 Hz, *J* = 6.8 Hz, *J* = 3.4 Hz, 1H), 2.88 – 2.72 (m, 1H), 2.06 (dddd, *J* = 14.4 Hz, *J* = 7.4 Hz, *J* = 3.4 Hz, *J* = 1.2 Hz, 1H), 1.92 – 1.49 (m, 4H), 1.39 – 1.29 (m, 3H), 1.22 – 1.09 (m, 2H), 0.85 (t, *J* = 7.3 Hz, 3H). **^{13}C NMR** (75 MHz, $CDCl_3$, 299 K, major isomer) δ (ppm) = 163.8 (dd, *J* = 32.9 Hz, *J* = 31.0 Hz, C), 145.1 (C), 128.8 (CH), 127.7 (CH), 126.7 (CH), 114.8 (dd, *J* = 257.9 Hz, *J* = 253.6 Hz, C), 70.9 (dd, *J* = 27.9 Hz, *J* = 24.9 Hz, CH), 63.1 (CH_2), 42.2 (CH), 38.3 (CH_2), 36.8 (CH_2), 20.5 (CH_2), 14.1 (CH_3), 14.1 (CH_3). **^{19}F{^{1}H} NMR** (282 MHz, $CDCl_3$, 299 K, major isomer) δ (ppm) = –113.81 (d, *J* = 263.7 Hz, 1F), –123.31 (d, *J* = 263.6 Hz, 1F). **HRMS** (ESI) *m/z* = 323.1429 calcd. for $C_{16}H_{26}F_2NaO_3^+$ [M+Na]$^+$, found: 323.1424.

***rac* Methyl ((3*S*,5*S*)-3-hydroxy-2,2-dimethyl-5-phenyloctanoyl)glycinate (3ab)**

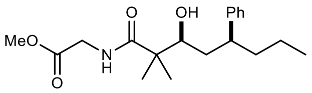

Alcohol **3ab** was prepared following **GP5** using **2ab** (1.0 equiv, 0.251 mmol, 132 mg), TTMSS (1.4 equiv, 0.352 mmol, 108 µL) and AIBN (0.3 equiv, 75.4 µmol, 12.4 mg) in benzene (8.7 mL). Two diastereomers were formed in a *dr* of 7:1 (determined by GC analysis of the crude product). Upon absorbing the crude product on silica containing 10% KF, the compound was obtained by flash column chromatography (pentane/EtOAc 4:1 → 7:3) as a colorless oil (23 mg, 0.069 mmol, 27%, *dr*: 6:1). A second fraction of a 1.0:1.0 mixture of the product and hydrolyzed starting material was collected containing 4.5 mg of the desired product (0.014 mmol, 5%). The overall yield was then calculated to 27.5 mg (0.082 mmol, 33%). **FT IR** (neat) ν (cm^{-1}): 3353, 2956, 2874, 2249, 1748, 1647, 1526, 1438, 1367, 1211, 1179, 1110, 1066, 1025, 986, 907, 763, 728, 701. **^{1}H NMR** (400 MHz, $CDCl_3$, 299 K, major isomer) δ (ppm) = 7.32 – 7.25 (m, 2H), 7.23 – 7.14 (m, 3H), 6.70 (t, *J* = 4.9 Hz, 1H), 4.08 – 3.91 (m, 2H), 3.79 – 3.64 (m, 4H), 2.83 – 2.72 (m, 1H), 2.43 (brs, 1H), 1.87 – 1.74

(m, 1H), 1.74 – 1.43 (m, 3H), 1.28 – 1.05 (m, 8H), 0.83 (t, J = 7.3 Hz, 3H). **^{13}C NMR** (126 MHz, $CDCl_3$, 299 K, major isomer) δ (ppm) = 178.0 (C), 170.8 (C), 146.2 (C), 128.7 (CH), 127.8 (CH), 126.4 (CH), 76.6 (CH), 52.4 (CH_3), 46.4 (C), 43.3 (CH), 41.3 (CH_2), 39.6 (CH_2), 37.8 (CH_2), 24.0 (CH_3), 21.1 (CH_3), 20.6 (CH_2), 14.2 (CH_3). **HRMS** (ESI) *m/z* = 358.1989 calcd. for $C_{19}H_{29}NNaO_4^+$ $[M+Na]^+$, found: 358.1995.

Determination of Stereochemistry of 3m and 3n: The determination of the relative configuration of the products was determined in two steps. First, oxidation of both isolated isomers of **3n** led to the formation of two different products. Thus, the stereocenter 1 is fixed. Compound **3n-a** was then analyzed by X-ray diffraction revealing its *cis* configuration.

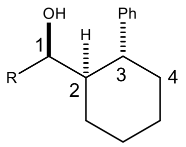

The *trans* configuration of **3n-b** was assigned of an homodecoupling ^{1}H NMR experiment. By irradiating the proton in position 1, the vicinal coupling of the proton in position 3 was analyzed. Its ^3J coupling to the protons in position 2 and 4 shows two large coupling constants (~11 Hz) to two axial protons in position 2 and 4 plus one small coupling constant (~3.8 Hz) to the second equatorial proton in position 4. Therefore, **3n-b** was assigned to be the *trans* isomer. These assignments are in agreement with the computational results, see chapter 0. Oxidation of **3m** to the corresponding ketones **S-20** also resulted in the formation of two different diastereomers. Therefore, we assume that the same assignments of the stereocenters as in **3n** applies as well.

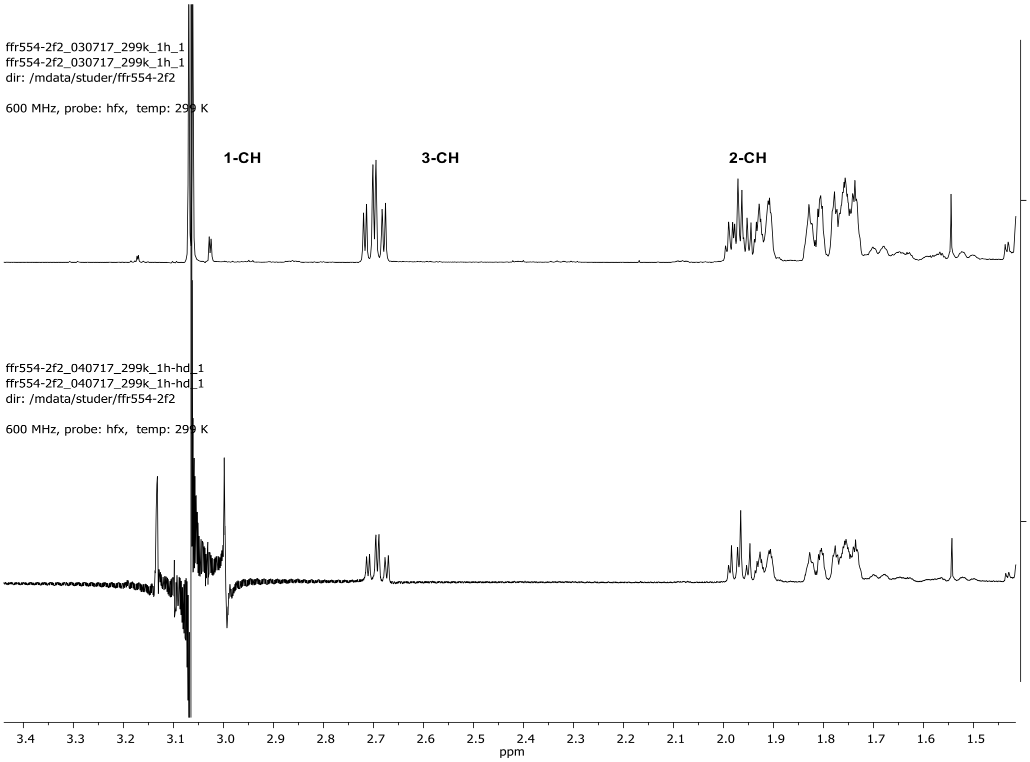

Supplementary Figure 1 ^{1}H NMR (600 MHz, $CDCl_3$, 299 K) of **3n-b**.

***rac*-2,2-Dimethyl-1-((1S,2R)-2-phenylcyclohexyl)propan-1-one (S-26)**

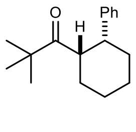

Compound **3n-a** (1.0 equiv, 93 µmol 23 mg) was oxidized by the addition of Dess–Martin periodinane (DMP, 2.0 equiv, 0.187 mmol, 79 mg) in anhydrous CH_2Cl_2 (2.0 mL) at rt. After 2 hrs, the mixture was absorbed on silica and the ketone was obtained by flash column

chromatography (pentane/Et_2O 99:1) as a colorless solid (21 mg, 86 µmol, 92%). Crystals suitable for X-ray diffraction analysis were obtained by slow evaporation of a solution of **S-26** in toluene resulting in the assignment of the relative configuration of the compound (CCDC 1583764). For further information see chapter **Fehler! Verweisquelle konnte nicht gefunden werden.**. **MP**.: 71 °C. **FT IR** (neat) ν (cm^{-1}): 3027, 2966, 2930, 2861, 1696, 1603, 1494, 1477, 1446, 1365, 1279, 1257, 1232, 1078, 1045, 1008, 973, 886, 857, 772, 747, 698, 625. ^{1}**H NMR** (300 MHz, $CDCl_3$, 299 K) δ (ppm) = 7.26 – 7.16 (m, 2H), 7.18 – 7.10 (m, 3H), 3.51 – 3.43 (m, 1H), 2.84 – 2.62 (m, 2H), 2.05 – 1.92 (m, 1H), 1.86 – 1.30 (m, 6H), 0.69 (s, 9H). ^{13}**C NMR** (75 MHz, $CDCl_3$, 299 K) δ (ppm) = 218.0 (C), 145.4 (C), 128.3 (CH), 128.2 (CH), 126.4 (CH), 47.0 (CH), 45.3 (CH), 45.2 (C), 29.8 (CH_2), 26.6 (CH_2), 26.4 (CH_2), 25.9 (CH_3), 20.5 (CH_2). **HRMS** (ESI) *m/z* = 267.1719 calcd. for $C_{17}H_{24}NaO^+$ $[M+Na]^+$, found: 267.1716.

***rac*-2,2-Dimethyl-1-((1*R*,2*R*)-2-phenylcyclohexyl)propan-1-one (S-27)**

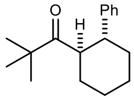

Compound **3n-b** (1.0 equiv, 0.102 mmol, 25 mg) was oxidized by the addition of DMP (2.0 equiv, 0.204 mmol, 86 mg) in anhydrous CH_2Cl_2 (2.0 mL) at rt. After 2 hrs, the mixture was absorbed on silica and the ketone was obtained by flash column chromatography (pentane/Et_2O 20:1) as a colorless oil (18 mg, 74 µmol, 72%). **FT IR** (neat) ν (cm^{-1}): 2967, 2930, 2855, 169, 1603, 1492, 1477, 1448, 1393, 1365, 1306, 12291206, 1009, 1068, 1030, 976, 907, 845, 773, 756, 699, 682, 622. ^{1}**H NMR** (300 MHz, $CDCl_3$, 299 K) δ (ppm) = 7.24 – 7.08 (m, 5H), 3.08 – 2.83 (m, 2H), 1.96 – 1.16 (m, 8H), 0.71 (s, 9H). ^{13}**C NMR** (75 MHz, $CDCl_3$, 299 K) δ (ppm) 217.9 (C), 145.2 (C), 128.3 (CH), 128.2 (CH), 126.4 (CH), 52.4 (CH), 46.1 (CH), 44.4 (C), 32.7 (CH_2), 32.0 (CH_2), 26.4 (CH_2), 25.9 (CH_3). **HRMS** (ESI) *m/z* = 267.1719 calcd. for $C_{17}H_{24}NaO^+$ $[M+Na]^+$, found: 267.1720.

Cyclohexyl(2-phenylcyclohexyl)methanone (S-28)

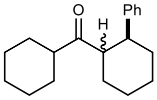

Alcohol **3m** (1.0 equiv, 0.137 mmol, 37 mg) was oxidized by the addition of DMP (1.5 equiv, 0.205 mmol, 87 mg) in anhydrous CH_2Cl_2 (2.0 mL) at 0 °C. After 2 hrs, the mixture was absorbed on silica and the ketones were obtained by flash column chromatography (pentane/Et_2O 9:1) as a colorless solid (25 mg, 92 µmol, 68%, *dr*: 1.2:1). **MP**.: 60 °C. **FT IR** (neat) ν (cm^{-1}): 2928, 2853, 1702, 1602, 1493, 1448, 1372, 1315, 1288, 1246, 1143, 1070, 1031, 980, 908, 850, 757, 729, 699, 648. ^{1}**H NMR** (300 MHz, $CDCl_3$, 299 K) δ (ppm) = 7.29 – 7.09 (m, 11H, both isomers), 3.27 – 3.14 (m, 1H, minor isomer), 2.90 – 2.71 (m, 4H, both isomers), 2.61 – 2.37 (m, 1H, major isomer), 2.02 – 0.65 (m, 44H, both isomers). ^{13}**C NMR** (75 MHz, $CDCl_3$, 299 K) δ (ppm) = 216.8 (C), 216.7 (C), 145.1 (C), 145.0 (C), 128.4 (CH), 128.2 (CH), 127.9 (CH), 127.8 (CH), 126.4 (CH), 126.2 (CH), 56.0 (CH), 51.8 (CH), 51.4 (CH), 50.4 (CH), 46.3 (CH), 45.7 (CH), 33.6 (CH_2), 30.3 (CH_2), 28.8 (CH_2), 28.3 (CH_2), 28.1 (CH_2), 27.3 (CH_2), 27.3 (CH_2), 26.5 (CH_2), 26.3 (CH_2), 26.3 (CH_2), 25.9 (CH_2), 25.9 (CH_2), 25.8 (CH_2), 25.8 (CH_2), 25.6 (CH_2), 25.6 (CH_2), 21.4 (CH_2). **HRMS** (ESI) *m/z* = 293.1876 calcd. for $C_{19}H_{26}NaO^+$ $[M+Na]^+$, found: 293.1879.

Optimization Studies: The optimization was carried out according to **GP5** using compound **2l-1** (0.29 mmol) as the test system and the reaction conditions were varied as described in Supplementary Table 1. After the mentioned reaction time, the ratio between the desired product **3l-1** and the hydro dehalogenated product **S-29** was determined by ^{1}H NMR spectroscopy ($CDCl_3$) of the crude product.

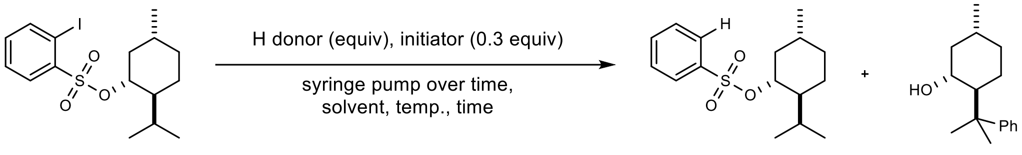

Supplementary Table 1 Screening of Reaction Conditions for the Arylation Reaction.

Entry	H-Donor (equiv)	Addition Time (h)	Solvent (mL)	Initiator (0.30 equiv)	S-29	:	3l-1[a]
**1**	Bu_3SnH (1.5)	6	PhH (10)	AIBN	49	:	51
**2**	TTMSS (1.5)	6	PhH (10)	AIBN	42	:	58
**3**[b]	TTMSS (1.5)	16	PhH (10)	AIBN	> 99	:	< 1
**4**	TTMSS (1.5)	2	PhH (10)	AIBN	34	:	66
**5**	TTMSS (1.5)	1	PhH (10)	AIBN	39	:	61
**6**	TTMSS (1.5)	2	PhH (15)	AIBN	41	:	59
**7**	TTMSS (1.5)	2	PhH (5)	AIBN	45	:	55
**8**	TTMSS (1.2)	2	PhH (10)	AIBN	34	:	66
**9**	TTMSS (1.3)	2	PhH (10)	AIBN	41	:	59
**10**	TTMSS (1.4)	2	PhH (10)	AIBN	32	:	68
**11**[c]	**TTMSS (1.4)**	**2**	PhH (10)	**AIBN**	**30**	**:**	**70/65%**[d]
**12**	TTMSS (1.4)	1.5	PhH (10)	AIBN	46	:	54
**13**	TTMSS (1.4)	2.5	PhH (10)	AIBN	32	:	68
**14**[e]	TTMSS (1.3)	2	MeCN (10)	AIBN	Traces of **3l-1**		
**15**[f]	TTMSS (1.4)	2	Toluene (10)	AIBN	55	**:**	45
**14**[g]	TTMSS (1.4)	2	BTF (10)	AIBN	52	:	48
**16**[h]	TTMSS (1.4)	2	PhH (10)	Et_3B	Traces of **3l-1**		
**17**	Ph_3SiH (1.4)	2	PhH (10)	AIBN	No conversion		
**18**	Ph_3SiH (1.4)	2	PhH (10)	BPO[i]	No conversion		

General conditions: 0.29 mmol of **2l-1**, 6 hrs total reaction time, 90 °C. [a]Determined by ^{1}H NMR spectroscopy ($CDCl_3$) of the crude product analyzing the CHO protons of the product. [b]Total reaction time 16 hrs. [c]95 °C. [d]Isolated yield. [e]TTMSS and AIBN dissolved in PhH. [f]110 °C. [g]100 °C. [h]Et_3B added to the starting material in one portion, TTMSS added via syringe pump, rt, flask open to air. [i]BPO: dibenzoyl peroxide.

DFT Calculations: All structures were optimized without geometry constraints using the PBE0 hybrid functional (*20*,*21*) and an atom-pairwise dispersion correction (D3) (*22*,*23*). A flexible triple zeta basis set (def2-TZVP) (*24*) was used in all calculations. For the calculation of the free enthalpy contributions (G^{RRHO}(298K)), a rotor approximation was applied for vibrational modes with wave numbers below 100 cm-1 (*25*). The nature of all optimized stationary points was proven by the presence of either 0 (minimum) or 1 (transition structure) imaginary vibrational frequency.

Electronic energies were recalculated with the double hybrid functional PWPB95(-D3) (*26*) using the structures optimized with PBE0-D3. PWPB95 includes a component of the correlation energy which is computed by perturbation theory and performs more accurately in the determination of energies, even for open shell molecules such as radicals. The final value for the free enthalpy ΔG(298) was obtained using the PWPB95-D3 electronic energies and $^{GRRHO(}$298K), obtained with PBE0-D3.

All geometry optimizations and vibrational frequency calculations were performed with the TURBOMOLE 7.1 and 7.2 program (*27*). PWPB95-D3 calculations were performed with the ORCA (4.0.2) program (*28*).

DFT exploration of the mechanism and regioselectivity for the n-hexyl derivative 2o

We first investigated the reaction of the radical generated after iodine atom abstraction from **2o** (**2o-PhRad**). Transition structures of 1,5-, (**2o-TS1(1,5)**) 1,6-, 1,7- and 1,8-H atom transfer from different methylene groups (both diastereotopic hydrogen atoms, **2o-TS1(1,*n*)a,b**) were located (Supplementary Figure 2, 3). All transition structures correspond to very low free energy barriers ($\Delta G^{‡}$(298)), but clearly the 1,7 hydrogen transfer is the fastest with a barrier of around 3 kcal/mol. It is therefore highly likely that hydrogen atom abstraction is the initial step after iodine atom release from **2a-u**, and that there is a preference for the 1,7-hydrogen transfer.

In the following, we have been looking at the subsequent reactions only of the 1,7-H transfer product **2o-INT1**. We have located cyclic transition structures with short distances between the *ipso* position of the phenyl group and the alkyl radical carbon atom (Supplementary Figure 4, 6). The C-C bond formation is stereoselective, as can be seen from the free energies of the stereoisomeric transition structures **2o-TS2a** and **2o-TS2b**. The formation of the *syn* cyclohexadienyl intermediate **2o-INT2a** has a lower barrier ($\Delta G^{‡}$(298) = 11.3 kcal/mol) than that of **2o-INT2b** ($\Delta G^{‡}$(298) = 13.7 kcal/mol). The final step, fragmentation of the C-S bond (via the cyclic transition structure **2o-TS3**) is much faster ($\Delta G^{‡}$(298) < 5 kcal/mol for both intermediates) and thus does not allow the reverse C-C bond breaking (Supplementary Figure 5).

Thus, the formation of the preferred stereoisomer of **3o** (not calculated here) can be explained by the kinetic preference in the C-C bond forming step of the mechanism.

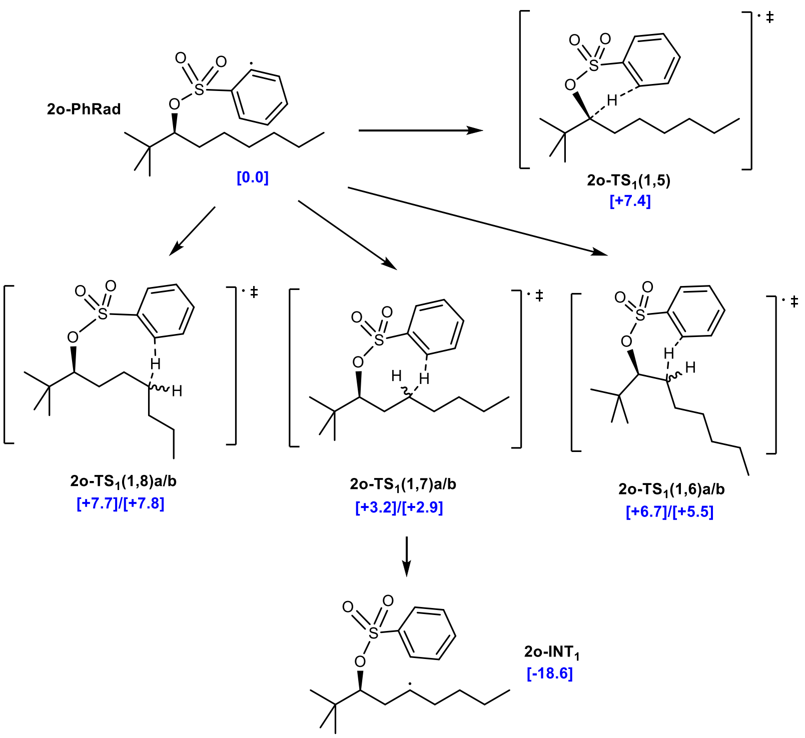

Supplementary Figure 2 DFT-calculated free energies ΔG(298)[kcal/mol] (in blue) in the hydrogen atom transfer step of the reaction of radical **2o-PhRad**. Free energies are all given relative to **2o-PhRad**.

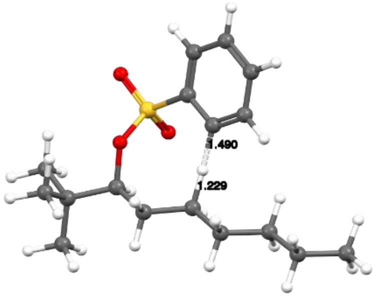

2o-TS1(1,7)a

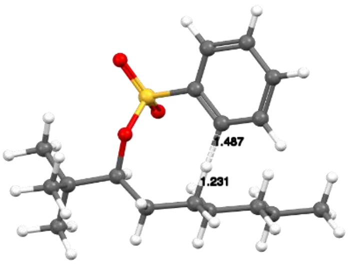

2o-TS1(1,7)b

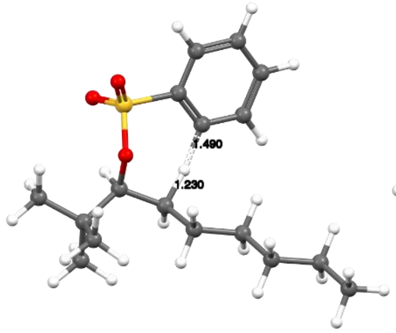

2o-TS1(1,6)a

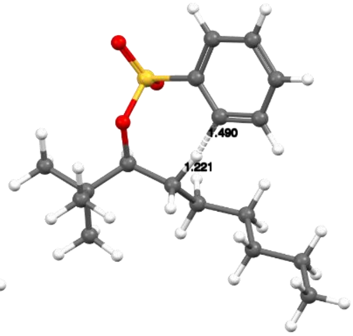

2o-TS1(1,6)b

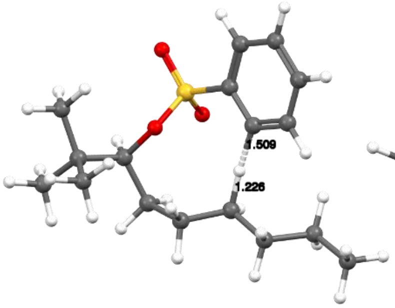

2o-TS1(1,8)a

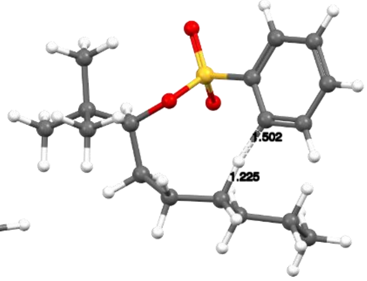

2o-TS1(1,8)b

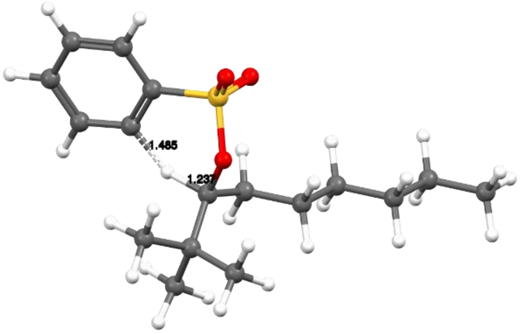

2o-TS1(1,5)

Supplementary Figure 3 Transition structure (PBE0-D3/def2-TZVP) of H atom transfer from **2o-PhRad**. Interatomic distances are given in Å.

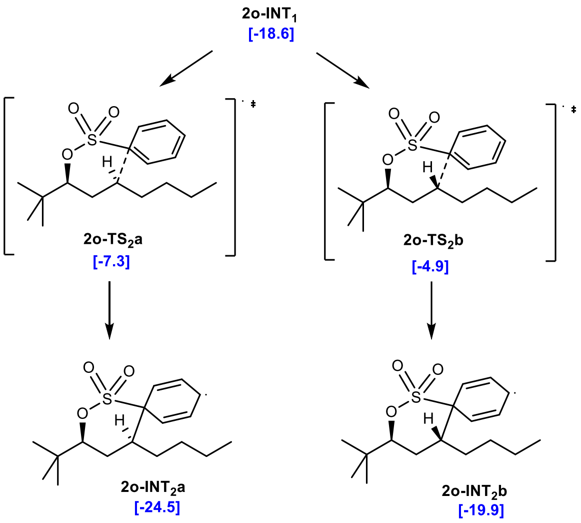

Supplementary Figure 4 DFT-calculated free energies ΔG(298)/[kcal/mol] (in blue) in the C-C bond formation step of the reaction of radical **2o-PhRad**. Free energies are all given relative to **2o-PhRad**.

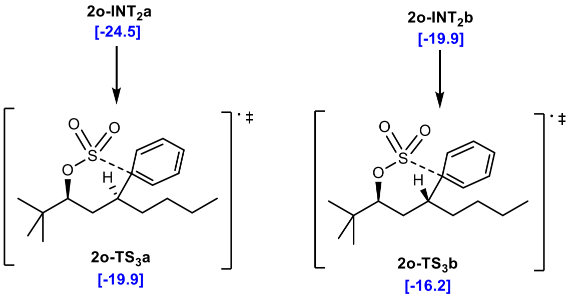

Supplementary Figure 5 DFT-calculated free energies ΔG(298)/[kcal/mol] (in blue) in the C-S bond dissociation step of the reaction of radical **2o-PhRad**. Free energies are all given relative to **2o-PhRad**.

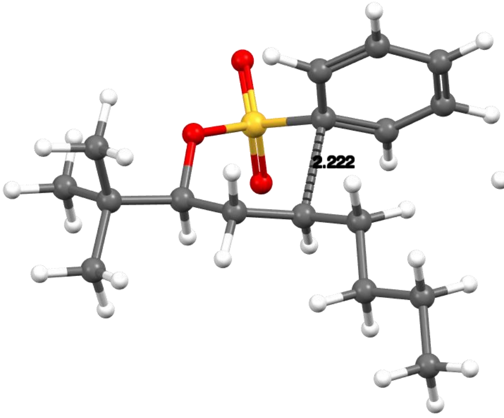

2o-TS2a

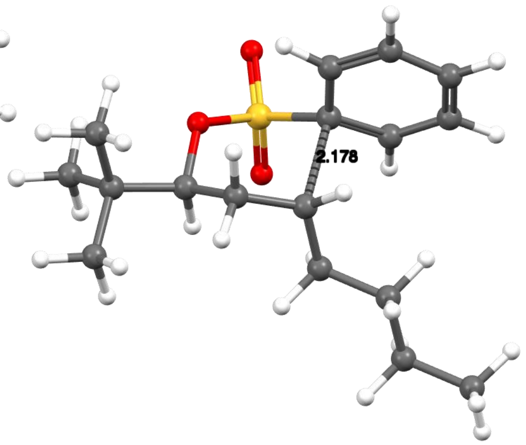

2o-TS2b

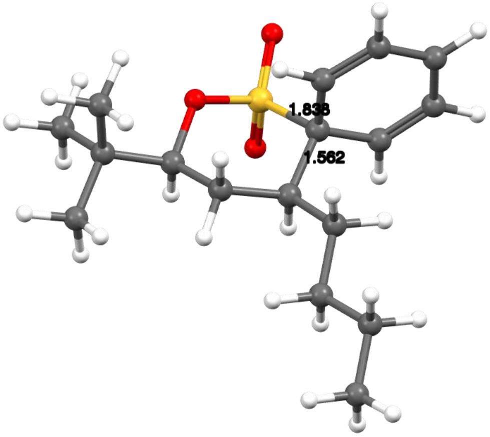

2o-INT2a

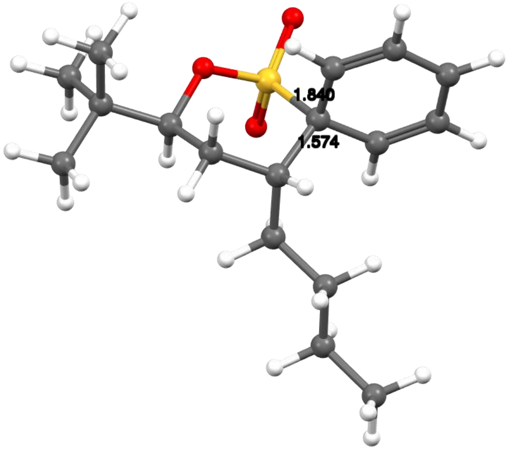

2o-INT2b

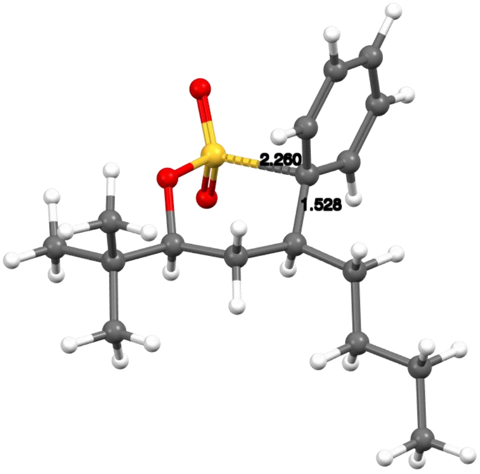

2o-TS3a

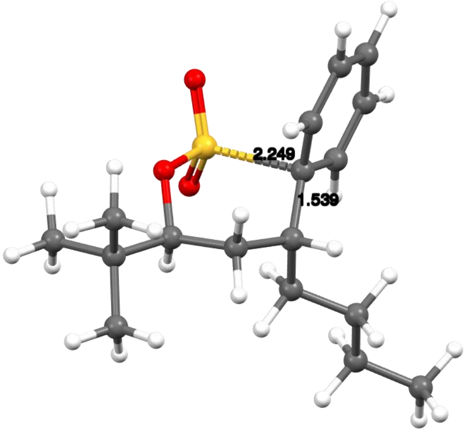

2o-TS3b

Supplementary Figure 6 Transition structures and cyclohexadienyl radical intermediate (PBE0-D3/def2-TZVP) of the aryl transfer for **2o-PhRad**. Interatomic distances are given in Å.

DFT exploration of the mechanism and regioselectivity for the cyclohexyl derivative 2n

The 1,7-H atom transfer in the initial step of the reaction of **2n-PhRad** may occur at two diastereotopic positions (**2n-TS1A(a/b**) and **2n-TS1B(a/b)**), leading to two intermediates **2n-INT1A** and **2n-INT1B** (Supplementary Figure 7). The free energies of the transition structures of the hydrogen atom transfer from the possible four positions indicate a very low selectivity. Both diastereomers of the alkyl radical, **2n-INT1(A/B)**, are formed via a low barrier of 3.7-3.9 kcal/mol. The experimental observation that the formation of **3n** is unselective with respect to the hydrogen atom transfer is fully confirmed.

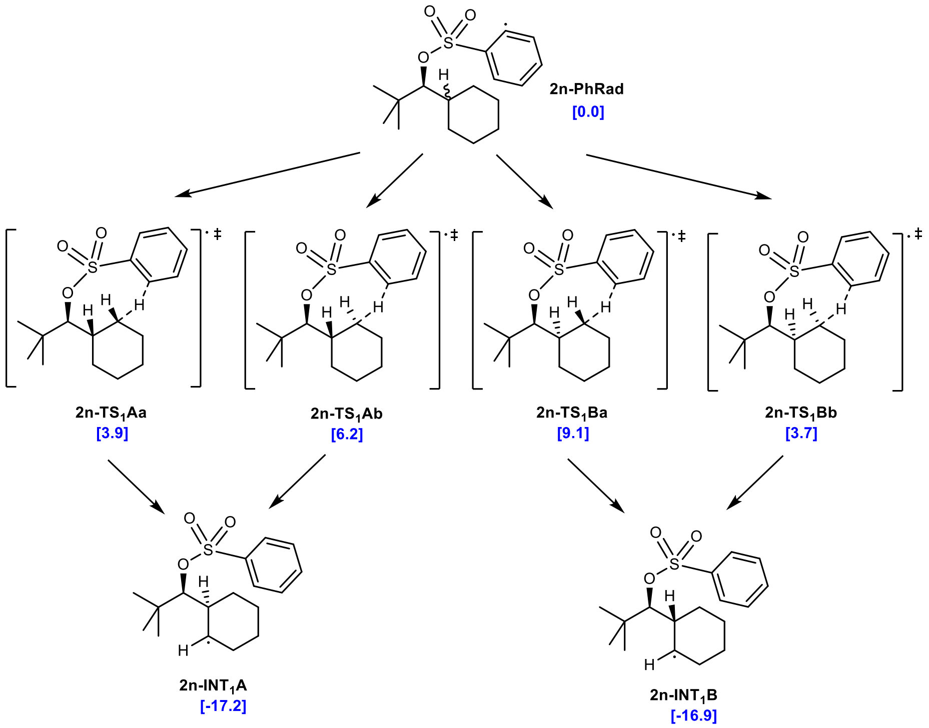

Supplementary Figure 7 DFT-calculated free energies ΔG(298)/[kcal/mol] (in blue) in the hydrogen atom transfer step of the reaction of **2n-PhRad**. Free energies are all given relative to **2n-PhRad**.

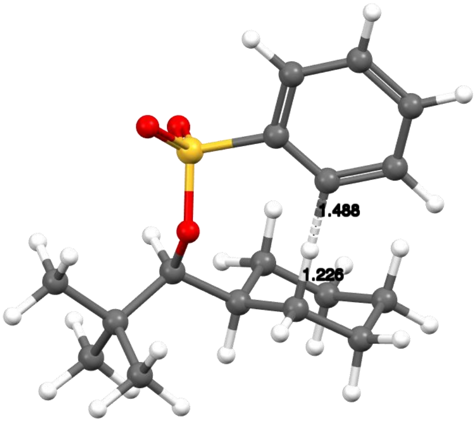

2n-TS1Aa

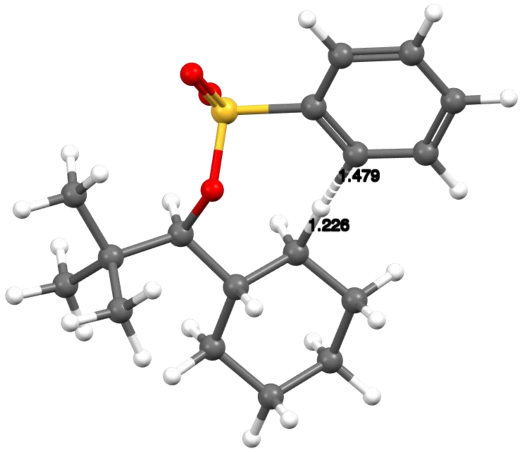

2n-TS1Ab

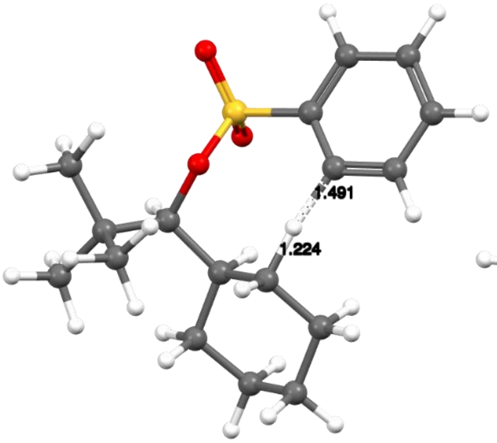

2n-TS1Ba

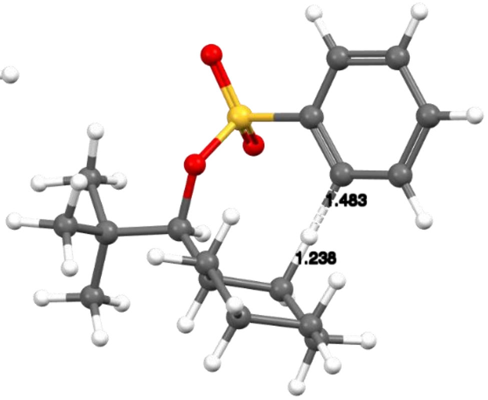

2n-TS1Bb

Supplementary Figure 8 Four diastereomeric transition structures (PBE0-D3/def2-TZVP) of H atom transfer from **2n-PhRad**. Interatomic distances are given in Å.

We have further optimized all possible stereoisomeric pathways. The C-C bond formation step of the two intermediates **2n-INT1A/B** kinetically favors one diastereoisomer over the other. In both cases the same relative configuration with respect to the initial stereocenter is formed faster via **2n-TS2Ab** and **2n-TS2Ba**, explaining the observed high stereoselectivity (Supplementary Figure 9). As in the reaction of **2o-PhRad**, the C-C bond formation is the step with the highest barrier (12.1 kcal/mol for **2n-TS2Ab**), i.e. it is the rate-determining step. The dissociation of the cyclohexadienyl radical **2n-INT2(A/B)(a/b)** has a comparably low barrier of 2-6 kcal/mol.

Supplementary Figure 9 DFT-calculated free energies ΔG(298)/[kcal/mol] (in blue) in the C-C bond formation step of the reaction of **2n-PhRad**. Free energies are all given relative to **2n-PhRad**.

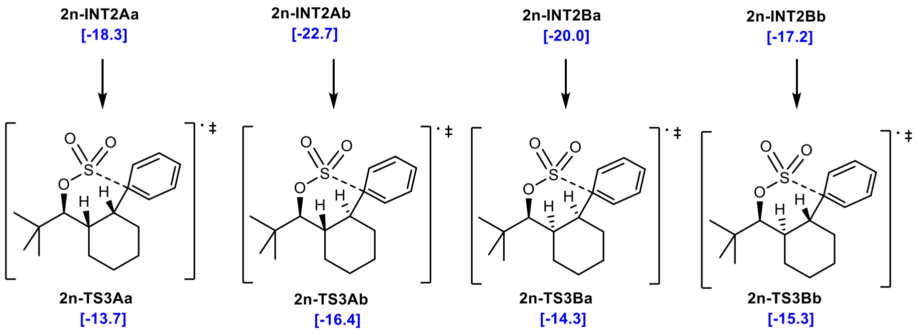

Supplementary Figure 10 DFT-calculated free energies ΔG(298)/[kcal/mol] (in blue) in the C-S bond dissociation step of the reaction of **2n-PhRad**. Free energies are all given relative to **2n-PhRad**.

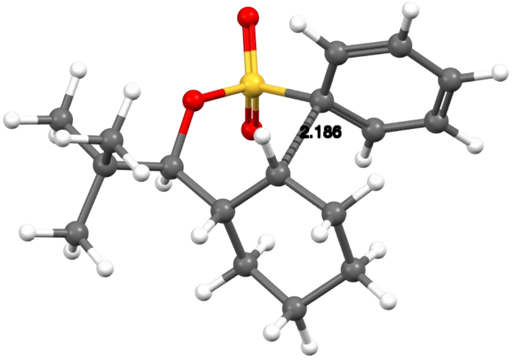

2n-TS2Aa

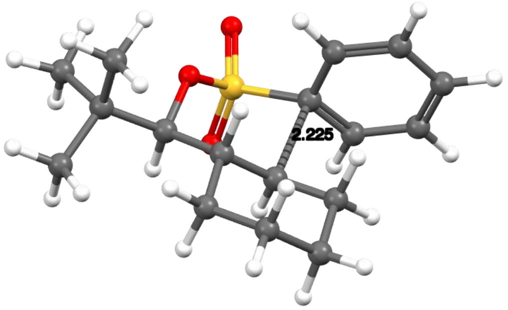

2n-TS2Ab

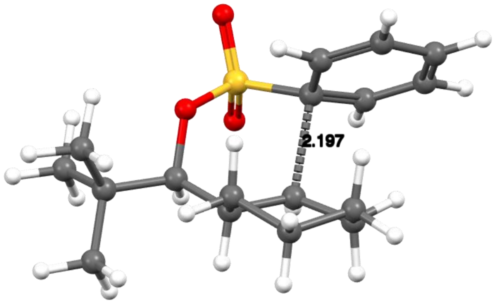

2n-TS2Ba

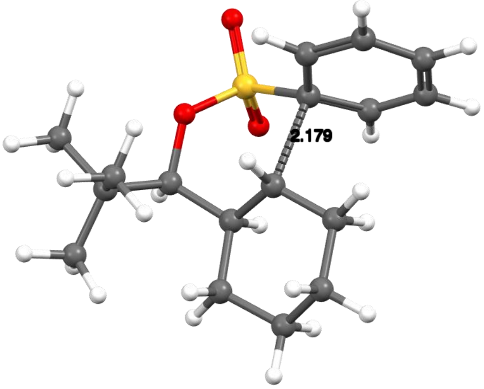

2n-TS2Bb

Supplementary Figure 11 Four diastereomeric transition structures (PBE0-D3/def2-TZVP) of C-C bond formation from **2n-INTa** or **2n-INTb**. Interatomic distances are given in Å.

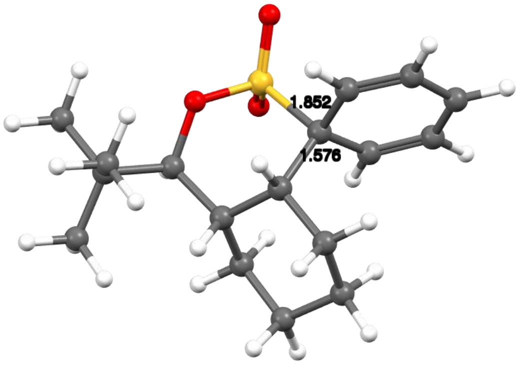

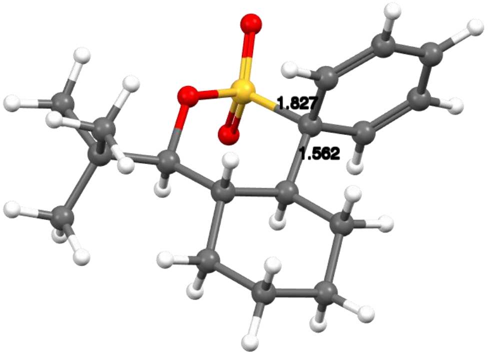

2n-INT2Aa **2n-INT2Ab**

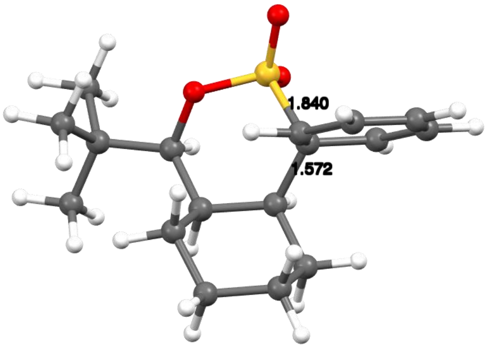

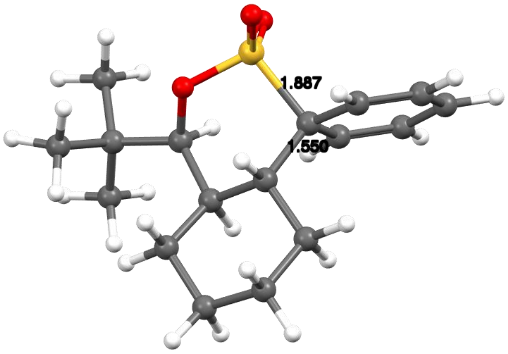

2n-INT2Ba **2n-INT2Bb**

Supplementary Figure 12 Cyclohexadienyl radical intermediates (PBE0-D3/def2-TZVP) during the aryl transfer for **2n-PhRad**. Interatomic distances are given in Å

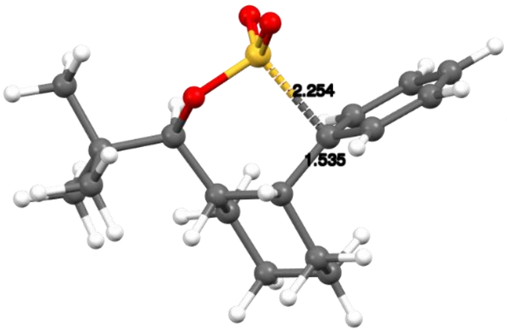

2n-TS3Aa

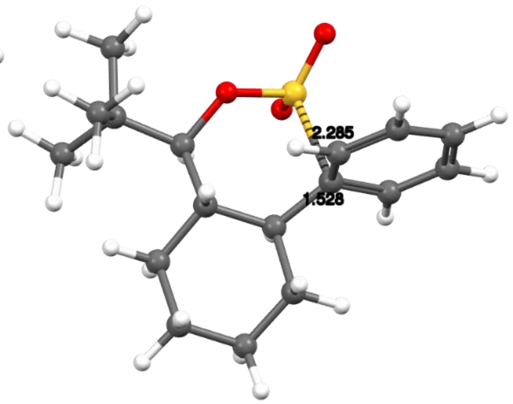

2n-TS3Ab

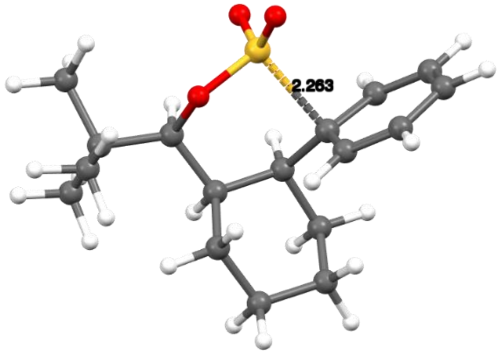

2n-TS3Ba

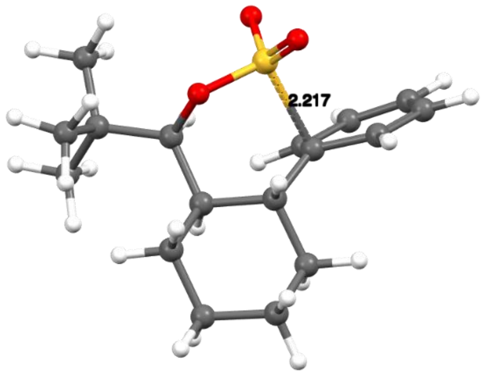

2n-TS3Bb

Supplementary Figure 13 Transition structures of homolytic C-S bond fragmentation (PBE0-D3/def2-TZVP) during the aryl transfer for **2n-PhRad**. Interatomic distances are given in Å.

Supplementary Table 2 Electronic energies (E) and thermodynamic correction to the Gibbs Free Energy at T = 298.15 K (G298) for the structures involved in the reactions

Structure	E(PBE0-D3)[a] [E_h]	G^{RRHO}(298)[a] [kcal/mol]	E(PWPB95-D3)[a][b] [E_h]	ΔG(298)[c] [kcal/mol]
**2n-PhRad**	-1285.872683	219.167	-1286.391728	0.0
**2n-TS1Aa**	-1285.864402	216.413	-1286.381188	3.9
**2n-TS1Ab**	-1285.861154	216.732	-1286.378024	6.2
**2n-TS1Ba**	-1285.856356	216.818	-1286.373451	9.1
**2n-TS1Bb**	-1285.865359	216.458	-1286.381550	3.7
**2n-INT1A**	-1285.898864	218.013	-1286.417215	-17.1
**2n-INT1B**	-1285.899203	218.496	-1286.417506	-16.8
**2n-TS2Aa**	-1285.880927	219.849	-1286.398517	-3.6
**2n-TS2Ab**	-1285.883273	219.946	-1286.401100	-5.1
**2n-TS2Ba**	-1285.881517	219.922	-1286.398360	-3.4
**2n-TS2Bb**	-1285.873720	220.082	-1286.391803	0.9
**2n-INT2Aa**	-1285.906986	221.419	-1286.424402	-18.3
**2n-INT2Ab**	-1285.914166	221.091	-1286.430971	-22.7
**2n-INT2Ba**	-1285.909119	221.518	-1286.425669	-18.9
**2n-INT2Bb**	-1285.904894	221.299	-1286.422460	-17.2
**2n-TS3Aa**	-1285.899083	220.584	-1286.415743	-13.7
**2n-TS3Ab**	-1285.901522	220.150	-1286.419447	-16.4
**2n-TS3Ba**	-1285.899234	220.581	-1286.416731	-14.3
**2n-TS3Bb**	-1285.900150	220.497	-1286.418230	-15.3
**2o-PhRad**	-1287.067724	228.680	-1287.583373	0.0
**2o-TS1(1,7)b**	-1287.062710	226.619	-1287.575430	2.9
**2o-TS1(1,7)a**	-1287.061856	226.384	-1287.574601	3.2
**2o-TS1(1,8)a**	-1287.054540	227.025	-1287.568471	7.7
**2o-TS1(1,8)b**	-1287.055190	227.409	-1287.568858	7.8
**2o-TS1(1,6)a**	-1287.056547	226.715	-1287.569536	6.7
**2o-TS1(1,6)b**	-1287.057616	226.415	-1287.571013	5.5
**2o-TS1(1,5)**	-1287.054479	226.073	-1287.567454	7.4
**2o-INT1**	-1287.096926	228.027	-1287.611966	-18.6
**2o-TS2a**	-1287.082526	229.706	-1287.596563	-7.3
**2o-TS2b**	-1287.079618	230.078	-1287.593456	-4.9
**2o-INT2a**	-1287.113142	231.282	-1287.626606	-24.5
**2o-INT2b**	-1287.106252	231.849	-1287.620139	-19.9
**2o-TS3a**	-1287.103379	230.170	-1287.617455	-19.9
**2o-TS3b**	-1287.097573	230.704	-1287.612345	-16.2

[a] All energies have been calculated with the def2-TZVP basis set
[b] Energy calculation for the structure optimized with PBE0-D3/def2-TZVP
[c] ΔG(298) = ΔE(PWPB95-B3) + ΔG^{RRHO}(298K, PBE0-D3)

X-Ray diffraction Analysis: Data sets for compound **S-26** were collected with a D8 Venture CMOS diffractometer. Data sets for compound **3p** were collected with an APEX II CCD diffractometer. Programs used: data collection: APEX3 V2016.1-0 (Bruker AXS Inc., **2016**); cell refinement: SAINT V8.37A (Bruker AXS Inc., **2015**); data reduction: SAINT V8.37A (Bruker AXS Inc., **2015**); absorption correction, SADABS V2014/7 (Bruker AXS Inc., **2014**); structure solution SHELXT-2015 (Sheldrick, **2015**); structure refinement SHELXL-2015 (Sheldrick, **2015**) (*29*). *R*-values are given for observed reflections, and wR^2 values are given for all reflections.

X-ray crystal structure analysis of 3p: A colorless prism-like specimen of $C_{16}H_{26}O$, approximate dimensions 0.060 mm x 0.060 mm x 0.100 mm, was used for the X-ray crystallographic analysis. The X-ray intensity data were measured. A total of 3325 frames were collected. The total exposure time was 59.22 hours. The frames were integrated with the Bruker SAINT software package using a wide-frame algorithm. The integration of the data using a triclinic unit cell yielded a total of 28420 reflections to a maximum θ angle of 66.74° (0.84 Å resolution), of which 7718 were independent (average redundancy 3.682, completeness = 96.8%, R_{int} = 10.04%, R_{sig} = 10.74%) and 4364 (56.54%) were greater than $2\sigma(F^2)$. The final cell constants of a = 12.0188(10) Å, b = 15.2479(13) Å, c = 15.2748(15) Å, α = 117.435(5)°, β = 96.968(7)°, γ = 107.897(8)°, volume = 2246.8(4) $Å^3$, are based upon the refinement of the XYZ-centroids of 1681 reflections above 20 σ(I) with 6.861° < 2θ < 128.1°. Data were corrected for absorption effects using the multi-scan method (SADABS). The ratio of minimum to maximum apparent transmission was 0.876. The calculated minimum and maximum transmission coefficients (based on crystal size) are 0.9550 and 0.9720. The final anisotropic full-matrix least-squares refinement on F^2 with 488 variables converged at R1 = 5.98%, for the observed data and wR2 = 16.54% for all data. The goodness-of-fit was 1.007. The largest peak in the final difference electron density synthesis was 0.252 $e^-/Å^3$ and the largest hole was -0.276 $e^-/Å^3$ with an RMS deviation of 0.059 $e^-/Å^3$. On the basis of the final model, the calculated density was 1.039 g/cm^3 and F(000), 780 e^-.

X-ray crystal structure analysis of S-26: A colorless needle-like specimen of $C_{17}H_{24}O$, approximate dimensions 0.020 mm x 0.050 mm x 0.132 mm, was used for the X-ray crystallographic analysis. The X-ray intensity data were measured. A total of 1687 frames were collected. The total exposure time was 24.82 hours. The frames were integrated with the Bruker SAINT software package using a wide-frame algorithm. The integration of the data using a monoclinic unit cell yielded a total of 21741 reflections to a maximum θ angle of 68.37° (0.83 Å resolution), of which 2621 were independent (average redundancy 8.295, completeness = 99.8%, R_{int} = 5.93%, R_{sig} = 3.12%) and 2246 (85.69%) were greater than $2\sigma(F^2)$. The final cell constants of a = 13.0527(4) Å, b = 5.7006(2) Å, c = 19.3889(6) Å, β = 97.0260(10)°, volume = 1431.86(8) $Å^3$, are based upon the refinement of the XYZ-centroids of 9612 reflections above 20 σ(I) with 6.823° < 2θ < 136.7°. Data were corrected for absorption effects using the multi-scan method (SADABS). The ratio of minimum to maximum apparent transmission was 0.878. The calculated minimum and maximum transmission coefficients (based on crystal size) are 0.9350 and 0.9900. The structure was solved and refined using the Bruker SHELXTL Software Package, using the space group $P2_1/c$, with Z = 4 for the formula unit,

$C_{17}H_{24}O$. The final anisotropic full-matrix least-squares refinement on F^2 with 166 variables converged at R1 = 3.98%, for the observed data and wR2 = 9.17% for all data. The goodness-of-fit was 1.044. The largest peak in the final difference electron density synthesis was 0.173 $e^-/Å^3$ and the largest hole was -0.175 $e^-/Å^3$ with an RMS deviation of 0.039 $e^-/Å^3$. On the basis of the final model, the calculated density was 1.134 g/cm^3 and F(000), 536 e^-.

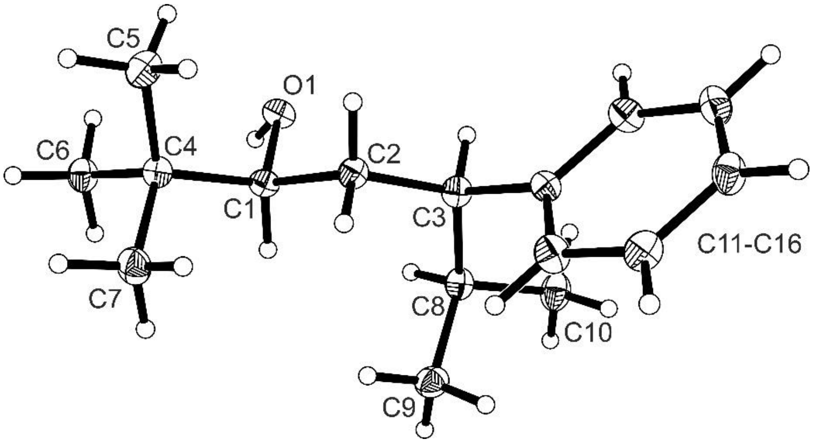

Supplementary Figure 14 Crystal structure of compound **3p**. Only one molecule (molecule "A") of tree found in the asymmetric unit is shown (Thermals ellipsoids are shown with 50% probability.).

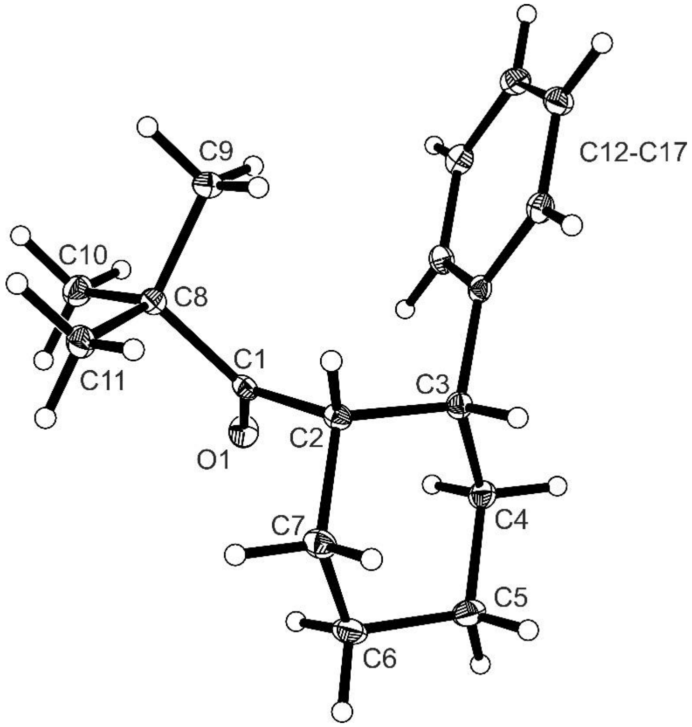

Supplementary Figure 15 Crystal structure of compound **S-26** (Thermals ellipsoids are shown with 50% probability.).

Spectral Data

^{1}H NMR (300 MHz, D_2O)

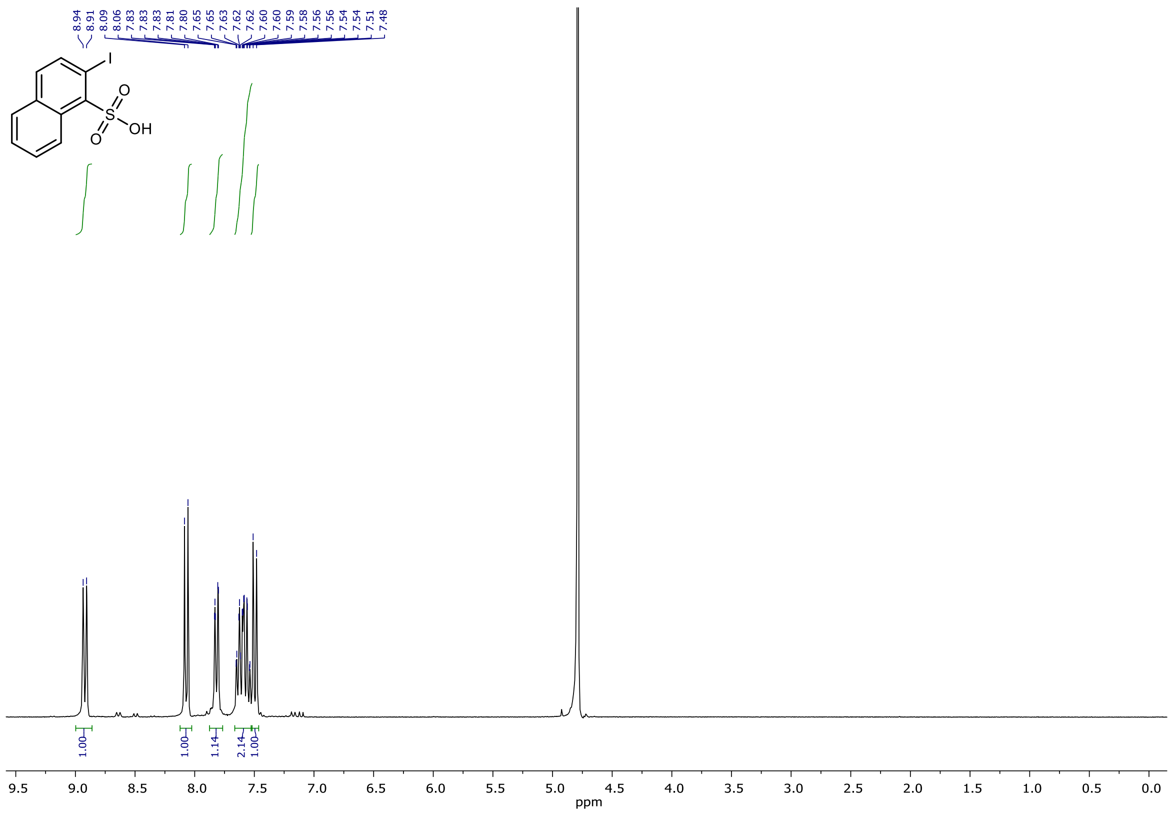

^{13}C NMR (75 MHz, D_2O)

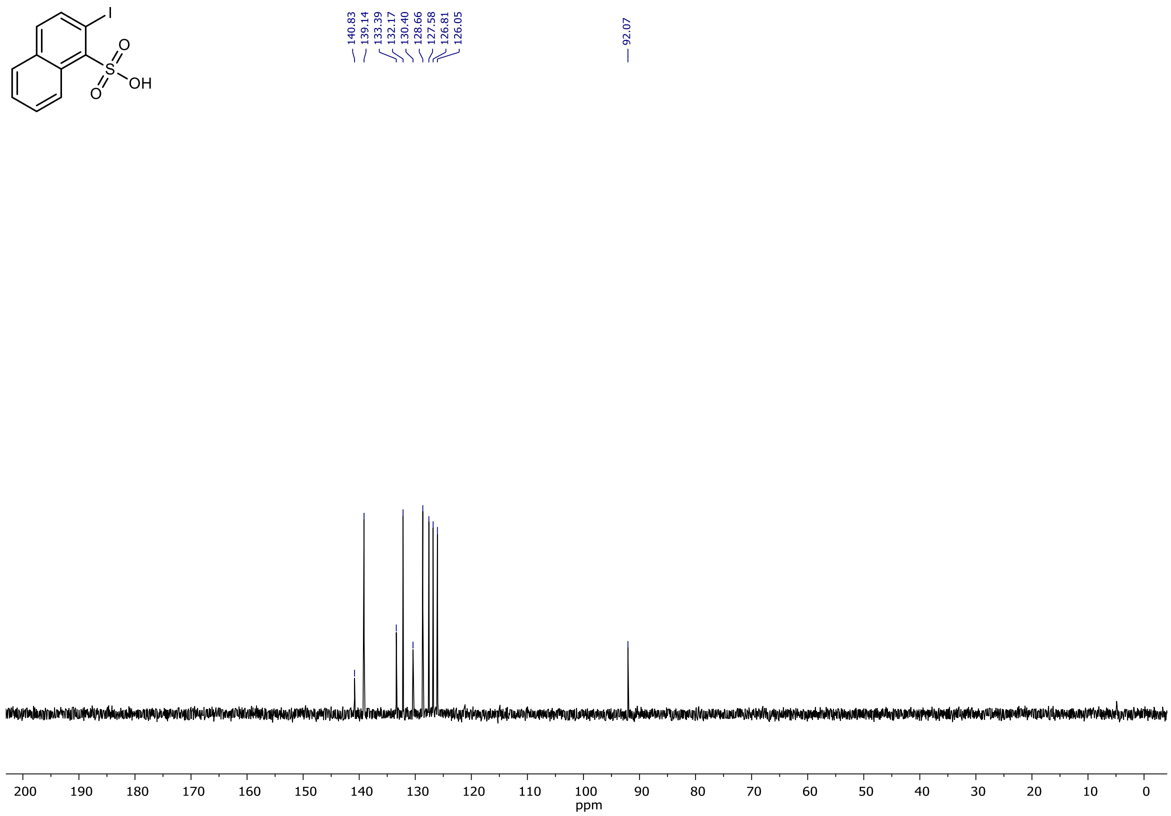

Supplementary Figure 16: 2-Iodonaphthalene-1-sulfonic acid (S-2)

^{1}H NMR (300 MHz, D_2O)

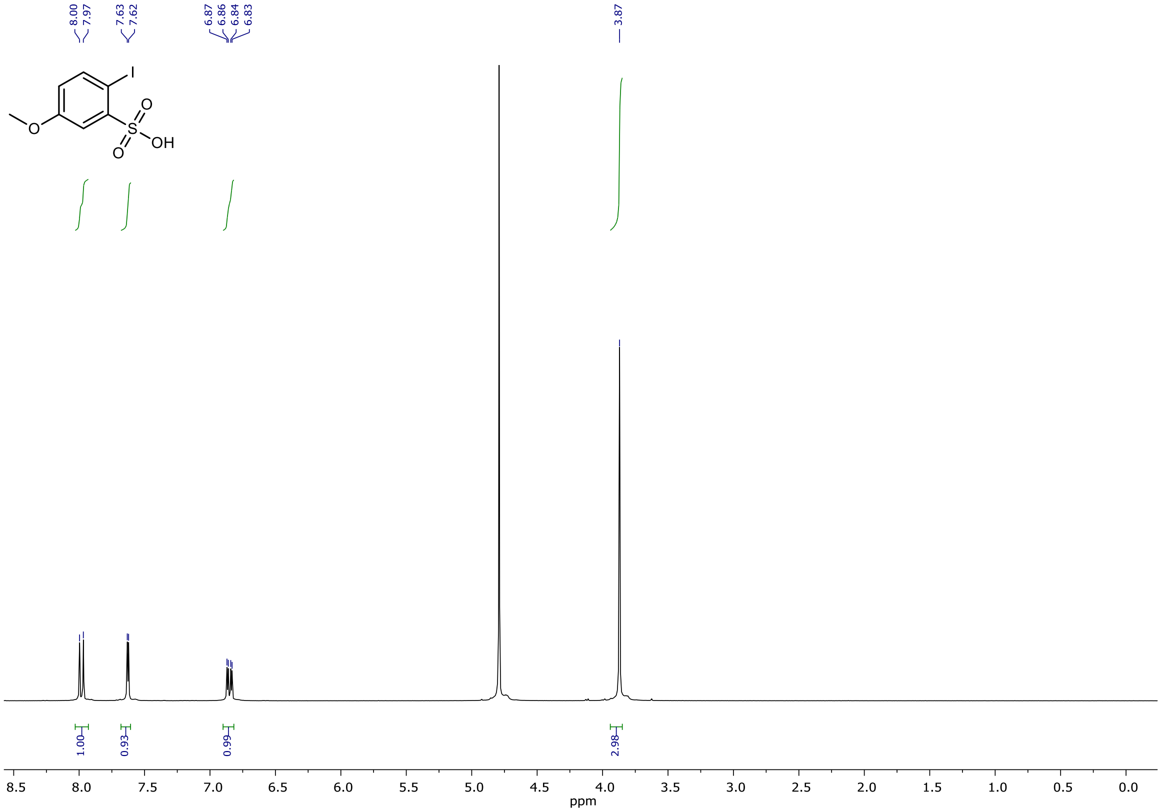

^{13}C NMR (75 MHz, D_2O)

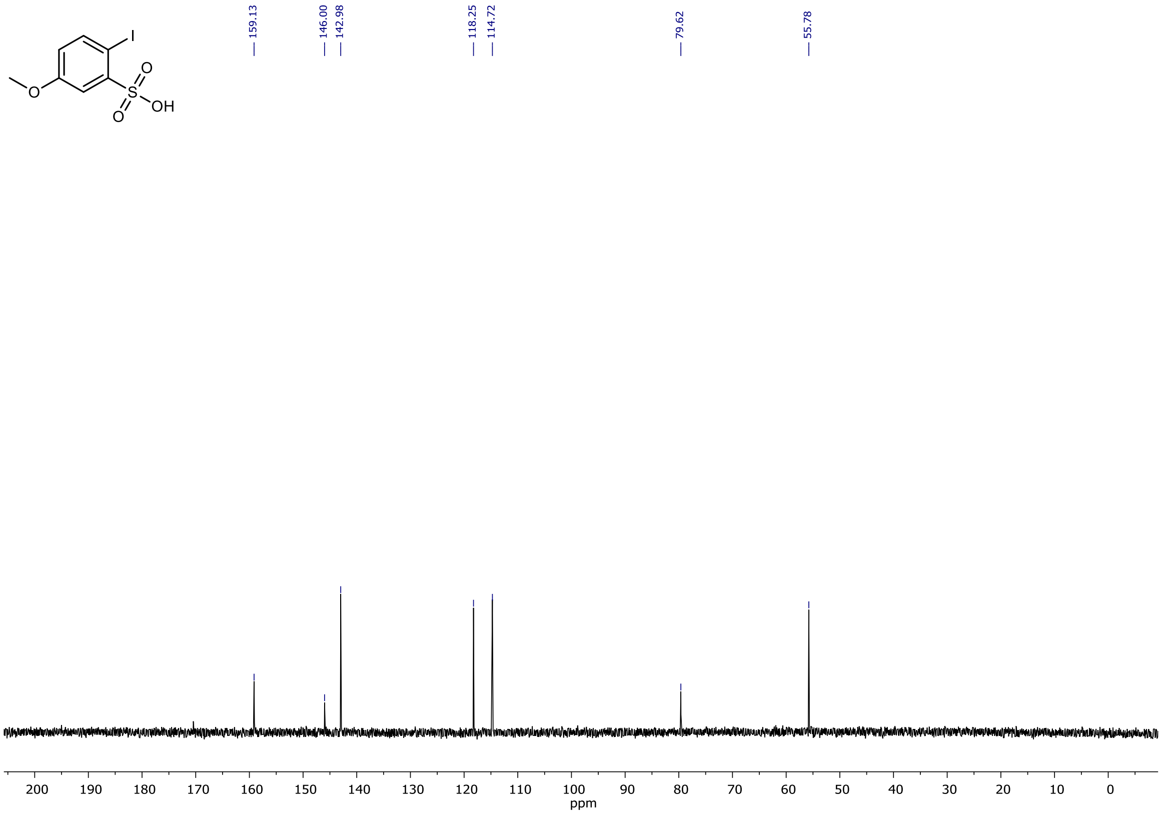

Supplementary Figure 17: NMR Spectra of 2-Iodo-5-methoxybenzenesulfonic acid (S-4)

^1H NMR (300 MHz, D_2O)

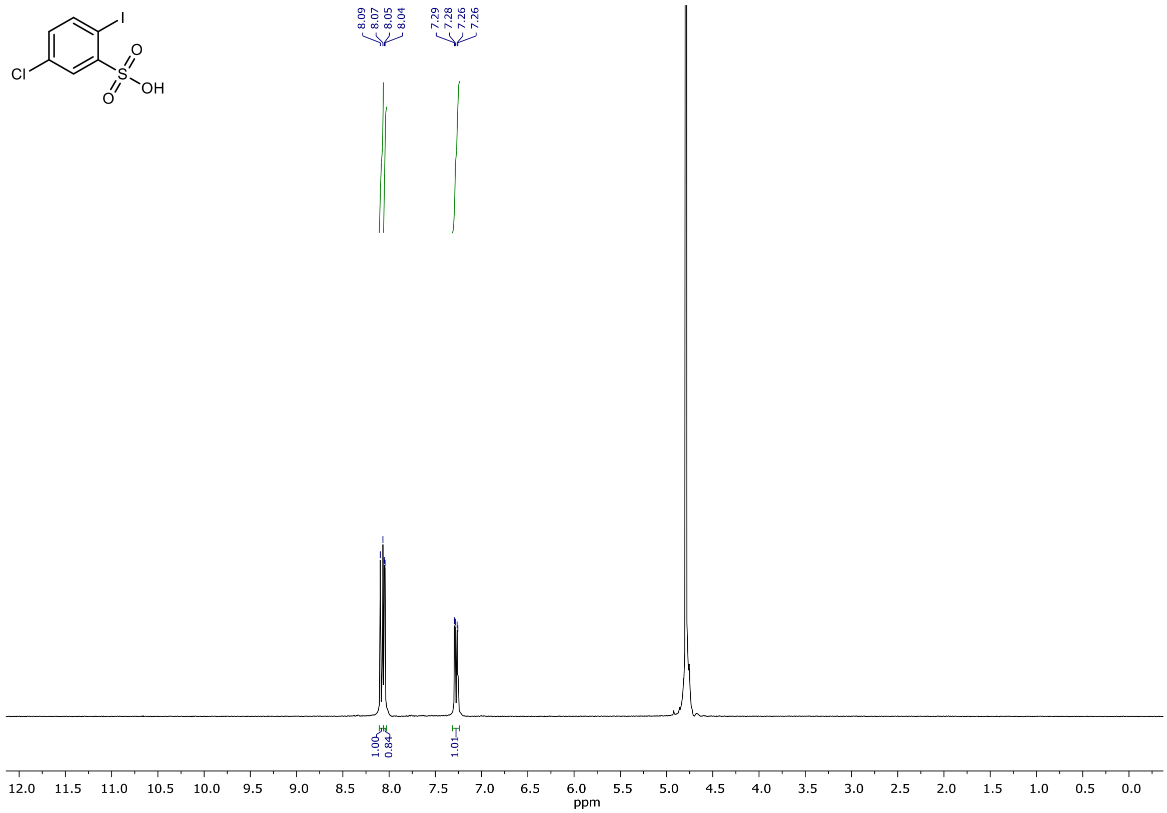

^{13}C NMR (75 MHz, D_2O)

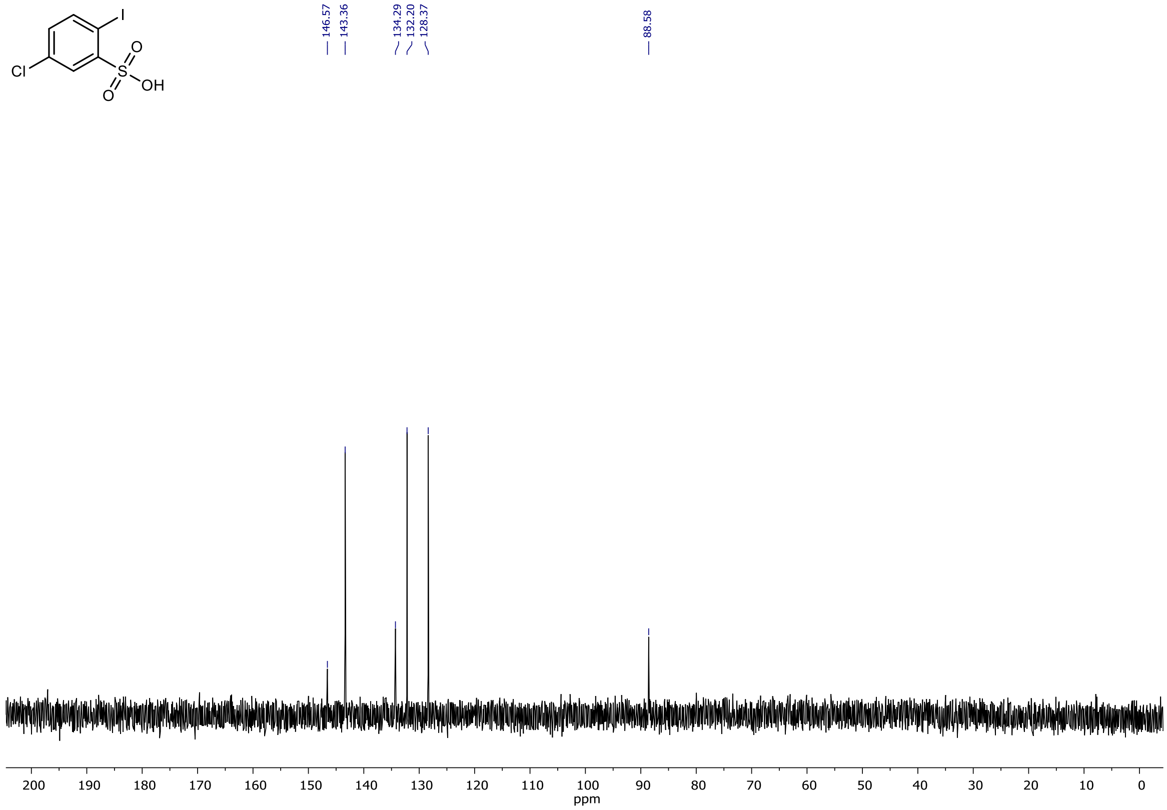

Supplementary Figure 18: NMR Spectra of 5-Chloro-2-iodo-benzenesulfonic acid (S-5)

^{1}H NMR (300 MHz, DMSO-d_6)

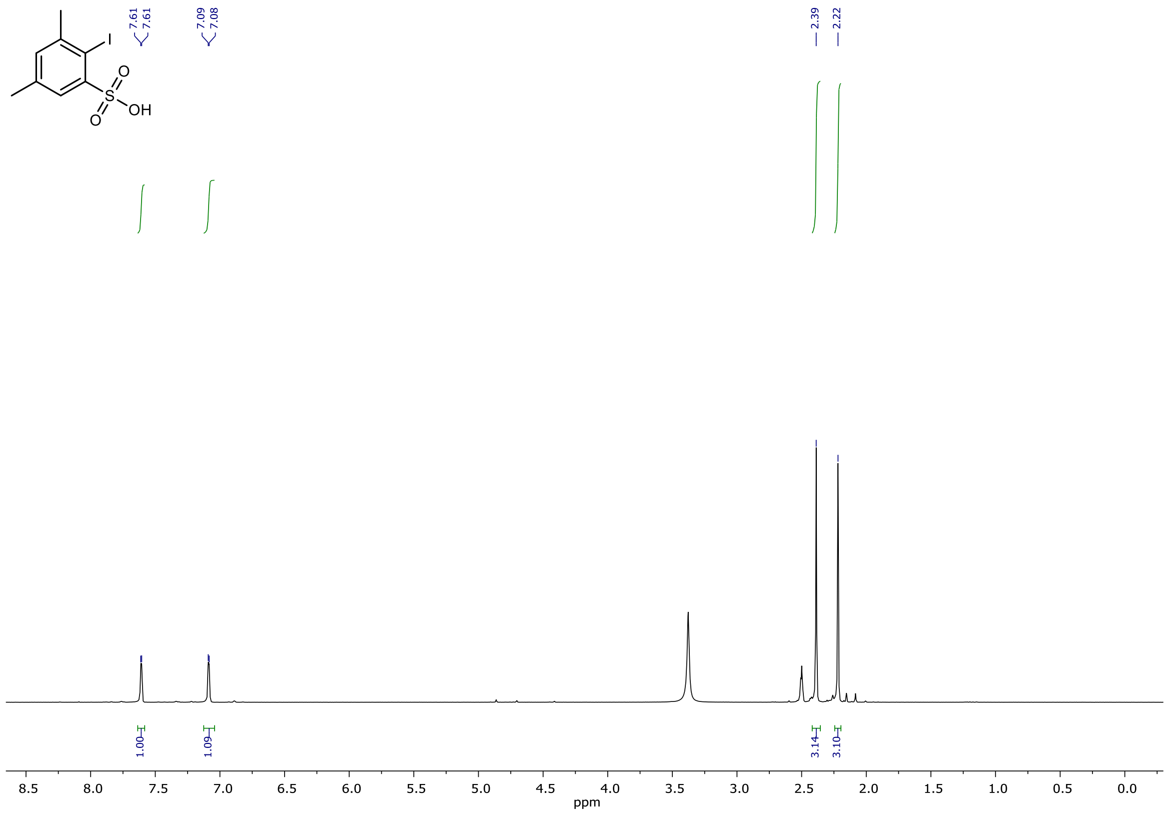

^{13}C NMR (75 MHz, DMSO-d_6)

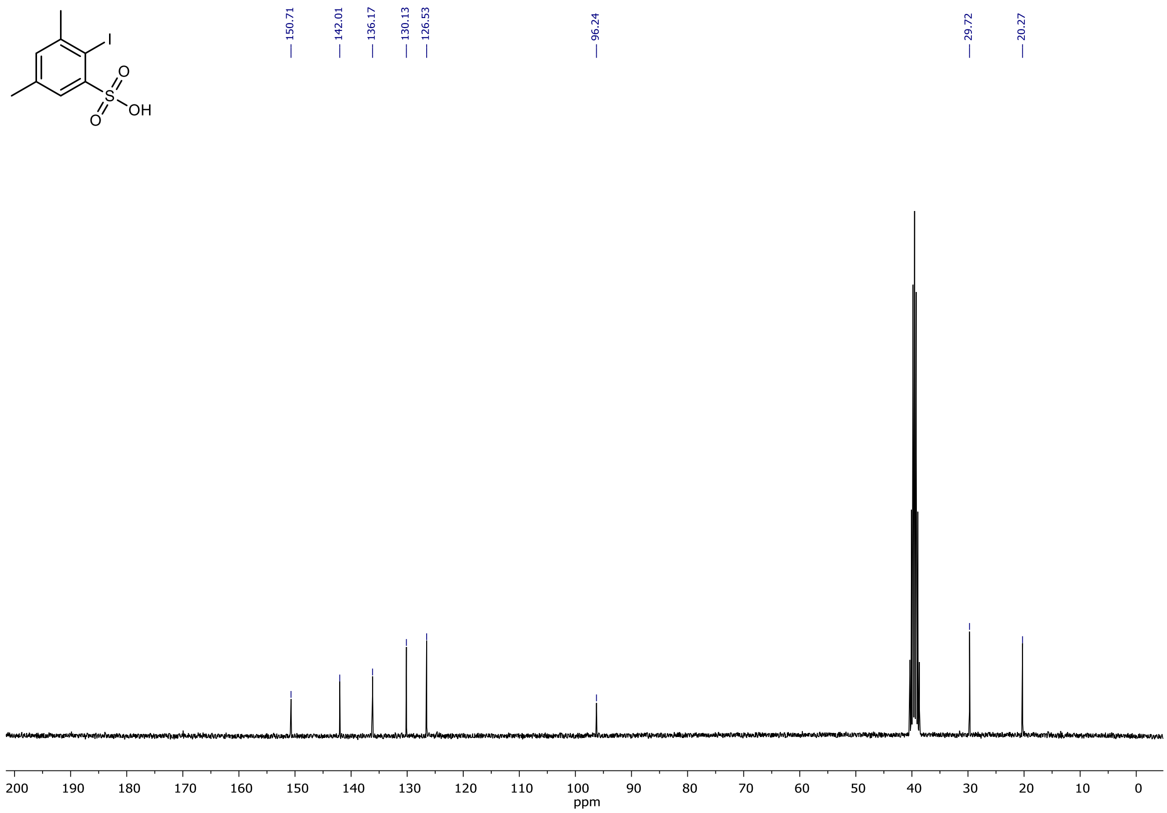

Supplementary Figure 19: NMR Spectra of 2-Iodo-3,5-dimethylbenzenesulfonic acid (S-6)

^{1}H NMR (400 MHz, DMSO-d_6)

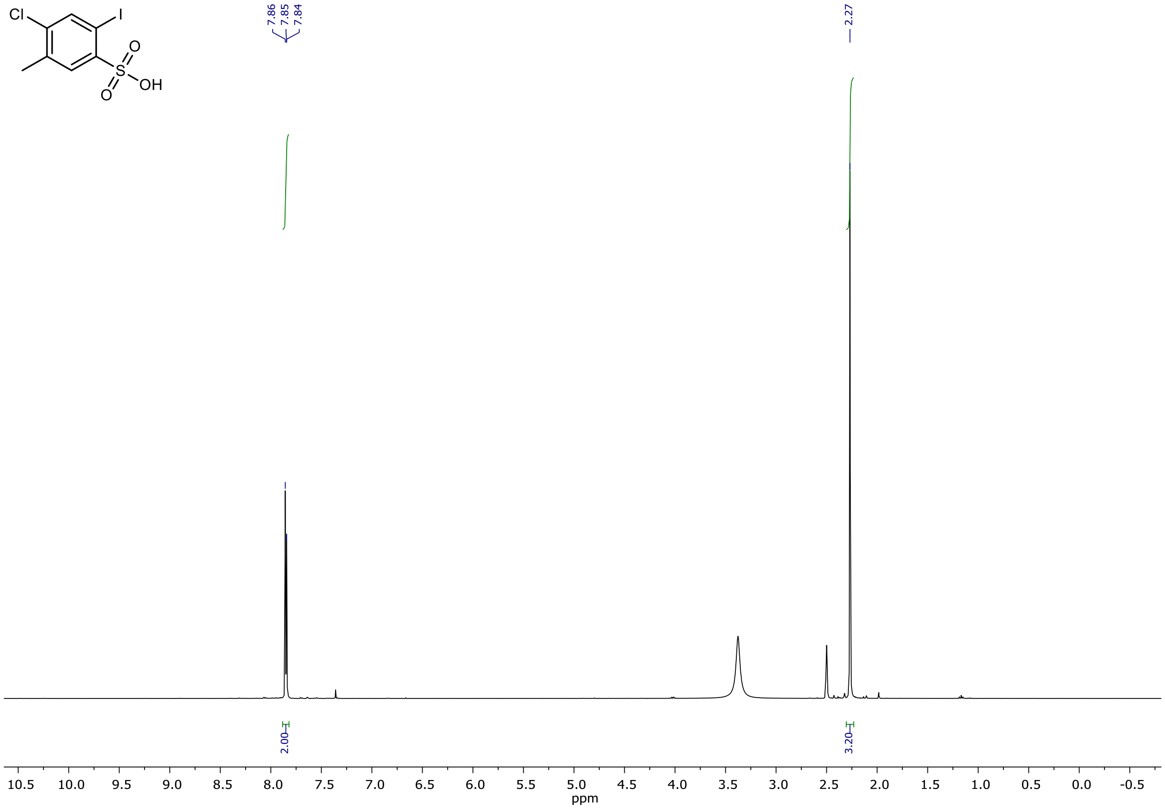

^{13}C NMR (101 MHz, DMSO-d_6)

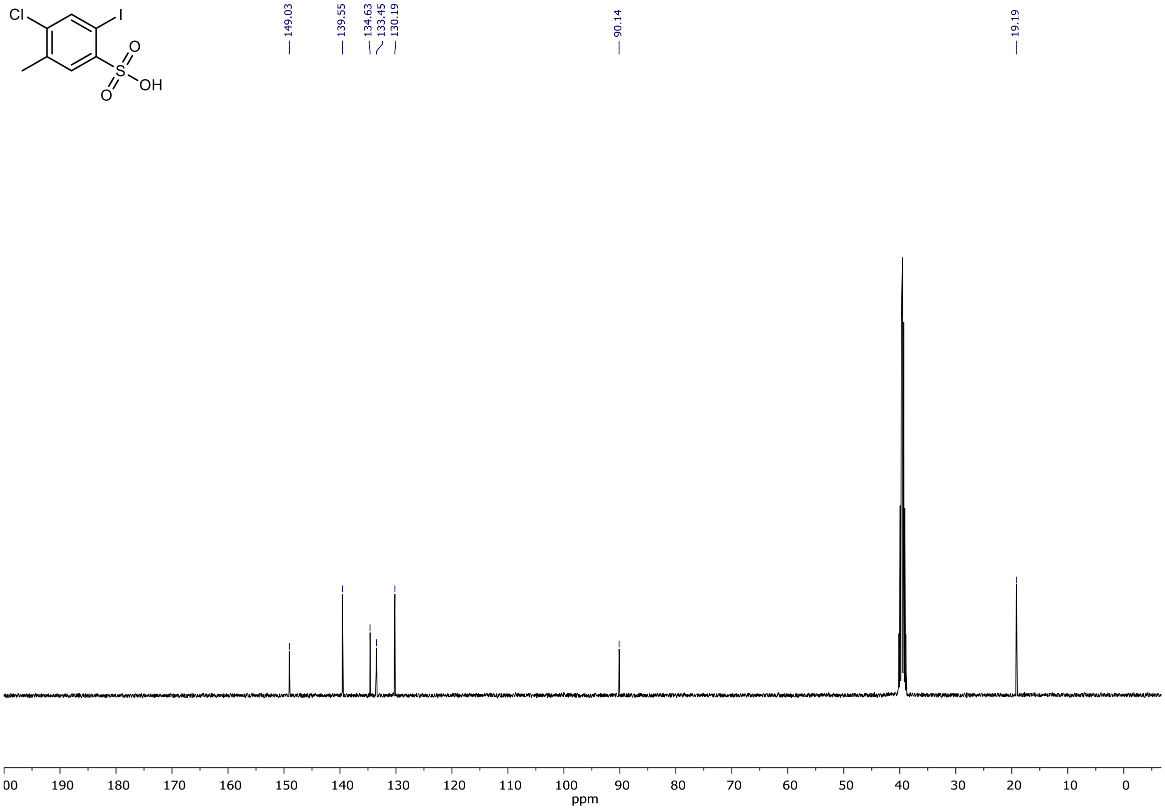

Supplementary Figure 20: NMR Spectra of 4-Chloro-2-iodo-5-methylbenzenesulfonic acid (S-7)

^{1}H NMR (300 MHz, DMSO-d_6)

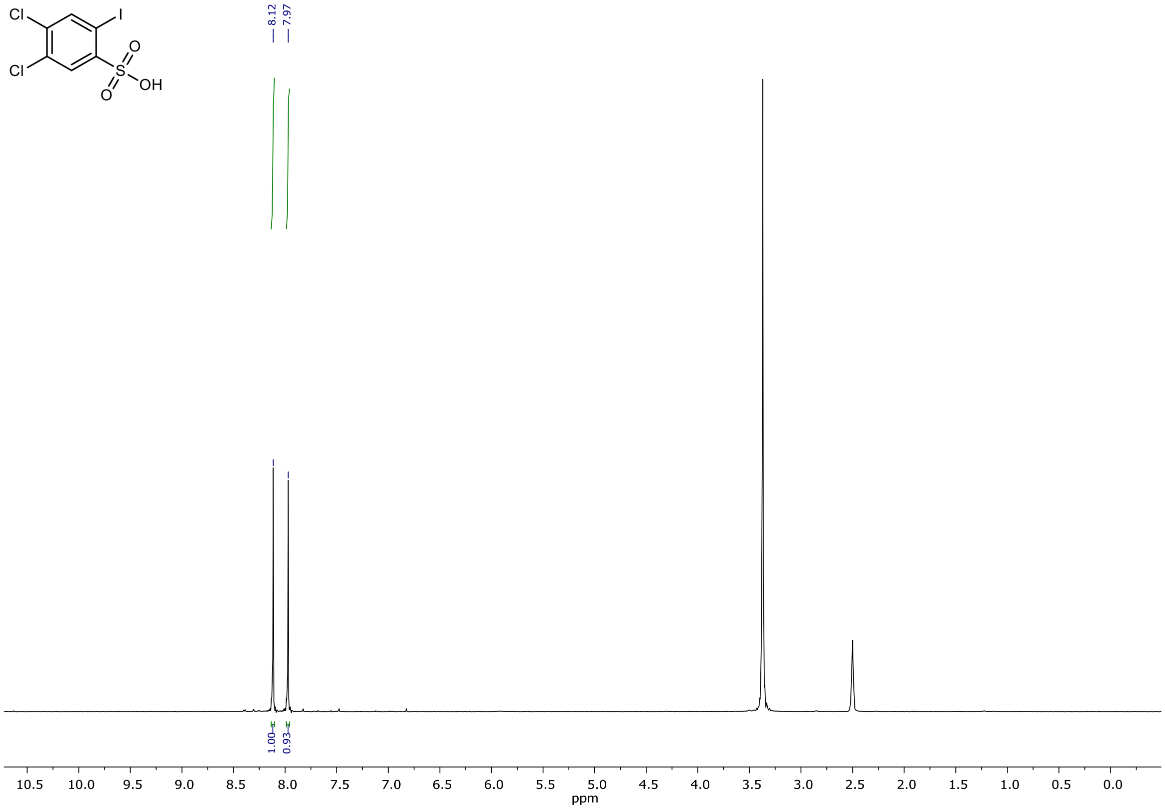

^{13}C NMR (75 MHz, DMSO-d_6)

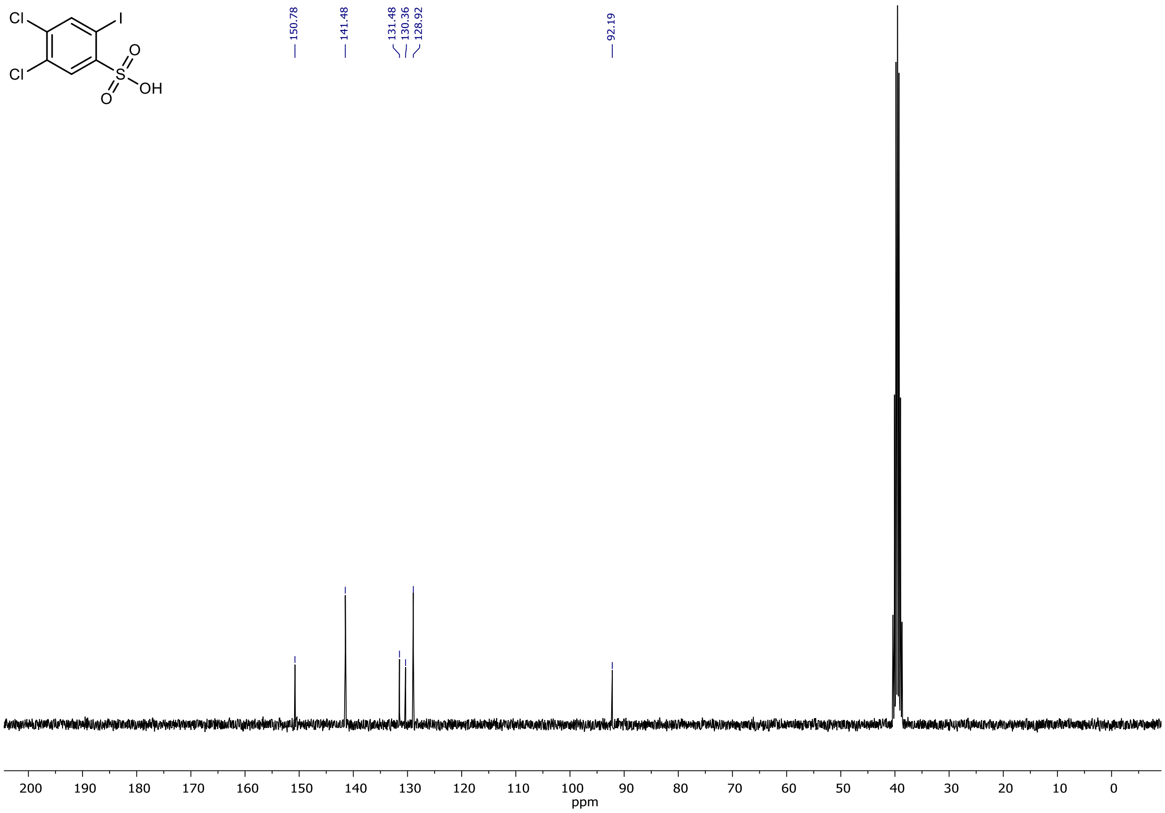

Supplementary Figure 21: NMR Spectra of 4,5-Dichloro-2-iodobenzenesulfonic acid (S-8)

^{1}H NMR (300 MHz, D_2O)

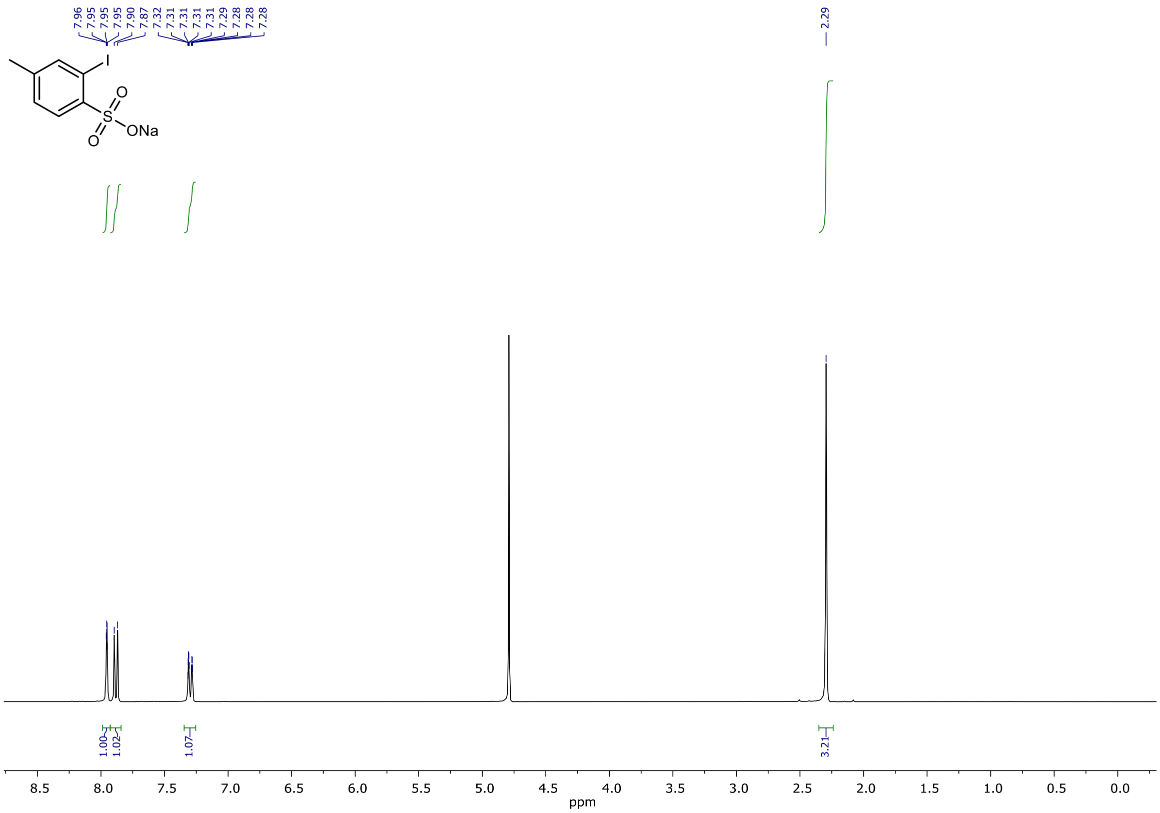

^{13}C NMR (75 MHz, D_2O)

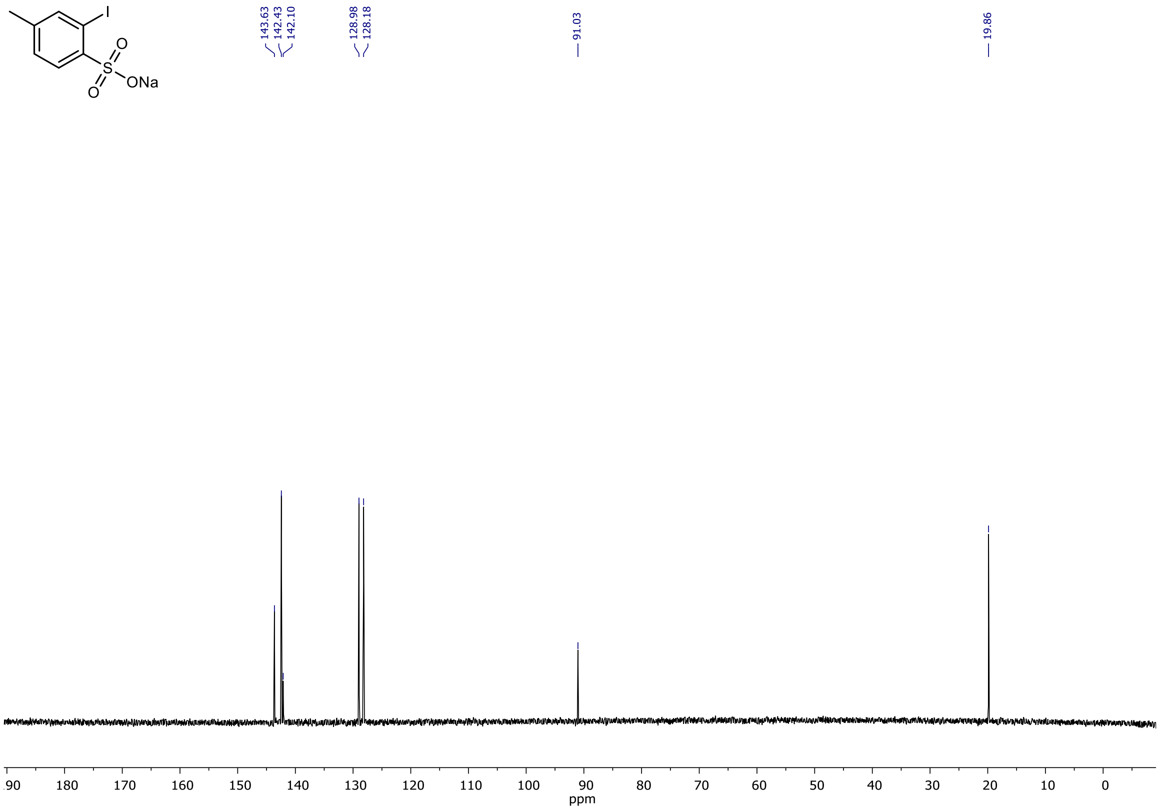

Supplementary Figure 22: NMR Spectra of Sodium 2-iodo-4-methylbenzenesulfonate (S-10)

^{1}H NMR (300 MHz, $CDCl_3$)

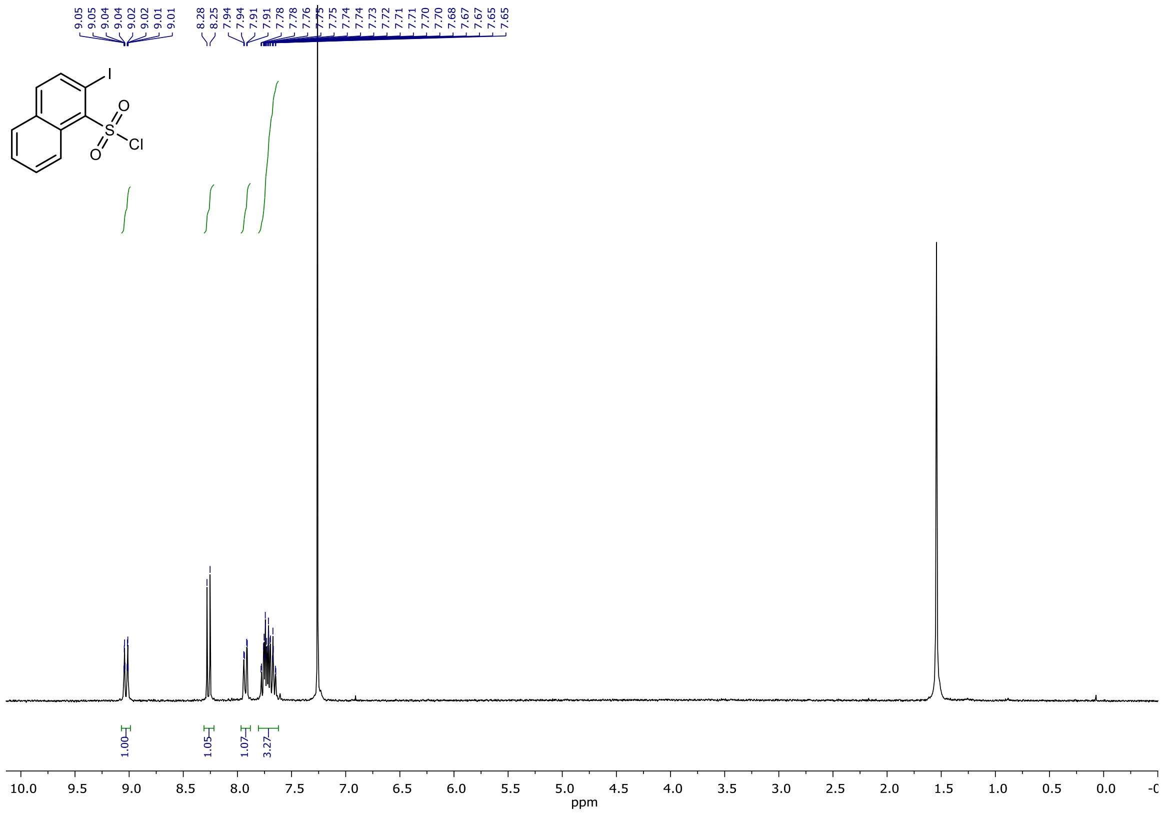

^{13}C NMR (75 MHz, $CDCl_3$)

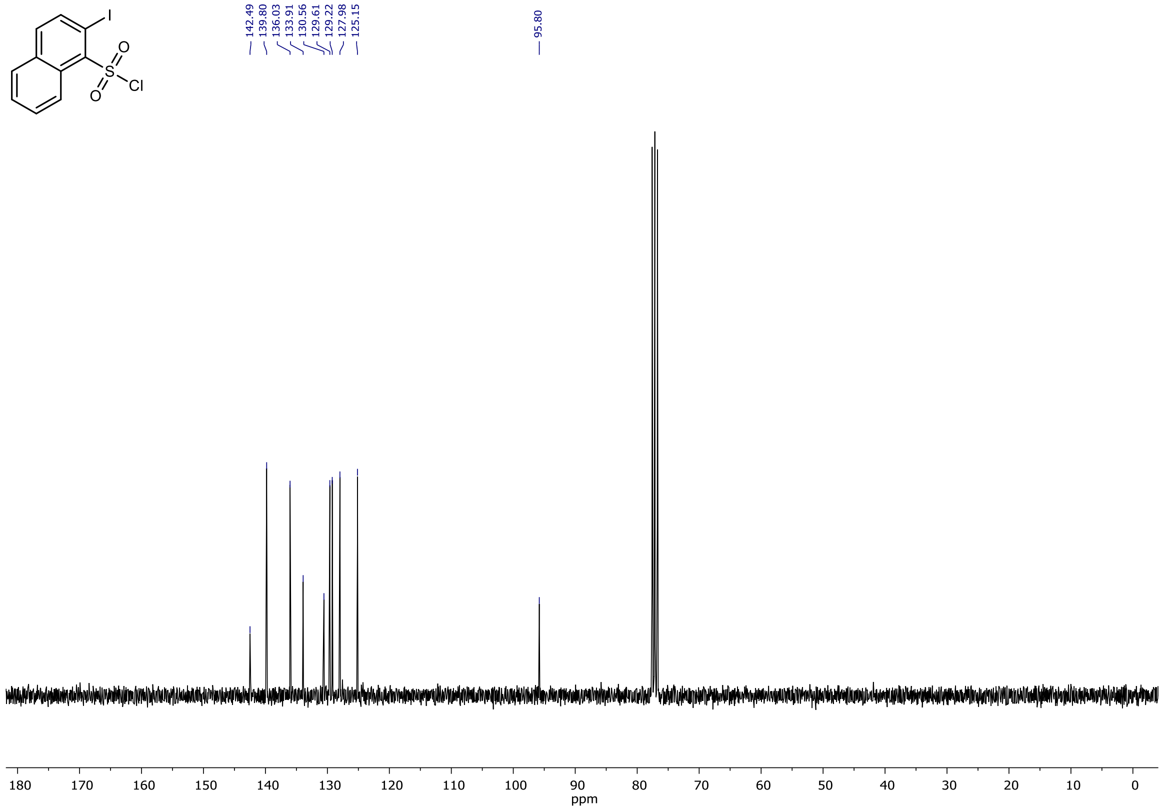

Supplementary Figure 23: NMR Spectra of 2-Iodonaphthalene-1-sulfonyl chloride (S-12)

^1H NMR (300 MHz, $CDCl_3$)

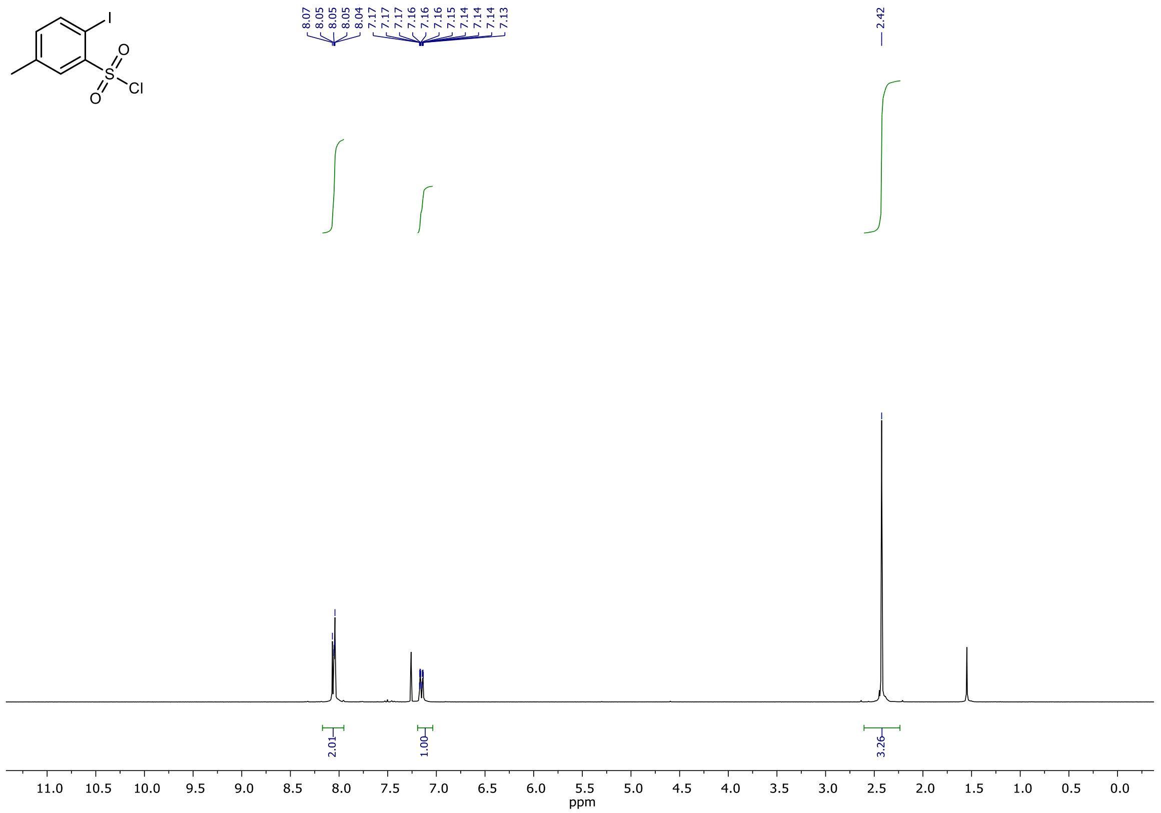

^{13}C NMR (75 MHz, $CDCl_3$)

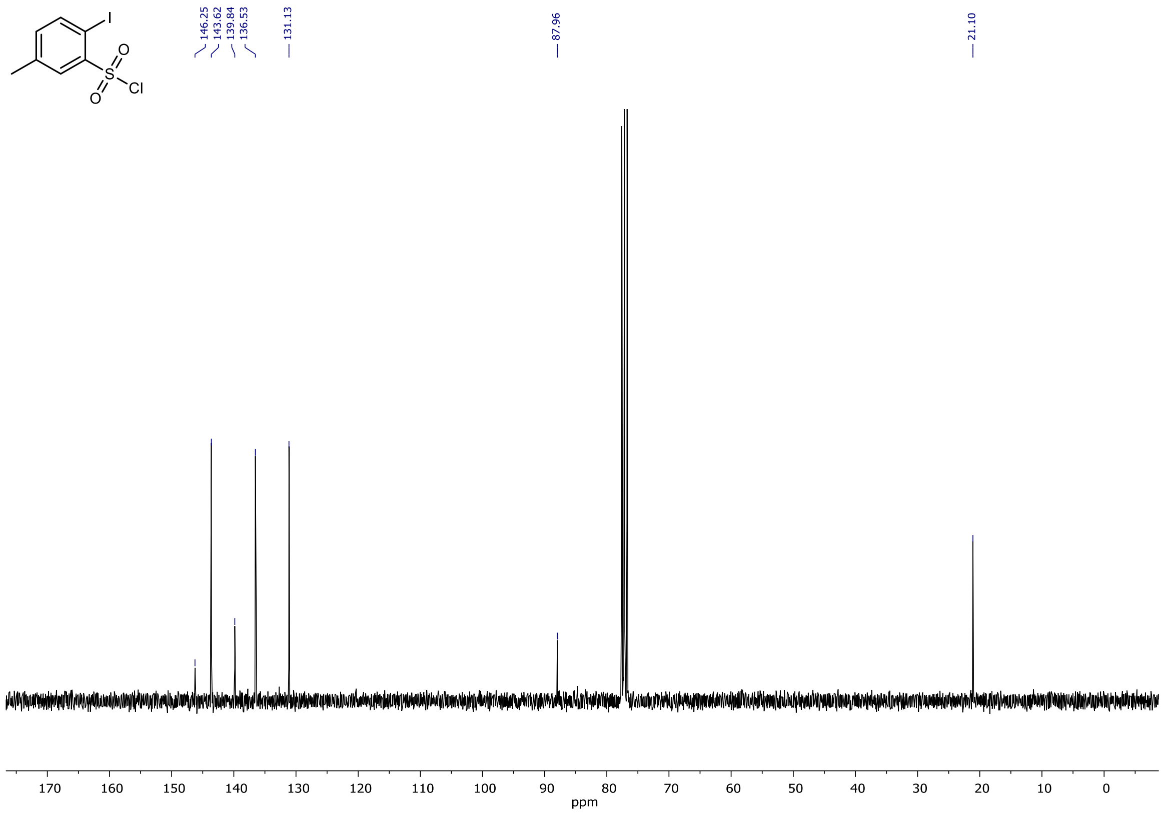

Supplementary Figure 23: NMR Spectra of 2-Iodo-5-methylbenzenesulfonyl chloride (S-13)

^{1}H NMR (300 MHz, $CDCl_3$)

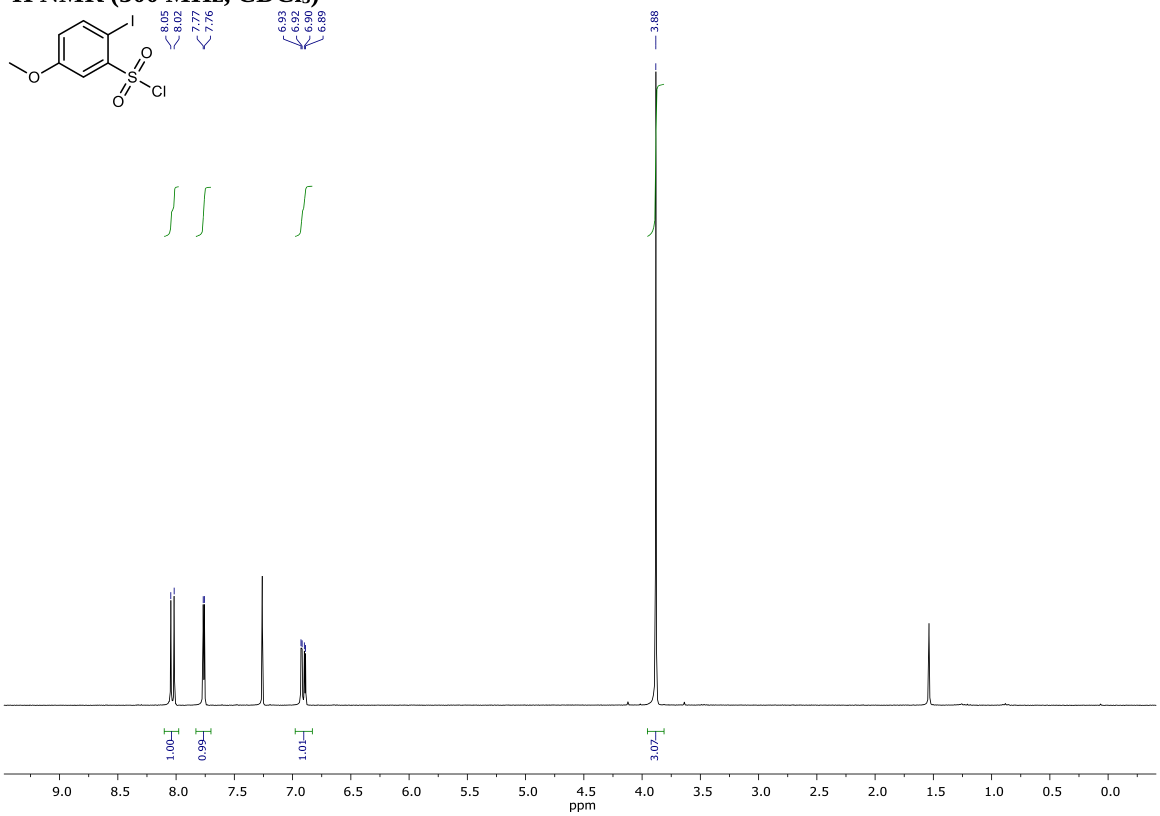

^{13}C NMR (75 MHz, $CDCl_3$)

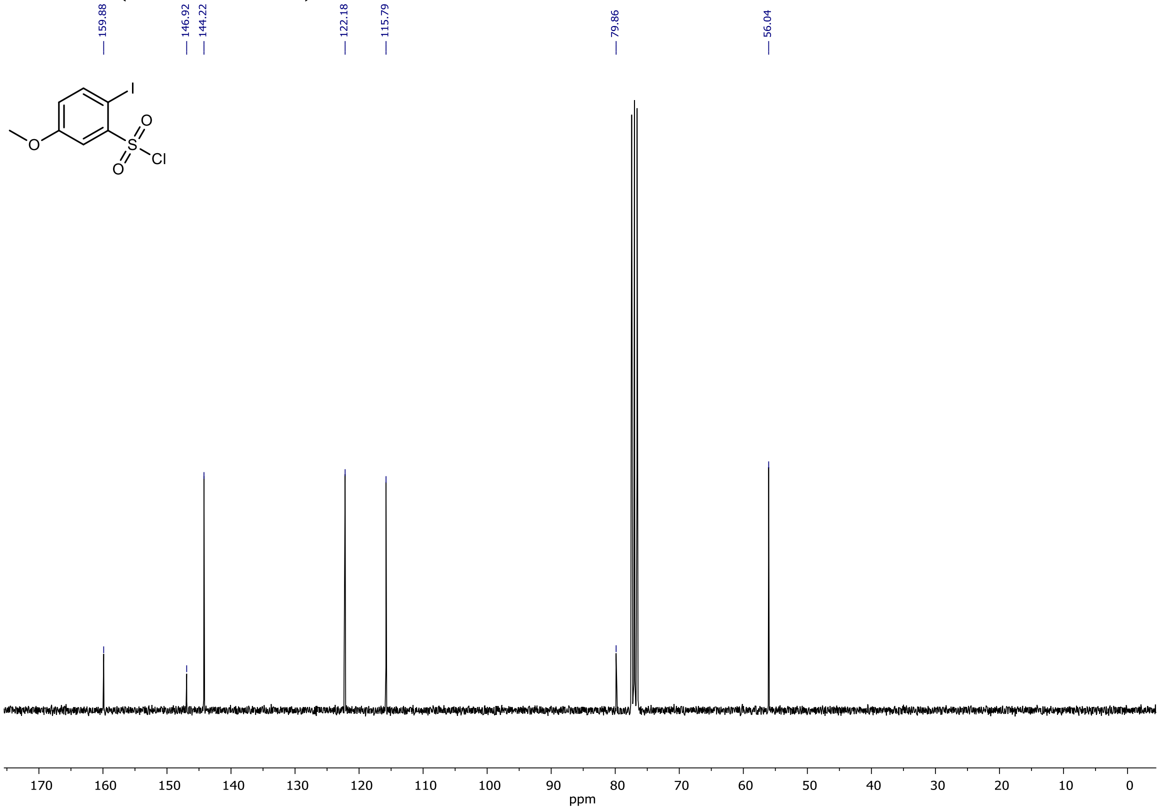

Supplementary Figure 24: NMR Spectra of 2-Iodo-5-methoxybenzenesulfonyl chloride (S-14)

^{1}H NMR (300 MHz, $CDCl_3$)

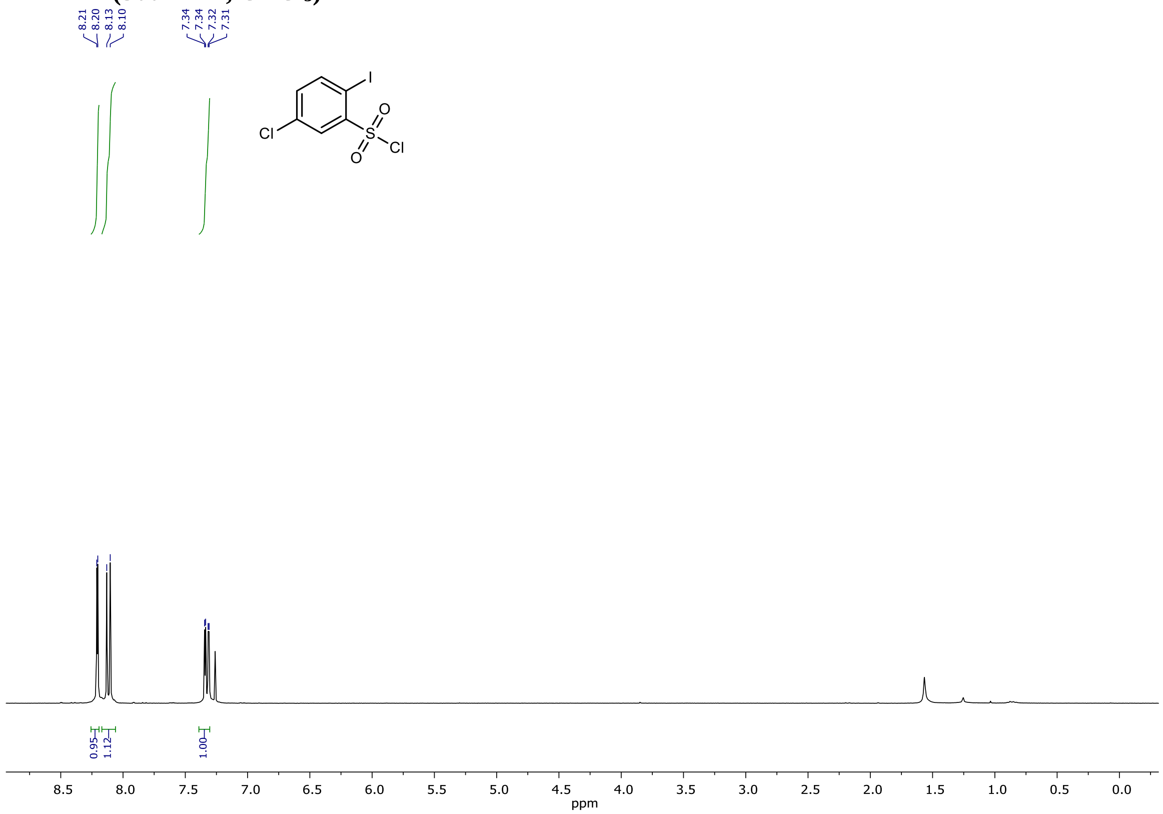

^{13}C NMR (75 MHz, $CDCl_3$)

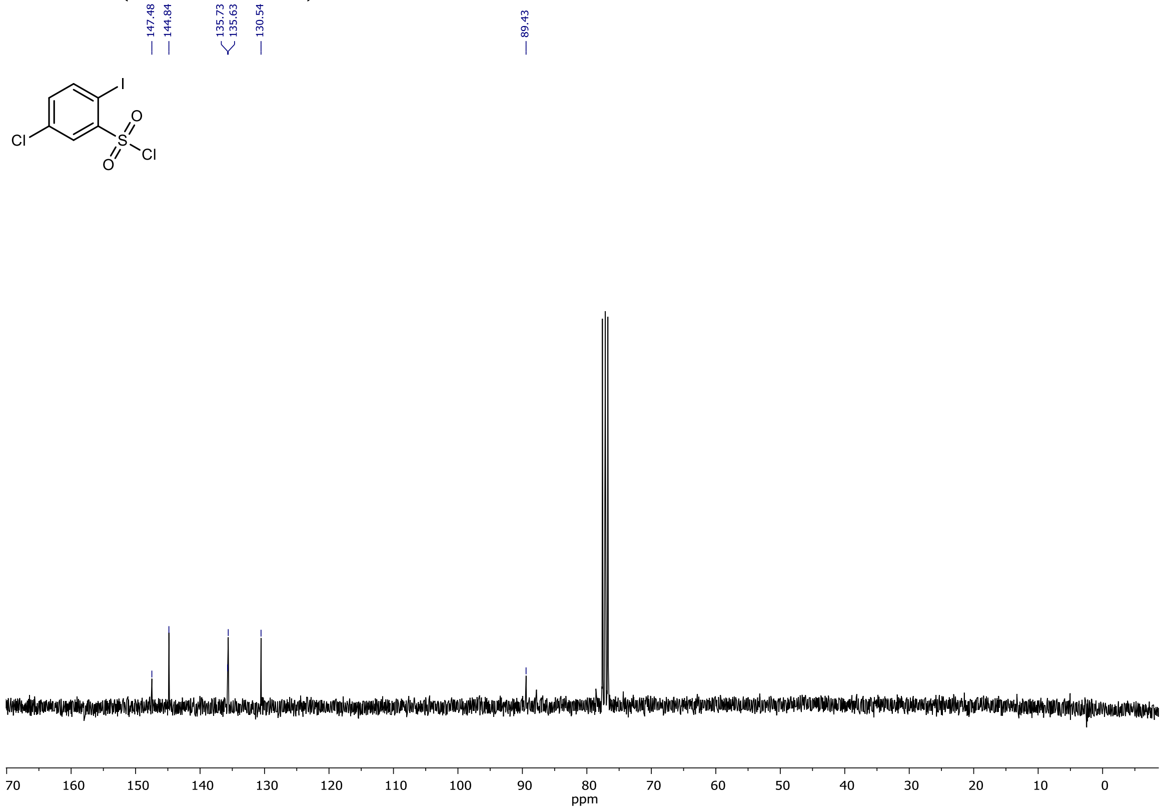

Supplementary Figure 25: NMR Spectra of 5-Chloro-2-iodobenzenesulfonyl chloride (S-15)

^{1}H NMR (300 MHz, CDCl$_3$)

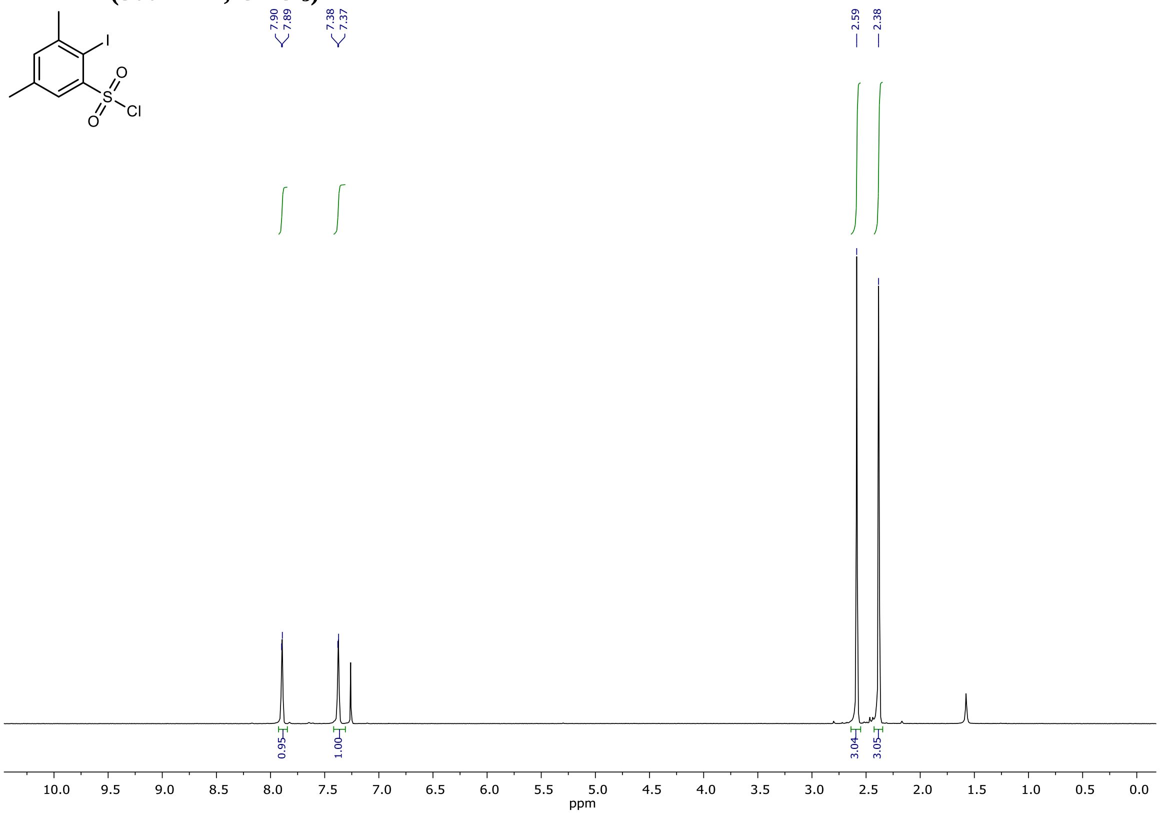

^{13}C NMR (75 MHz, CDCl$_3$)

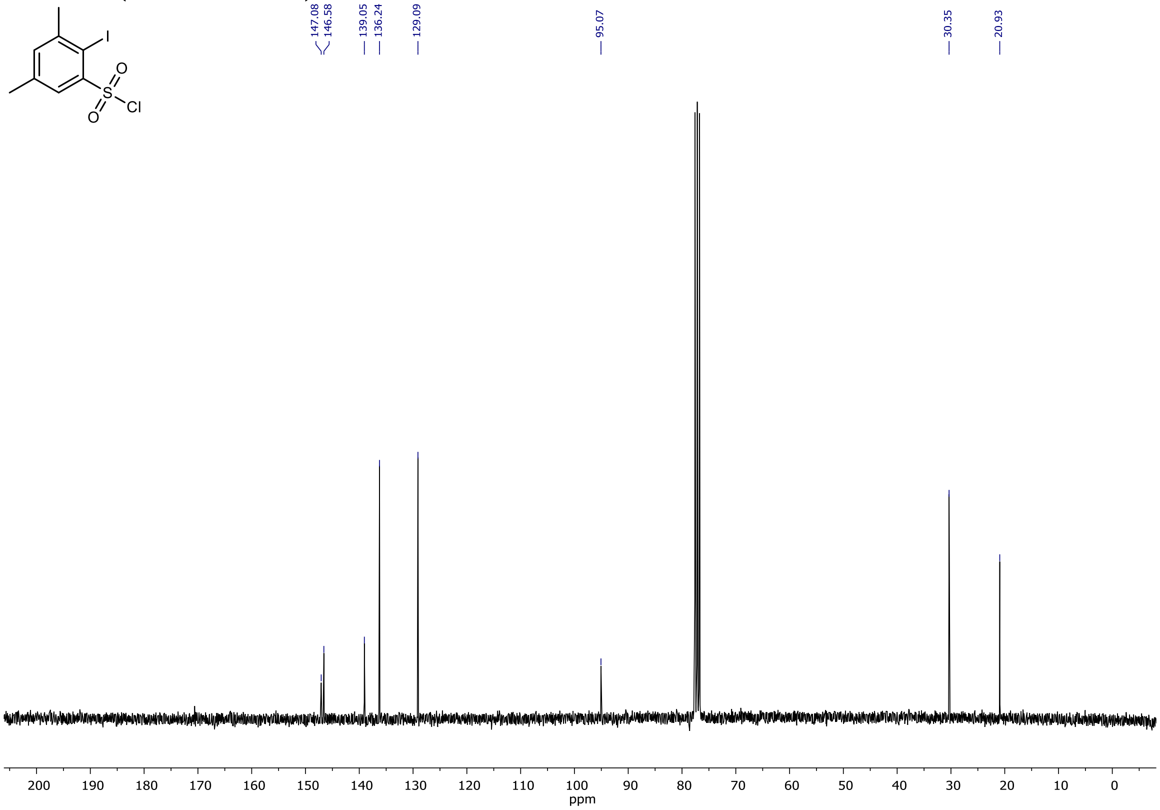

Supplementary Figure 26: NMR Spectra of 2-Iodo-3,5-dimethylbenzenesulfonyl chloride (S-16)

^{1}H NMR (300 MHz, $CDCl_3$)

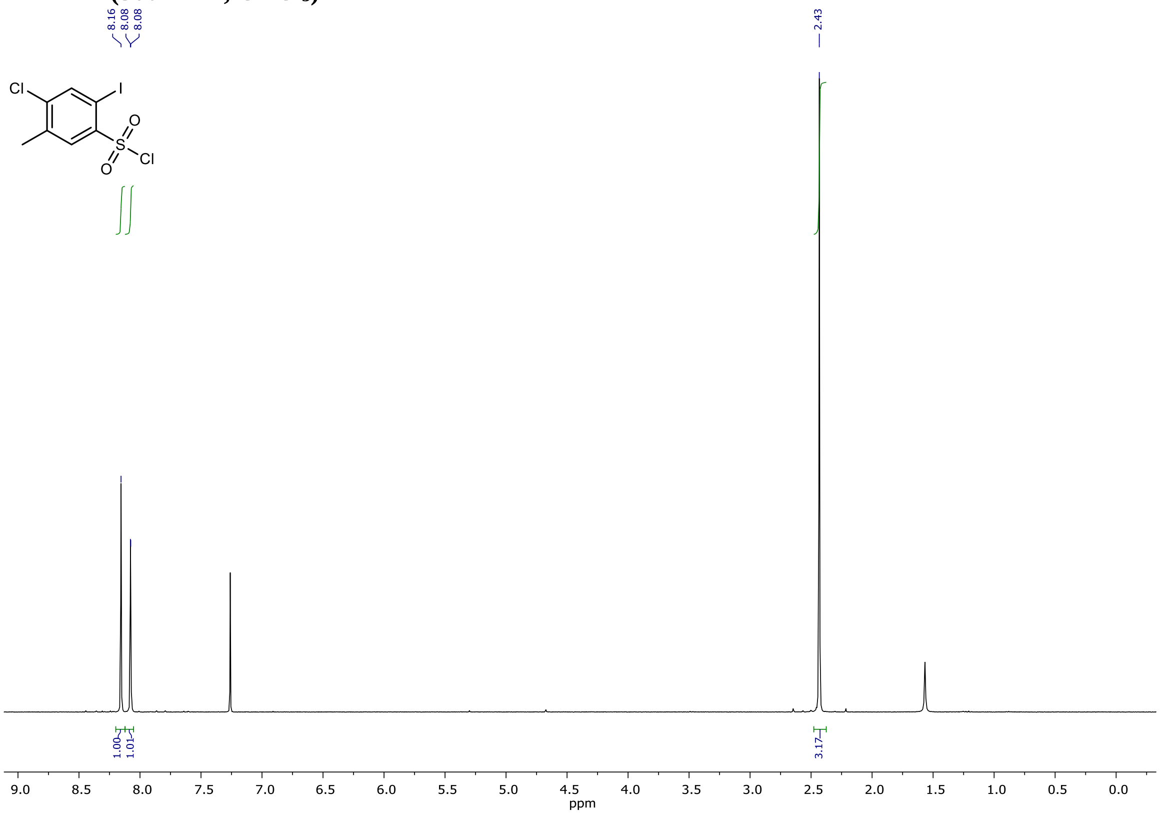

^{13}C NMR (75 MHz, $CDCl_3$)

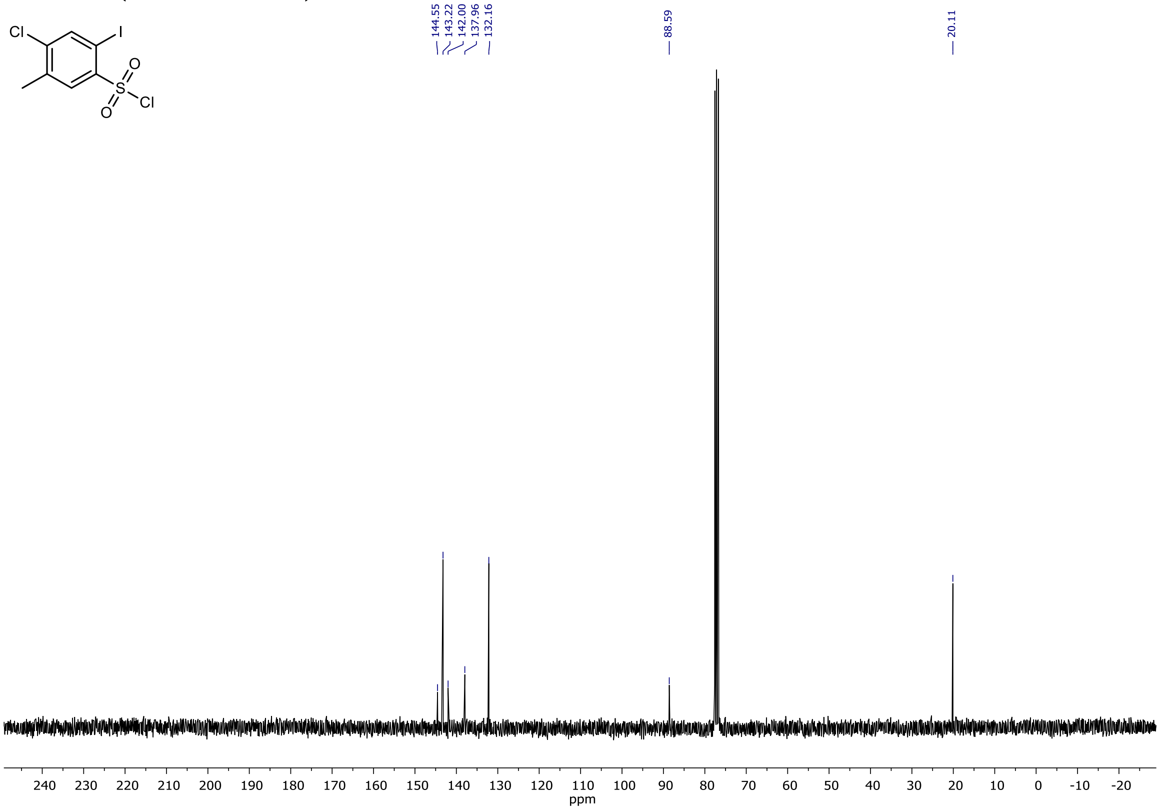

Supplementary Figure 27: NMR Spectra of 4-Chloro-2-iodo-5-methylbenzenesulfonyl chloride (S-17)

1H NMR (400 MHz, $CDCl_3$)

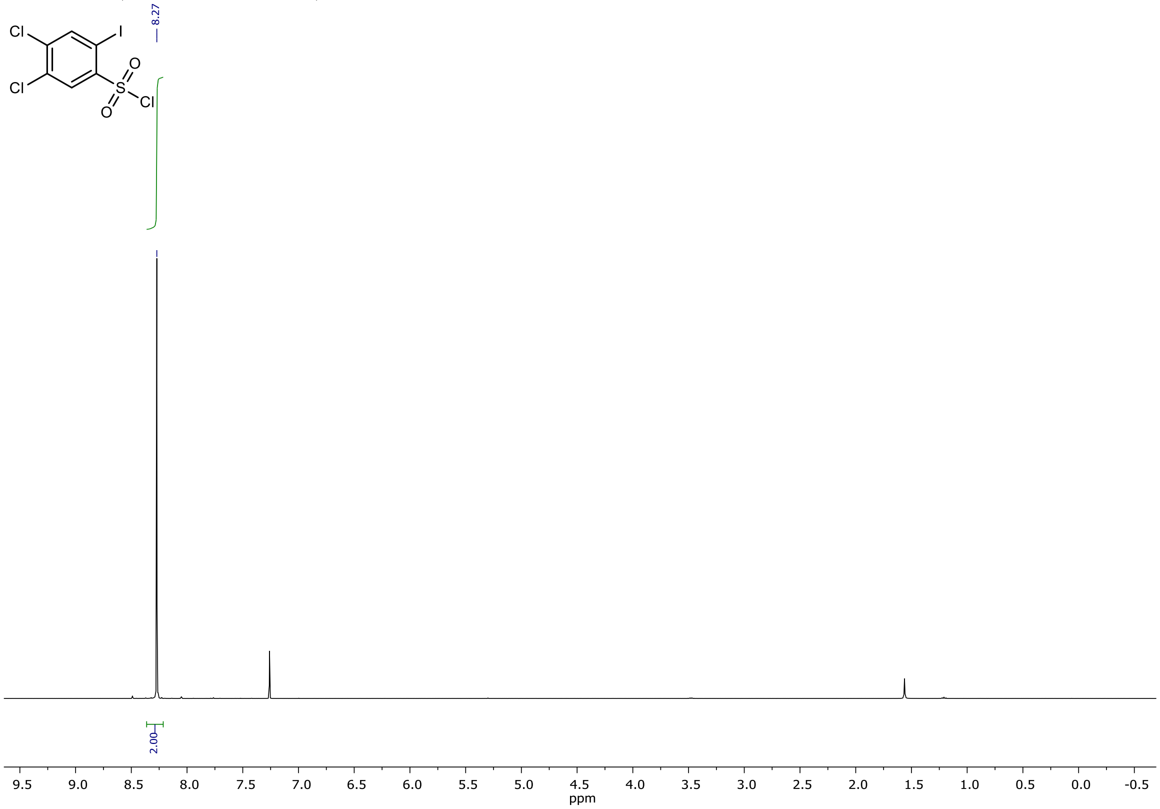

^{13}C NMR (101 MHz, $CDCl_3$)

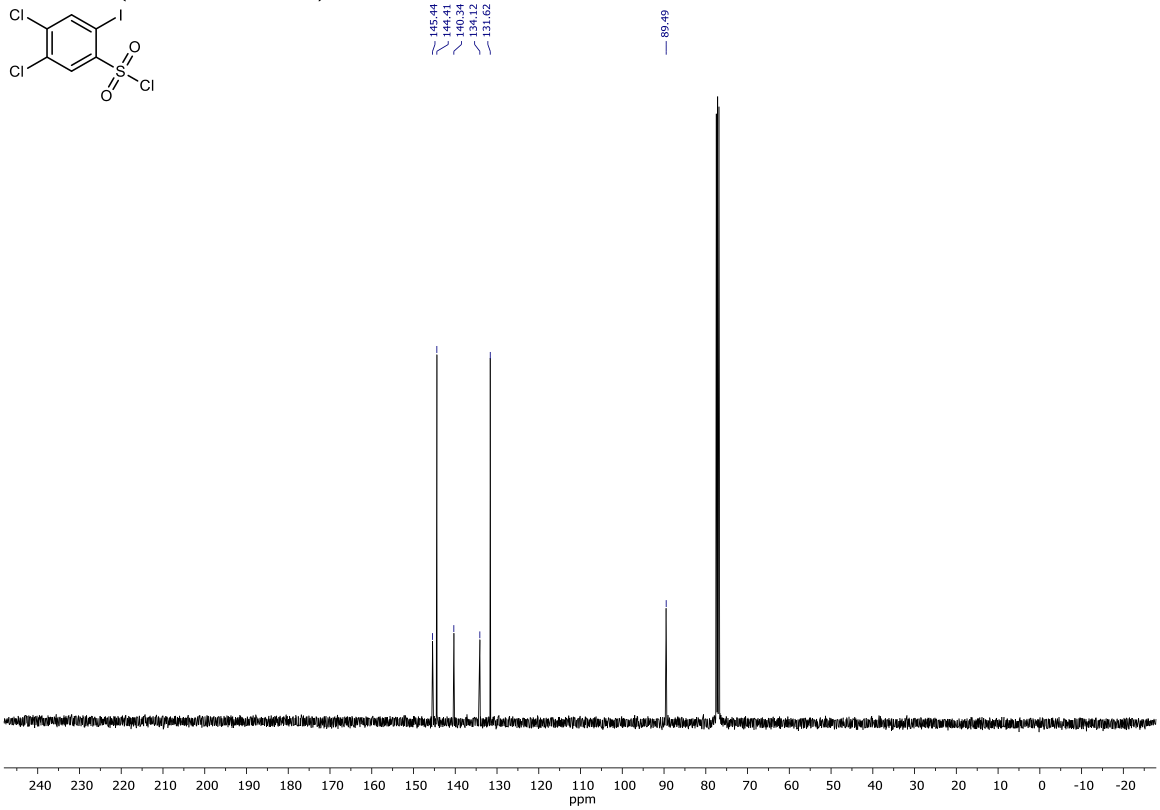

Supplementary Figure 28: NMR Spectra of 4,5-Dichloro-2-iodobenzenesulfonyl chloride (S-18)

^{1}H NMR (300 MHz, $CDCl_3$)

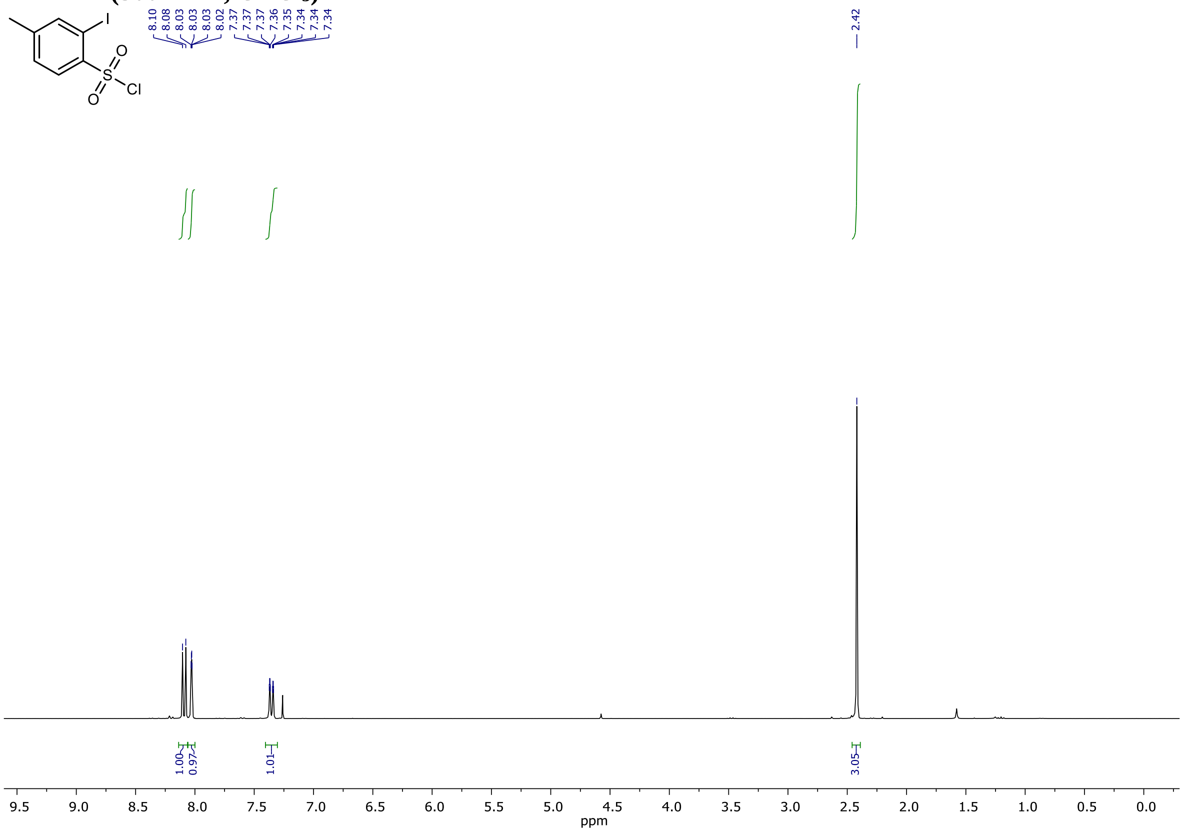

^{13}C NMR (75 MHz, $CDCl_3$)

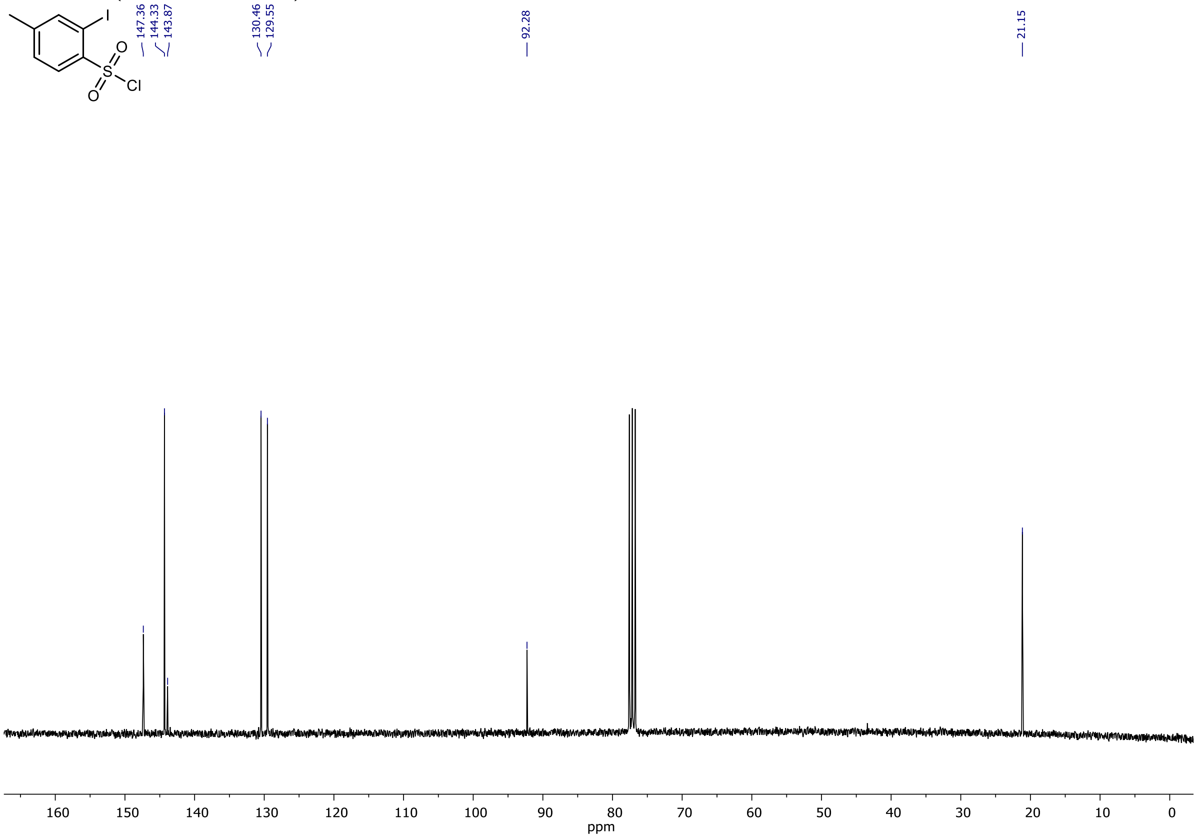

Supplementary Figure 29: NMR Spectra of 2-Iodo-4-methylbenzenesulfonyl chloride (S-19)

^{1}H NMR (300 MHz, $CDCl_3$)

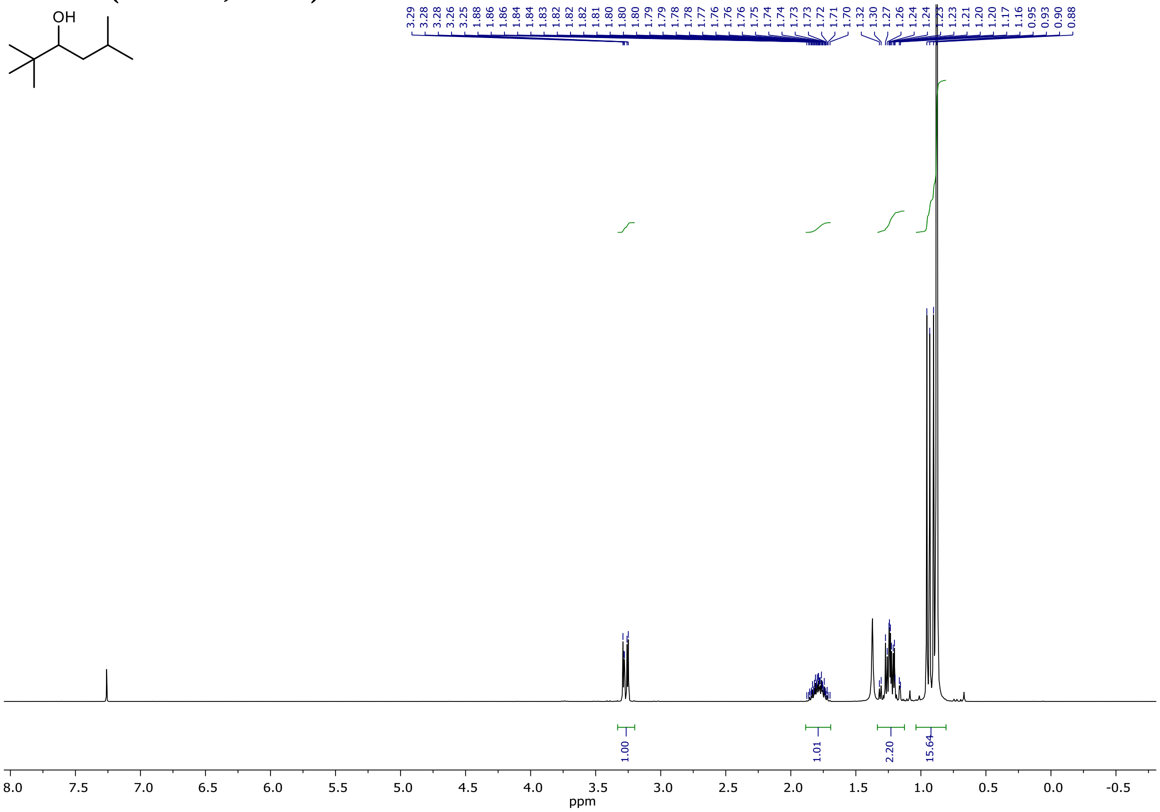

^{13}C NMR (75 MHz, $CDCl_3$)

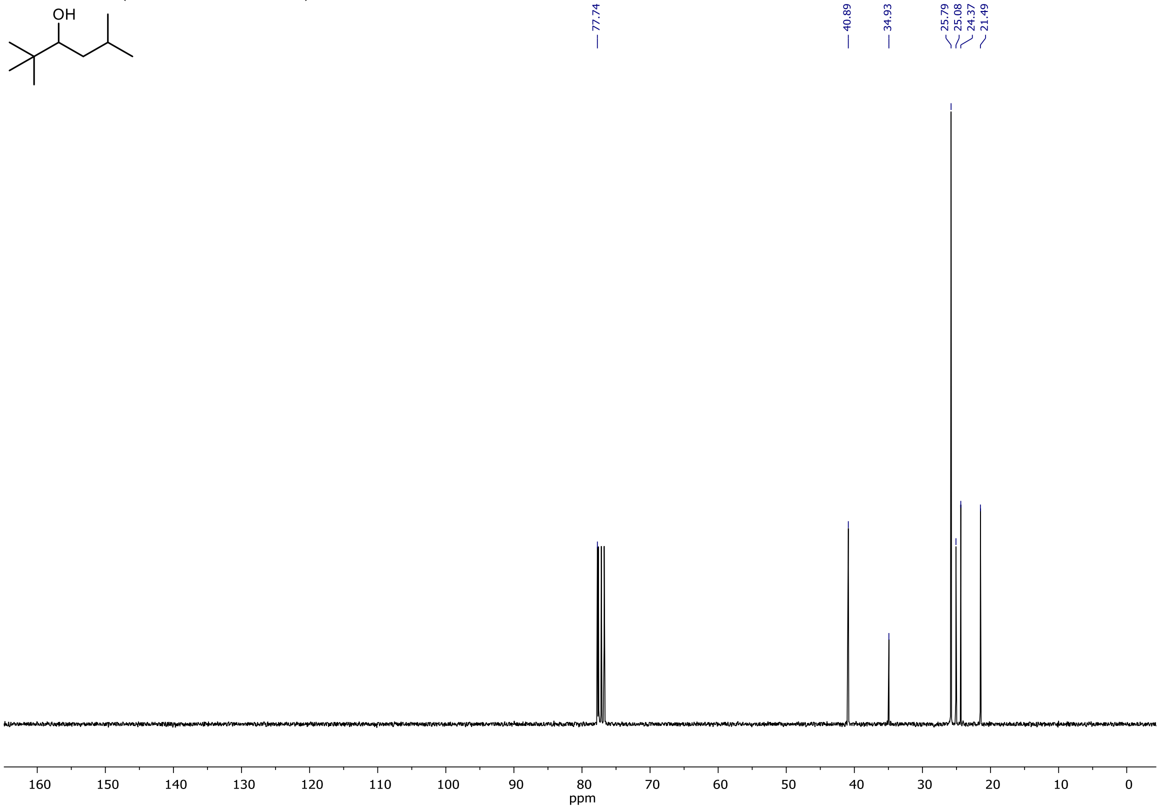

Supplementary Figure 30: NMR Spectra of 2,2,5-Trimethylhexan-3-ol (1a)

^{1}H NMR (400 MHz, CDCl$_3$)

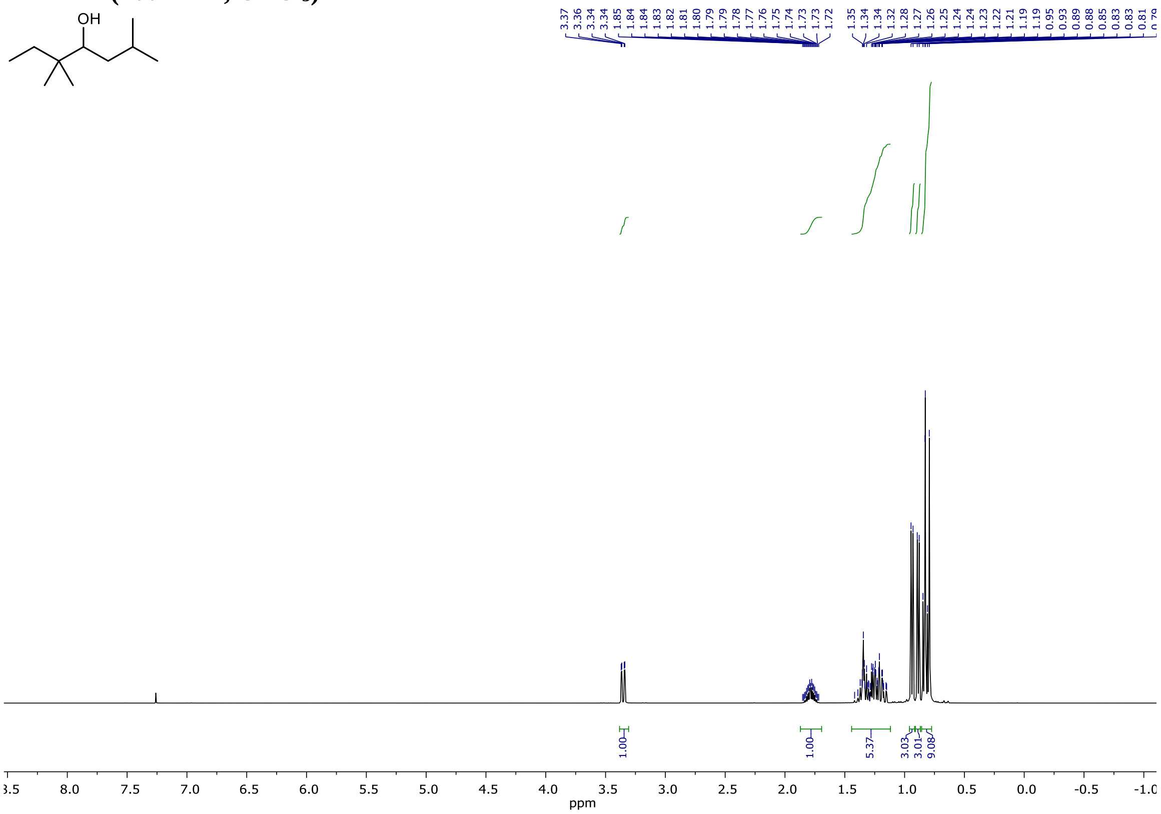

^{13}C NMR (101 MHz, CDCl$_3$)

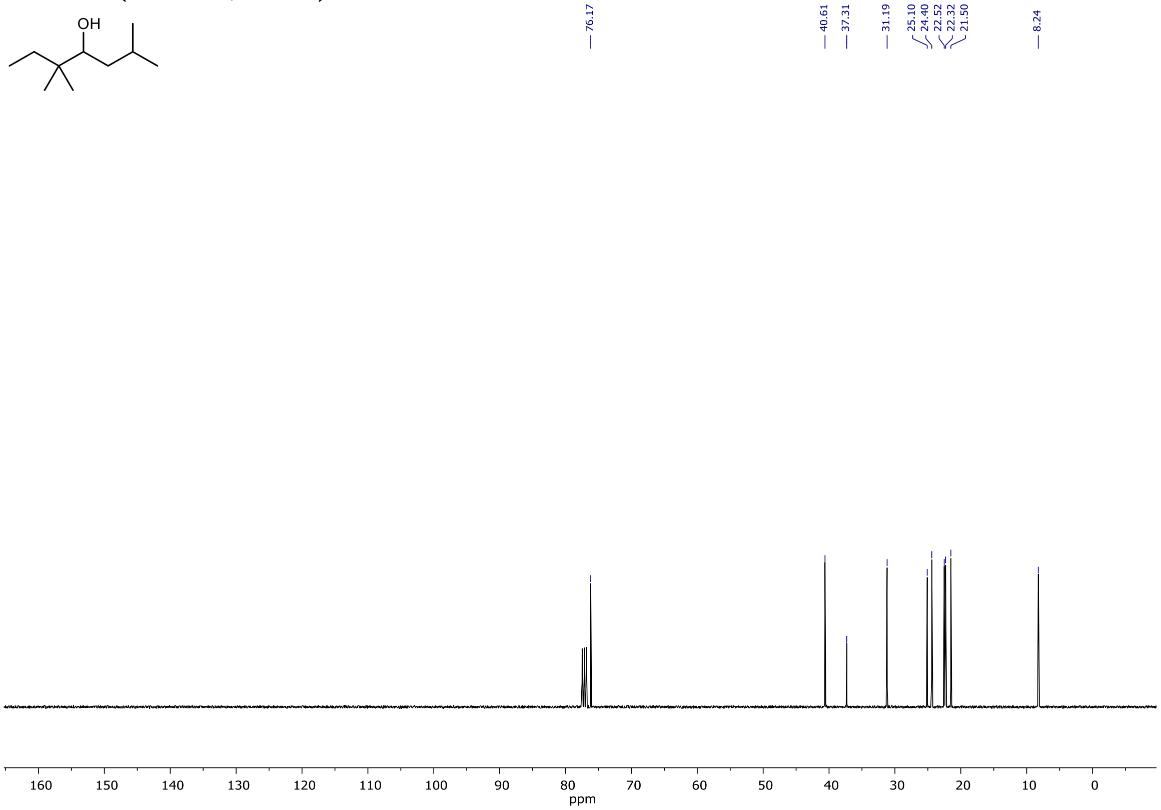

Supplementary Figure 31: NMR Spectra of 2,5,5-Trimethylheptan-4-ol (1b)

^{1}H NMR (300 MHz, $CDCl_3$)

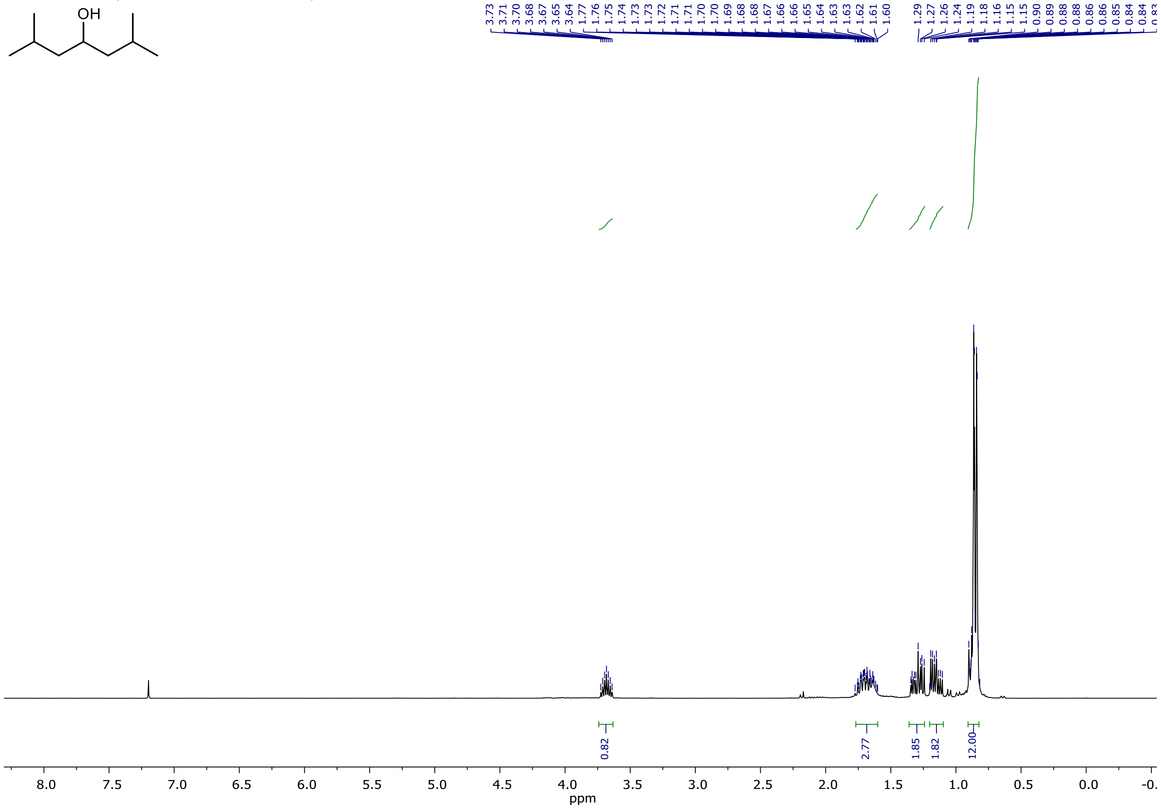

^{13}C NMR (75 MHz, $CDCl_3$)

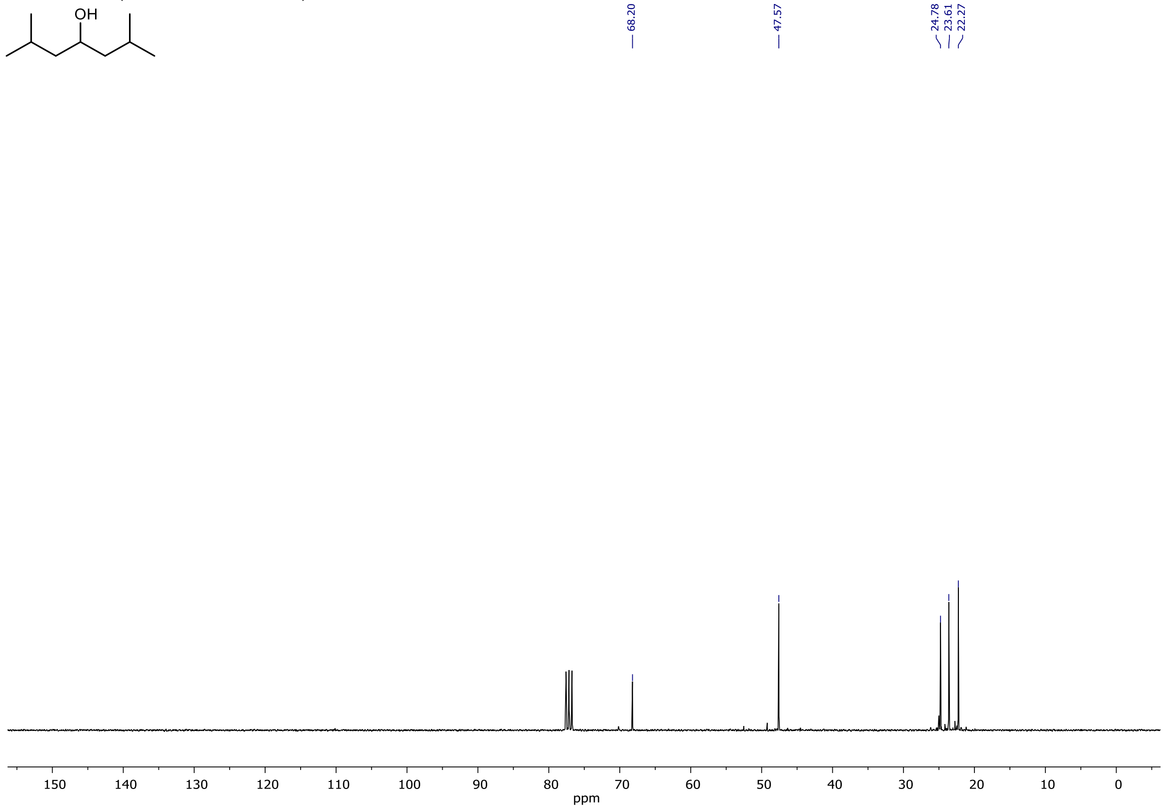

Supplementary Figure 32: NMR Spectra of 2,6-Dimethylheptan-4-ol (1d)

^{1}H NMR (300 MHz, CDCl$_3$)

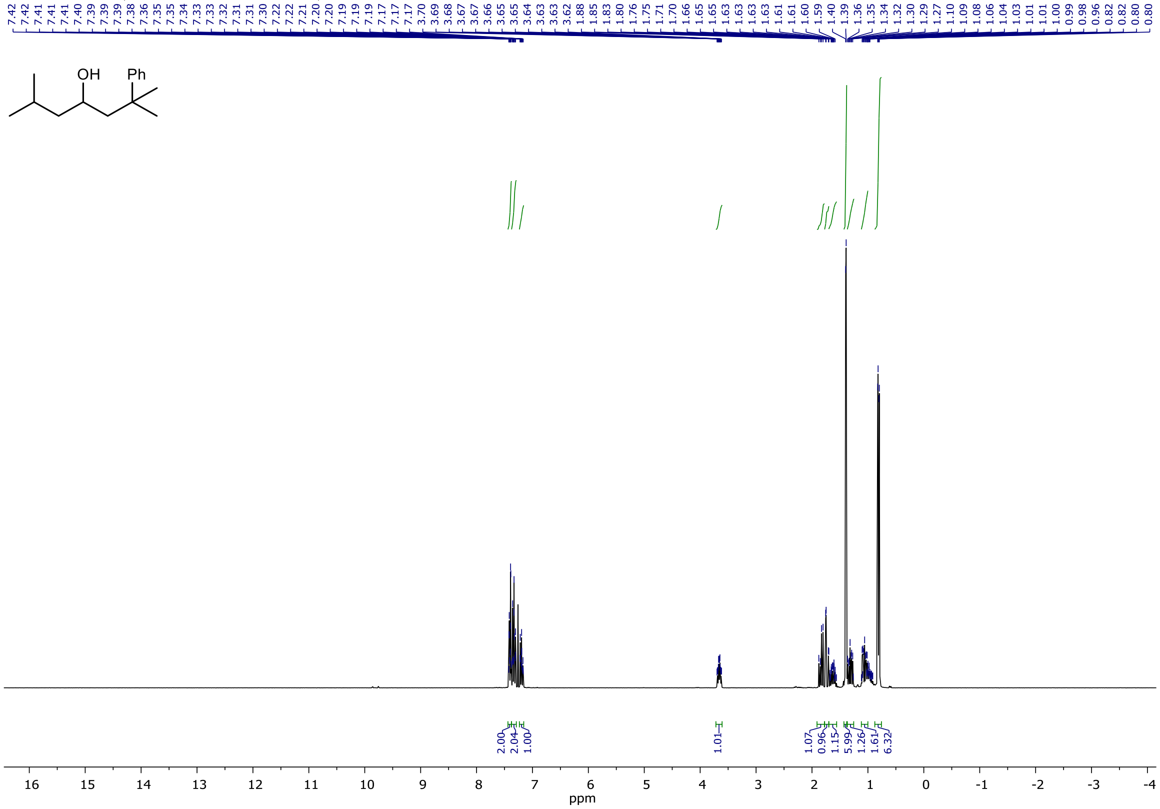

^{13}C NMR (75 MHz, CDCl$_3$)

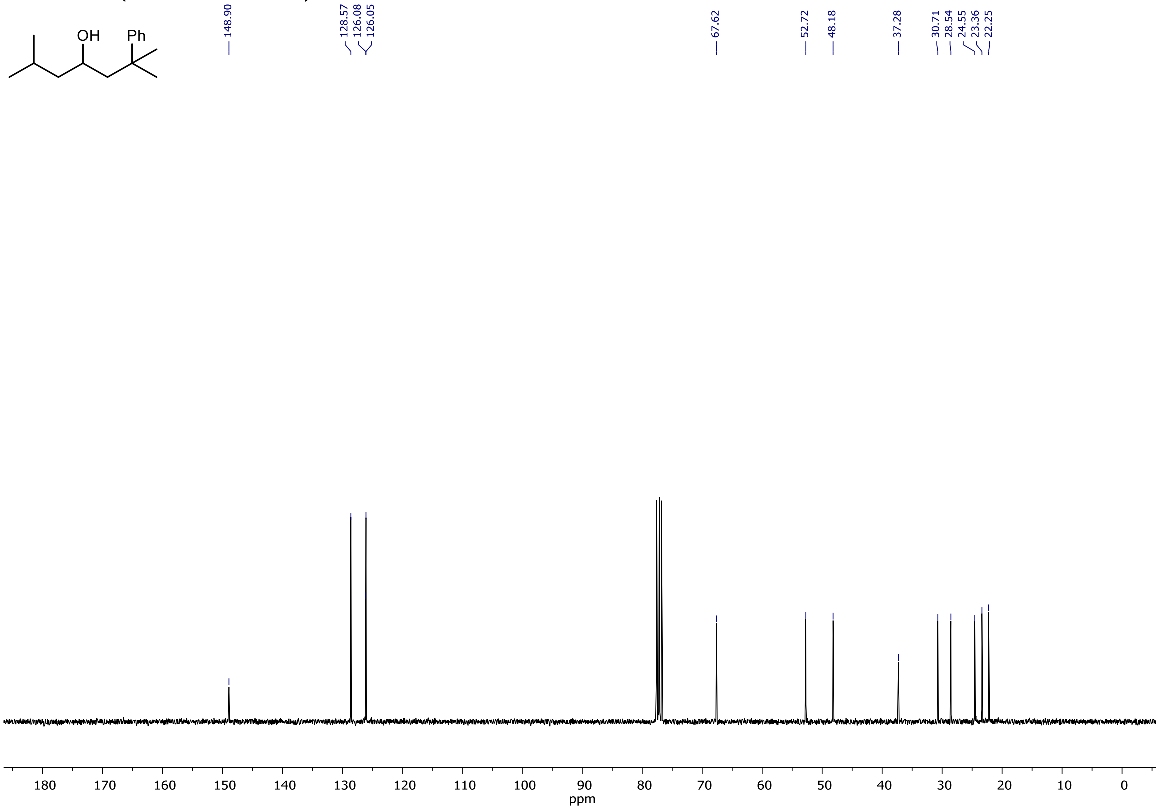

Supplementary Figure 33: NMR Spectra of 2,6-Dimethyl-2-phenylheptan-4-ol (1e)

^{1}H NMR (300 MHz, $CDCl_3$)

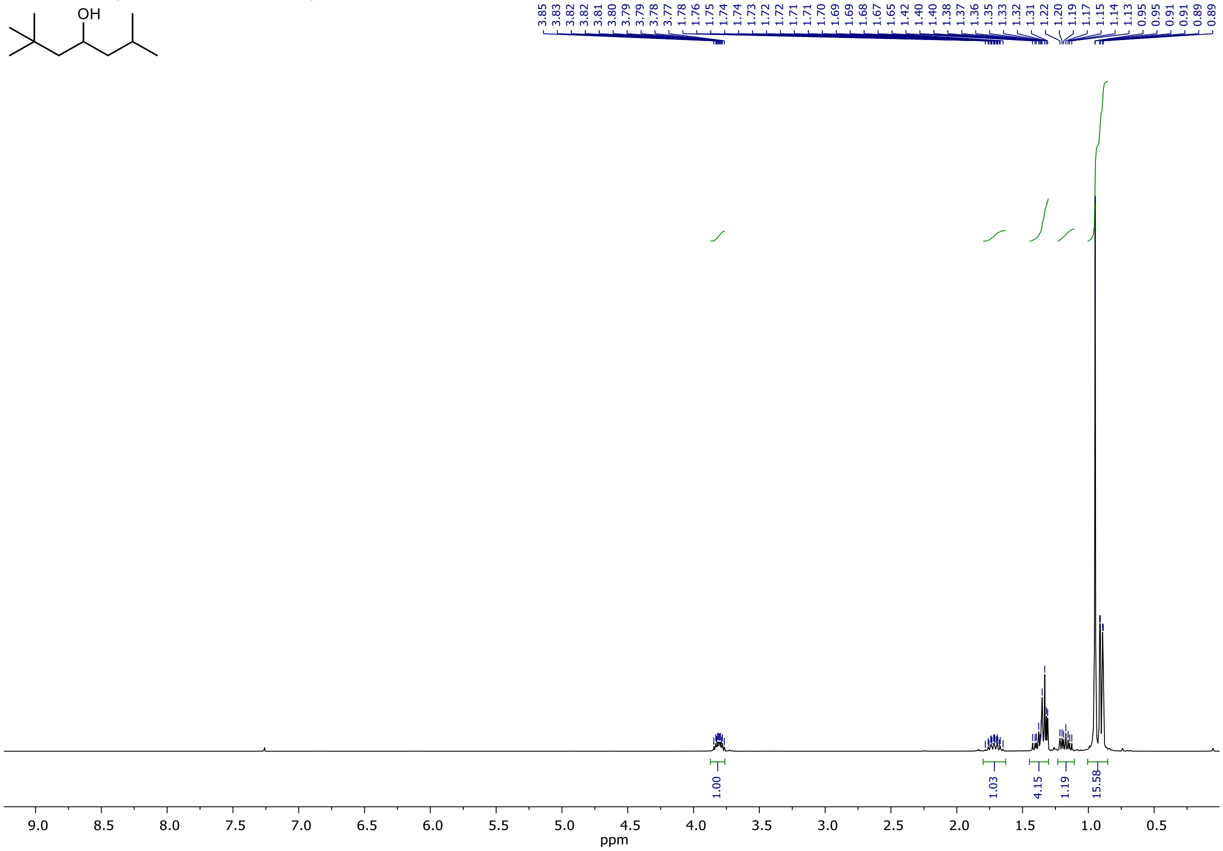

^{13}C NMR (75 MHz, $CDCl_3$)

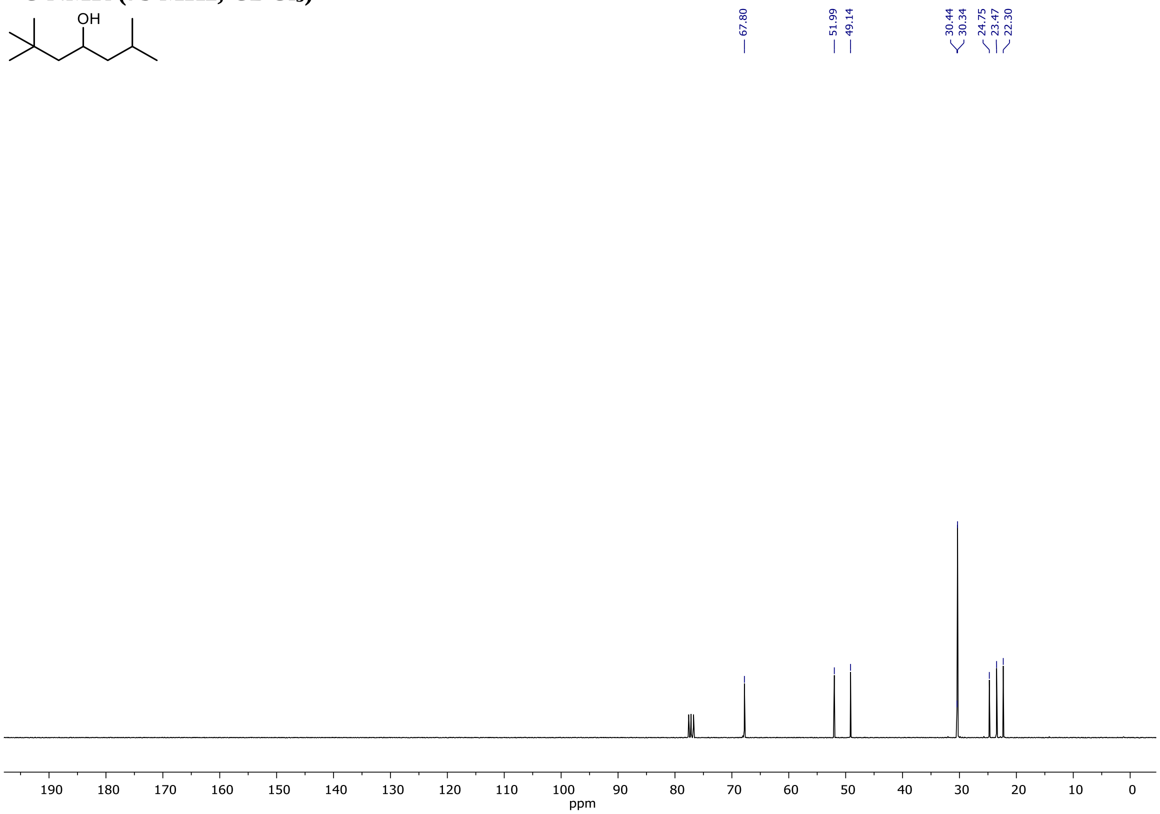

Supplementary Figure 34: NMR Spectra of 2,2,6-Trimethylheptan-4-ol (1f)

^{1}H NMR (400 MHz, $CDCl_3$)

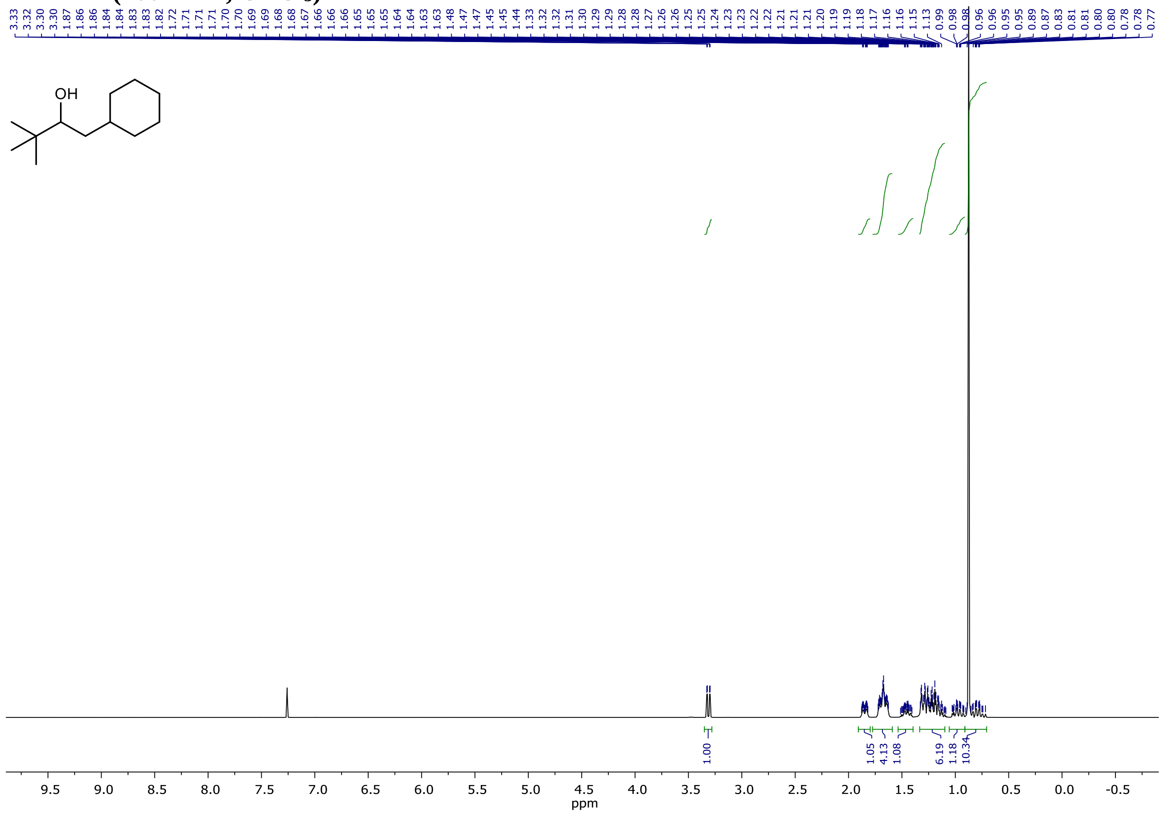

^{13}C NMR (101 MHz, $CDCl_3$)

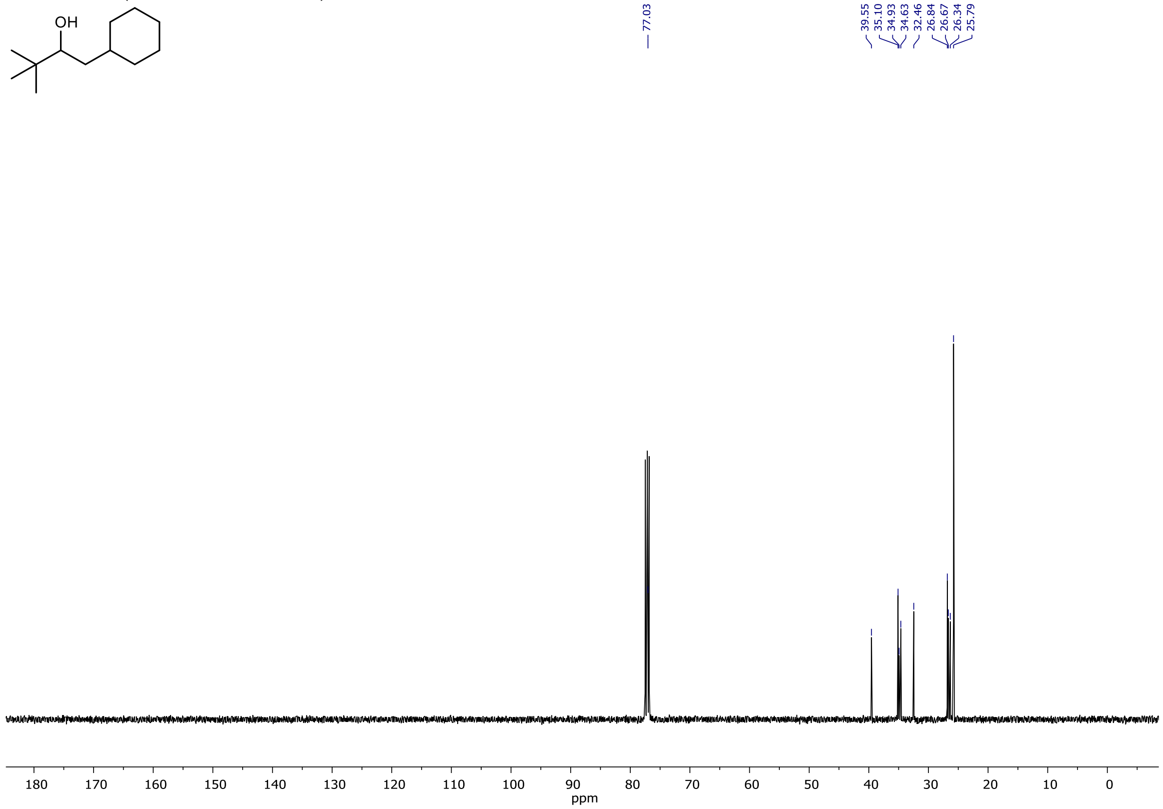

Supplementary Figure 35: NMR Spectra of 1-Cyclohexyl-3,3-dimethylbutan-2-ol (1g)

^{1}H NMR (300 MHz, $CDCl_3$)

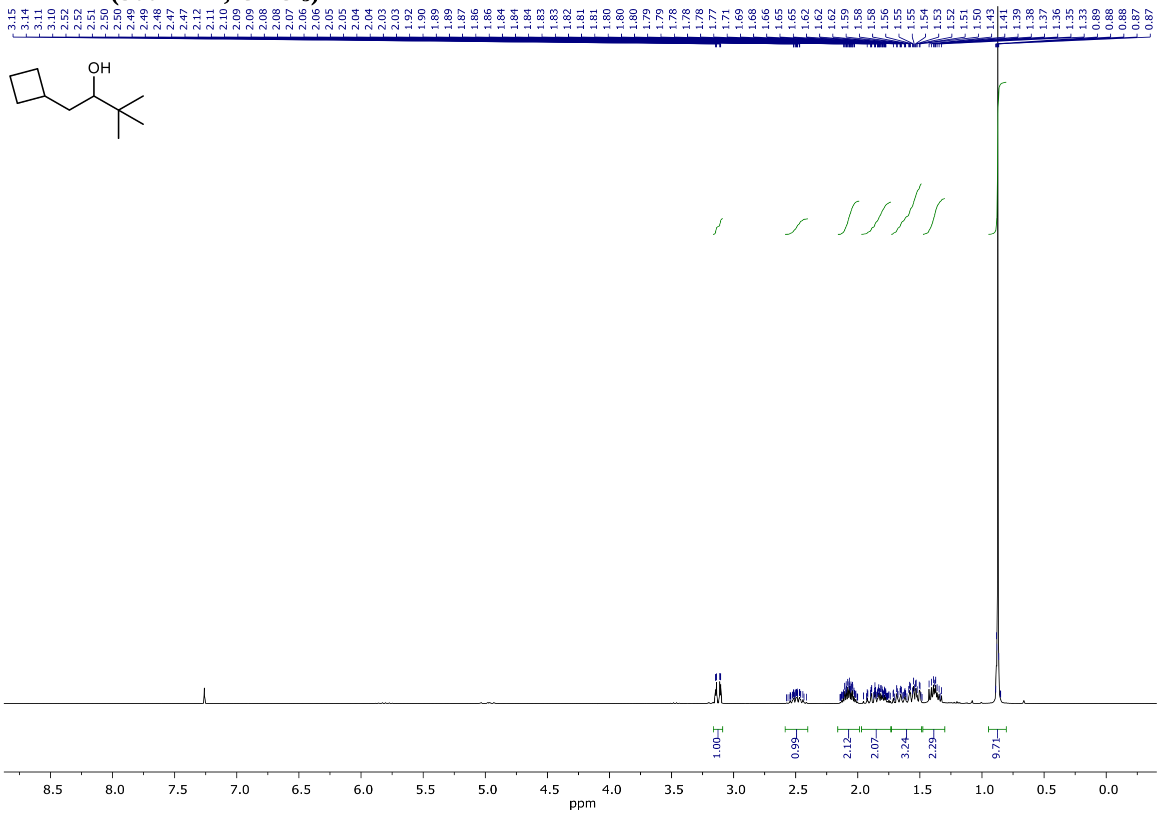

^{13}C NMR (75 MHz, $CDCl_3$)

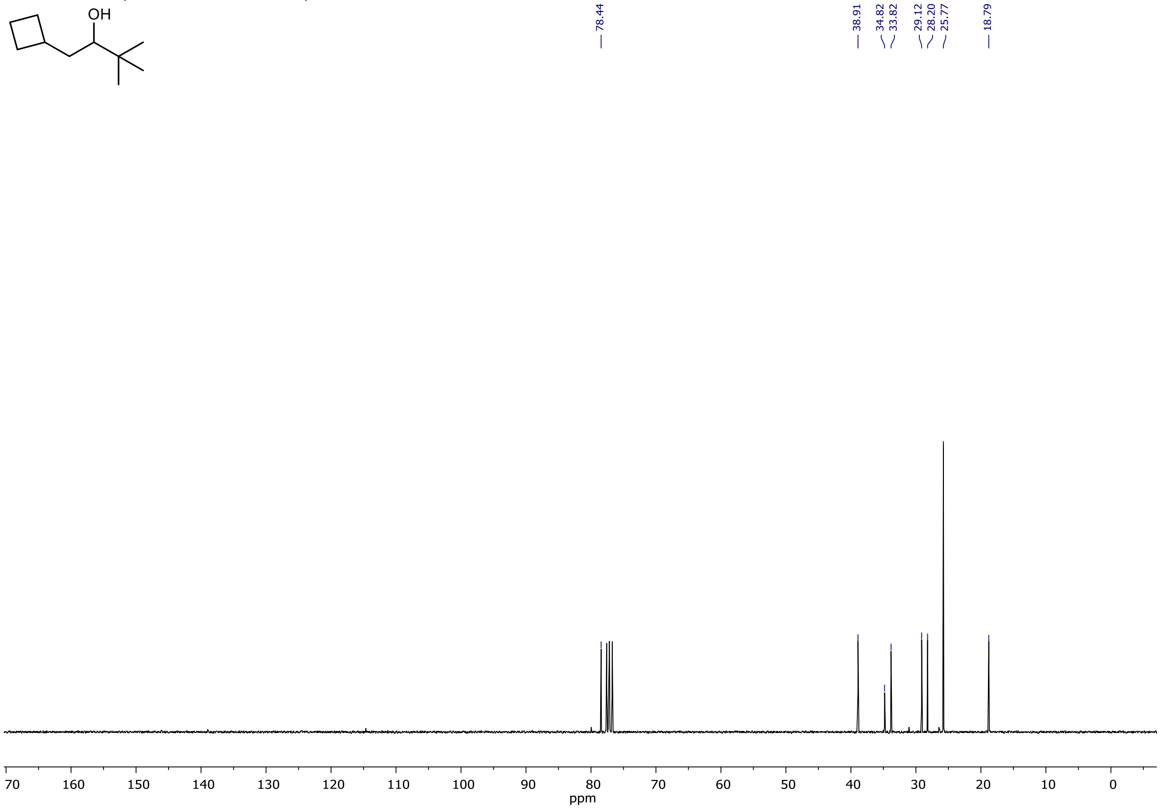

Supplementary Figure 36: NMR Spectra of 1-Cyclobutyl-3,3-dimethylbutan-2-ol (1i)

^{1}H NMR (400 MHz, $CDCl_3$)

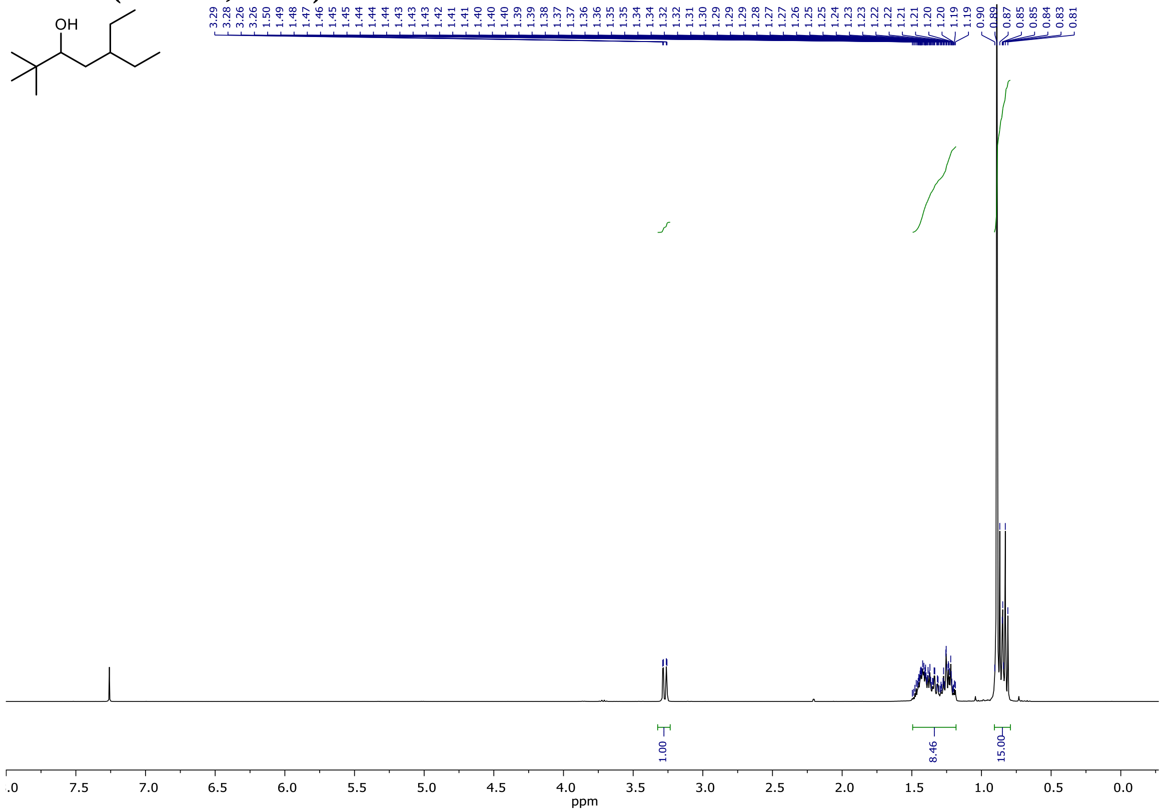

^{13}C NMR (101 MHz, $CDCl_3$)

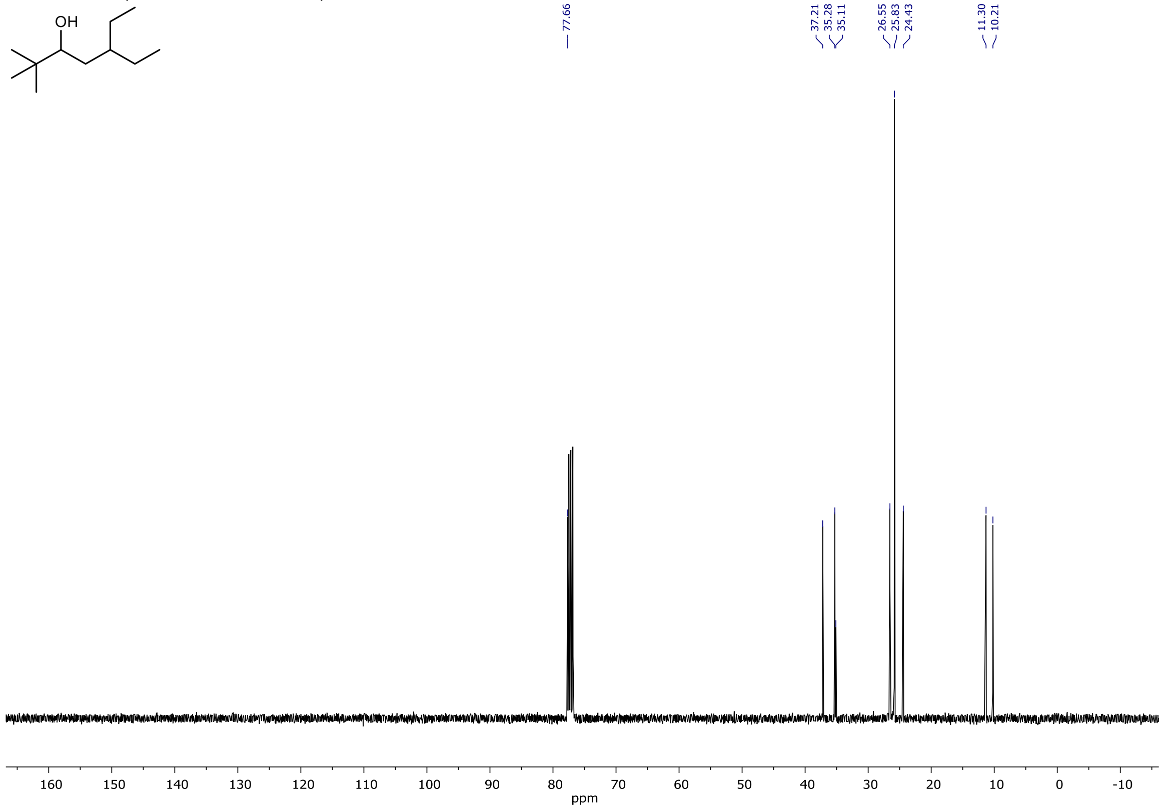

Supplementary Figure 37: NMR Spectra of 5-Ethyl-2,2-dimethylheptan-3-ol (1j)

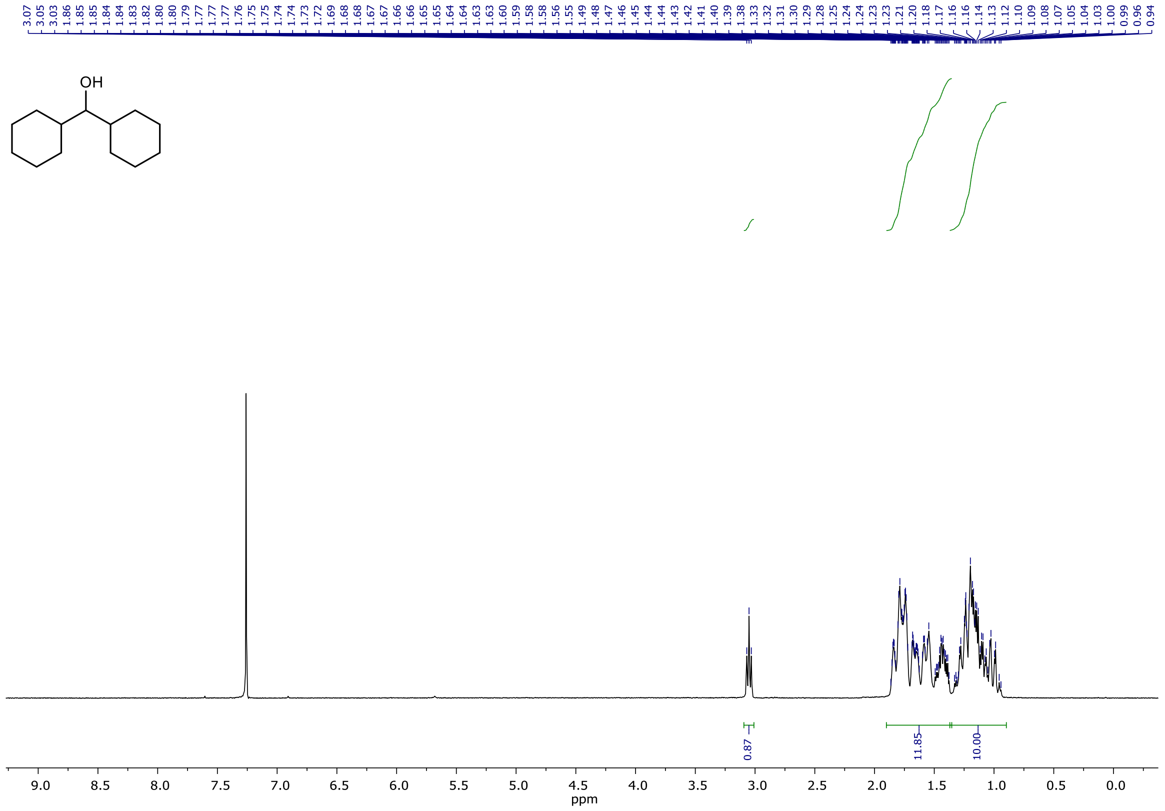

^{13}C NMR (75 MHz, $CDCl_3$)

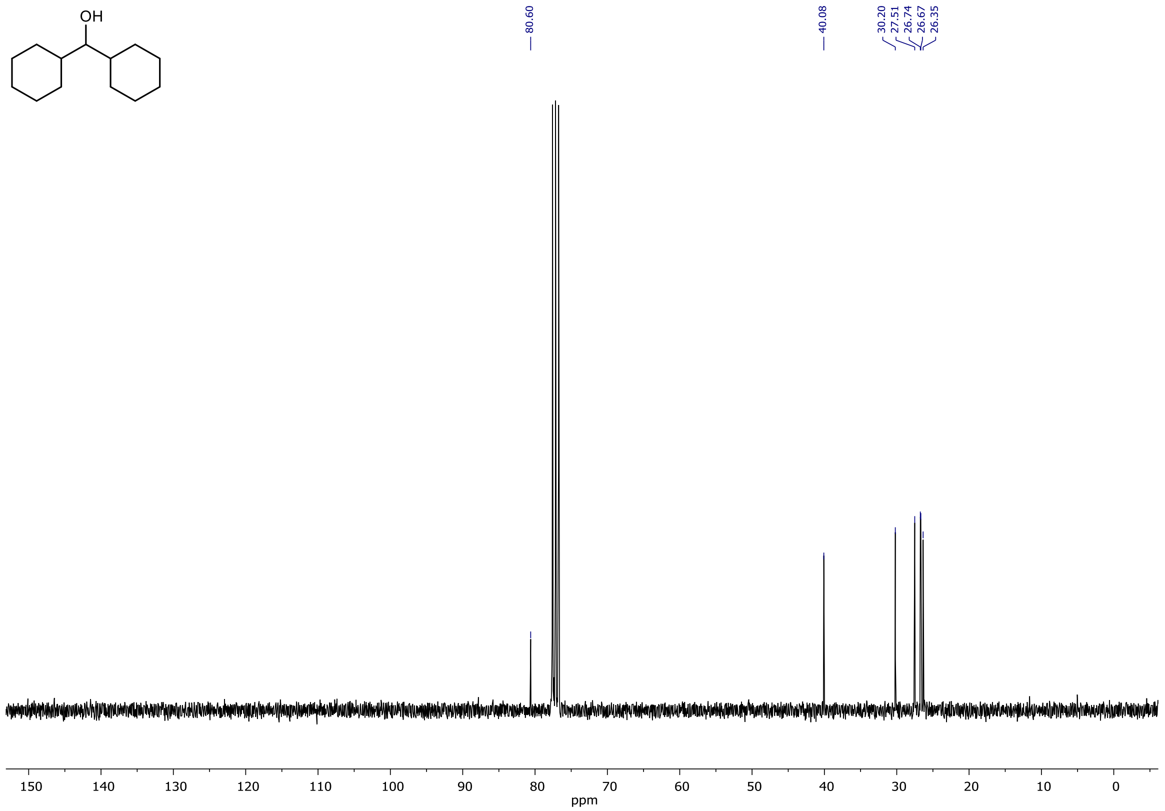

Supplementary Figure 38: NMR Spectra of Dicyclohexylmethanol (1m)

^{1}H NMR (400 MHz, CDCl$_3$)

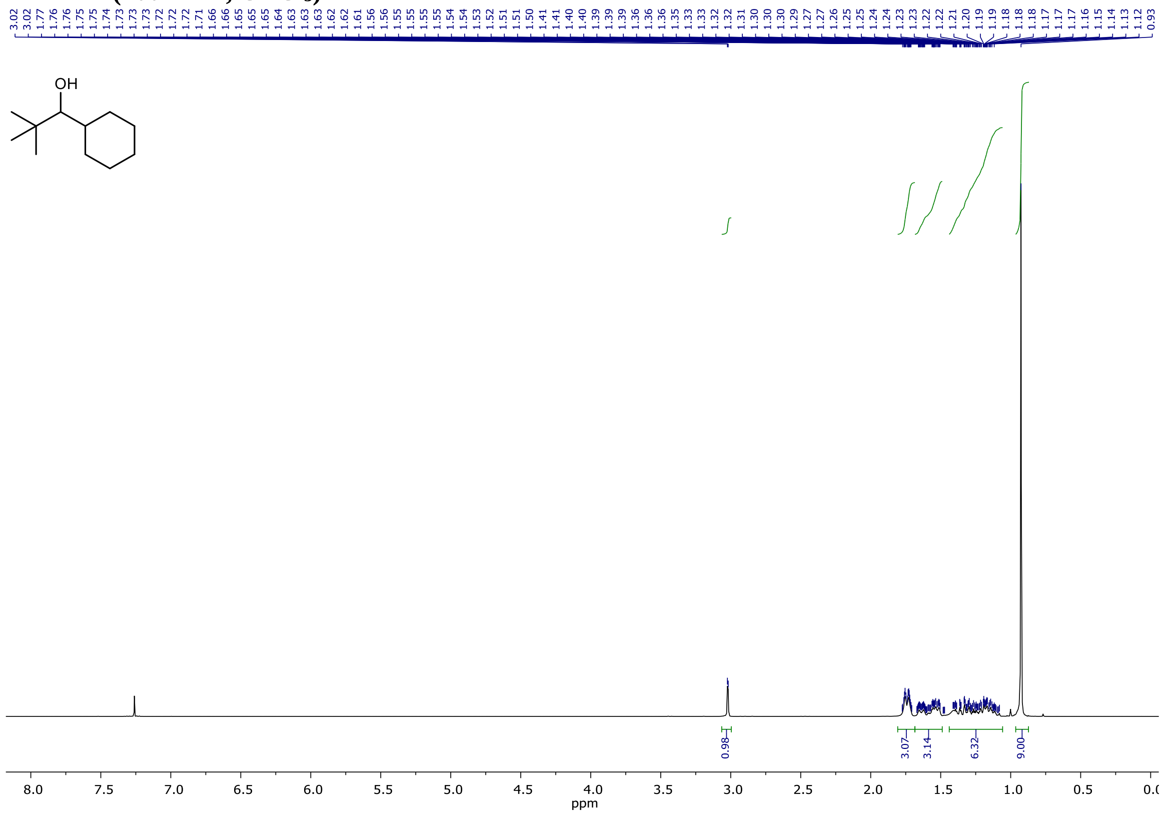

^{13}C NMR (101 MHz, CDCl$_3$)

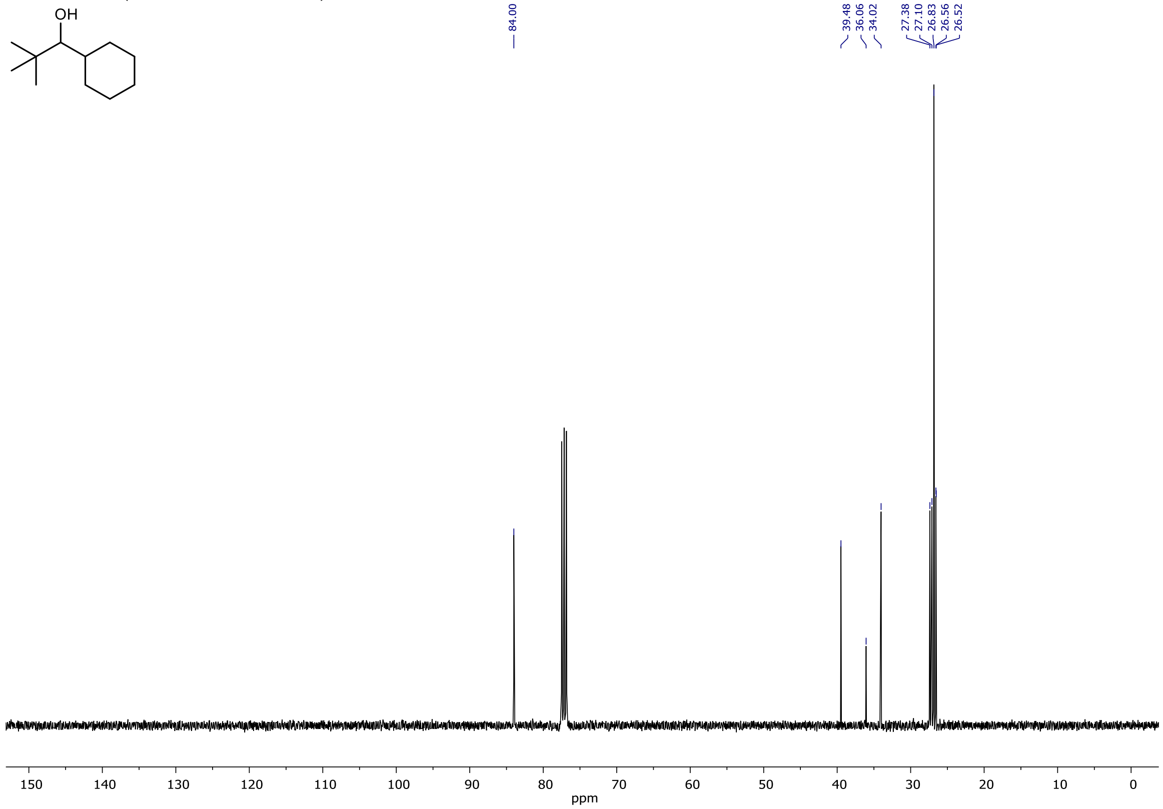

Supplementary Figure 39: NMR Spectra of 1-Cyclohexyl-2,2-dimethylpropan-1-ol (1n)

^{1}H NMR (300 MHz, $CDCl_3$)

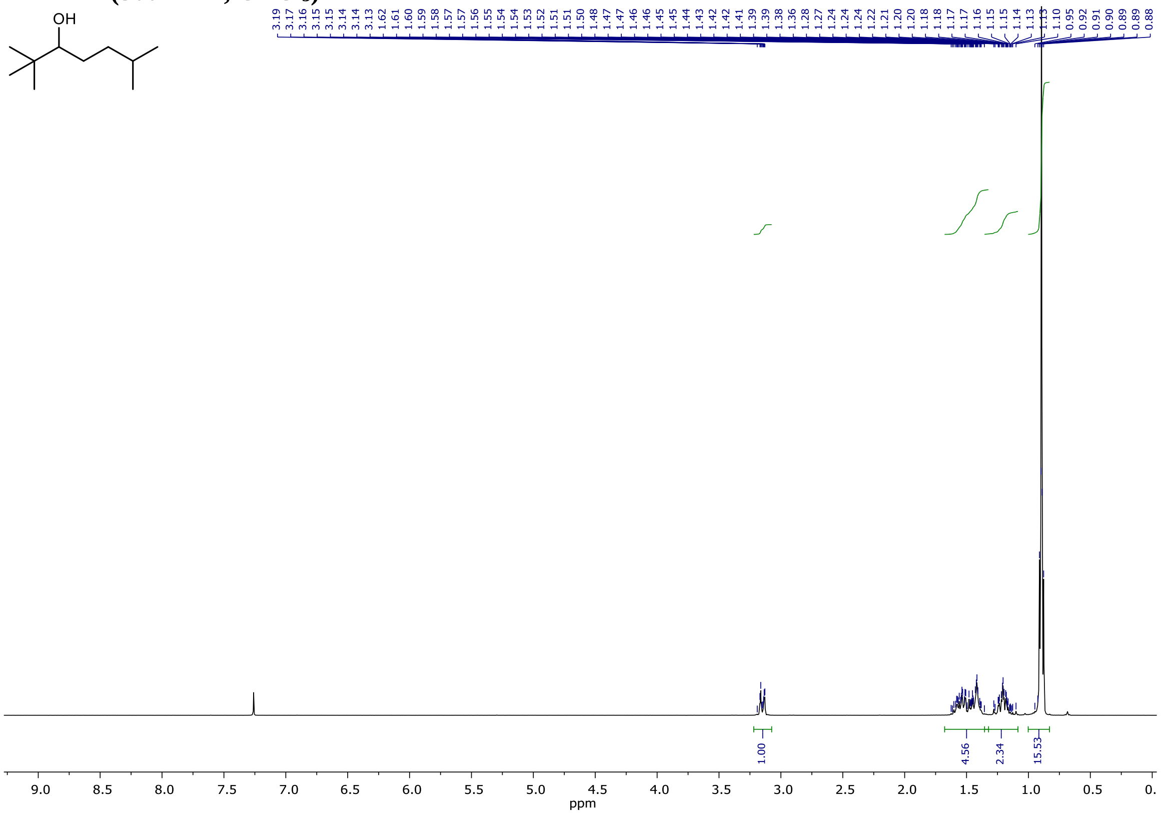

^{13}C NMR (75 MHz, $CDCl_3$)

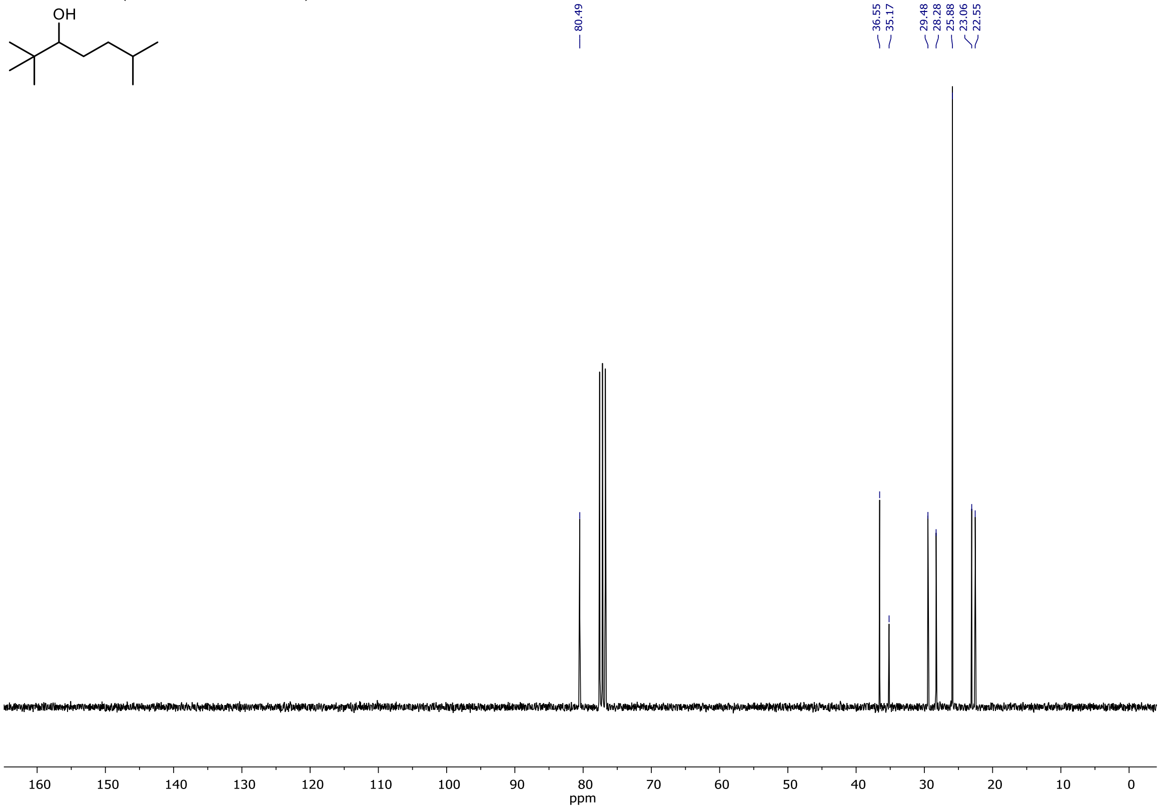

Supplementary Figure 40: NMR Spectra of 2,2,6-trimethylheptan-3-ol (1p)

^{1}H NMR (300 MHz, $CDCl_3$)

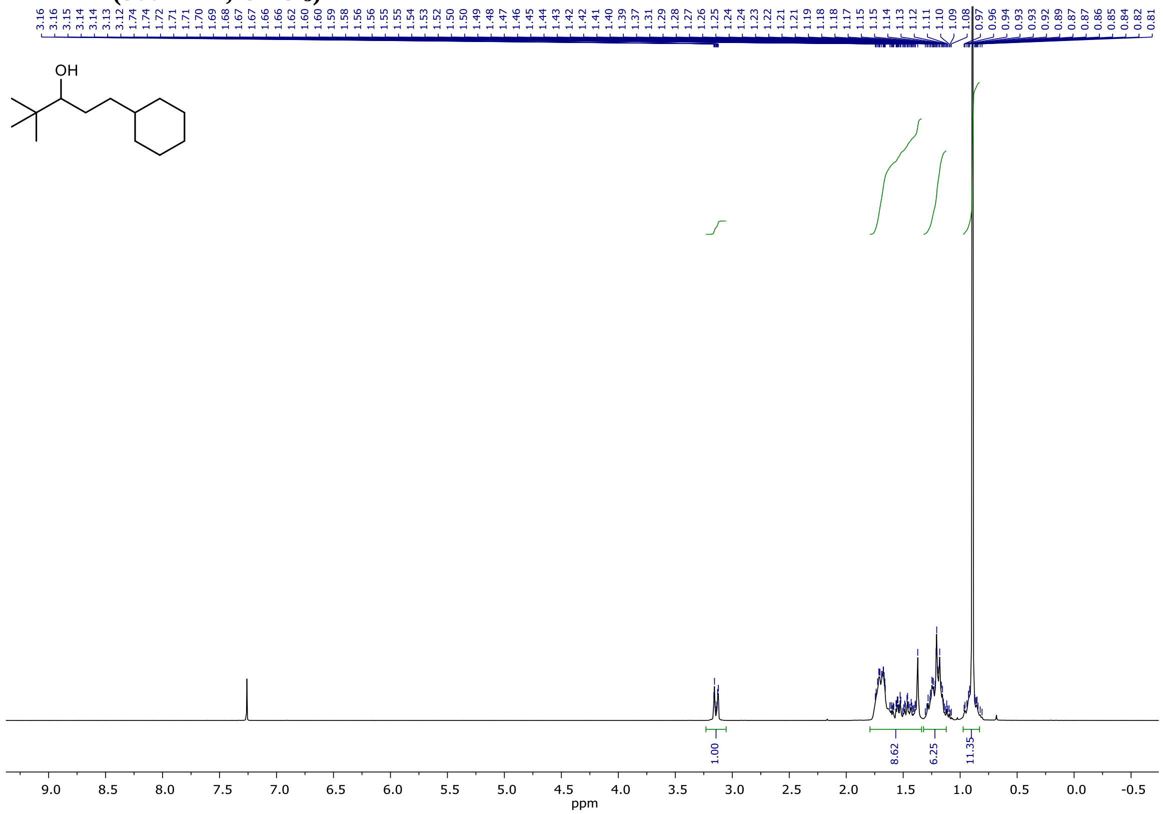

^{13}C NMR (75 MHz, $CDCl_3$)

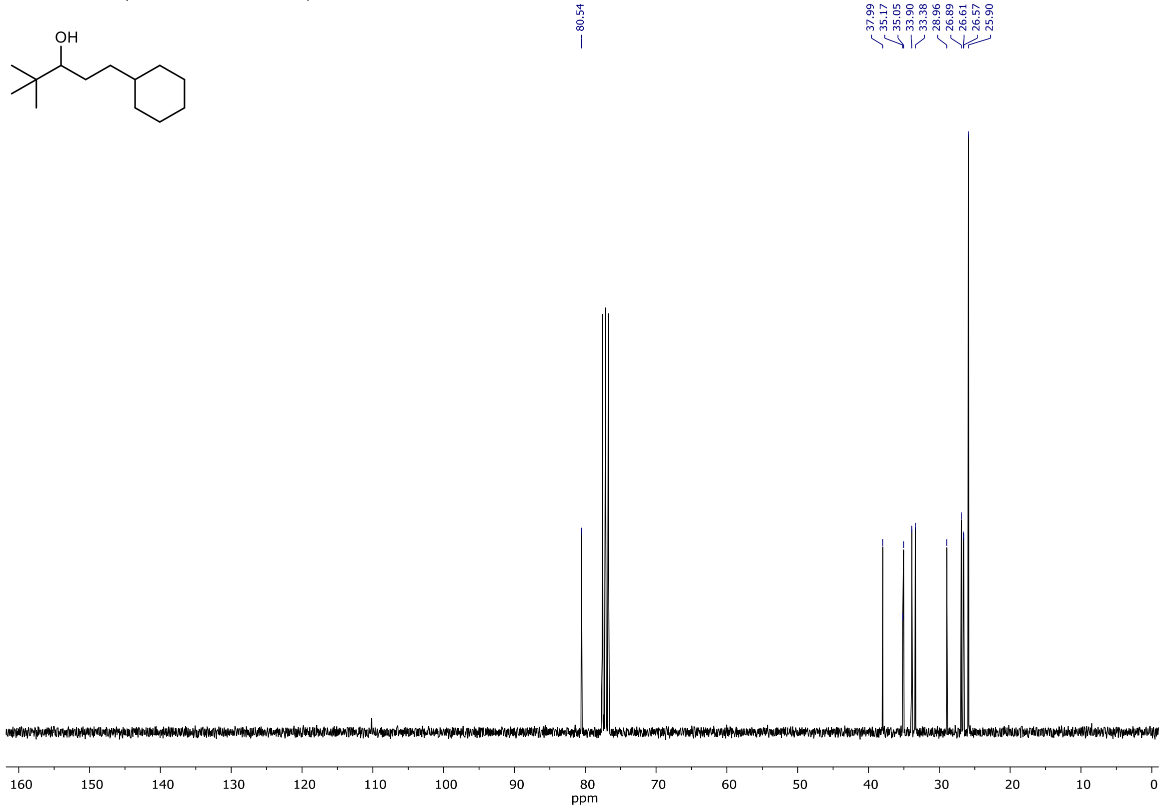

Supplementary Figure 41: NMR Spectra of 1-Cyclohexyl-4,4-dimethylpentan-3-ol (1q)

¹H NMR (300 MHz, CDCl₃)

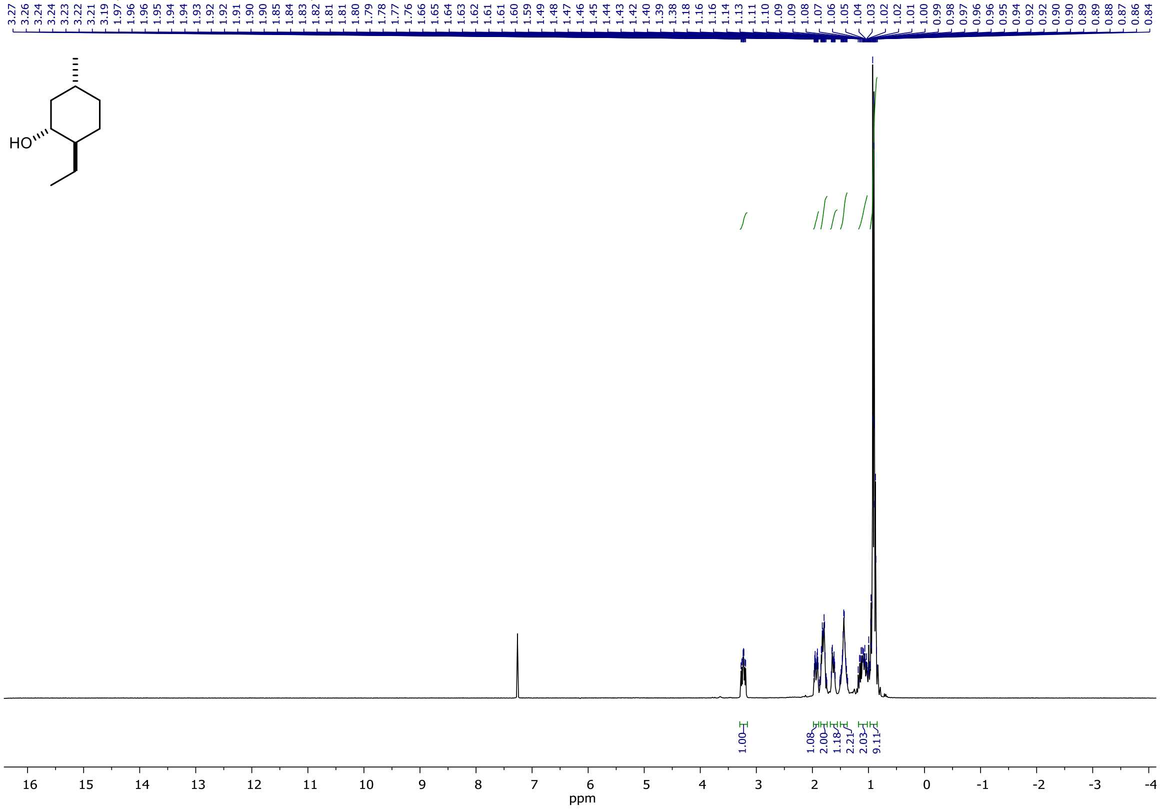

¹³C NMR (75 MHz, CDCl₃)

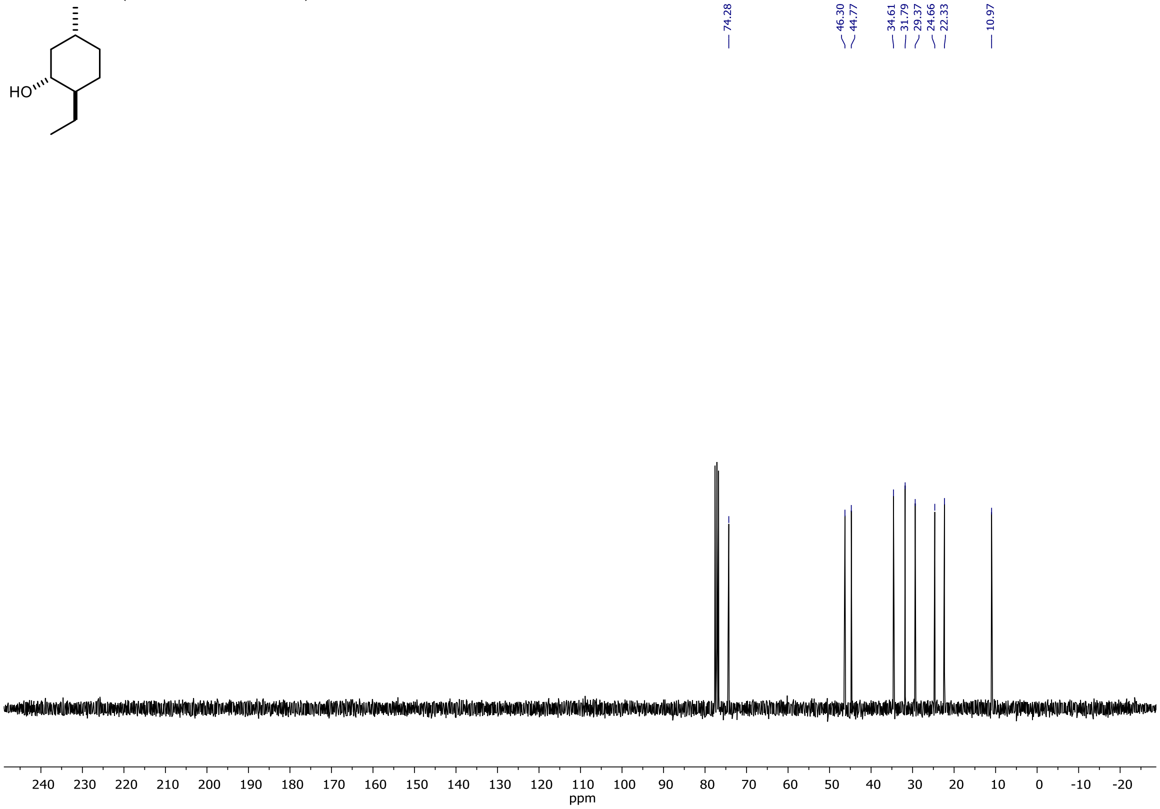

Supplementary Figure 42: NMR Spectra of 1-((1*R*,2*R*,4*R*)-2-Hydroxy-4-methylcyclohexyl)ethan-1-one (1r)

^{1}H NMR (300 MHz, $CDCl_3$)

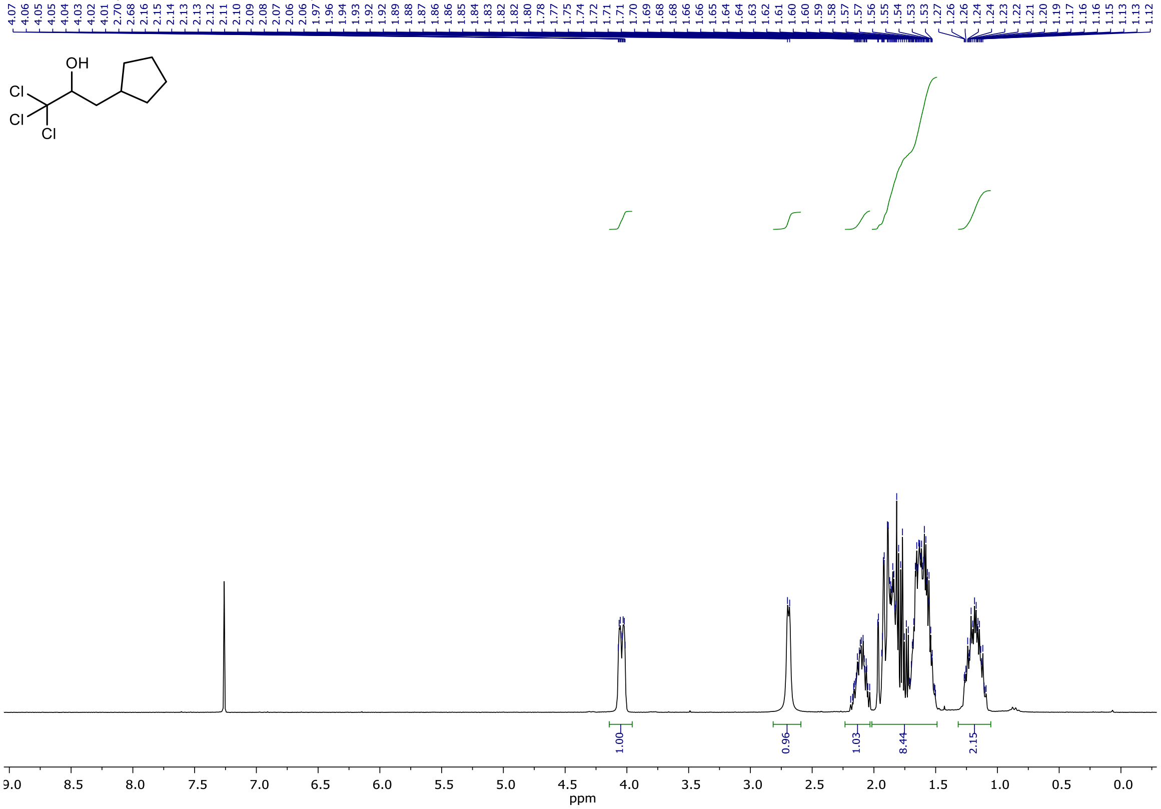

^{13}C NMR (75 MHz, $CDCl_3$)

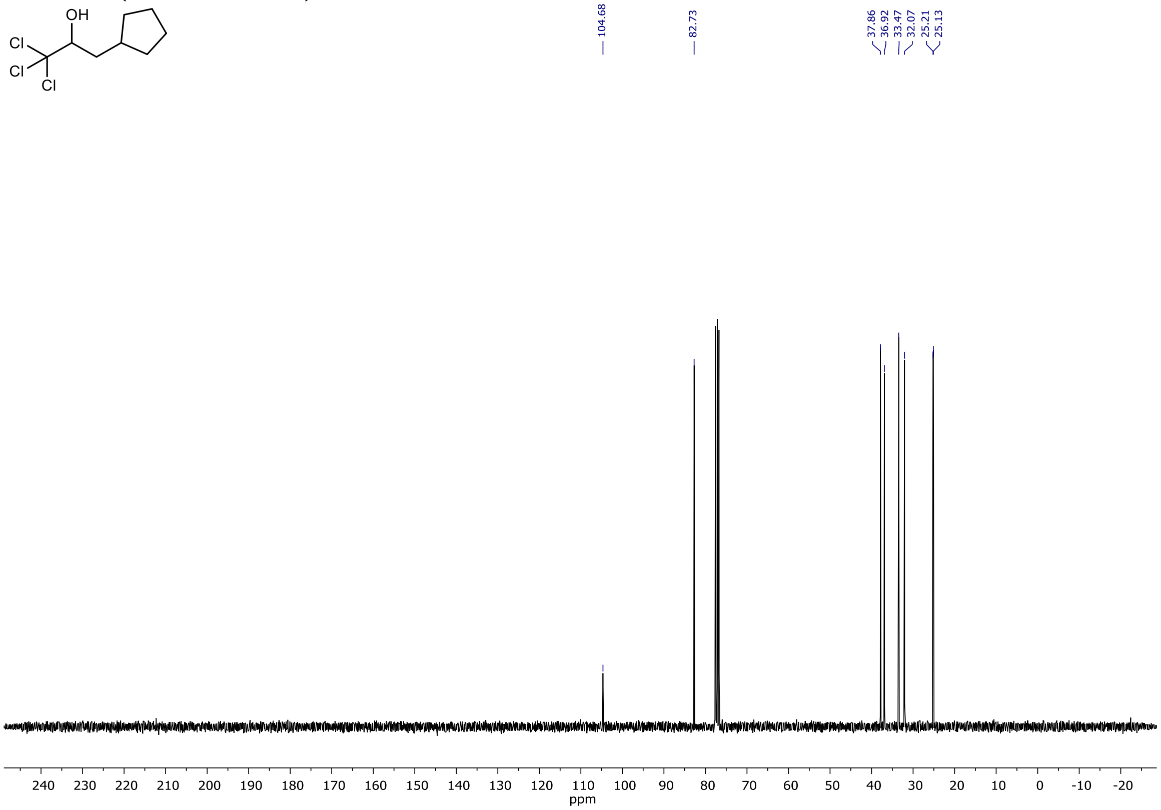

Supplementary Figure 43: NMR Spectra of 1,1,1-Trichloro-3-cyclopentylpropan-2-ol (1t)

^{1}H NMR (300 MHz, $CDCl_3$)

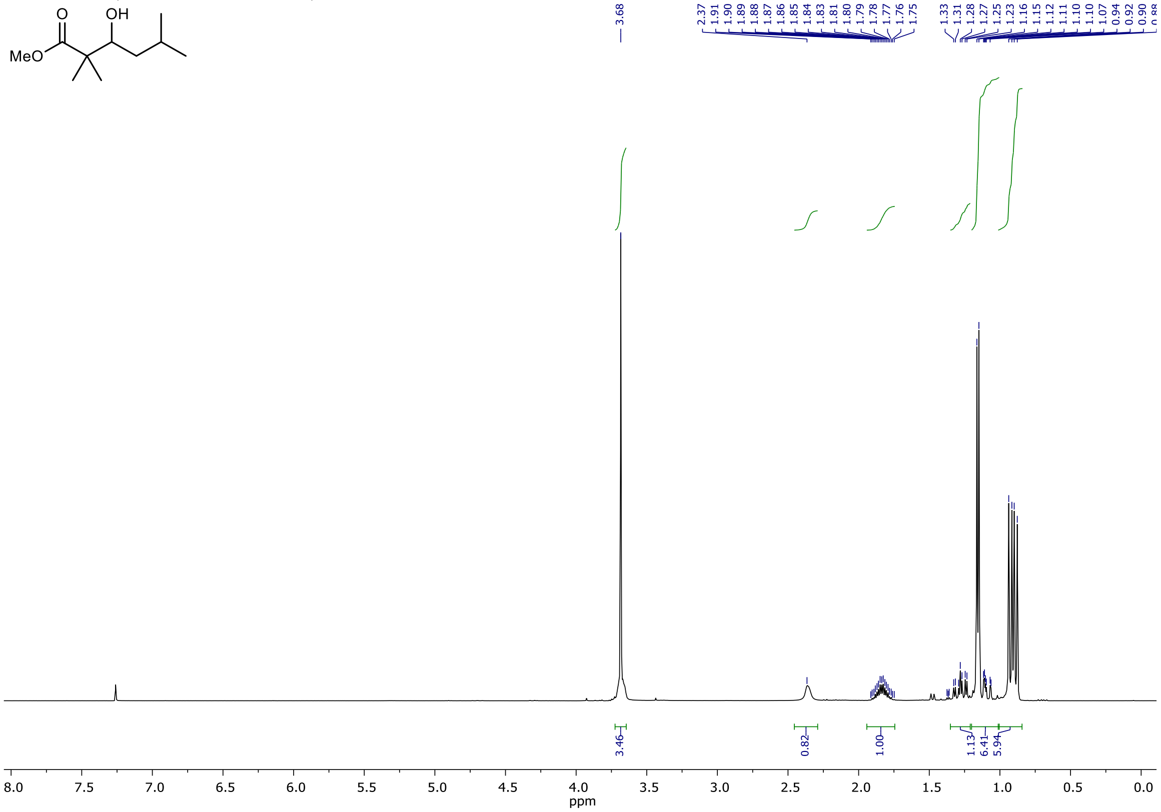

^{13}C NMR (75 MHz, $CDCl_3$)

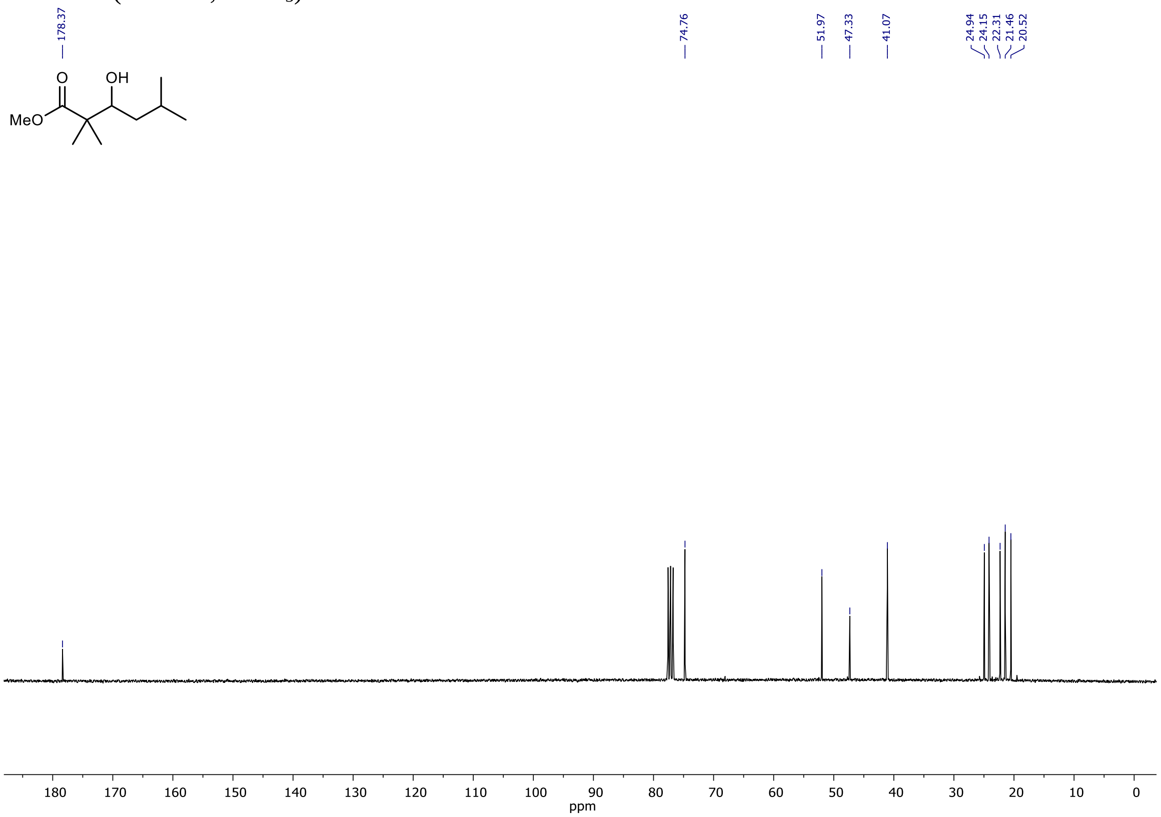

Supplementary Figure 44: NMR Spectra of Methyl 3-hydroxy-2,2,5-trimethylhexanoate (1v)

^{1}H NMR (300 MHz, CDCl$_3$)

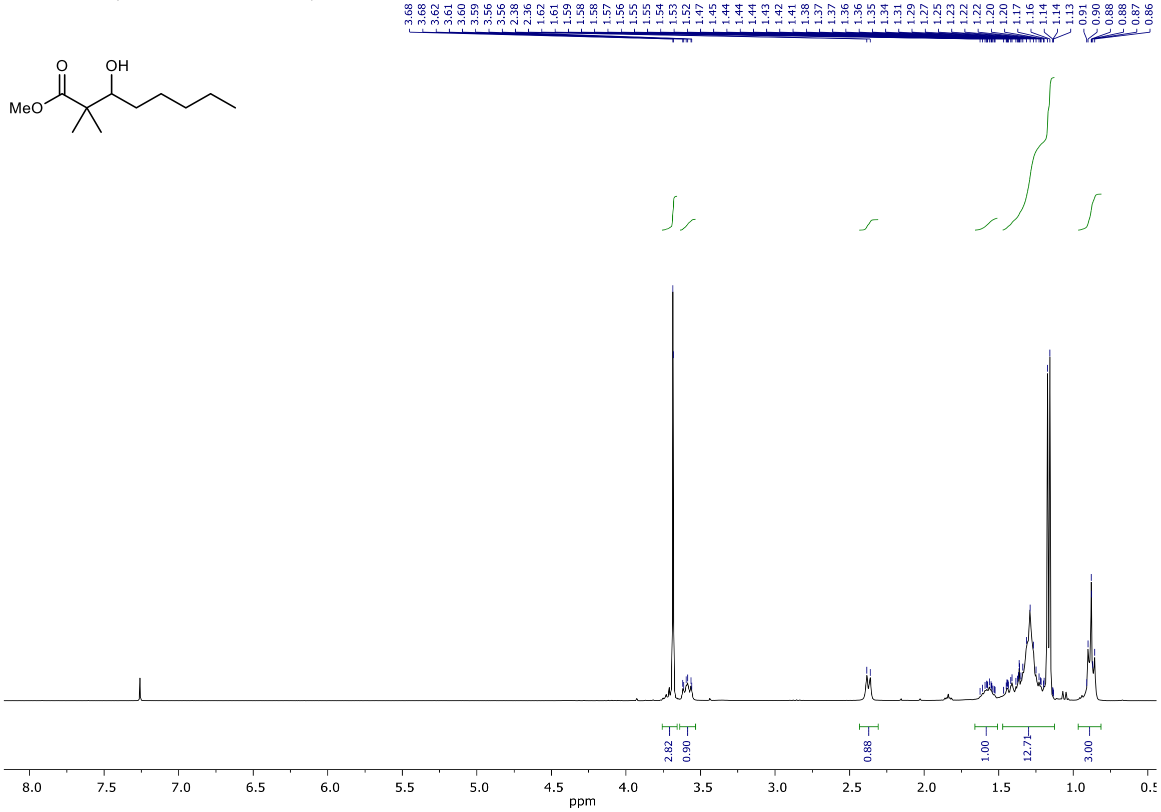

^{13}C NMR (75 MHz, CDCl$_3$)

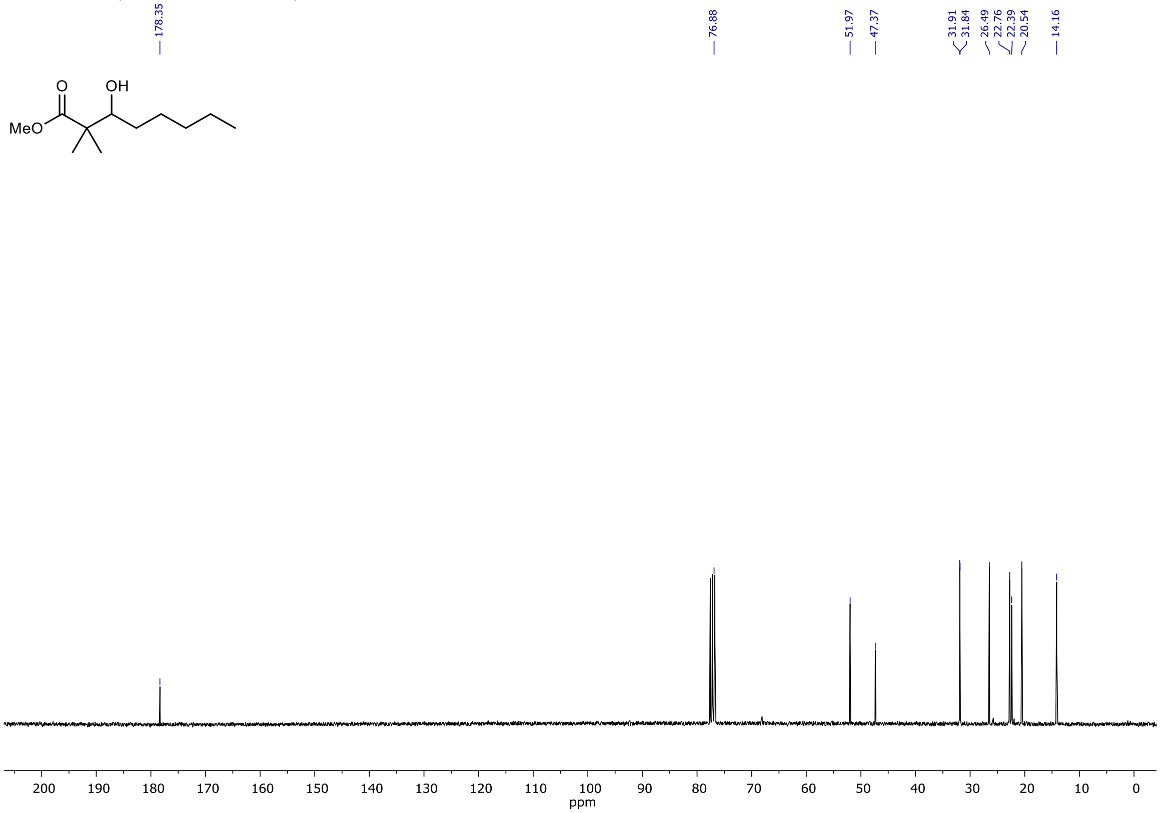

Supplementary Figure 45: NMR Spectra of Methyl 3-hydroxy-2,2-dimethyloctanoate (1w)

^{1}H NMR (300 MHz, $CDCl_3$)

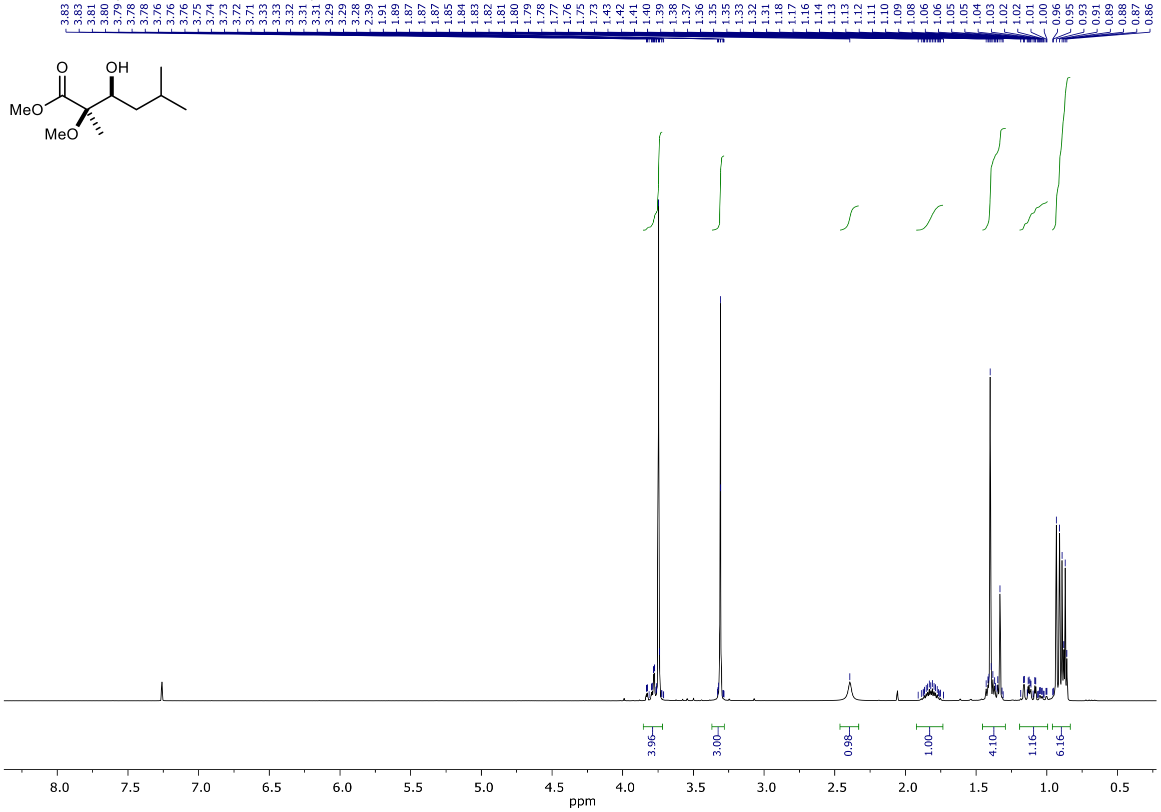

^{13}C NMR (75 MHz, $CDCl_3$)

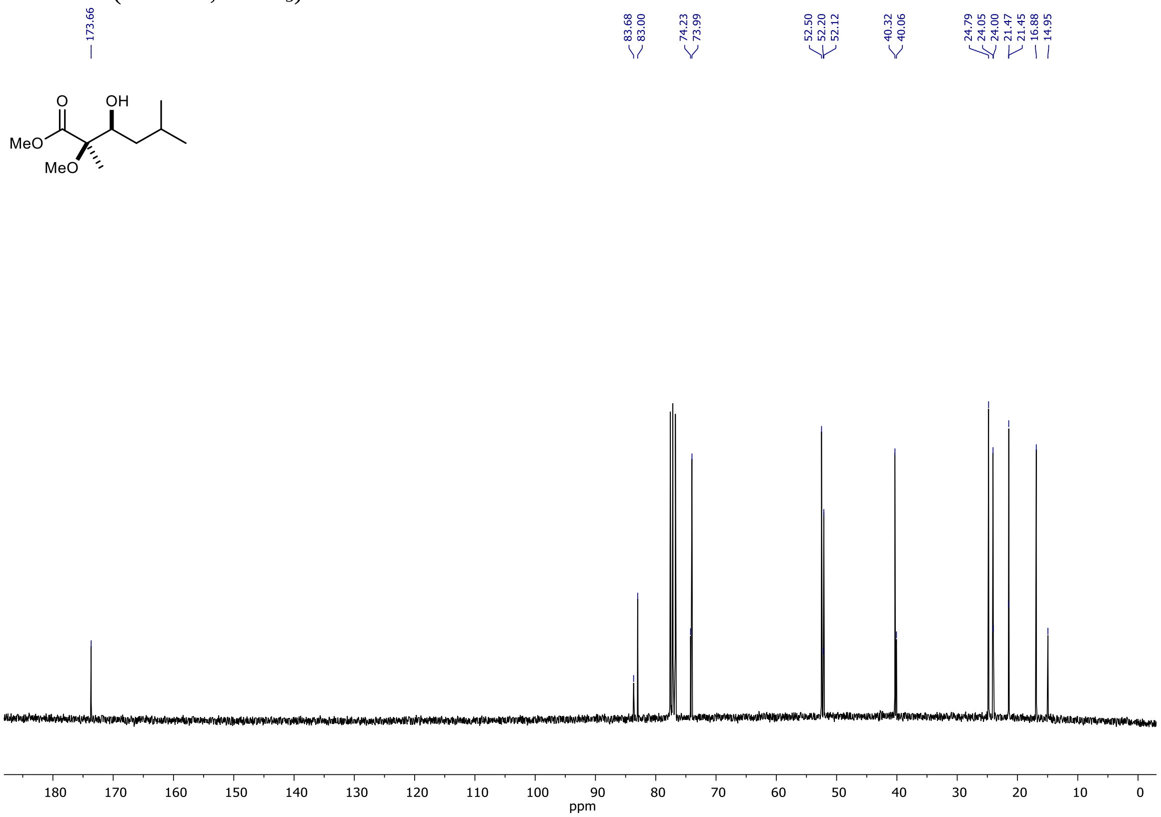

Supplementary Figure 46: NMR Spectra of *rac* Methyl (2*R*,3*S*)-3-hydroxy-2-methoxy-2,5-dimethylhexanoate (1x)

^{1}H NMR (300 MHz, $CDCl_3$)

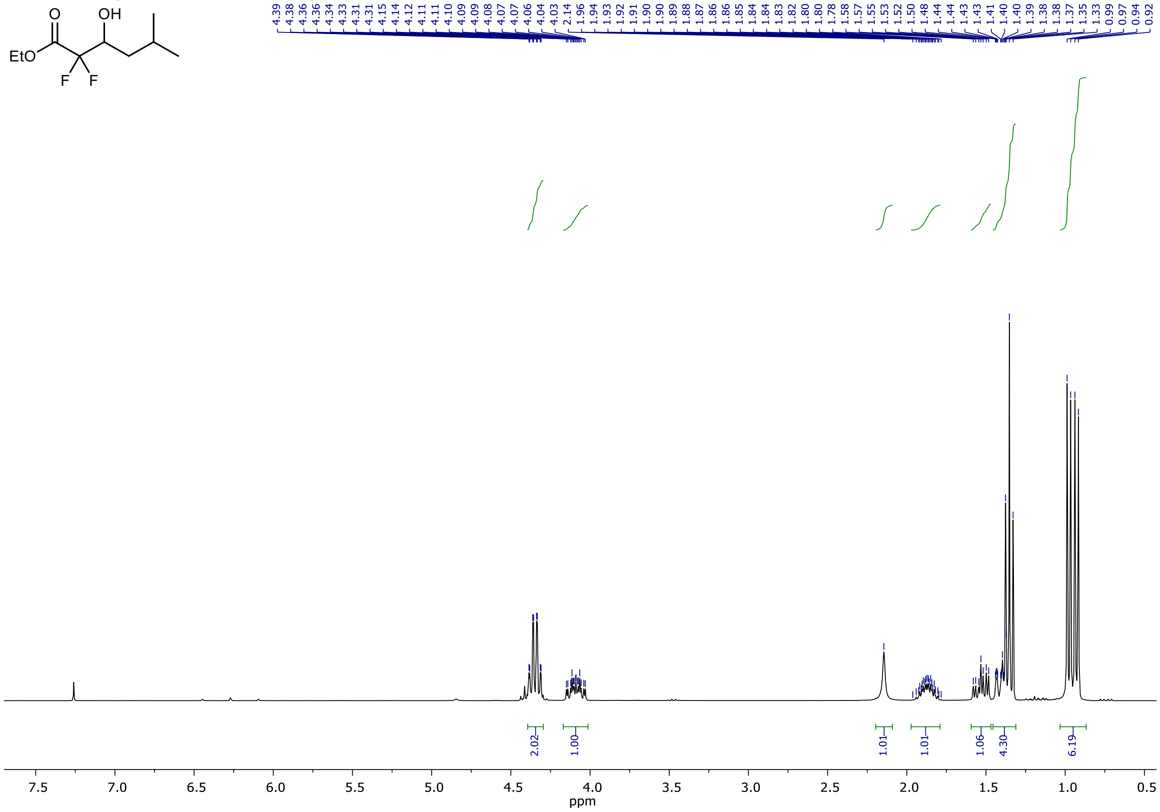

^{13}C NMR (75 MHz, $CDCl_3$)

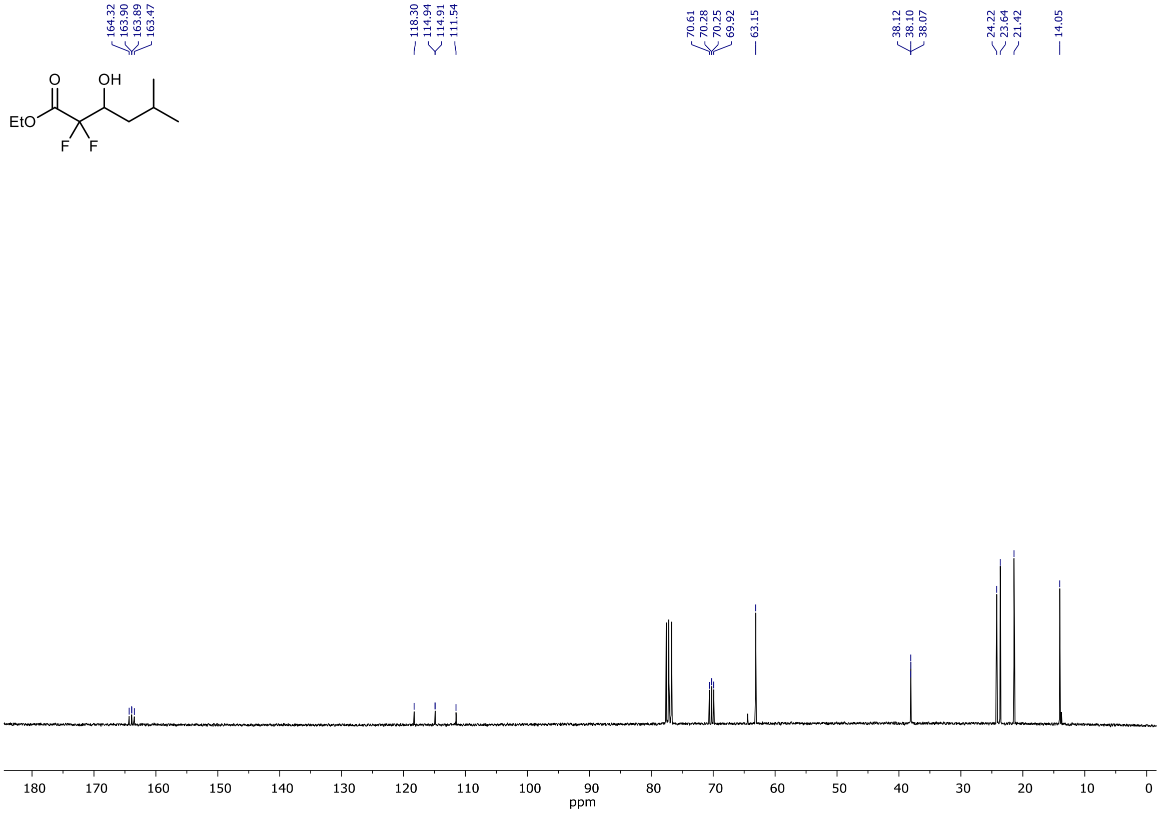

$^{19}F\{^{1}H\}$ NMR (282 Hz, $CDCl_3$)

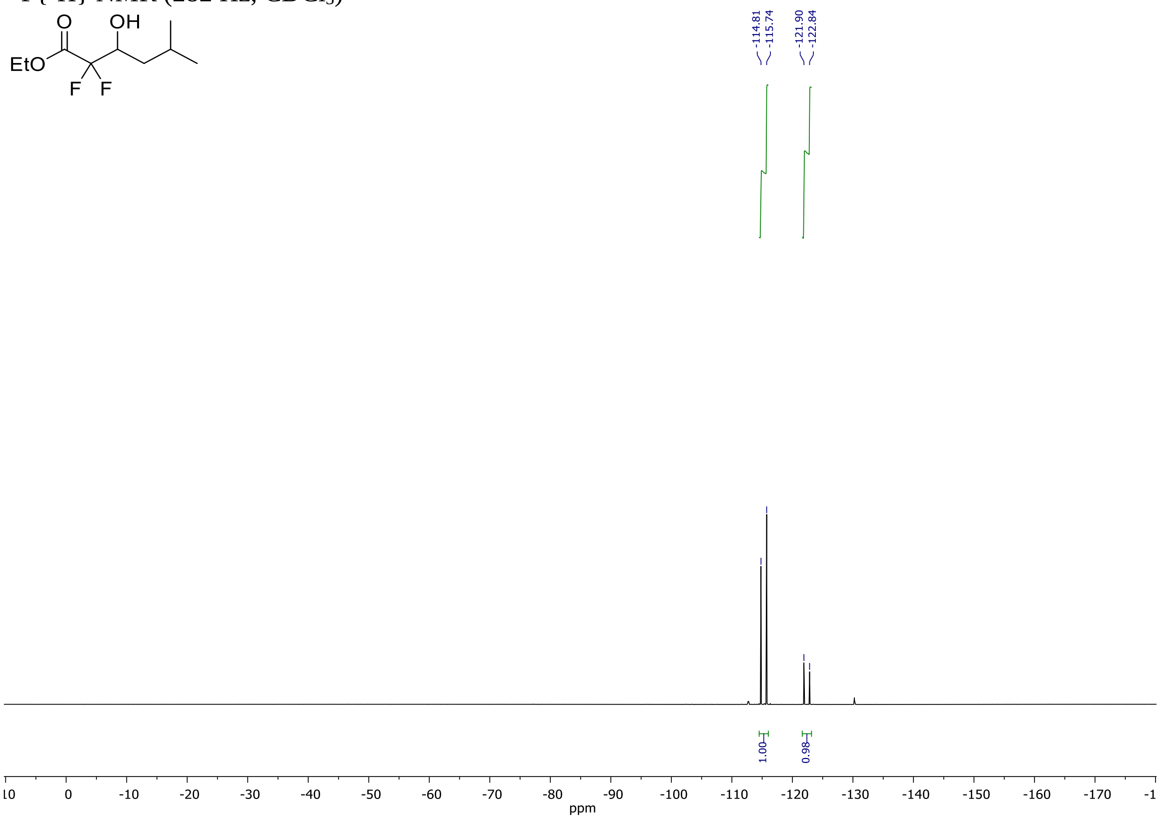

Supplementary Figure 47: NMR Spectra of Ethyl 2,2-difluoro-3-hydroxy-5-methylhexanoate (1z)

^{1}H NMR (300 MHz, $CDCl_3$)

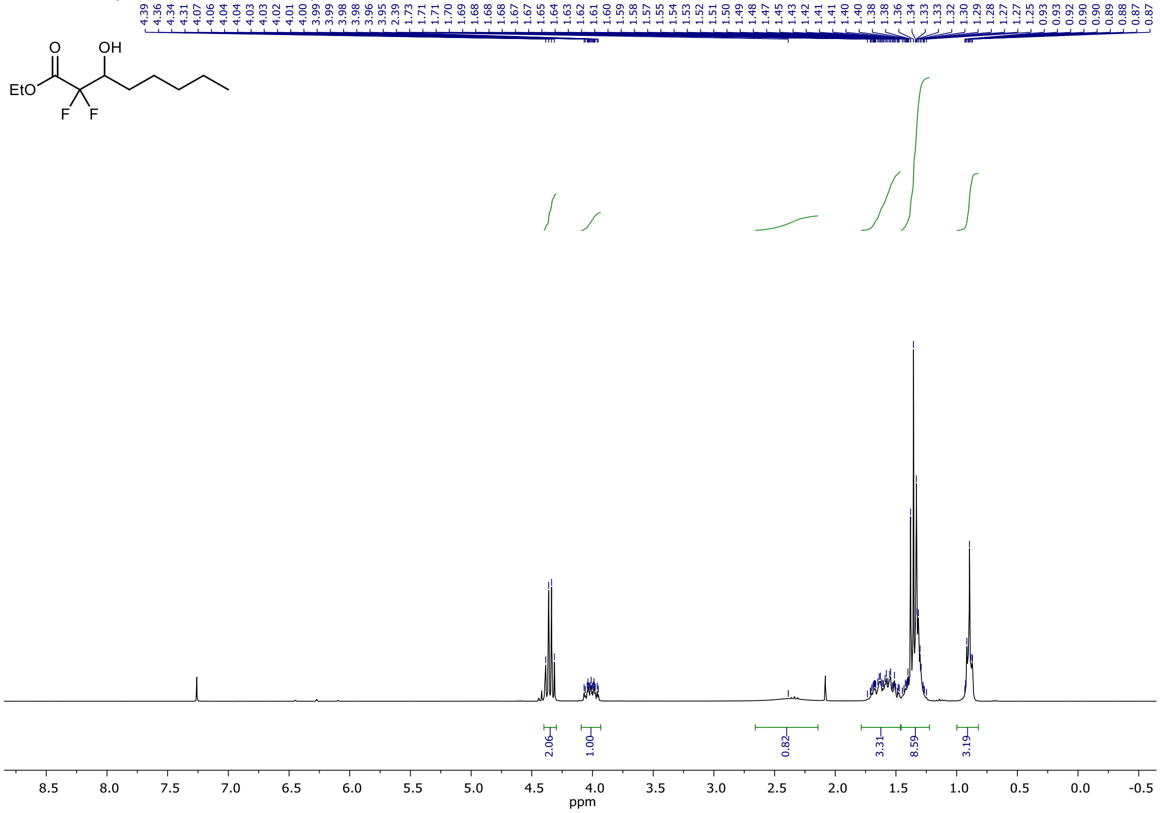

^{13}C NMR (75 MHz, $CDCl_3$)

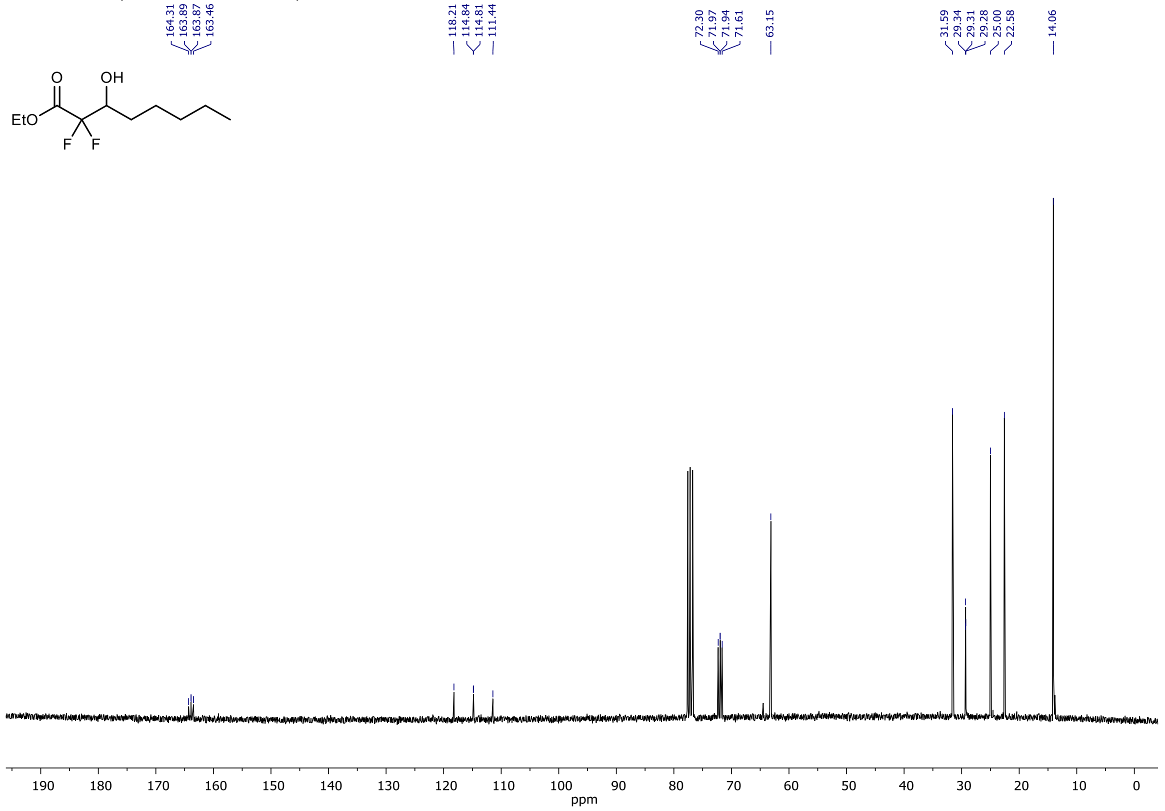

$^{19}F\{^1H\}$ NMR (282 Hz, $CDCl_3$)

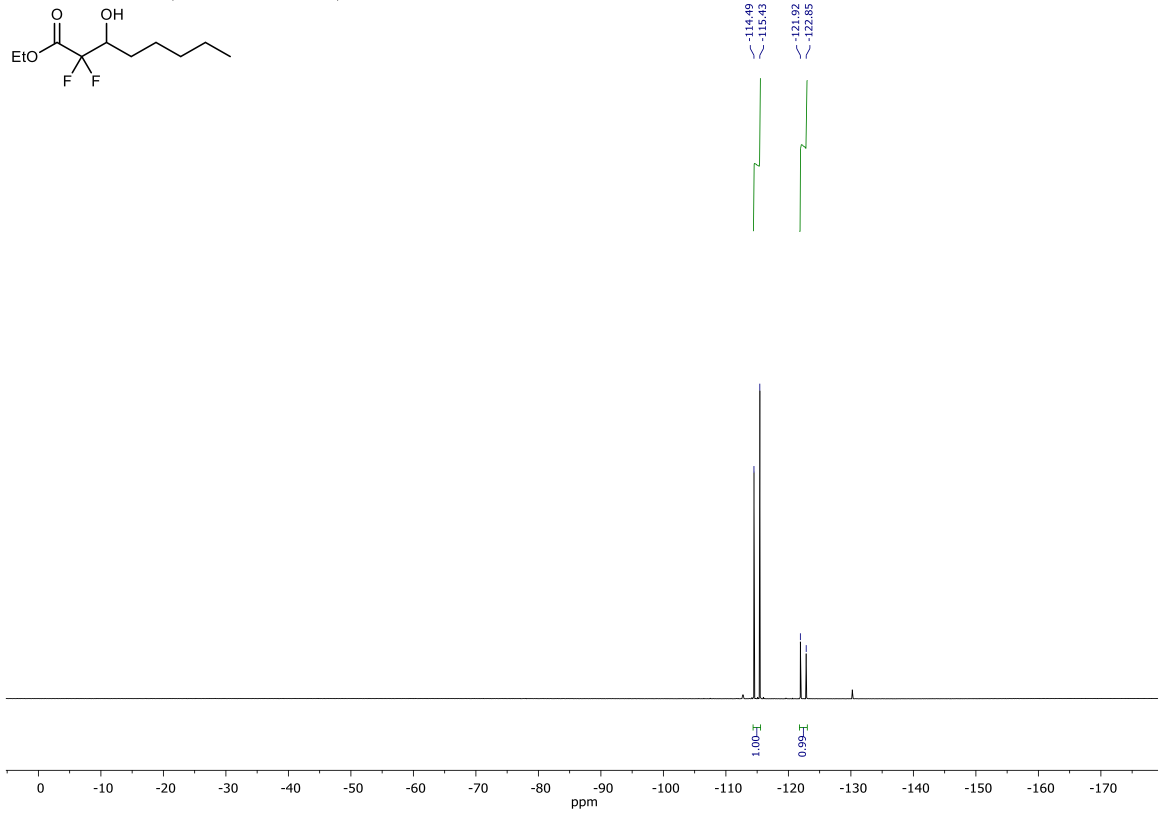

Supplementary Figure 48: NMR Spectra of Ethyl 2,2-difluoro-3-hydroxyoctanoate (1aa)

^{1}H NMR (300 MHz, $CDCl_3$)

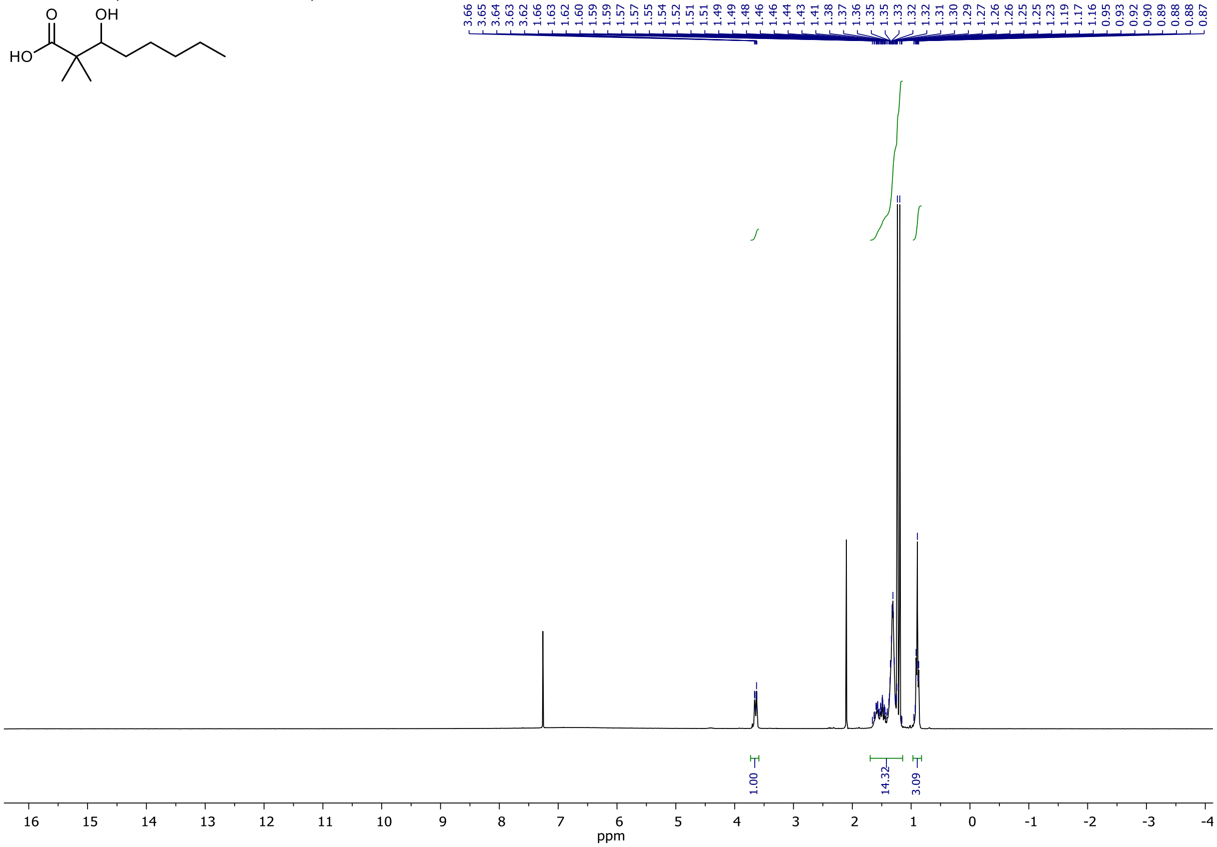

^{13}C NMR (75 MHz, $CDCl_3$)

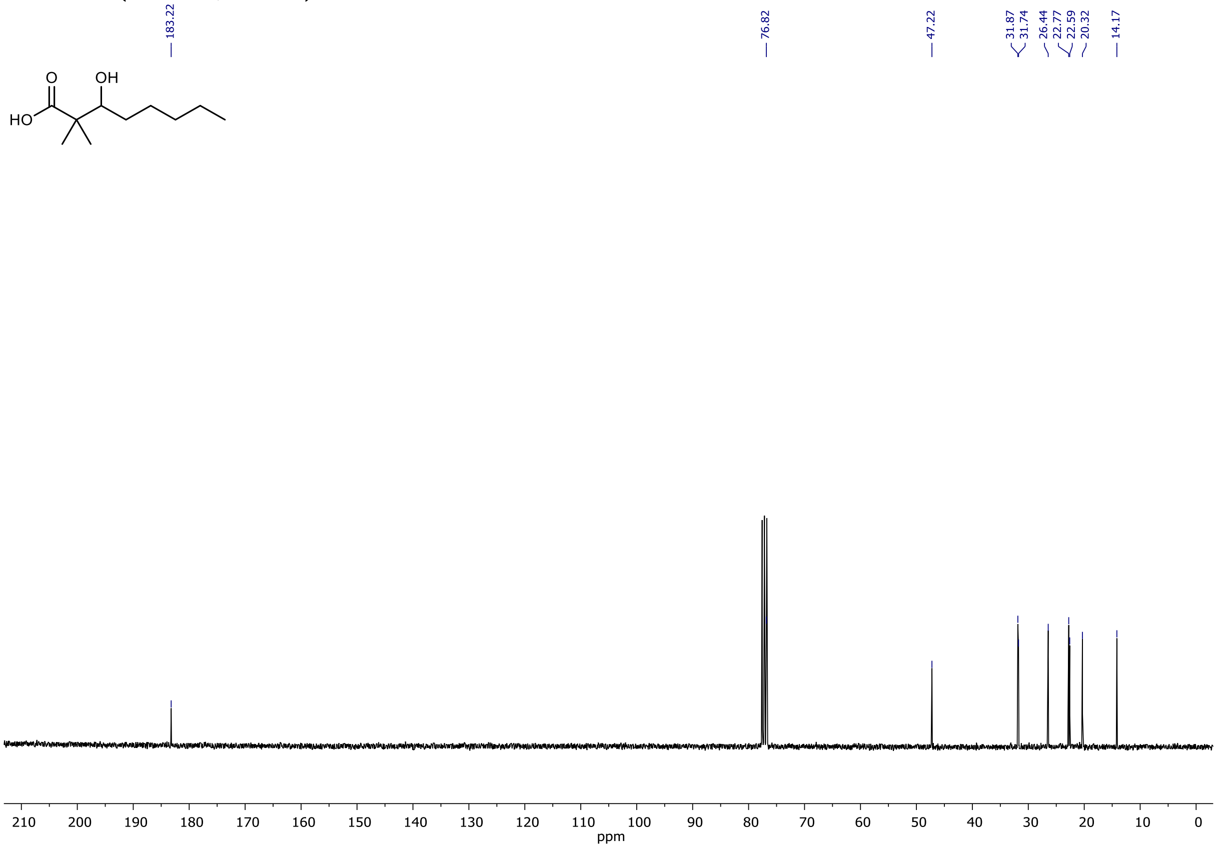

Supplementary Figure 49: NMR Spectra of 3-Hydroxy-2,2-dimethyloctanoic acid (S-21)

^{1}H NMR (400 MHz, $CDCl_3$)

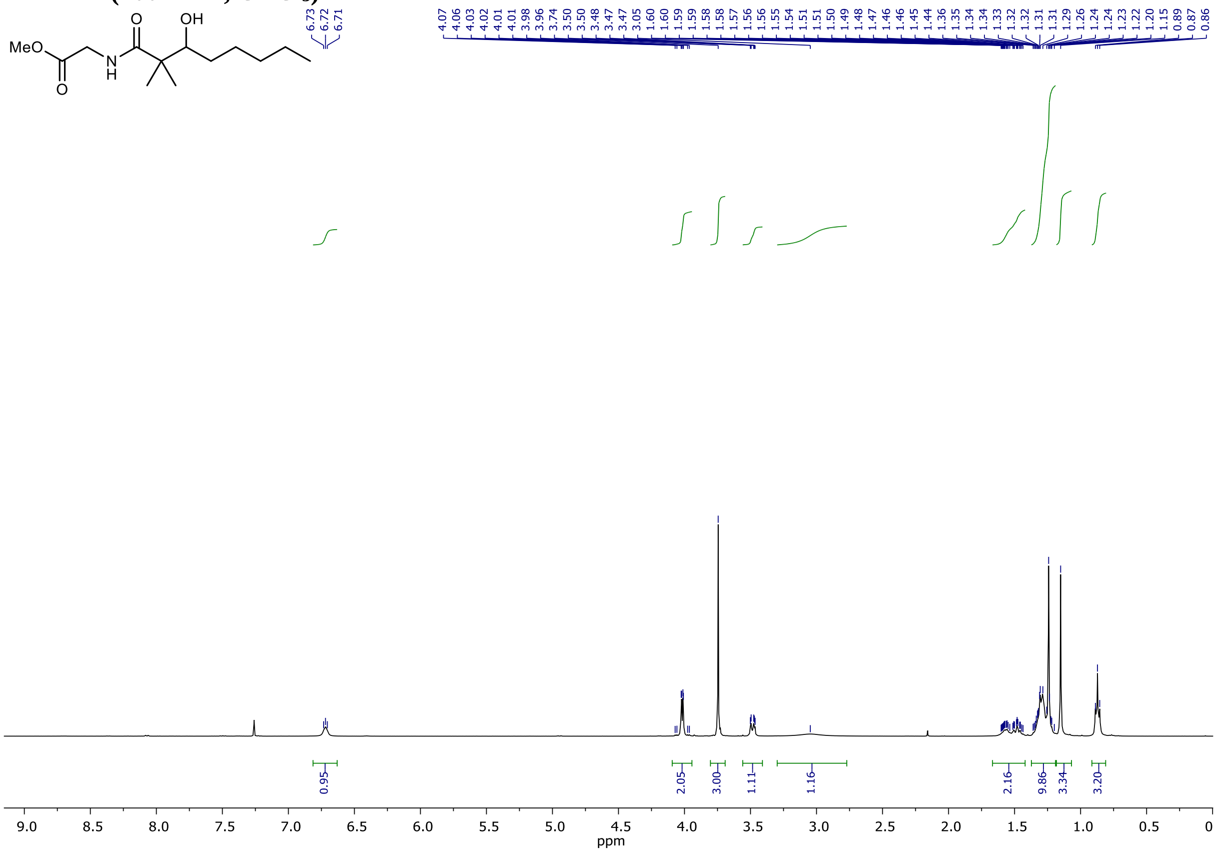

^{13}C NMR (101 MHz, $CDCl_3$)

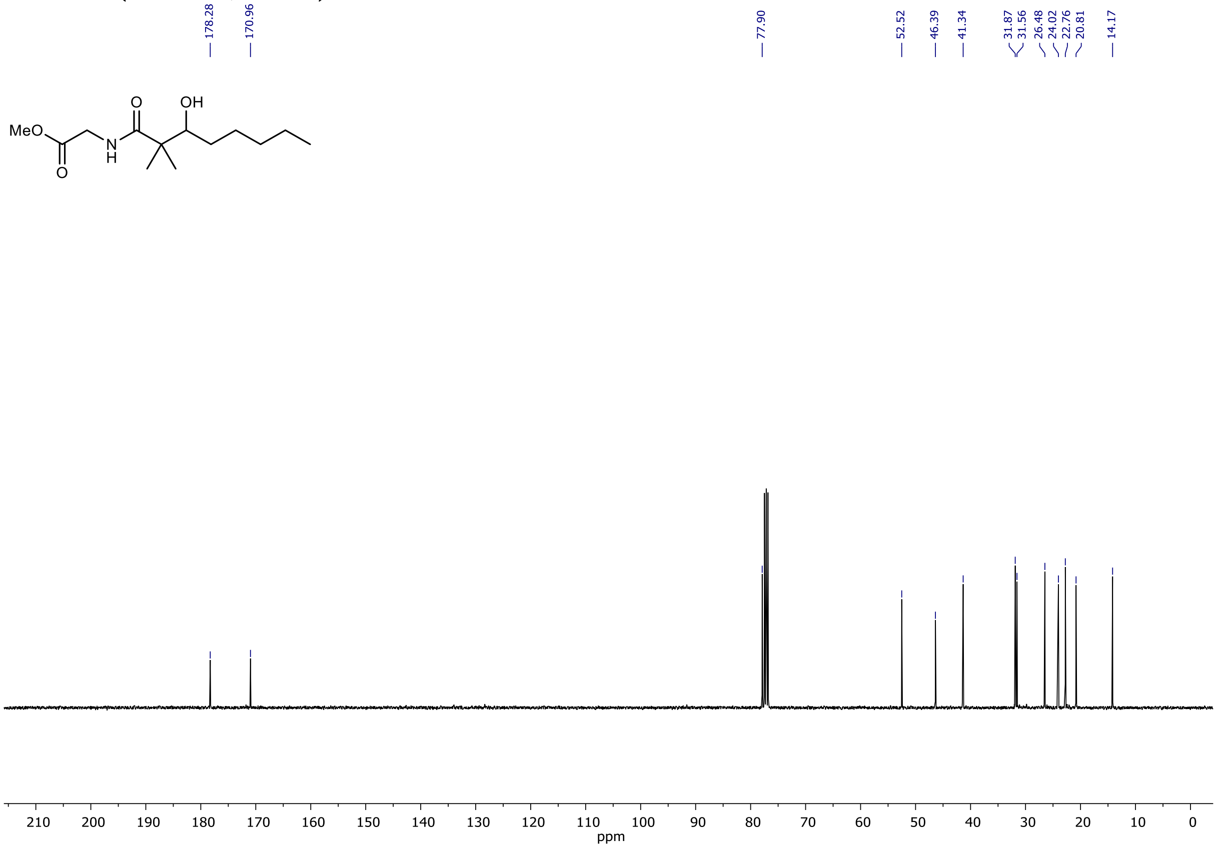

Supplementary Figure 50: NMR Spectra of **Methyl (3-hydroxy-2,2-dimethyloctanoyl)glycinate (1ab)**

^{1}H NMR (300 MHz, $CDCl_3$)

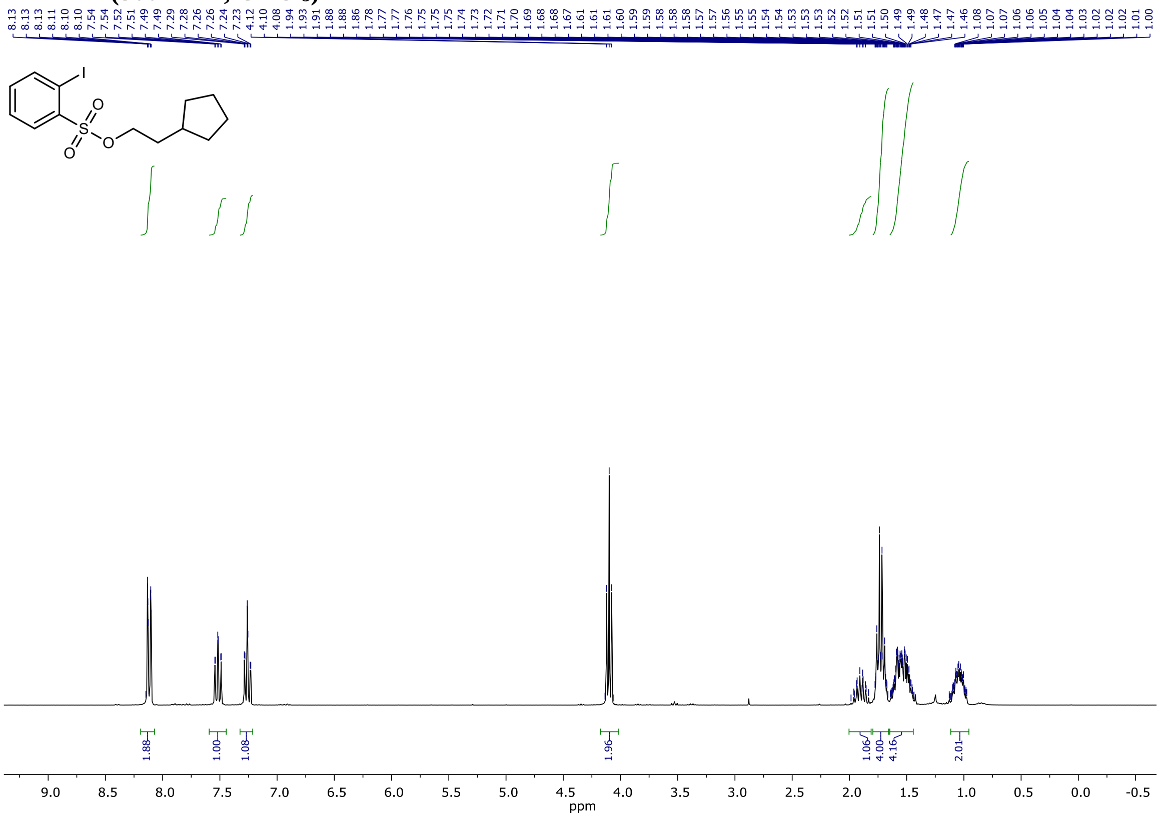

^{13}C NMR (75 MHz, $CDCl_3$)

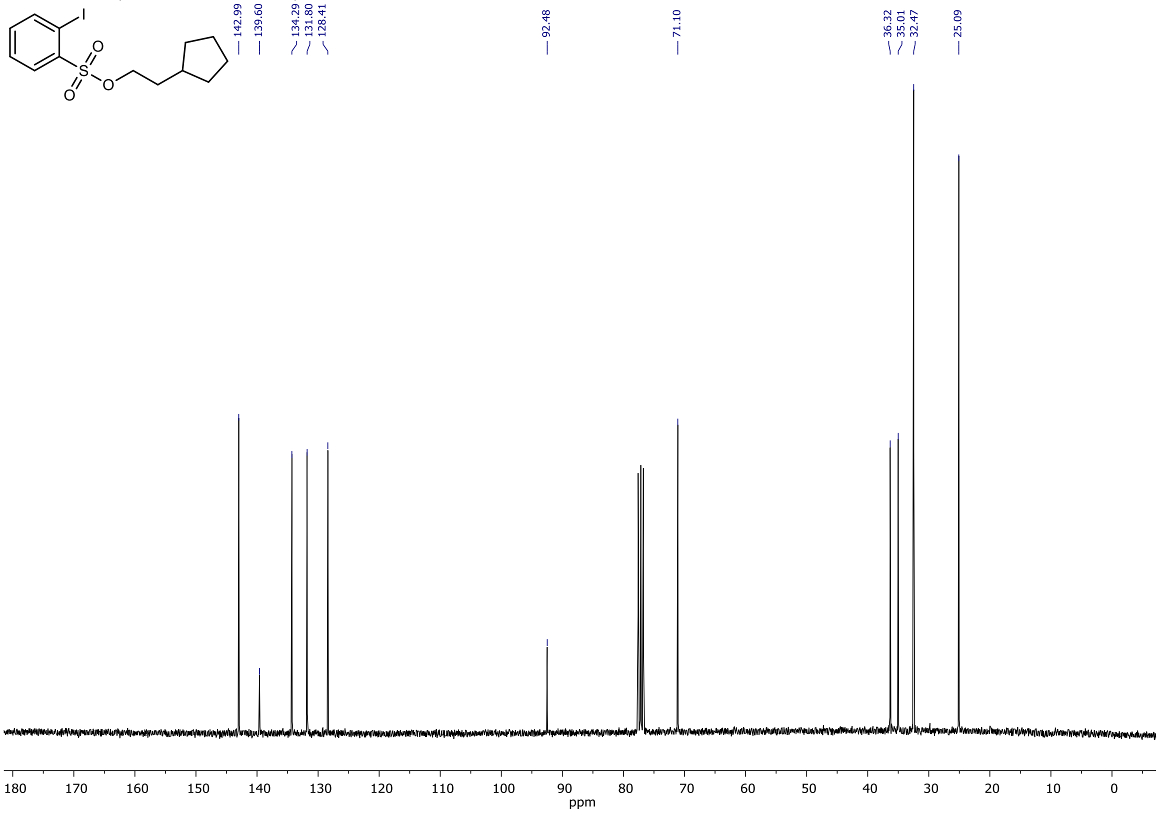

Supplementary Figure 51: NMR Spectra of 2-Cyclopentylethyl 2-iodobenzenesulfonate (S-22)

^{1}H NMR (400 MHz, CDCl3)

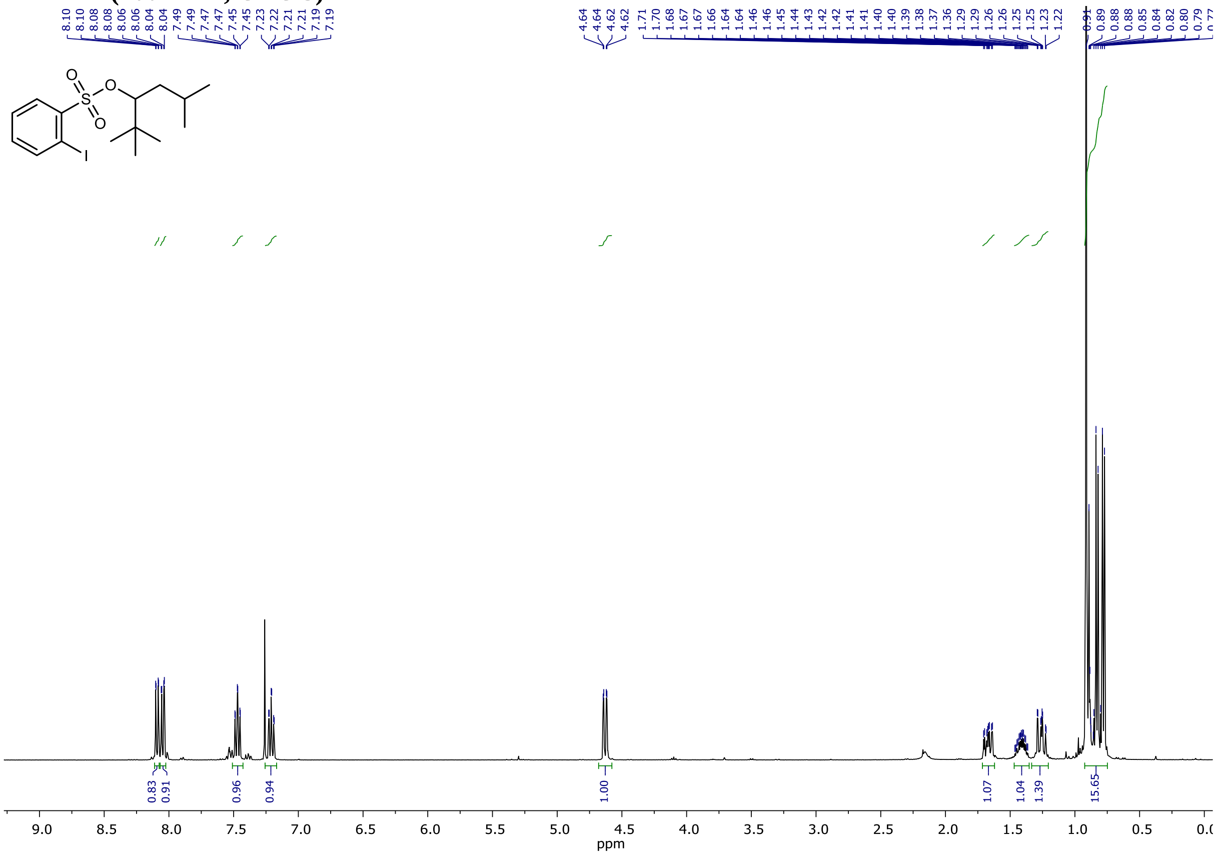

^{13}C NMR (101 MHz, CDCl3)

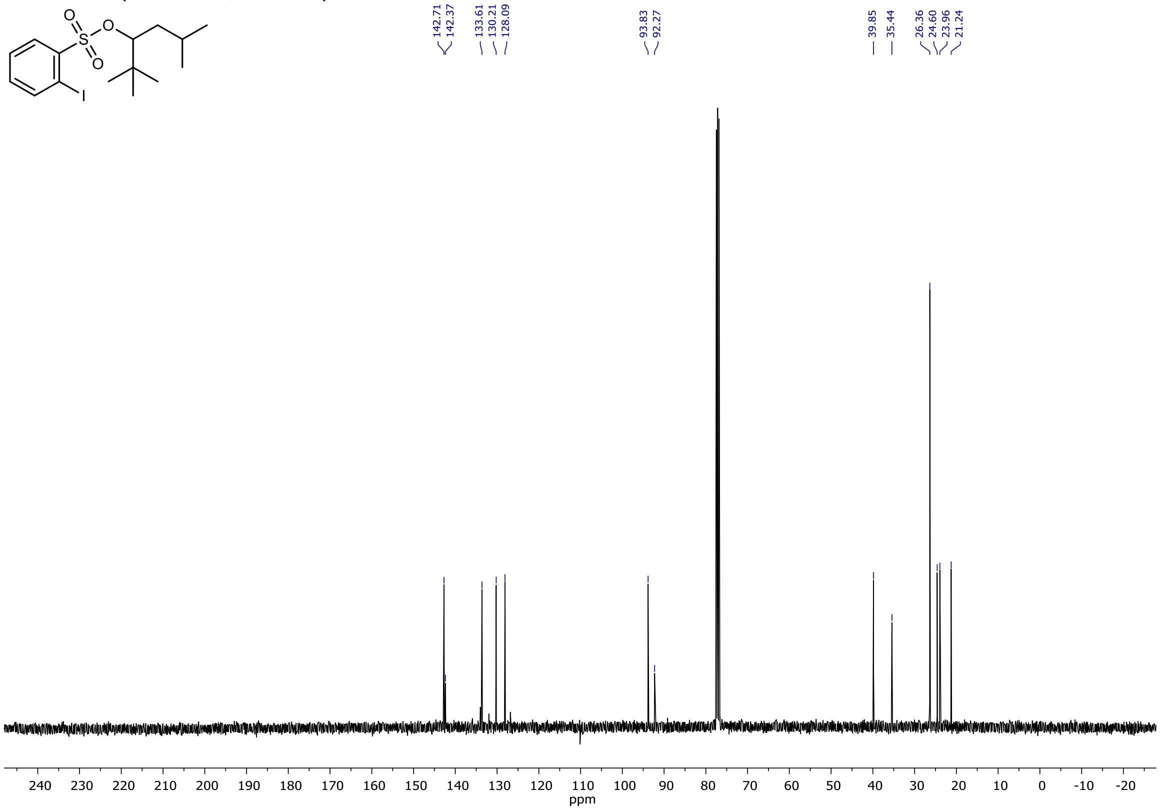

Supplementary Figure 52: NMR Spectra of 2,2,5-Trimethylhexan-3-yl 2-iodobenzenesulfonate (2a-1)

^{1}H NMR (300 MHz, $CDCl_3$)

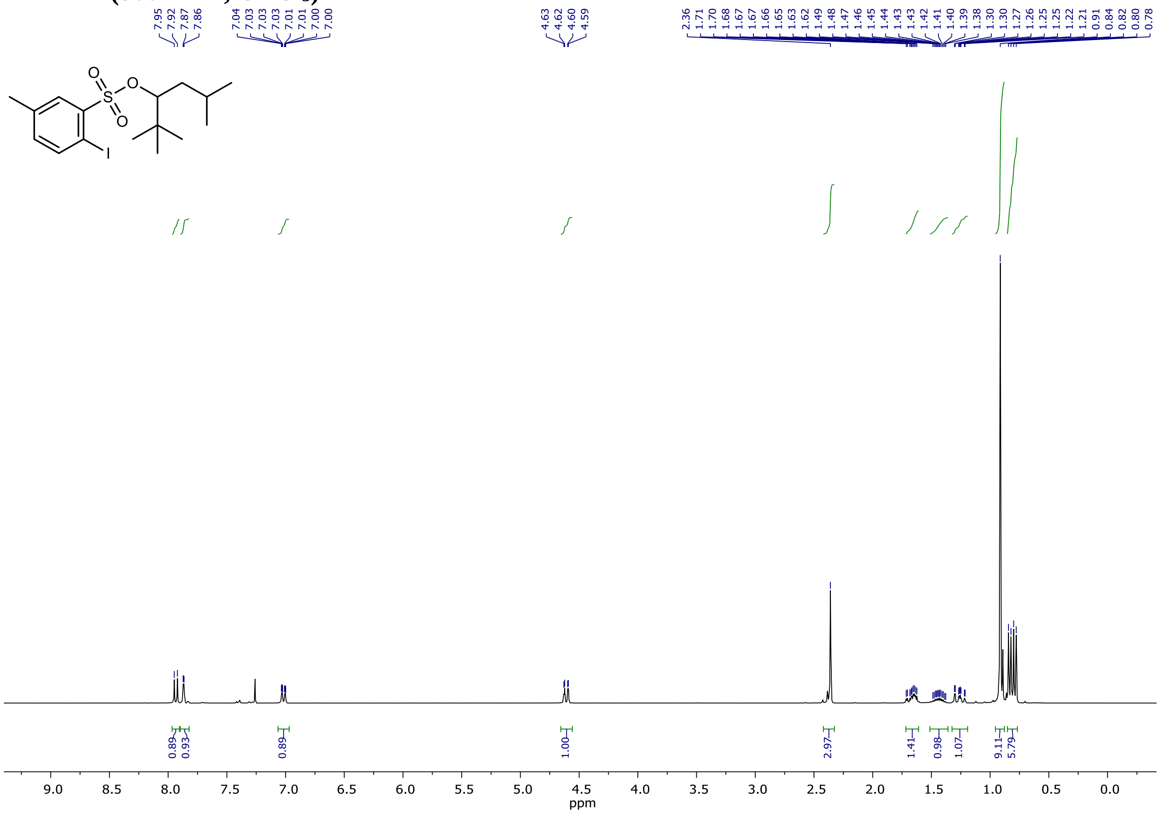

^{13}C NMR (75 MHz, $CDCl_3$)

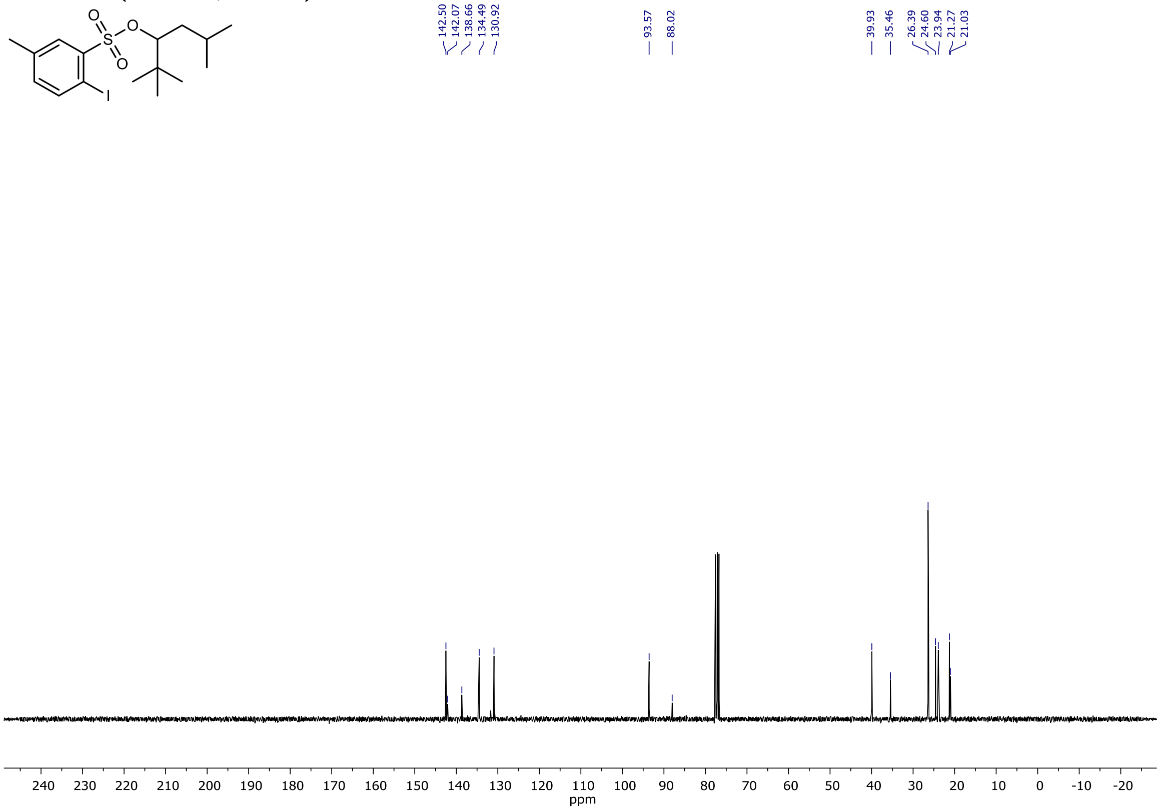

Supplementary Figure 53: NMR Spectra of 2,2,5-Trimethylhexan-3-yl 2-iodo-5-methylbenzenesulfonate (2a-2)

^{1}H NMR (300 MHz, $CDCl_3$)

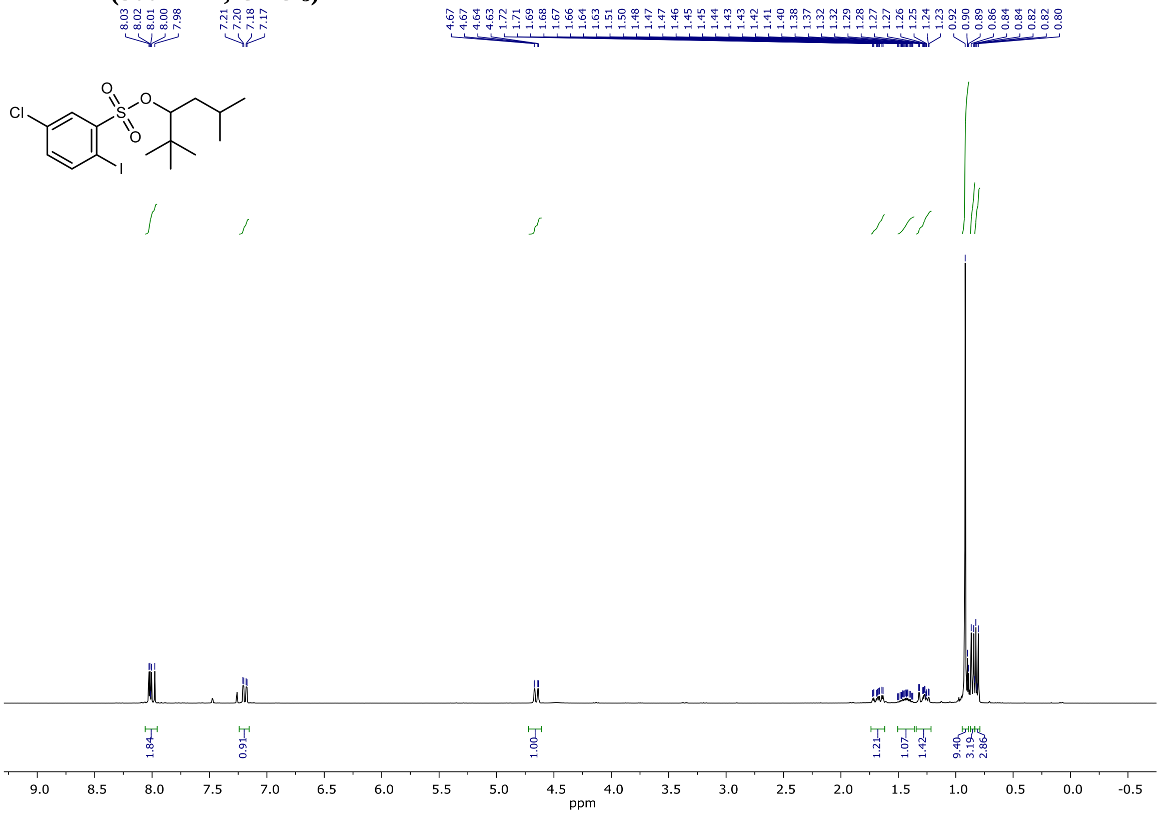

^{13}C NMR (75 MHz, $CDCl_3$)

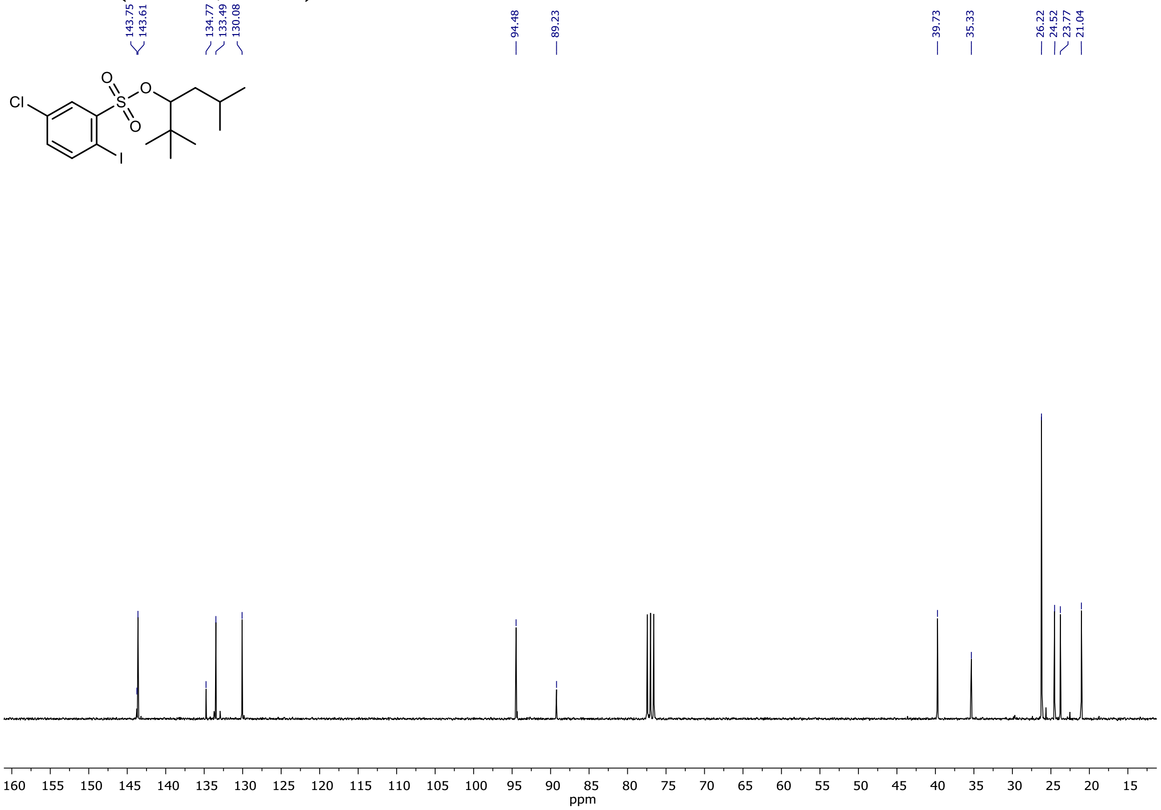

Supplementary Figure 54: NMR Spectra of 2,2,5-Trimethylhexan-3-yl 5-chloro-2-iodobenzenesulfonate (2a-3)

^{1}H NMR (300 MHz, CDCl$_3$)

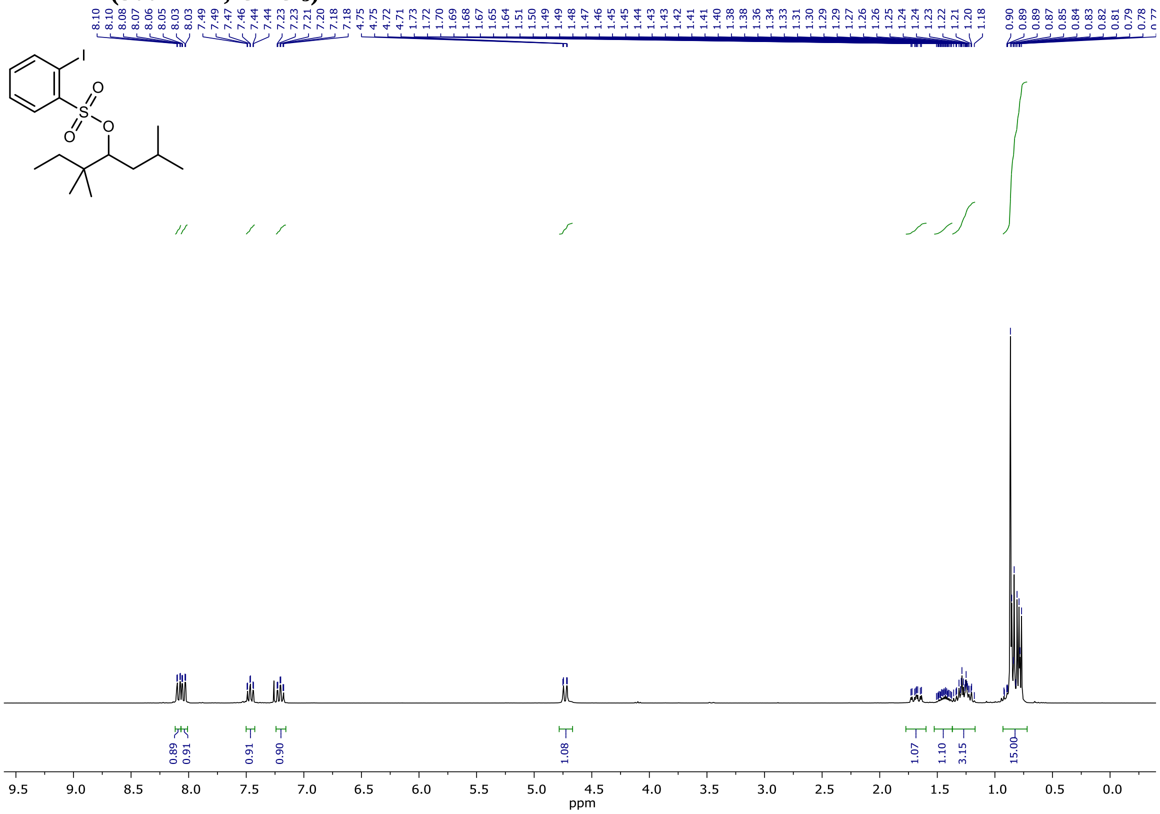

^{13}C NMR (75 MHz, CDCl$_3$)

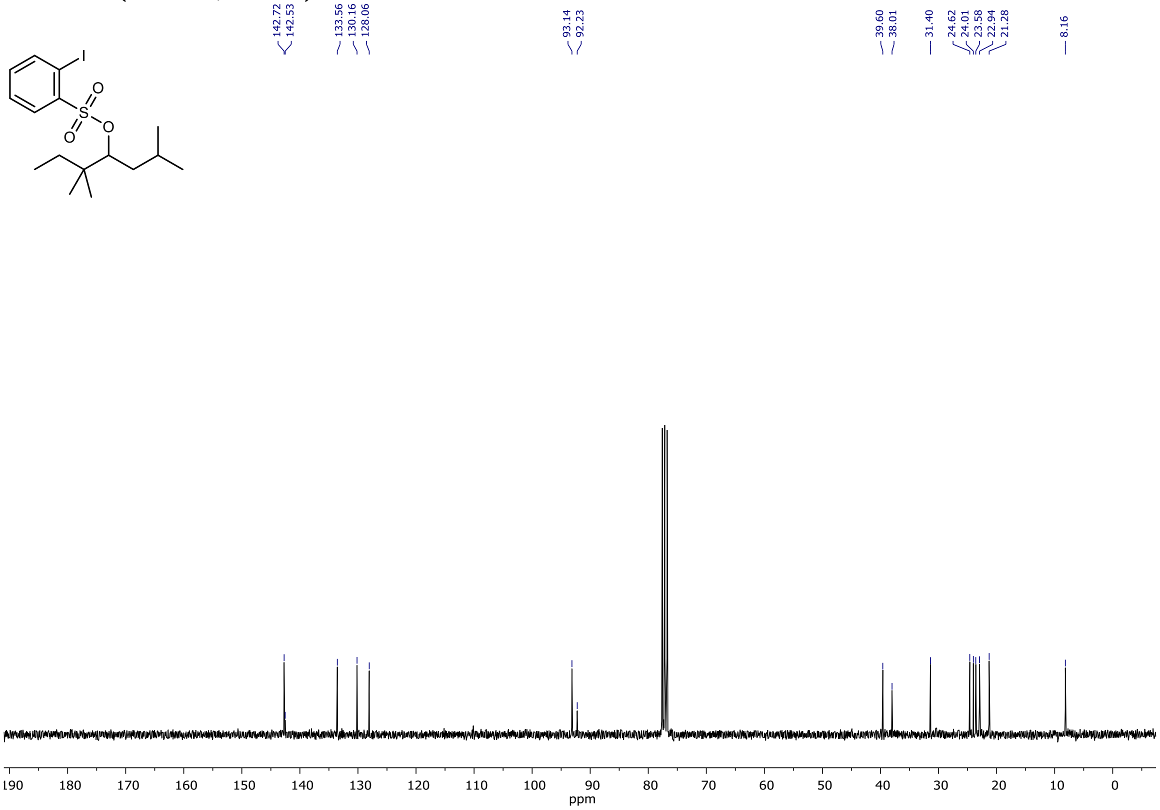

Supplementary Figure 55: NMR Spectra of 2,5,5-Trimethylheptan-4-yl 2-iodobenzenesulfonate (2b)

^{1}H NMR (400 MHz, CDCl$_3$)

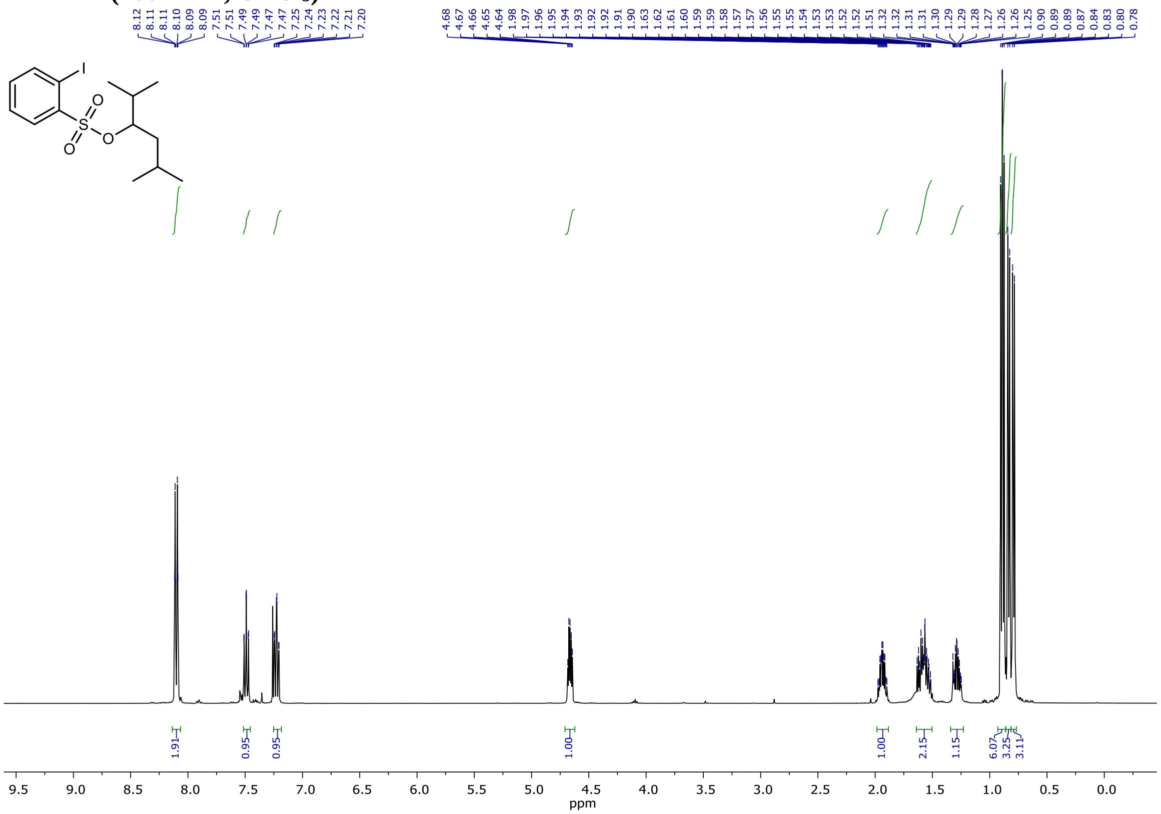

^{13}C NMR (101 MHz, CDCl$_3$)

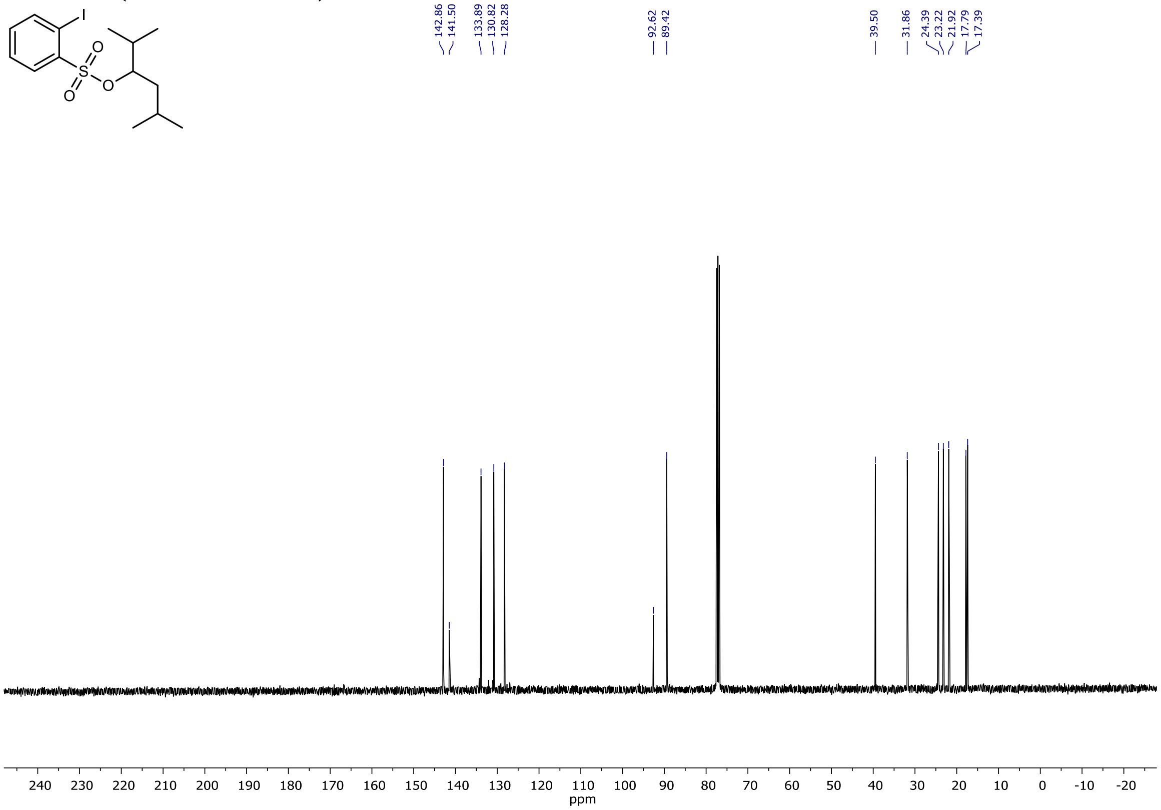

Supplementary Figure 56: NMR Spectra of 2,5-Dimethylhexan-3-yl 2-iodobenzenesulfonate (2c)

^{1}H NMR (300 MHz, CDCl$_3$)

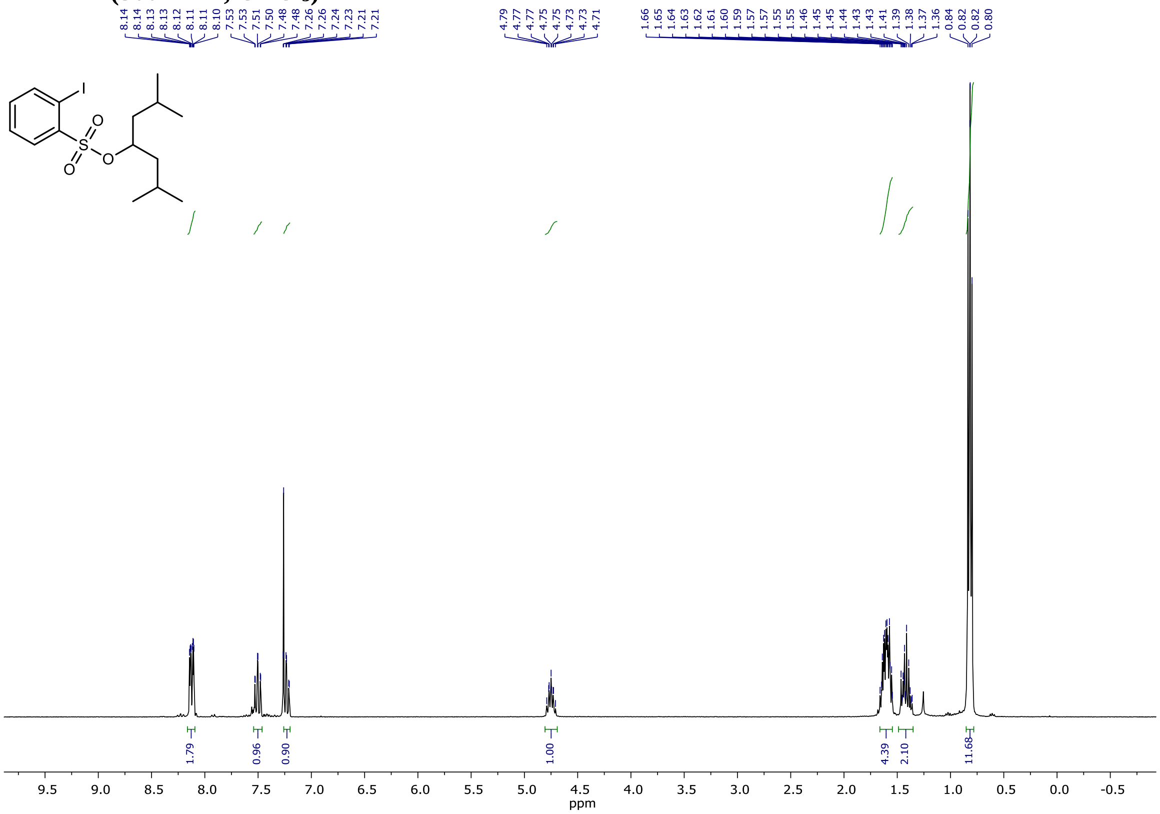

^{13}C NMR (75 MHz, CDCl$_3$)

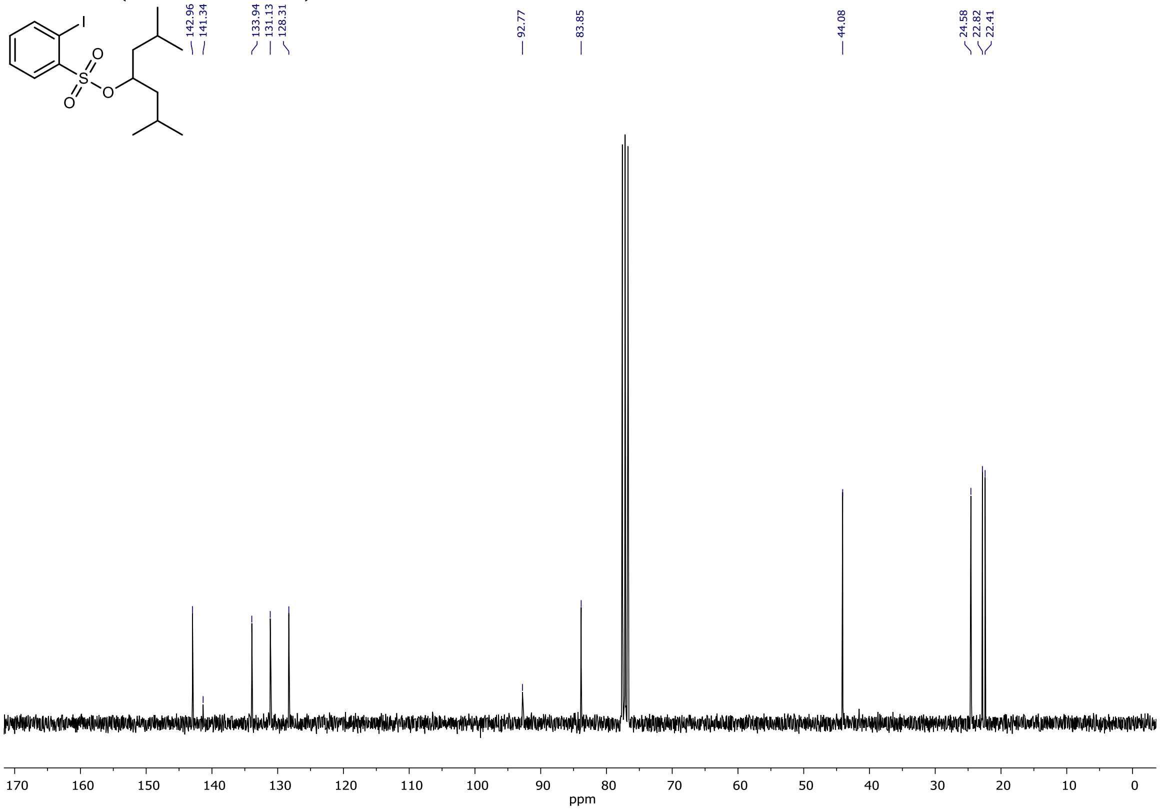

Supplementary Figure 57: NMR Spectra of 2,6-Dimethylheptan-4-yl 2-iodobenzenesulfonate (2d)

^{1}H NMR (400 MHz, $CDCl_3$)

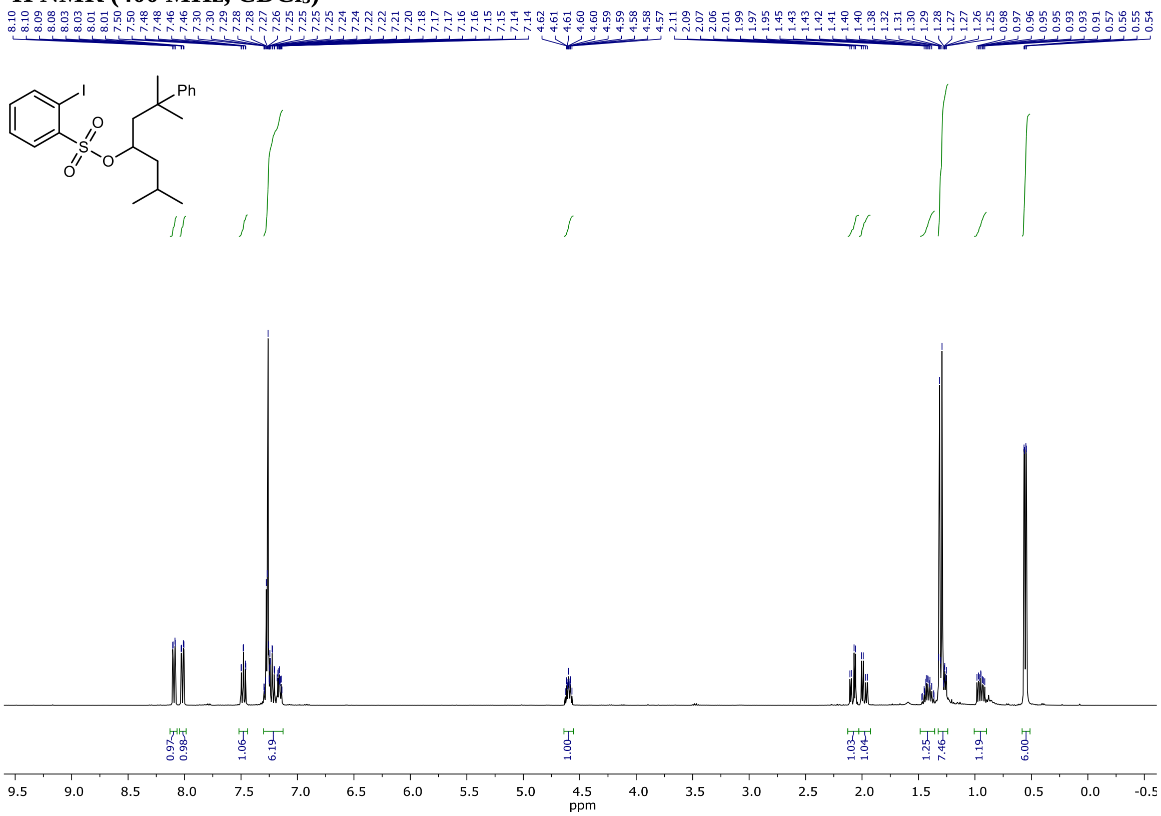

^{13}C NMR (101 MHz, $CDCl_3$)

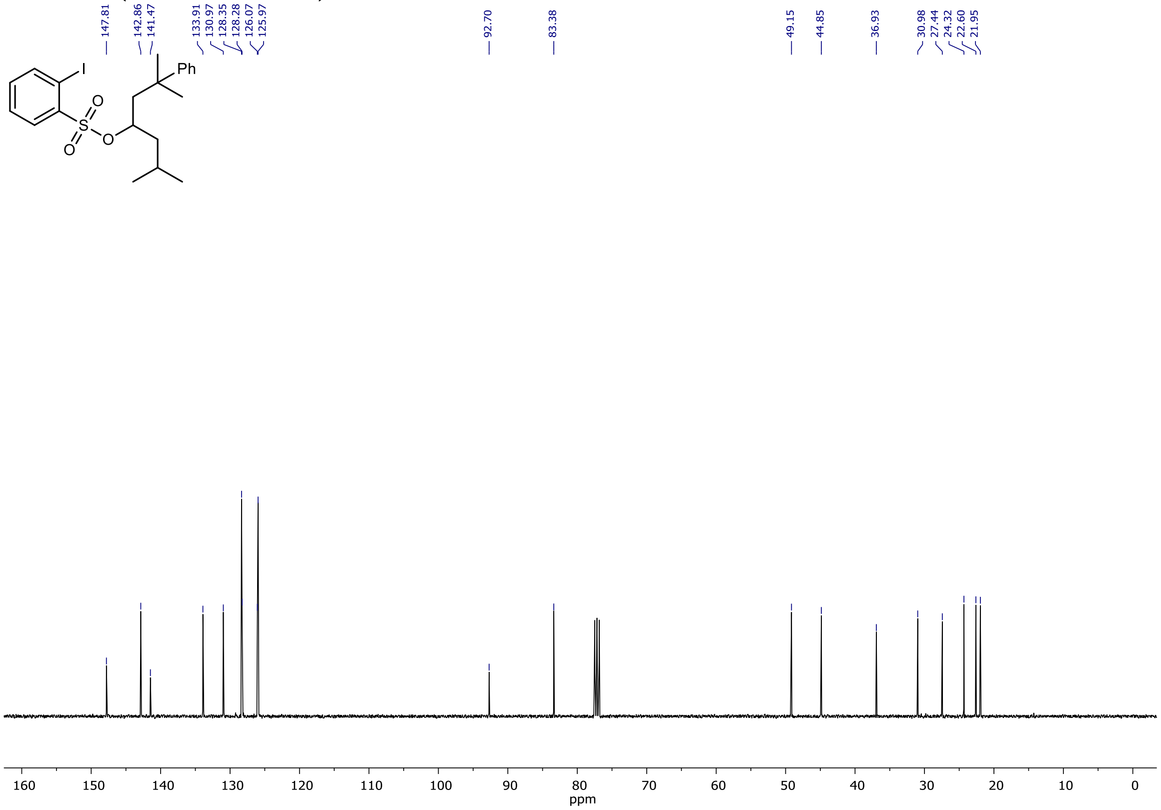

Supplementary Figure 58: NMR Spectra of 2,6-Dimethyl-2-phenylheptan-4-yl 2-iodobenzenesulfonate (2e)

^{1}H NMR (300 MHz, $CDCl_3$)

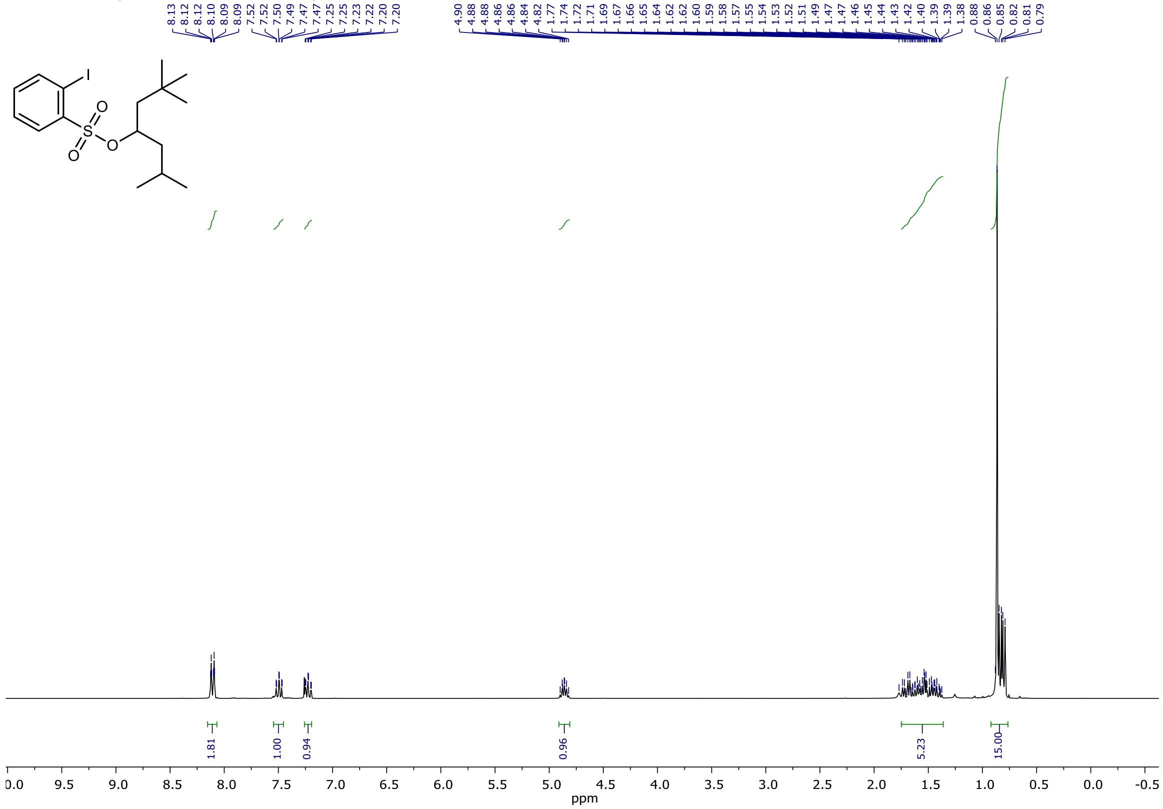

^{13}C NMR (75 MHz, $CDCl_3$)

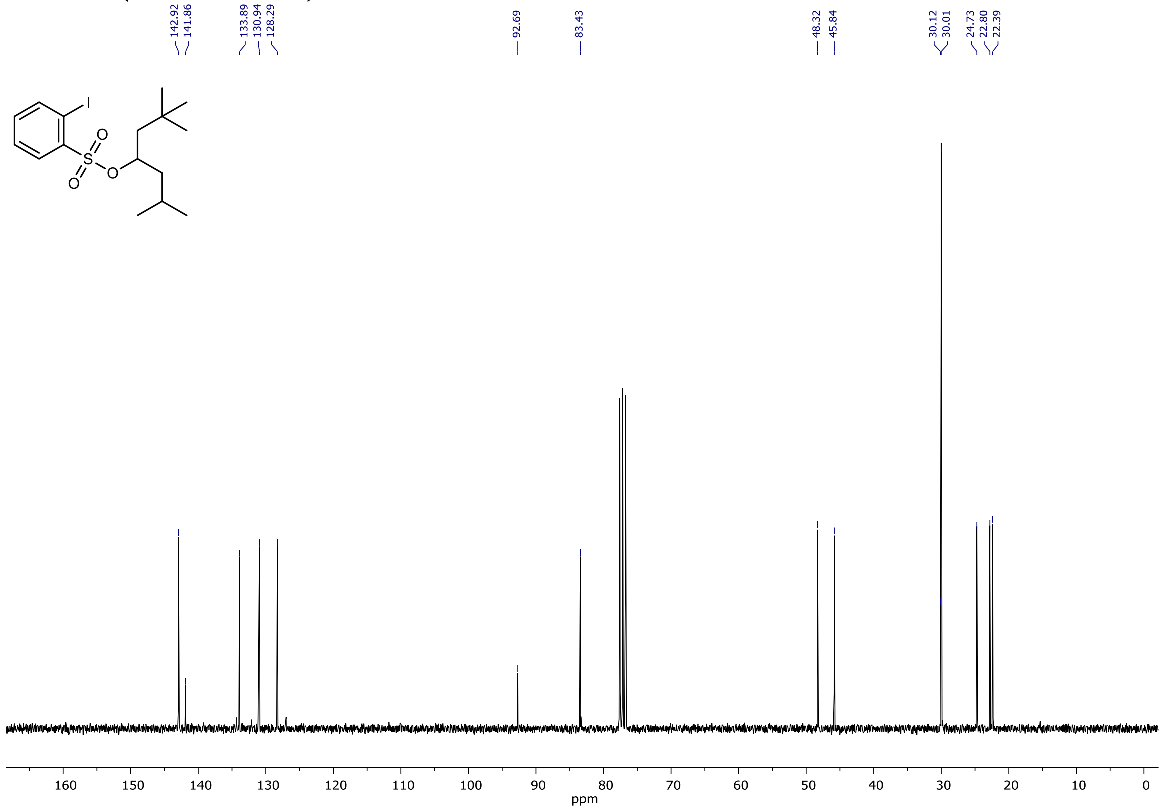

Supplementary Figure 59: NMR Spectra of 2,2,6-Trimethylheptan-4-yl 2-iodobenzenesulfonate (2f)

^{1}H NMR (300 MHz, CDCl3)

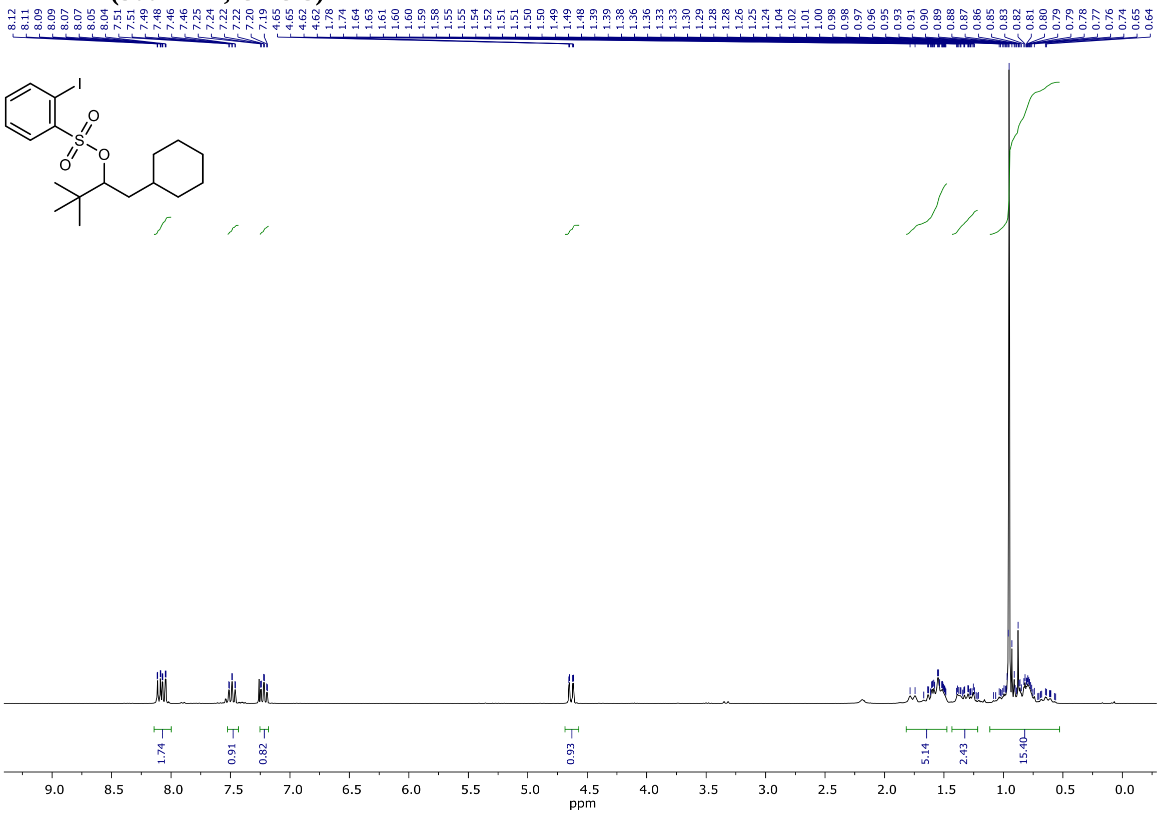

^{13}C NMR (75 MHz, $CDCl_3$)

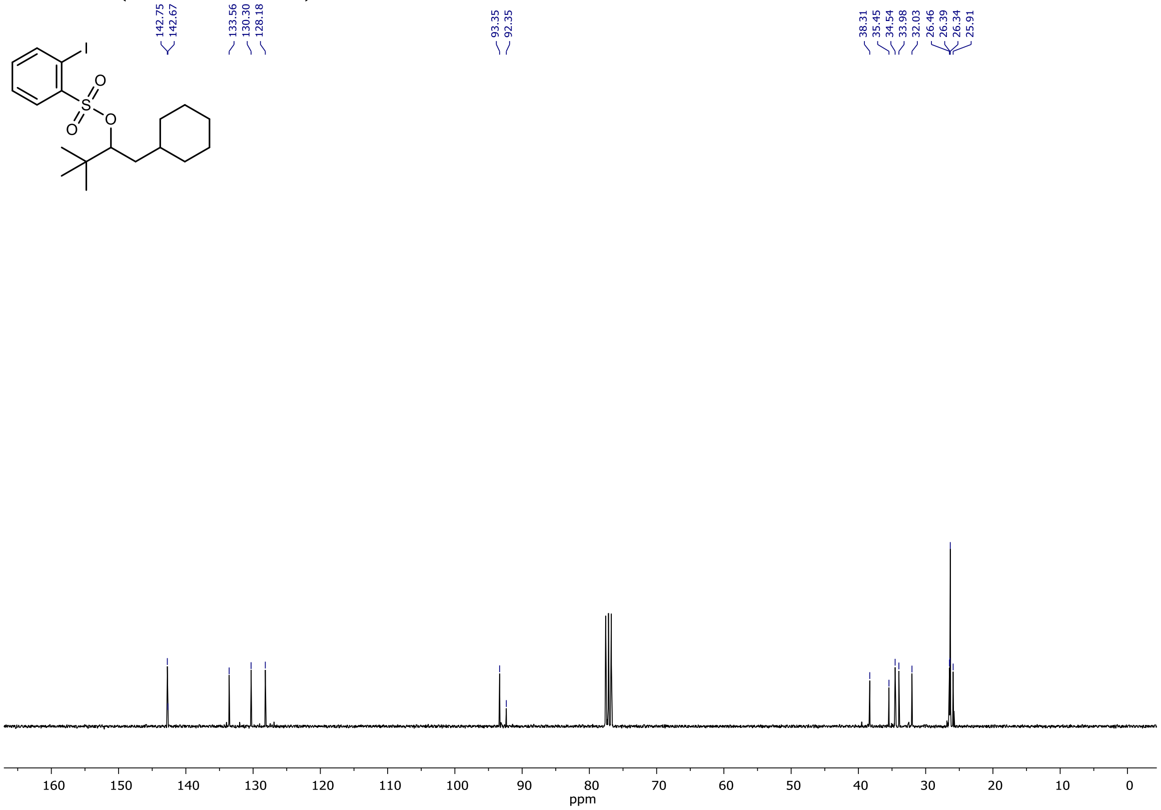

Supplementary Figure 60: NMR Spectra of 1-Cyclohexyl-3,3-dimethylbutan-2-yl 2-iodobenzenesulfonate (2g)

^{1}H NMR (300 MHz, $CDCl_3$)

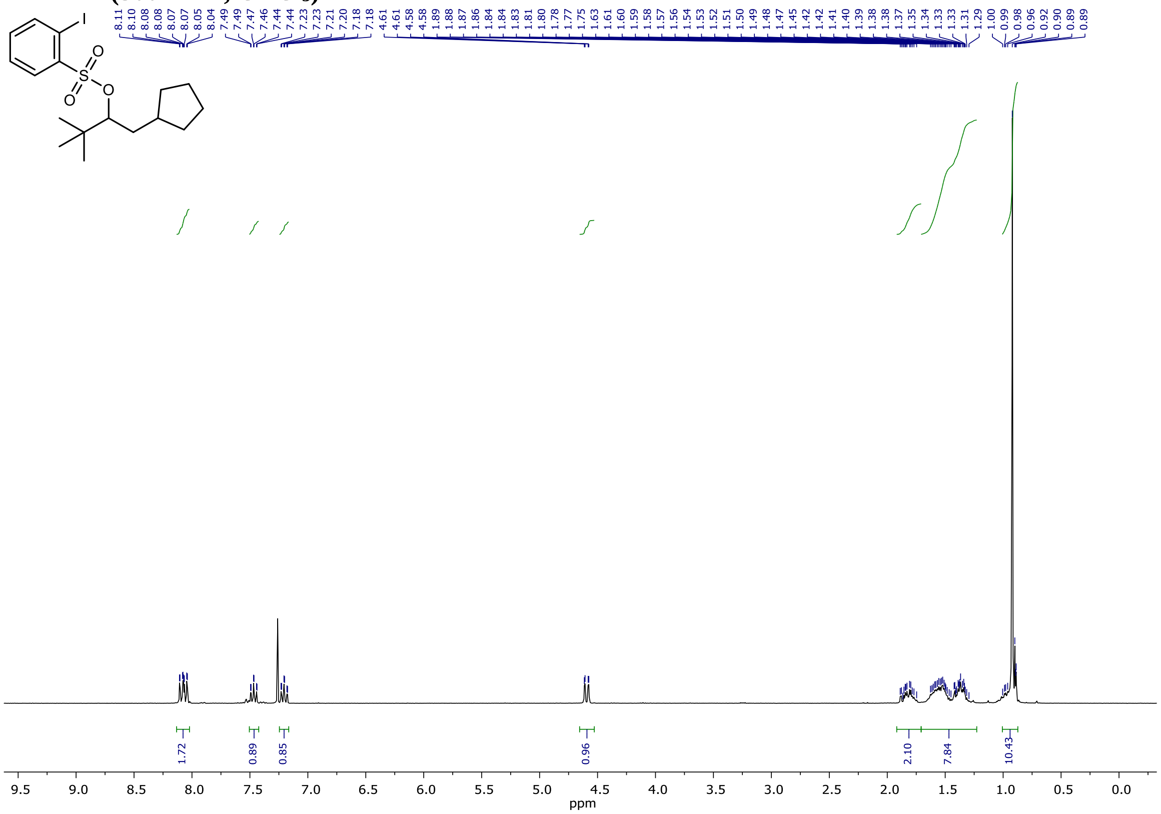

^{13}C NMR (75 MHz, $CDCl_3$)

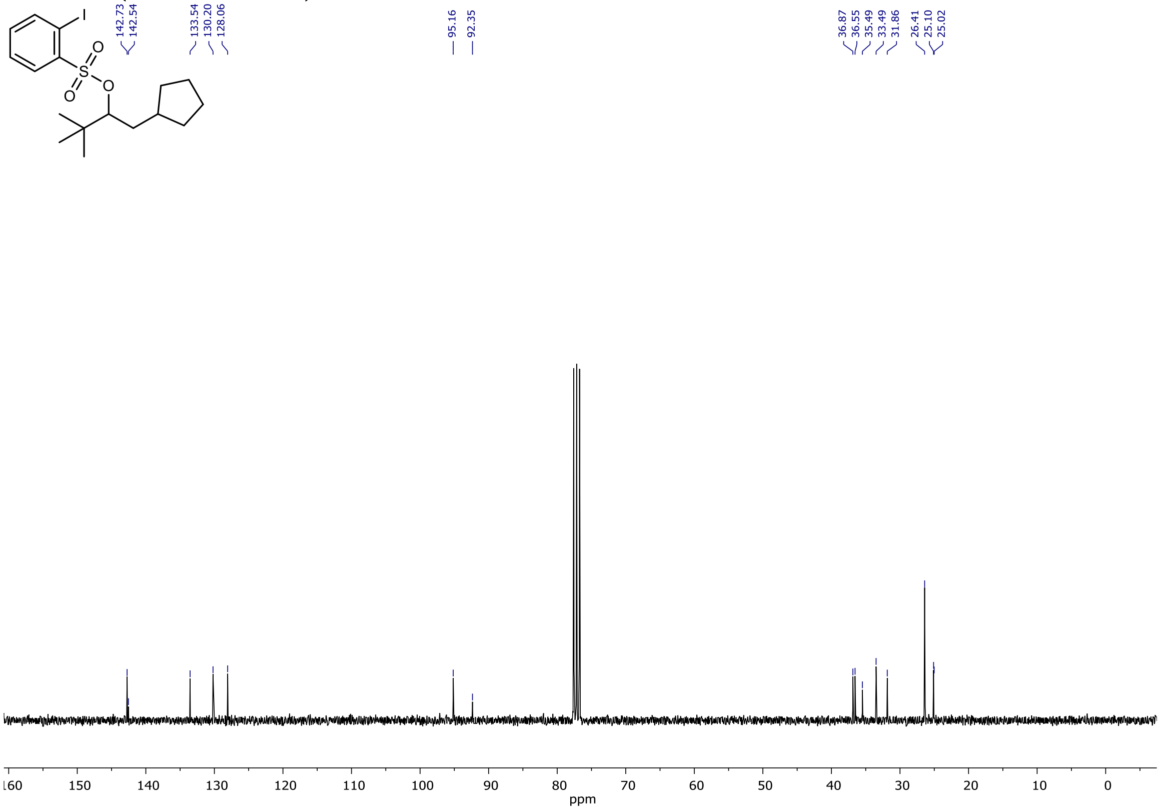

Supplementary Figure 61: NMR Spectra of 1-Cyclopentyl-3,3-dimethylbutan-2-yl 2-iodobenzenesulfonate (2h)

^{1}H NMR (300 MHz, $CDCl_3$)

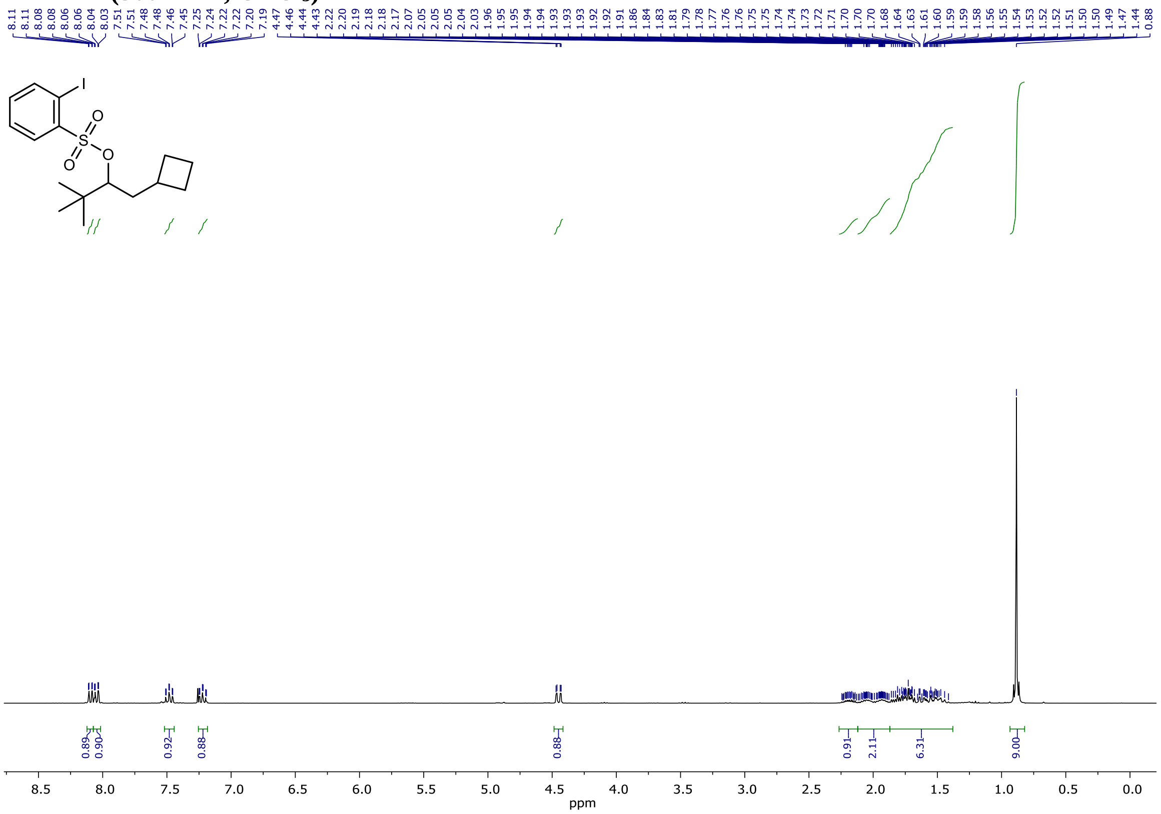

^{13}C NMR (75 MHz, $CDCl_3$)

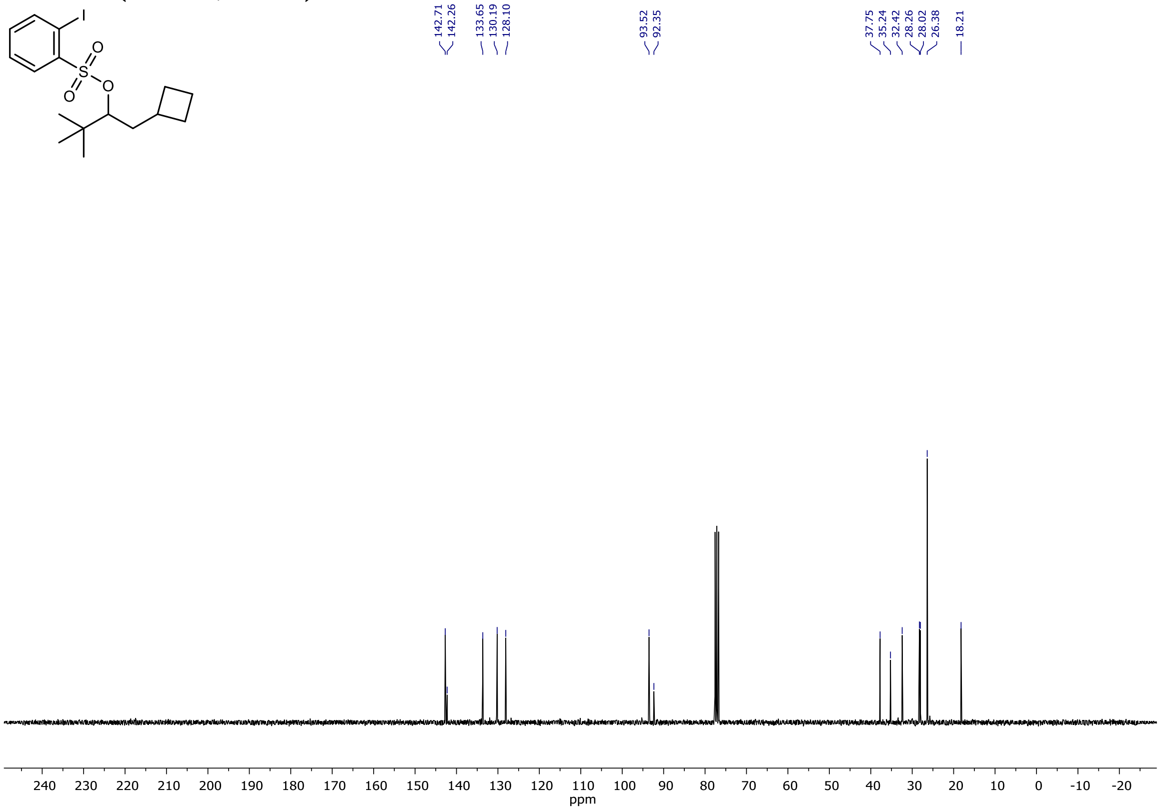

Supplementary Figure 62: NMR Spectra of 1-Cyclobutyl-3,3-dimethylbutan-2-yl 2-iodobenzenesulfonate (2i-1)

^{1}H NMR (300 MHz, $CDCl_3$)

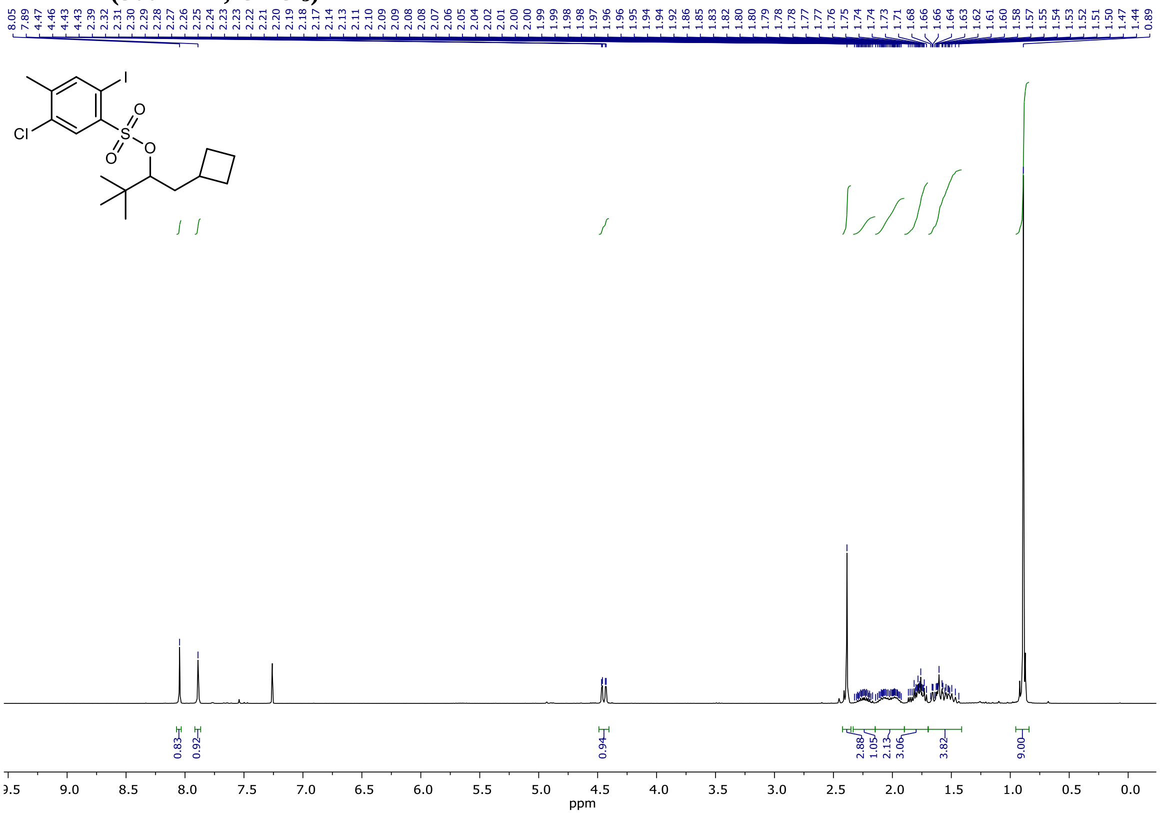

^{13}C NMR (75 MHz, $CDCl_3$)

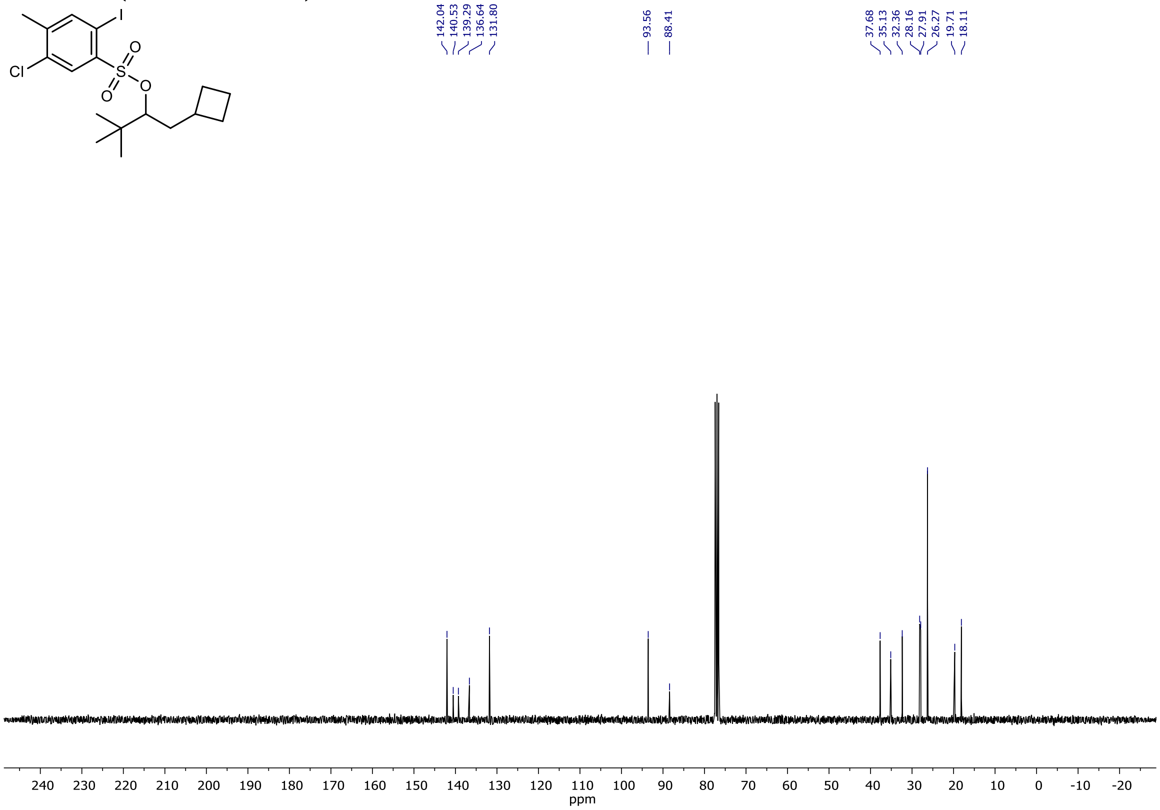

Supplementary Figure 63: NMR Spectra of 1-Cyclobutyl-3,3-dimethylbutan-2-yl 5-chloro-2-iodo-4-methylbenzenesulfonate (2i-2)

^{1}H NMR (400 MHz, $CDCl_3$)

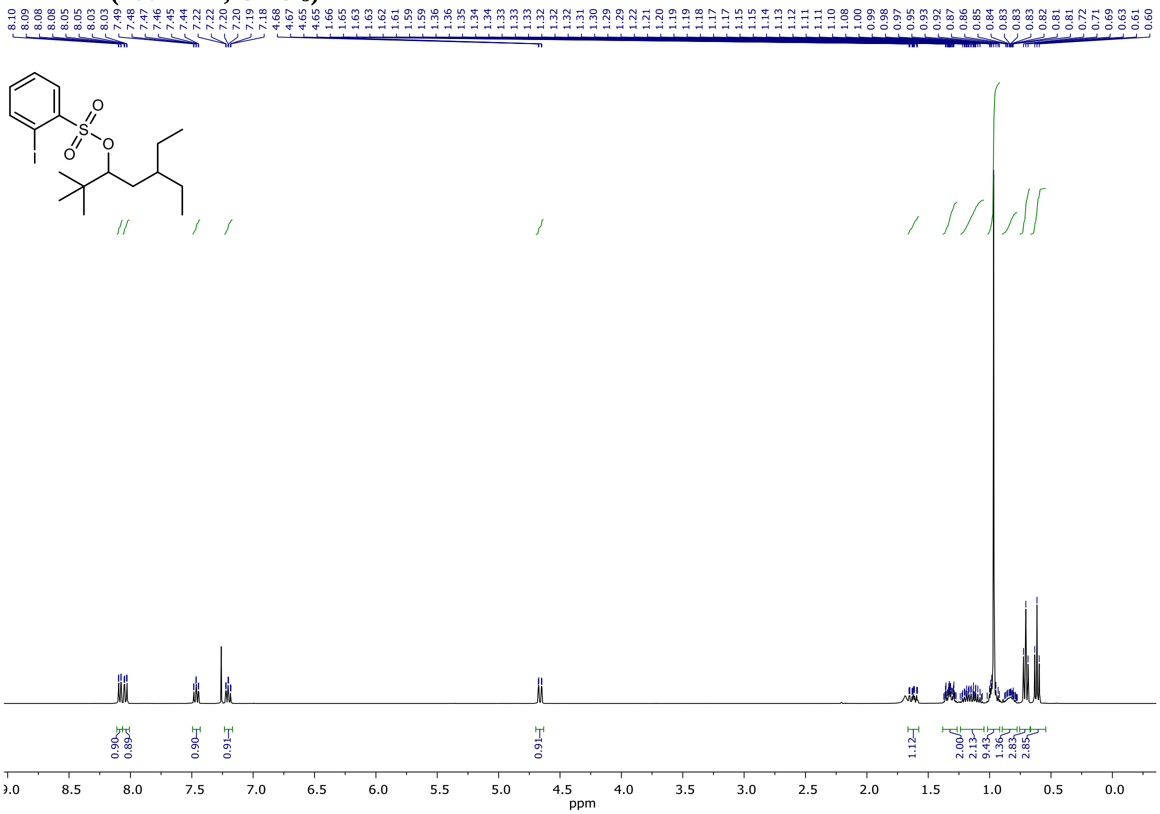

^{13}C NMR (101 MHz, $CDCl_3$)

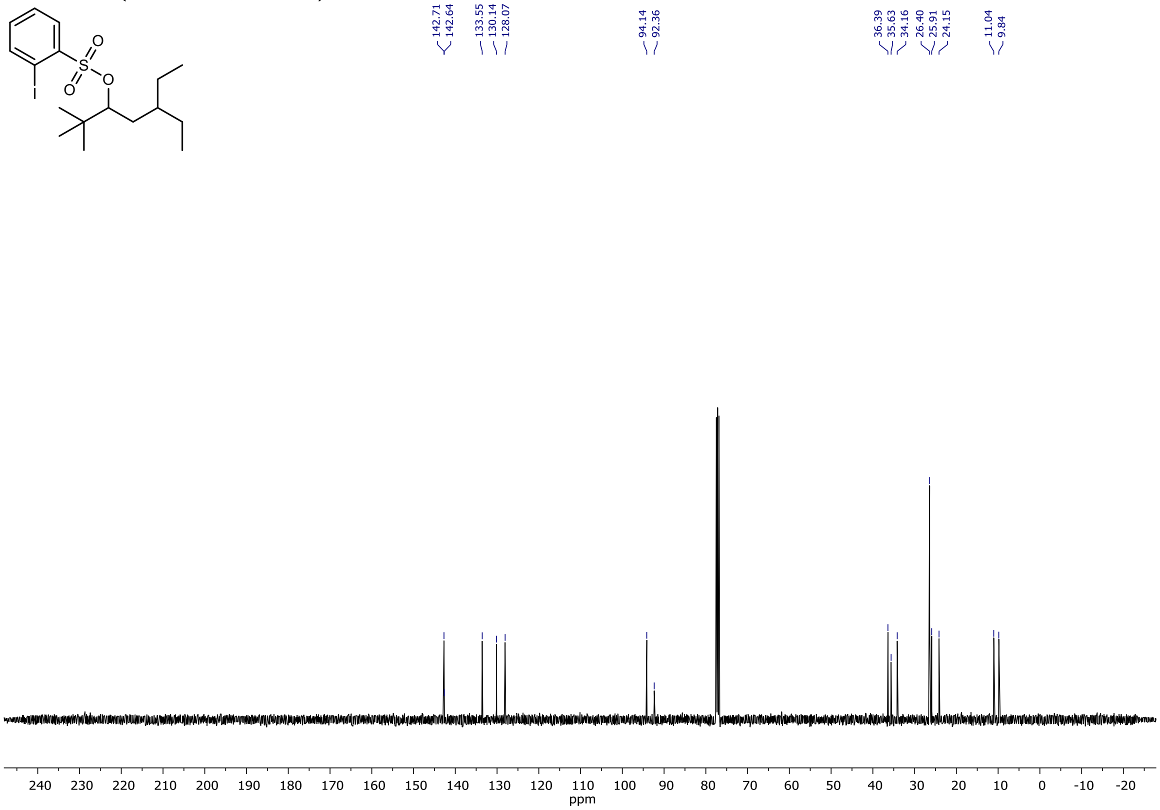

Supplementary Figure 64: NMR Spectra of 5-Ethyl-2,2-dimethylheptan-3-yl 2-iodobenzenesulfonate (2j)

^{1}H NMR (300 MHz, $CDCl_3$)

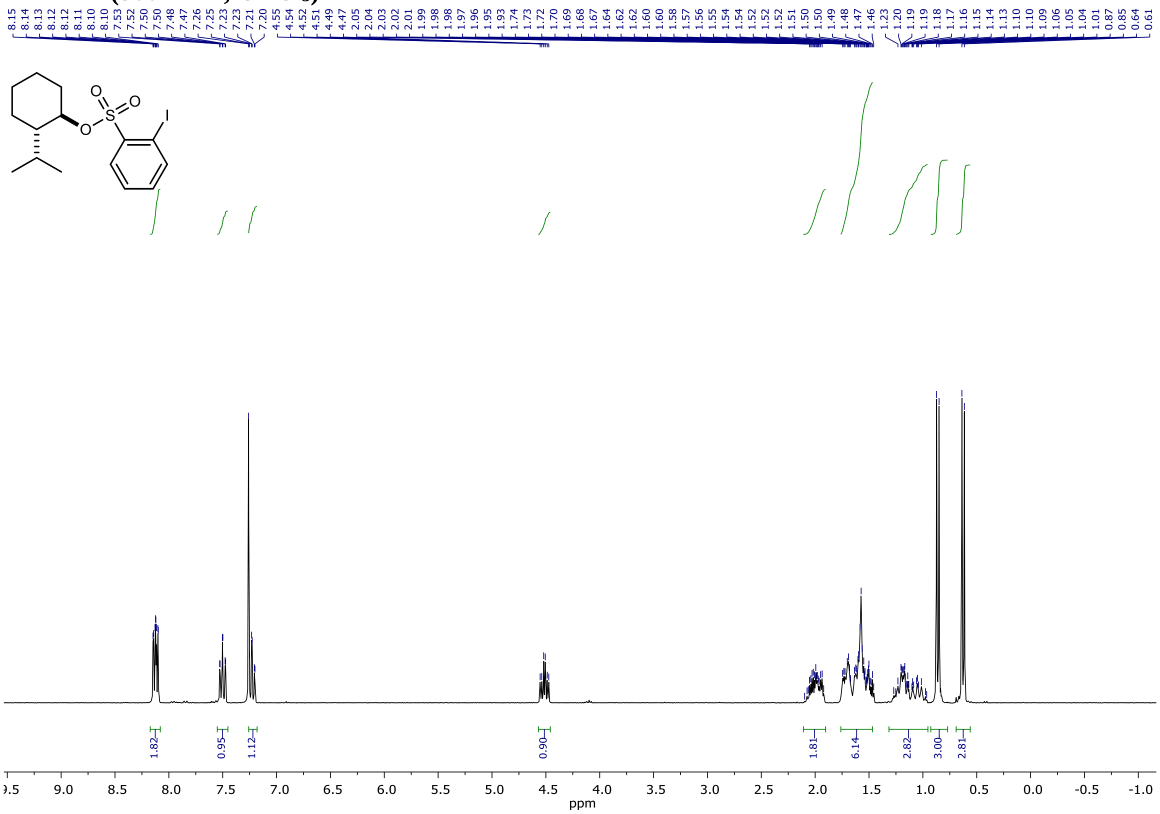

^{13}C NMR (75 MHz, $CDCl_3$)

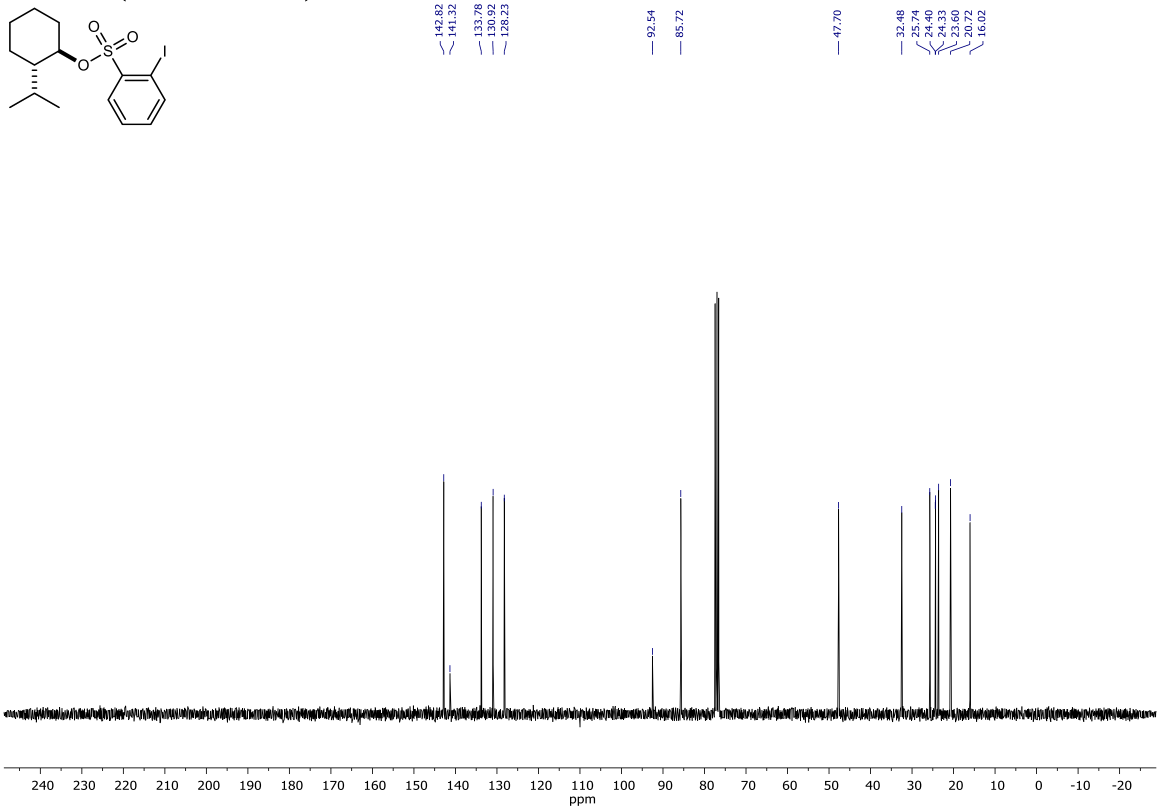

Supplementary Figure 65: NMR Spectra of *trans*-2-Isopropylcyclohexyl 2-iodobenzenesulfonate (2k)

^{1}H NMR (300 MHz, $CDCl_3$)

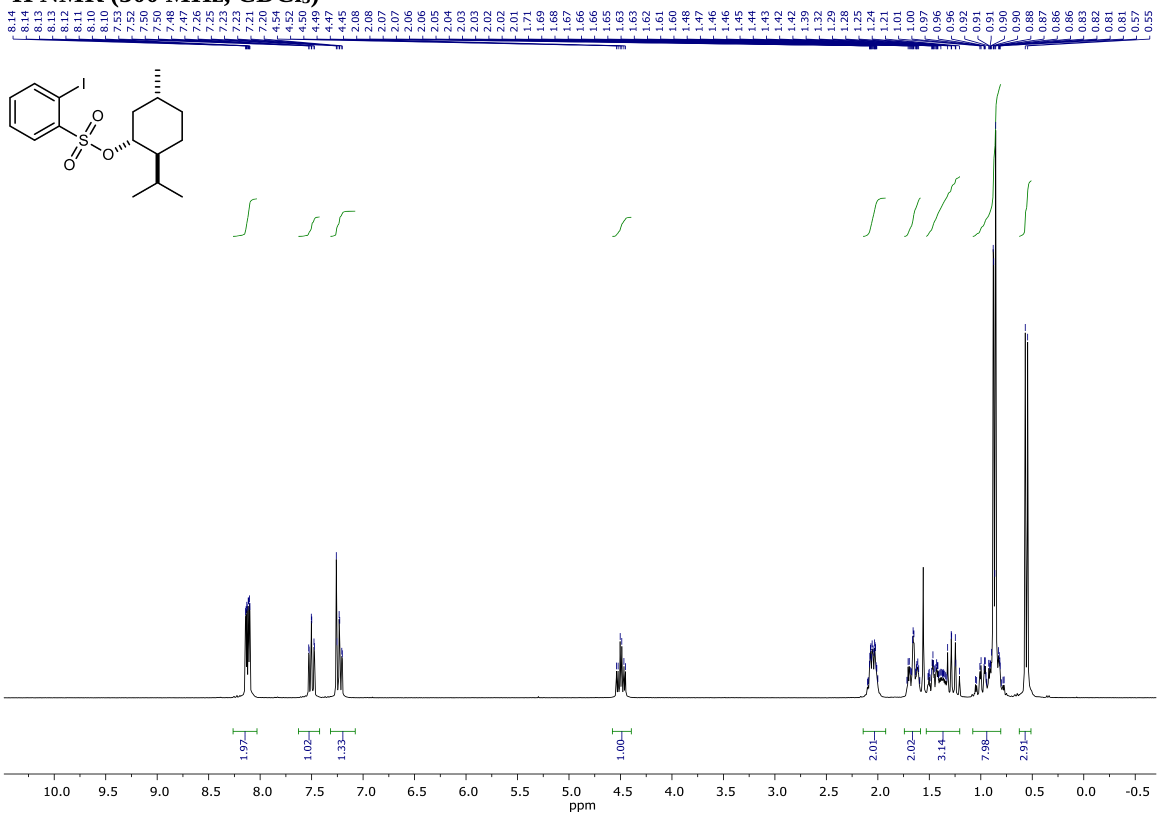

^{13}C NMR (75 MHz, $CDCl_3$)

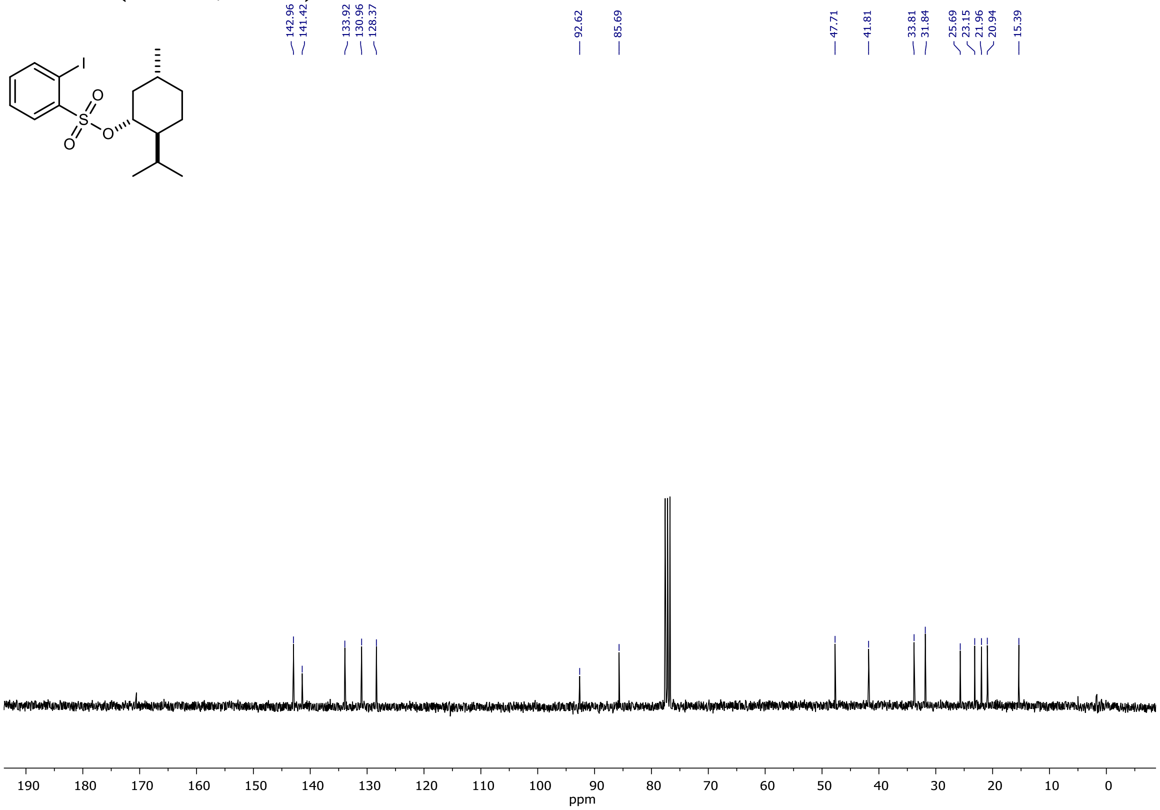

Supplementary Figure 66: NMR Spectra of (1*R*,2*S*,5*R*)-2-Isopropyl-5-methylcyclohexyl 2-iodobenzenesulfonate (2l-1)

^{1}H NMR (300 MHz, $CDCl_3$)

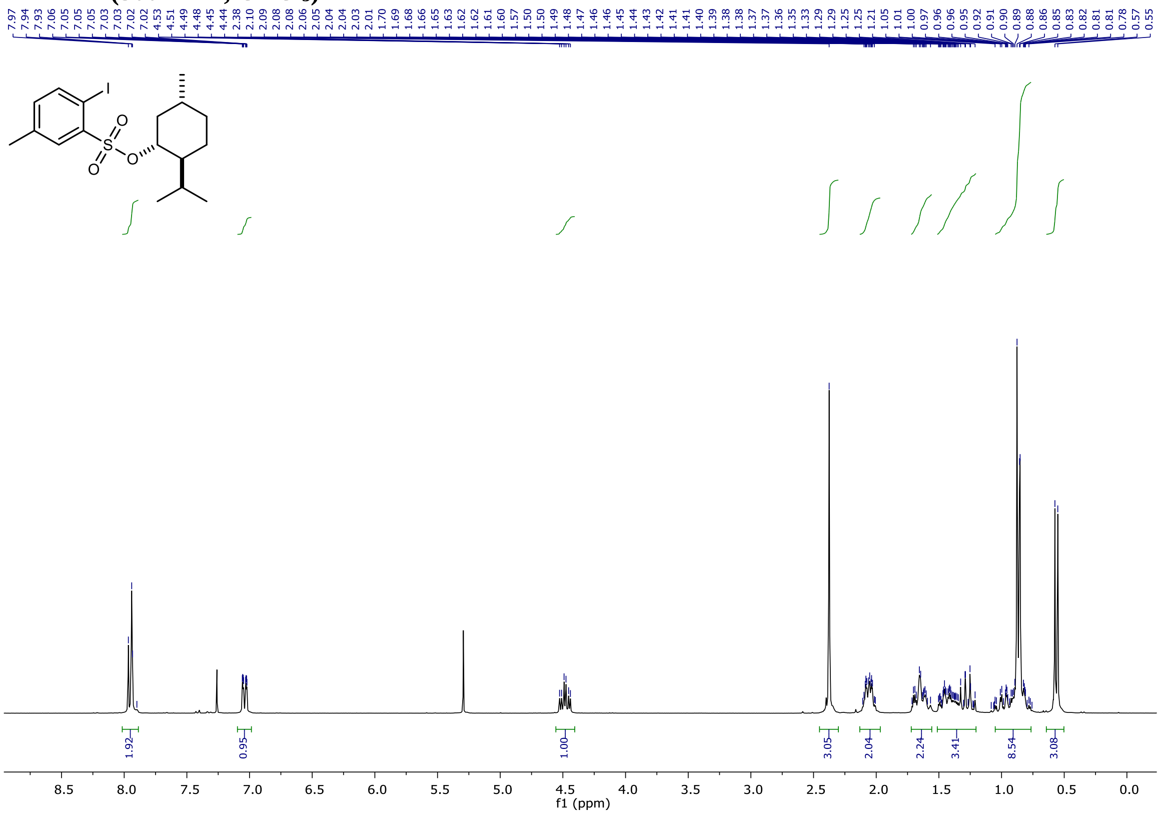

^{13}C NMR (75 MHz, $CDCl_3$)

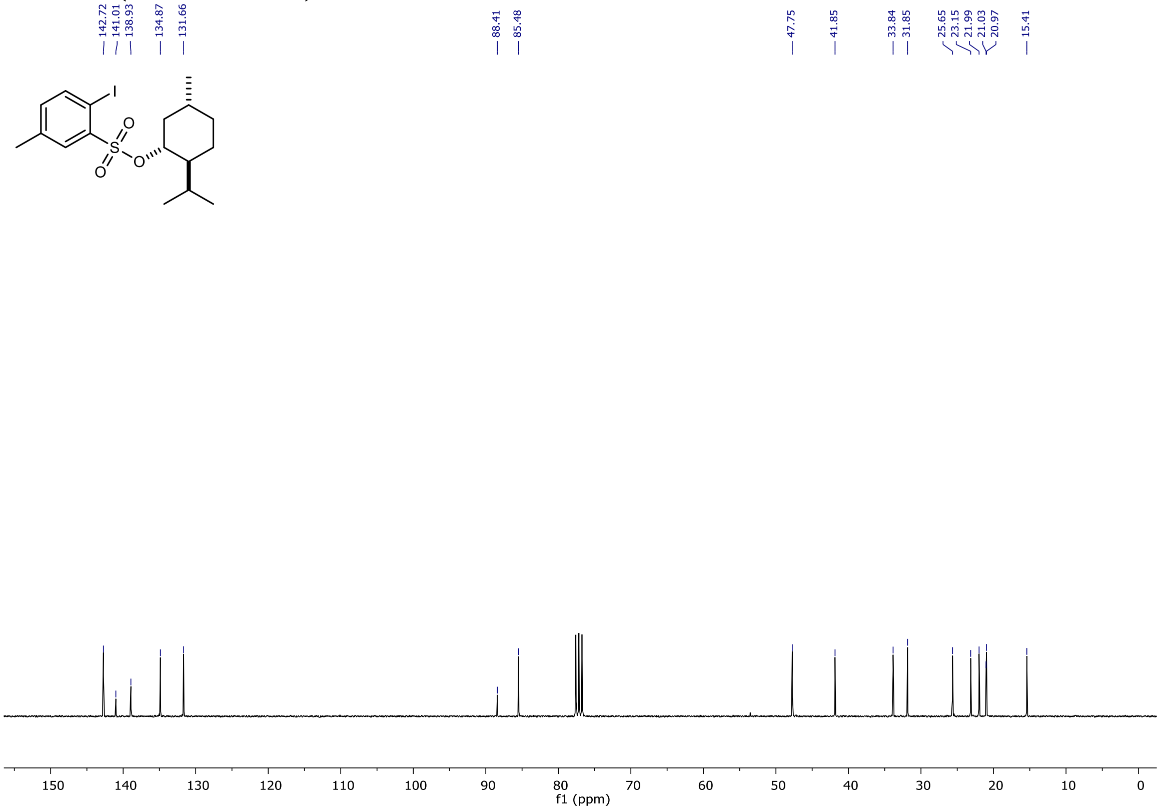

Supplementary Figure 67: NMR Spectra of (1*R*,2*S*,5*R*)-2-Isopropyl-5-methylcyclohexyl 2-iodo-5-methylbenzenesulfonate (2l-2)

^{1}H NMR (300 MHz, CDCl$_3$)

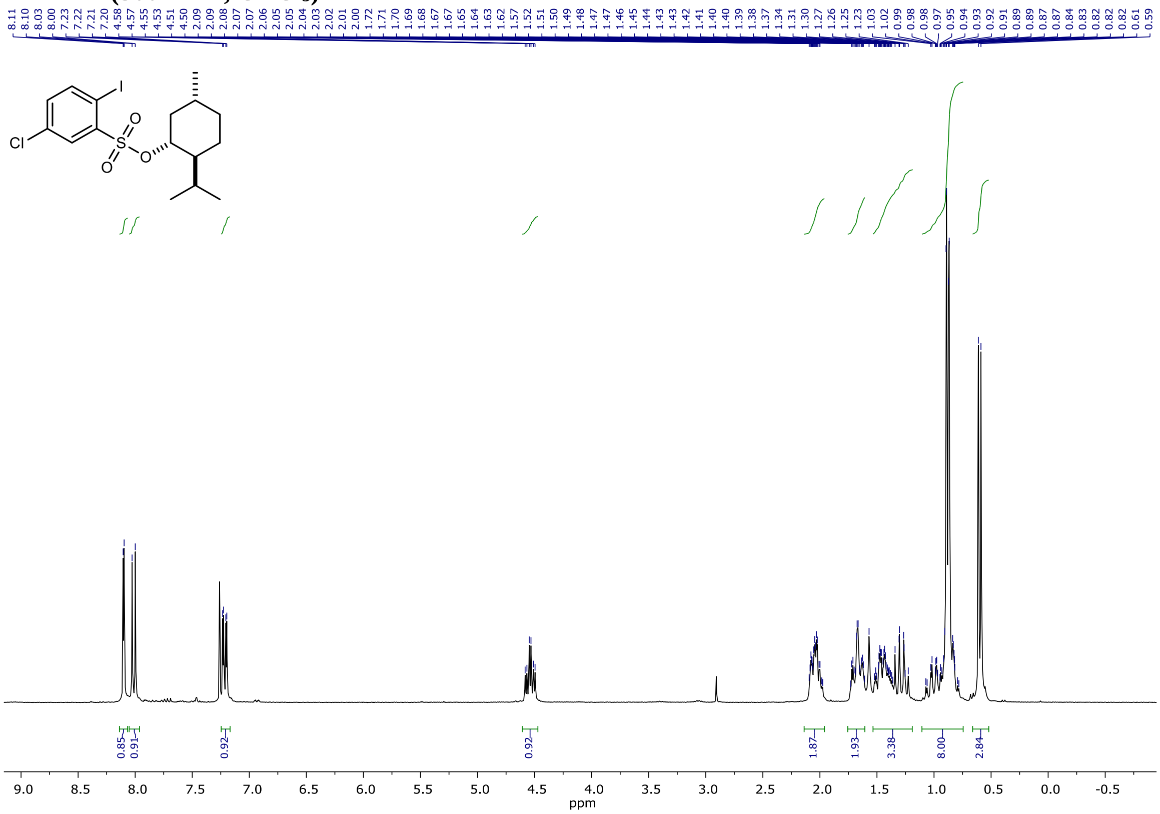

^{13}C NMR (75 MHz, CDCl$_3$)

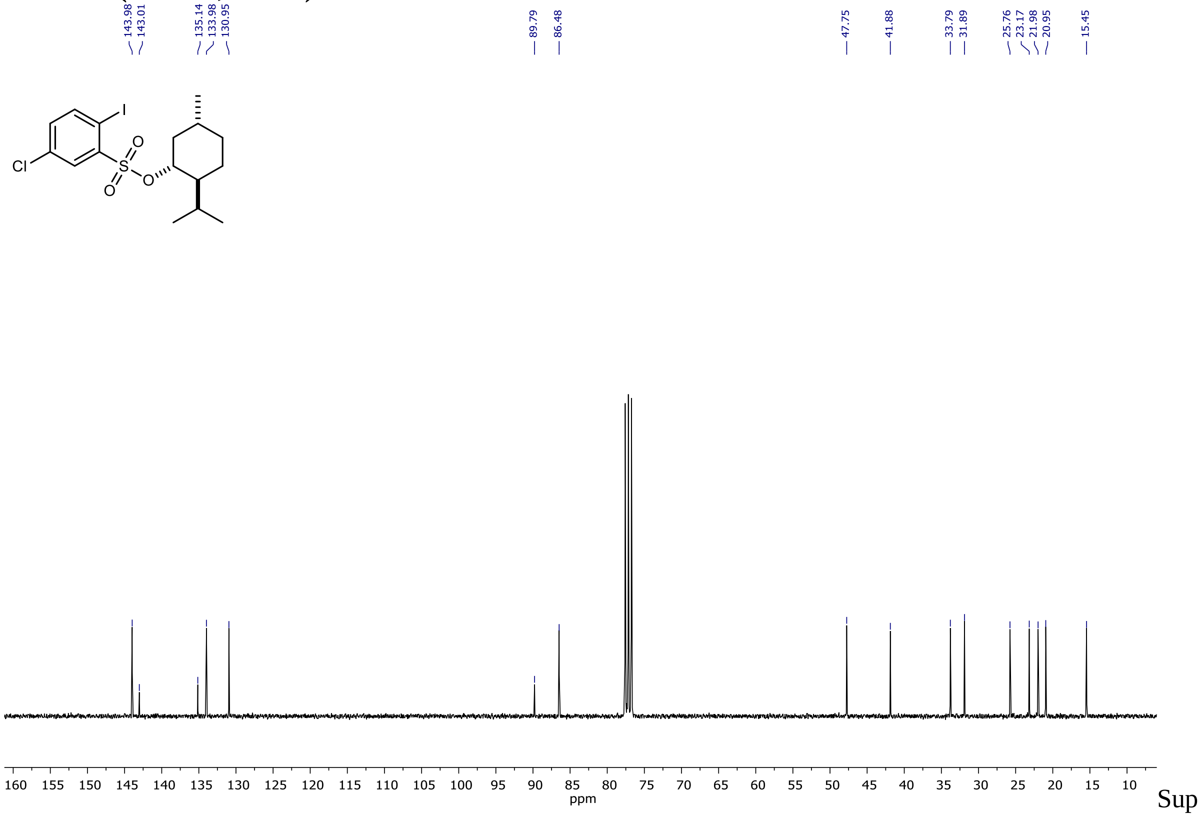

Sup

NMR Spectra of plementary Figure 68: NMR Spectra of (1*R*,2*S*,5*R*)-2-Isopropyl-5-methylcyclohexyl 5-chloro-2-iodobenzenesulfonate (2l-3)

^{1}H NMR (300 MHz, $CDCl_3$)

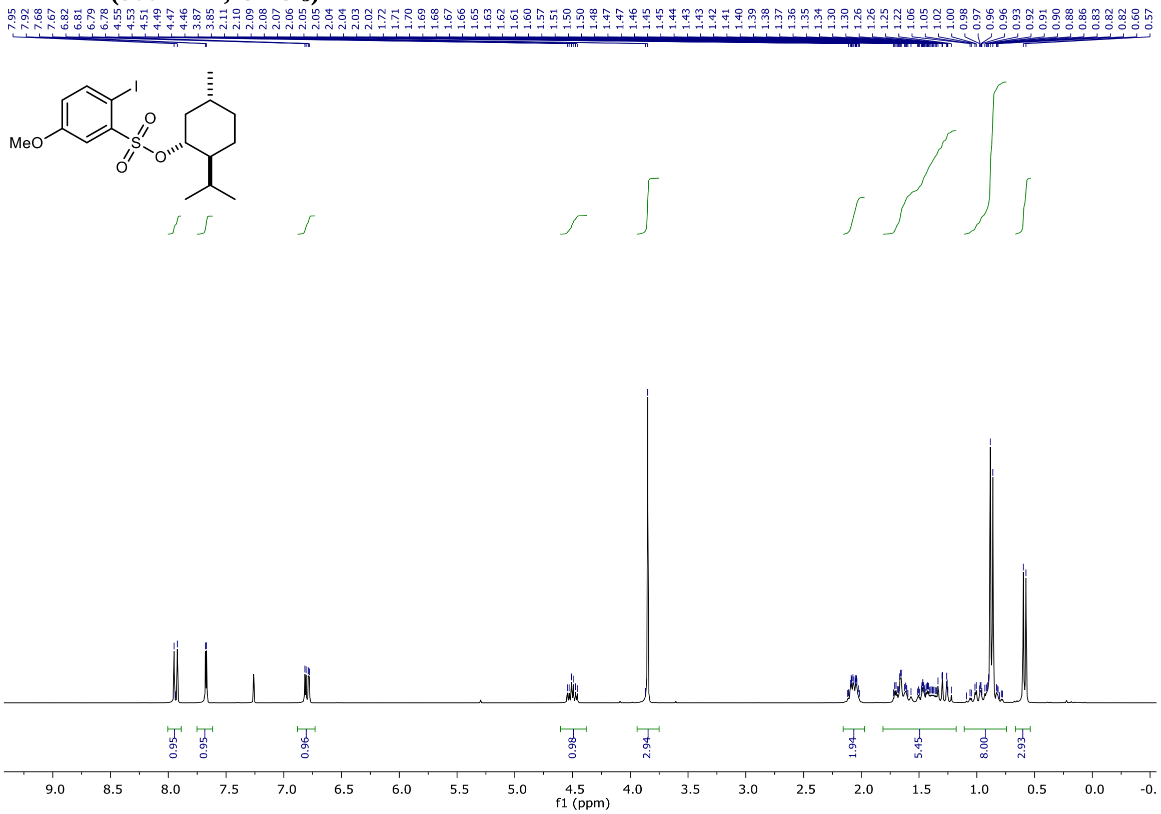

^{13}C NMR (75 MHz, $CDCl_3$

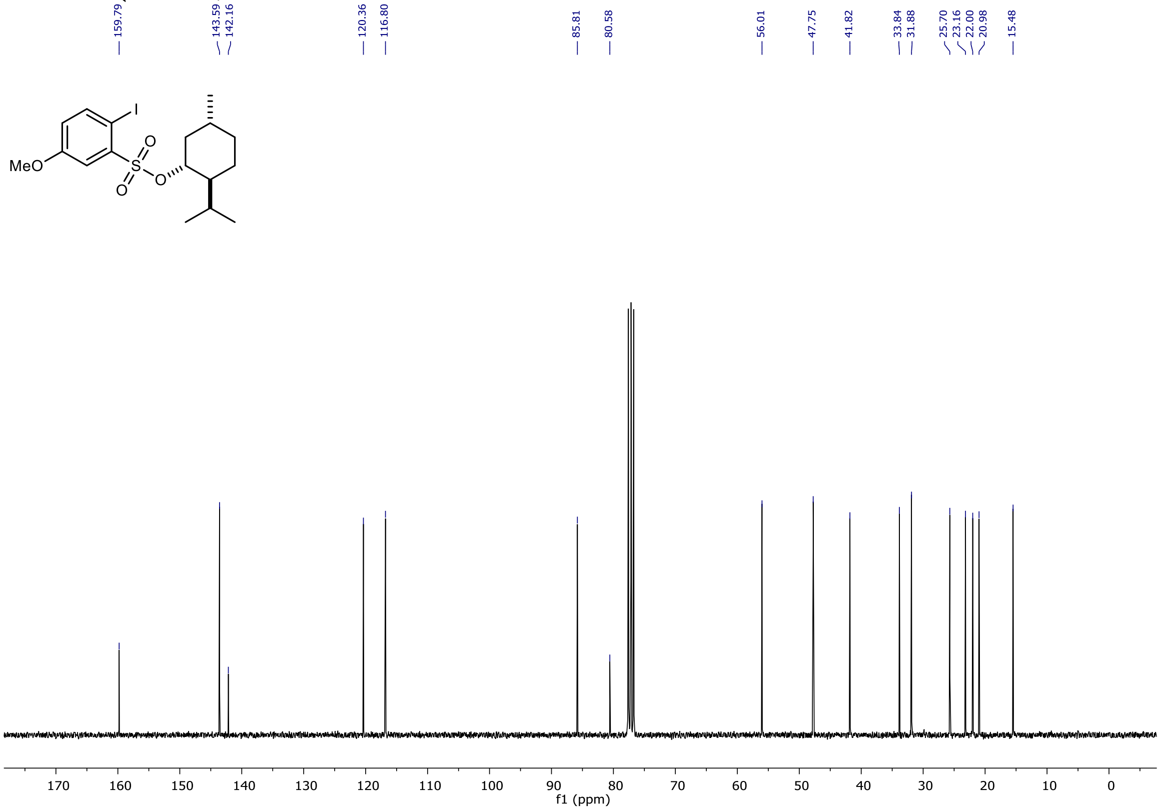

Supplementary Figure 69: NMR Spectra of (1*R*,2*S*,5*R*)-2-Isopropyl-5-methylcyclohexyl 2-iodo-5-methoxybenzenesulfonate (2l-4)

^{1}H NMR (300 MHz, $CDCl_3$)

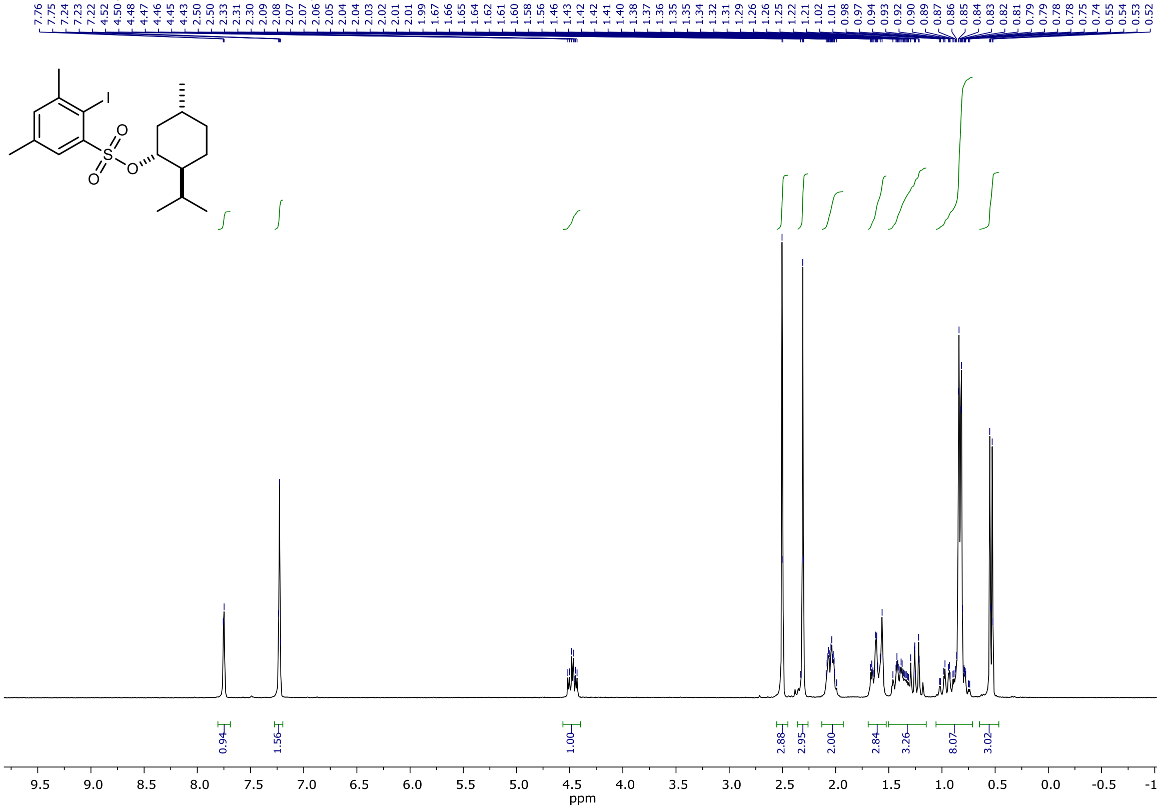

^{13}C NMR (75 MHz, $CDCl_3$)

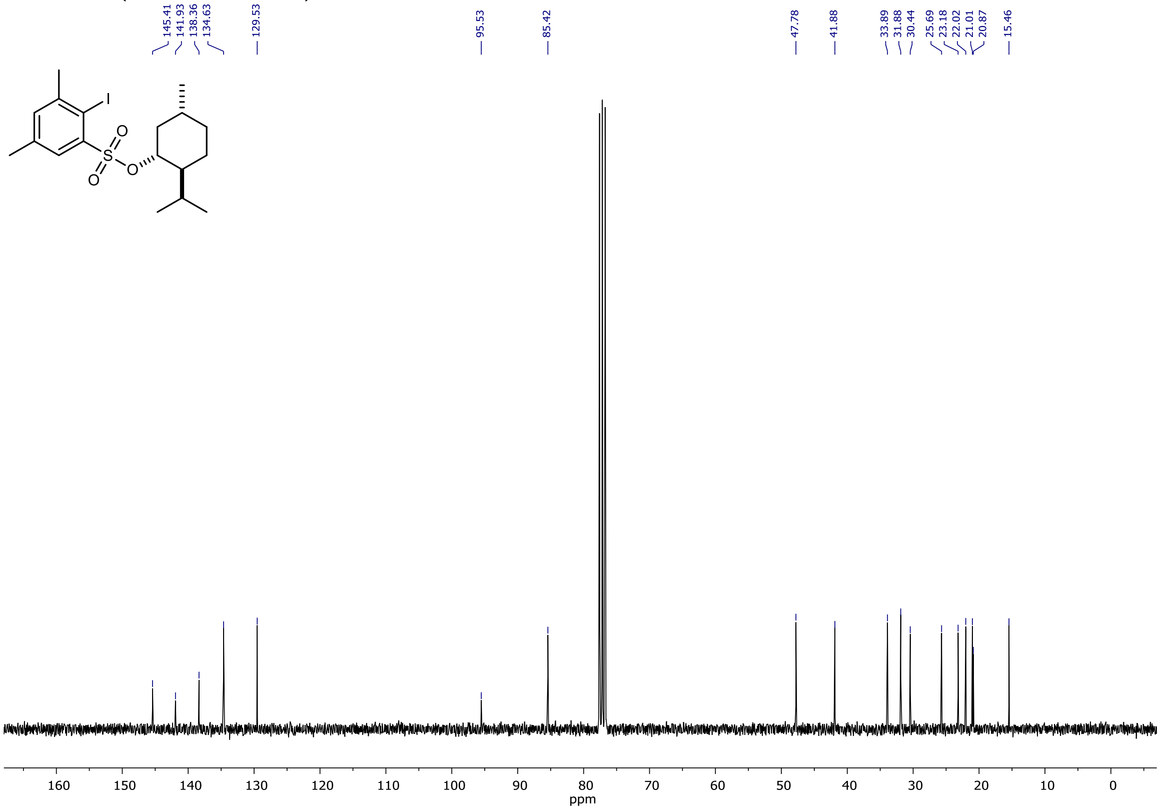

Supplementary Figure 70: NMR Spectra of (1*R*,2*S*,5*R*)-2-Isopropyl-5-methylcyclohexyl 2-iodo-3,5-dimethylbenzenesulfonate (2l-5)

^{1}H NMR (300 MHz, $CDCl_3$)

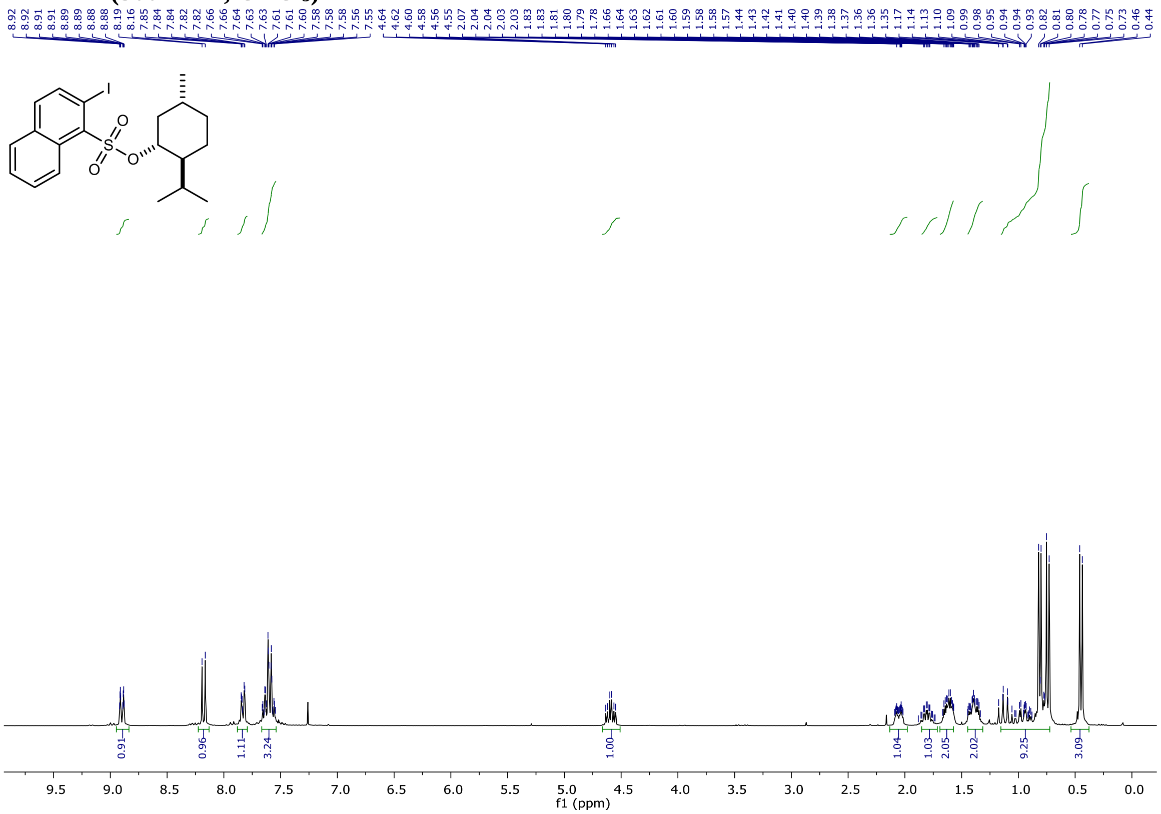

^{13}C NMR (75 MHz, $CDCl_3$)

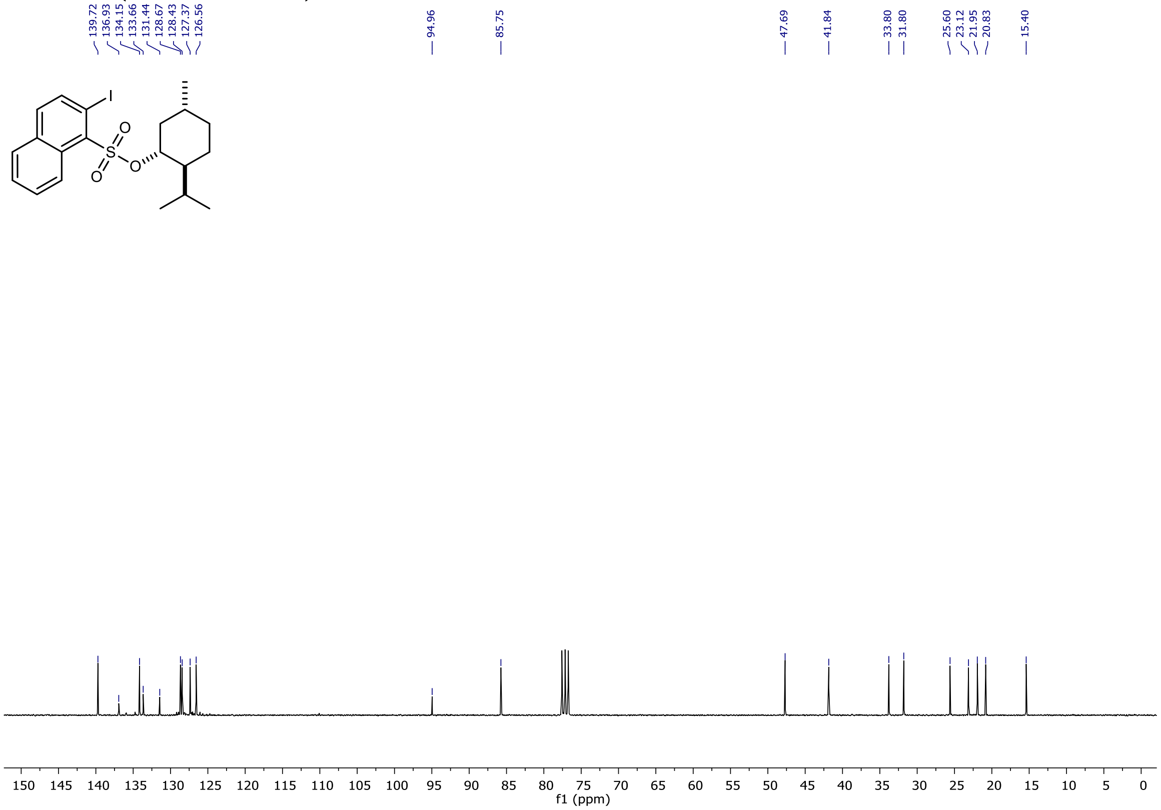

Supplementary Figure 71: NMR Spectra of (1*R*,2*S*,5*R*)-2-Isopropyl-5-methylcyclohexyl 2-iodonaphthalene-1-sulfonate (2l-6)

^{1}H NMR (300 MHz, CDCl$_3$)

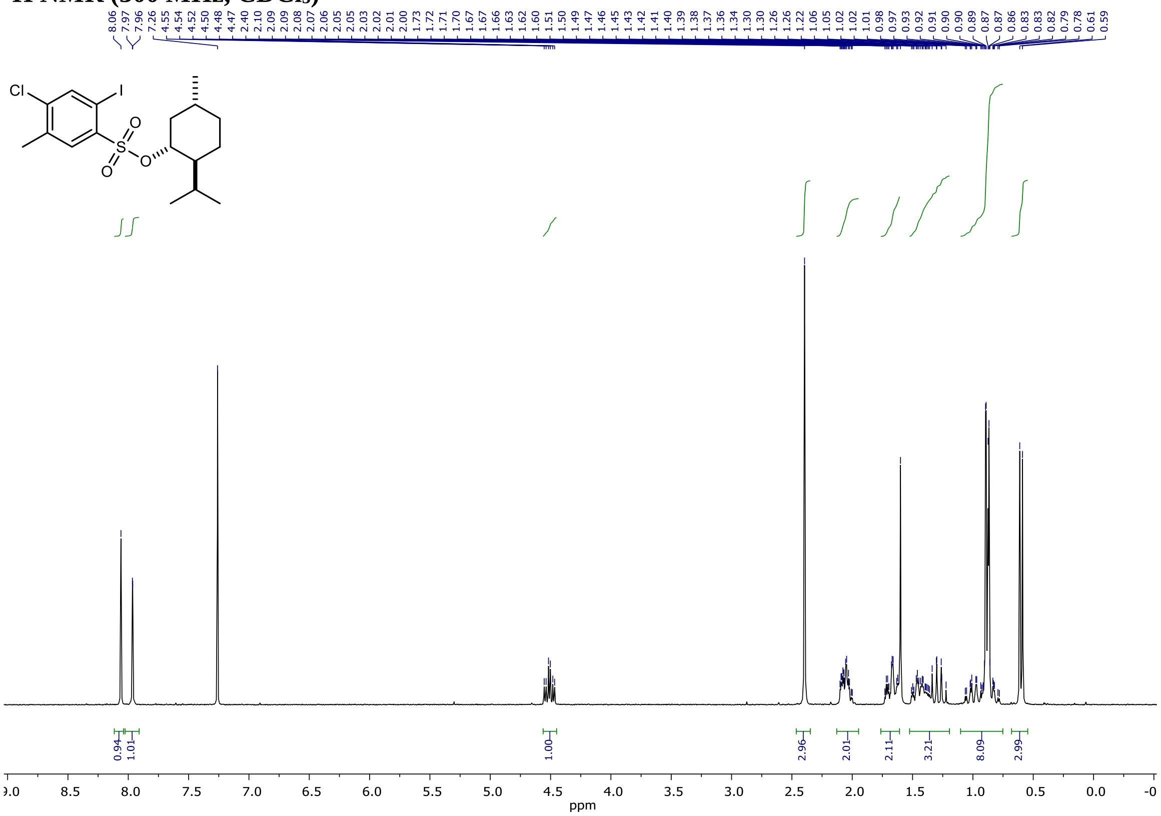

^{13}C NMR (75 MHz, CDCl$_3$)

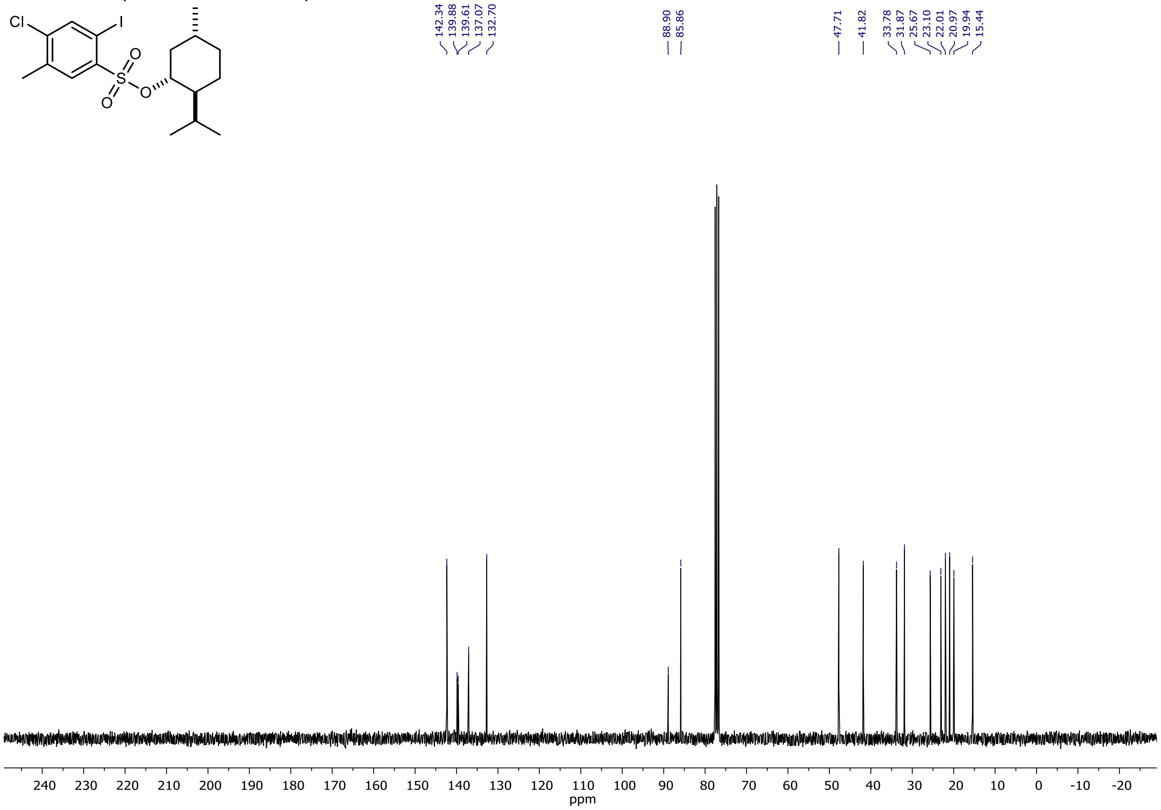

Supplementary Figure 72: NMR Spectra of (1*R*,2*S*,5*R*)-2-Isopropyl-5-methylcyclohexyl 4-chloro-2-iodo-5-methylbenzenesulfonate (2l-7)

^{1}H NMR (300 MHz, CDCl$_3$)

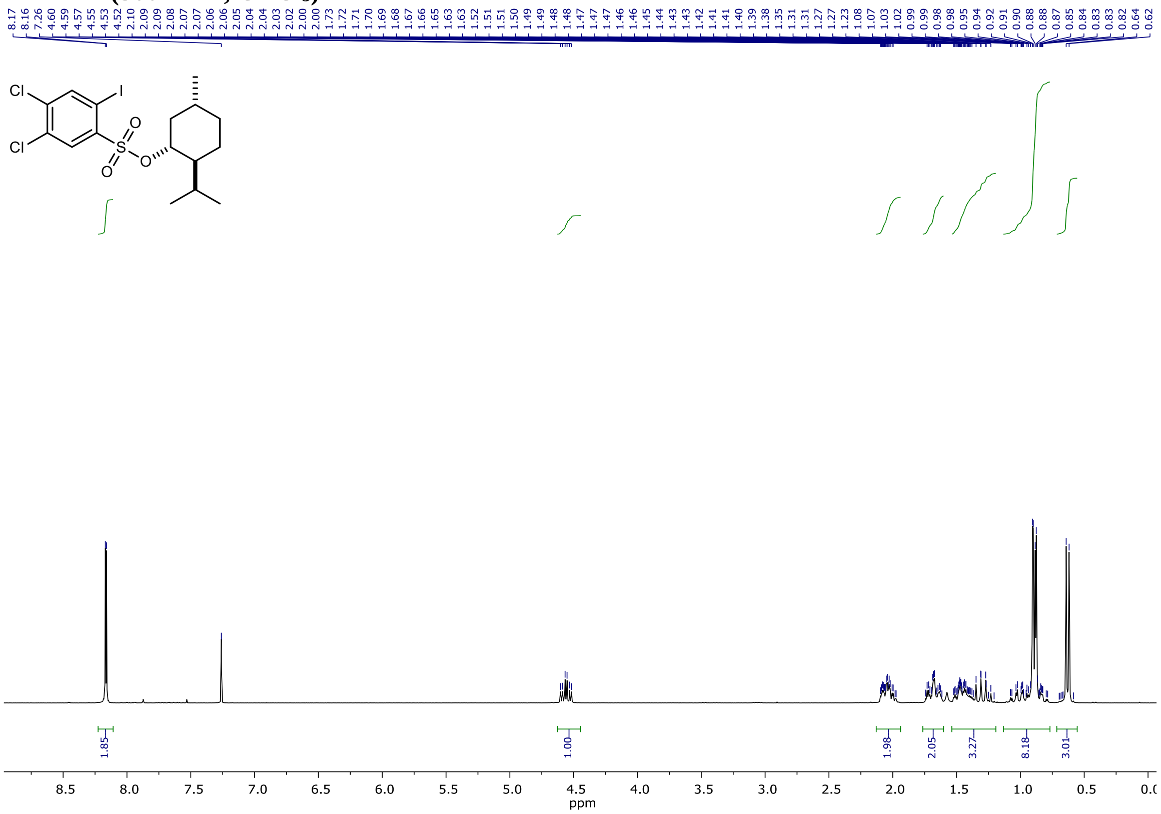

^{13}C NMR (75 MHz, CDCl$_3$)

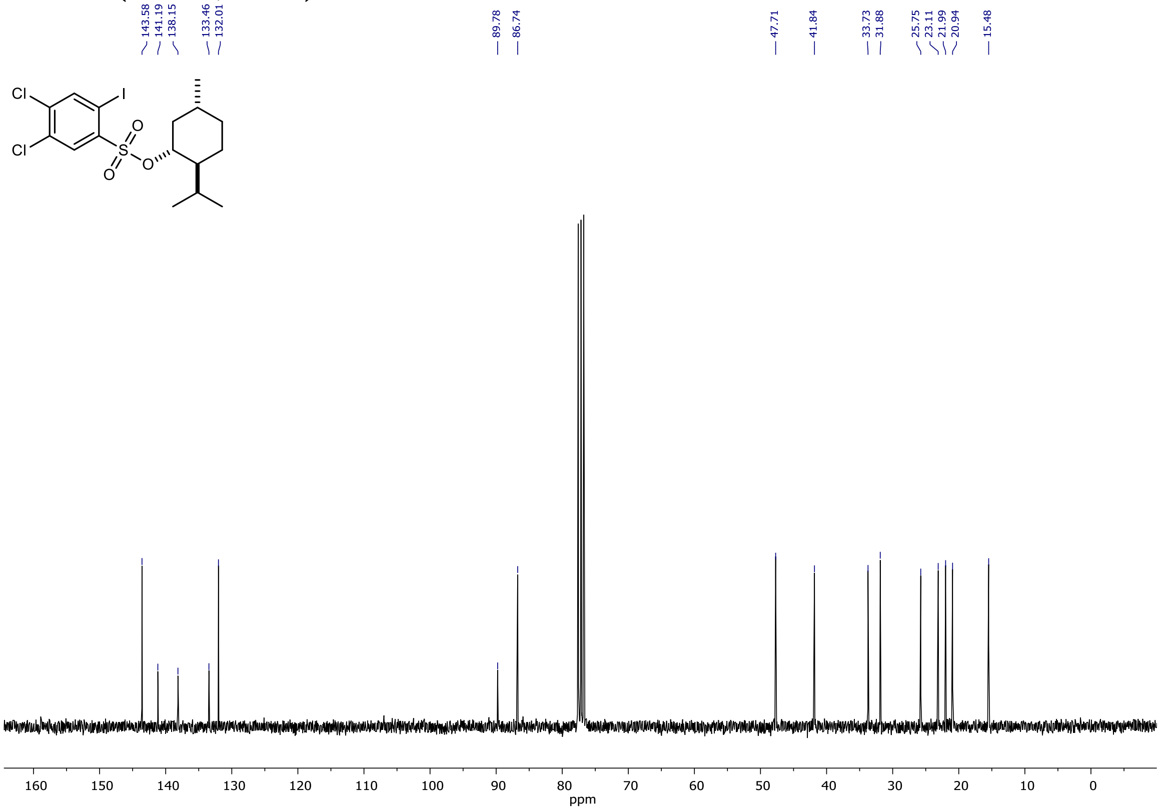

Supplementary Figure 73: NMR Spectra of (1*R*,2*S*,5*R*)-2-Isopropyl-5-methylcyclohexyl 4,5-dichloro-2-iodobenzenesulfonate (2l-8)

^{1}H NMR (300 MHz, $CDCl_3$)

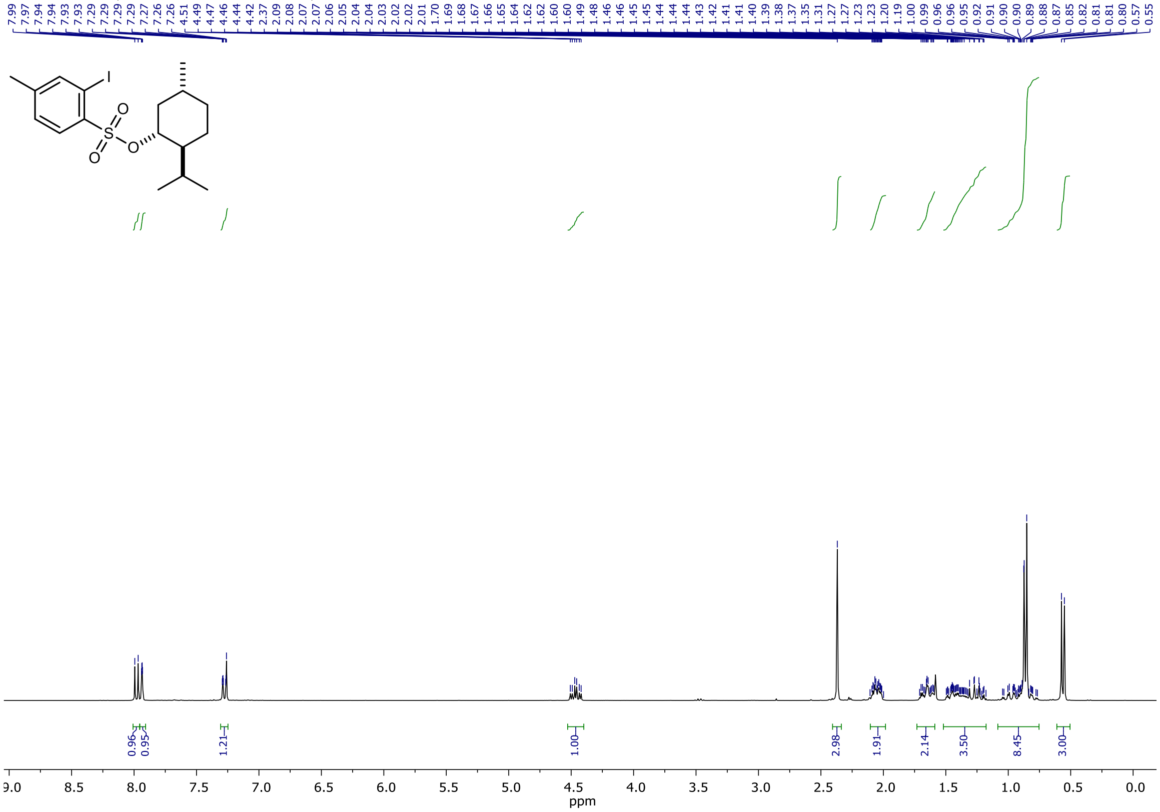

^{13}C NMR (75 MHz, $CDCl_3$)

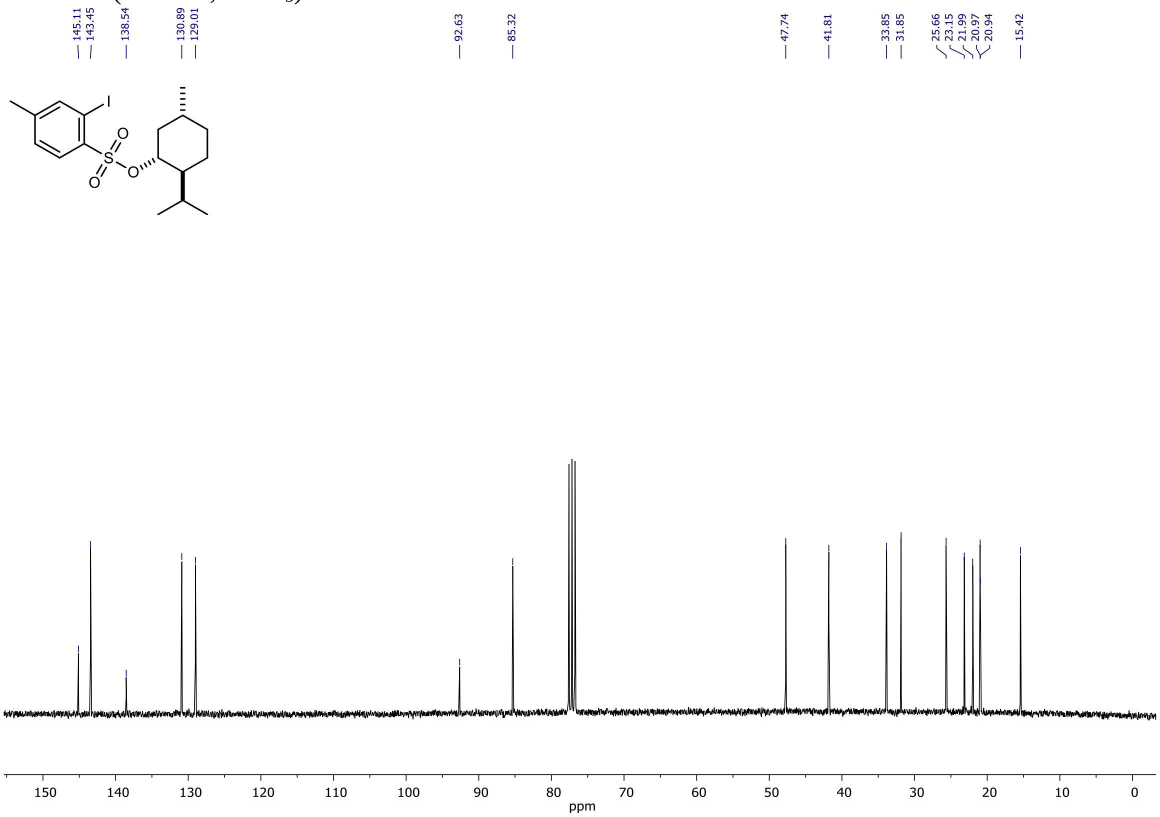

Supplementary Figure 74: NMR Spectra of (1*R*,2*S*,5*R*)-2-Isopropyl-5-methylcyclohexyl 2-iodo-4-methylbenzenesulfonate (2l-9)

^{1}H NMR (300 MHz, $CDCl_3$)

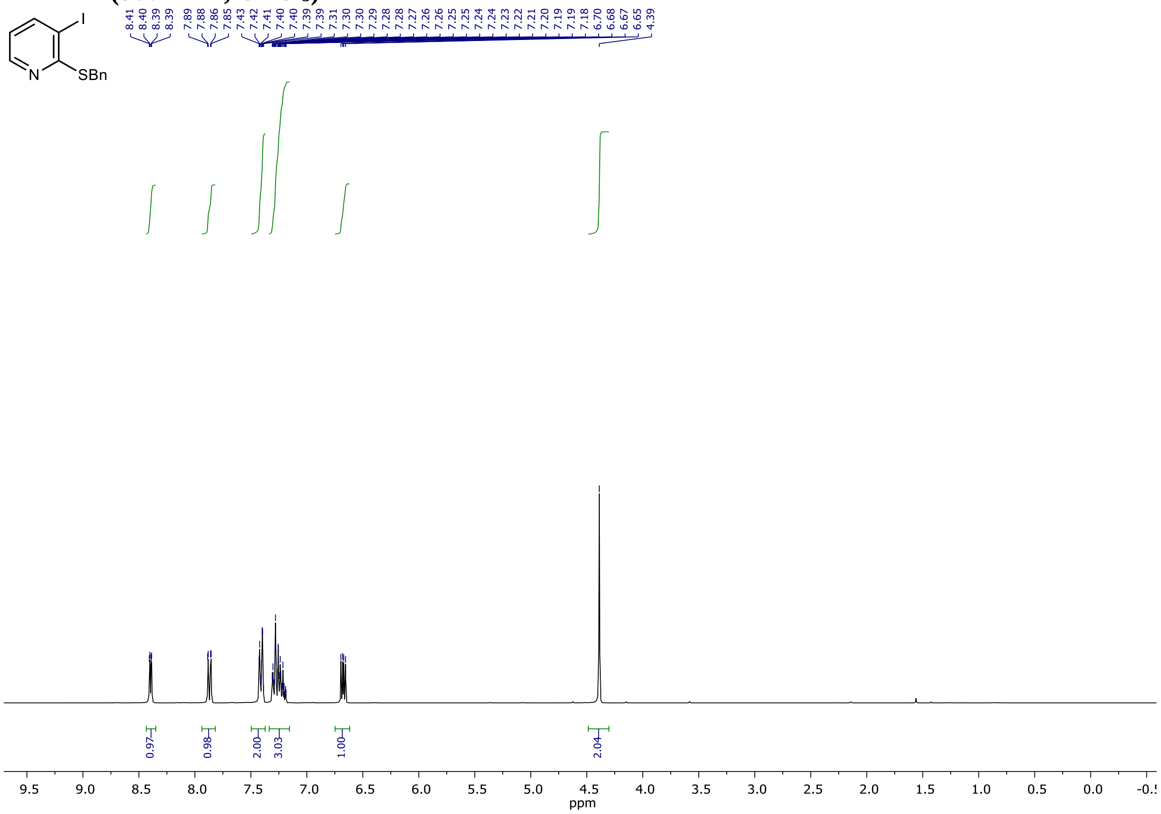

^{13}C NMR (75 MHz, $CDCl_3$)

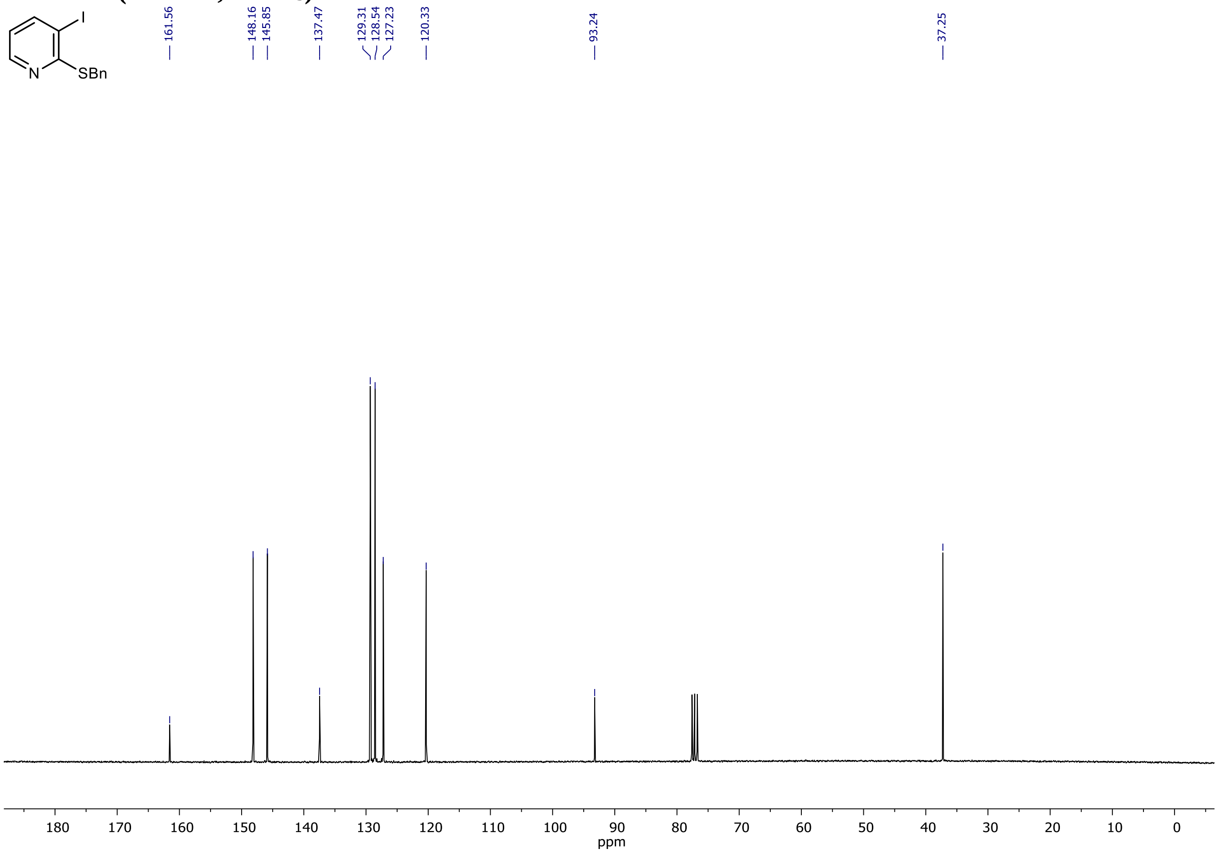

Supplementary Figure 75: NMR Spectra of 2-(Benzylthio)-3-iodopyridine (S-23)

^{1}H NMR (300 MHz, $CDCl_3$)

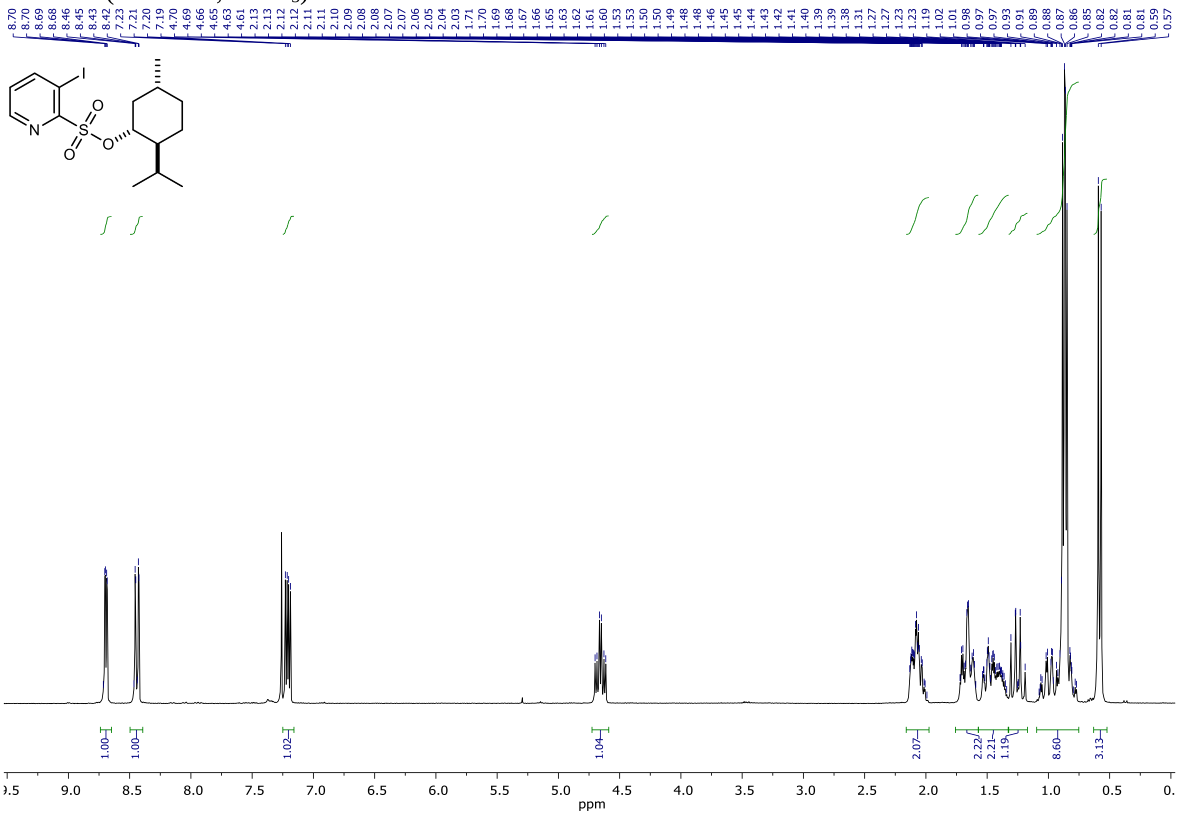

^{13}C NMR (75 MHz, $CDCl_3$)

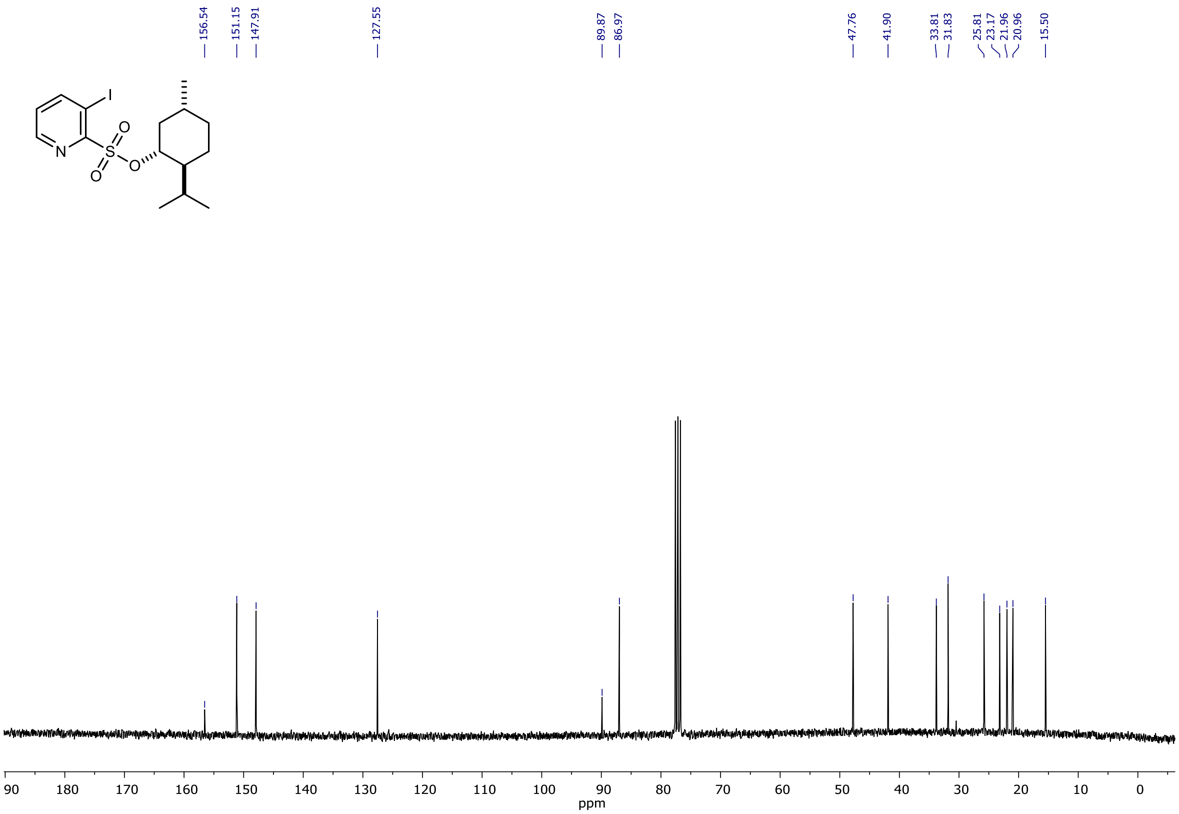

Supplementary Figure 76: NMR Spectra of (1*R*,2*S*,5*R*)-2-Isopropyl-5-methylcyclohexyl 3-iodopyridine-2-sulfonate (2l-10)

^{1}H NMR (300 MHz, $CDCl_3$)

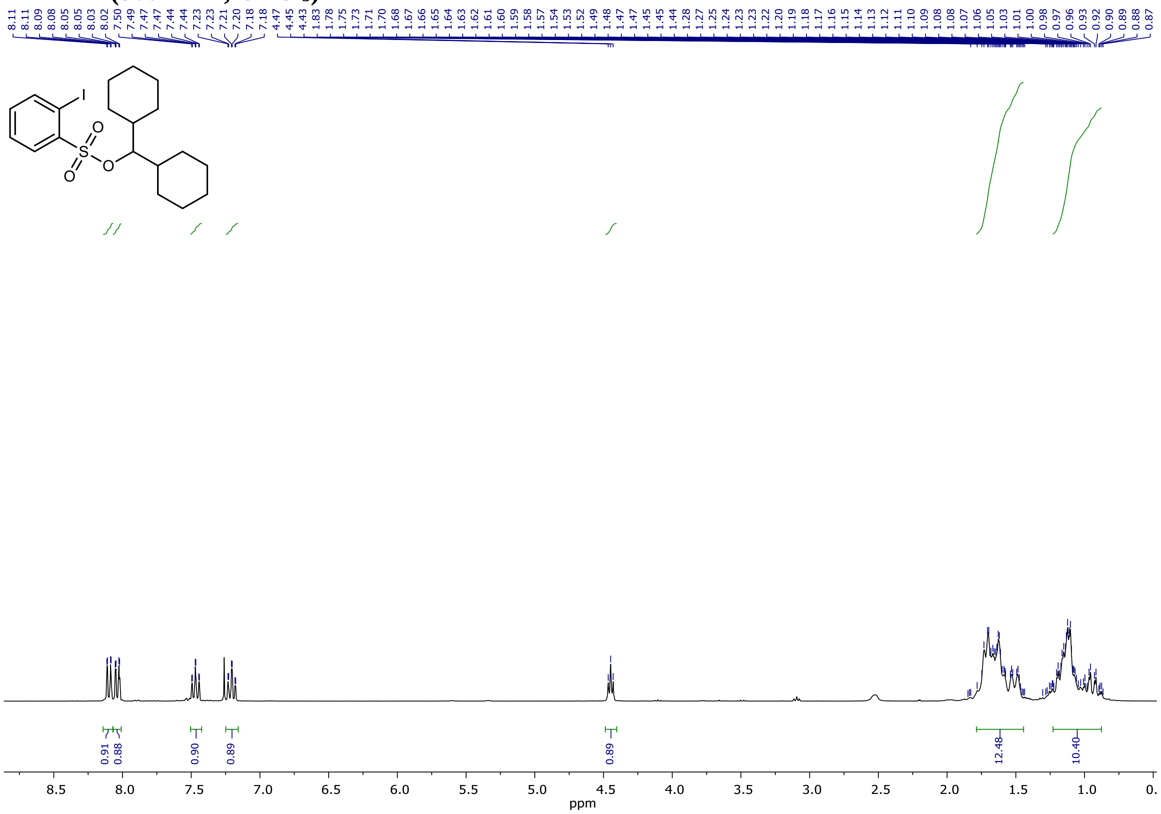

^{13}C NMR (75 MHz, $CDCl_3$)

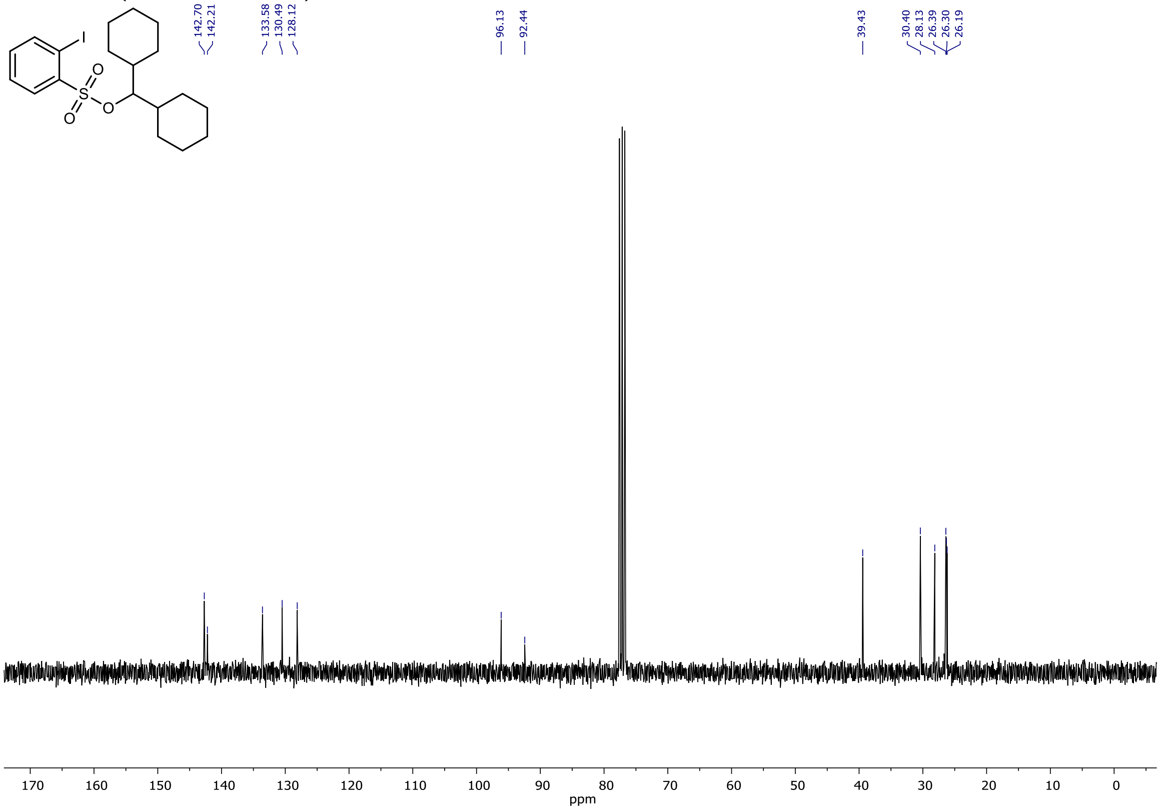

Supplementary Figure 77: NMR Spectra of Dicyclohexylmethyl 2-iodobenzenesulfonate (2m)

^{1}H NMR (400 MHz, CDCl$_3$)

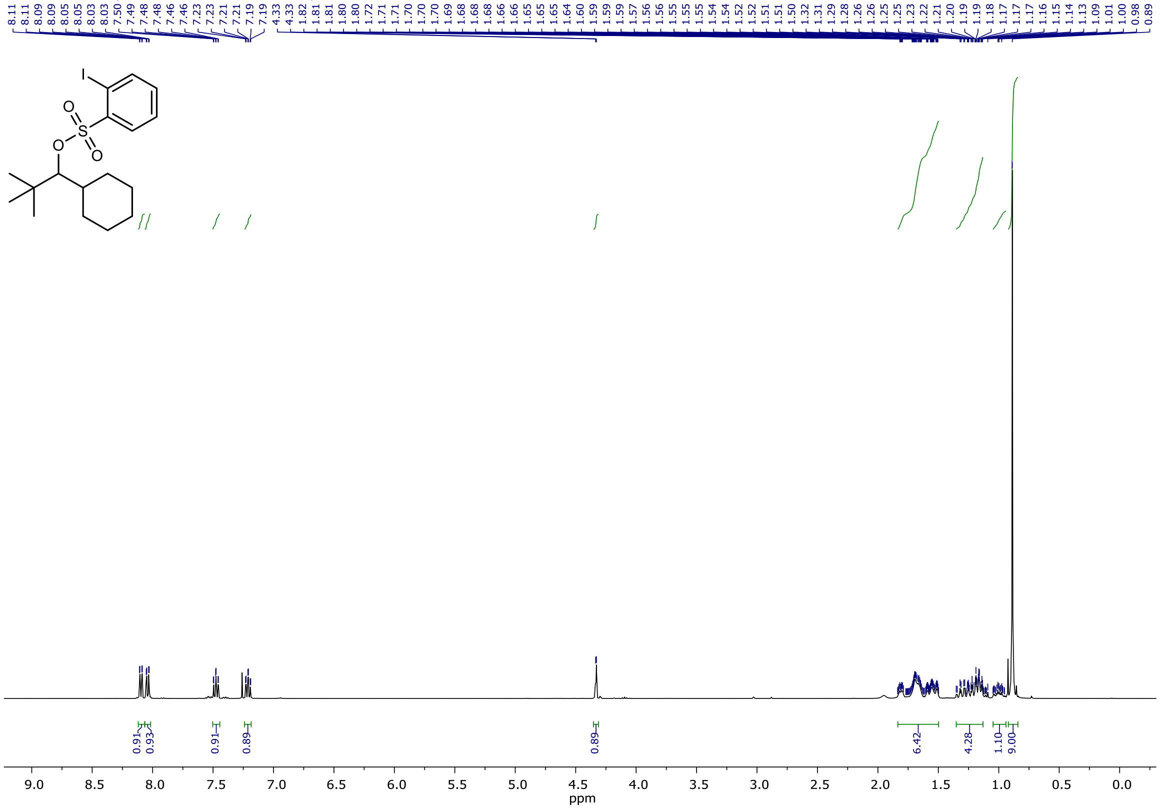

^{13}C NMR (101 MHz, CDCl$_3$)

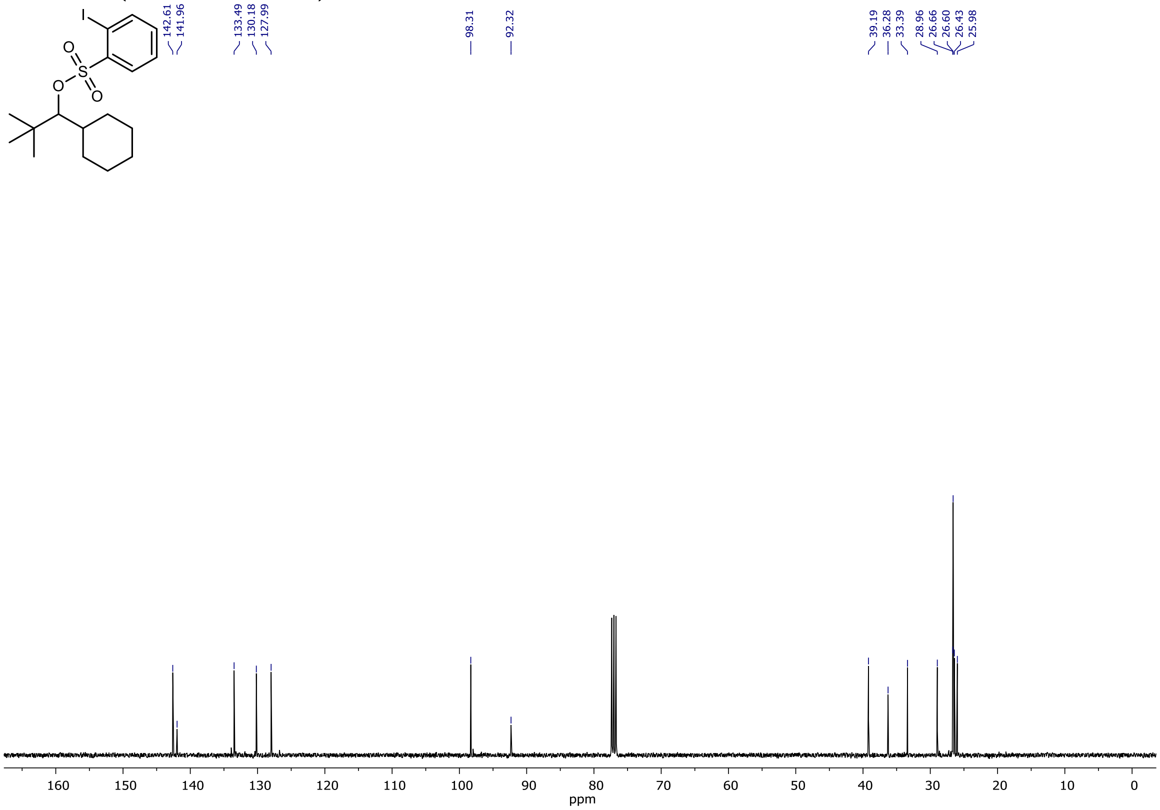

Supplementary Figure 78: NMR Spectra of 1-Cyclohexyl-2,2-dimethylpropyl 2-iodobenzenesulfonate (2n)

^{1}H NMR (300 MHz, $CDCl_3$)

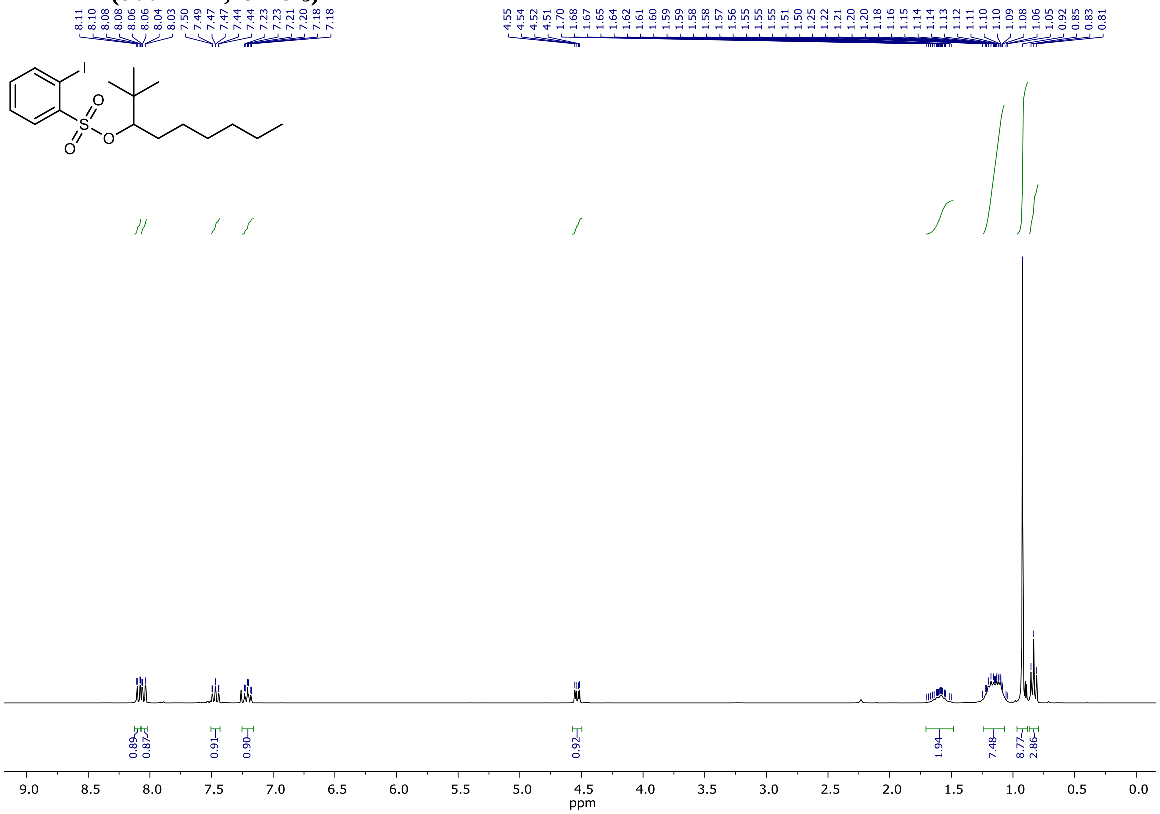

^{13}C NMR (75 MHz, $CDCl_3$)

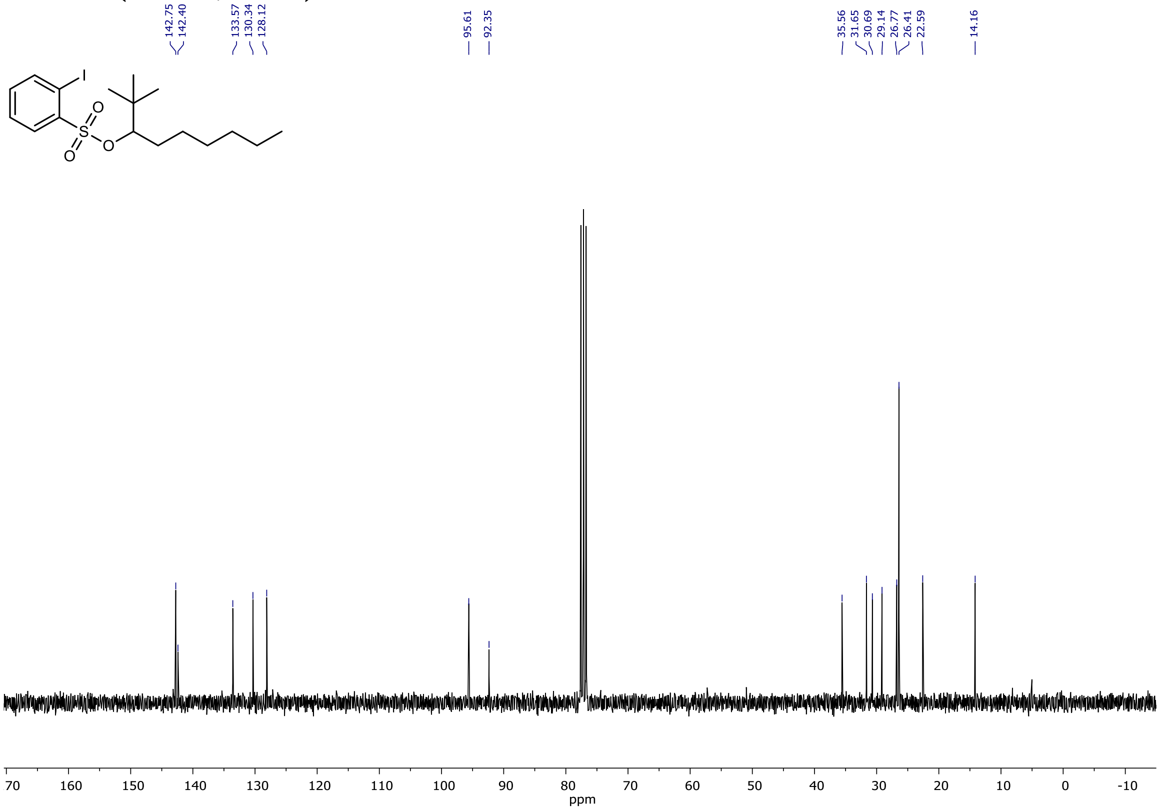

Supplementary Figure 79: NMR Spectra of 2,2-Dimethylnonan-3-yl 2-iodobenzenesulfonate (2o)

^{1}H NMR (400 MHz, $CDCl_3$)

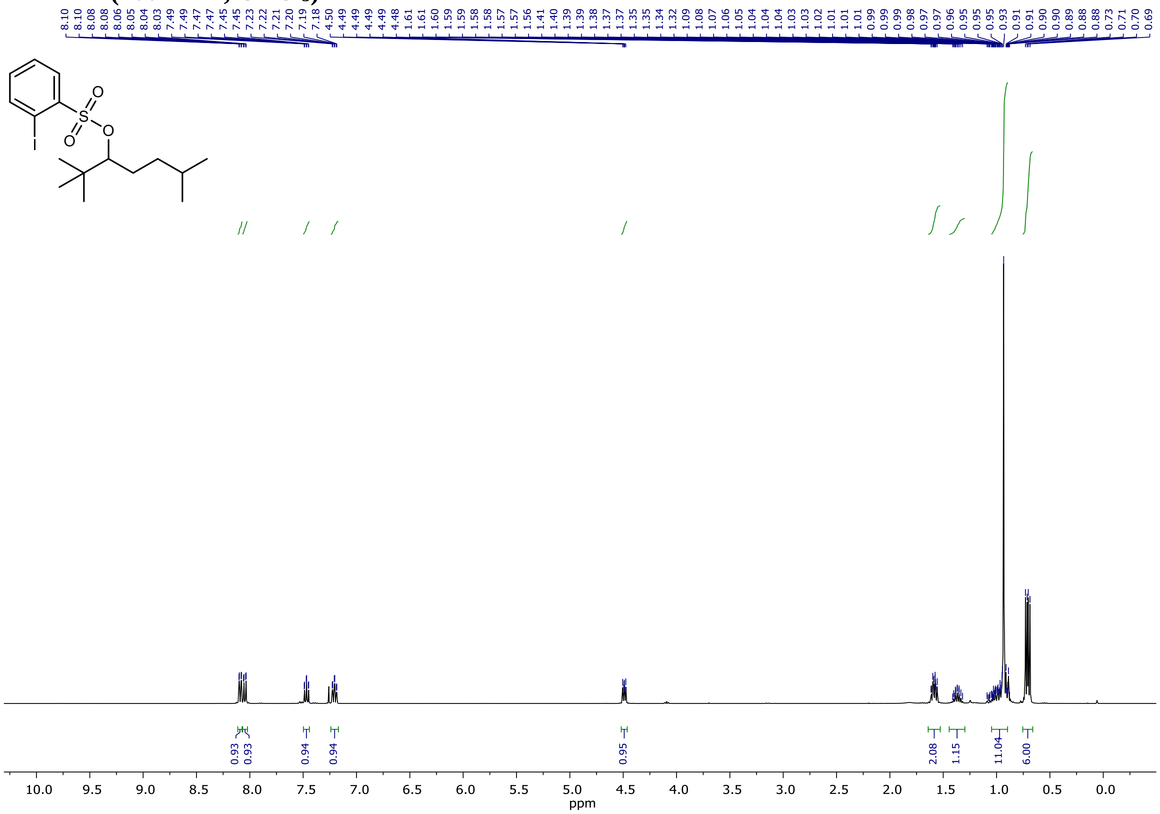

^{13}C NMR (101 MHz, $CDCl_3$)

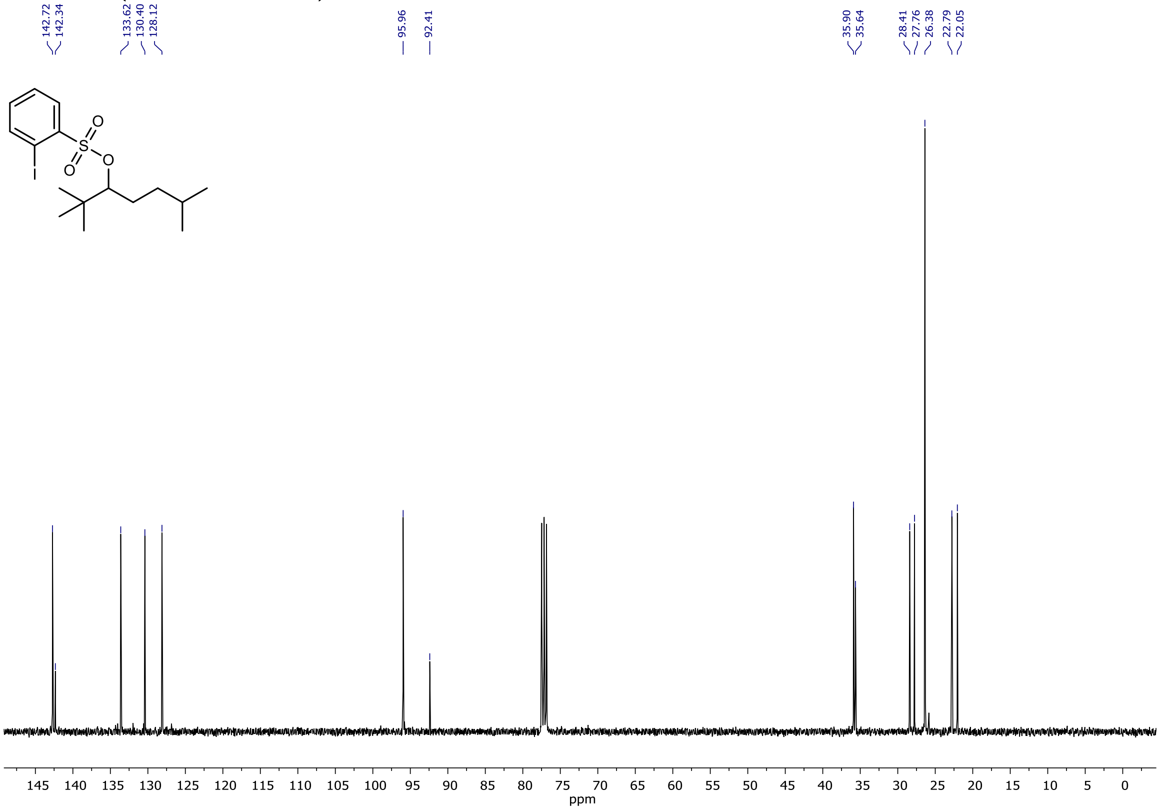

Supplementary Figure 80: NMR Spectra of 2,2,6-Trimethylheptan-3-yl 2-iodobenzenesulfonate (2p)

^{1}H NMR (300 MHz, CDCl$_3$)

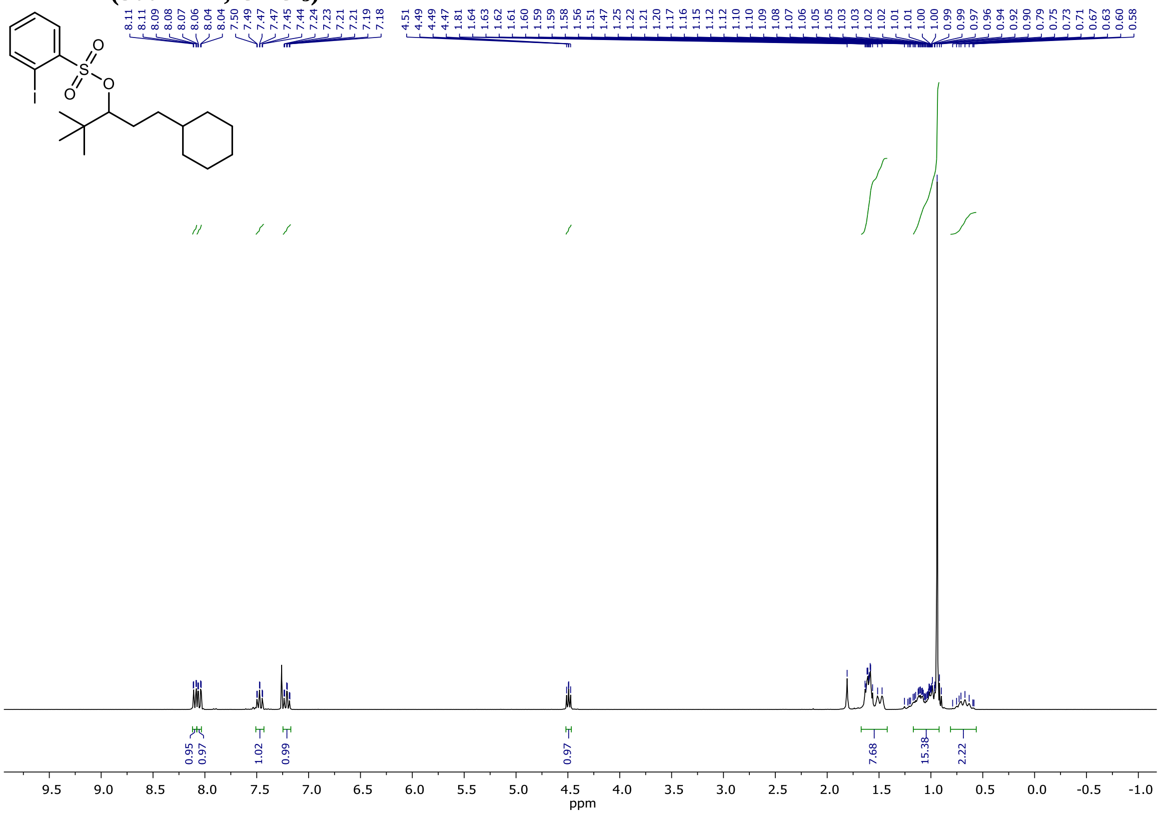

^{13}C NMR (75 MHz, CDCl$_3$)

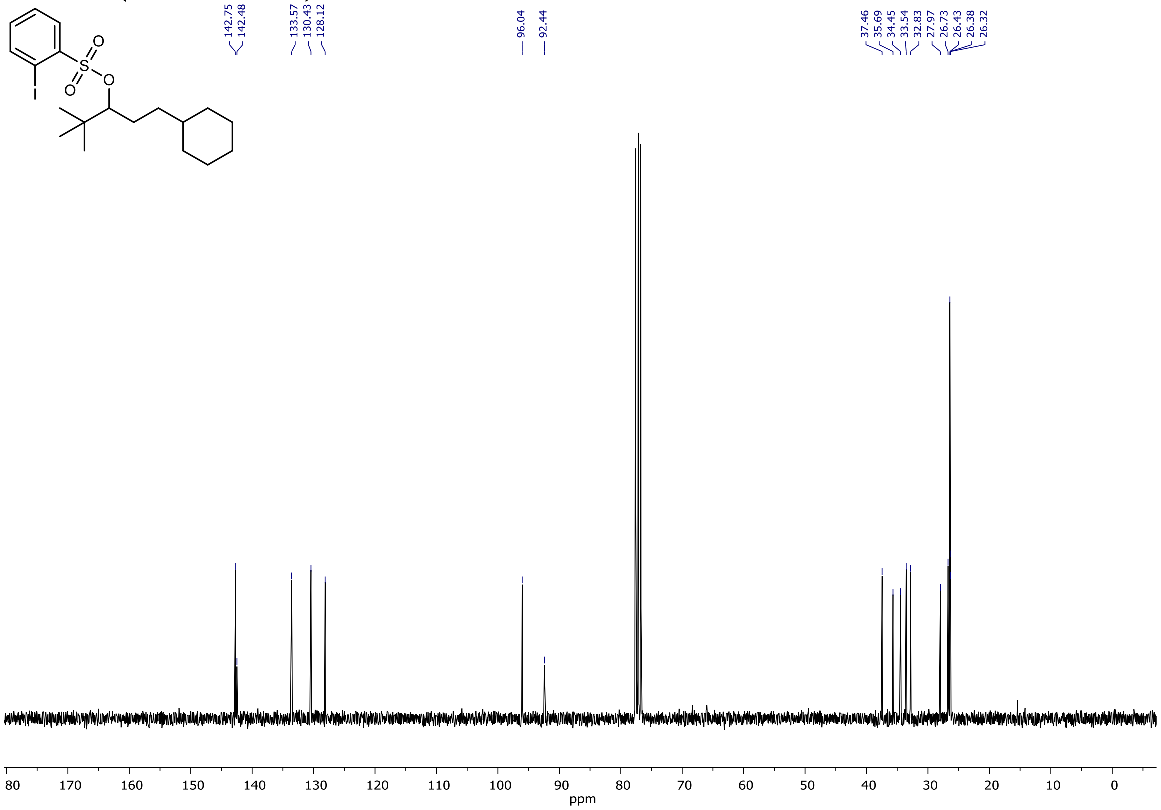

Supplementary Figure 81: NMR Spectra of 1-Cyclohexyl-4,4-dimethylpentan-3-yl 2-iodobenzenesulfonate (2q)

^{1}H NMR (400 MHz, CDCl$_3$)

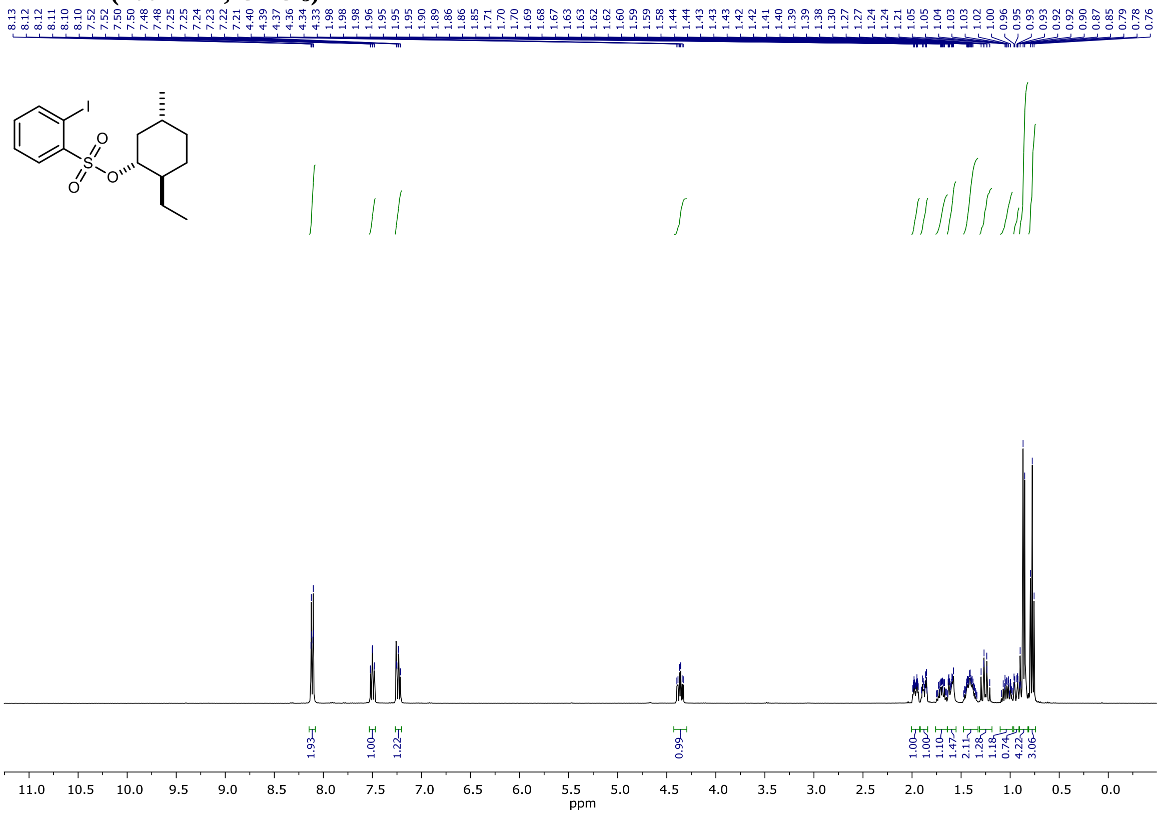

^{13}C NMR (101 MHz, CDCl$_3$)

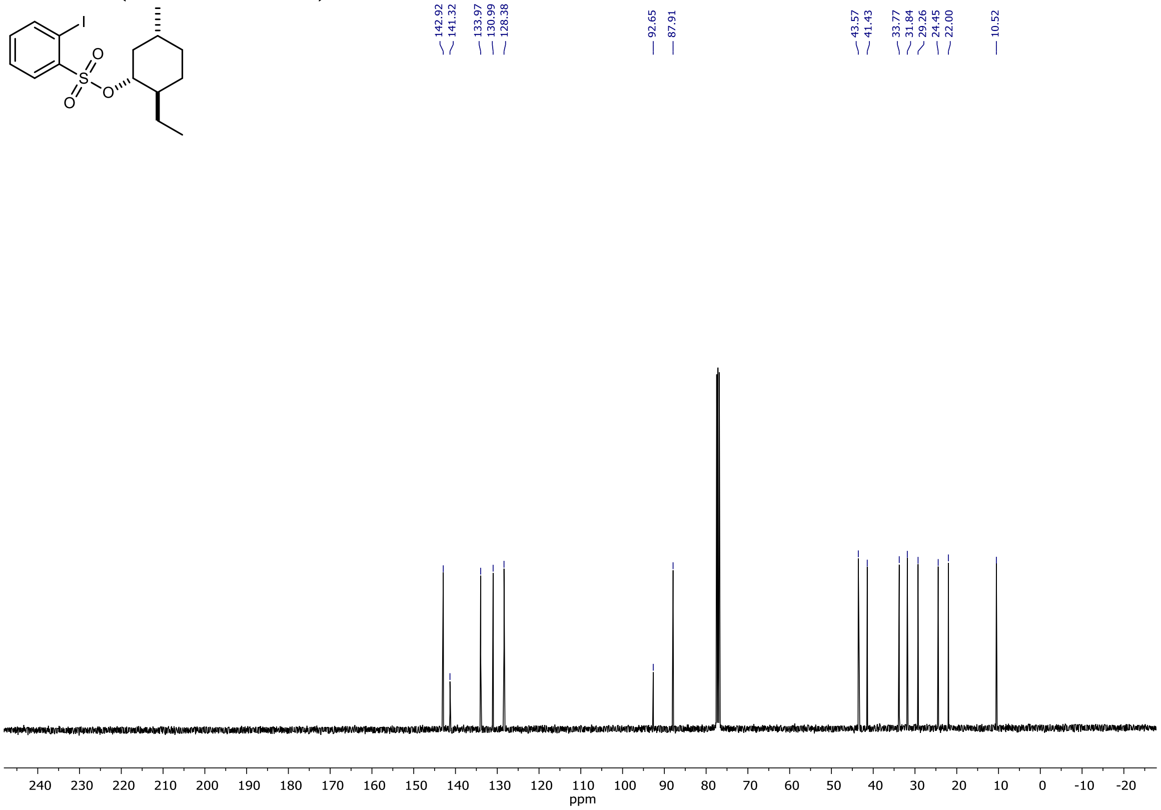

Supplementary Figure 82: NMR Spectra of (1*R*,2*R*,5*R*)-2-Ethyl-5-methylcyclohexyl 2-iodobenzenesulfonate (2r-1)

^{1}H NMR (300 MHz, $CDCl_3$)

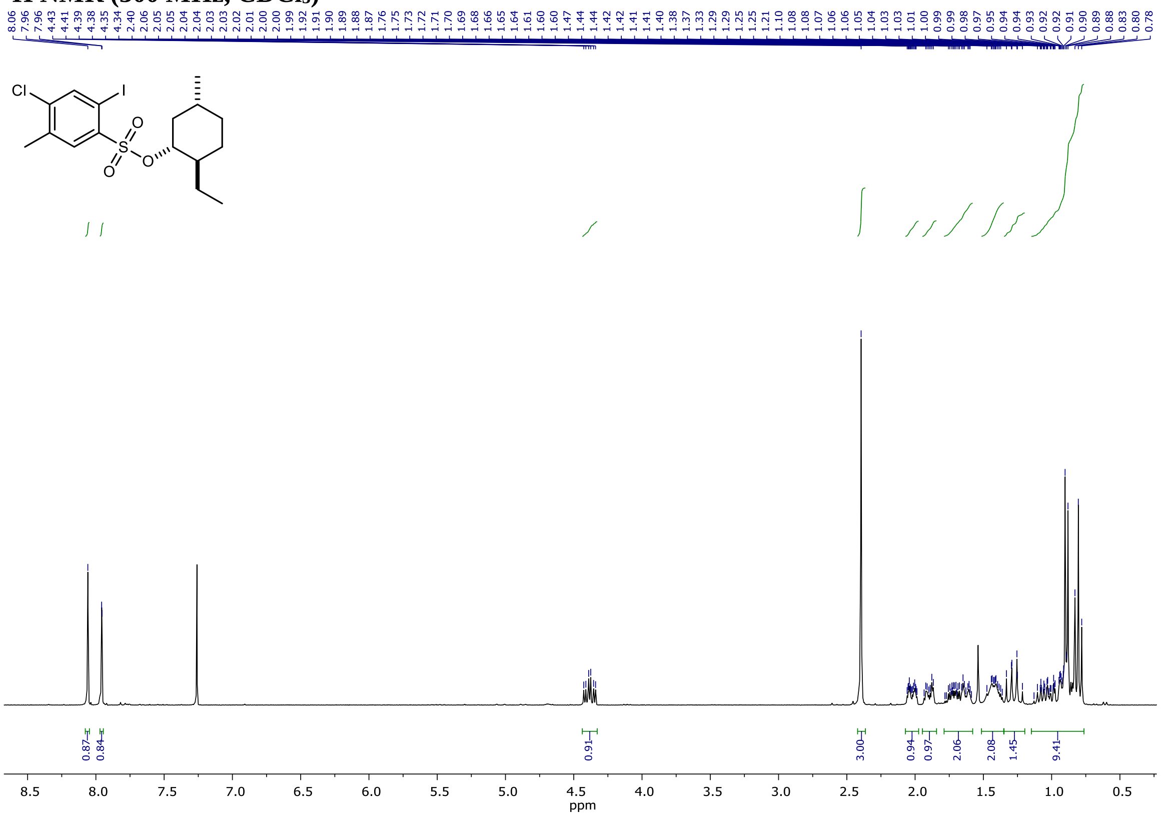

^{13}C NMR (75 MHz, $CDCl_3$)

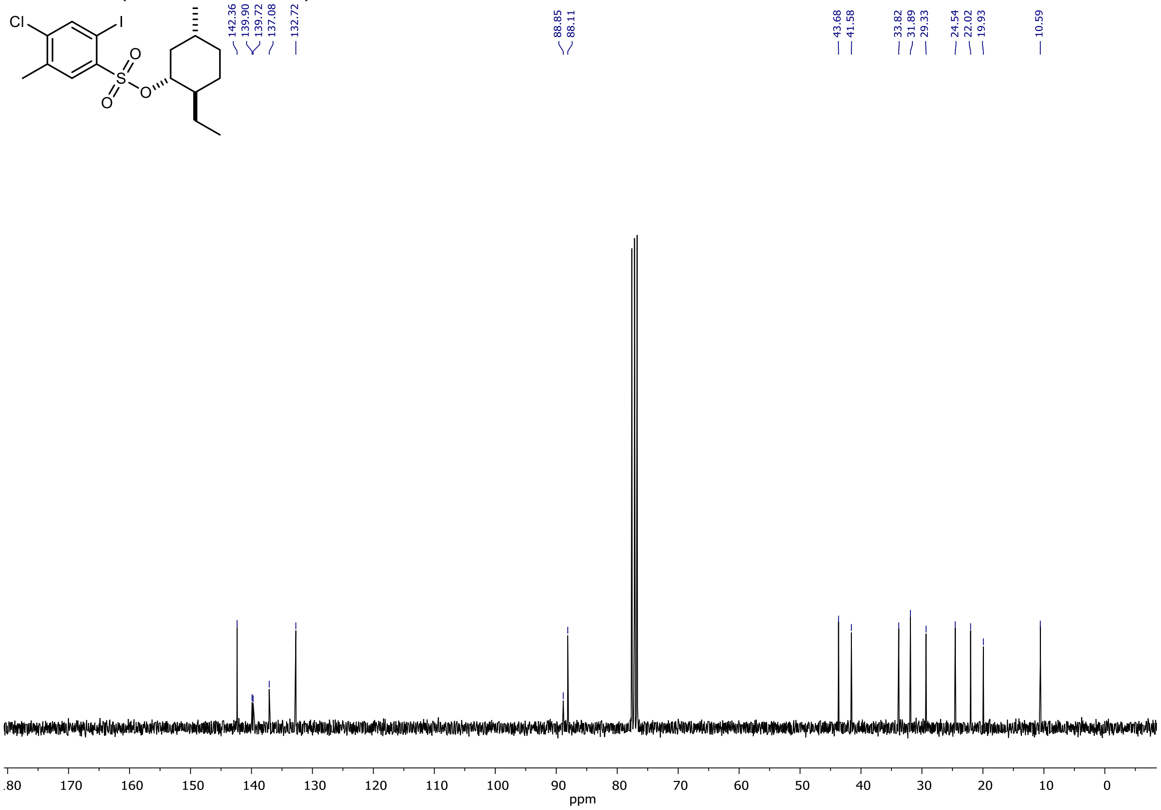

Supplementary Figure 83: NMR Spectra of (1*R*,2*R*,5*R*)-2-Ethyl-5-methylcyclohexyl 4-chloro-2-iodo-5-methylbenzenesulfonate (2r-2)

^{1}H NMR (300 MHz, $CDCl_3$)

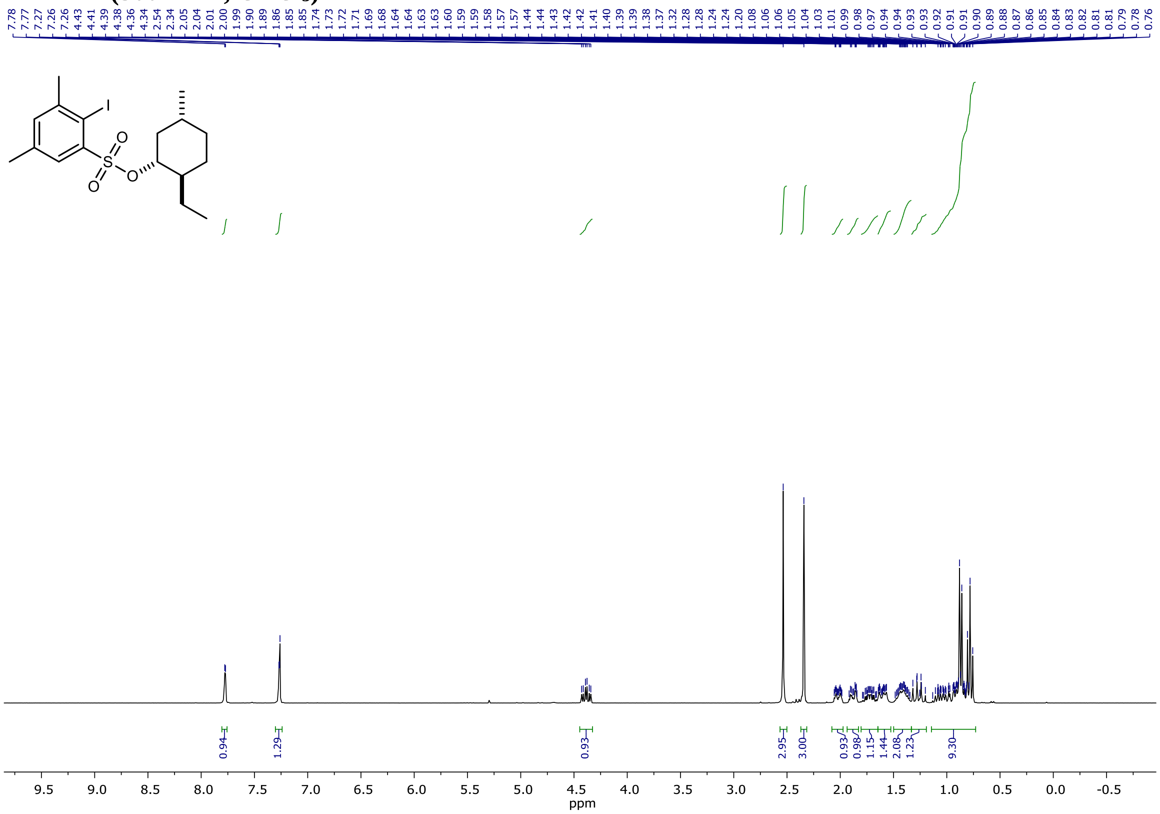

^{13}C NMR (75 MHz, $CDCl_3$)

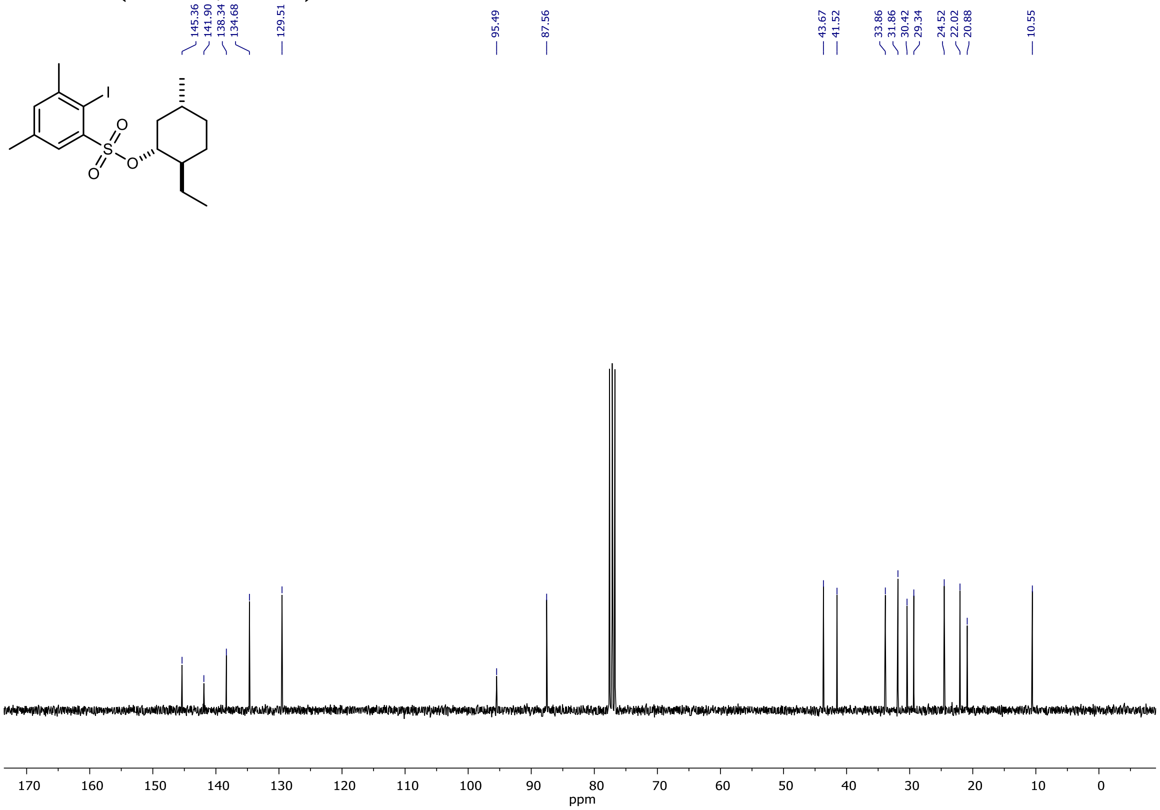

Supplementary Figure 84: NMR Spectra of (1*R*,2*R*,5*R*)-2-Ethyl-5-methylcyclohexyl 2-iodo-3,5-dimethylbenzenesulfonate (2r-3)

^{1}H NMR (300 MHz, $CDCl_3$)

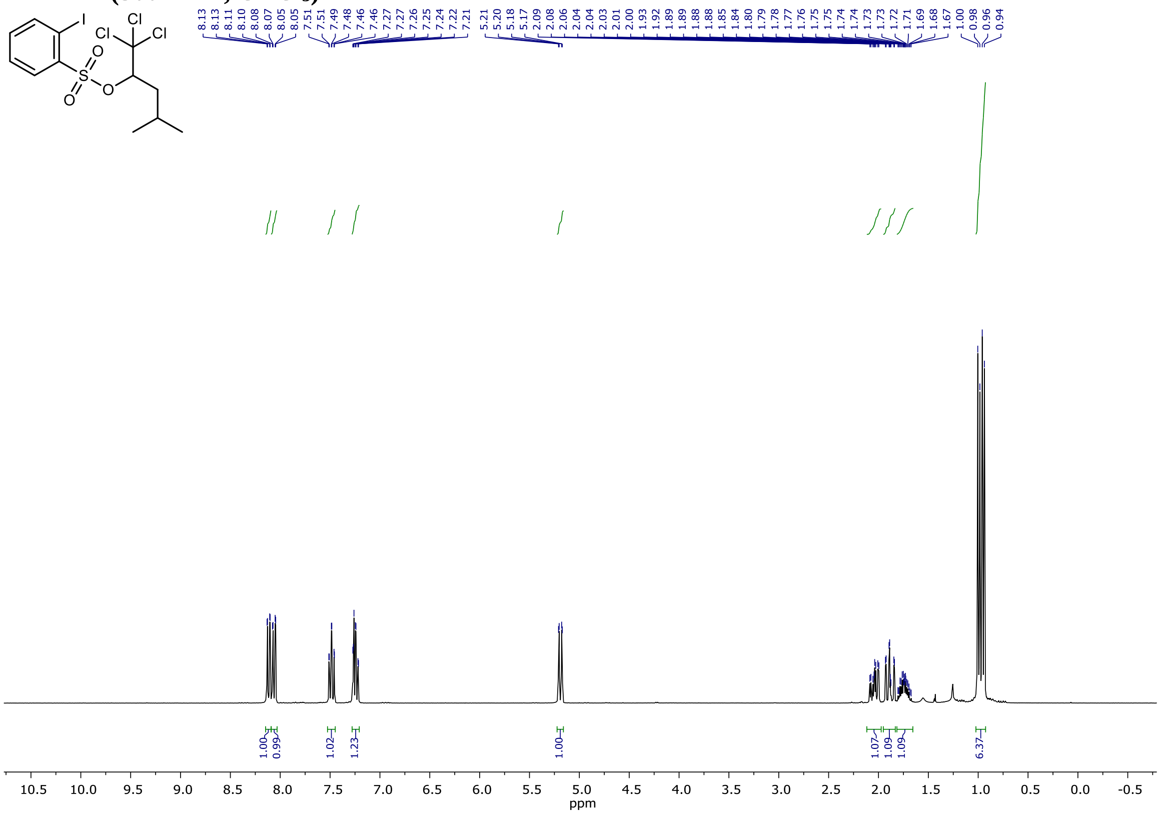

^{13}C NMR (75 MHz, $CDCl_3$)

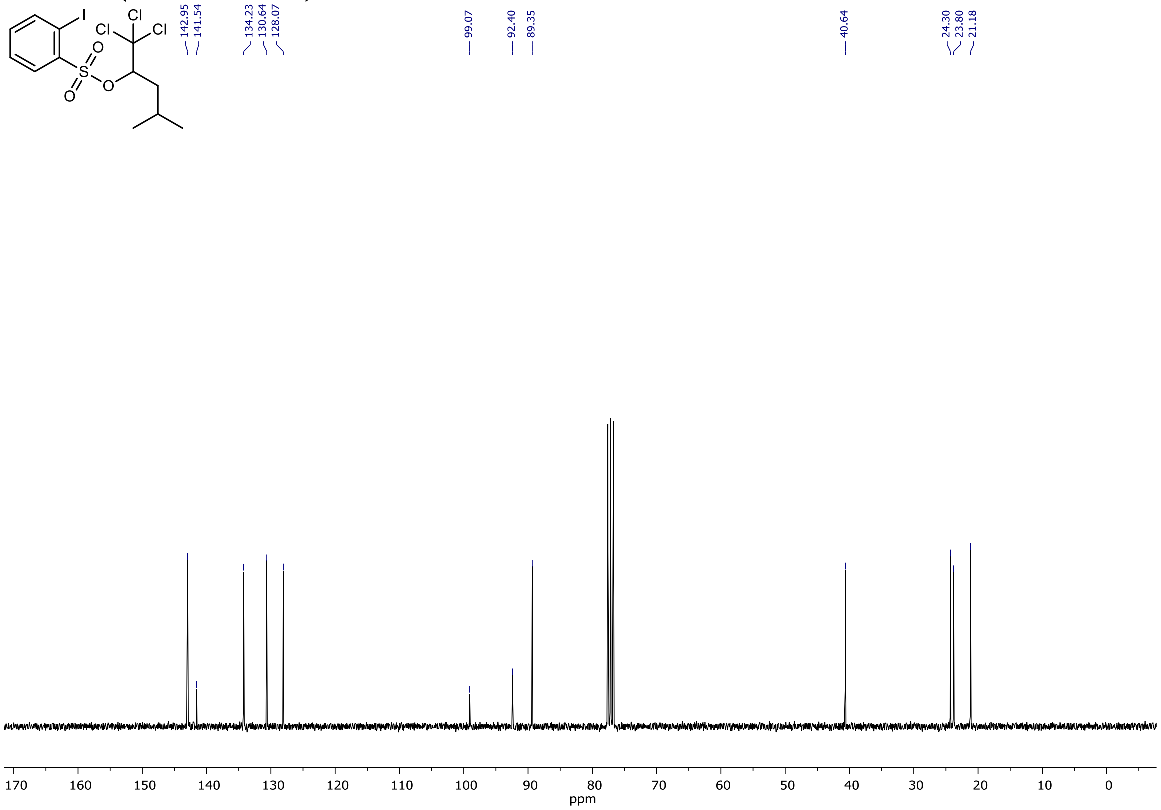

Supplementary Figure 85: NMR Spectra of 1,1,1-Trichloro-4-methylpentan-2-yl 2-iodobenzenesulfonate (2s)

^{1}H NMR (300 MHz, $CDCl_3$)

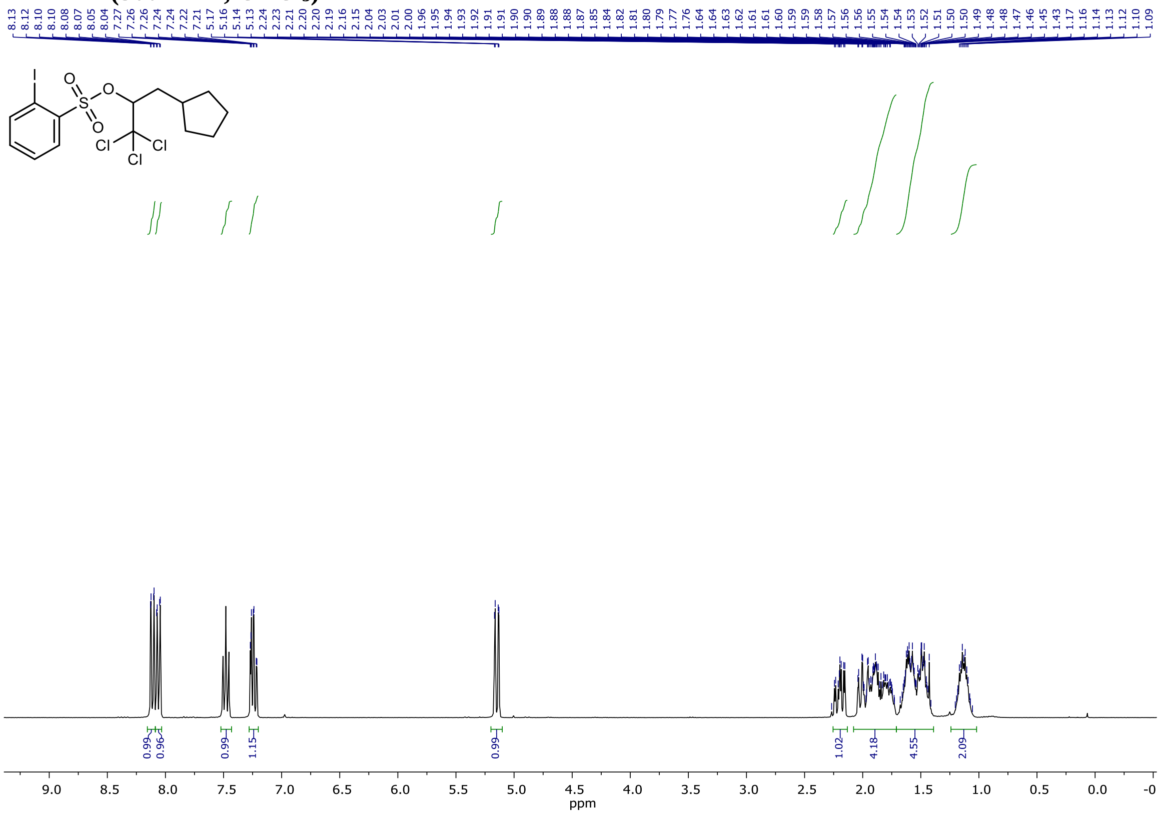

^{13}C NMR (75 MHz, $CDCl_3$)

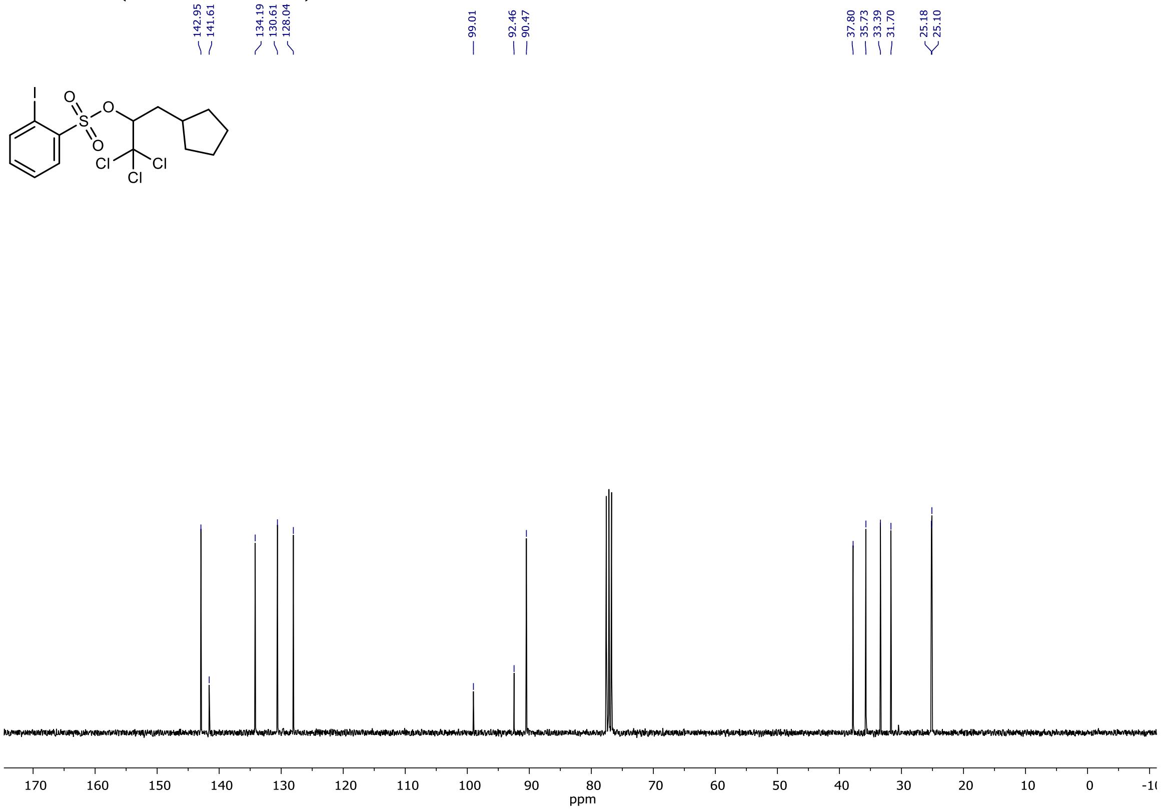

Supplementary Figure 86: NMR Spectra of 1,1,1-Trichloro-3-cyclopentylpropan-2-yl 2-iodobenzenesulfonate (2t)

^{1}H NMR (300 MHz, $CDCl_3$)

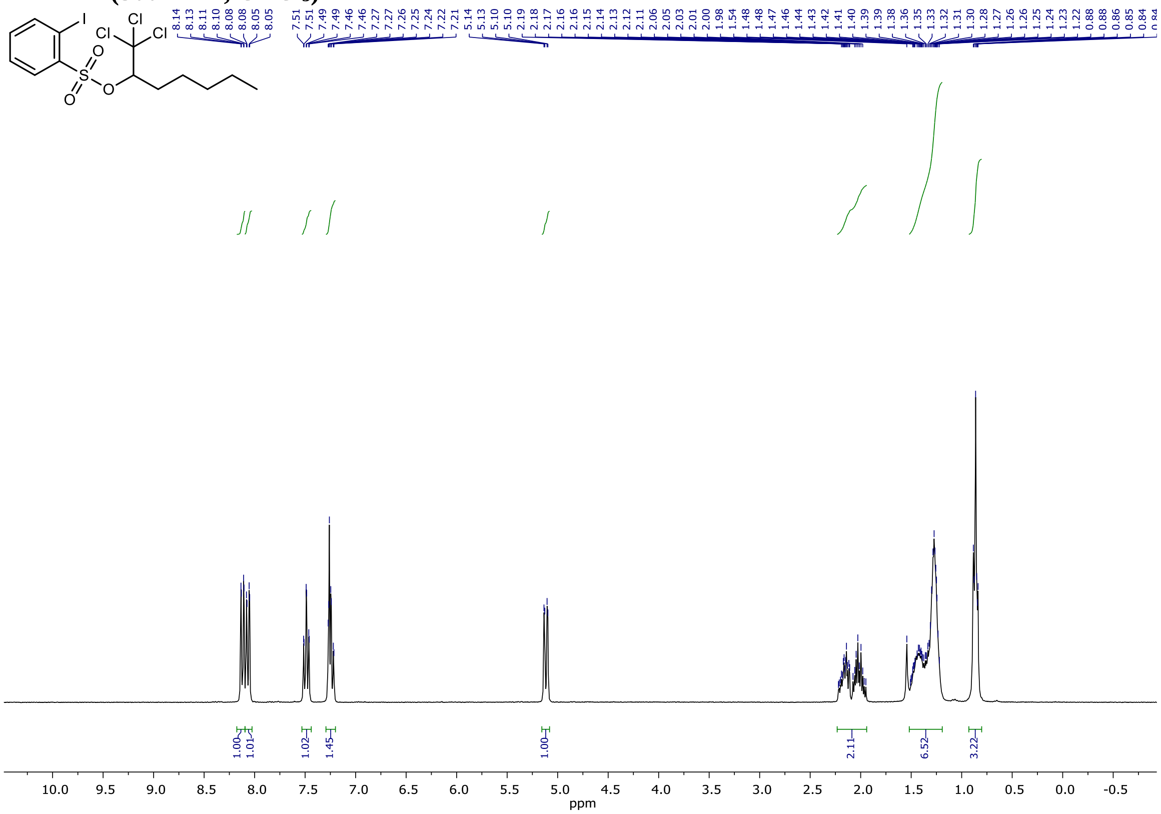

^{13}C NMR (75 MHz, $CDCl_3$)

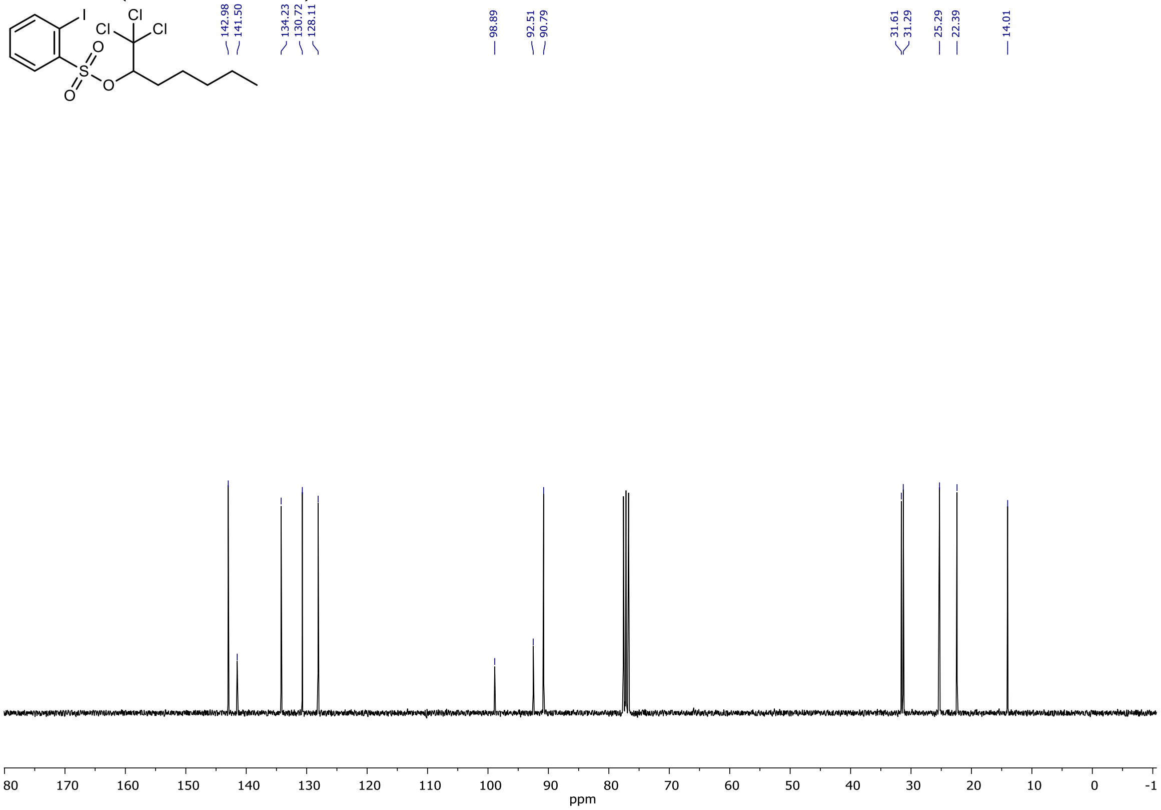

Supplementary Figure 87: NMR Spectra of 1,1,1-Trichloroheptan-2-yl 2-iodobenzenesulfonate (2u)

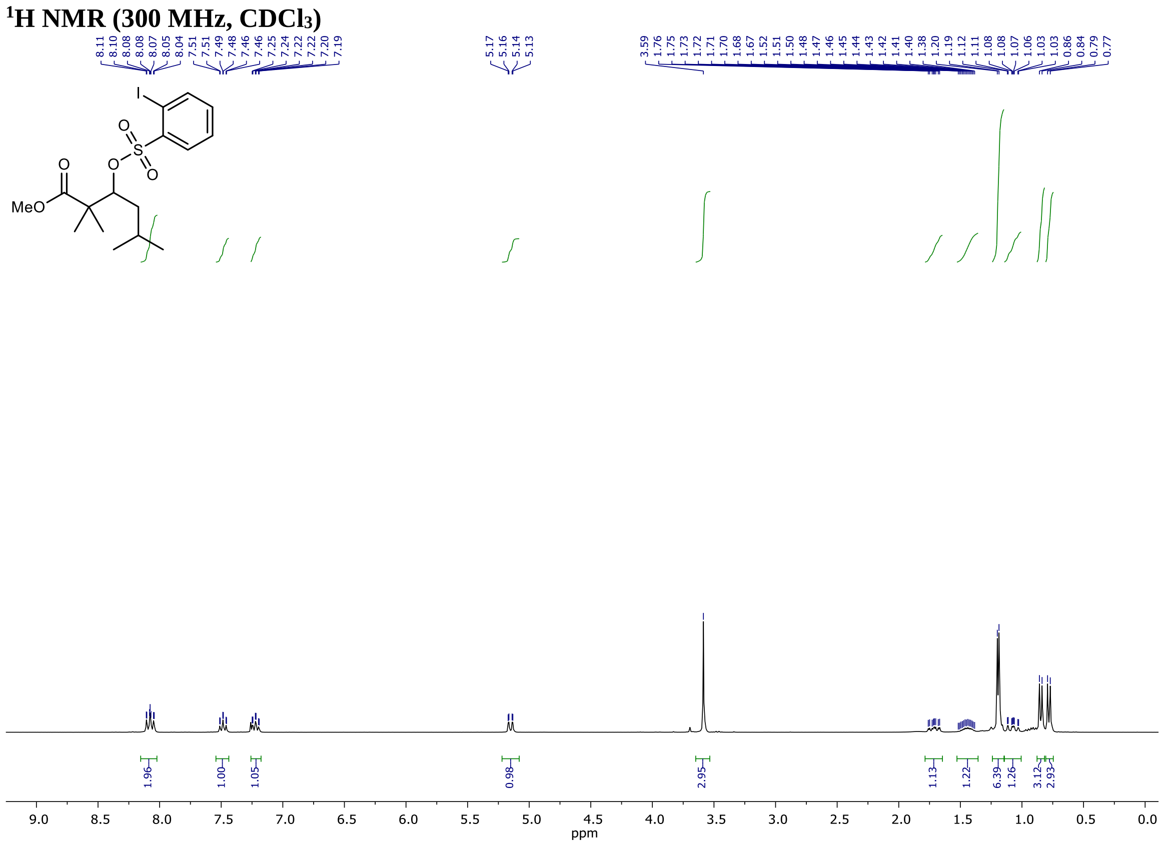

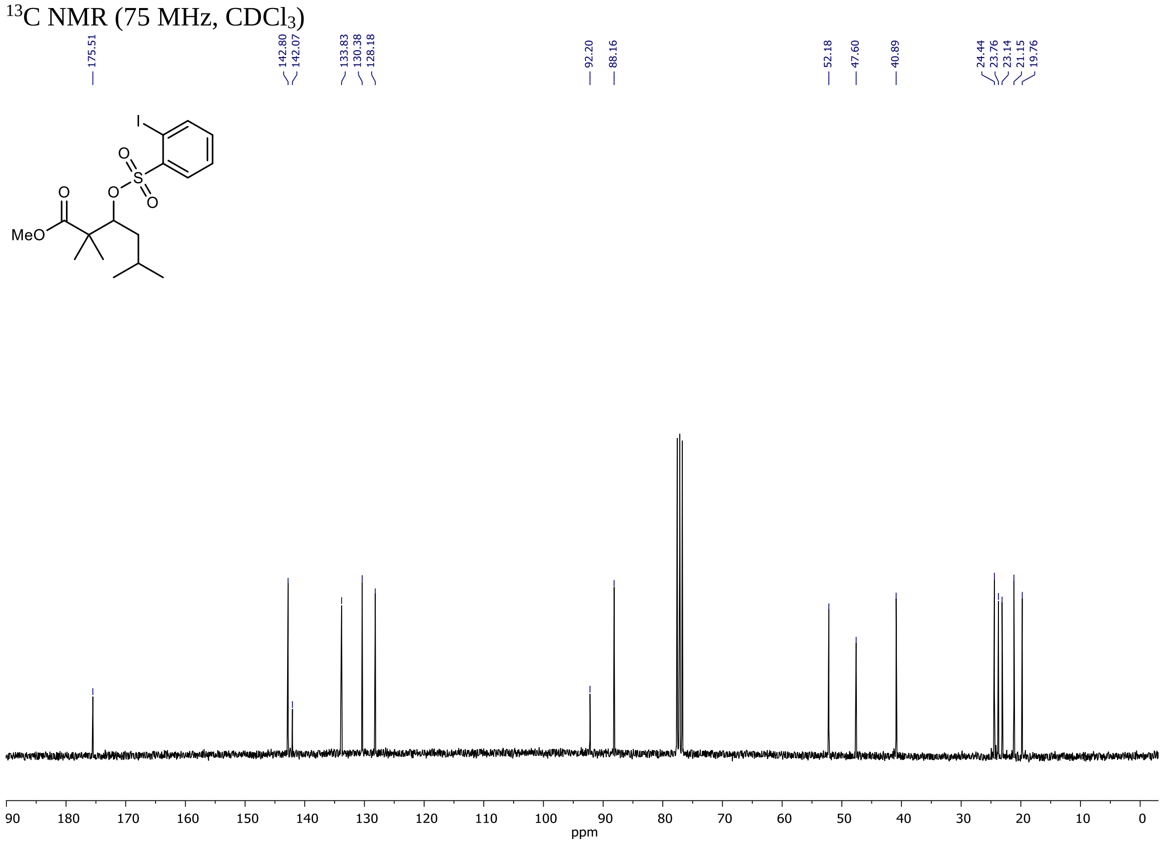

Supplementary Figure 88: NMR Spectra of Methyl 3-(((2-iodophenyl)sulfonyl)oxy)-2,2,5-trimethylhexanoate (2v)

^{1}H NMR (300 MHz, CDCl$_3$)

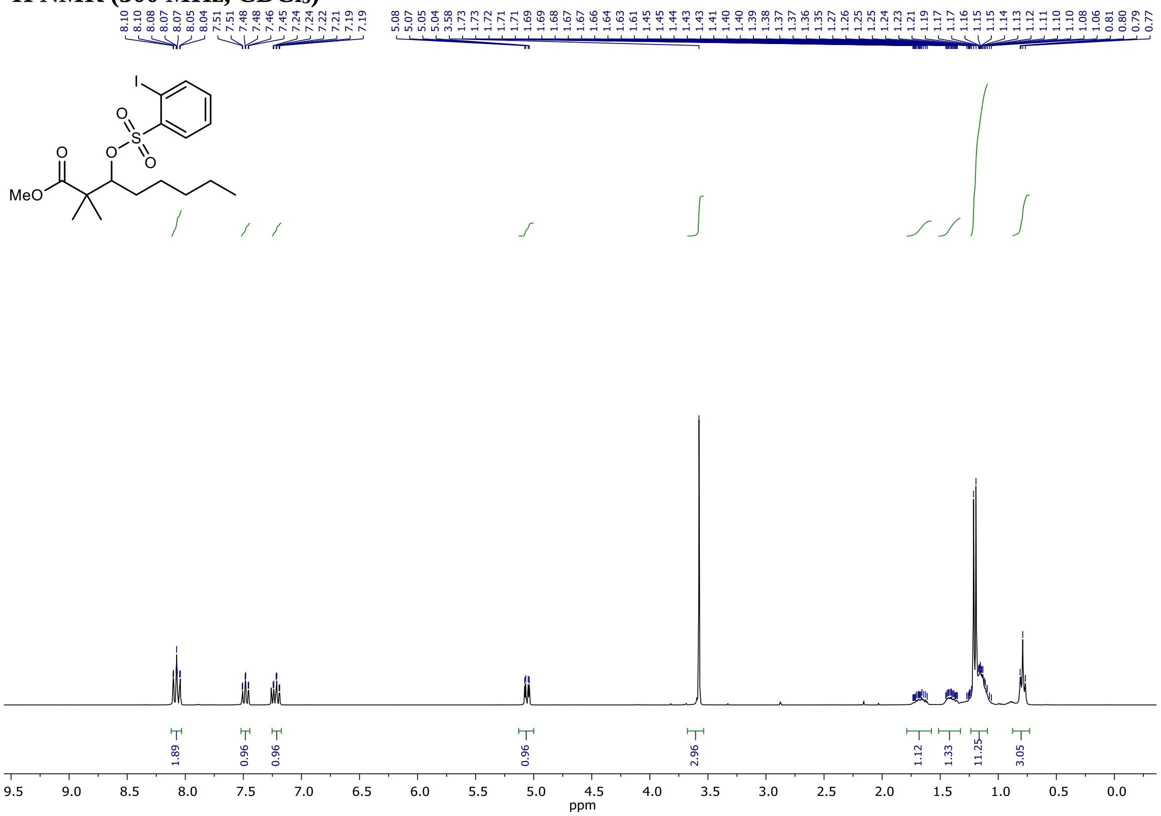

^{13}C NMR (75 MHz, CDCl$_3$)

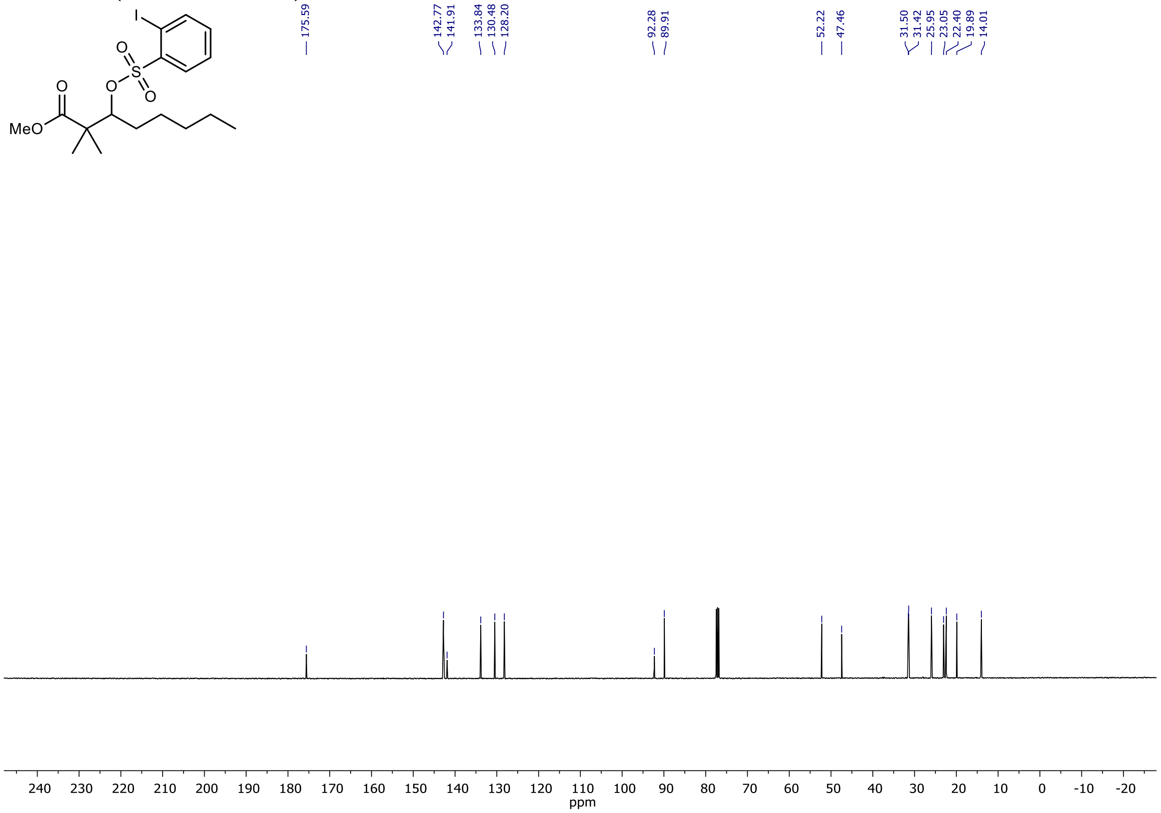

Supplementary Figure 89: NMR Spectra of Methyl 3-(((2-iodophenyl)sulfonyl)oxy)-2,2-dimethyloctanoate (2w)

^{1}H NMR (300 MHz, $CDCl_3$)

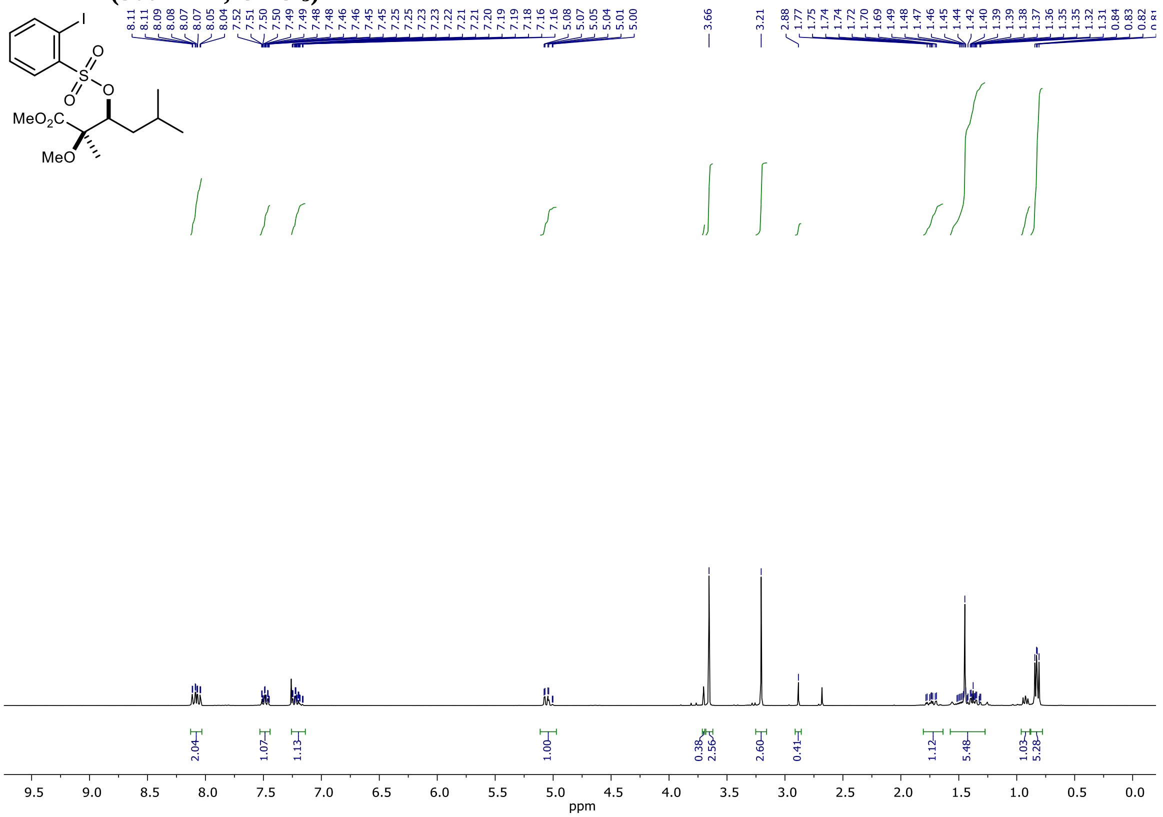

^{13}C NMR (101 MHz, $CDCl_3$)

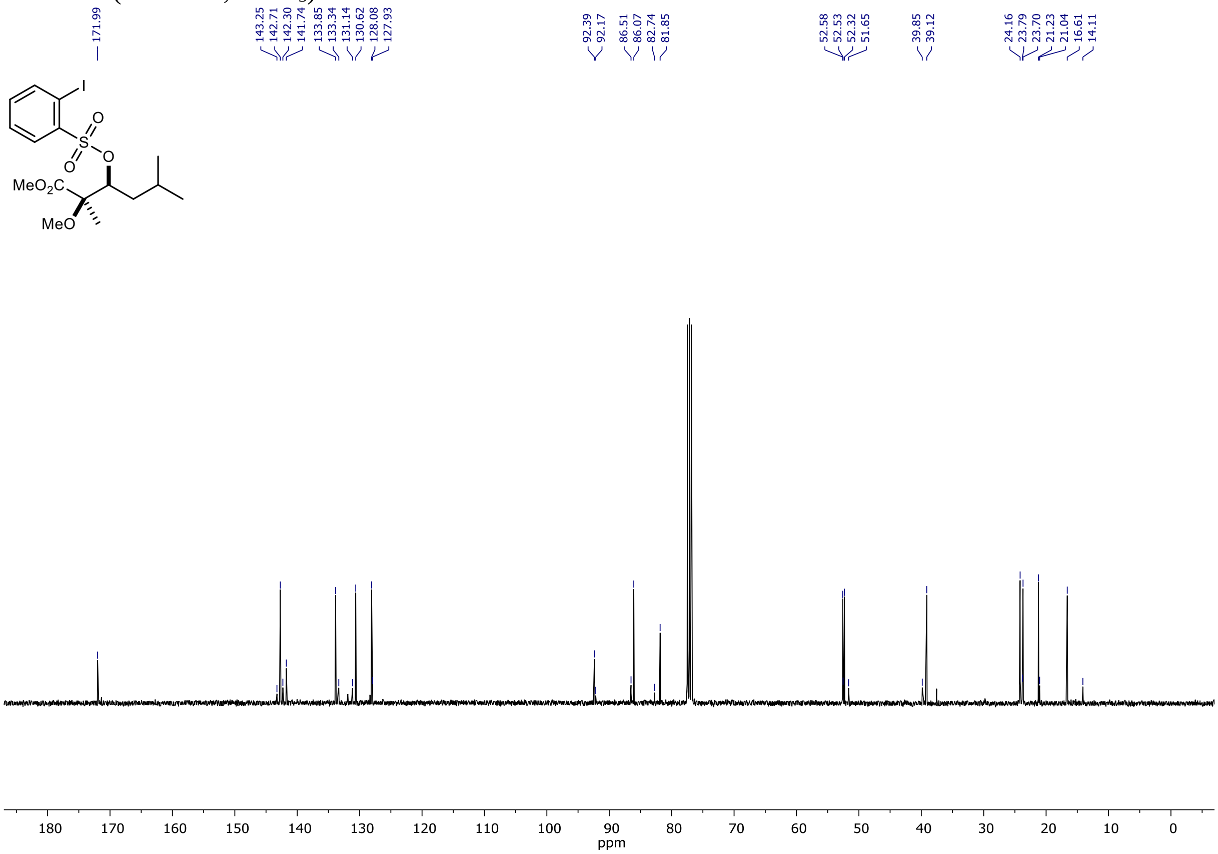

Supplementary Figure 90: NMR Spectra of *rac* Methyl (2*R*,3*S*)-3-(((2-iodophenyl)sulfonyl)oxy)-2-methoxy-2,5-dimethylhexanoate (2x)

^{1}H NMR (400 MHz, CDCl$_3$)

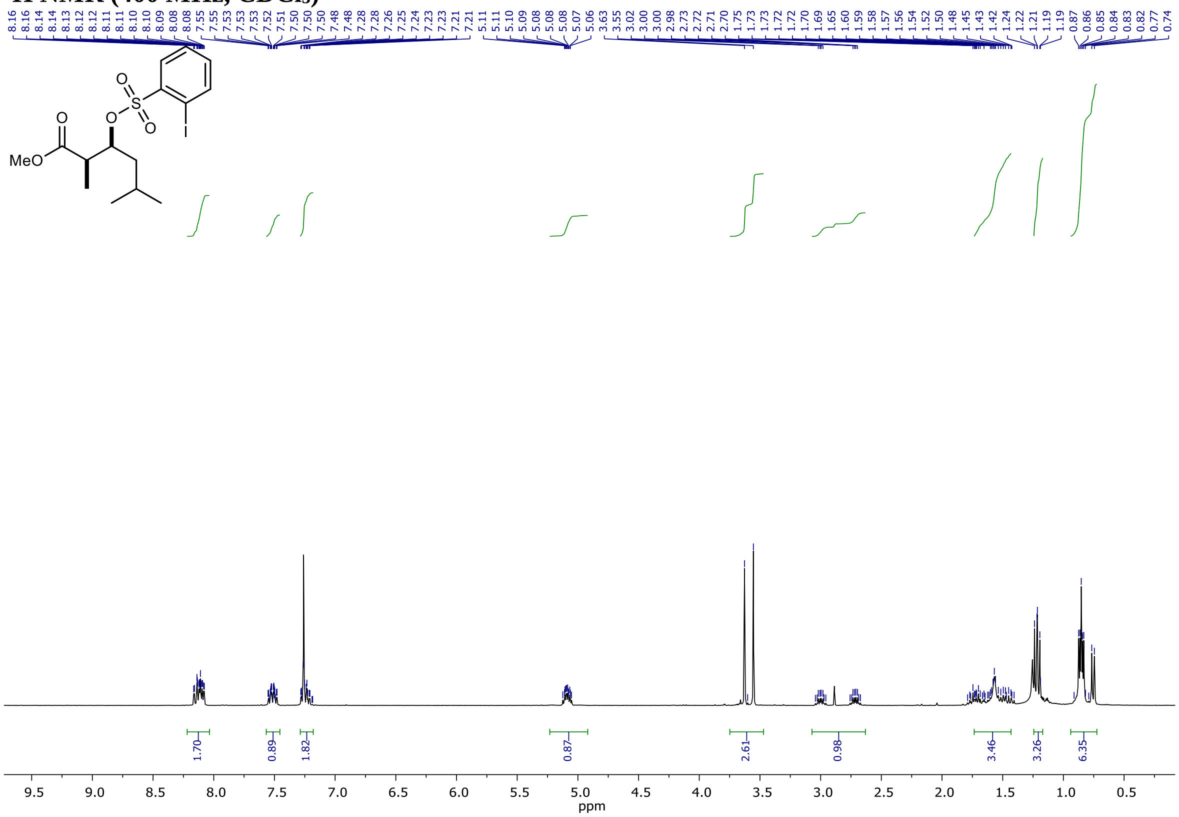

^{13}C NMR (126 MHz, CDCl$_3$)

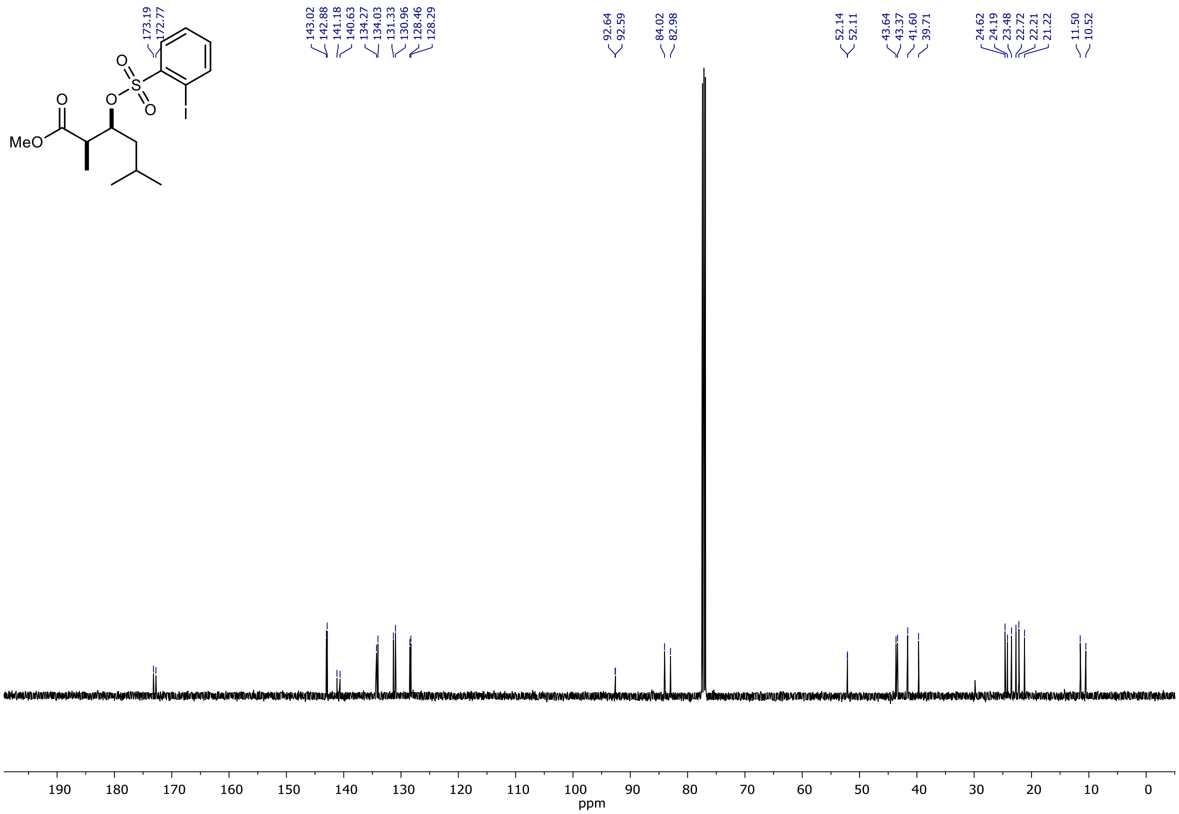

Supplementary Figure 91: NMR Spectra of *rac* Methyl (2*R*,3*S*)-3-(((2-iodophenyl)sulfonyl)oxy)-2,5-dimethylhexanoate (2y)

^{1}H NMR (300 MHz, CDCl$_3$)

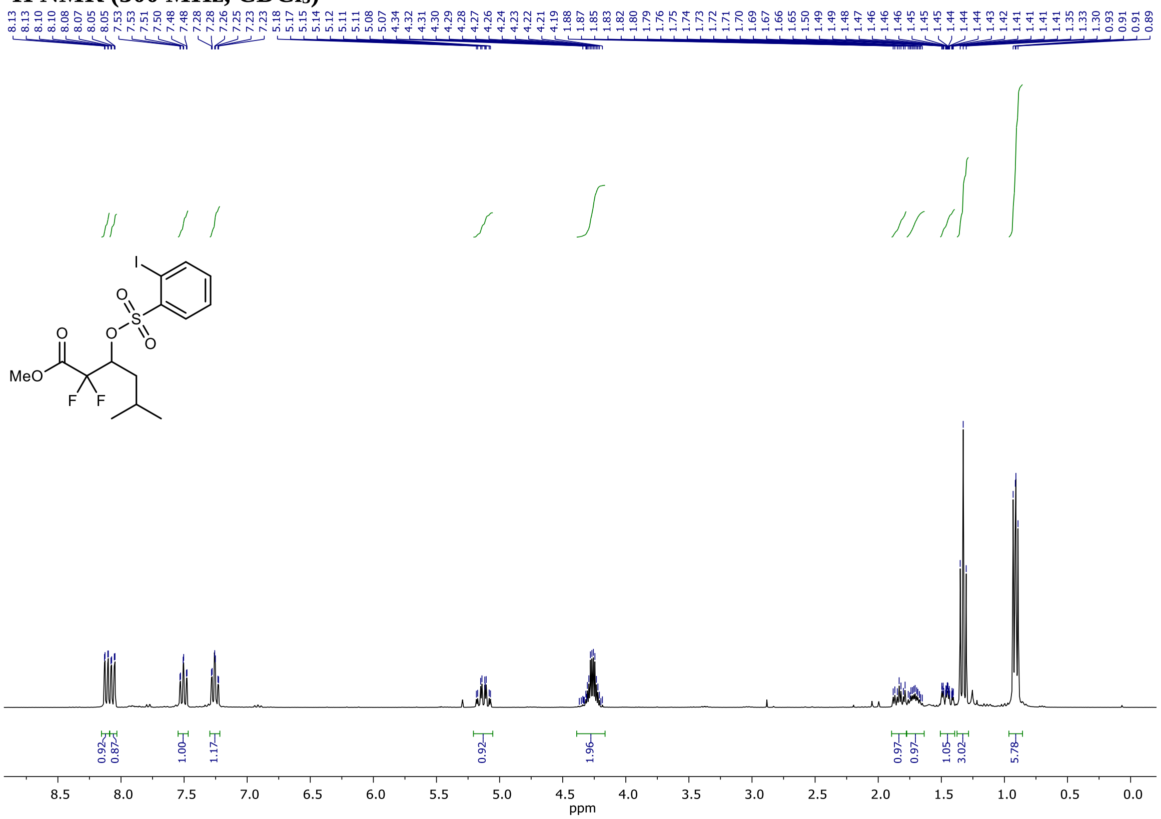

^{13}C NMR (75 MHz, CDCl$_3$)

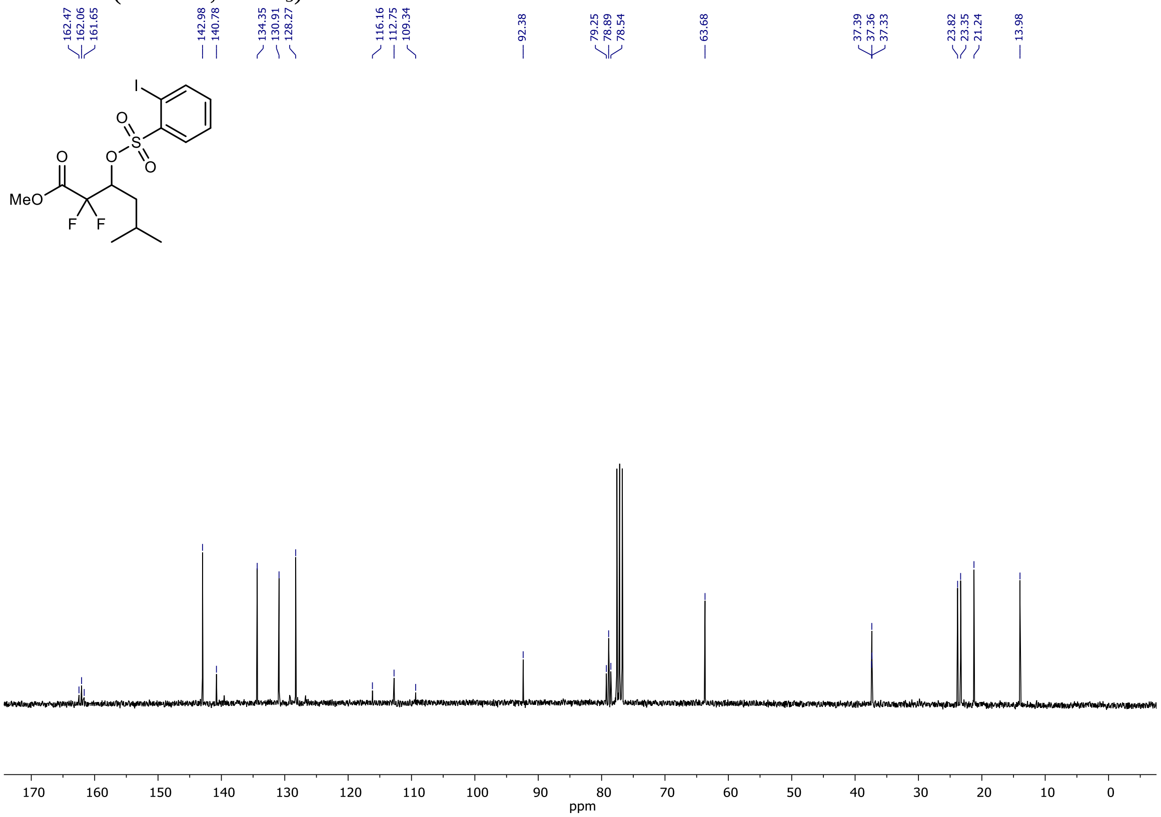

$^{19}F\{^{1}H\}$ NMR (282 Hz, $CDCl_3$)

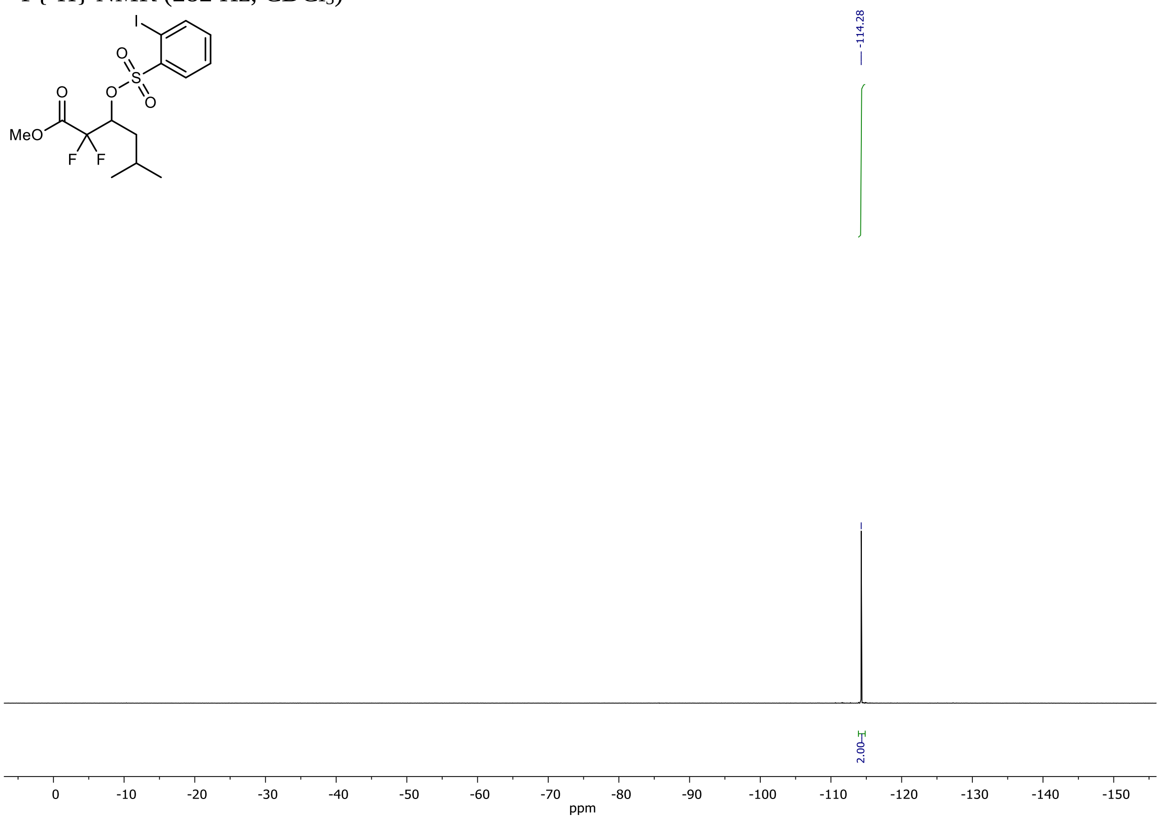

Supplementary Figure 92: NMR Spectra of Methyl 2,2-difluoro-3-(((2-iodophenyl)sulfonyl)oxy)-5-methylhexanoate (2z)

^{1}H NMR (300 MHz, $CDCl_3$)

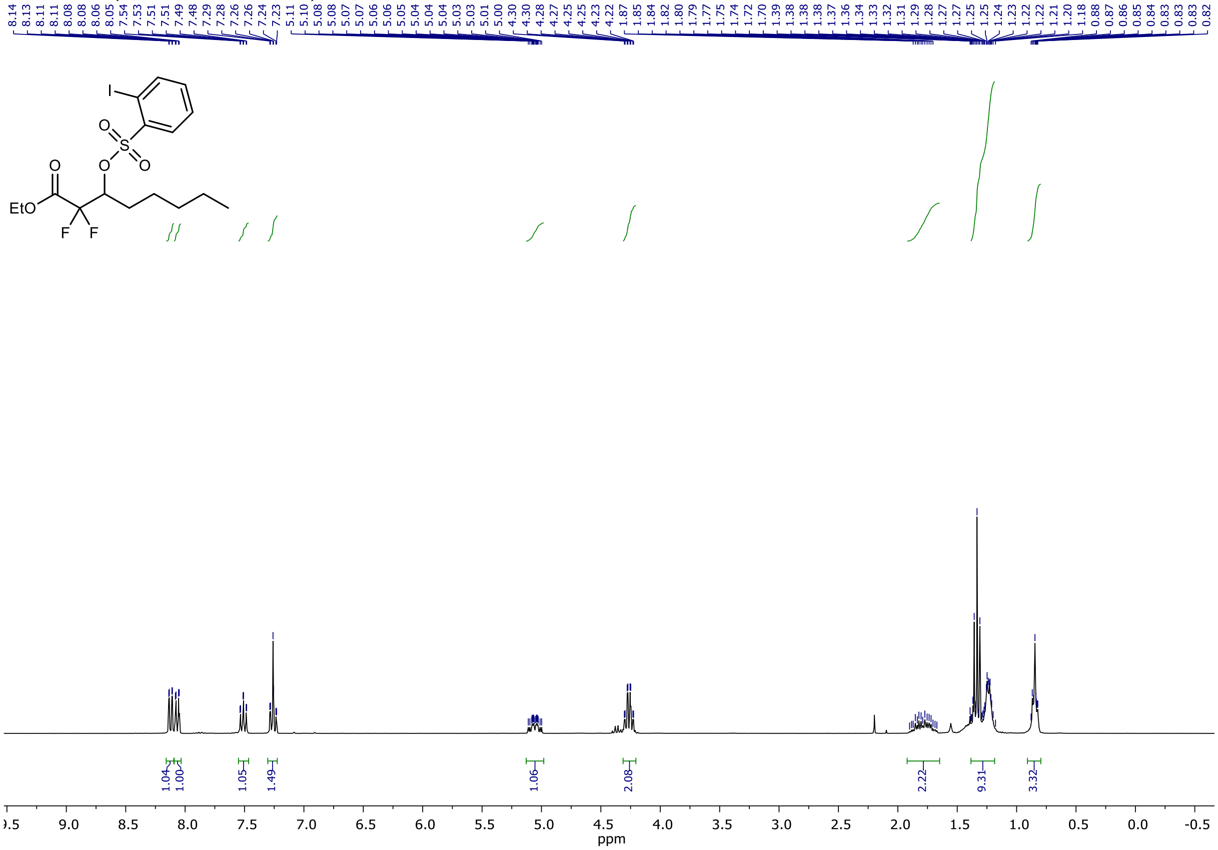

^{13}C NMR (75 MHz, CDCl$_3$)

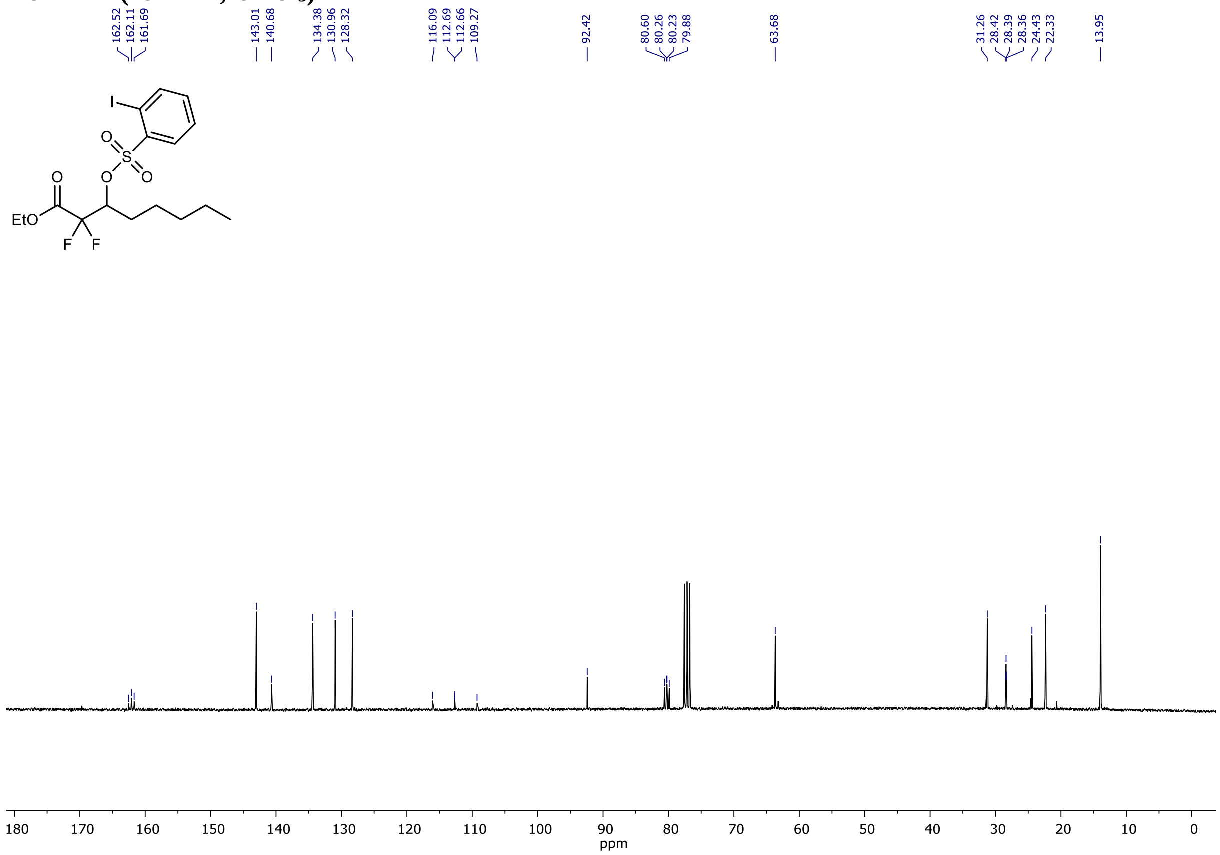

^{19}F{^{1}H} NMR (282 MHz, CDCl$_3$)

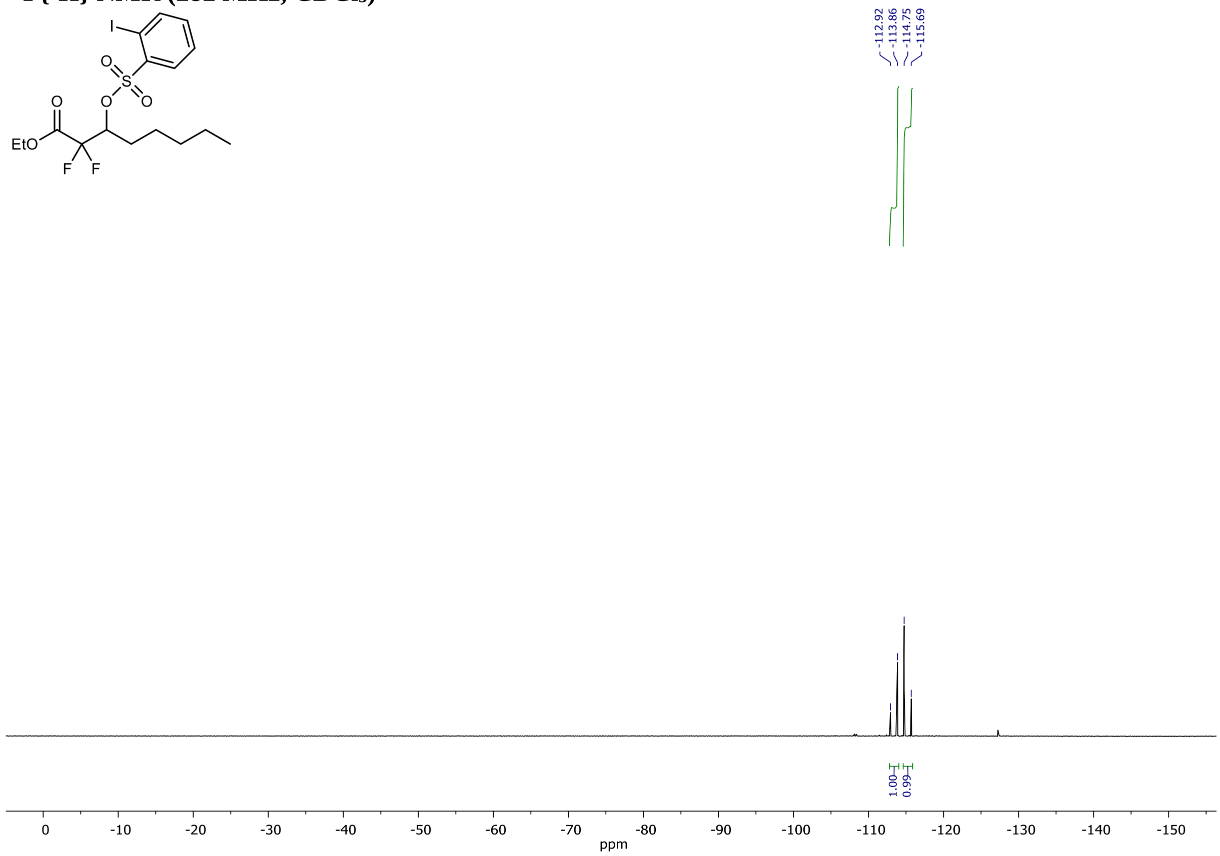

Supplementary Figure 93: NMR Spectra of Ethyl 2,2-difluoro-3-(((2-iodophenyl)sulfonyl)oxy)octanoate (2aa)

^{1}H NMR (400 MHz, $CDCl_3$)

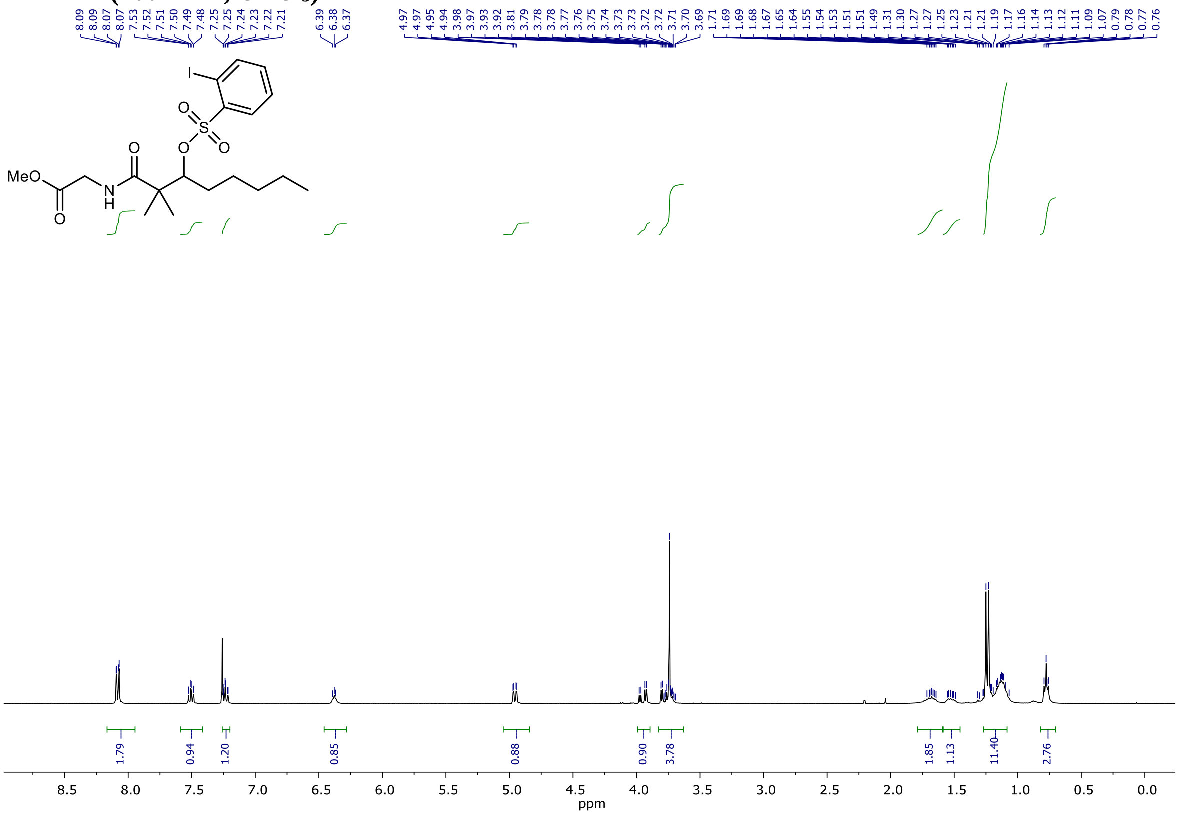

^{13}C NMR (101 MHz, $CDCl_3$)

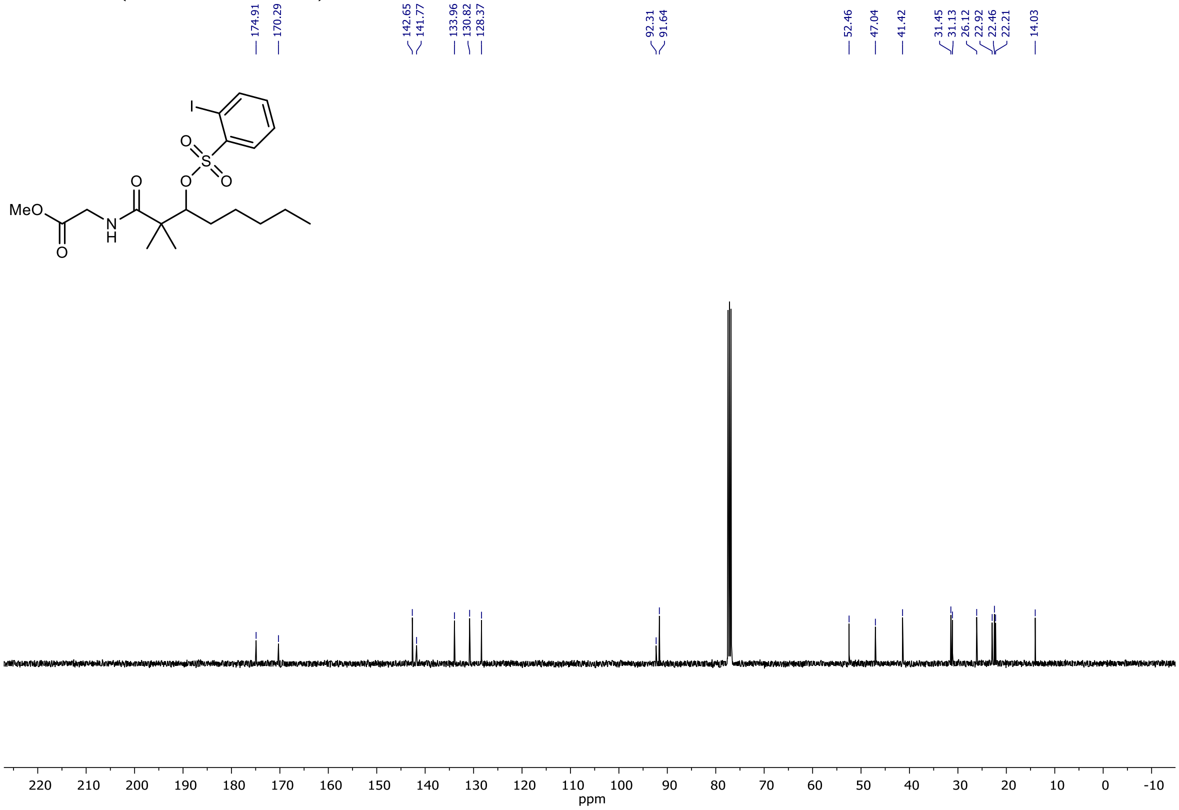

Supplementary Figure 94: NMR Spectra of Methyl (3-(((2-iodophenyl)sulfonyl)oxy)-2,2-dimethyloctanoyl)glycinate (2ab)

^{1}H NMR (300 MHz, $CDCl_3$)

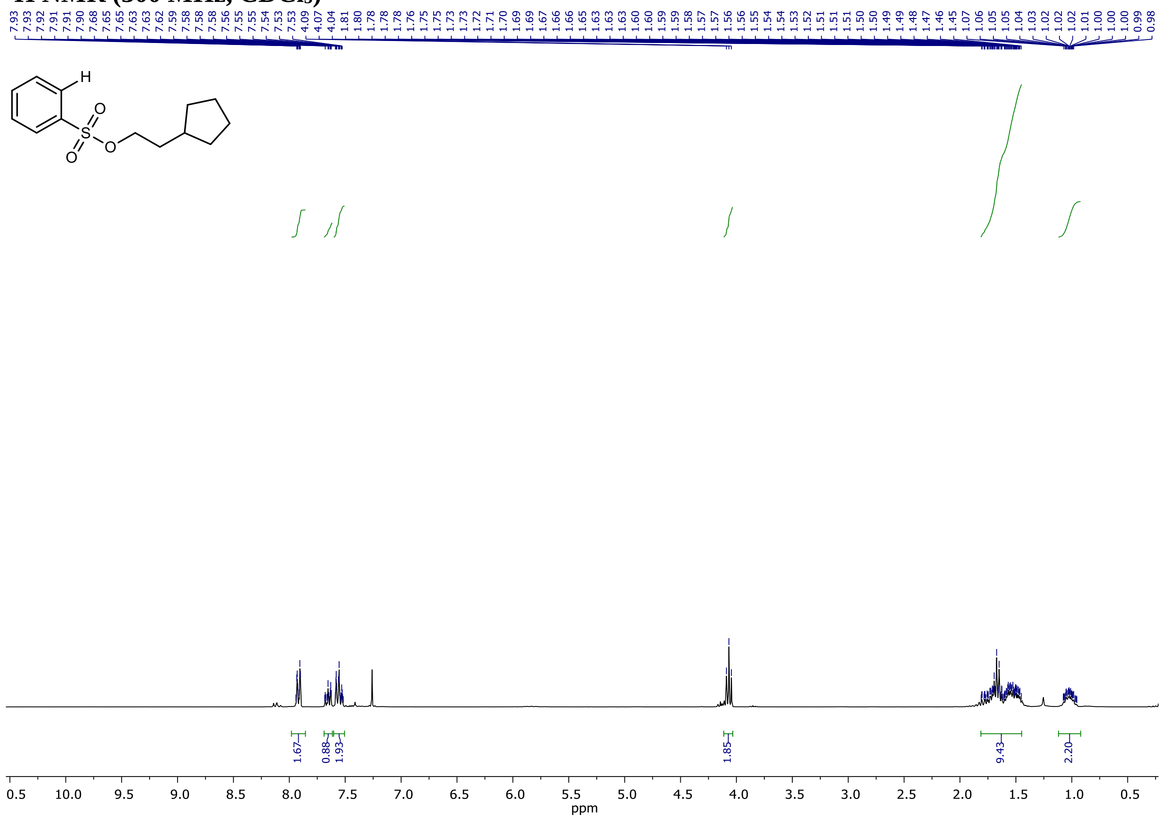

^{13}C NMR (75 MHz, $CDCl_3$)

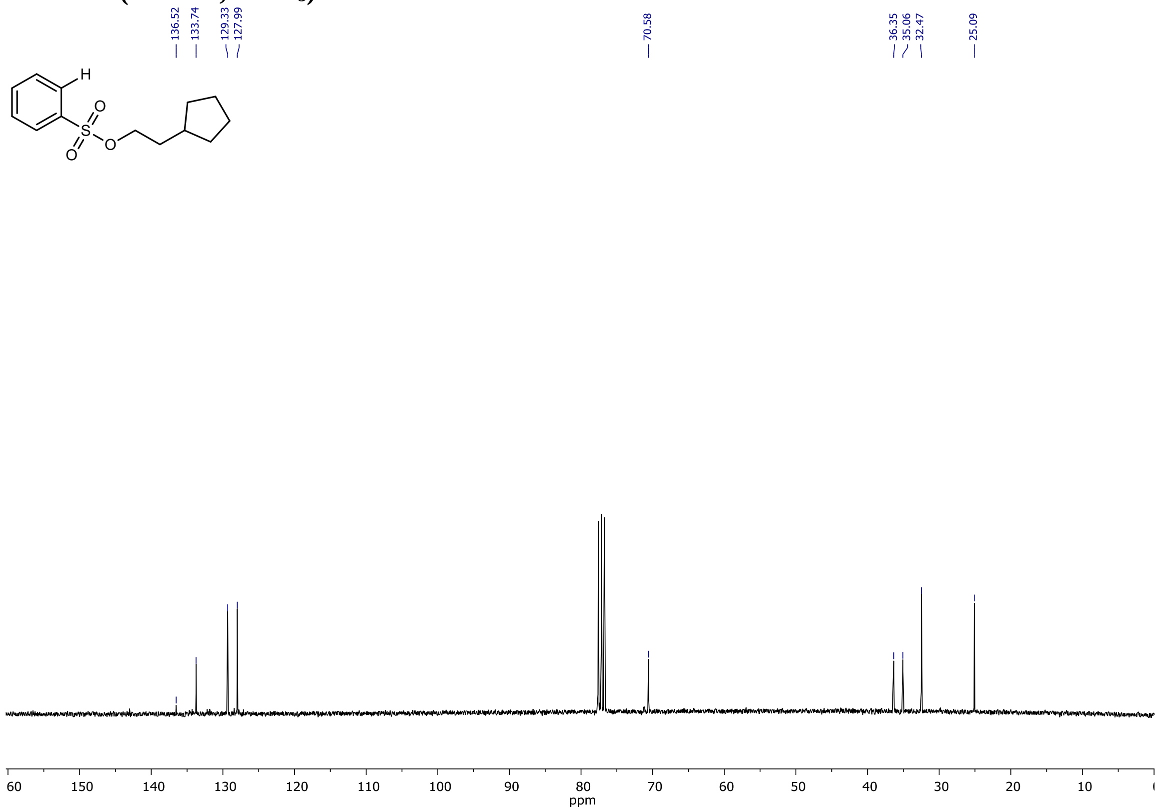

Supplementary Figure 95: NMR Spectra of 2-Cyclopentylethyl benzenesulfonate (S-24)

^{1}H NMR (300 MHz, $CDCl_3$)

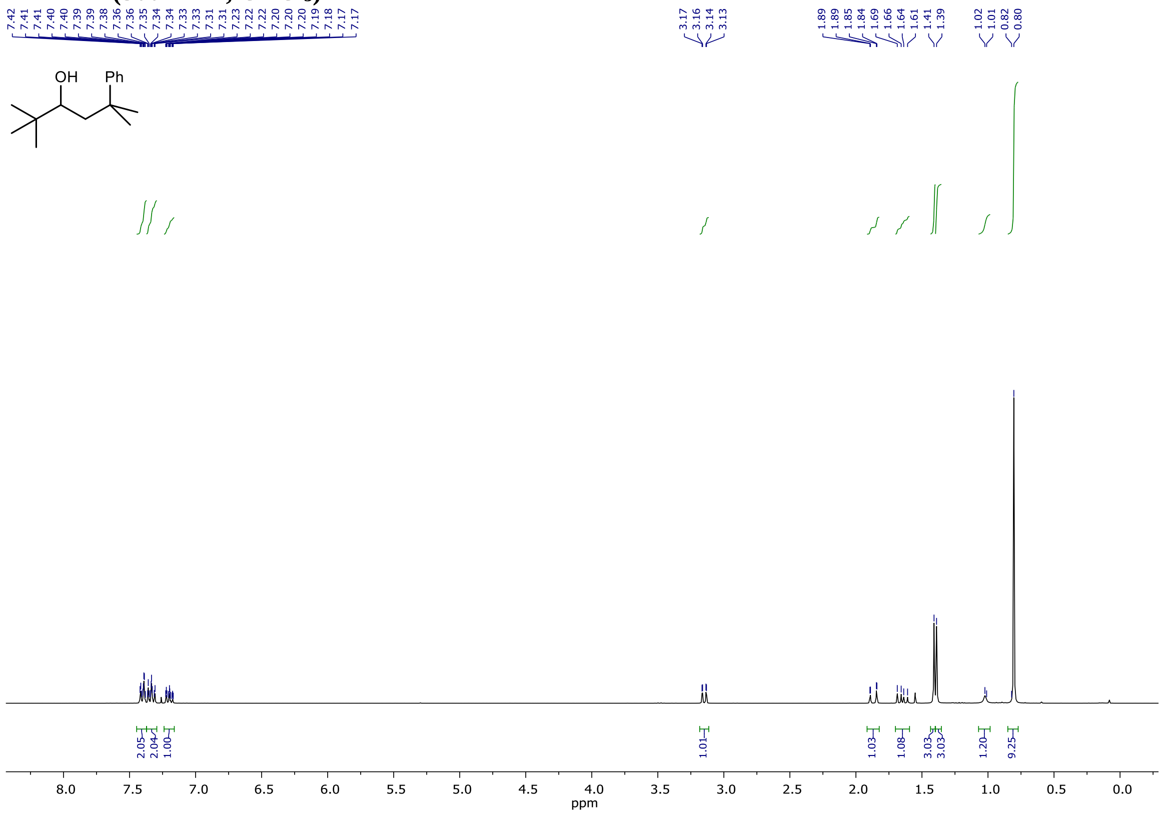

^{13}C NMR (75 MHz, $CDCl_3$)

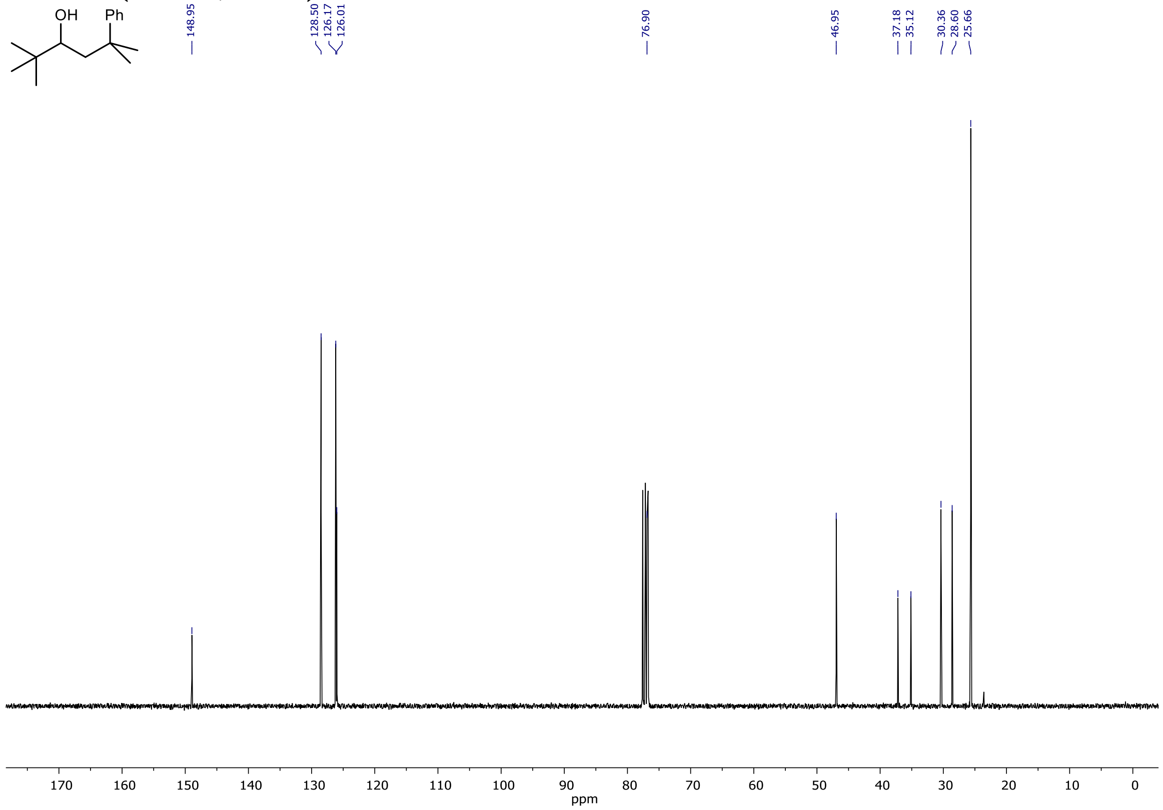

Supplementary Figure 96: NMR Spectra of 2,2,5-Trimethyl-5-phenylhexan-3-ol (3a-1)

^{1}H NMR (400 MHz, CDCl$_3$)

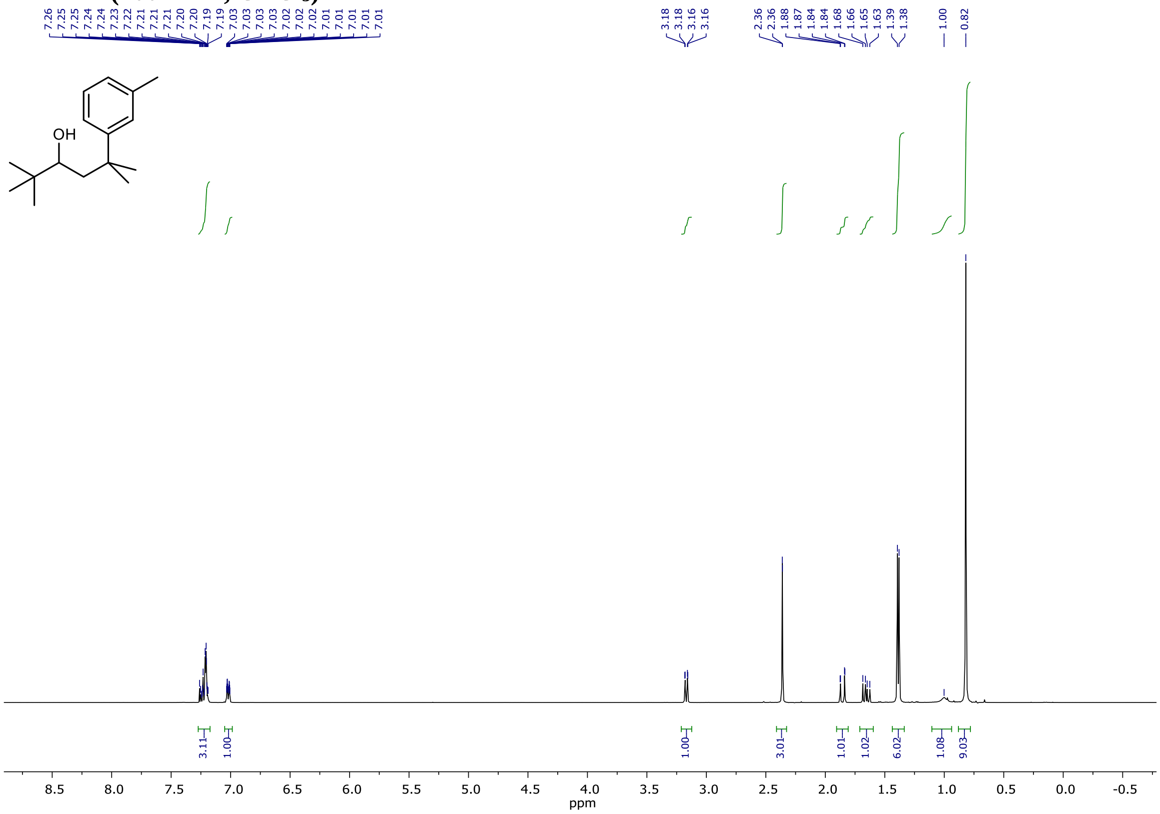

^{13}C NMR (101 MHz, CDCl$_3$)

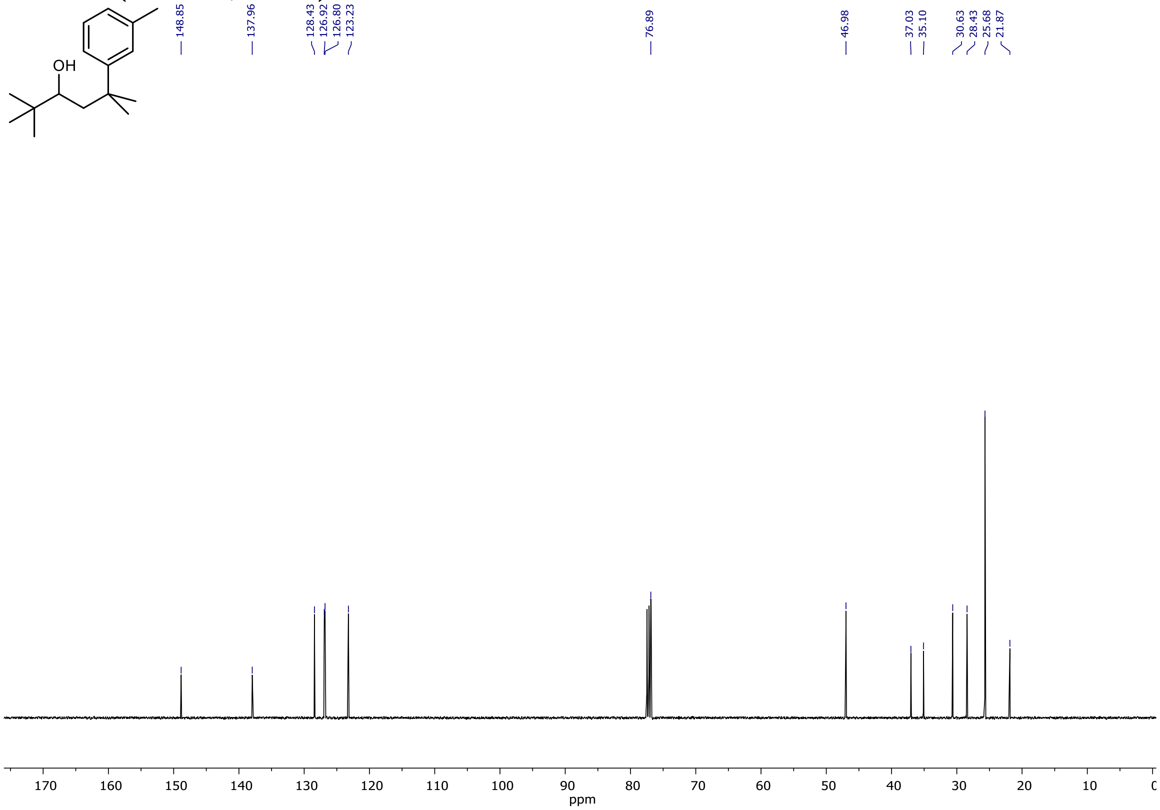

Supplementary Figure 97: NMR Spectra of 2,2,5-Trimethyl-5-(m-tolyl)hexan-3-ol (3a-2)

^{1}H NMR (300 MHz, $CDCl_3$)

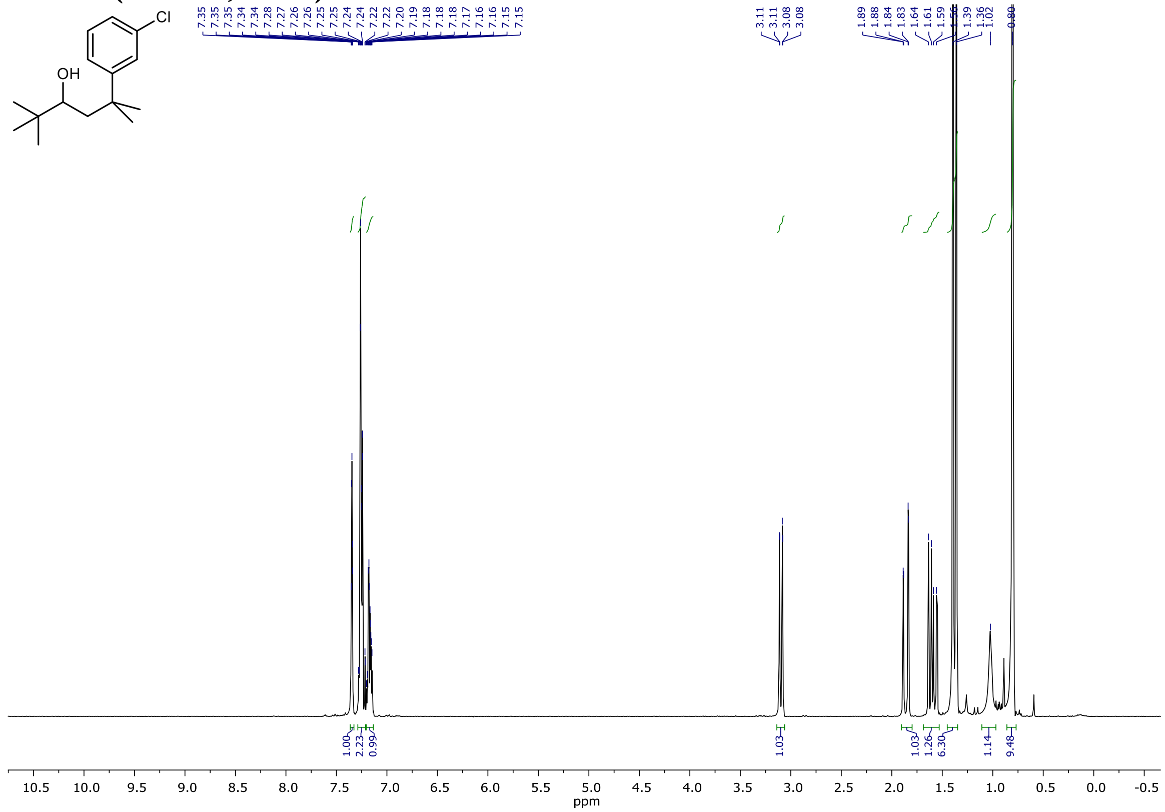

^{13}C NMR (75 MHz, $CDCl_3$)

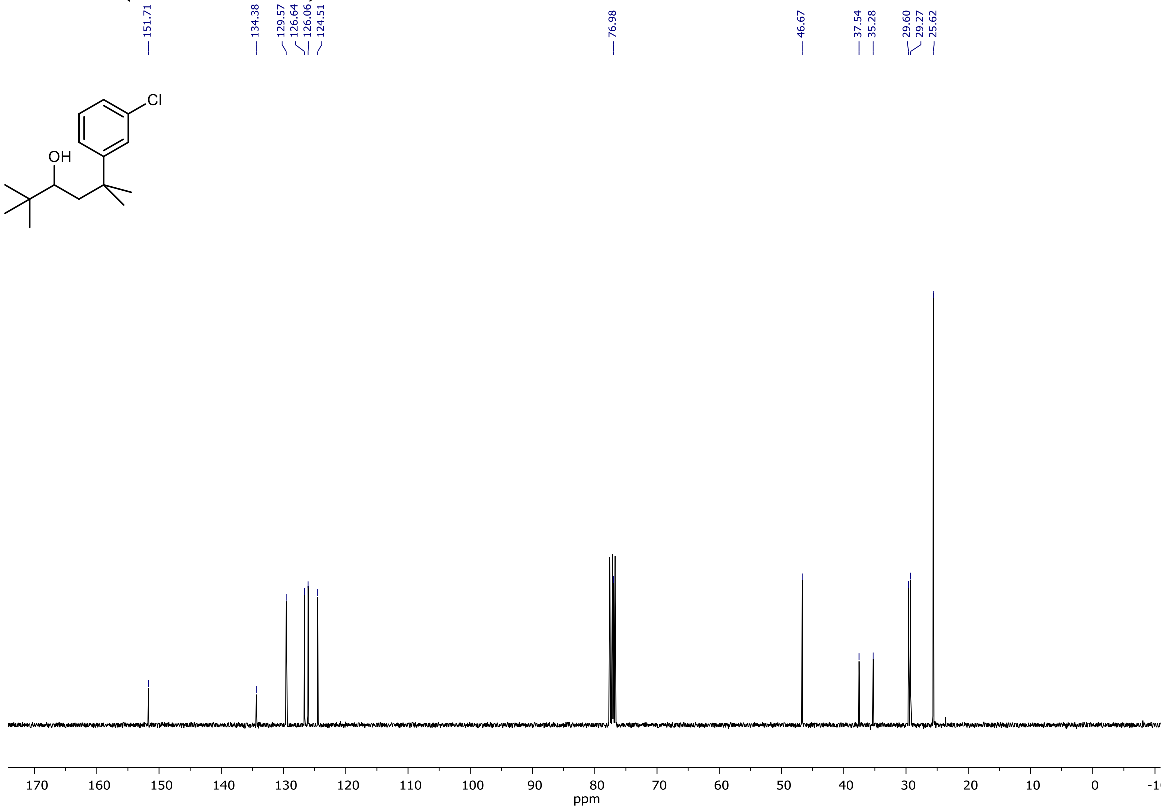

Supplementary Figure 98: NMR Spectra of 5-(3-Chlorophenyl)-2,2,5-trimethylhexan-3-ol (3a-3)

^{1}H NMR (300 MHz, $CDCl_3$)

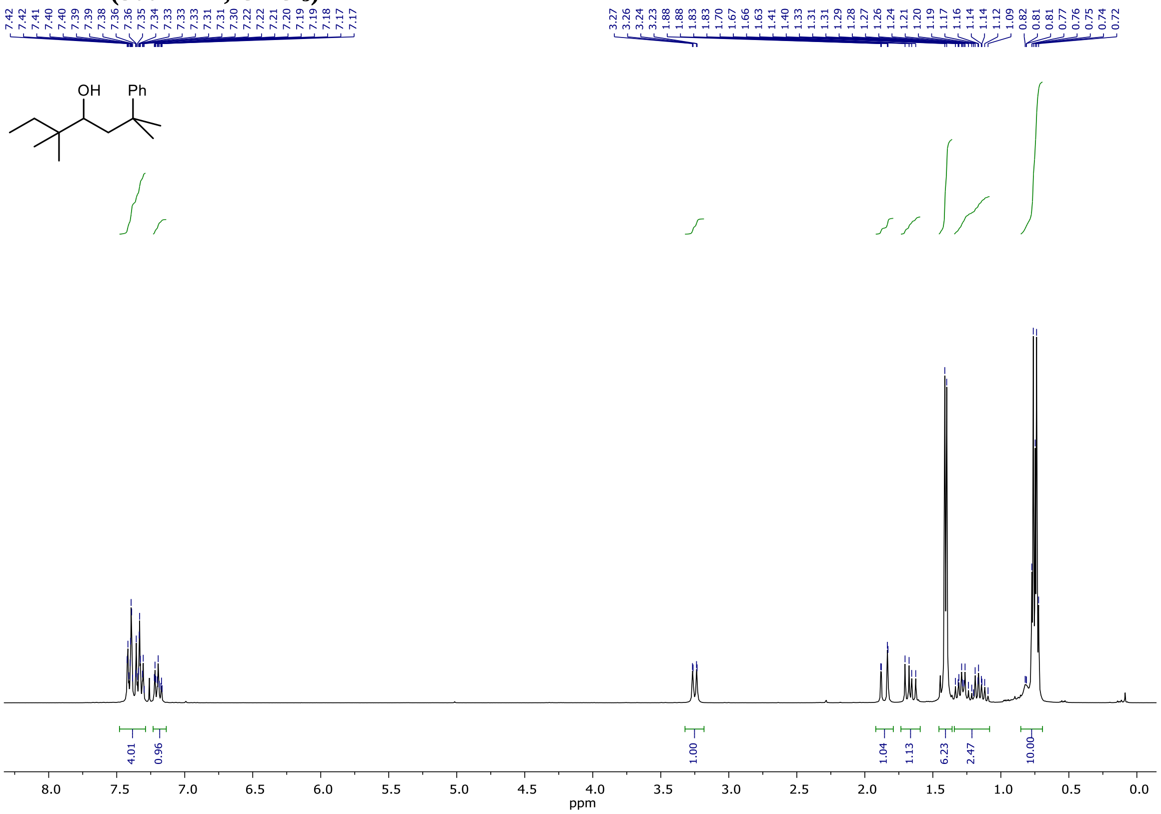

^{13}C NMR (75 MHz, $CDCl_3$)

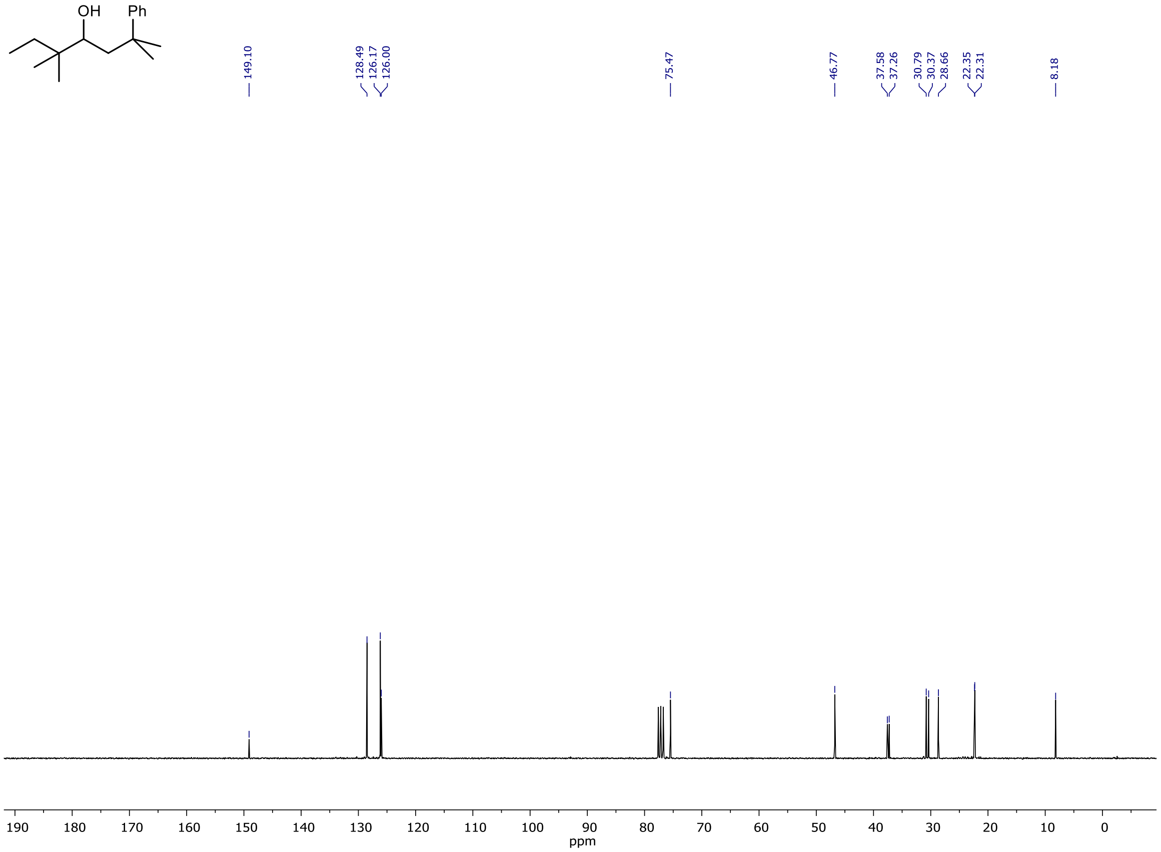

Supplementary Figure 99: NMR Spectra of 2,5,5-Trimethyl-2-phenylheptan-4-ol (3b)

^{1}H NMR (300 MHz, $CDCl_3$)

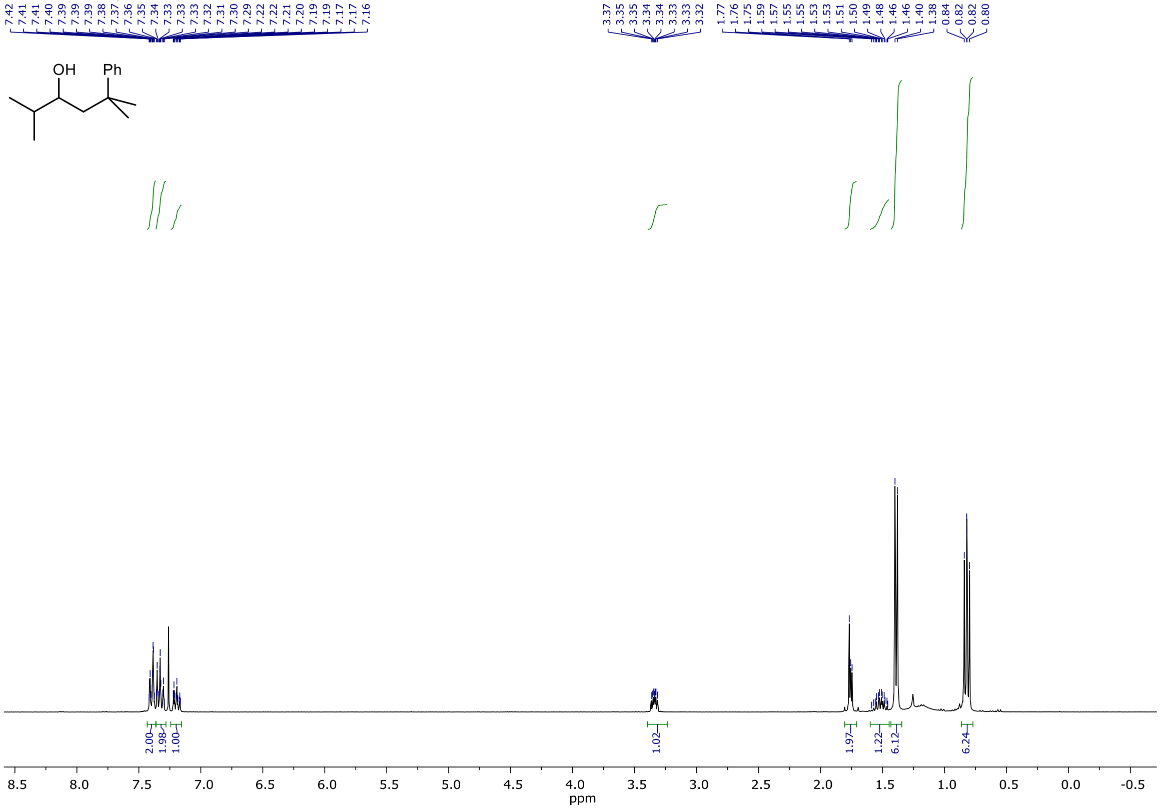

^{13}C NMR (101 MHz, $CDCl_3$)

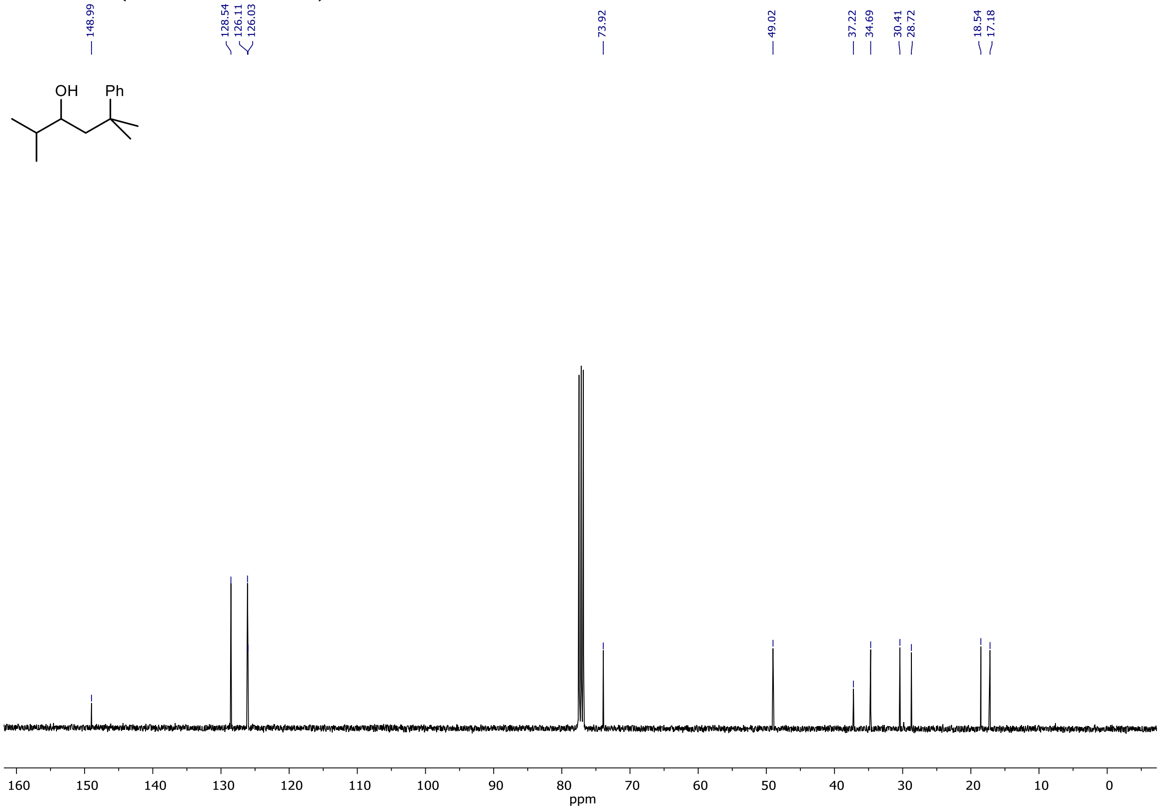

Supplementary Figure 100: NMR Spectra of 2,5-Dimethyl-5-phenylhexan-3-ol (3c)

^{1}H NMR (300 MHz, $CDCl_3$)

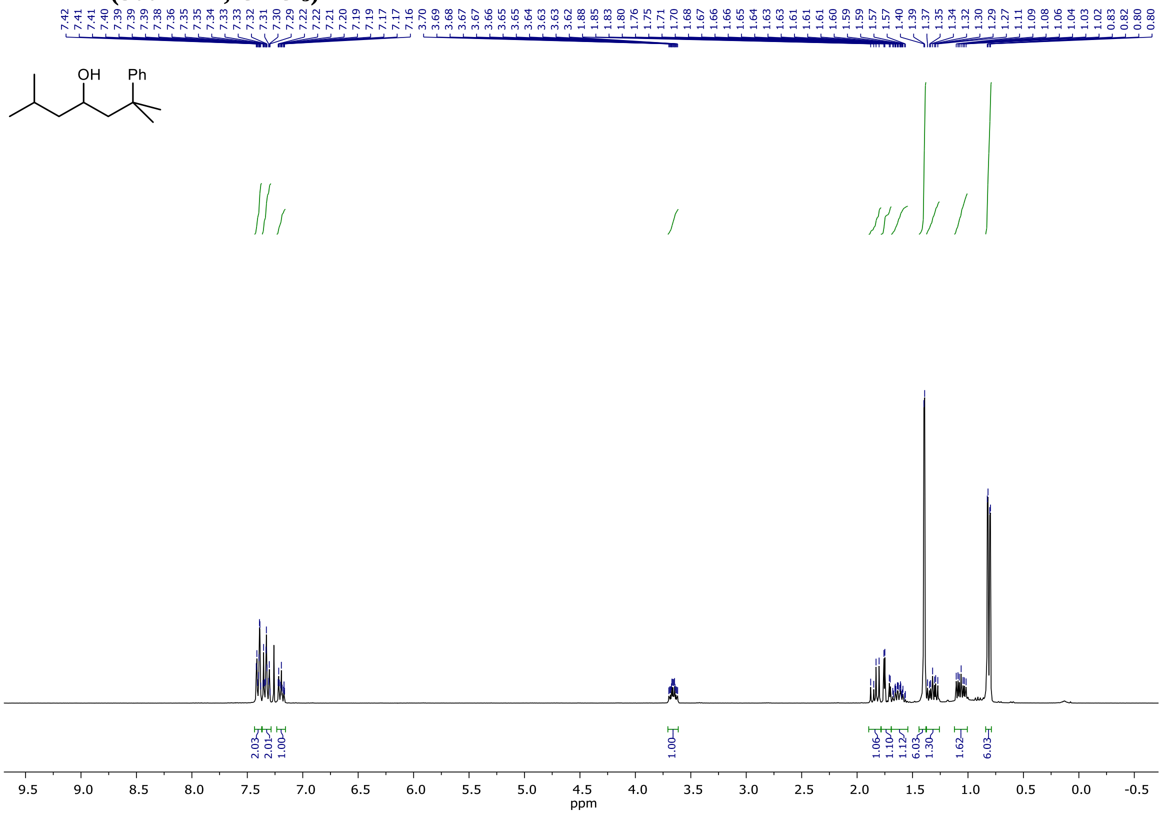

^{13}C NMR (75 MHz, $CDCl_3$)

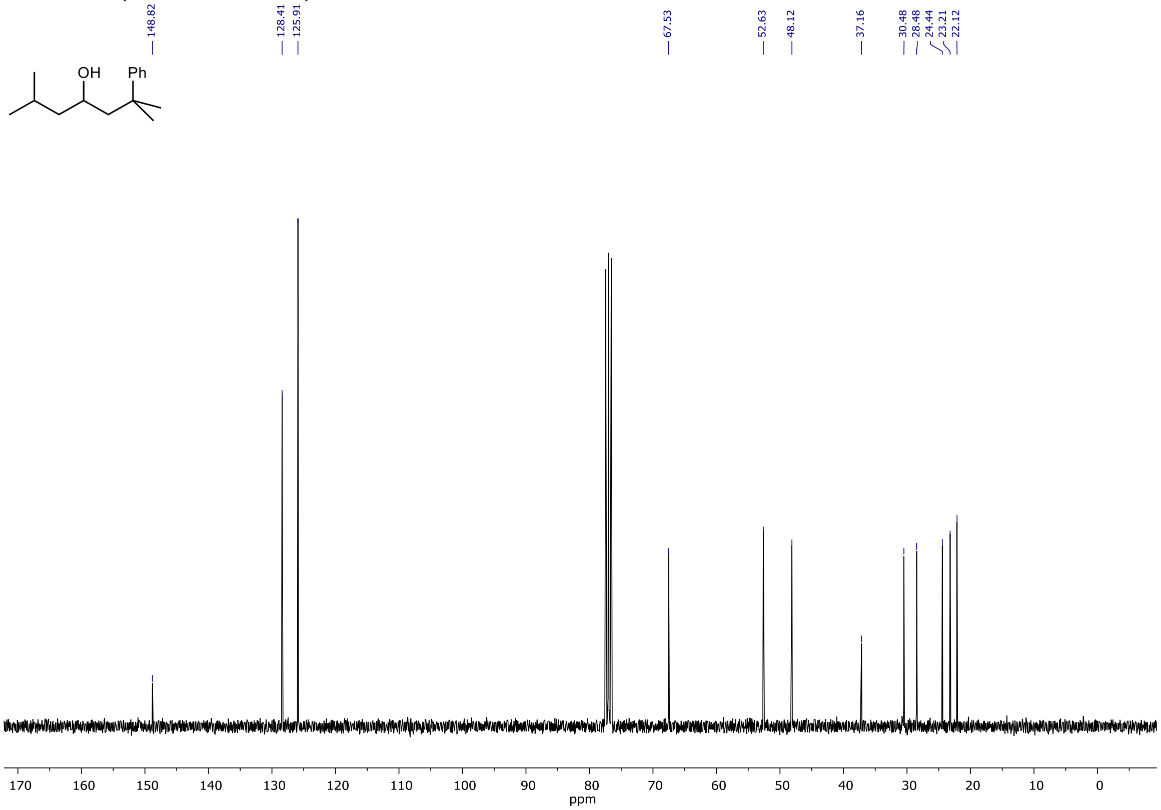

Supplementary Figure 101: NMR Spectra of 2,6-Dimethyl-2-phenylheptan-4-ol (3d)

^{1}H NMR (300 MHz, $CDCl_3$)

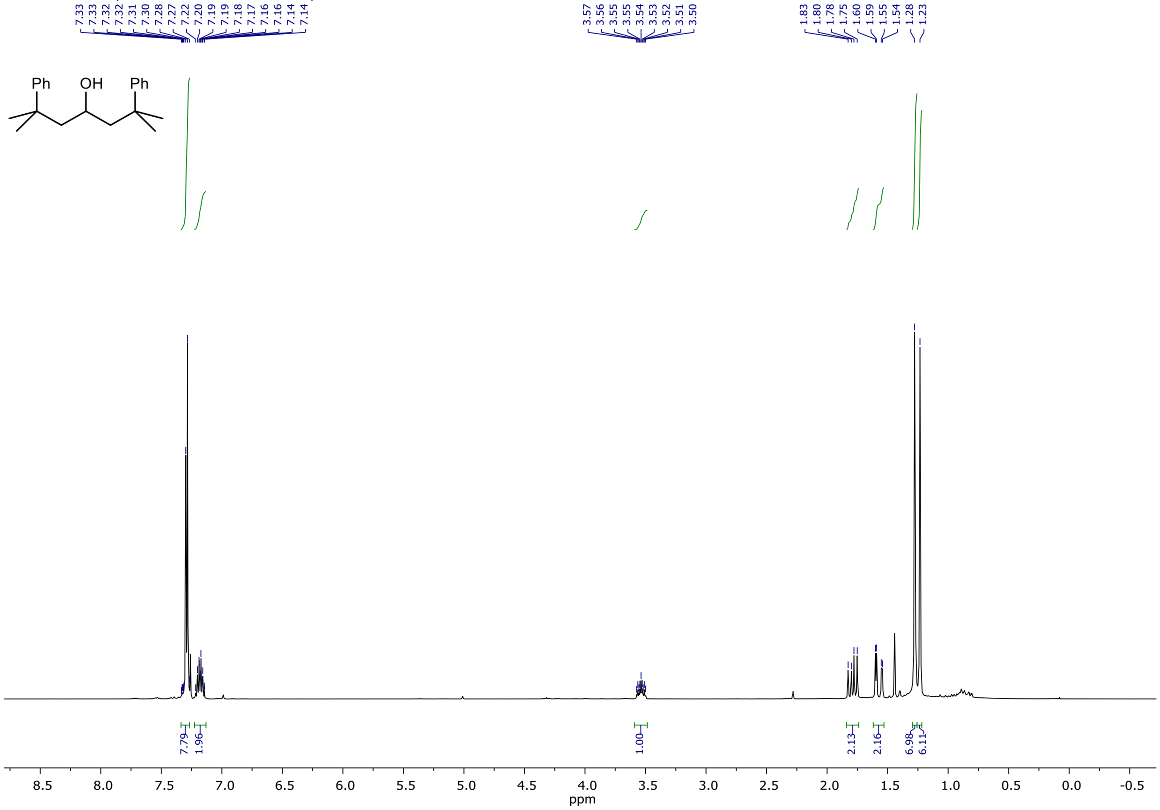

^{13}C NMR (75 MHz, $CDCl_3$)

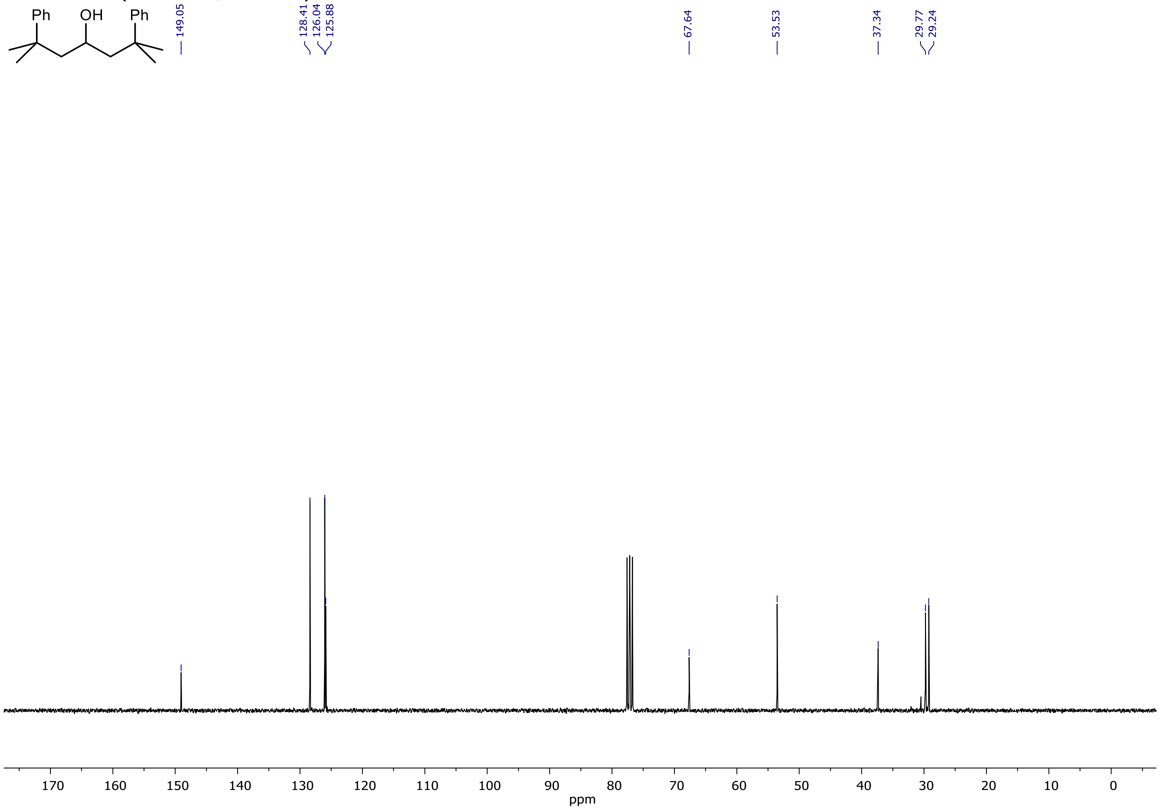

Supplementary Figure 102: NMR Spectra of 2,6-Dimethyl-2,6-diphenylheptan-4-ol (3e)

^{1}H NMR (300 MHz, $CDCl_3$)

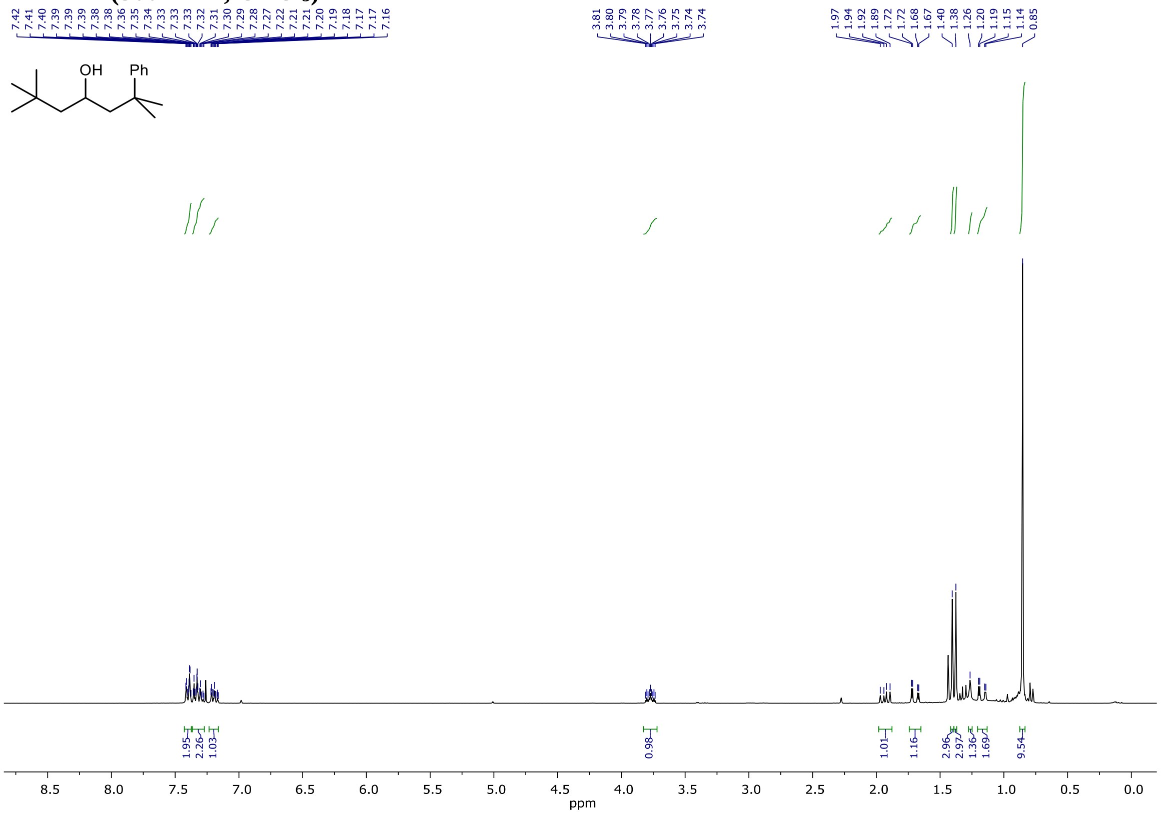

^{13}C NMR (75 MHz, $CDCl_3$)

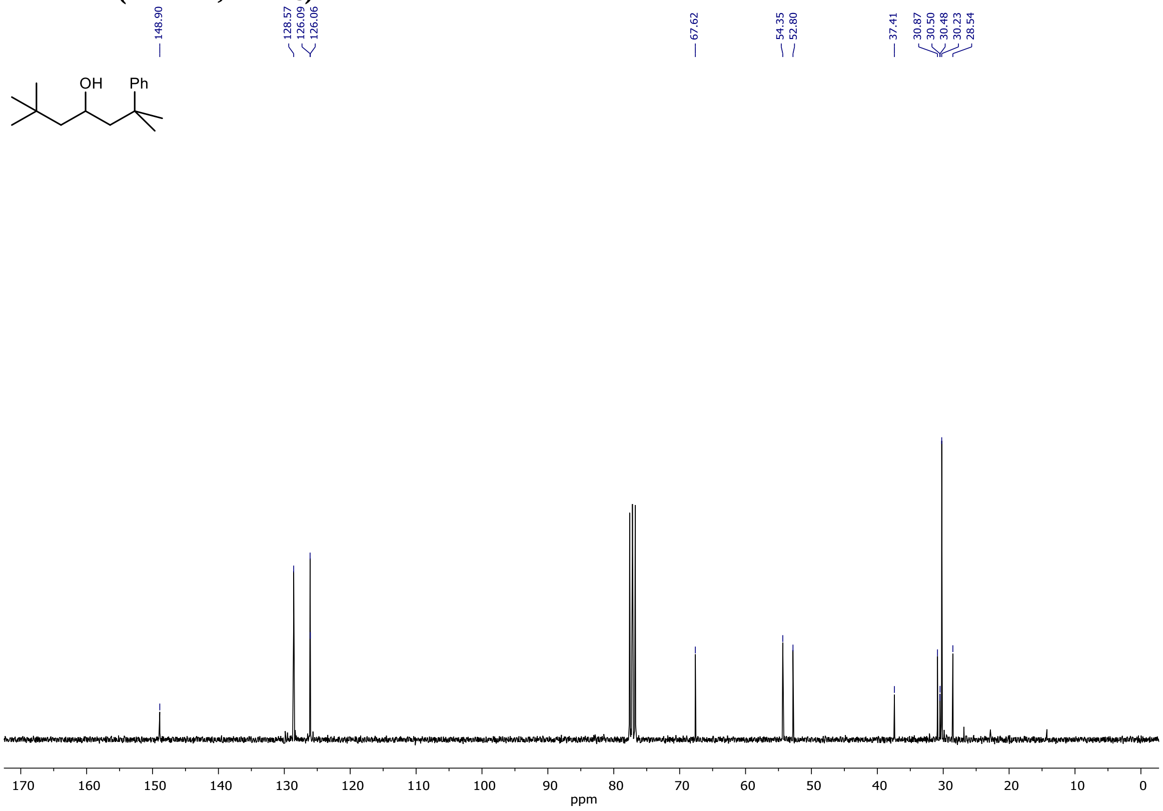

Supplementary Figure 103: NMR Spectra of 2,2,6-Trimethyl-6-phenylheptan-4-ol (3f)

^{1}H NMR (400 MHz, $CDCl_3$)

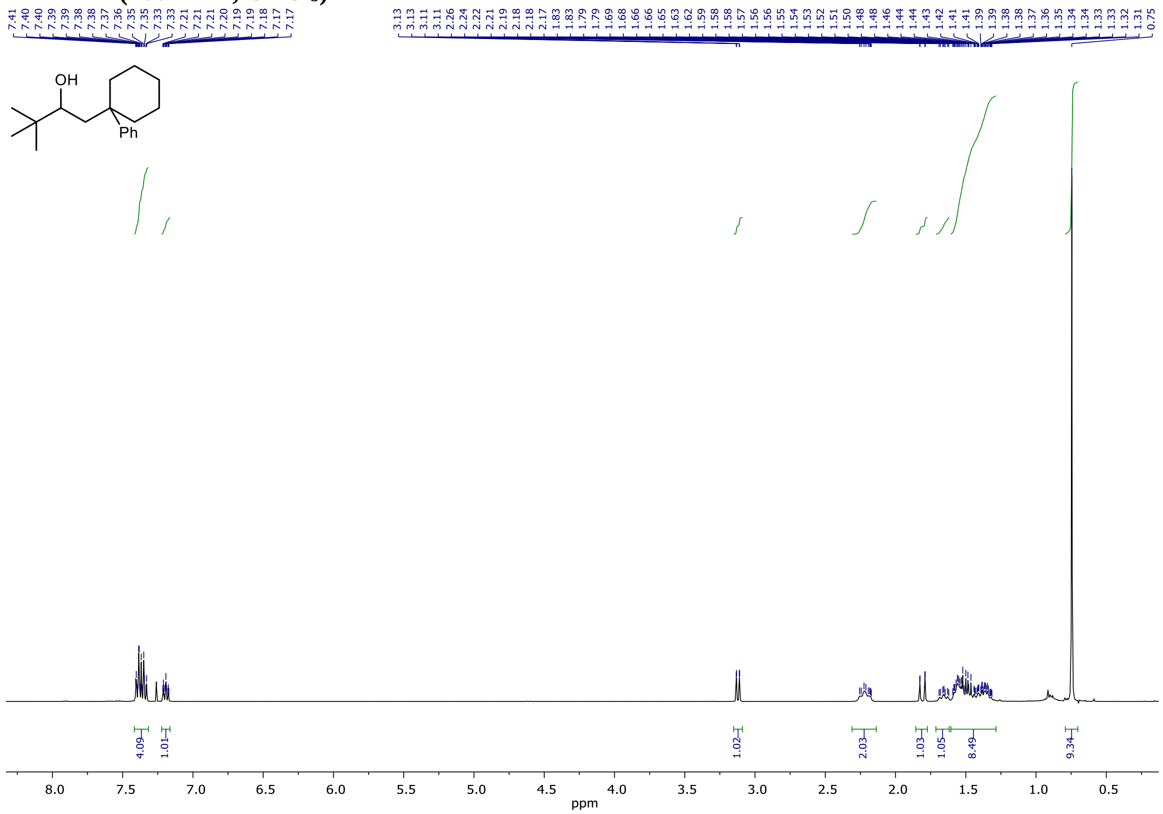

^{13}C NMR (101 MHz, $CDCl_3$)

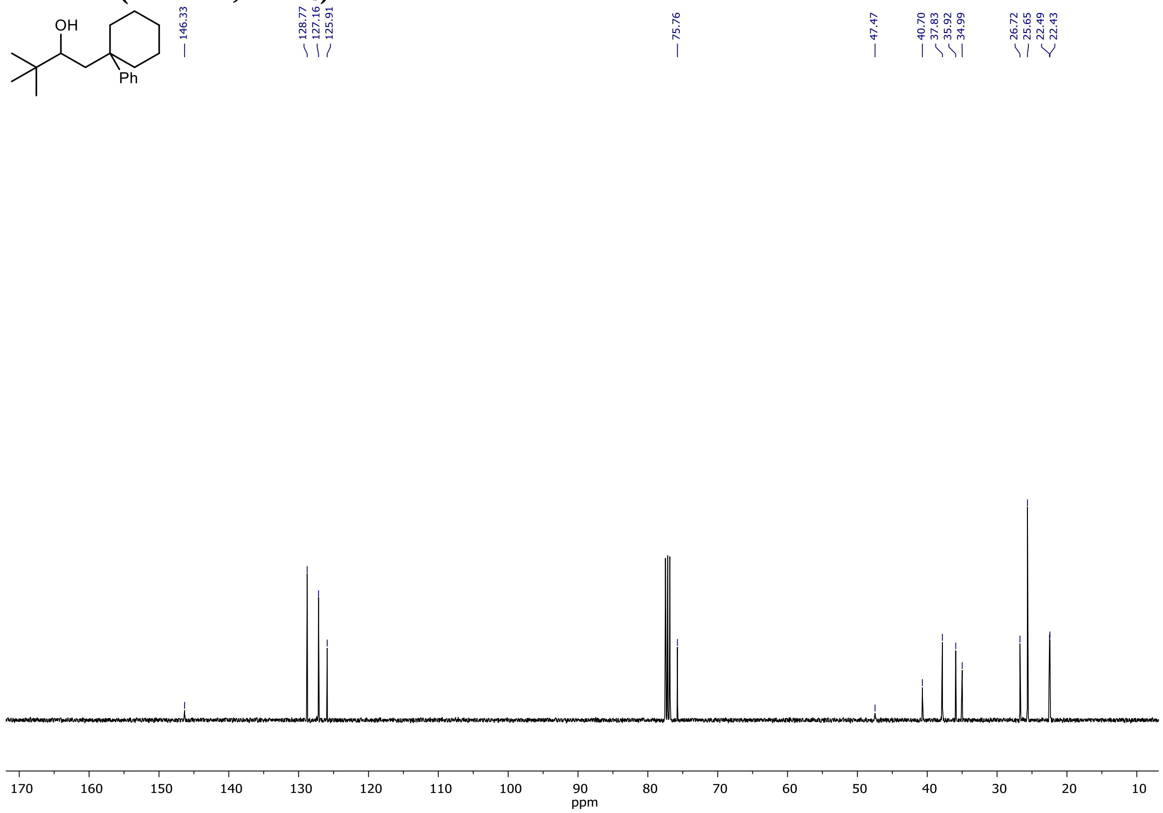

Supplementary Figure 104: NMR Spectra of 3,3-Dimethyl-1-(1-phenylcyclohexyl)butan-2-ol (3g)

^{1}H NMR (400 MHz, $CDCl_3$)

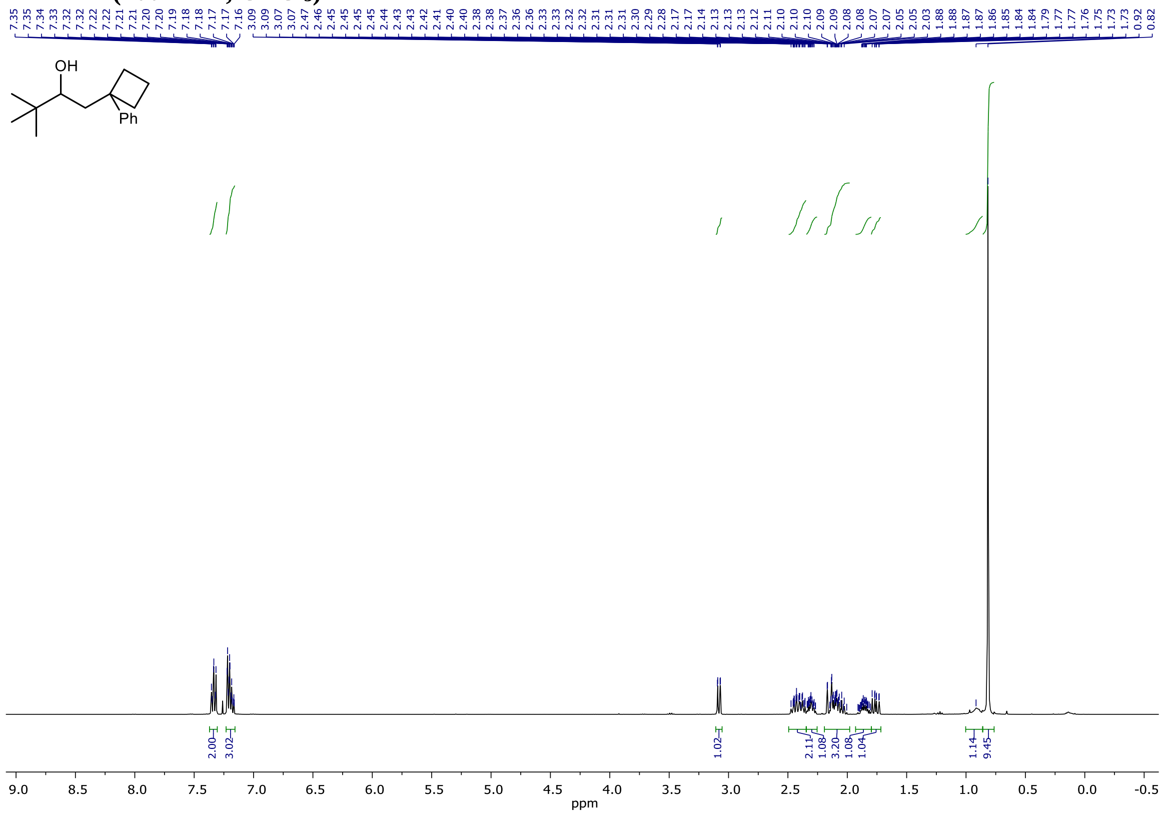

^{13}C NMR (101 MHz, $CDCl_3$)

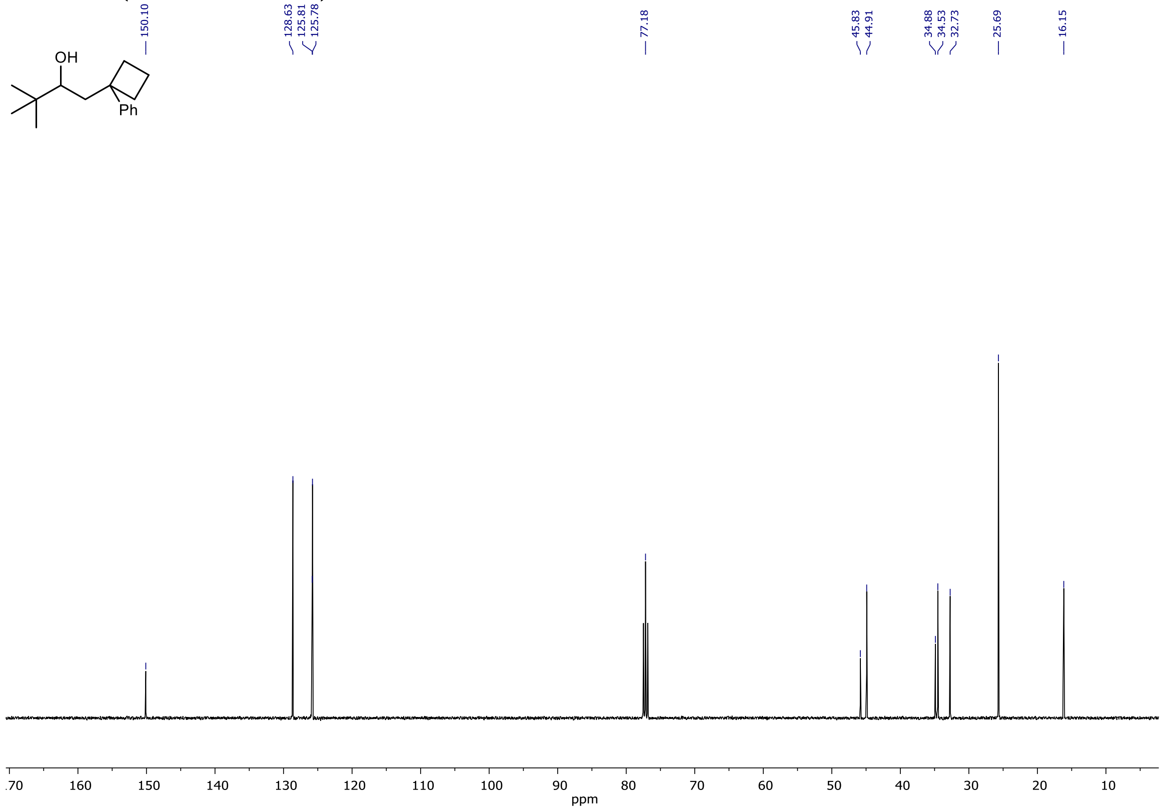

Supplementary Figure 105: NMR Spectra of 3,3-Dimethyl-1-(1-phenylcyclobutyl)butan-2-ol (3i-1)

^{1}H NMR (300 MHz, $CDCl_3$)

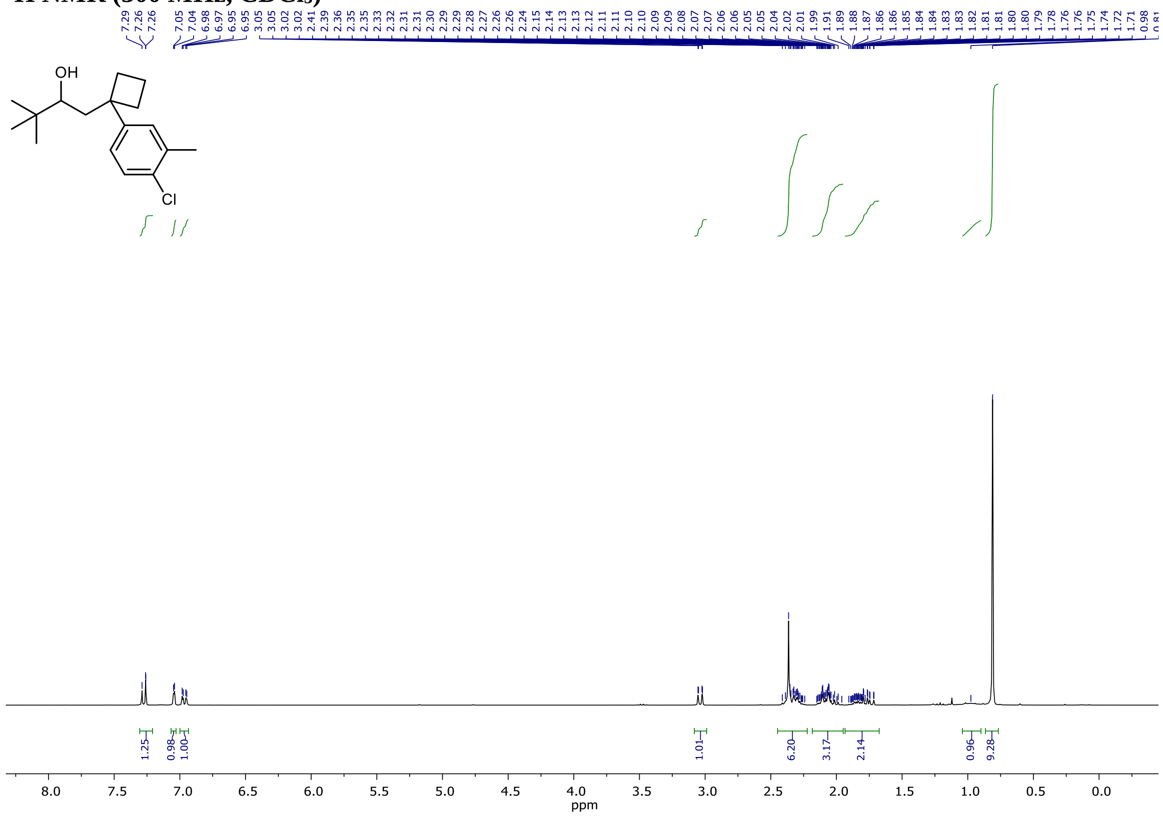

^{13}C NMR (75 MHz, $CDCl_3$)

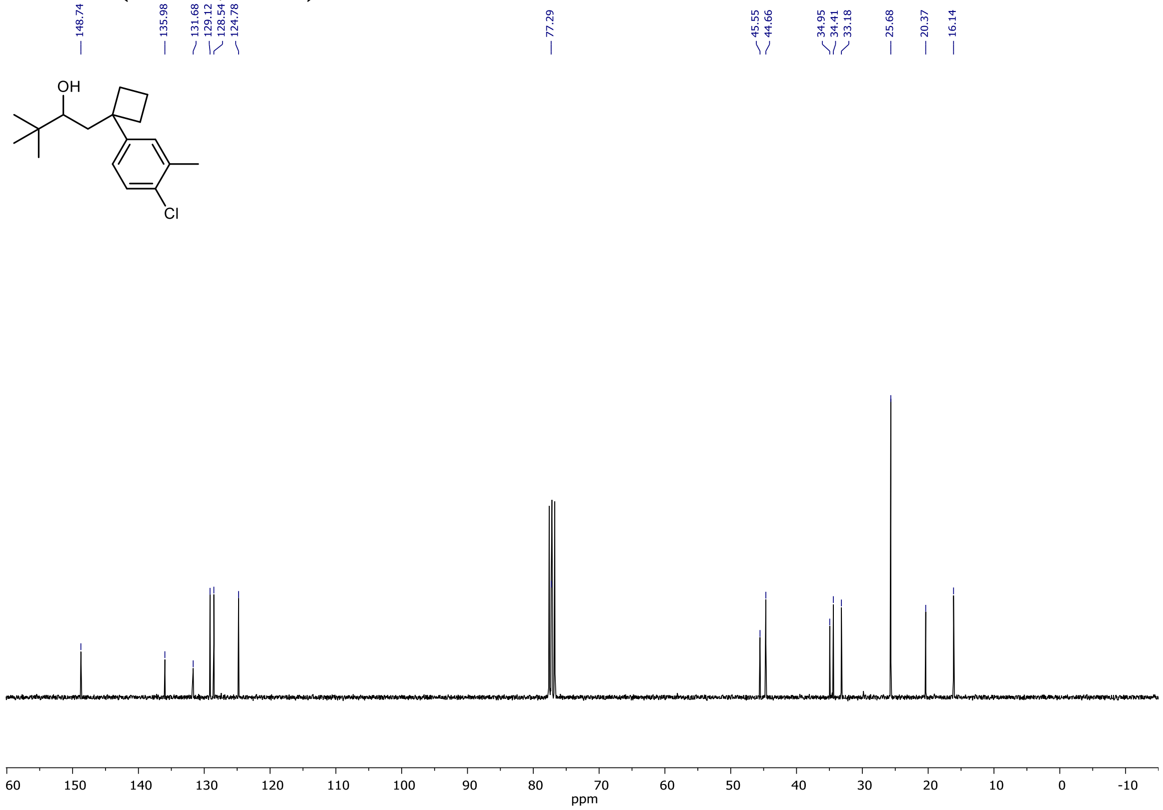

Supplementary Figure 106: NMR Spectra of 1-(1-(4-Chloro-3-methylphenyl)cyclobutyl)-3,3-dimethylbutan-2-ol (3i-2)

^{1}H NMR (300 MHz, $CDCl_3$)

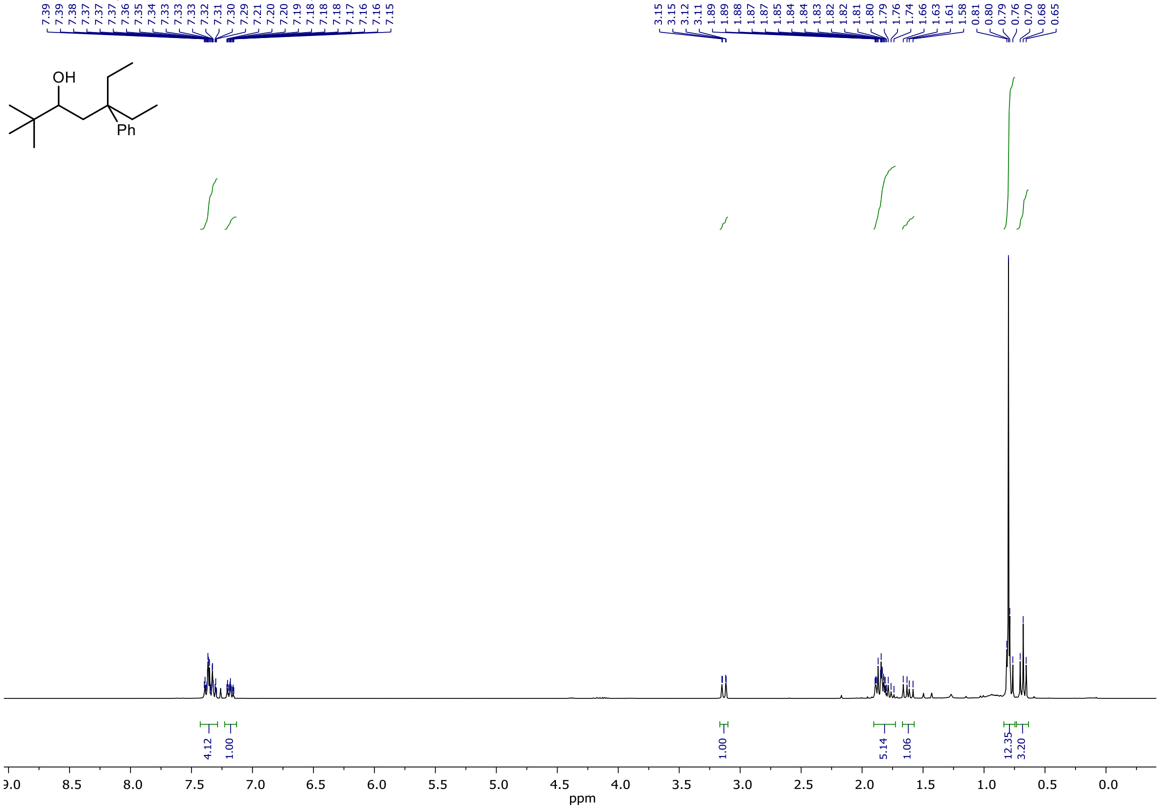

^{13}C NMR (75 MHz, $CDCl_3$)

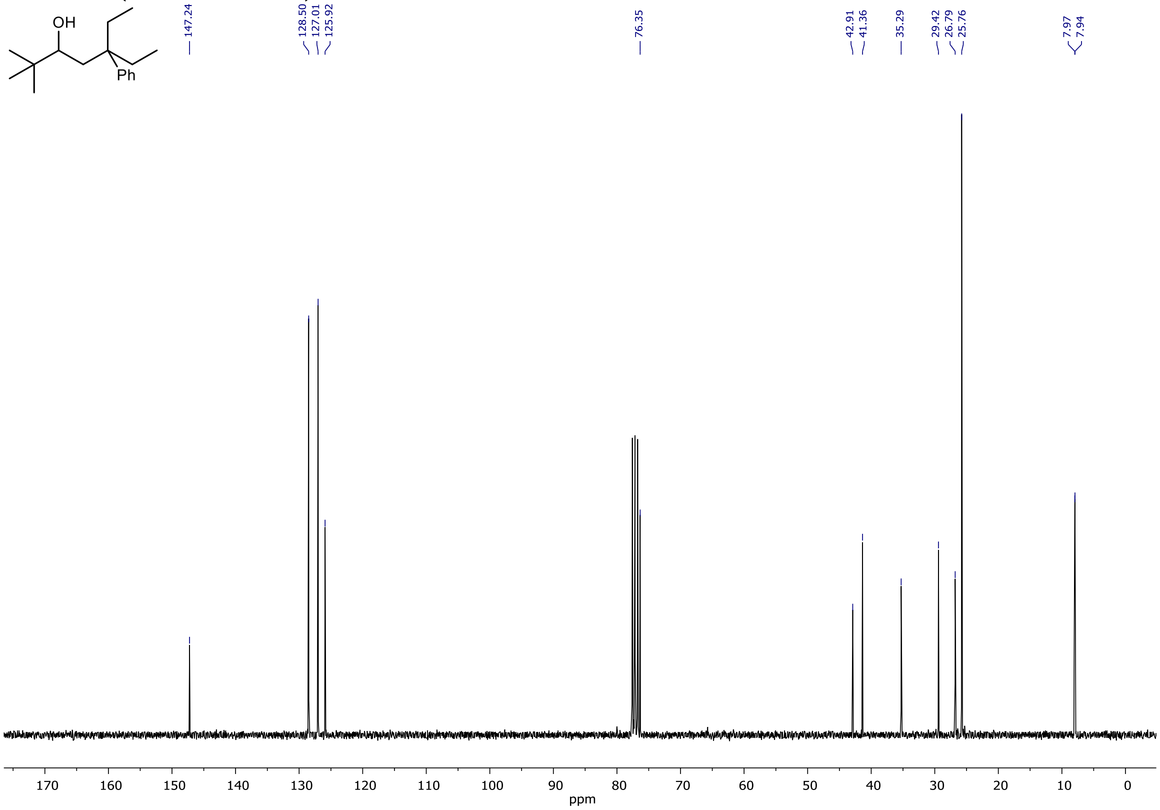

Supplementary Figure 107: NMR Spectra of 5-Ethyl-2,2-dimethyl-5-phenylheptan-3-ol (3j)

^{1}H NMR (400 MHz, CDCl$_3$)

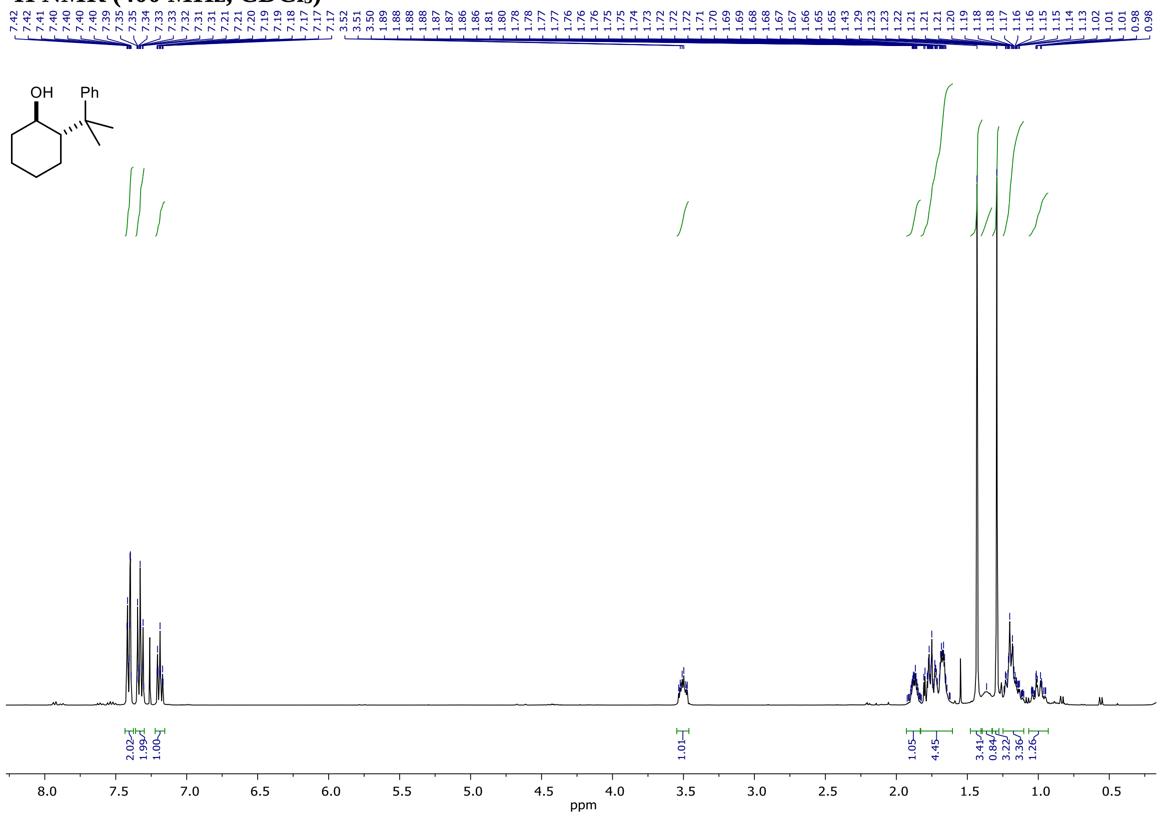

^{13}C NMR (101 MHz, CDCl$_3$)

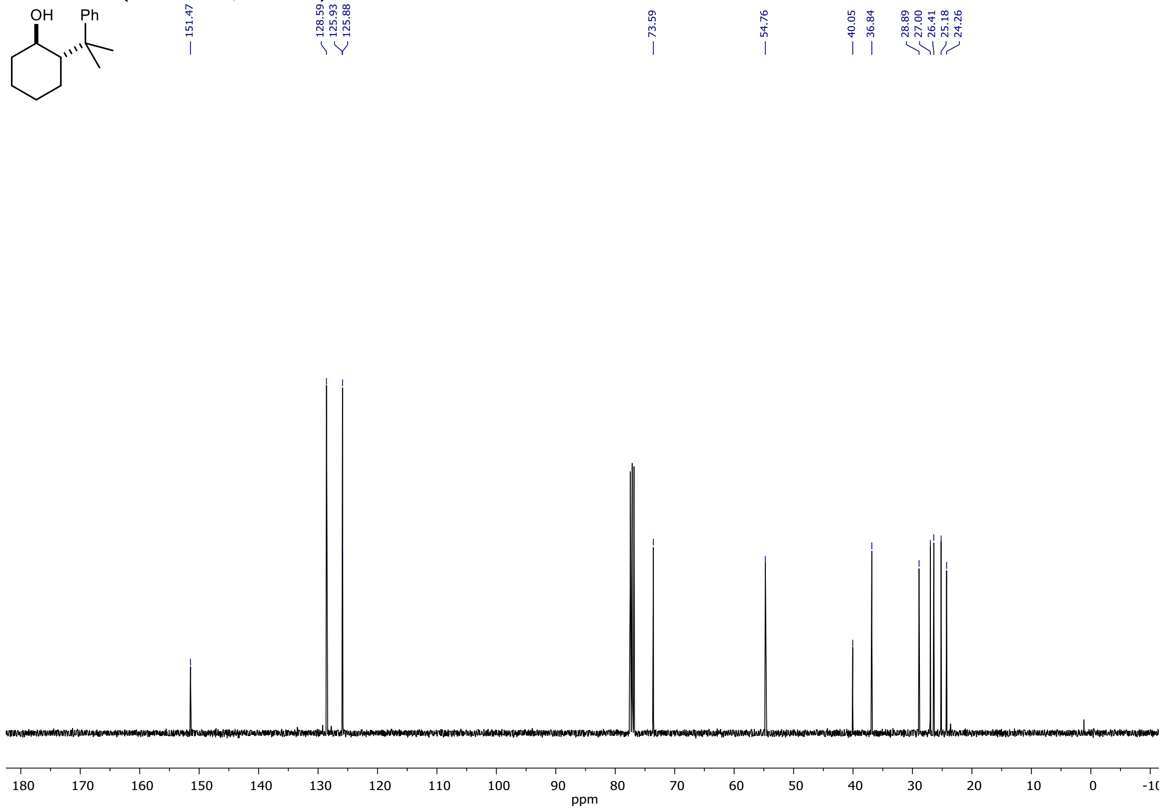

Supplementary Figure 108: NMR Spectra of *trans*-2-(2-Phenylpropan-2-yl)cyclohexan-1-ol (3k)

^{1}H NMR (300 MHz, $CDCl_3$)

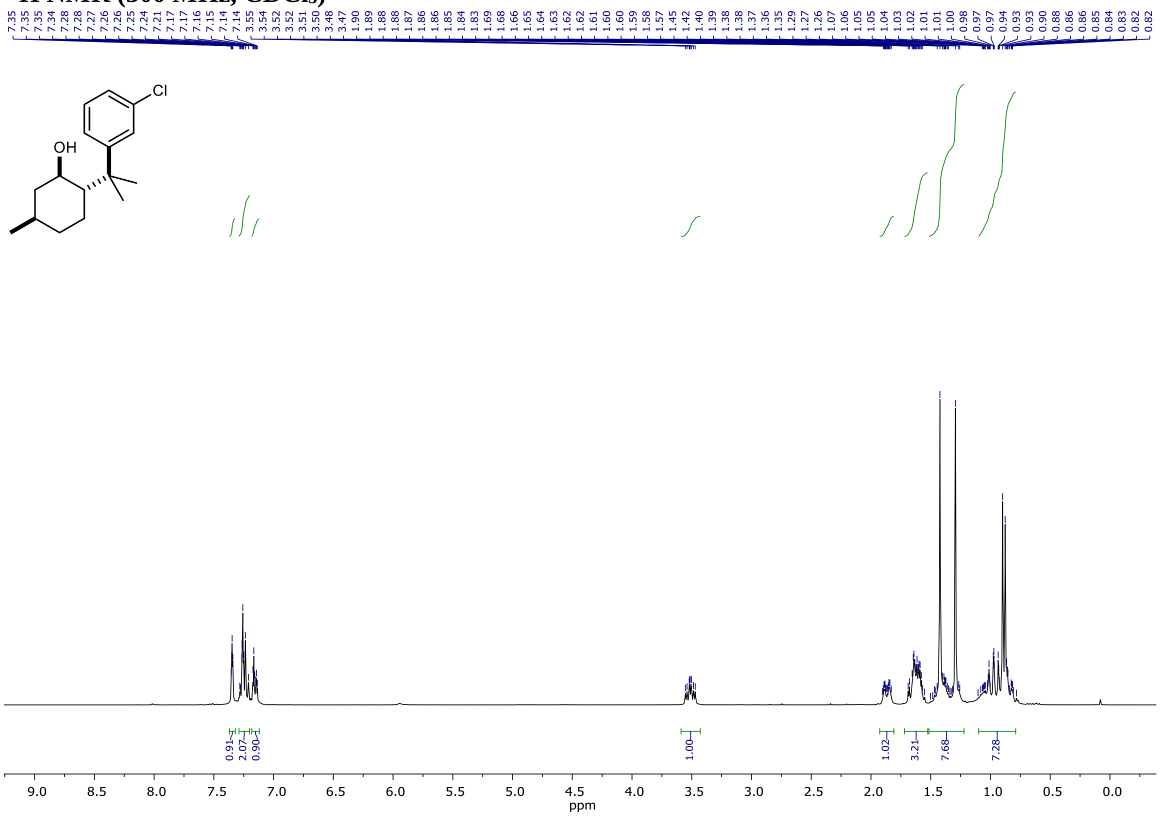

^{13}C NMR (75 MHz, $CDCl_3$)

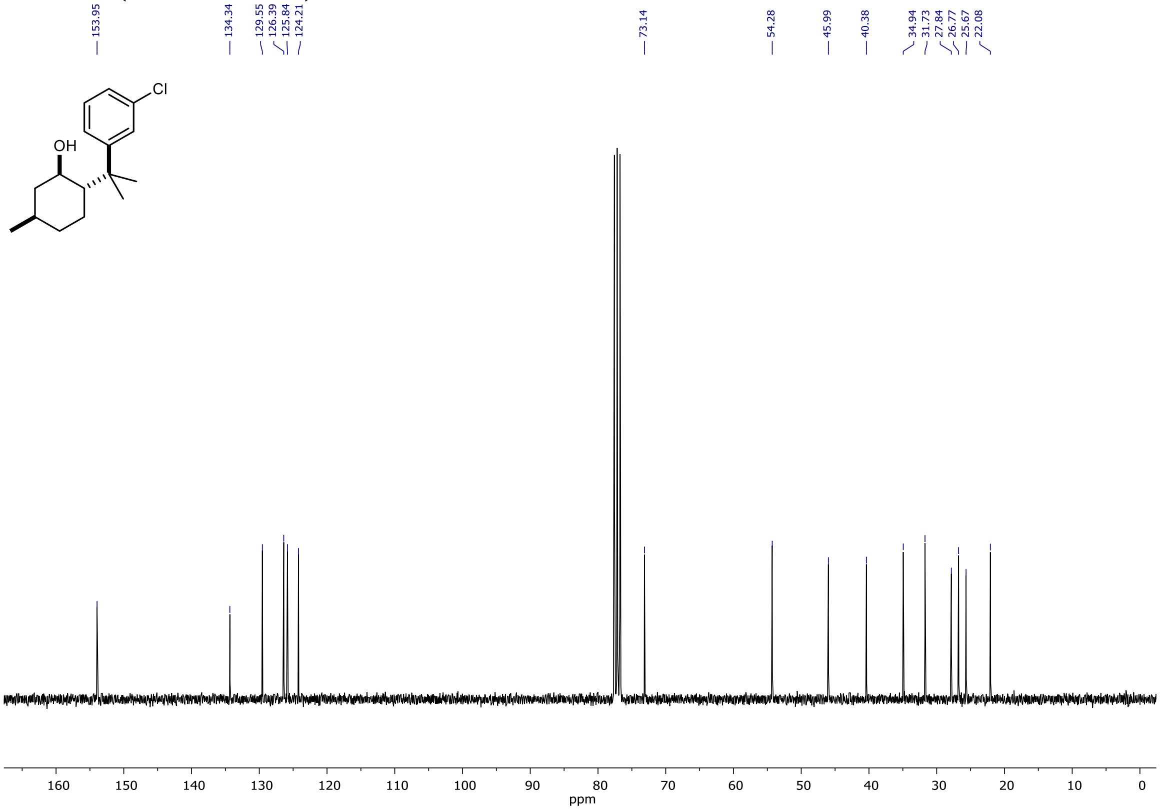

Supplementary Figure 109: NMR Spectra of (1*R*,2*S*,5*R*)-2-(2-(3-Chlorophenyl)propan-2-yl)-5-methylcyclohexan-1-ol (3l-3)

^{1}H NMR (300 MHz, $CDCl_3$)

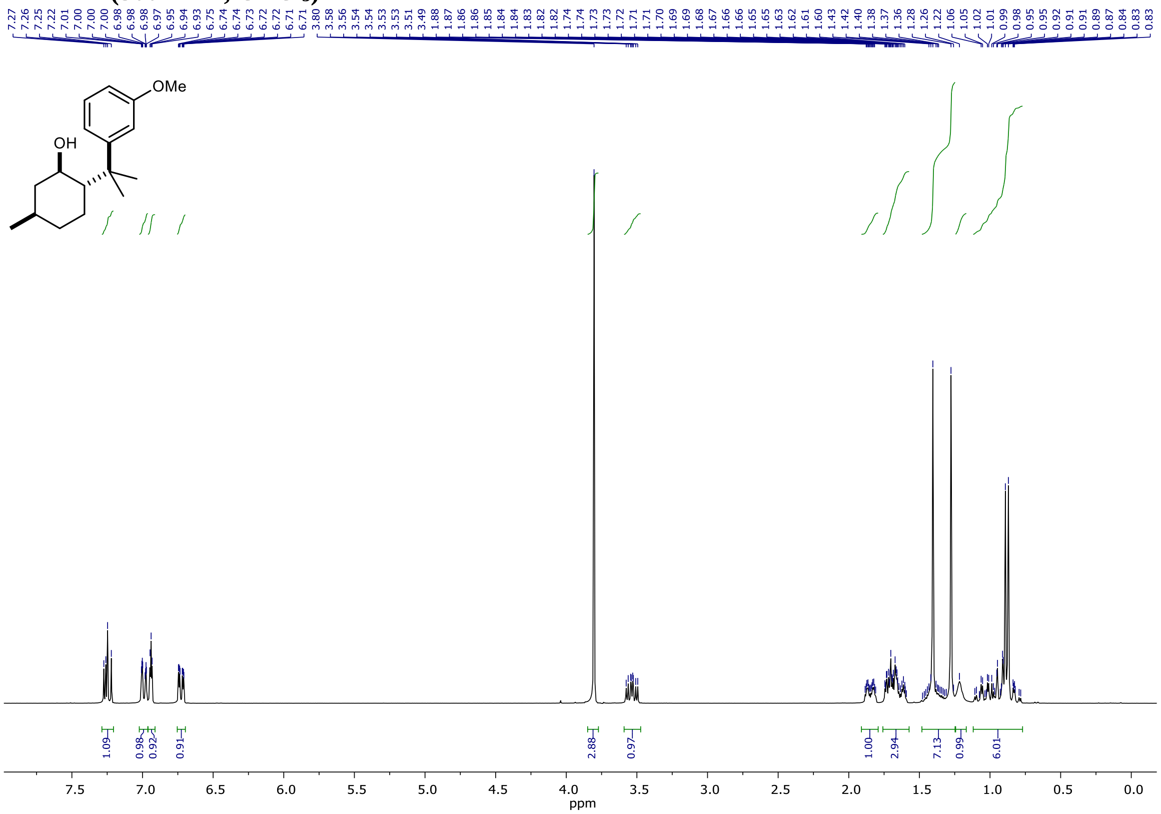

^{13}C NMR (75 MHz, $CDCl_3$)

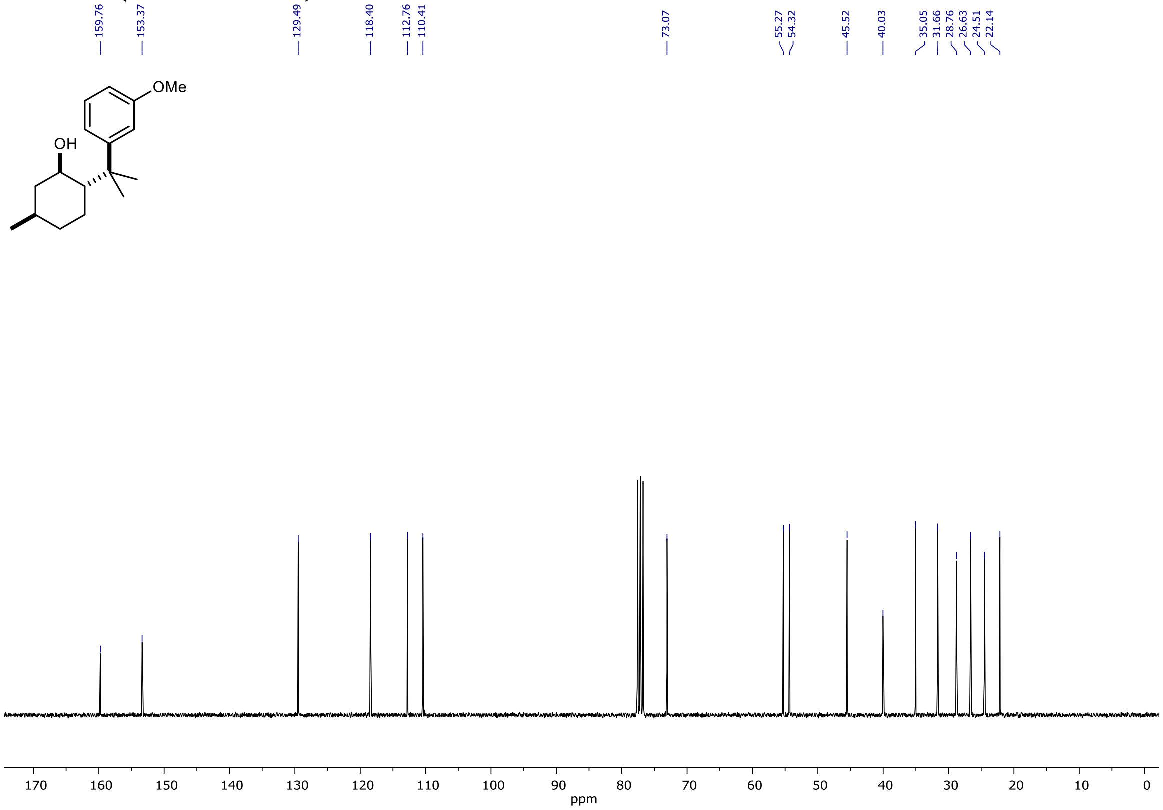

Supplementary Figure 110: NMR Spectra of (1*R*,2*S*,5*R*)-2-(2-(3-Methoxyphenyl)propan-2-yl)-5-methylcyclohexan-1-ol (3l-4)

^{1}H NMR (300 MHz, $CDCl_3$)

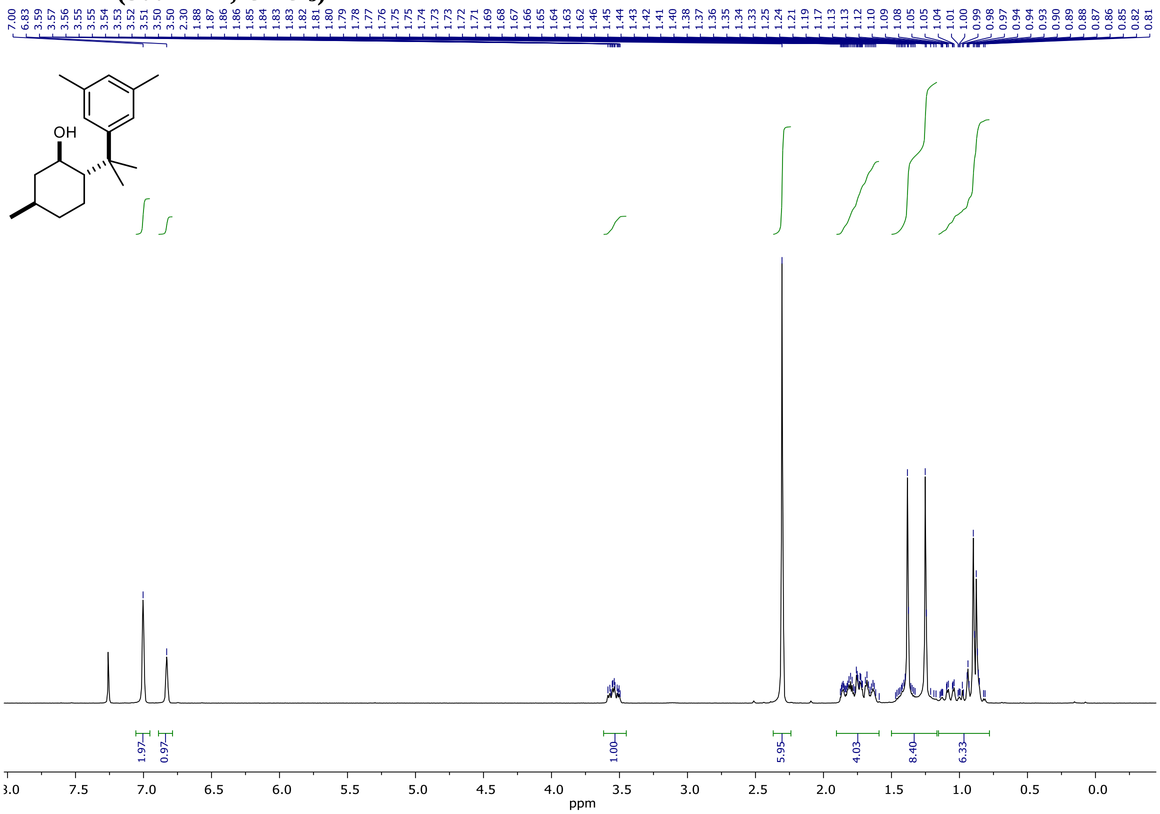

^{13}C NMR (75 MHz, $CDCl_3$)

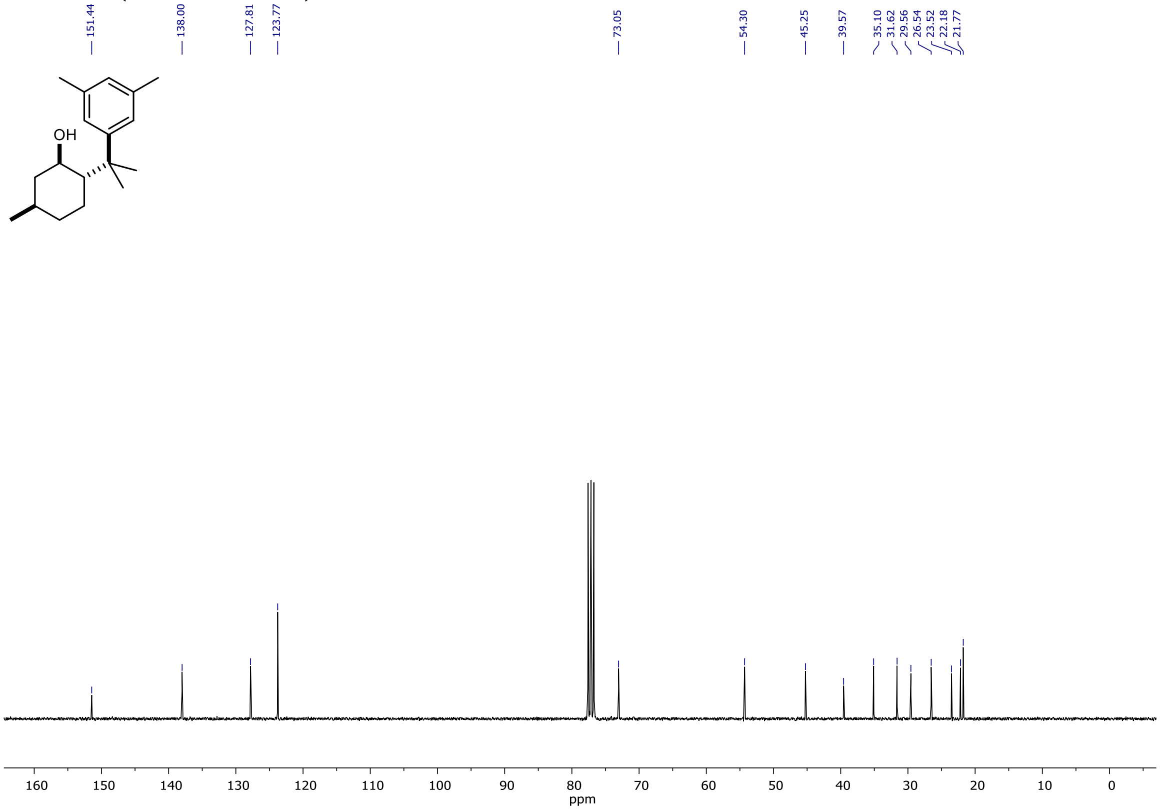

Supplementary Figure 111: NMR Spectra of (1*R*,2*S*,5*R*)-2-(2-(3,5-Dimethylphenyl)propan-2-yl)-5-methylcyclohexan-1-ol (3l-5)

^{1}H NMR (400 MHz, $CDCl_3$)

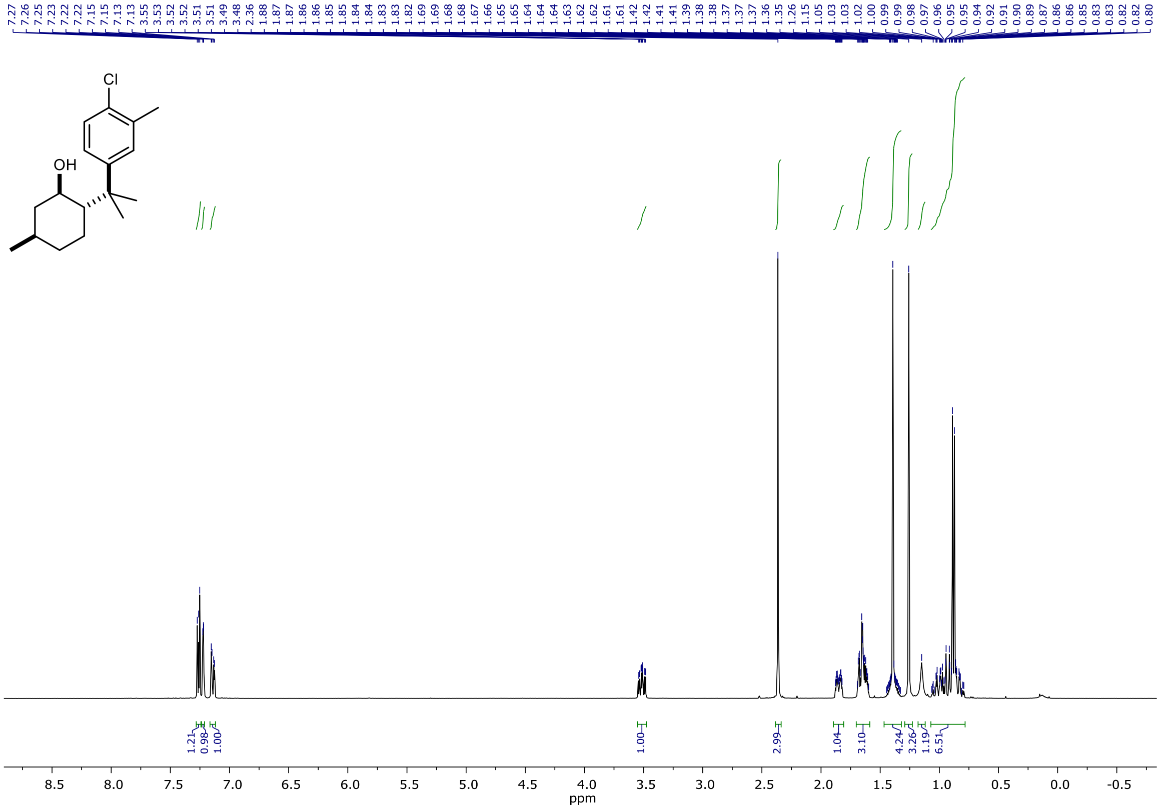

^{13}C NMR (101 MHz, $CDCl_3$)

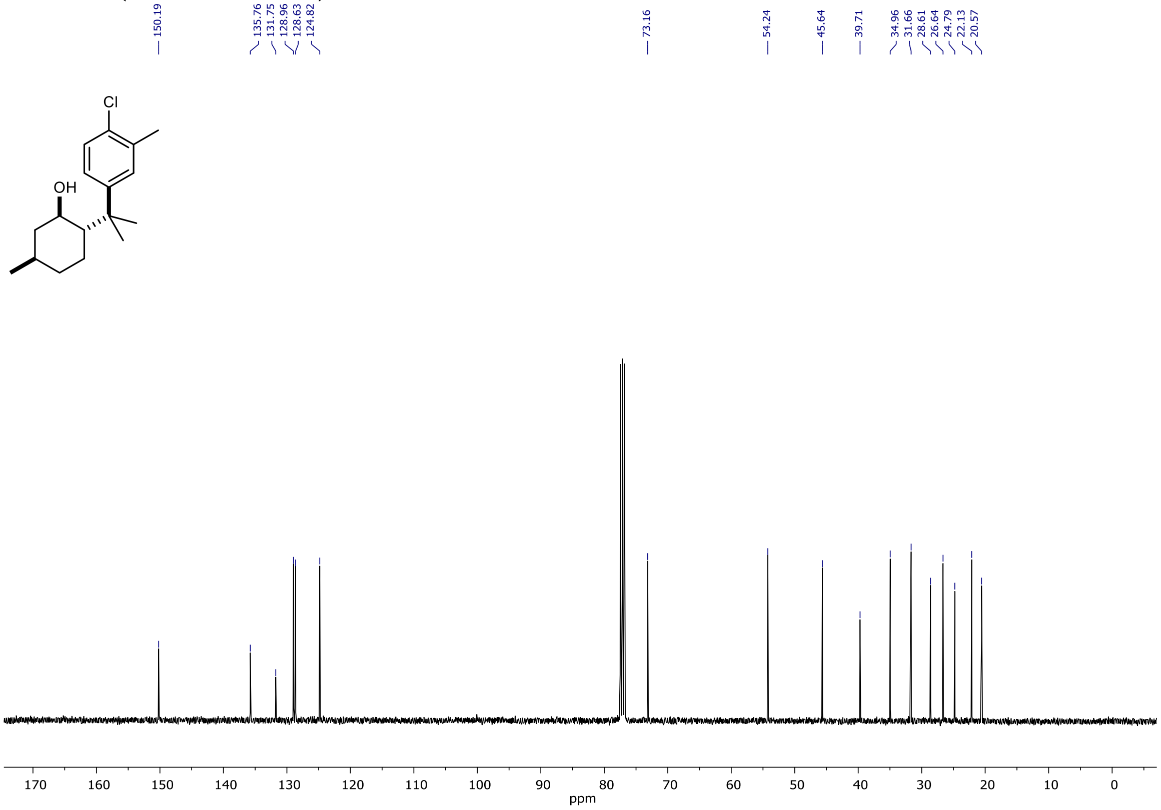

Supplementary Figure 112: NMR Spectra of (1*R*,2*S*,5*R*)-2-(2-(4-Chloro-3-methylphenyl)propan-2-yl)-5-methylcyclohexan-1-ol (3l-7)

^{1}H NMR (300 MHz, CDCl$_3$)

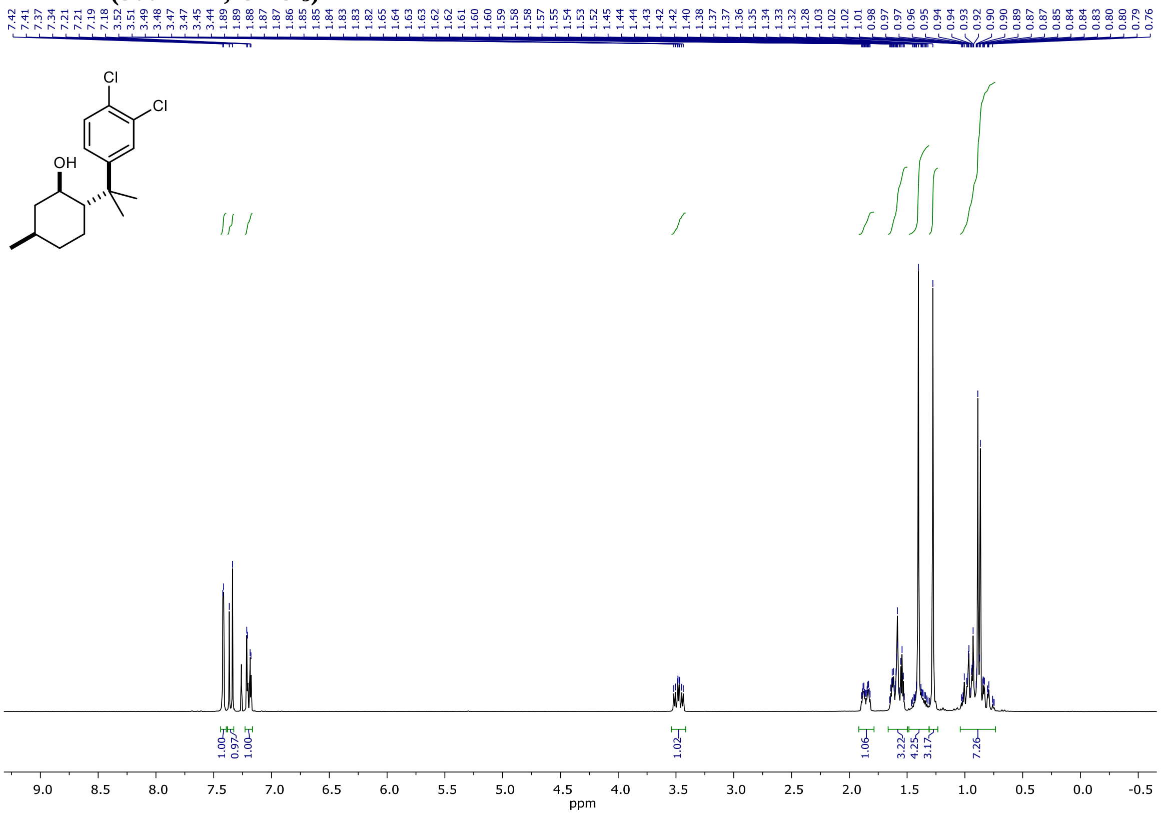

^{13}C NMR (75 MHz, CDCl$_3$)

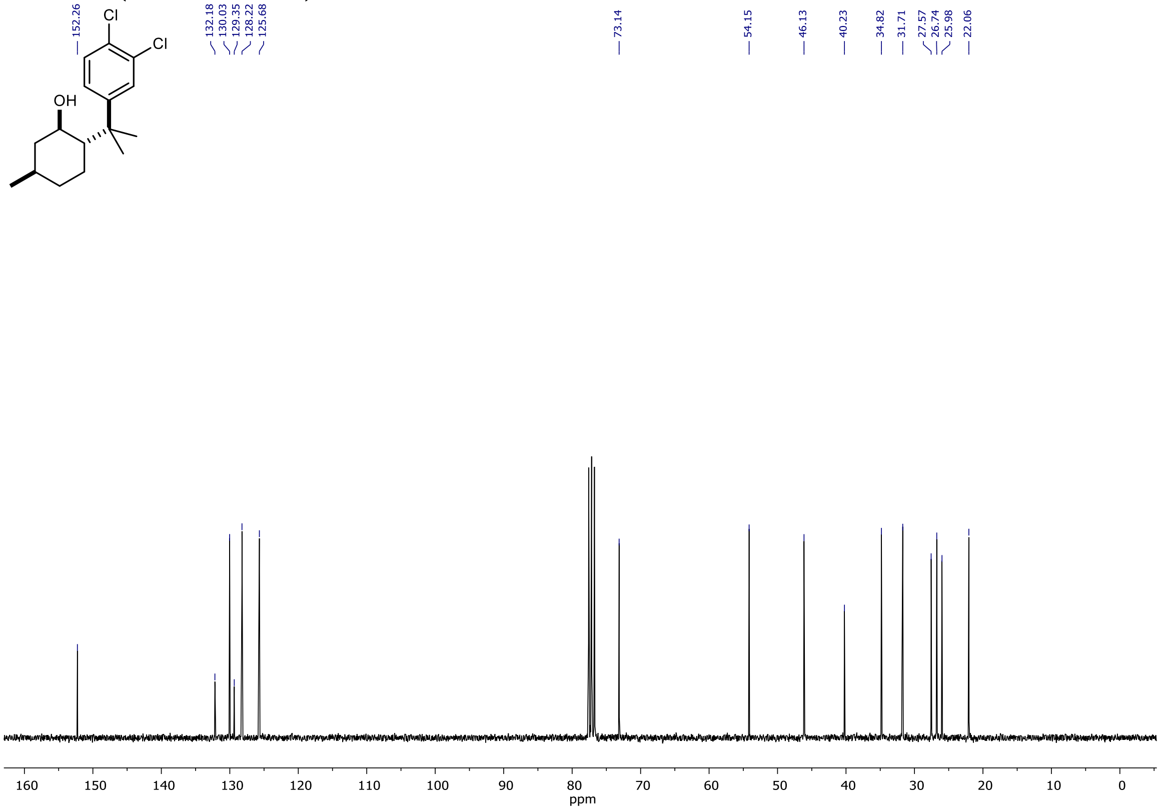

Supplementary Figure 113: NMR Spectra of (1*R*,2*S*,5*R*)-2-(2-(3,4-Dichlorophenyl)propan-2-yl)-5-methylcyclohexan-1-ol (3l-8)

^{1}H NMR (300 MHz, $CDCl_3$)

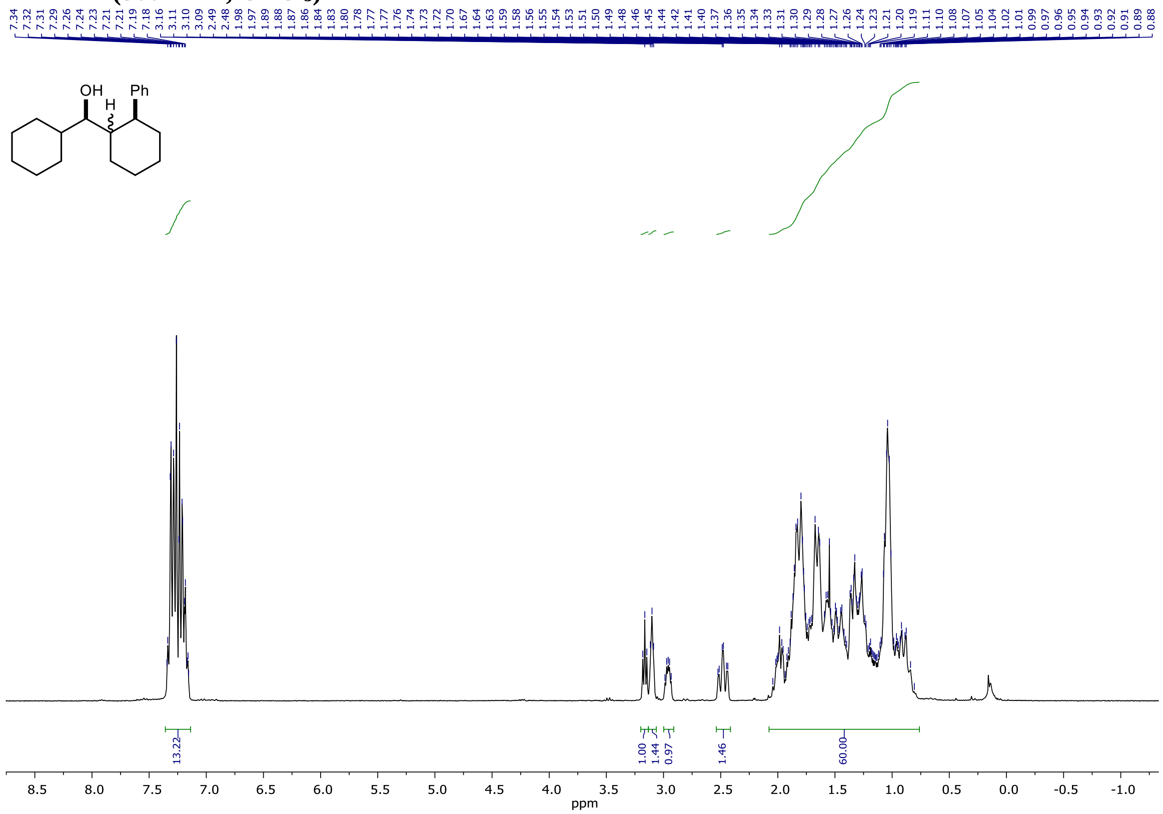

^{13}C NMR (151 MHz, $CDCl_3$)

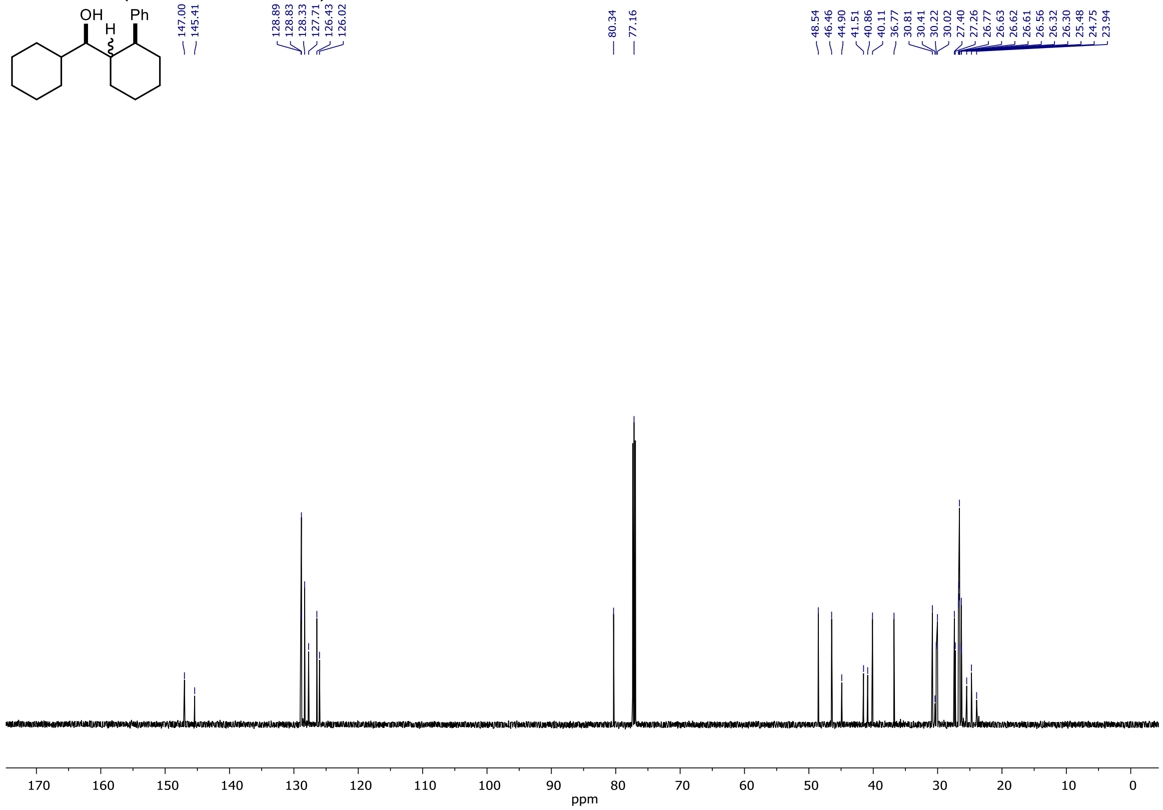

Supplementary Figure 114: NMR Spectra of Cyclohexyl(2-phenylcyclohexyl)methanol (3m)

^{1}H NMR (300 MHz, $CDCl_3$)

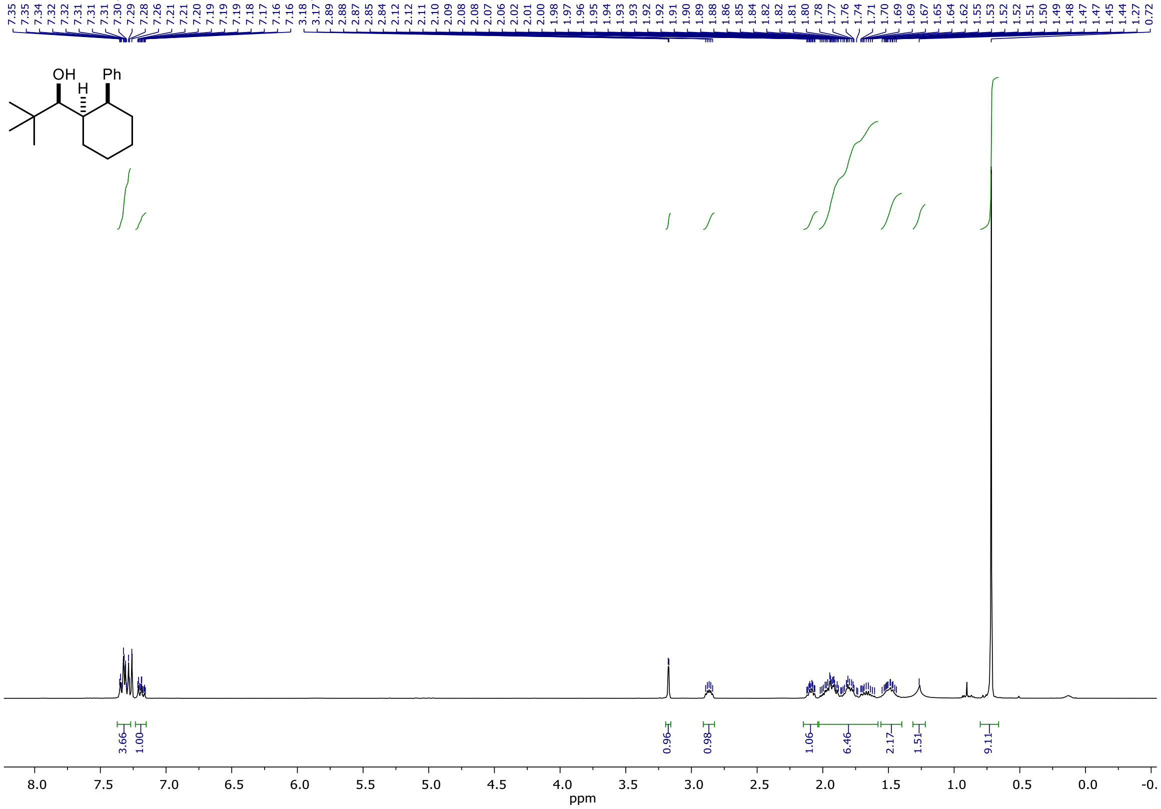

^{13}C NMR (75 MHz, $CDCl_3$)

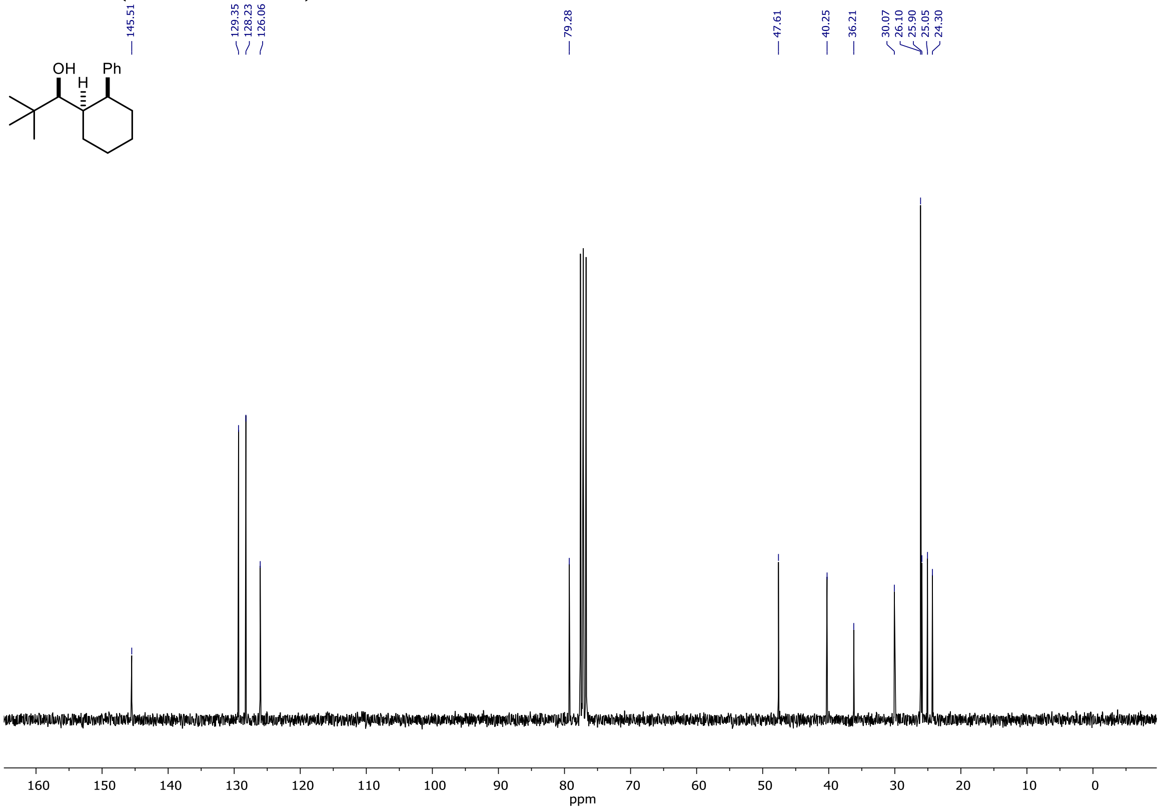

Supplementary Figure 115: NMR Spectra of 2,2-Dimethyl-1-(2-phenylcyclohexyl)propan-1-ol (3n-a)

^{1}H NMR (300 MHz, CDCl$_3$)

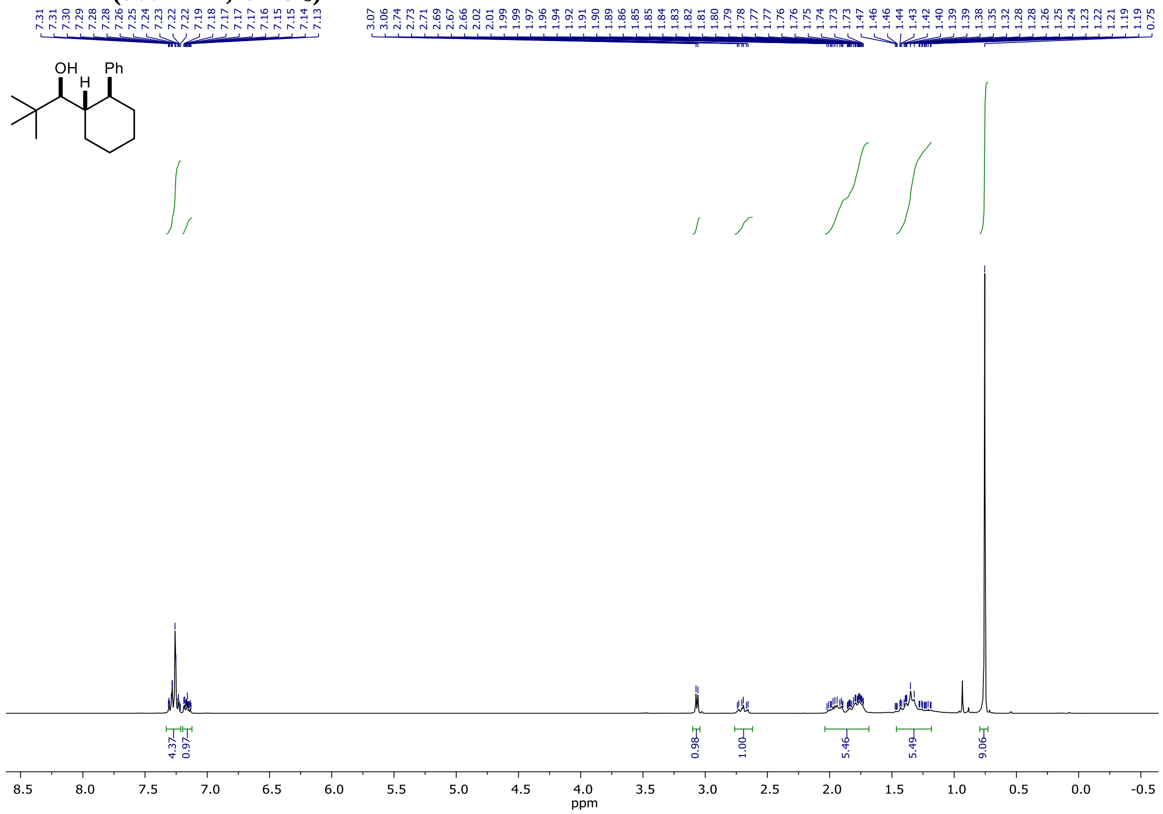

^{13}C NMR (75 MHz, CDCl$_3$)

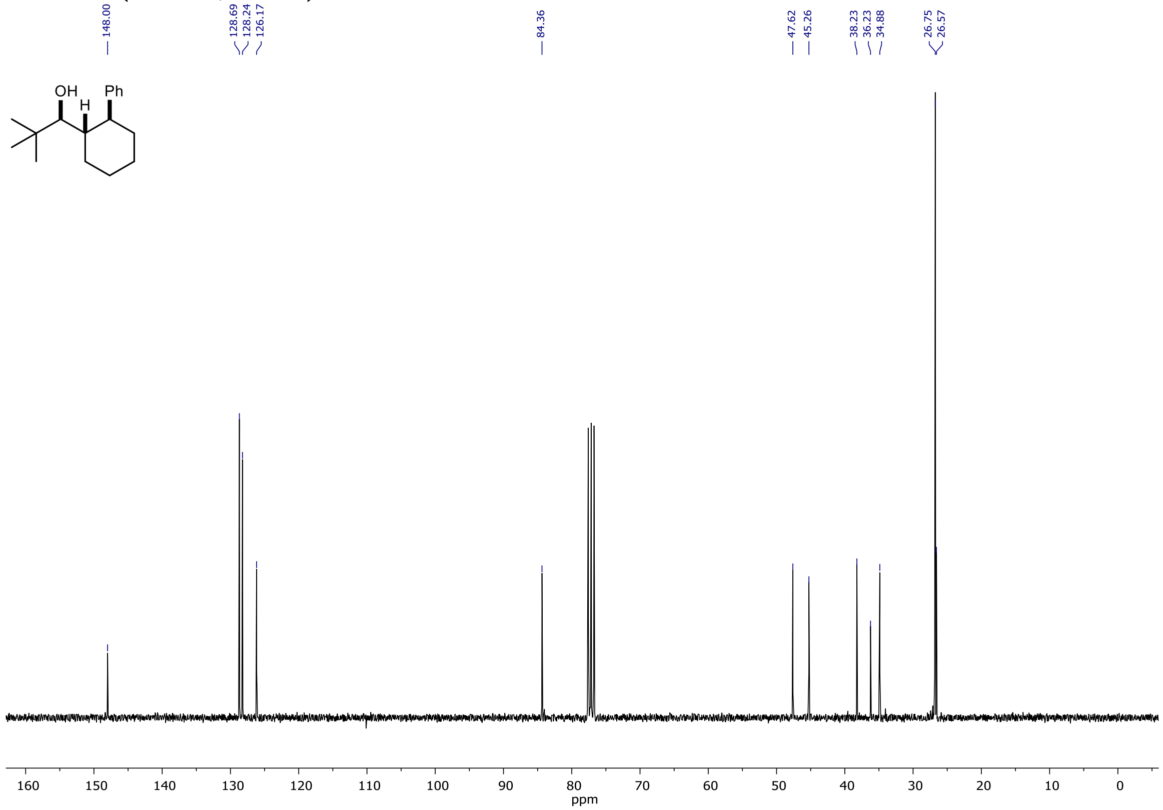

Supplementary Figure 116: NMR Spectra of 2,2-Dimethyl-1-(2-phenylcyclohexyl)propan-1-ol (3n-b)

^{1}H NMR (300 MHz, $CDCl_3$)

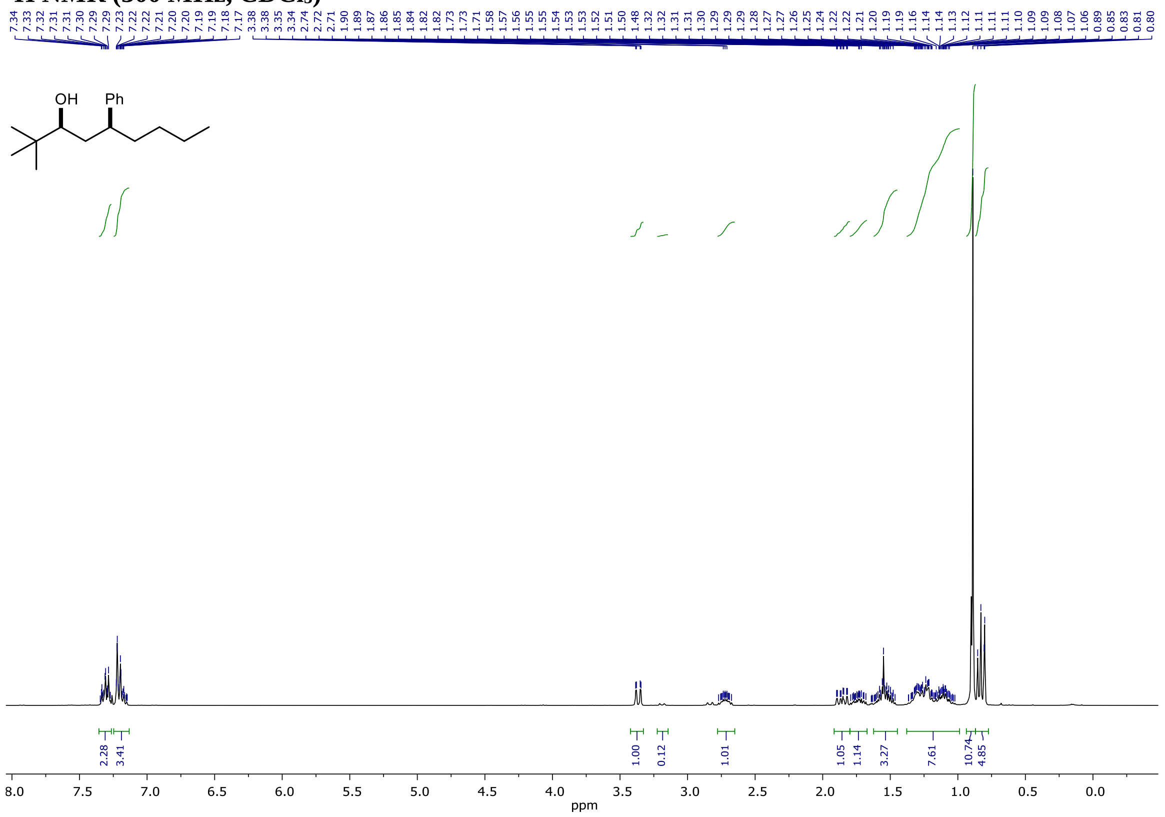

^{13}C NMR (75 MHz, $CDCl_3$)

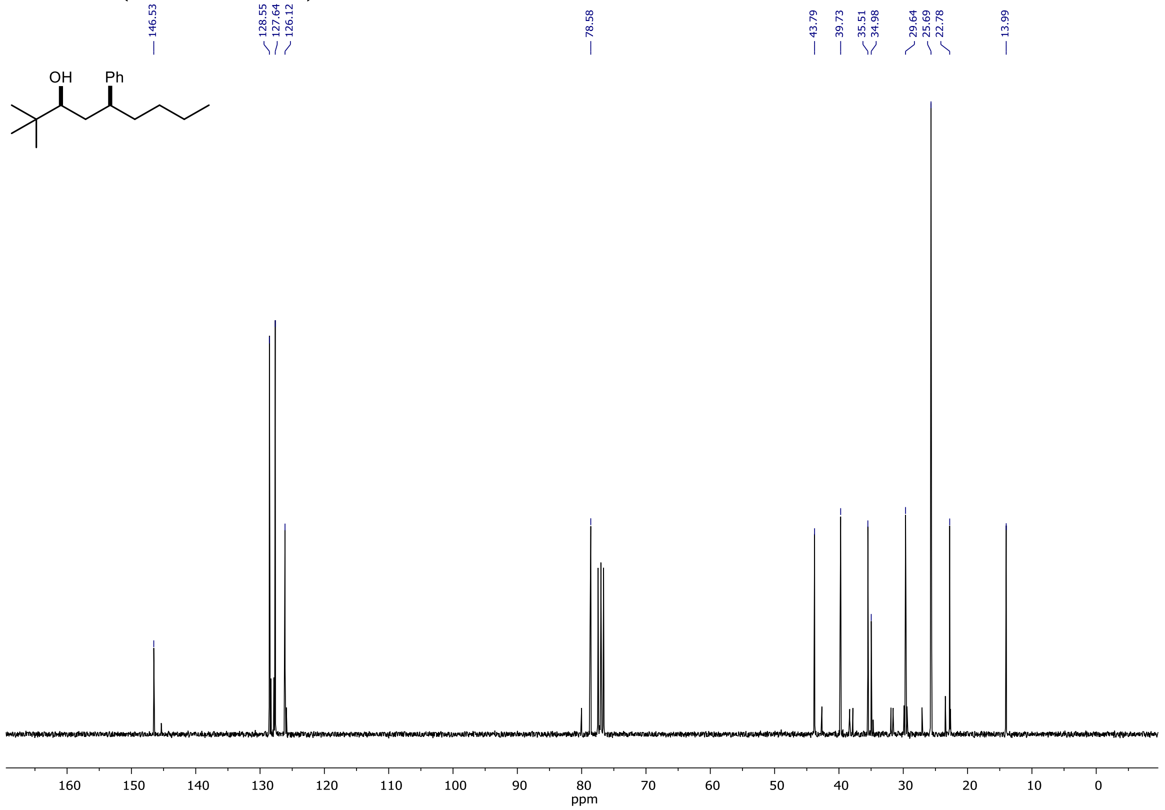

Supplementary Figure 117: NMR Spectra of 2,2-Dimethyl-5-phenylnonan-3-ol (3o)

^{1}H NMR (400 MHz, CDCl$_3$)

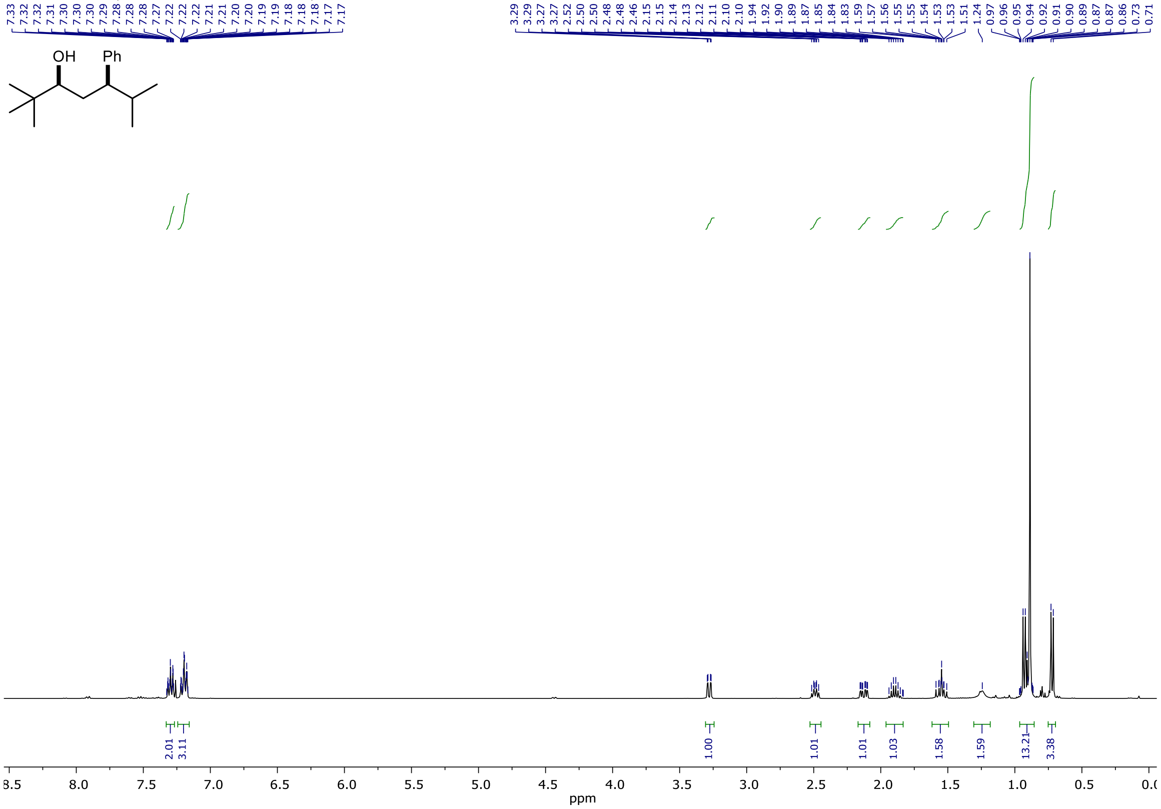

^{13}C NMR (101 MHz, CDCl$_3$)

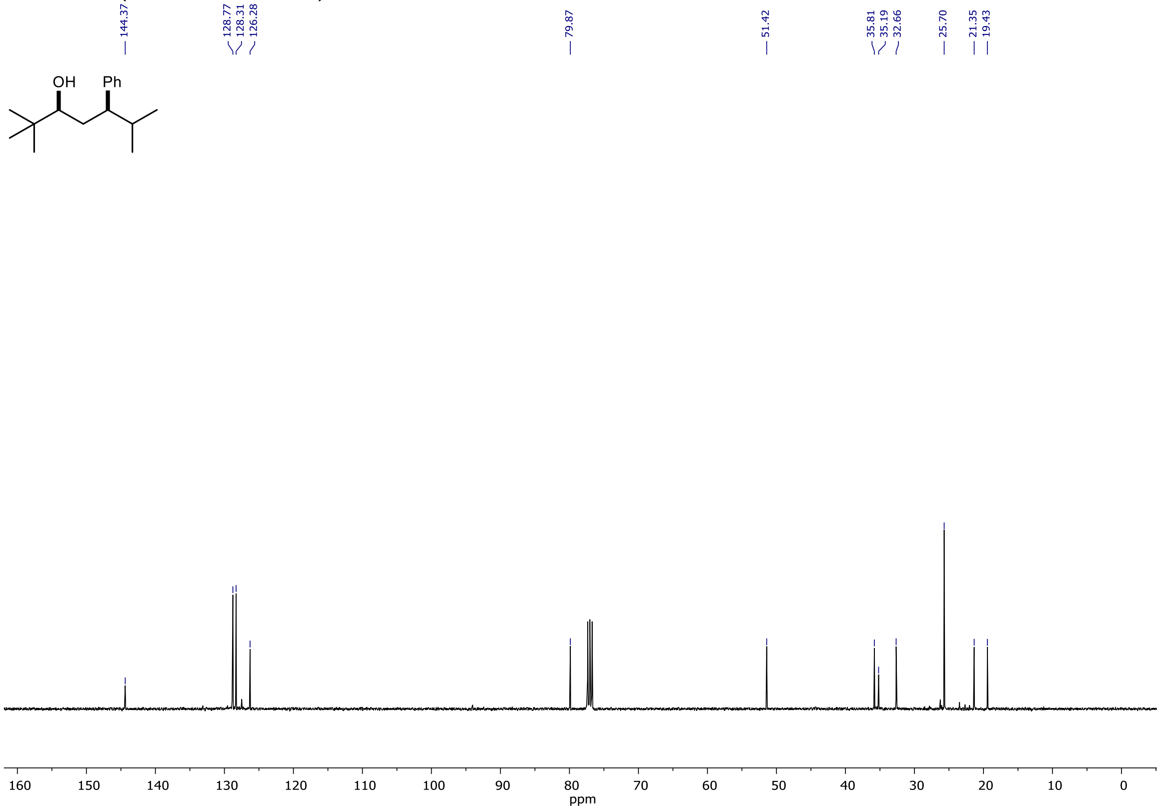

Supplementary Figure 118: NMR Spectra of 2,2,6-Trimethyl-5-phenylheptan-3-ol (3p)

^{1}H NMR (300 MHz, CDCl$_3$)

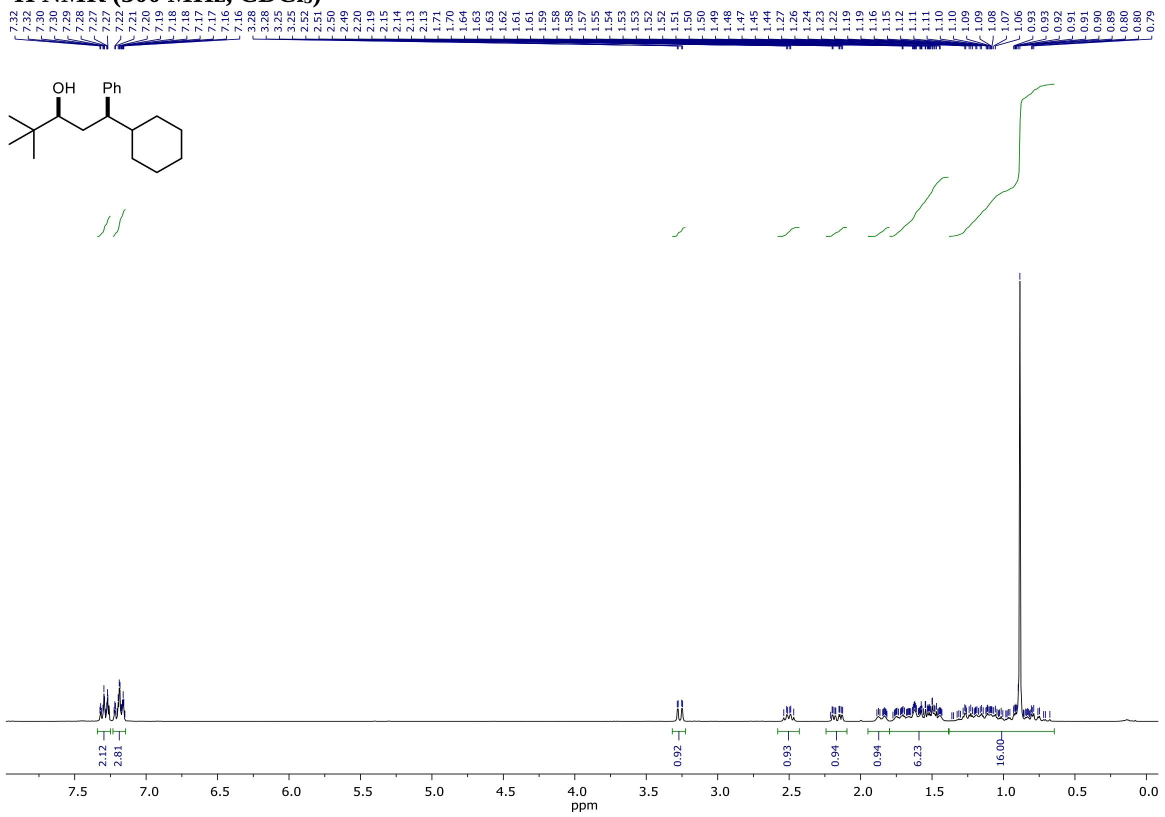

^{13}C NMR (75 MHz, CDCl$_3$)

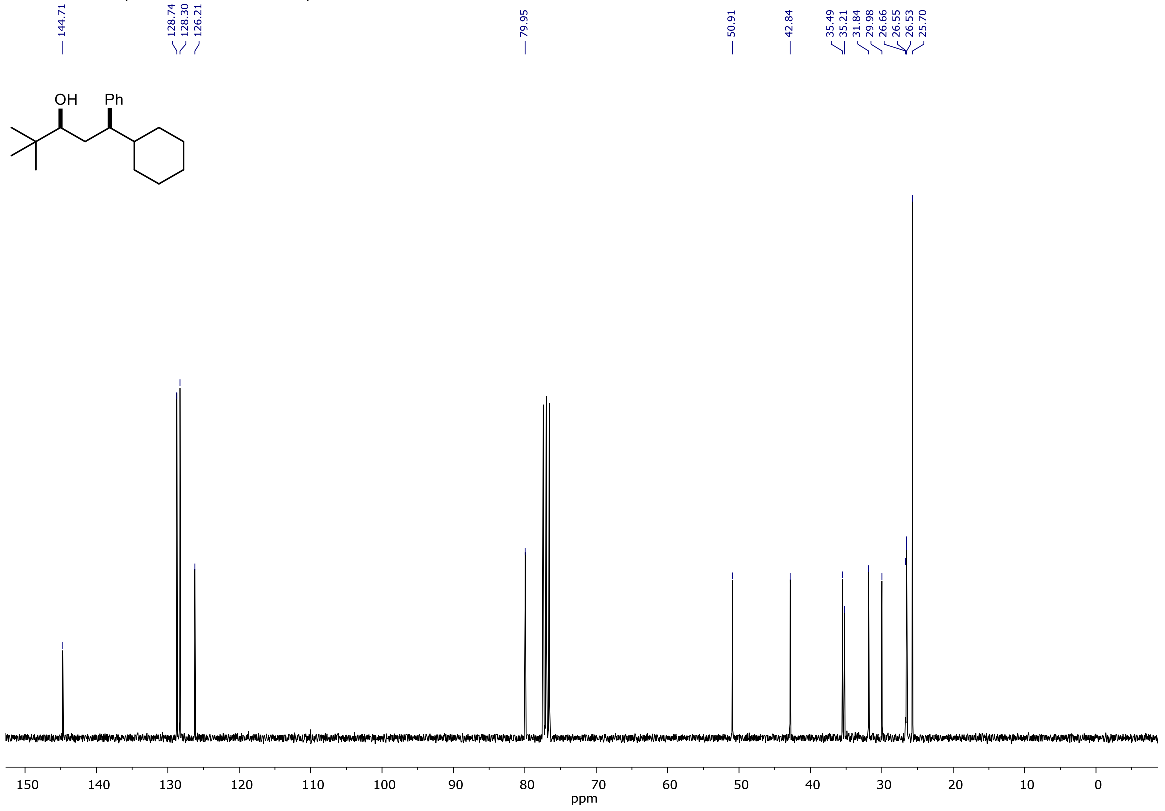

Supplementary Figure 119: NMR Spectra of 1-Cyclohexyl-4,4-dimethyl-1-phenylpentan-3-ol (3q)

^{1}H NMR (300 MHz, $CDCl_3$)

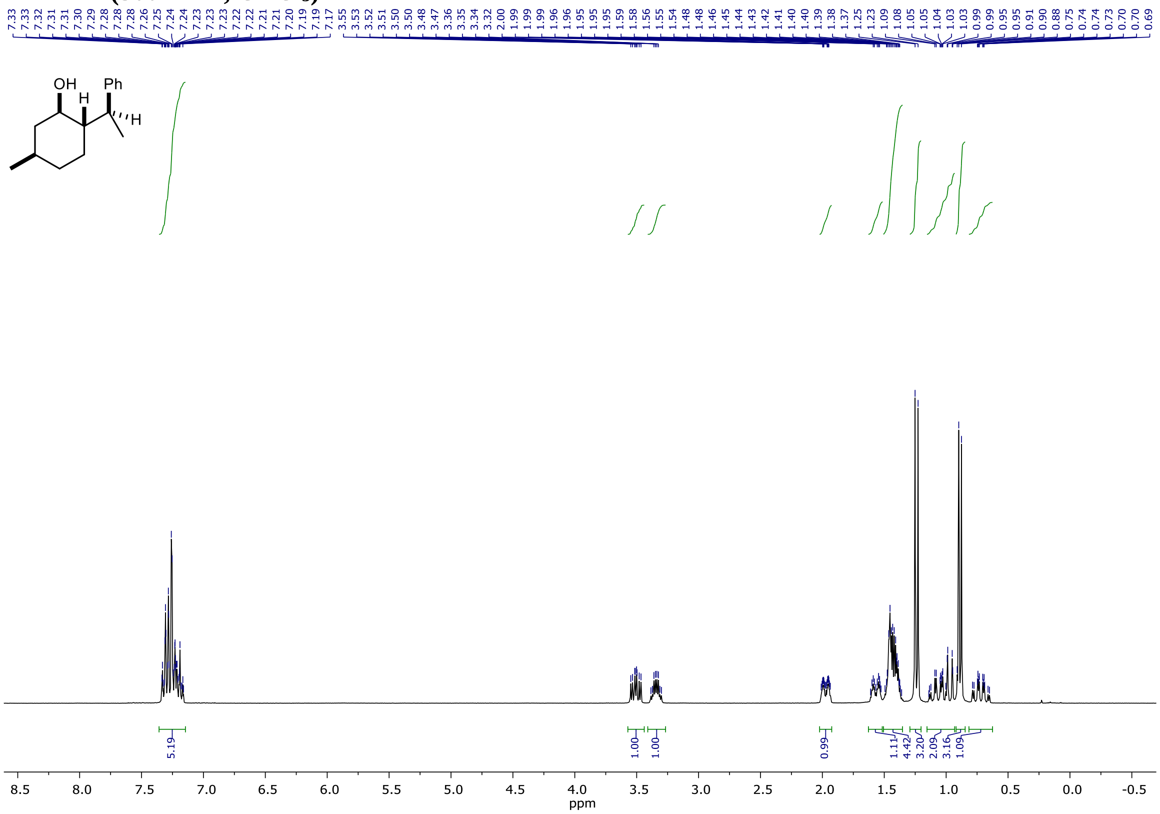

^{13}C NMR (75 MHz, $CDCl_3$)

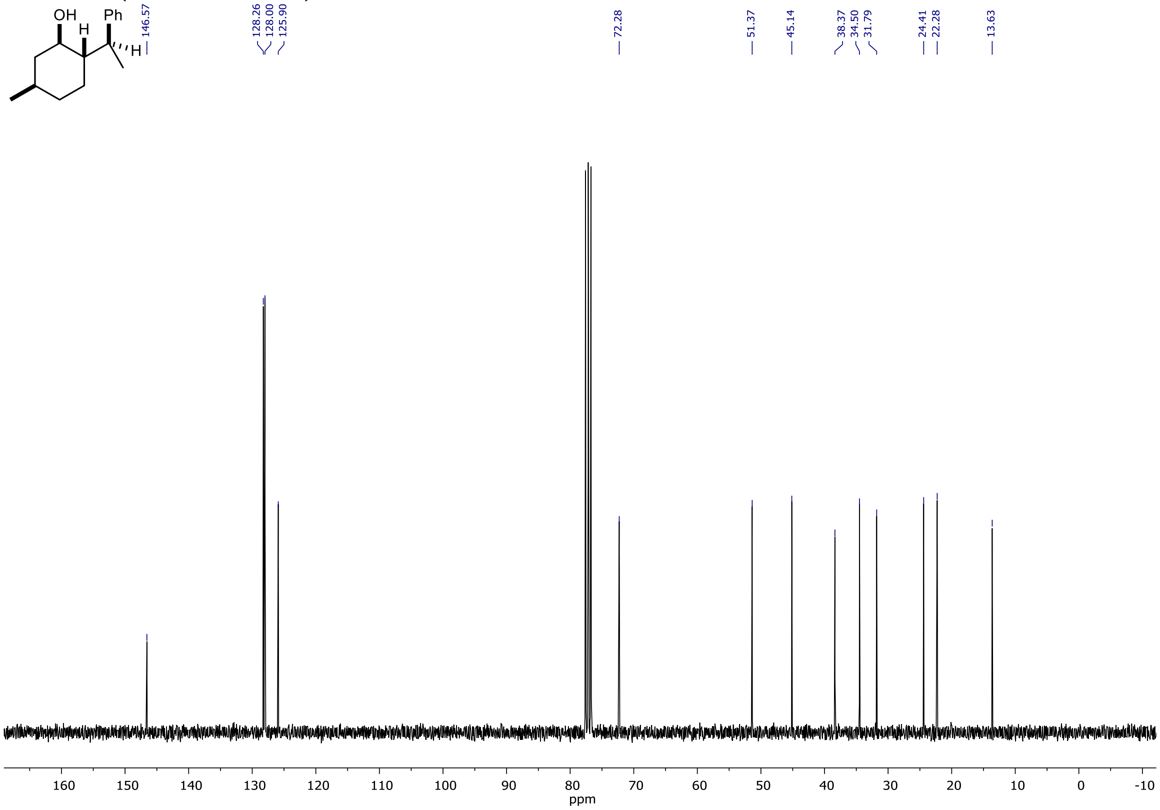

Supplementary Figure 120: NMR Spectra of (1*R*,2*S*,5*R*)-5-Methyl-2-((S)-1-phenylethyl)cyclohexan-1-ol (3r-1)

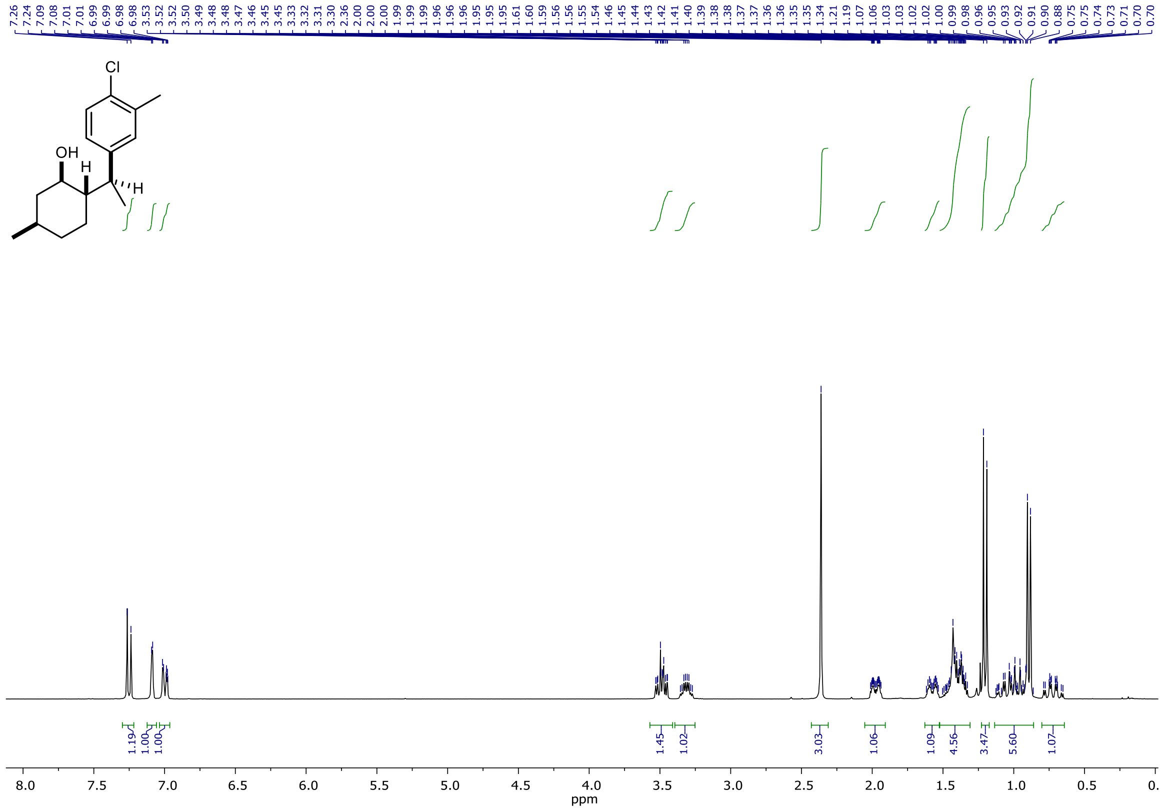

^{13}C NMR (75 MHz, $CDCl_3$)

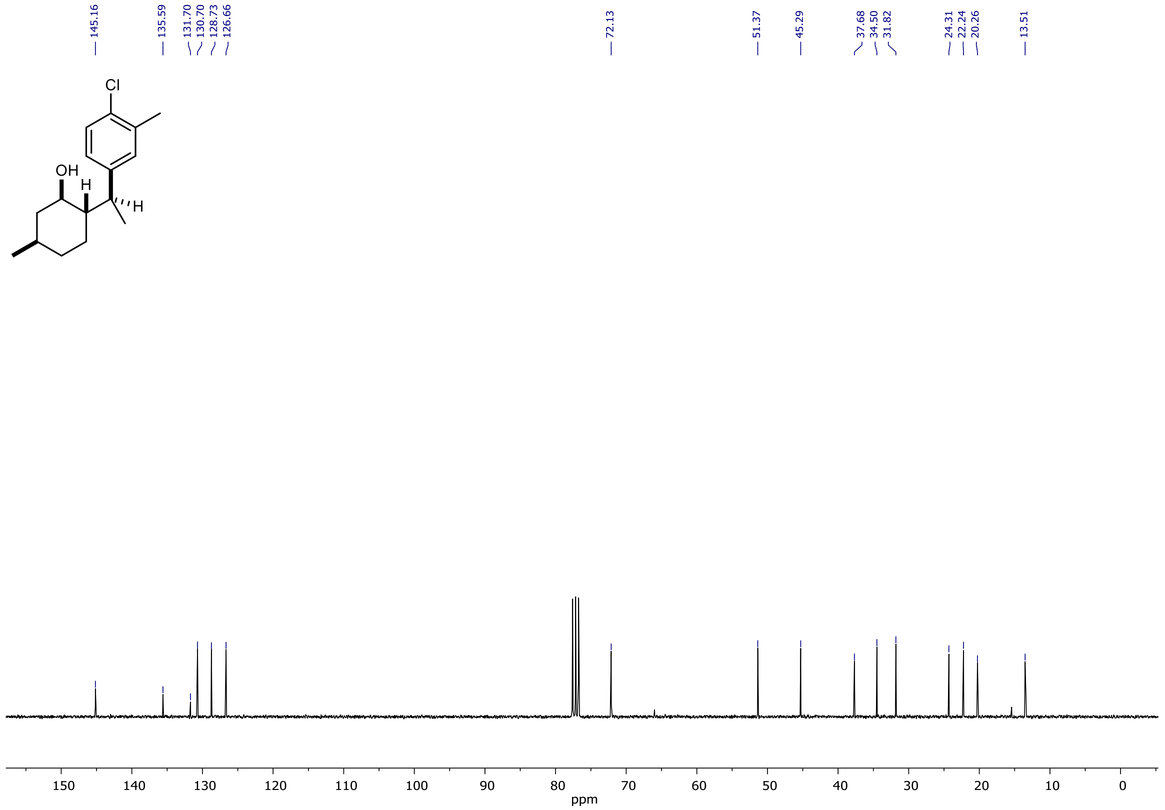

Supplementary Figure 121: NMR Spectra of (1*R*,2*S*,5*R*)-2-((S)-1-(4-Chloro-3-methylphenyl)ethyl)-5-methylcyclohexan-1-ol (3r-2)

^{1}H NMR (300 MHz, $CDCl_3$)

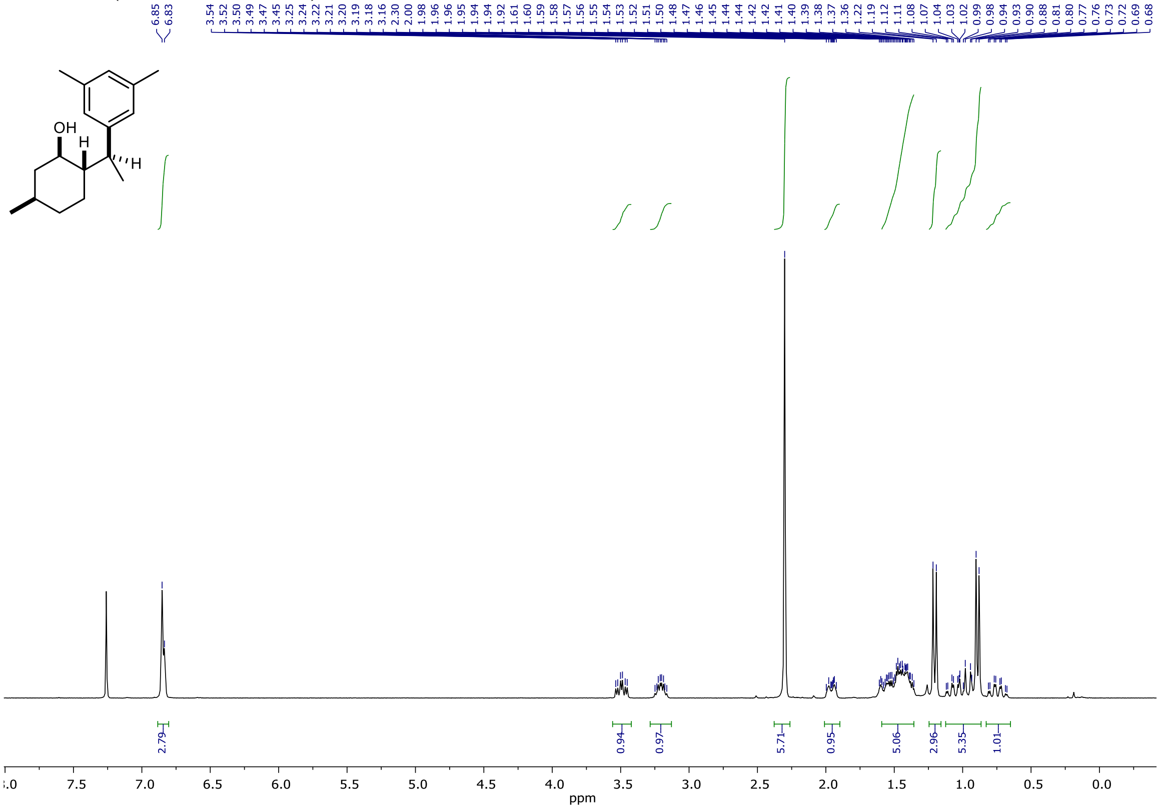

^{13}C NMR (75 MHz, $CDCl_3$)

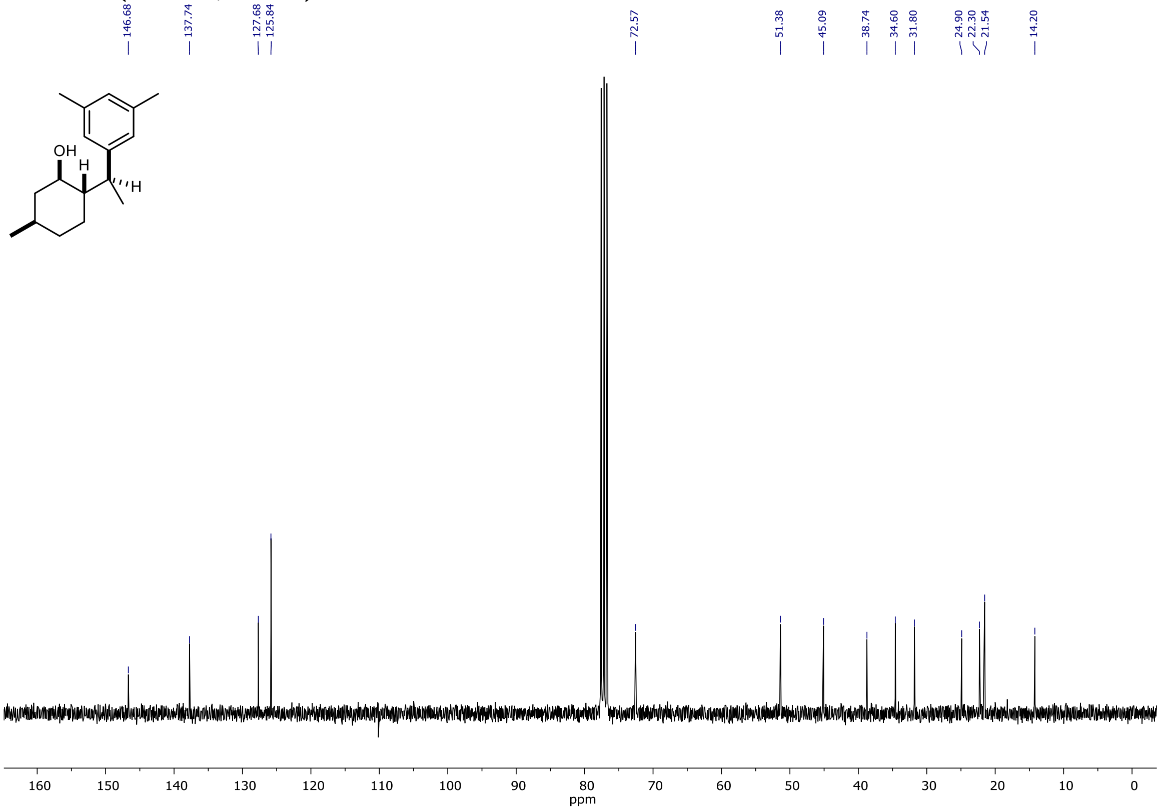

Supplementary Figure 122: NMR Spectra of (1*R*,2*S*,5*R*)-2-((S)-1-(3,5-Dimethylphenyl)ethyl)-5-methylcyclohexan-1-ol (3r-3)

^{1}H NMR (300 MHz, CDCl$_3$)

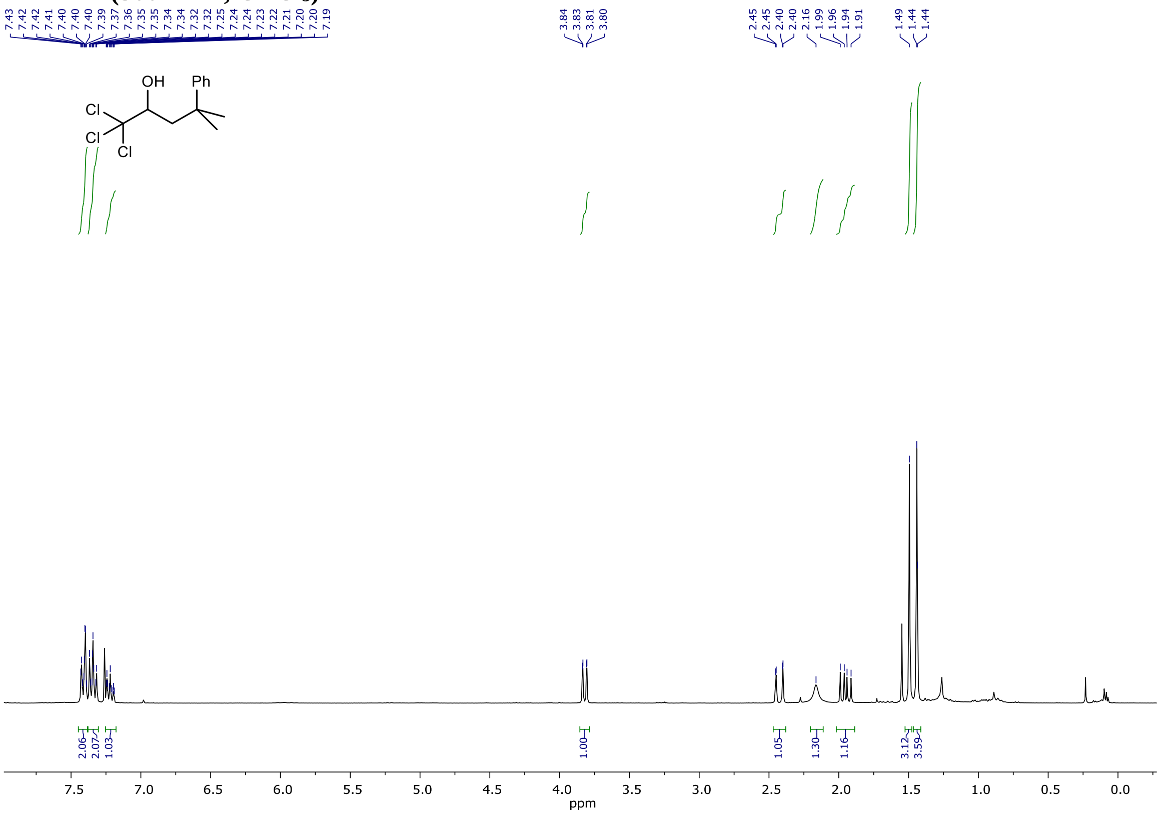

^{13}C NMR (101 MHz, CDCl$_3$)

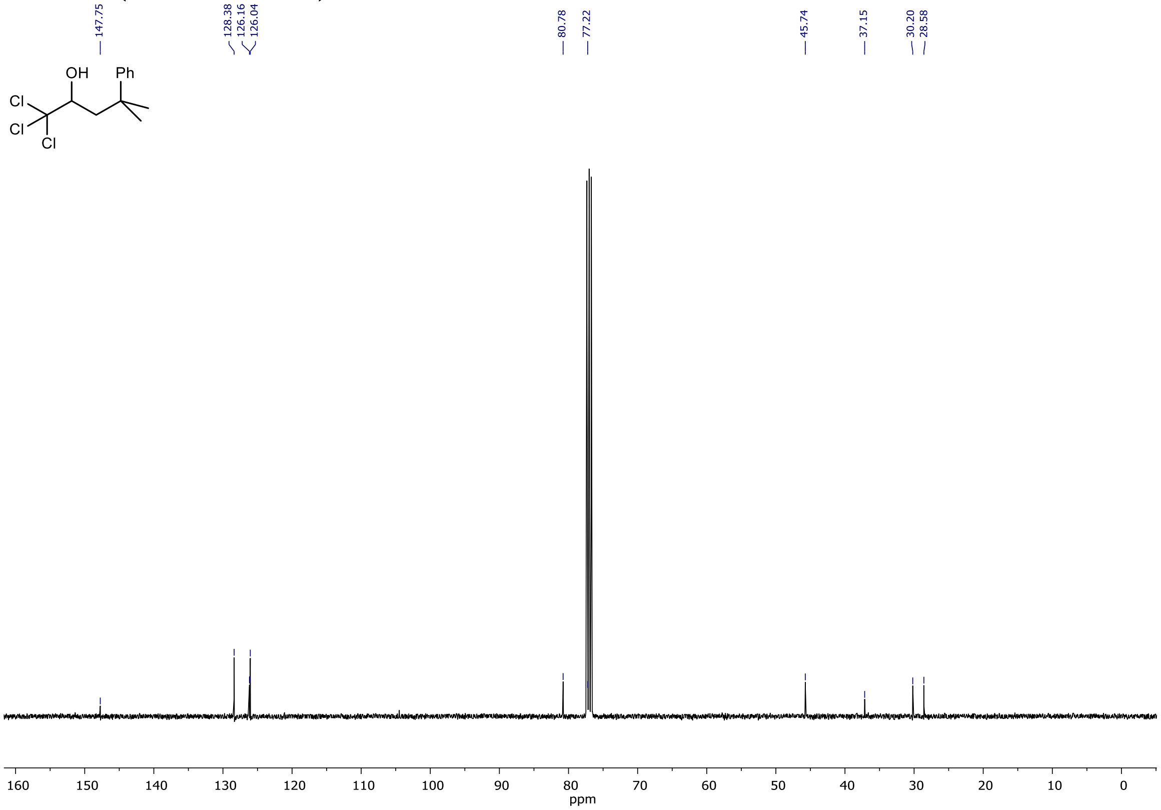

Supplementary Figure 123: NMR Spectra of 1,1,1-Trichloro-4-methyl-4-phenylpentan-2-ol (3s)

^{1}H NMR (300 MHz, $CDCl_3$)

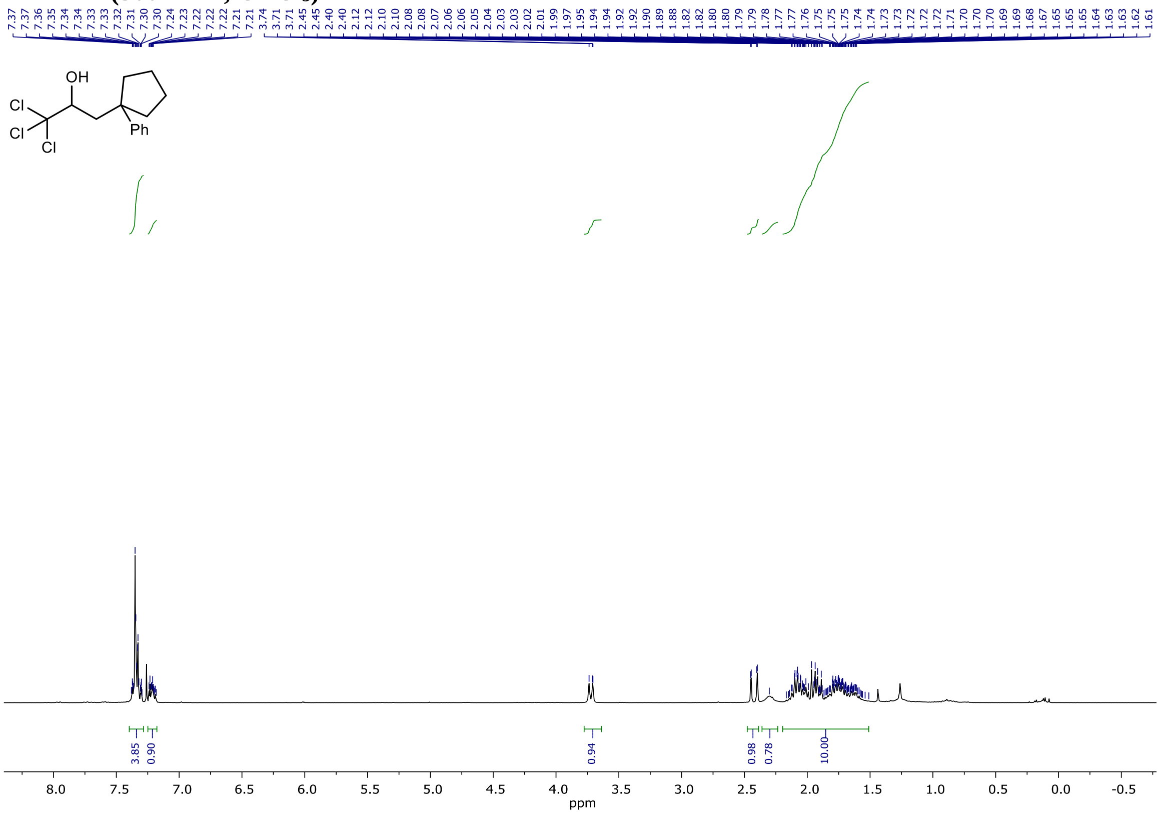

^{13}C NMR (75 MHz, $CDCl_3$)

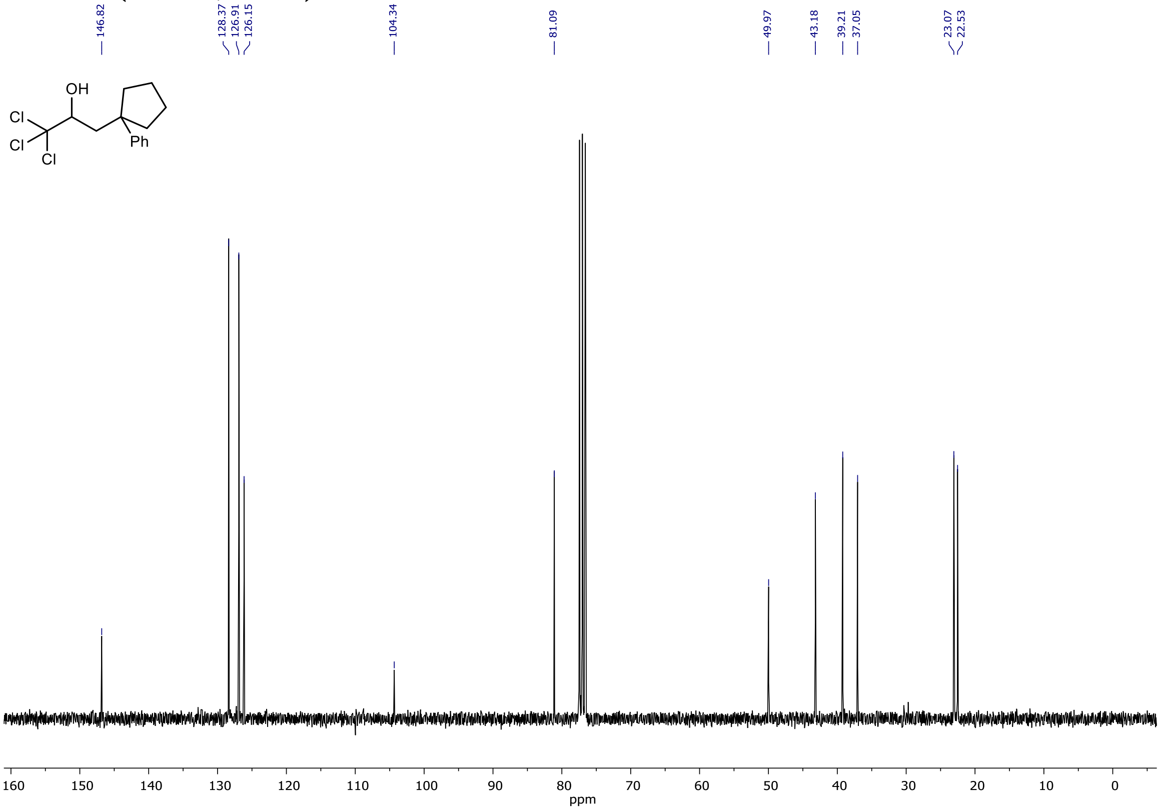

Supplementary Figure 124: NMR Spectra of 1,1,1-Trichloro-3-(1-phenylcyclopentyl)propan-2-ol (3t)

^{1}H NMR (300 MHz, $CDCl_3$)

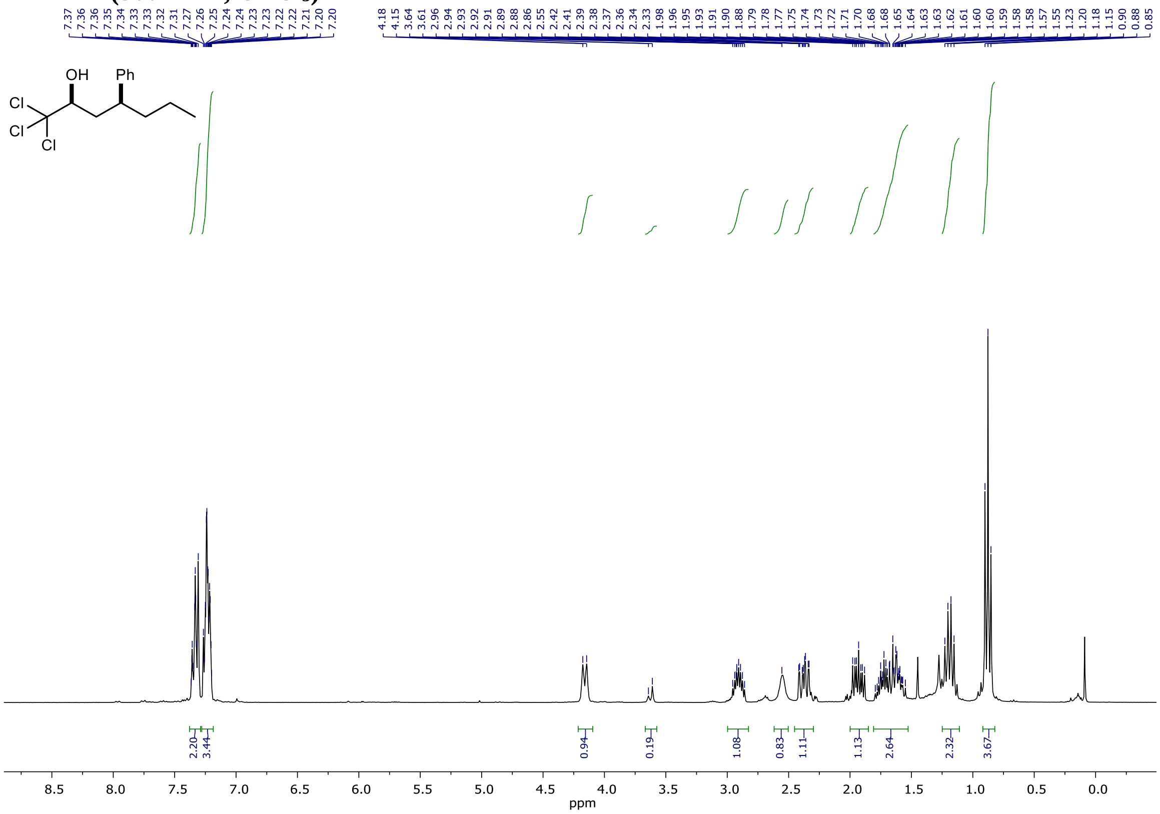

^{13}C NMR (75 MHz, $CDCl_3$)

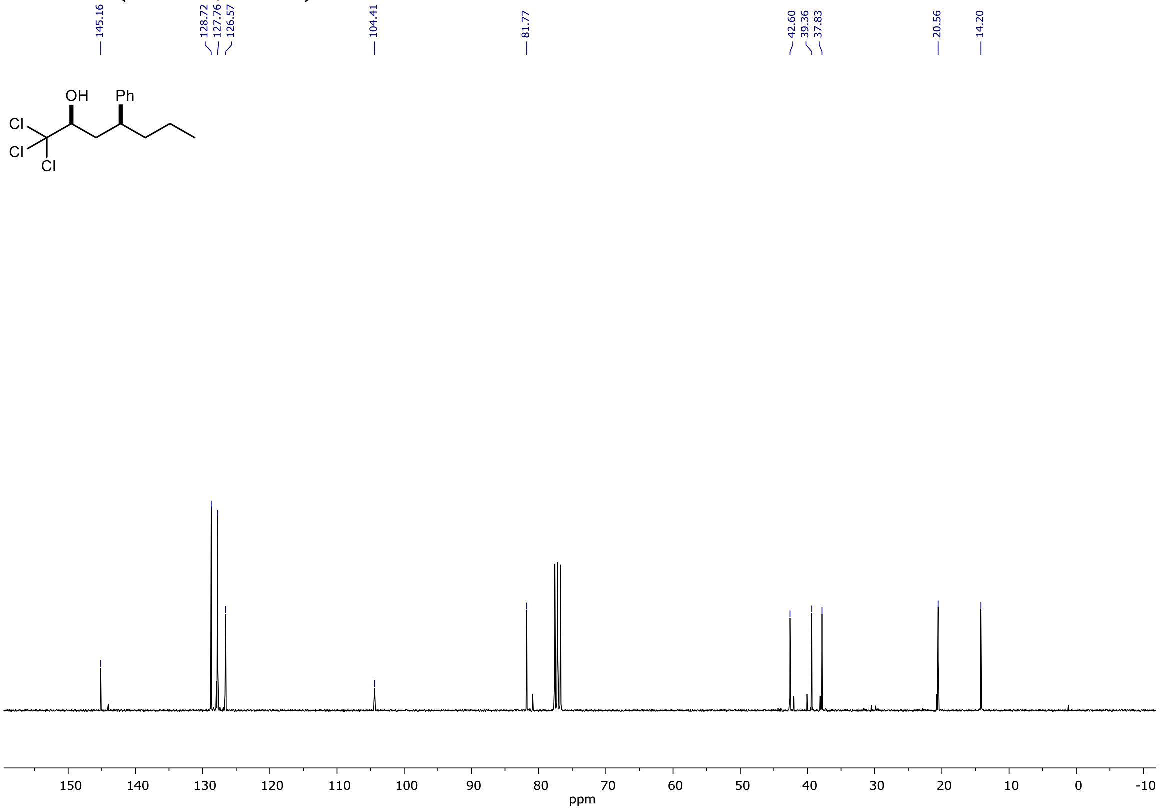

Supplementary Figure 125: NMR Spectra of 1,1,1-Trichloro-4-phenylheptan-2-ol (3u)

^{1}H NMR (300 MHz, CDCl$_3$)

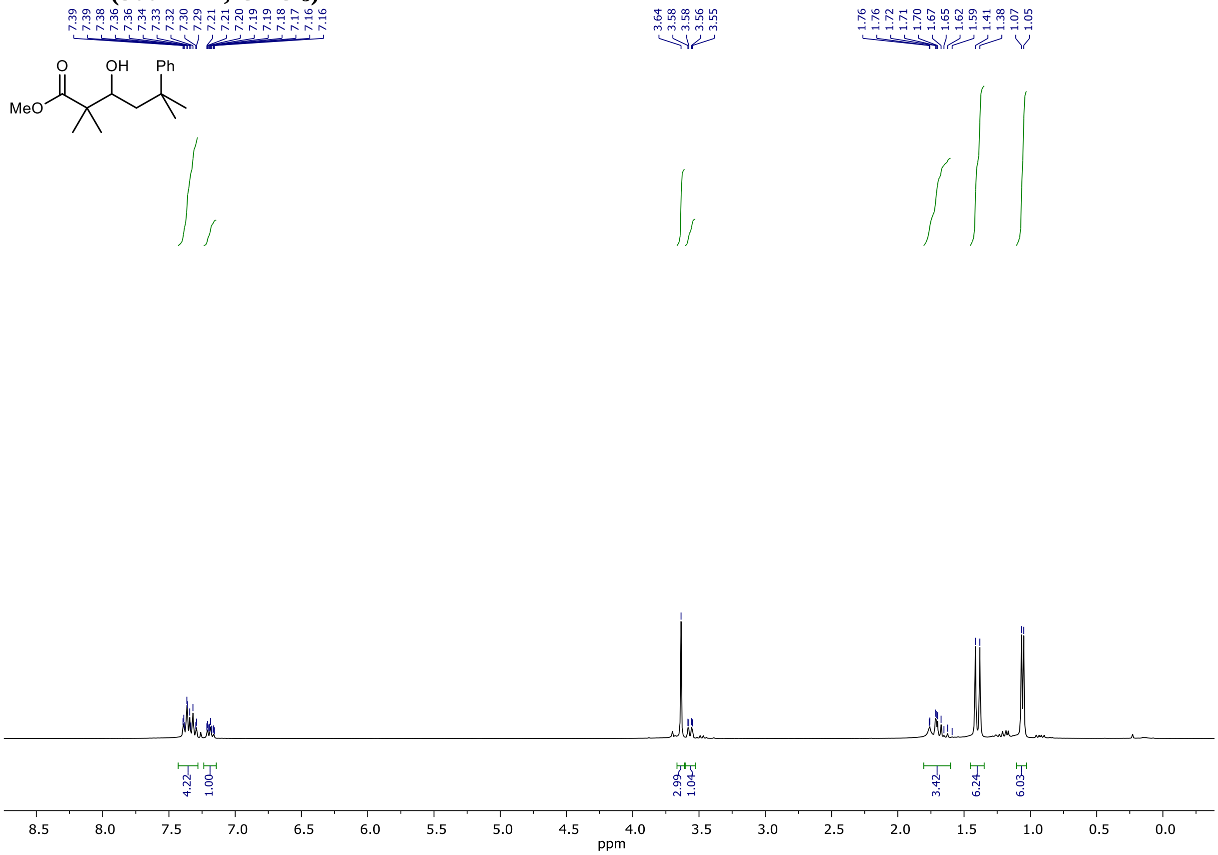

^{13}C NMR (75 MHz, CDCl$_3$)

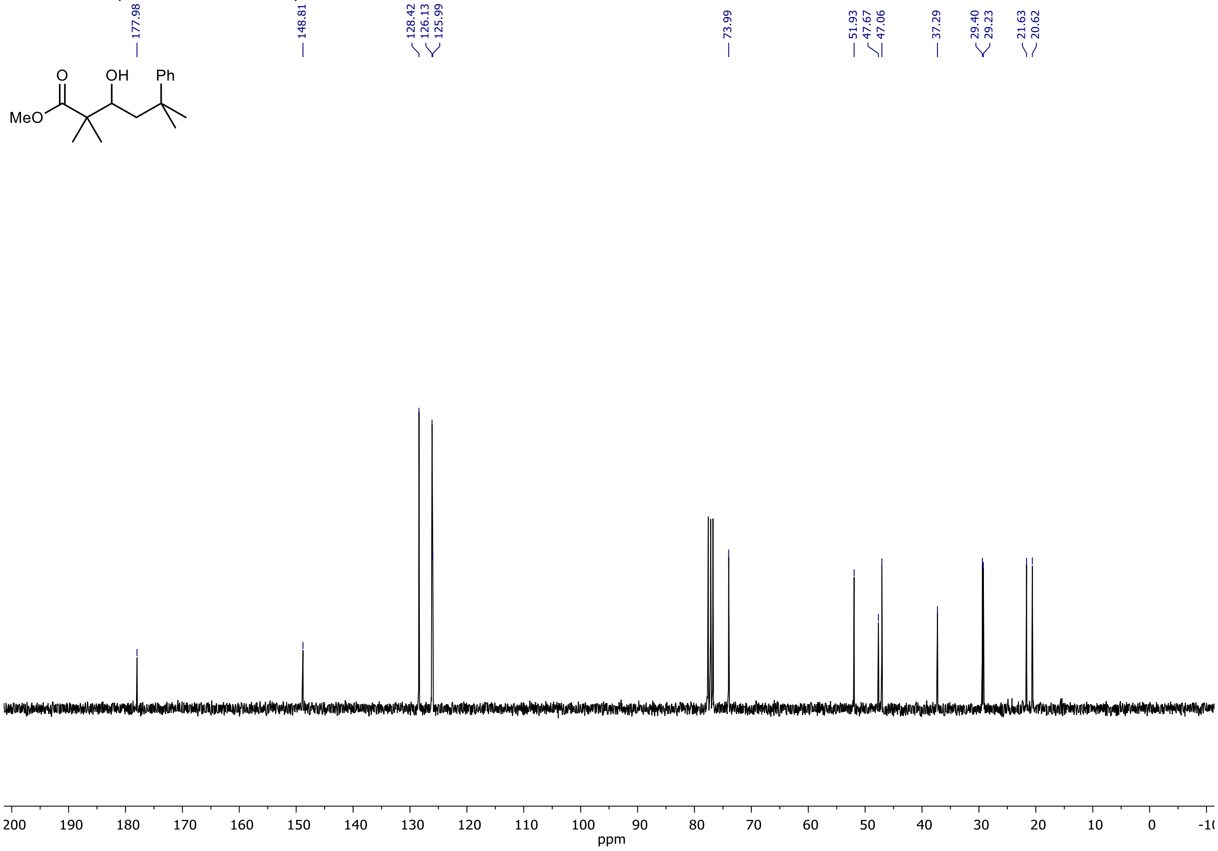

Supplementary Figure 126: NMR Spectra of Methyl 3-hydroxy-2,2,5-trimethyl-5-phenylhexanoate (3v)

^{1}H NMR (300 MHz, CDCl$_3$)

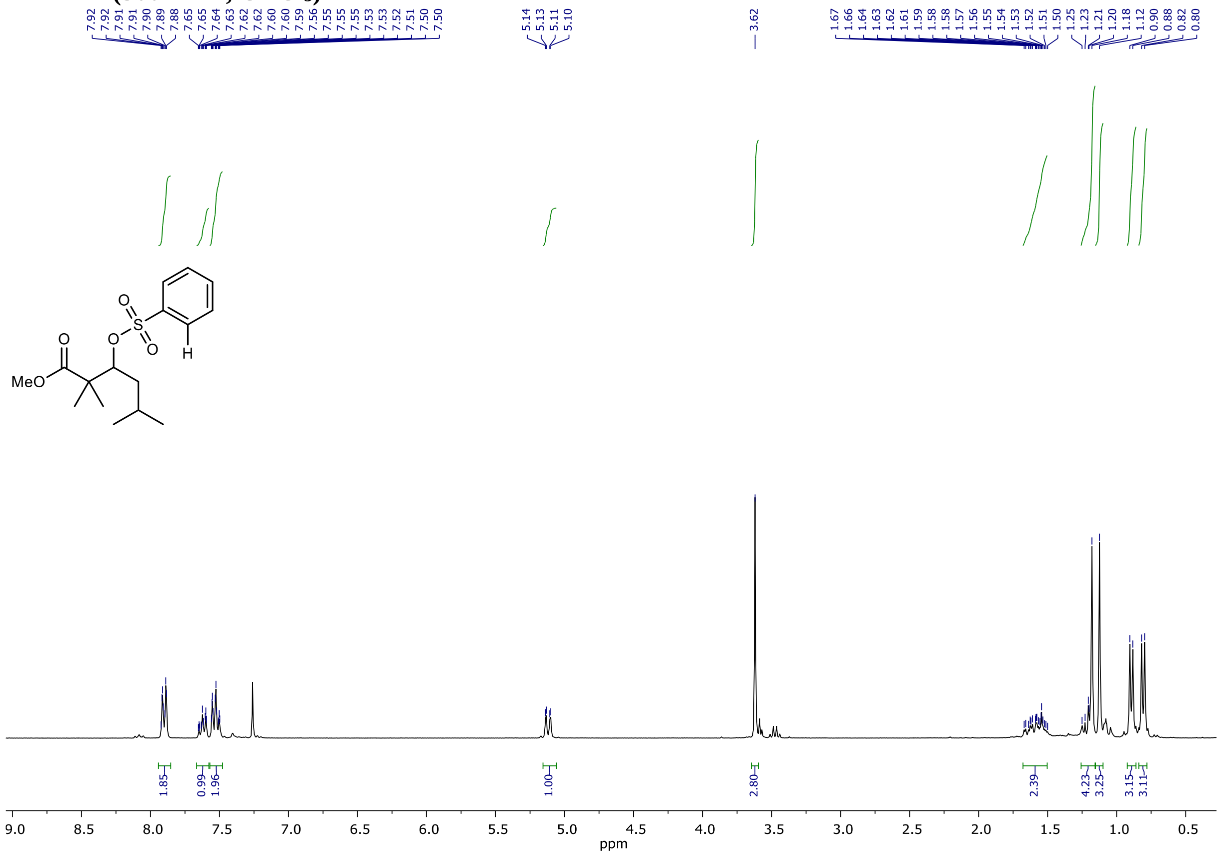

^{13}C NMR (75 MHz, CDCl$_3$)

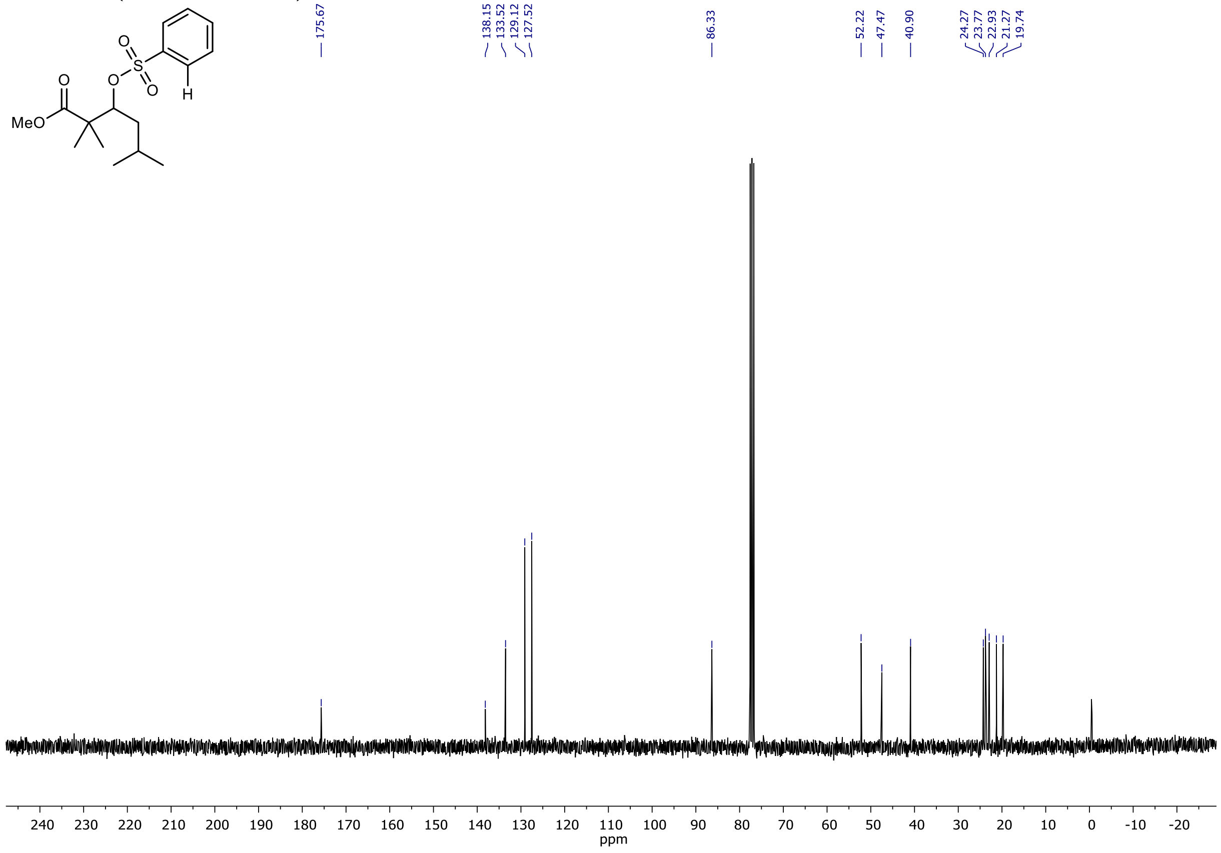

Supplementary Figure 127: NMR Spectra of Methyl 2,2,5-trimethyl-3-((phenylsulfonyl)oxy)hexanoate (S-25)

^{1}H NMR (400 MHz, $CDCl_3$)

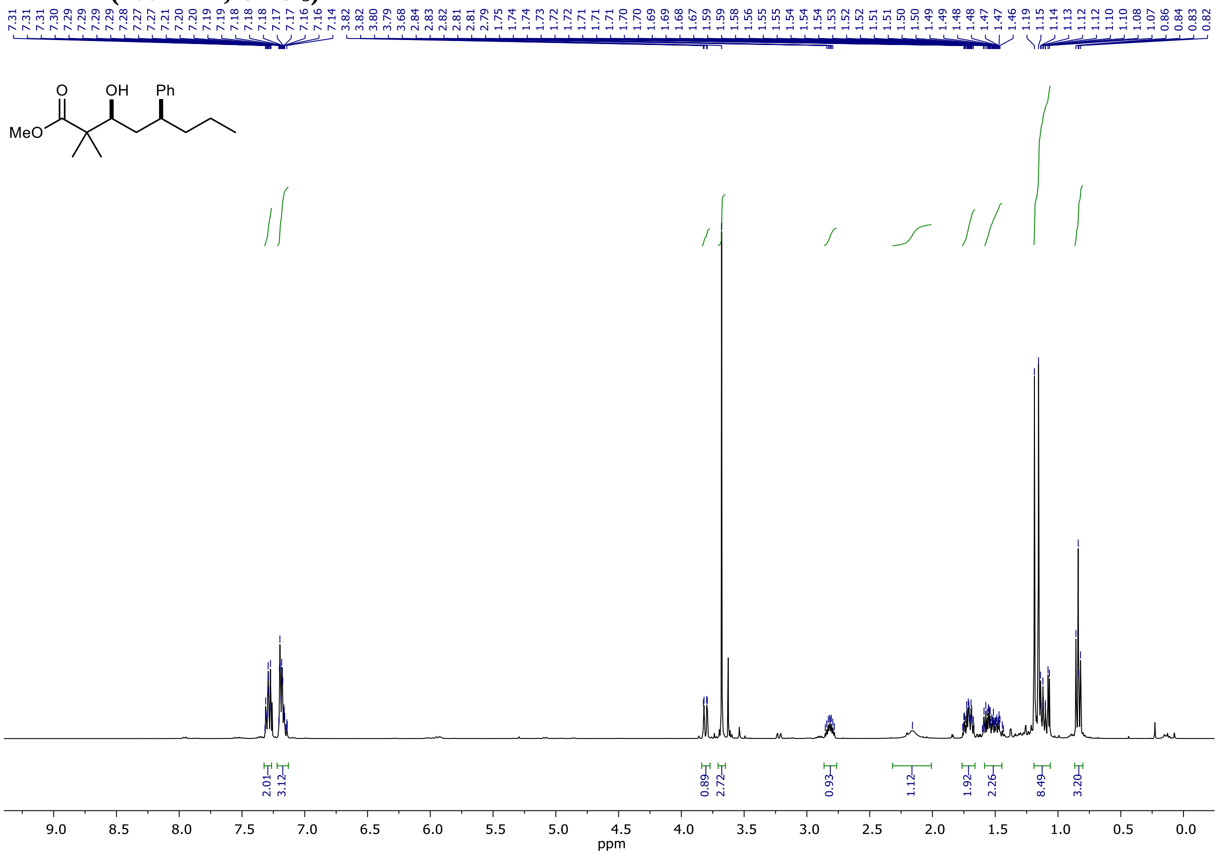

^{13}C NMR (101 MHz, $CDCl_3$)

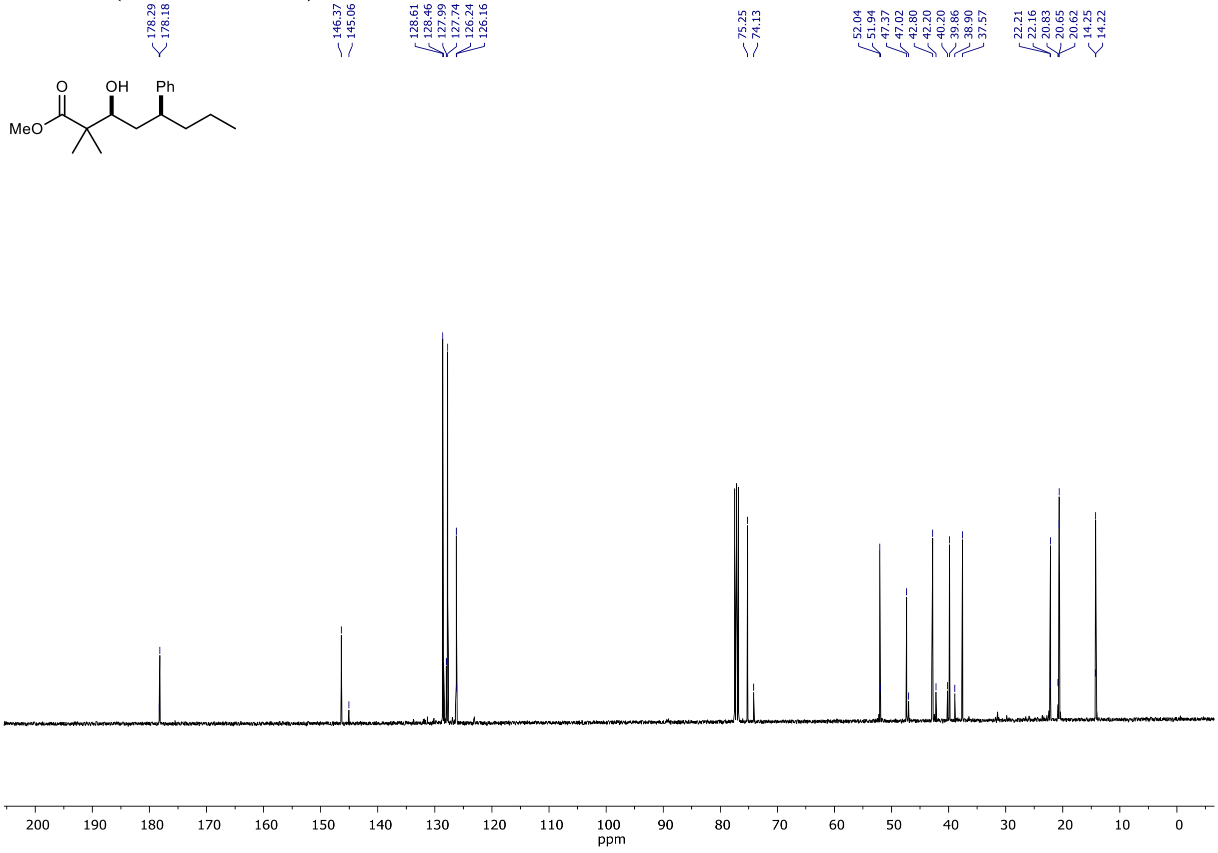

Supplementary Figure 128: NMR Spectra of rac Methyl (3S,5S)-3-hydroxy-2,2-dimethyl-5-phenyloctanoate (3w)

^{1}H NMR (400 MHz, $CDCl_3$)

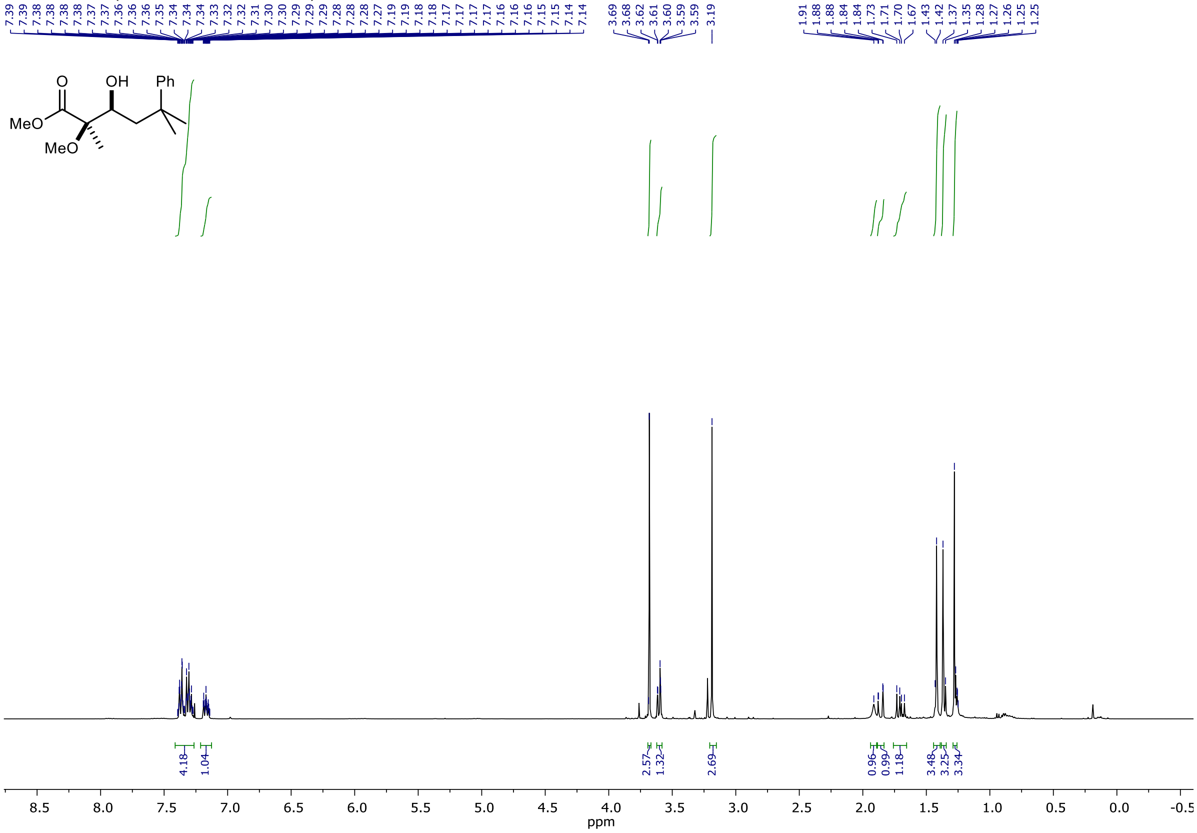

^{13}C NMR (101 MHz, $CDCl_3$)

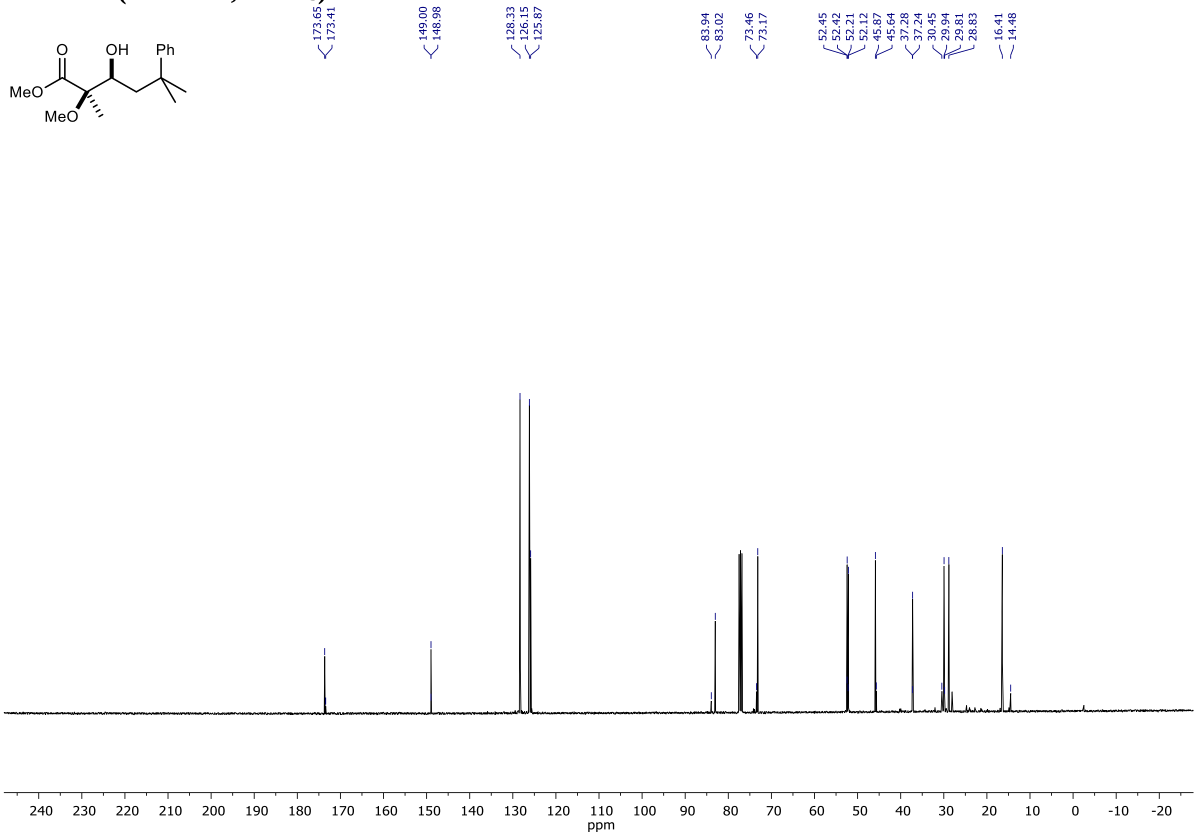

Supplementary Figure 129: NMR Spectra of rac Methyl (2R,3S)-3-hydroxy-2-methoxy-2,5-dimethyl-5-phenylhexanoate (3x)

^{1}H NMR (400 MHz, $CDCl_3$)

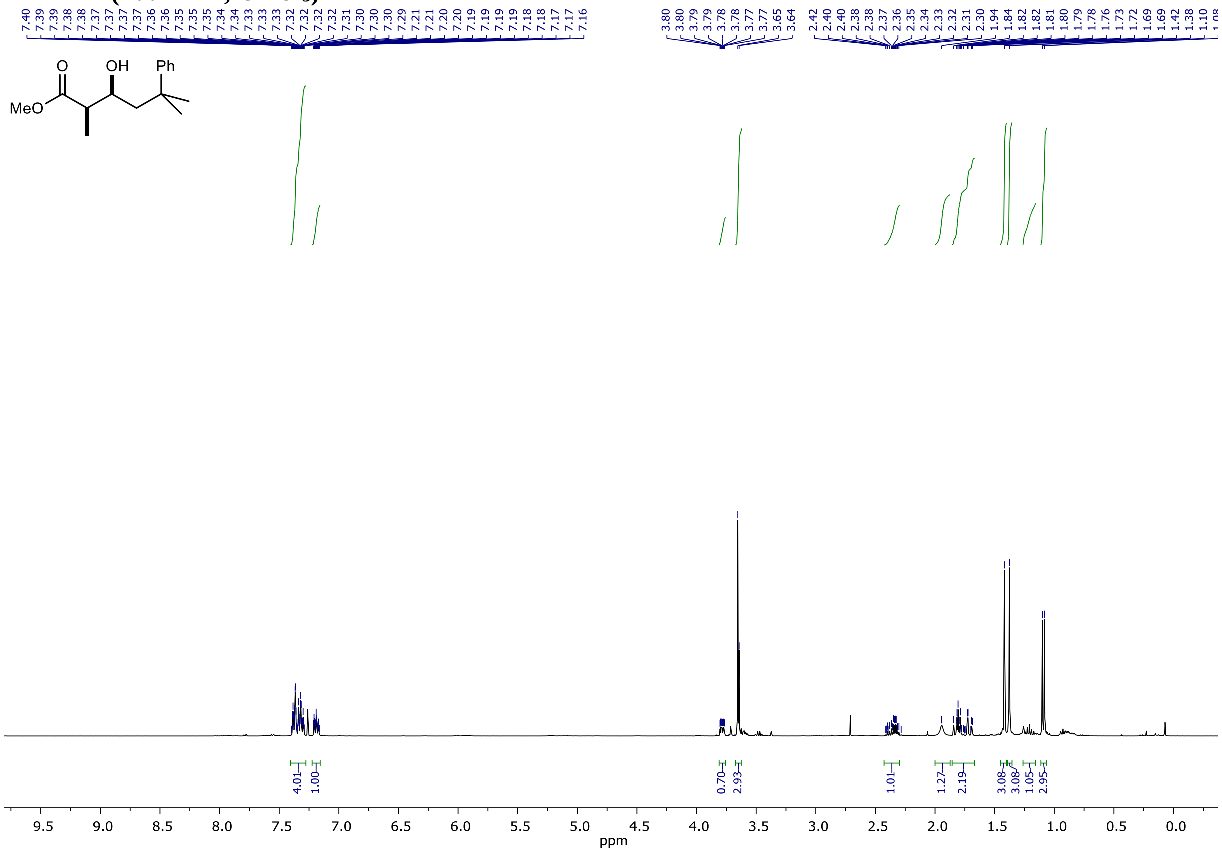

^{13}C NMR (101 MHz, $CDCl_3$)

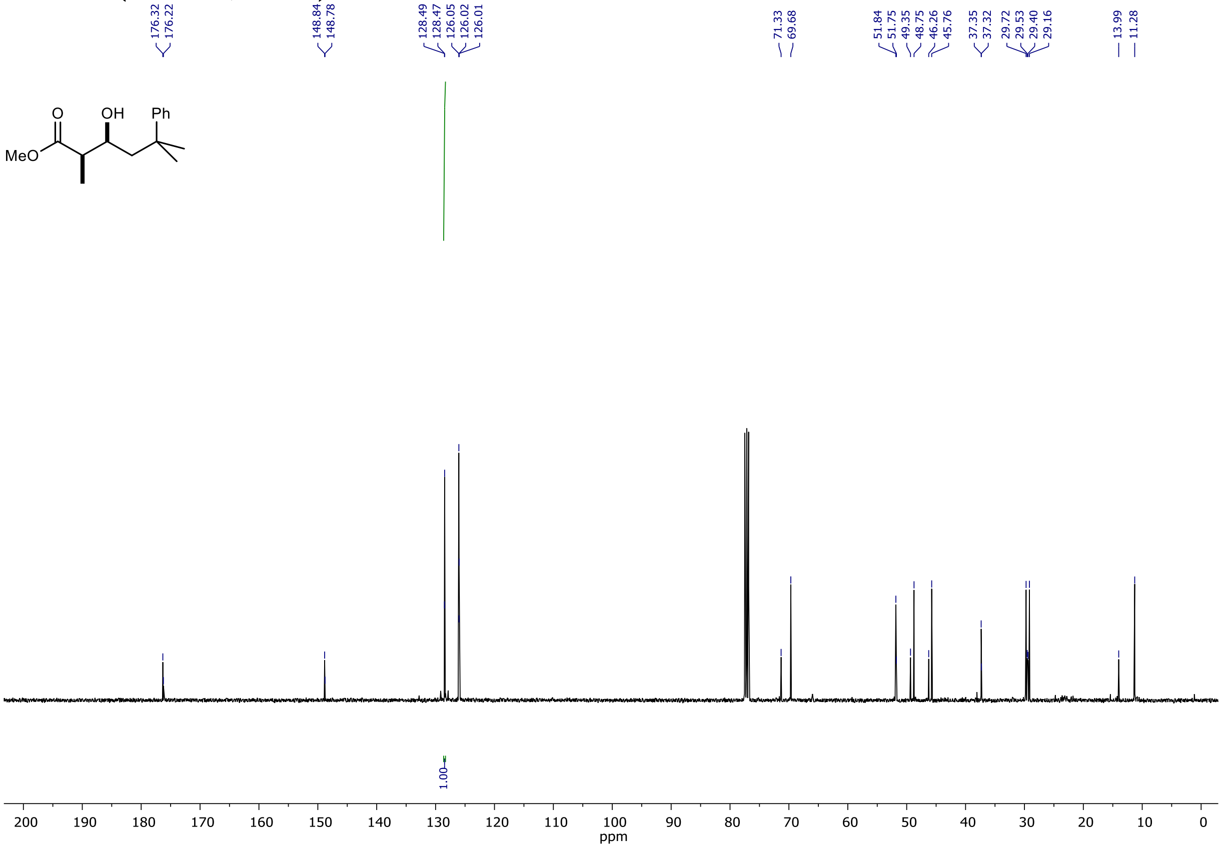

Supplementary Figure 130: NMR Spectra of *rac* Methyl (2*R*,3*S*)-3-hydroxy-2,5-dimethyl-5-phenylhexanoate (3y)

^{1}H NMR (400 MHz, CDCl$_3$)

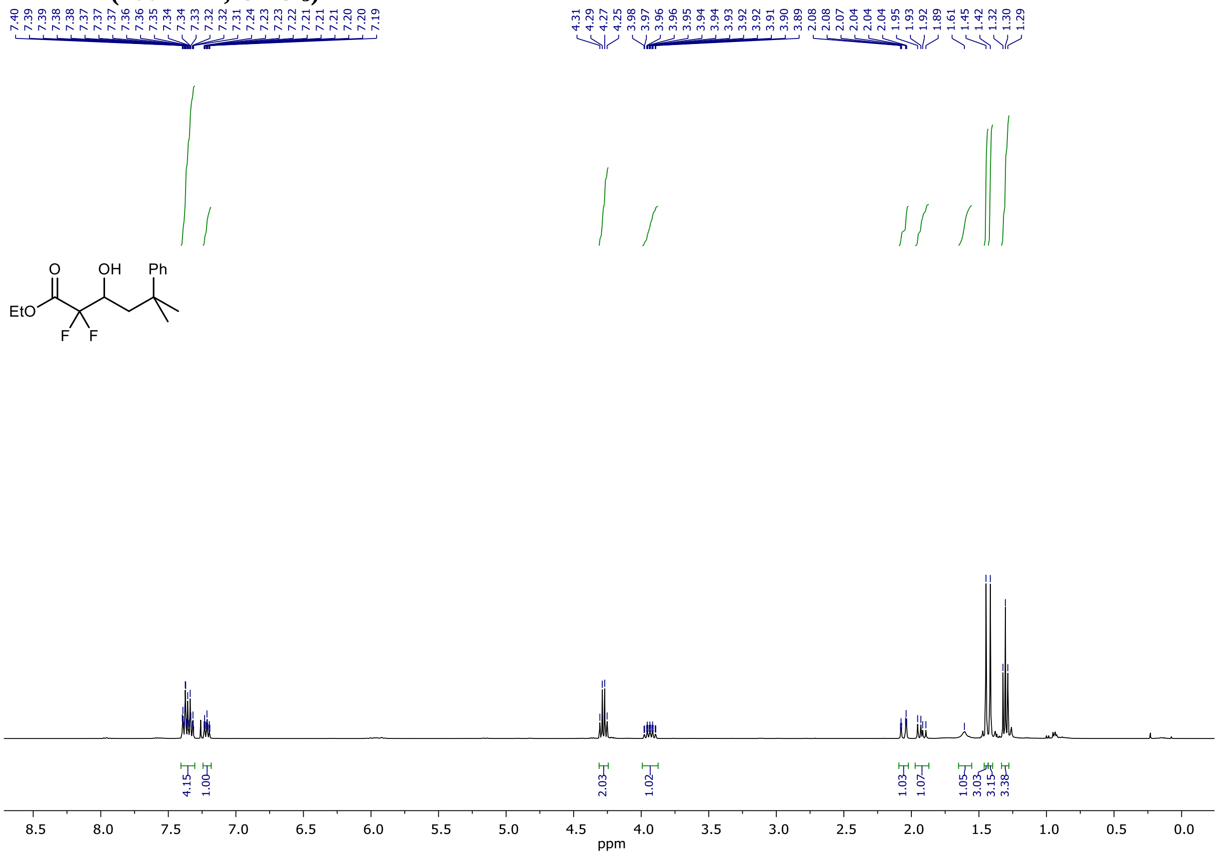

^{13}C NMR (101 MHz, CDCl$_3$)

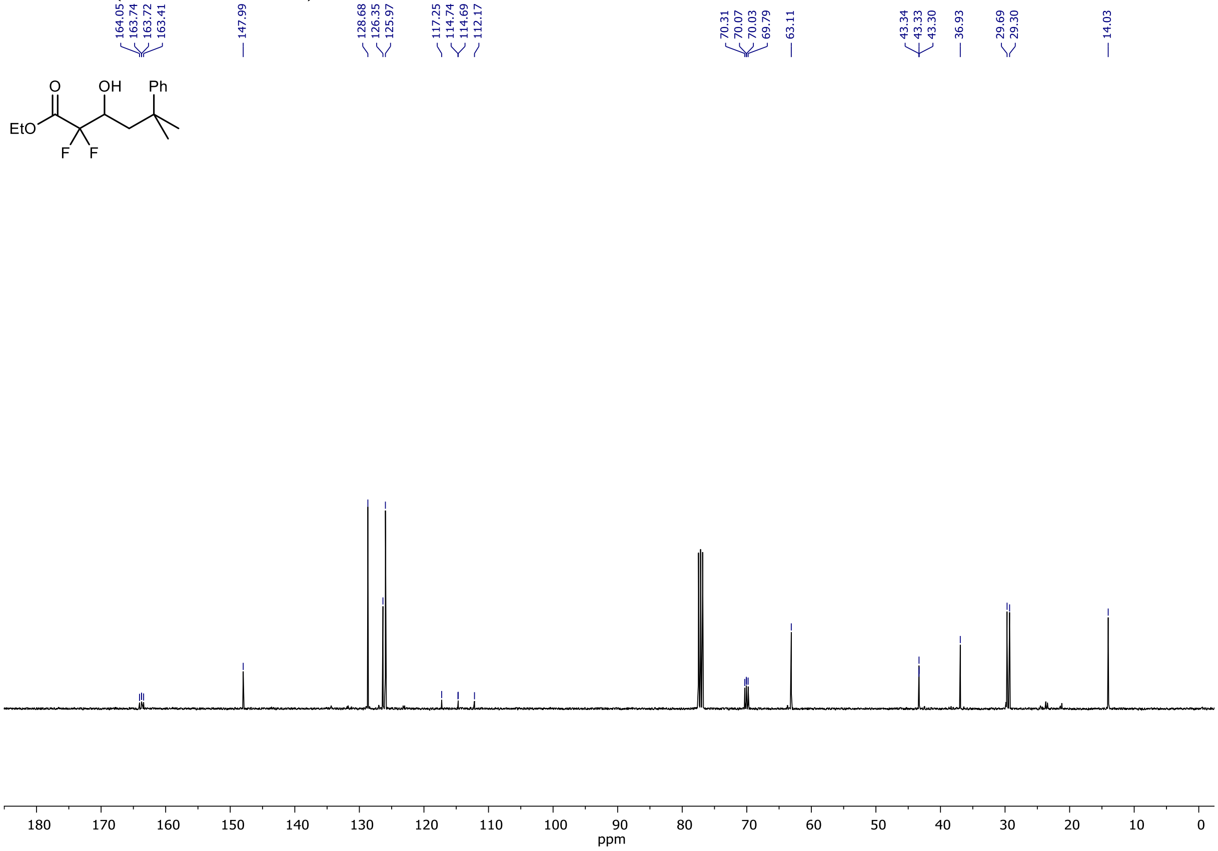

$^{19}F\{^{1}H\}$ NMR (282 Hz, $CDCl_3$)

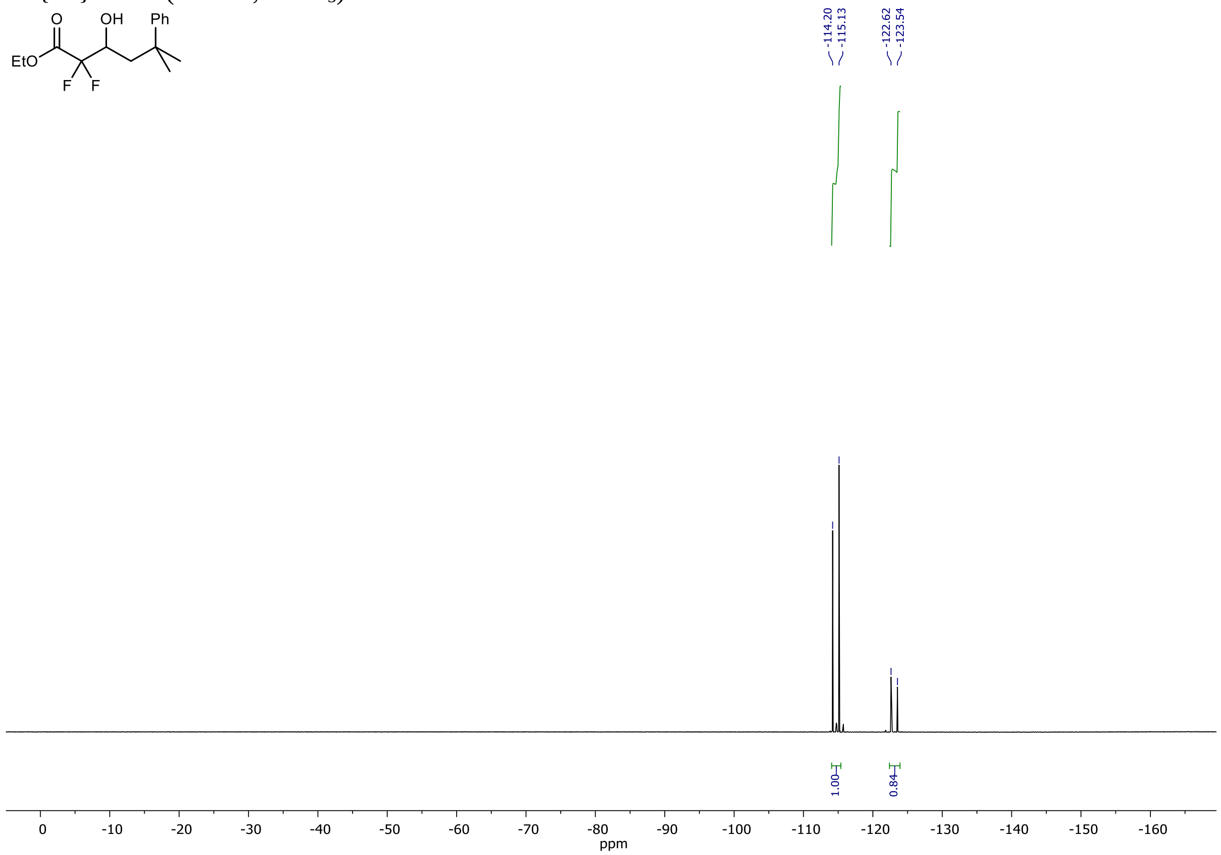

Supplementary Figure 131: NMR Spectra of Ethyl 2,2-difluoro-3-hydroxy-5-methyl-5-phenylhexanoate (3z)

^{1}H NMR (300 MHz, $CDCl_3$)

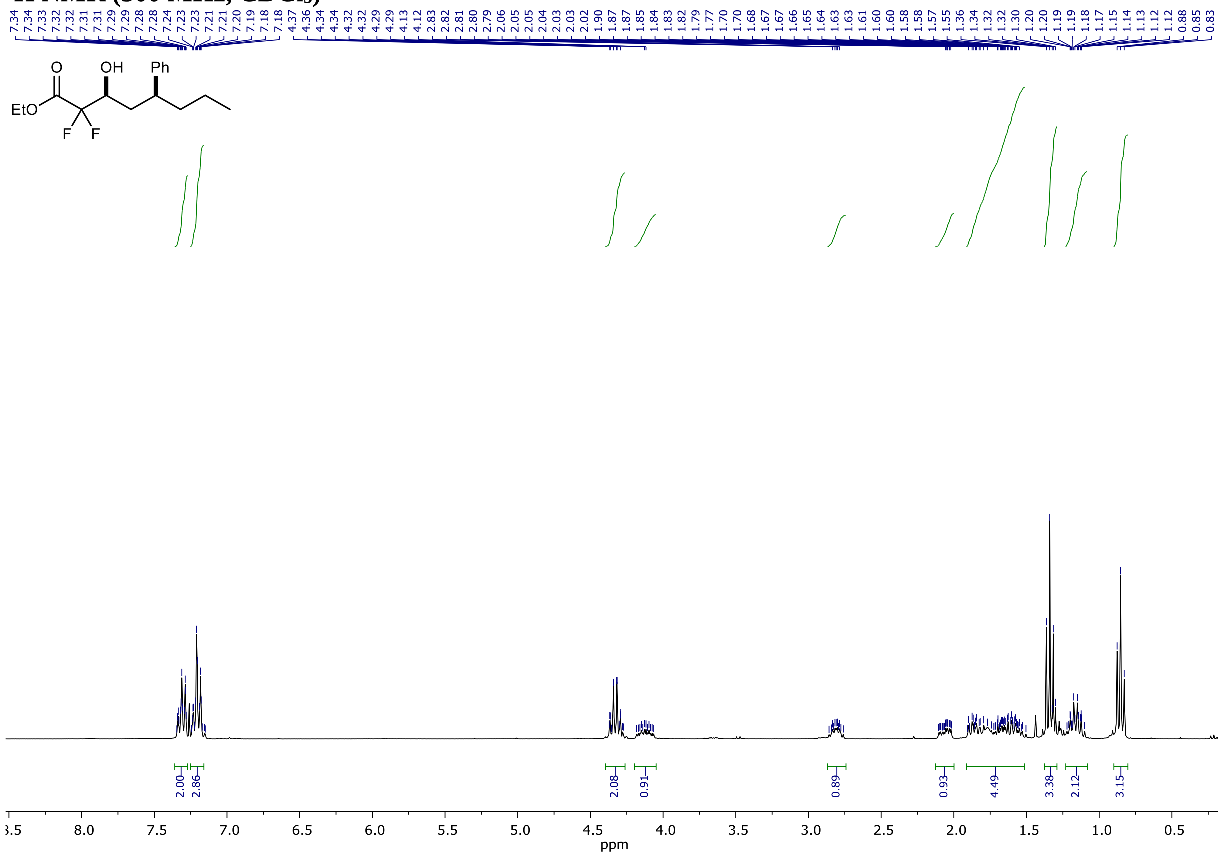

^{13}C NMR (75 MHz, $CDCl_3$)

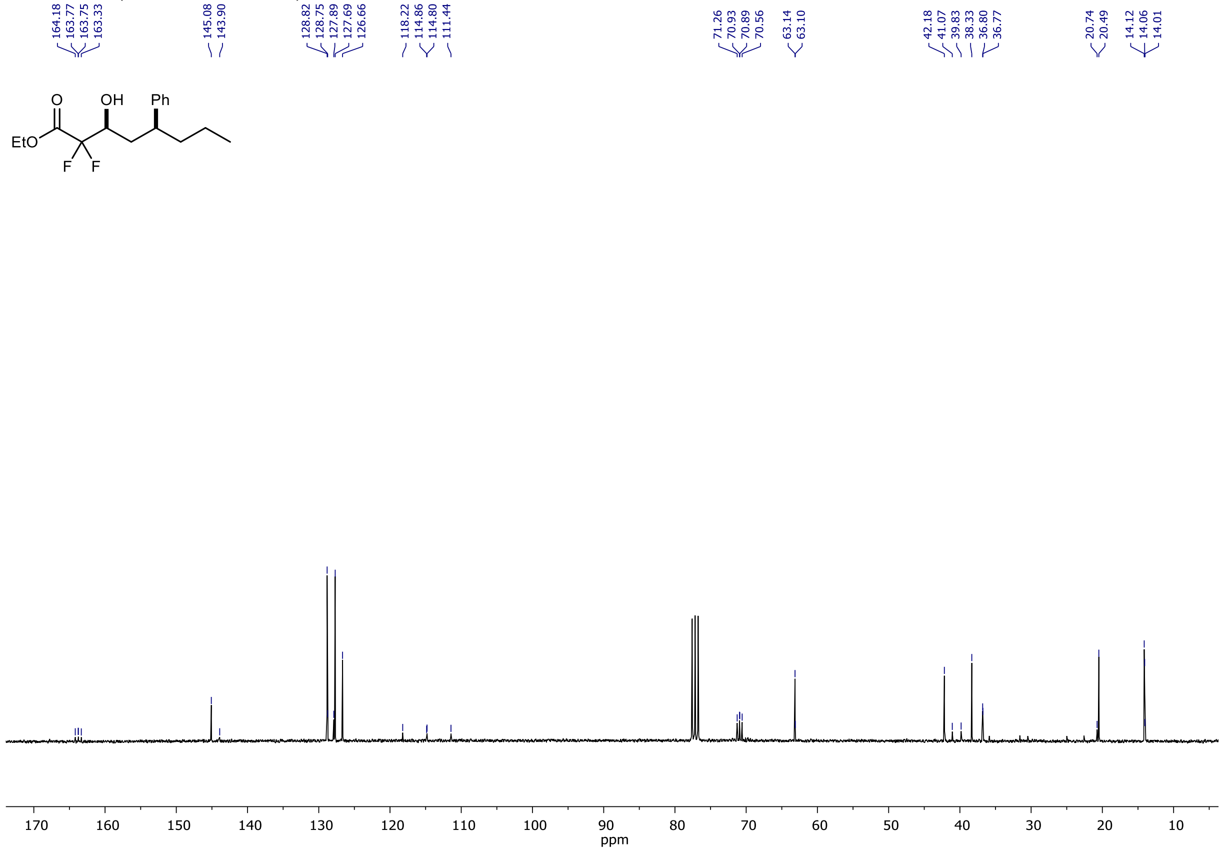

$^{19}F\{^1H\}$ NMR (282 Hz, $CDCl_3$)

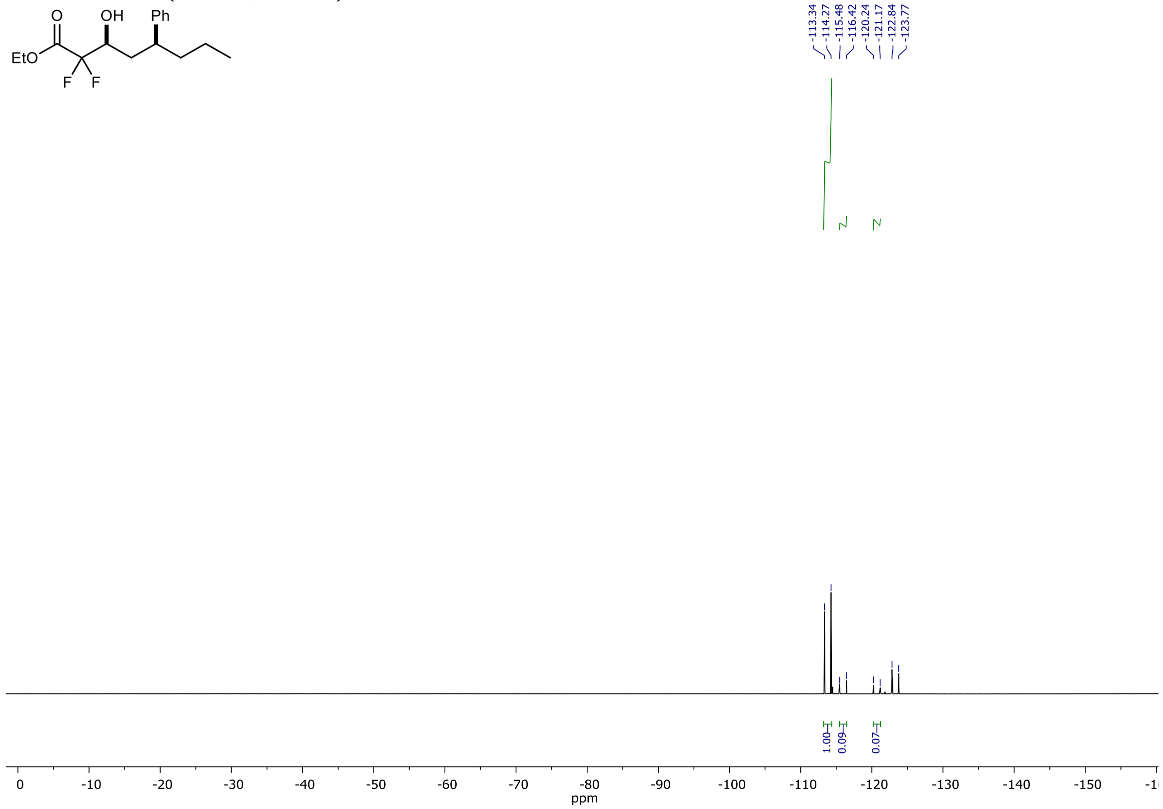

Supplementary Figure 132: NMR Spectra of *rac* Ethyl (3*S*,5*S*)-2,2-difluoro-3-hydroxy-5-phenyloctanoate(3aa)

^{1}H NMR (300 MHz, $CDCl_3$)

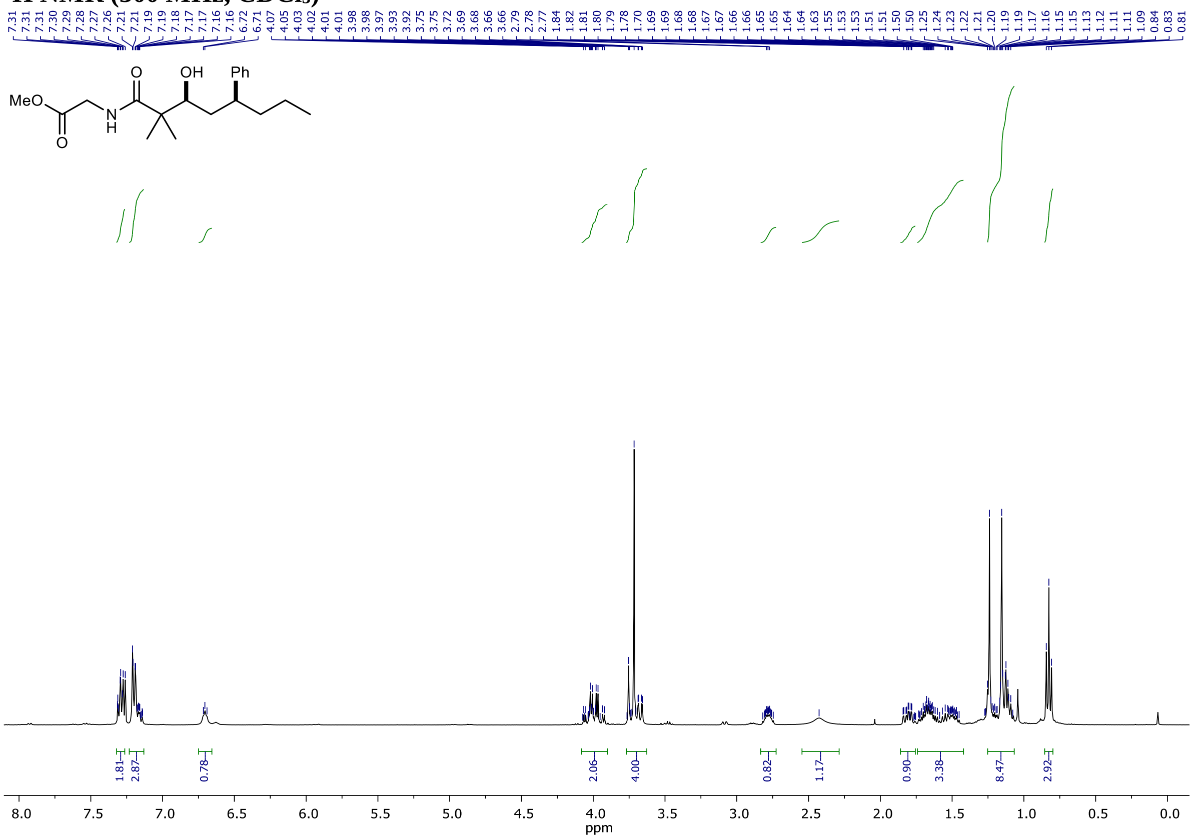

^{13}C NMR (126 MHz, $CDCl_3$)

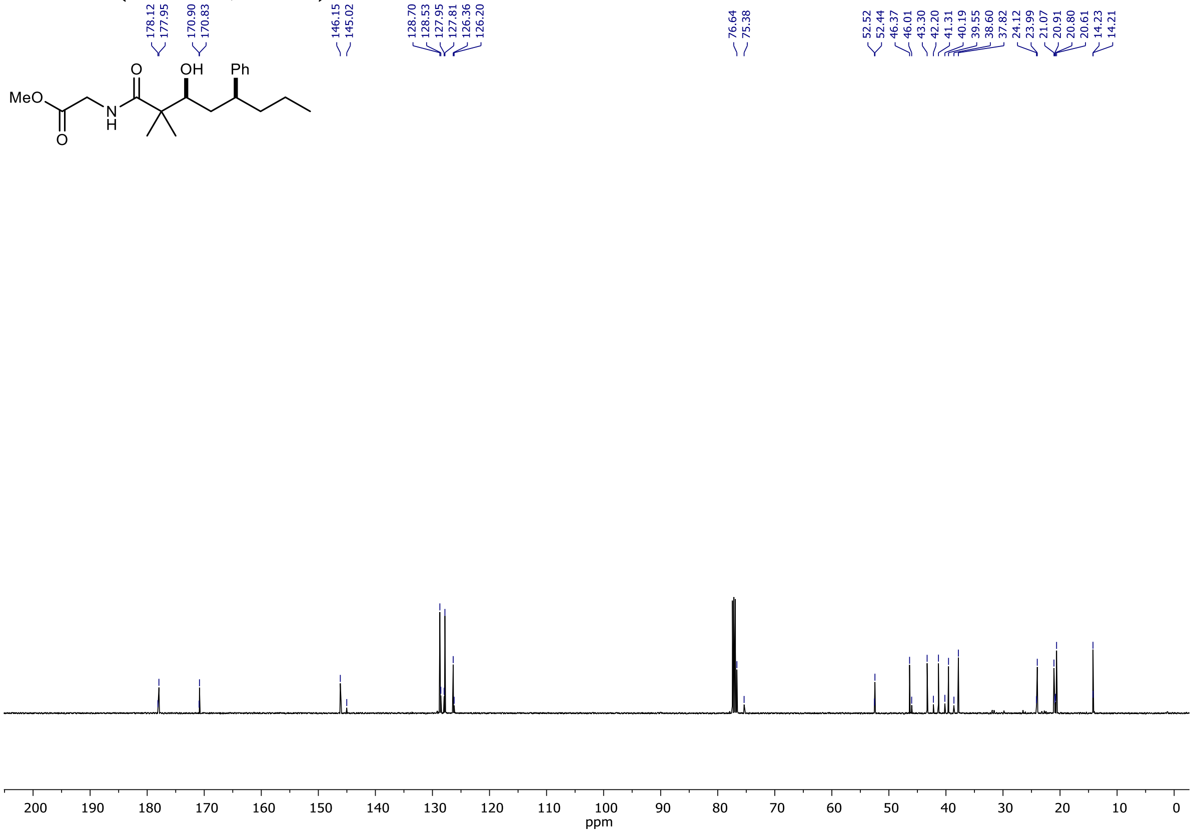

Supplementary Figure 133: NMR Spectra of *rac* Methyl ((3*S*,5*S*)-3-hydroxy-2,2-dimethyl-5-phenyloctanoyl)glycinate (3ab)

^{1}H NMR (300 MHz, $CDCl_3$)

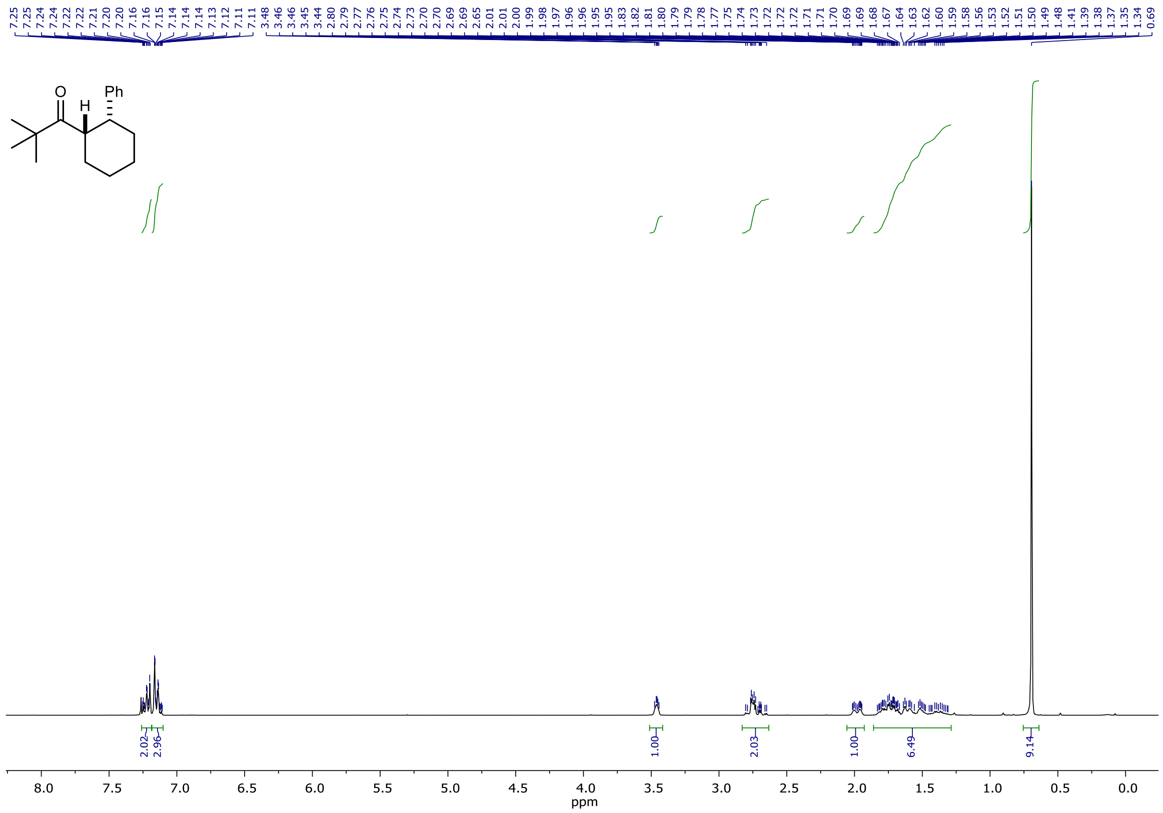

^{13}C NMR (75 MHz, $CDCl_3$)

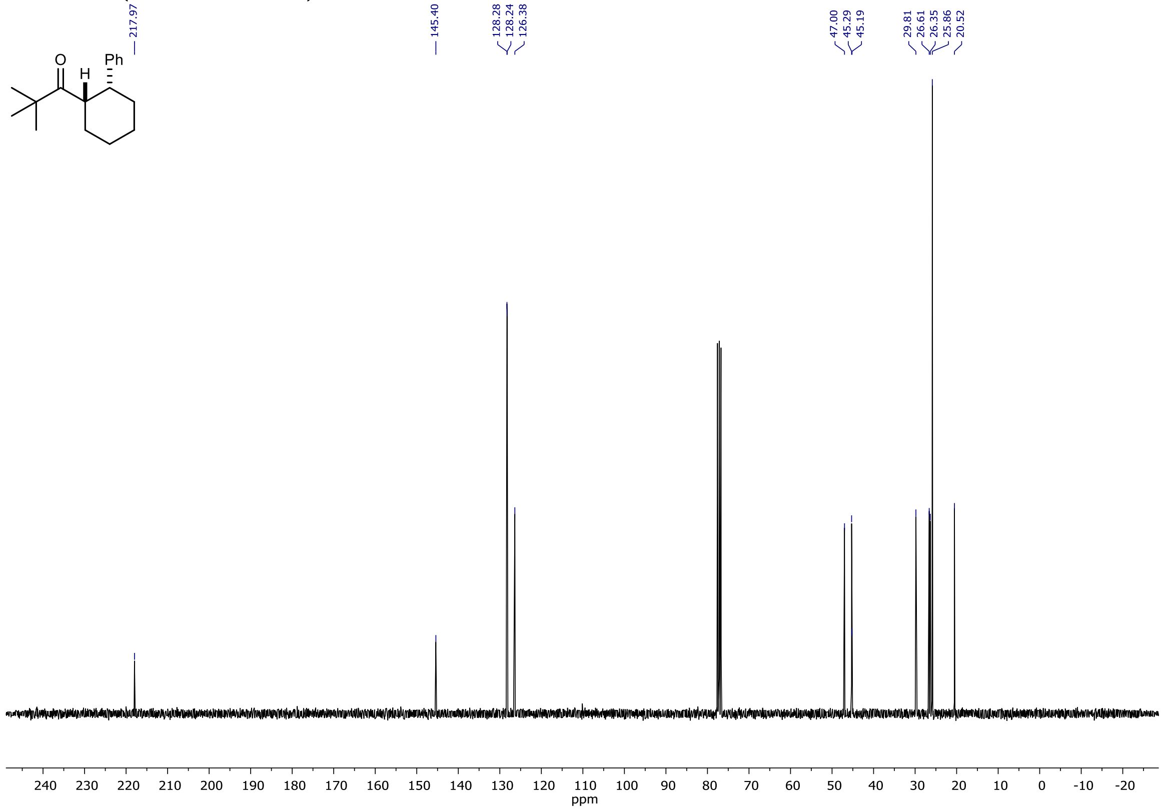

Supplementary Figure 134: NMR Spectra of *rac*-2,2-Dimethyl-1-((1S,2R)-2-phenylcyclohexyl)propan-1-one (S-26)

^{1}H NMR (300 MHz, $CDCl_3$) δ

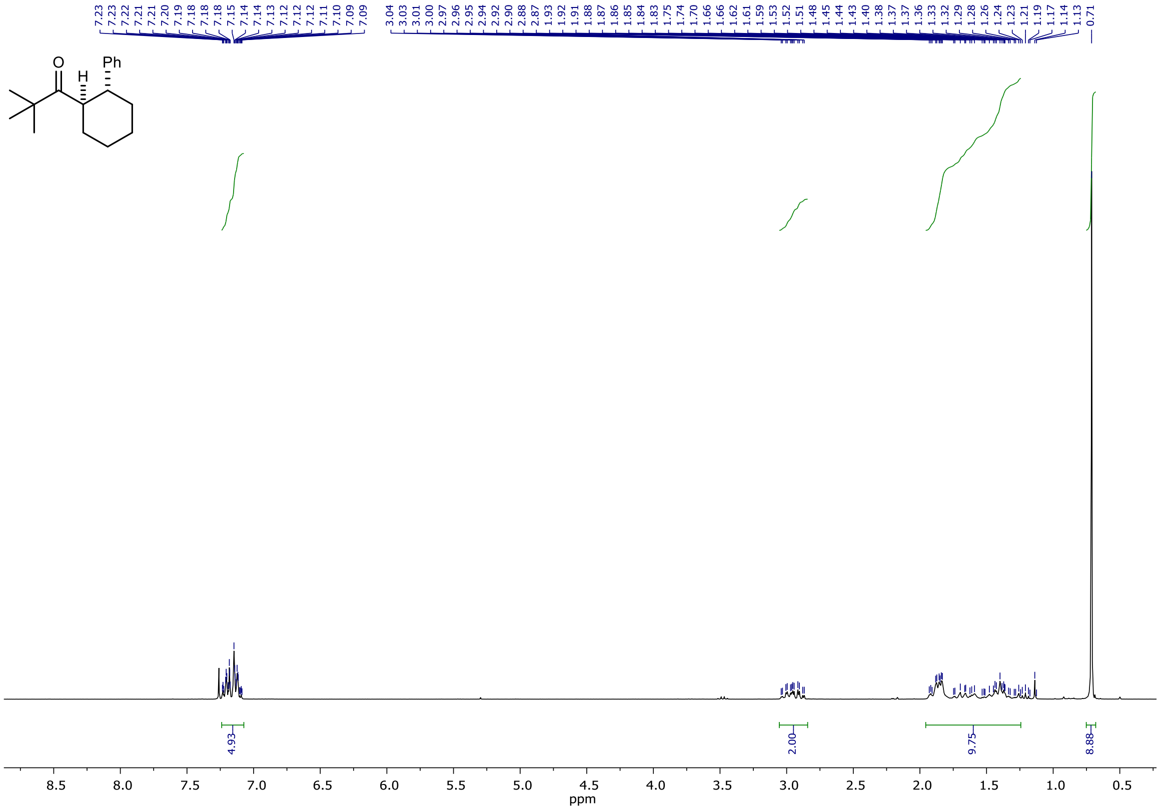

^{13}C NMR (75 MHz, $CDCl_3$)

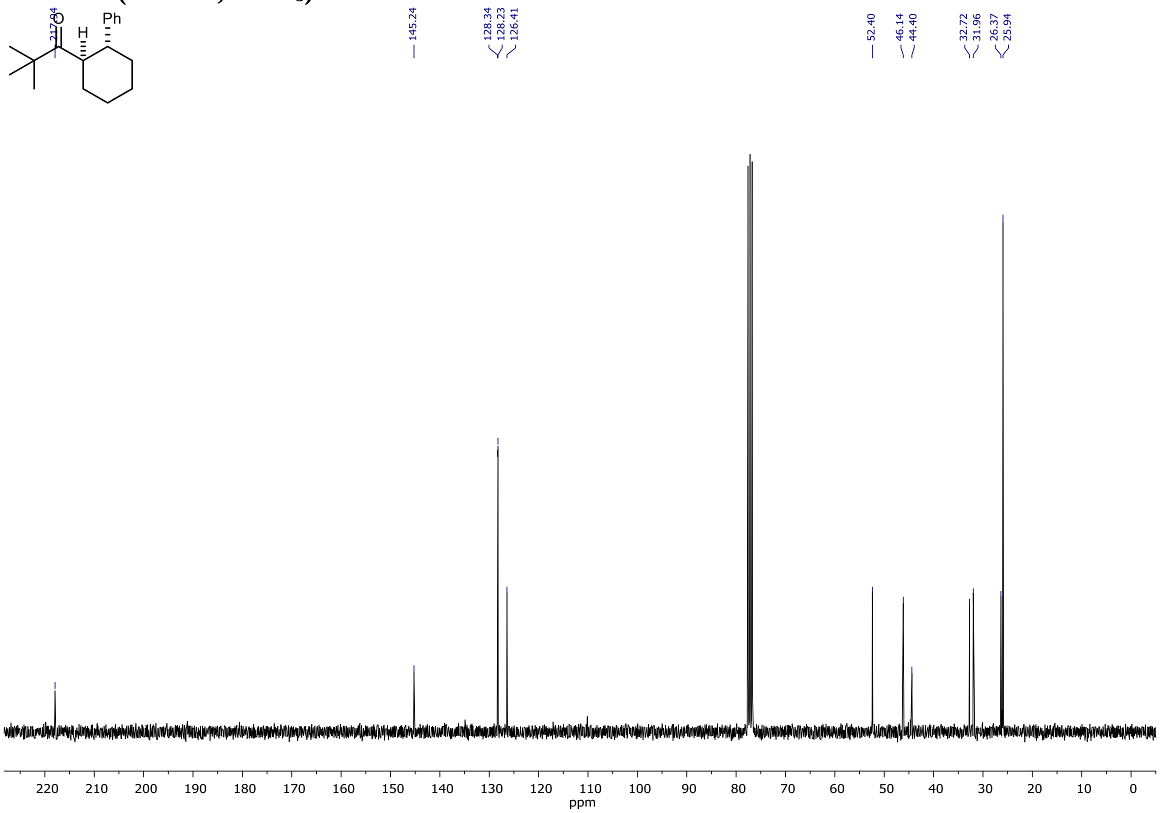

Supplementary Figure 135: NMR Spectra of *rac*-2,2-Dimethyl-1-((1*R*,2*R*)-2-phenylcyclohexyl)propan-1-one (S-27)

^{1}H NMR (300 MHz, $CDCl_3$)

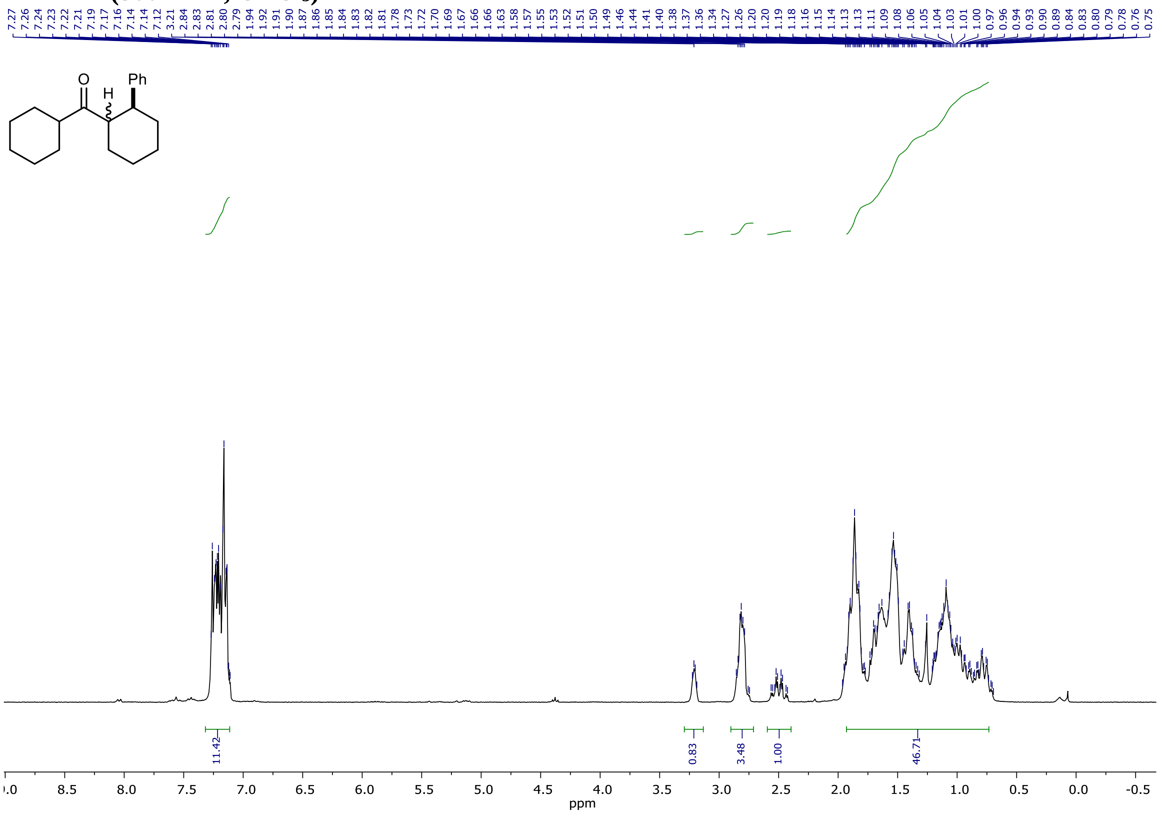

^{13}C NMR (75 MHz, $CDCl_3$)

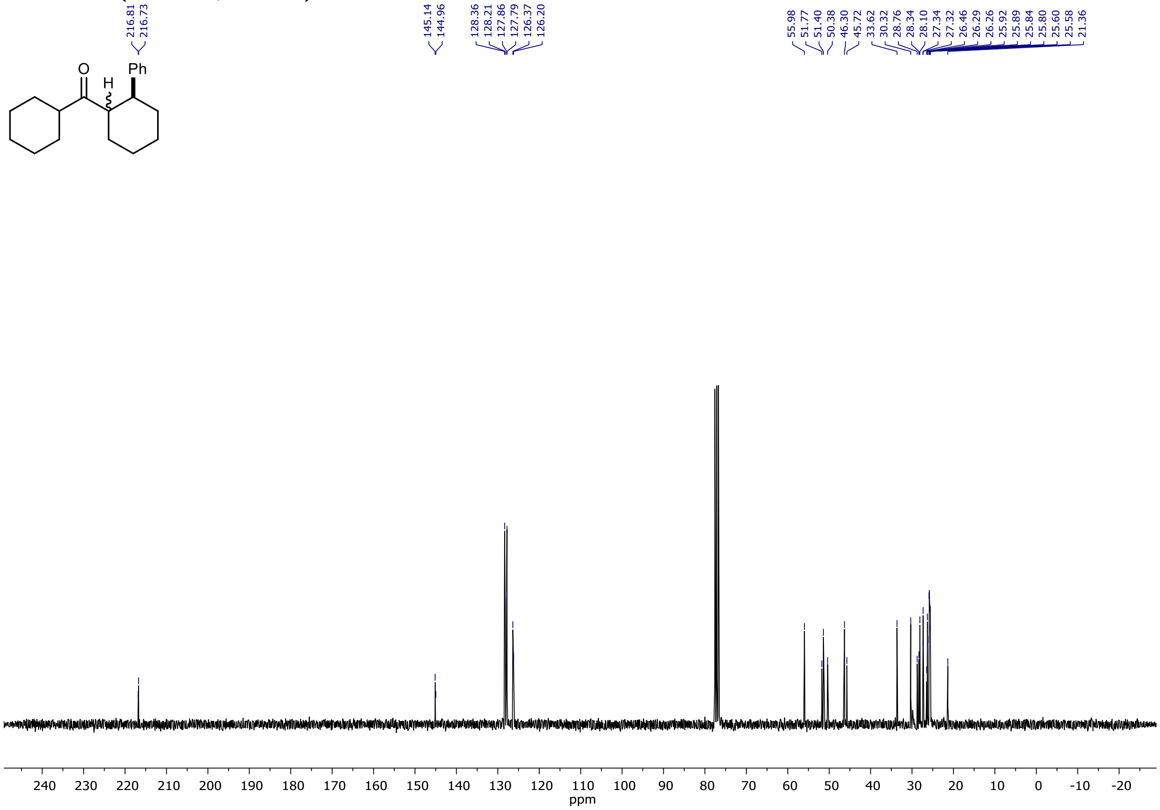

Supplementary Figure 136: NMR Spectra of Cyclohexyl(2-phenylcyclohexyl)methanone (S-28)

Supplementary References

(*1*) Chau, M. M. & Kice, J. L. The anomalous course of the reduction of diphenyl-2,2'-disulfonyl chloride. An old mystery reexamined and explained. *J. Org. Chem.* **42**, 3265–3270 (1977).

(*2*) Becker, H. G., Berger; W. & Domschke, G. *Organikum: Organisch-chemisches Grundpraktikum* (Wiley-VCH, Weinheim, 1998).

(*3*) Yoshida, Y., Sakakura, Y., Aso, N., Okada, S. & Tanabe, T. Practical and efficient methods for sulfonylation of alcohols using Ts(Ms)Cl/Et_3N and catalytic Me_3H·HCl as combined base: Promising alternative to traditional pyridine. *Tetrahedron* **55**, 2183–2192 (1999).

(*4*) Koser, G. F., Sun, G., Porter, C. W. & Youngs, W. J. Synthesis of 1H-1-(1-alkynyl)-5-methyl-1,2,3-benziodoxathiole 3,3-dioxides: alkynyl(aryl)iodonium sulfonates with heterocyclic iodine. *J. Org. Chem.* **58** ,7310–7312 (1993**)**.

(*5*) Kazemi, F., Massah A. R., Javaherian, M. Chemoselective and scalable preparation of alkyl tosylates under solvent-free conditions. *Tetrahedron* **63**, 5083-5087 (2007).

(*6*) Bonfiglio, J. N. Directed Ortho-Lithiation of Alkyl Arenesulfonates. *J. Org. Chem.* **51**, 2835–2838 (1986).

(*7*) Huynh, C., Derguini-Boumechal, F. & Linstrumelle, G. Copper-catalysed reactions of grignard reagents with epoxides and oxetane. *Tetrahedron Lett.* **20**, 1503−1506 (1979).

(*8*) Eagon, S., DeLieto, C., McDonald, W. J., Haddenham, D., Saavedra, J., Kim, J., Singaram, B. Mild and Expedient Asymmetric Reductions of α,β-Unsaturated Alkenyl and Alkynyl Ketones by TarB-NO2 and Mechanistic Investigations of Ketone Reduction. *J. Org. Chem*. **75**, 7717–7725 (2010).

(*9*) Hull, C., Mortlock, S. V. & Thomas, E. J. On the stereoselectivity of reaction between α-methylbut-2-enylstannanes and aldehydes. *Tetrahedron*, **45**, 1007−1015 (1989).

(*10*) Kumar, P. & Saravanan, K. Facile synthesis of unsaturated lactones via intramolecular Wittig reaction. *Tetrahedron* **54**, 2161−2168 (1998).

(*11*) Wang, Z., Campagna, S., Yang, K., Xu, G., Pierce, M. E., Fortunak, J. M. & Confalone, P. N. A Practical Preparation of Terminal Alkynes from Aldehydes. *J. Org. Chem.* **65**, 1889–1891 (2000).

(*12*) Perryman, M. S., Harris, M. E., Foster, J. L., Joshi, A., Clarkson, G. J. & Fox, D. J. Trichloromethyl ketones: asymmetric transfer hydrogenation and subsequent Jocic-type reactions with amines. *Chem*. *Commun*. **49**, 10022–10024 (2013).

(*13*) Horner, J. H., Bagnol, L., Newcomb, M. Kinetics of Radical Heterolysis Reactions Forming Alkene Radical Cations. *J. Am. Chem. Soc.* **126**, 14979–14987 (2004).

14 Crimmins, M. T., King, B. W., Tabet, E. A. & Chaudhary, K. Asymmetric Aldol Additions: Use of Titanium Tetrachloride and (−)-Sparteine for the Soft Enolization of N-Acyl Oxazolidinones, Oxazolidinethiones, and Thiazolidinethiones. *J. Org. Chem.* **66**, 894–902 (2001).

(*15*) Sailer, M., Dubicki, K. I., Sorensen, J. L. The Synthesis of Medium-Chain-Length β-Hydroxy Esters via the Reformatsky Reaction. *Synthesis* **47**, 79–82 (2015).

16 Nicolaou, K. C., Schlawe, D., Kim, D. W., Longbottom, D. A., de Noronha, R. G., Lizos, D. E., Manam, R. R. & Faulkner, D. J. Total Synthesis of Halipeptins: Isolation of Halipeptin D and Synthesis of Oxazoline Halipeptin Analogues. *Chem. Eur. J.* **11**, 6197–6211 (2005)

(*17*) Kehne, H., Willins, L., Ort, O., Bauer, K., Bieringer, K. Pyridyl sulphonyl ureas as herbicides and plant growth regulators. US5529976 (1996).

(*18*) Yus, M., Ortiz, R., Huerta, F. F. Intramolecular carbolithiation promoted by a DTBB-catalysed chlorine–lithium. *Tetrahedron* **59**, 8525–8542 (2003).

(*19*) Crossley, S. W. M., Martinez, R. M., Guevara-Zuluaga, S. & Shenvi, R. A. Synthesis of the Privileged 8-Arylmenthol Class by Radical Arylation of Isopulegol. *Org. Lett.* **18**, 2620–2623 (2016).

(*20*) Perdew, J. P., Ernzerhof, M. & Burke, K. Rationale for mixing exact exchange with density functional approximations. *J. Chem. Phys.* **105**, 9982–9985 (1996).

(*21*) Adamo, C. & Barone, V. Toward reliable density functional methods without adjustable parameters: The PBE0 model. *J. Chem. Phy.* **110**, 6158–6170 (1999).

(*22*) Grimme, S., Antony, J., Ehrlich, S. & Krieg, H. A consistent and accurate ab initio parametrization of density functional dispersion correction (DFT-D) for the 94 elements H-Pu. *J. Chem. Phys.* ***132***, 154104 (2010).

(*23*) Grimme, S., Ehrlich, S. & Goerigk, L. Effect of the damping function in dispersion corrected density functional theory. *J. Comput. Chem.* **32**, 1456–1465 (2011).

(*24*) Weigend, F. & Ahlrichs, R. Balanced basis sets of split valence. triple zeta valence and quadruple zeta valence quality for H to Rn: Design and assessment of accuracy. *Phys. Chem. Chem. Phys.* **7**, 3297–3305 (2005).

(*25*) Grimme, S. Supramolecular binding thermodynamics by dispersion-corrected density functional theory. *Chem. Eur. J.* **18**, 9955–9964 (2012).

(*26*) Goerigk, L., Grimme, S., Efficient and accurate double-hybrid-meta-GGA density functionals - Evaluation with the extended GMTKN30 database for general main group thermos - chemistry, kinetics, and noncovalent interactions. *J. Chem. Theory Comput.* **7**, 291–309 (2011).

(*27*) TURBOMOLE V7.1 (2016), V7.2 (2017), a development of University of Karlsruhe and Forschungszentrum Karlsruhe GmbH, 1989–2007, TURBOMOLE GmbH, since 2007; available from *http://www.turbomole.com*.

(*28*) Neese, F. The ORCA Program System, *Wiley Interdisciplinary Reviews: Computational Molecular Science* **2,** 73–78 (2012).

(*29*) *APEX3* (2016), *SAINT* (2015) and *SADABS* (2015), Bruker AXS Inc., Madison, Wisconsin, USA; *SHELX* software: G. M. Sheldrick, *SHELXT* – Integrated space-group and crystal-structure determination *Acta Cryst.* **A71**, 3–8 (2015).
