# Supplementary material for: Remote C−H functionalization using radical translocating arylating groups
Source: Nat Commun. 2018 Jul 18;9:2808. doi: 10.1038/s41467-018-05193-6 (PMC6051993; doi:10.1038/s41467-018-05193-6)
Supplement: Supplementary file 2 — Supplementary Data 1 [file 41467_2018_5193_MOESM2_ESM.pdf]

Cartesian coordinates of all DFT-optimized structures

2n-PhRad

```
E(PBE0-D3/def2-TZVP) = -1285.872683203 (conv)
Lowest Freq. =    17.45 cm^-1
46
2n-PhRad (001/c1/pbe0-d3.def2-TZVP)
C    -2.8062755   -1.1065998    0.7114825
C    -3.2617673    0.0268660   -0.1958273
C    -1.3157466   -1.0231886    1.0006801
H    -3.0242119   -2.0652919    0.2237361
H    -3.3714960   -1.1002219    1.6480881
C    -0.4853008   -0.9687417   -0.2798984
H    -1.1064082   -0.1273542    1.5983665
H    -0.9988113   -1.8780778    1.6069215
C    -2.4413188    0.0600054   -1.4766511
H    -3.1400666    0.9819893    0.3303225
H    -4.3266998   -0.0734413   -0.4248151
C    -0.9548112    0.1765389   -1.1737472
H    -0.6862283   -1.8961167   -0.8286014
C     1.0180242   -0.9516696    0.0378011
H    -0.3739945    0.1998029   -2.0988158
H    -0.7709388    1.1300506   -0.6708685
H    -2.6292317   -0.8558757   -2.0512102
H    -2.7568363    0.8944947   -2.1094828
H     1.1673259   -1.1531332    1.1013642
O     1.5610888    0.3746054   -0.2131143
C     1.8831896   -1.9557341   -0.7418075
C     1.4571469   -3.3657560   -0.3353539
C     3.3470999   -1.7650668   -0.3494800
C     1.7334652   -1.7739473   -2.2483008
H     1.5574841   -3.5114884    0.7437572
H     2.0967875   -4.0996293   -0.8307295
H     0.4254408   -3.5893726   -0.6140582
H     0.7105050   -1.9623539   -2.5829313
H     2.3865006   -2.4743150   -2.7747162
H     2.0145328   -0.7627181   -2.5490061
H     3.4777538   -1.8310497    0.7337123
H     3.7280048   -0.7966271   -0.6759256
H     3.9593836   -2.5418036   -0.8136213
S     2.1030296    1.3003443    0.9378453
O     3.1601305    2.0517671    0.3488252
O     2.3030457    0.5338510    2.1252614
C     0.7513537    2.4004065    1.2013081
C     0.6039445    3.5397670    0.4107429
C    -0.4930299    4.3626714    0.6083981
H     1.3542437    3.7629044   -0.3387563
C    -1.4378261    4.0678894    1.5845365
H    -0.6111853    5.2489796   -0.0028760
C    -1.2961268    2.9375652    2.3891586
H    -2.2901915    4.7225941    1.7287557
C    -0.1997557    2.1571513    2.1512490
H    -2.0212221    2.6993297    3.1582819
```

2n-TS1Aa

```
E(PBE0-D3/def2-TZVP) = -1285.864402242 (conv)
Lowest Freq. =  -890.49 cm^-1
46
2n-TS1Aa (TS001A/c1/pbe0-d3.def2-TZVP)
C    -2.8535289   -1.3909160    0.6523276
C    -3.3149253   -0.1465925   -0.0913822
```

```
C    -1.3697791   -1.3239001    0.9774704
H    -3.0482886   -2.2754865    0.0324453
H    -3.4329285   -1.5210184    1.5704653
C    -0.5243162   -1.1146260   -0.2785603
H    -1.1760360   -0.5043773    1.6785368
H    -1.0493525   -2.2409943    1.4811196
C    -2.4811912    0.0836221   -1.3447201
H    -3.2164669    0.7211356    0.5711194
H    -4.3739876   -0.2257782   -0.3518216
C    -1.0028857    0.1042288   -1.0380167
H    -0.7032903   -1.9793764   -0.9326296
C     0.9752232   -1.0983522    0.0505181
H    -0.3872083    0.3401009   -1.9067810
H    -0.8127452    1.0661033   -0.3020623
H    -2.6839393   -0.7210251   -2.0663218
H    -2.7737094    1.0145789   -1.8396757
H     1.1151147   -1.4686997    1.0682146
O     1.4660472    0.2697288    0.0304378
C     1.8899583   -1.9204868   -0.8695482
C     1.5176232   -3.3949550   -0.7208743
C     3.3357830   -1.7409918   -0.4106826
C     1.7595138   -1.4921517   -2.3271202
H     1.5896627   -3.7171379    0.3216143
H     2.2061407   -4.0080904   -1.3066156
H     0.5070127   -3.6105859   -1.0739157
H     0.7505604   -1.6598802   -2.7120362
H     2.4482557   -2.0697249   -2.9483613
H     2.0053034   -0.4352630   -2.4481304
H     3.4497965   -2.0114503    0.6428747
H     3.6714470   -0.7106562   -0.5299870
H     3.9950244   -2.3857412   -0.9965249
S     1.7615563    1.0703066    1.3533730
O     3.0830093    1.5982119    1.2590145
O     1.3993193    0.2695330    2.4836485
C     0.6268949    2.4098285    1.1943110
C     0.9436889    3.6164416    1.8127850
C     0.0349673    4.6602709    1.7546487
H     1.8949703    3.7275182    2.3198062
C    -1.1701236    4.5087954    1.0805640
H     0.2728794    5.6028446    2.2326090
C    -1.4757987    3.3058692    0.4517386
H    -1.8722467    5.3340342    1.0359024
C    -0.5680245    2.2749507    0.5294389
H    -2.4089801    3.1901546   -0.0892159
```

2n-TS1Ab

```
E(PBE0-D3/def2-TZVP) = -1285.861154006 (conv)
Lowest Freq. =  -963.80 cm^-1
46
2n-TS1Ab (TS001A/c2/pbe0-d3.def2-TZVP)
C    -2.8130515   -1.8721880   -1.1013840
C    -3.5321642   -1.2291914    0.0707692
C    -1.3284487   -2.0689881   -0.8248611
H    -2.9299844   -1.2373399   -1.9883681
H    -3.2670282   -2.8362691   -1.3476724
C    -0.6301604   -0.7506987   -0.4694389
H    -1.2018729   -2.7820081   -0.0015596
H    -0.8665946   -2.5145736   -1.7055303
C    -2.8580117    0.0867217    0.4372188
H    -3.5018970   -1.9048351    0.9336542
```

```
H    -4.5874172   -1.0644980   -0.1655076
C    -1.3837258   -0.1169860    0.6883350
H    -0.7102777   -0.0878659   -1.3407678
C     0.8605160   -0.8680493   -0.1307518
H    -0.9429085    1.0017676    0.9287981
H    -1.1965897   -0.6569345    1.6226269
H    -2.9903823    0.7980456   -0.3875472
H    -3.3301007    0.5294576    1.3188439
H     0.9730086   -1.2879340    0.8727741
O     1.3272179    0.5135890   -0.0763851
C     1.8127319   -1.6295061   -1.0786394
C     1.6706751   -3.1401316   -0.8749020
C     3.2559285   -1.2807198   -0.7054670
C     1.5778582   -1.2447910   -2.5358373
H     1.8395255   -3.4052041    0.1724419
H     2.4310184   -3.6527992   -1.4687917
H     0.7034610   -3.5381470   -1.1715083
H     0.5896921   -1.5411317   -2.8930064
H     2.3190717   -1.7315553   -3.1742439
H     1.6799034   -0.1655677   -2.6704103
H     3.4477855   -1.4681576    0.3547290
H     3.4952285   -0.2384866   -0.9098731
H     3.9381950   -1.9078728   -1.2837725
S     1.9277272    1.1636237    1.2291857
O     3.2325265    1.6578537    0.9313272
O     1.7417224    0.2627918    2.3263433
C     0.8507277    2.5453707    1.3983951
C     1.3702051    3.8015759    1.6847435
C     0.4916589    4.8617626    1.8547795
H     2.4426864    3.9346256    1.7596947
C    -0.8778360    4.6701950    1.7325829
H     0.8811537    5.8476411    2.0780951
C    -1.3914309    3.4086161    1.4367455
H    -1.5535270    5.5084628    1.8628600
C    -0.5061442    2.3709031    1.2786898
H    -2.4606713    3.2594264    1.3325922
```

2n-TS1Ba

```
E(PBE0-D3/def2-TZVP) = -1285.856355867 (conv)
Lowest Freq. =  -979.97 cm^-1
46
2n-TS1Ba (TS001B/c1/pbe0-d3.def2-TZVP)
C    -2.4312743    0.1879947   -1.3213115
C    -3.0691296   -1.1081410   -1.8081286
C    -0.9299968    0.0632200   -1.1854400
H    -2.8544620    0.4416947   -0.3415555
H    -2.6796801    1.0131600   -1.9948838
C    -0.5135649   -1.1115686   -0.3198364
H    -0.4148334    0.1083484   -2.1461876
H    -0.5851770    1.0821560   -0.6006033
C    -2.6722709   -2.2657293   -0.9070843
H    -2.7389116   -1.3171677   -2.8328695
H    -4.1570226   -1.0000484   -1.8437334
C    -1.1589298   -2.4007627   -0.8339312
H    -0.9587771   -0.9257690    0.6669398
C     0.9923333   -1.2192587   -0.0227499
H    -0.8827737   -3.2398285   -0.1889735
H    -0.7861481   -2.6295307   -1.8368298
H    -3.0735086   -2.0994942    0.0997804
H    -3.1098862   -3.2000460   -1.2698924
```

H	1.0962734	-1.7913258	0.9033841
O	1.4713337	0.1304333	0.2467791
C	2.0084234	-1.8166551	-1.0212570
C	1.8496247	-3.3367184	-1.0975970
C	3.4139169	-1.5489236	-0.4726739
C	1.8978167	-1.2091642	-2.4153808
H	1.8750514	-3.7865810	-0.1013391
H	2.6849351	-3.7520973	-1.6661315
H	0.9326172	-3.6516347	-1.5901698
H	0.9567343	-1.4726057	-2.9025884
H	2.7094721	-1.5819761	-3.0451956
H	1.9779263	-0.1208569	-2.3782410
H	3.5124012	-1.9118419	0.5541937
H	3.6597437	-0.4882613	-0.4749512
H	4.1480155	-2.0756174	-1.0866187
S	1.3777423	0.7753827	1.6771784
O	2.6916665	1.1254240	2.1084143
O	0.5635529	-0.0536686	2.5146581
C	0.5184665	2.2587777	1.2724630
C	0.7424915	3.3958967	2.0425099
C	0.0270504	4.5482493	1.7593594
H	1.4762693	3.3701493	2.8395277
C	-0.8915367	4.5696640	0.7176741
H	0.1933086	5.4396156	2.3520868
C	-1.1035338	3.4325281	-0.0568446
H	-1.4419585	5.4787388	0.5015256
C	-0.3898713	2.2960560	0.2421939
H	-1.8127794	3.4494680	-0.8773410

2n-TS1Bb

E(PBE0-D3/def2-TZVP) = -1285.865358672 (conv)
Lowest Freq. = -1023.10 cm^-1
46
2n-TS1Bb (TS001B/c2/pbe0-d3.def2-TZVP)

C	-2.9175727	-0.6121965	0.3842856
C	-3.1931765	0.3460520	-0.7664300
C	-1.4461422	-0.7089342	0.7062250
H	-3.2788446	-1.6139991	0.1102328
H	-3.4808214	-0.3224561	1.2766220
C	-0.5559515	-0.9903916	-0.4904247
H	-1.1000274	0.3926082	1.1538051
H	-1.2397282	-1.3777525	1.5456200
C	-2.3199303	0.0243870	-1.9686385
H	-2.9837835	1.3719427	-0.4440490
H	-4.2518837	0.3114318	-1.0380578
C	-0.8435911	0.0235146	-1.5975201
H	-0.8377534	-1.9846459	-0.8658291
C	0.9056262	-1.0896958	-0.0518585
H	-0.2365837	-0.1802811	-2.4788083
H	-0.5565085	1.0192747	-1.2466482
H	-2.5967311	-0.9591460	-2.3693909
H	-2.4998587	0.7474574	-2.7690209
H	0.9258476	-1.4872688	0.9657922
O	1.4497203	0.2550985	0.0133486
C	1.8718396	-1.9657149	-0.8689995
C	1.3829701	-3.4115678	-0.7968406
C	3.2493224	-1.8906237	-0.2088651
C	1.9973164	-1.5361904	-2.3267164
H	1.2565253	-3.7378455	0.2393794
H	2.1174709	-4.0710587	-1.2644890

```
H     0.4353276   -3.5564926   -1.3197049
H     1.0848437   -1.7362717   -2.8916381
H     2.8053858   -2.0957246   -2.8045953
H     2.2337736   -0.4733925   -2.4068503
H     3.1965703   -2.1514186    0.8512225
H     3.6787549   -0.8909921   -0.2877510
H     3.9312567   -2.5898802   -0.6980680
S     1.8750326    0.9180009    1.3802883
O     3.0967259    1.6100785    1.1346019
O     1.7931486   -0.0588300    2.4225075
C     0.6059068    2.1176544    1.6228133
C     0.9626566    3.4317050    1.9029901
C    -0.0354905    4.3577297    2.1674161
H     2.0097189    3.7090502    1.9082536
C    -1.3697852    3.9777768    2.1488567
H     0.2324291    5.3839465    2.3878360
C    -1.7225291    2.6627279    1.8539105
H    -2.1435908    4.7082555    2.3578769
C    -0.7218017    1.7558374    1.5999445
H    -2.7660841    2.3682413    1.8273658
```

2n-INT1A

```
E(PBE0-D3/def2-TZVP) = -1285.898863725 (conv)
Lowest Freq. =     9.46 cm^-1
46
2n-INT1A (002A/c1/pbe0-d3.def2-TZVP)
C    -2.8047467   -0.8411262    0.6077526
C    -3.1160794    0.3302562   -0.3097525
C    -1.3211506   -0.9018903    0.9296387
H    -3.1131357   -1.7768531    0.1234230
H    -3.3833517   -0.7636128    1.5327544
C    -0.4659387   -1.0126377   -0.3398678
H    -1.0358326    0.0015682    1.4763283
C    -2.3095411    0.2358855   -1.6041682
H    -2.8607149    1.2637811    0.2032689
H    -4.1852642    0.3688426   -0.5368286
C    -0.8666528    0.0079707   -1.3453204
H    -0.6989598   -2.0040783   -0.7644434
C     1.0334766   -1.0201686   -0.0070726
H    -0.1262109    0.4172817   -2.0187099
H    -2.7187820   -0.5991809   -2.2012016
H    -2.4500026    1.1332876   -2.2128042
H     1.1644401   -1.2227267    1.0592379
O     1.5943061    0.2942639   -0.2601056
C     1.8946849   -2.0383368   -0.7731605
C     1.4599003   -3.4433837   -0.3589180
C     3.3570369   -1.8521621   -0.3723710
C     1.7541250   -1.8693142   -2.2819364
H     1.5391363   -3.5779868    0.7234454
H     2.1085531   -4.1827234   -0.8340267
H     0.4339521   -3.6706138   -0.6558875
H     0.7280944   -2.0369210   -2.6176094
H     2.3950568   -2.5883827   -2.7979940
H     2.0576495   -0.8676412   -2.5927575
H     3.4803076   -1.9108797    0.7121221
H     3.7436666   -0.8873902   -0.7020850
H     3.9681360   -2.6351406   -0.8274583
S     2.0404295    1.2638139    0.8977697
O     3.0192862    2.1076898    0.2987705
O     2.3358257    0.5086555    2.0761410
```

```
C     0.5909071    2.2160668    1.1997933
C     0.0433349    2.9604752    0.1630107
C    -1.0990226    3.7040470    0.3997951
H     0.5122572    2.9540174   -0.8127036
C    -1.6806990    3.7051541    1.6618082
H    -1.5384666    4.2850447   -0.4020960
C    -1.1196500    2.9655835    2.6915350
H    -2.5758555    4.2889197    1.8418336
C     0.0233199    2.2129256    2.4635976
H    -1.5712380    2.9719779    3.6762410
H     0.4772354    1.6216007    3.2485433
H    -1.1024040   -1.7524197    1.5832212
```

2n-INT1B

```
E(PBE0-D3/def2-TZVP) = -1285.899203295 (conv)
Lowest Freq. =    18.52 cm^-1
46
2n-INT1B (002B/c1/pbe0-d3.def2-TZVP)
C    -2.7209415   -0.5852996    0.8551333
C    -3.0823950    0.4070512   -0.2464058
C    -1.2631980   -0.8485138    0.9208777
H    -3.2550932   -1.5320298    0.6556340
H    -3.0892011   -0.2390089    1.8254921
C    -0.4665479   -1.0161540   -0.3275212
H    -0.8338925   -1.2011766    1.8534548
C    -2.4013791    0.0356235   -1.5531891
H    -2.7549078    1.4086094    0.0524295
H    -4.1680813    0.4473937   -0.3712096
C    -0.8900664    0.0087716   -1.3870629
H    -0.7135921   -2.0073622   -0.7478674
C     1.0382919   -1.0421562   -0.0212227
H    -0.4020606   -0.2077874   -2.3384711
H    -0.5538336    1.0011886   -1.0798877
H    -2.7581695   -0.9464735   -1.8897055
H    -2.6716156    0.7500262   -2.3360496
H     1.1771894   -1.3025420    1.0307408
O     1.5911232    0.2868026   -0.1985483
C     1.9092206   -2.0194040   -0.8291804
C     1.4921745   -3.4427451   -0.4615611
C     3.3676962   -1.8255647   -0.4156042
C     1.7790150   -1.8137287   -2.3338497
H     1.5774414   -3.6139136    0.6150323
H     2.1459452   -4.1582966   -0.9652238
H     0.4669690   -3.6692692   -0.7617628
H     0.7716033   -2.0418223   -2.6893026
H     2.4702046   -2.4778495   -2.8588027
H     2.0230493   -0.7869585   -2.6135979
H     3.4859697   -1.9121201    0.6677544
H     3.7433442   -0.8472337   -0.7166628
H     3.9907183   -2.5888847   -0.8875339
S     1.9857493    1.2015177    1.0254655
O     3.0378530    2.0273253    0.5351186
O     2.1606962    0.3904887    2.1905570
C     0.5563876    2.2030924    1.2512483
C     0.3111167    3.2358305    0.3559860
C    -0.8269974    4.0079610    0.5146987
H     1.0127936    3.4288744   -0.4459732
C    -1.6994951    3.7533802    1.5652534
H    -1.0300335    4.8158370   -0.1779144
C    -1.4358953    2.7287377    2.4618276
```

```
H    -2.5869109    4.3632015    1.6884575
C    -0.3043261    1.9427104    2.3060503
H    -2.1126817    2.5389138    3.2862523
H    -0.0732373    1.1429572    2.9966465
```

2n-TS2Aa

```
E(PBE0-D3/def2-TZVP) = -1285.880926802 (conv)
Lowest Freq. =  -399.40 cm^-1
46
2n-TS2Aa (TS002A/c1/pbe0-d3.def2-TZVP)
C    -2.4886598   -1.4129151   -1.1540211
C    -3.2125406   -1.5214591    0.1781524
C    -1.0149108   -1.7429987   -0.9904567
H    -2.5920176   -0.3942655   -1.5503800
H    -2.9465615   -2.0807712   -1.8886679
C    -0.3547418   -0.8041619    0.0155683
H    -0.8915572   -2.7760974   -0.6522411
C    -2.5766565   -0.6354011    1.2423992
H    -3.1866496   -2.5622675    0.5109549
H    -4.2676525   -1.2566803    0.0677027
C    -1.0875908   -0.7413657    1.3206203
H    -0.4619981    0.2118829   -0.3999881
C     1.1526268   -1.0705544    0.1692766
H    -0.6350889   -0.0581886    2.0339012
H    -2.8161999    0.4160281    1.0146471
H    -3.0151749   -0.8292581    2.2253363
H     1.4252905   -1.9220772   -0.4595835
O     1.4806089   -1.4401898    1.5287413
C     2.0796381    0.0979641   -0.1888612
C     1.8762953    0.4374579   -1.6637302
C     3.5280793   -0.3427532    0.0137272
C     1.7936584    1.3204775    0.6779010
H     2.0761042   -0.4294023   -2.2994551
H     2.5669118    1.2314186   -1.9565097
H     0.8641289    0.7886815   -1.8768823
H     0.7778413    1.6973791    0.5339984
H     2.4815630    2.1290388    0.4199786
H     1.9282835    1.0892163    1.7360760
H     3.7511752   -1.2354198   -0.5770524
H     3.7329758   -0.5712031    1.0596427
H     4.2083576    0.4506062   -0.3049152
S     0.9217023   -2.8069185    2.0866664
O     1.7514573   -3.1063420    3.2048134
O     0.7911499   -3.7332463    1.0031238
C    -0.7063256   -2.4086536    2.6825622
C    -0.7679027   -1.6896359    3.9127934
C    -1.8745548   -1.7912445    4.7162755
H     0.0592091   -1.0470441    4.1890759
C    -2.9086214   -2.6790447    4.4004485
H    -1.9280784   -1.2023296    5.6246255
C    -2.7936195   -3.4904771    3.2749718
H    -3.7701633   -2.7649878    5.0507157
C    -1.6954934   -3.3984124    2.4477032
H    -3.5608460   -4.2245955    3.0571938
H    -1.5724395   -4.0658054    1.6050765
H    -0.4935275   -1.6617280   -1.9490666
```

2n-TS2Ab

```
E(PBE0-D3/def2-TZVP) = -1285.883272554 (conv)
Lowest Freq. =  -333.80 cm^-1
```

46
2n-TS2Ab (TS002A/c2/pbe0-d3.def2-TZVP)
C   -1.1969965   2.1848682   -0.1548428
C   -2.5178950   1.4388571   -0.0985026
C   -0.0588578   1.3001095   -0.6443364
H   -0.9522308   2.5583927   0.8471707
H   -1.2786998   3.0624566   -0.8023470
C   0.0893939   0.0340563   0.2177545
H   -0.2348561   1.0125299   -1.6871773
C   -2.3774524   0.1867644   0.7705277
H   -2.8204486   1.1489228   -1.1111649
H   -3.3093927   2.0814582   0.2979326
C   -1.2490994   -0.6451671   0.2722740
H   0.3535595   0.3637038   1.2320144
C   1.1601123   -0.9575851   -0.2355396
H   -1.4720054   -1.2820717   -0.5822507
H   -2.1791799   0.4969584   1.8031697
H   -3.3068811   -0.3849595   0.7769302
H   0.8230982   -1.4714542   -1.1421440
O   1.2410792   -1.9560437   0.8170847
C   2.6099436   -0.4884809   -0.4672115
C   2.7283203   0.2194233   -1.8185759
C   3.5107240   -1.7251847   -0.5405337
C   3.0924041   0.4134367   0.6640860
H   2.3617859   -0.4212288   -2.6252343
H   3.7810164   0.4308022   -2.0201186
H   2.1913614   1.1639308   -1.8676857
H   2.5341933   1.3508886   0.7128910
H   4.1458649   0.6641482   0.5189354
H   3.0002839   -0.0909630   1.6286196
H   3.1430548   -2.4348917   -1.2866110
H   3.5699788   -2.2447255   0.4145060
H   4.5187169   -1.4213531   -0.8321388
S   0.1714852   -3.1077278   0.9059004
O   0.7494141   -4.0690781   1.7835932
O   -0.2433070   -3.4901362   -0.4097999
C   -1.1810903   -2.3222283   1.7335603
C   -0.9029567   -1.6887360   2.9696187
C   -1.9172149   -1.4863925   3.8769242
H   0.1048748   -1.3531813   3.1794946
C   -3.1999151   -1.9785198   3.6336469
H   -1.7064950   -0.9641817   4.8031668
C   -3.4505738   -2.7043290   2.4701983
H   -3.9874863   -1.8270969   4.3612891
C   -2.4542406   -2.9122546   1.5430854
H   -4.4333600   -3.1282821   2.2989352
H   -2.6311988   -3.4973781   0.6490859
H   0.8685983   1.8737490   -0.6225206

2n-TS2Ba

E(PBE0-D3/def2-TZVP) = -1285.881516641 (conv)
Lowest Freq. = -399.07 cm^-1
46
2n-TS2Ba (TS002B/c1/pbe0-d3.def2-TZVP)
C   -2.3757768   0.0868560   0.9926646
C   -2.9188684   0.4546354   -0.3819659
C   -0.9067020   -0.1867725   1.0216074
H   -2.8799734   -0.8316158   1.3354532
H   -2.6287621   0.8523843   1.7307414
C   -0.3395131   -0.9890112   -0.1202523

```
H    -0.5315287   -0.4991533    1.9941500
C    -2.4162982   -0.5009722   -1.4481061
H    -2.6086869    1.4703266   -0.6349285
H    -4.0118671    0.4584719   -0.3527825
C    -0.8958005   -0.5569065   -1.4727053
H    -0.6993859   -2.0182909    0.0556170
C     1.1766620   -1.0814581    0.0164797
H    -0.5760716   -1.2597735   -2.2422830
H    -0.4885959    0.4200672   -1.7484590
H    -2.8073439   -1.5085328   -1.2550352
H    -2.7924403   -0.2058300   -2.4315277
H     1.3710682   -1.4651137    1.0230901
O     1.7452006    0.2475238   -0.0386993
C     2.0007547   -1.9554593   -0.9459850
C     1.3237856   -3.3155170   -1.0860153
C     3.3753375   -2.1528172   -0.3022778
C     2.2117266   -1.3217859   -2.3203203
H     1.1515114   -3.7779973   -0.1097279
H     1.9650586   -3.9880243   -1.6604220
H     0.3658005   -3.2481684   -1.6060643
H     1.2921085   -1.2413928   -2.8980492
H     2.9076672   -1.9381672   -2.8947462
H     2.6451907   -0.3254194   -2.2279151
H     3.2894511   -2.6408747    0.6720550
H     3.8826282   -1.1973382   -0.1583095
H     4.0040722   -2.7777962   -0.9406024
S     1.6998693    1.1398733    1.2624429
O     2.6066712    2.2054668    0.9979423
O     1.8712920    0.3145233    2.4197629
C     0.0445673    1.7786510    1.2610957
C    -0.2997126    2.6031181    0.1536123
C    -1.2492418    3.5873589    0.2935721
H     0.1938372    2.4434976   -0.7966456
C    -1.8292051    3.8494683    1.5348216
H    -1.5284055    4.1825010   -0.5684517
C    -1.4077152    3.1305929    2.6548923
H    -2.5680366    4.6343933    1.6367227
C    -0.4573242    2.1441943    2.5411744
H    -1.8122137    3.3677416    3.6322281
H    -0.0947908    1.6125428    3.4121513
```

2n-TS2Bb

```
E(PBE0-D3/def2-TZVP) = -1285.873720036 (conv)
Lowest Freq. =  -383.25 cm^-1
46
2n-TS2Bb (TS002B/c2/pbe0-d3.def2-TZVP)
C    -2.2731686    0.3009741    0.1817790
C    -2.6485935    1.4155881   -0.7951654
C    -0.7937734    0.1291220    0.2630347
H    -2.7269128   -0.6273683   -0.1832981
H    -2.6902181    0.4861006    1.1739538
C    -0.0871531   -0.0530418   -1.0476325
H    -0.2904759    0.7698610    0.9812732
C    -2.0233372    1.1403697   -2.1514868
H    -2.2956414    2.3822635   -0.4173832
H    -3.7373992    1.4837998   -0.8729817
C    -0.5106729    1.0458571   -2.0368151
H    -0.4651322   -0.9885866   -1.4790277
C     1.4351108   -0.2603683   -0.8931196
H    -0.0662765    0.8446634   -3.0152941
```

```
H    -0.1345813    2.0160613   -1.7042783
H    -2.4243518    0.2022763   -2.5536907
H    -2.2866440    1.9267437   -2.8644389
H     1.7616554   -0.9372679   -1.6885340
O     1.6826164   -0.9474456    0.3624828
C     2.4349293    0.9174705   -0.8744697
C     2.0712736    1.9780262    0.1601052
C     2.5641390    1.5448188   -2.2644035
C     3.8153365    0.3469918   -0.5261354
H     1.1188554    2.4639541   -0.0583454
H     2.8408958    2.7536901    0.1794503
H     2.0139740    1.5432620    1.1600973
H     3.8469244   -0.0648286    0.4810028
H     4.5622980    1.1407281   -0.5988133
H     4.0988974   -0.4459706   -1.2237514
H     1.7059204    2.1468190   -2.5523890
H     2.7172540    0.7789808   -3.0298973
H     3.4392459    2.1984846   -2.2774833
S     0.9046203   -2.2682540    0.7189506
O     1.6968944   -2.8960696    1.7226220
O     0.5732689   -2.9637893   -0.4877964
C    -0.5917471   -1.6748551    1.4684891
C    -0.4664625   -1.0758999    2.7566800
C    -1.5336167   -1.0713645    3.6175850
H     0.4687679   -0.6015571    3.0283852
C    -2.7155270   -1.7473388    3.2933950
H    -1.4451572   -0.5683150    4.5735488
C    -2.7952517   -2.4590268    2.0995637
H    -3.5457021   -1.7537183    3.9887113
C    -1.7414655   -2.4659256    1.2117243
H    -3.6851261   -3.0346393    1.8719472
H    -1.7784899   -3.0512755    0.3018492
```

2n-INT2Aa

```
E(PBE0-D3/def2-TZVP) = -1285.906985522 (conv)
Lowest Freq. =    23.68 cm^-1
46
2n-INT2Aa (002_spiroA/c1/pbe0-d3.def2-TZVP)
C    -2.4214748   -1.7230014   -0.2046301
C    -2.8964248   -0.5516724    0.6421574
C    -0.9054181   -1.8436180   -0.1935055
H    -2.7544057   -1.5817344   -1.2410405
H    -2.8807386   -2.6518826    0.1442651
C    -0.2661432   -0.5396942   -0.6543264
H    -0.5890692   -2.6520760   -0.8598165
C    -2.2077434    0.7537831    0.2607619
H    -2.7148389   -0.7696059    1.6970191
H    -3.9782627   -0.4269065    0.5386950
C    -0.6810530    0.6710802    0.1896765
H    -0.7045279   -0.3223128   -1.6355444
C     1.2534007   -0.6585676   -0.8551771
H    -2.5604448    1.0578357   -0.7317856
H    -2.4976504    1.5517795    0.9498363
H     1.5940619   -1.6245874   -0.4725389
O     1.9499109    0.3567845   -0.0905372
C     1.7473635   -0.5184851   -2.3000845
C     1.1175384   -1.6309475   -3.1347239
C     3.2648736   -0.6913687   -2.3178981
C     1.3855967    0.8449561   -2.8816401
H     1.3650691   -2.6165866   -2.7312461
```

```
H     1.4998125   -1.5845236   -4.1568500
H     0.0296461   -1.5479623   -3.1873632
H     0.3044880    1.0001011   -2.9258987
H     1.7676854    0.9268739   -3.9018246
H     1.8251354    1.6509954   -2.2915966
H     3.5533799   -1.6556665   -1.8908281
H     3.7613860    0.0931162   -1.7464682
H     3.6344213   -0.6536804   -3.3453849
S     1.7286391    0.2877803    1.4734892
O     2.6162993    1.2522316    2.0289224
O     1.7926513   -1.0823363    1.8871092
C    -0.0260030    0.8647936    1.6095608
C     0.0031123    2.3308654    1.8909776
C    -0.5404491    2.8791515    3.0058473
H     0.4786147    2.9581901    1.1462504
C    -1.1403350    2.0800477    3.9966763
H    -0.5034630    3.9548297    3.1359634
C    -1.1514943    0.6838603    3.8332858
H    -1.5639696    2.5317631    4.8840208
C    -0.6301741    0.0868161    2.7294501
H    -1.5794214    0.0611413    4.6111395
H    -0.6041168   -0.9906991    2.6571491
H    -0.5390402   -2.1105386    0.8020039
H    -0.3364242    1.5496777   -0.3635475
```

2n-INT2Ab

```
E(PBE0-D3/def2-TZVP) = -1285.914165913 (conv)
Lowest Freq. =    39.87 cm^-1
46
2n-INT2Ab (002_spiroA/c2/pbe0-d3.def2-TZVP)
C    -2.5369677   -1.5561380   -0.9896035
C    -3.1825783   -0.8541737    0.1879598
C    -1.0606684   -1.8165390   -0.7383767
H    -2.6449854   -0.9309744   -1.8843944
H    -3.0418568   -2.5015426   -1.2068688
C    -0.2717429   -0.5527807   -0.3952934
H    -0.6355567   -2.2801213   -1.6274063
C    -2.4331021    0.4257220    0.5001868
H    -3.1662315   -1.5139535    1.0639692
H    -4.2332372   -0.6340312   -0.0200514
C    -0.9549258    0.1720582    0.7827617
H    -0.3012233    0.1097637   -1.2686610
C     1.1998669   -0.8414690   -0.0838523
H    -0.8801812   -0.4845138    1.6593183
H    -2.5180368    1.1161325   -0.3471013
H    -2.8767731    0.9366506    1.3583375
H     1.2689238   -1.4277048    0.8392122
O     1.8268926    0.4433103    0.1825358
C     2.0935069   -1.5235965   -1.1445644
C     1.8990483   -3.0416566   -1.1175113
C     3.5596797   -1.2722153   -0.7760552
C     1.8319620   -0.9675162   -2.5403408
H     2.0803726   -3.4368094   -0.1143960
H     2.6247607   -3.5059268   -1.7893765
H     0.9104797   -3.3668137   -1.4331945
H     0.8272312   -1.2020262   -2.8989525
H     2.5430408   -1.3947961   -3.2513277
H     1.9579341    0.1177269   -2.5569291
H     3.7632727   -1.5707664    0.2556379
H     3.8345763   -0.2240687   -0.8808106
```

```
H     4.2008736   -1.8651985   -1.4322366
S     1.4498514    1.1434107    1.5449854
O     2.2384320    2.3258848    1.6019131
O     1.5014066    0.1826047    2.6069339
C    -0.3037284    1.5279323    1.2042150
C    -0.3386905    2.5592011    0.1291570
C    -0.8047074    3.8154858    0.3479380
H     0.0502180    2.2824085   -0.8423517
C    -1.2927944    4.2138020    1.6058834
H    -0.7987618    4.5286470   -0.4688427
C    -1.3169993    3.2834170    2.6620167
H    -1.6479135    5.2243355    1.7593526
C    -0.8723312    2.0120456    2.4960263
H    -1.7073715    3.5843306    3.6278504
H    -0.8900316    1.3013531    3.3139738
H    -0.9509328   -2.5408904    0.0783342
```

2n-INT2Ba

```
E(PBE0-D3/def2-TZVP) = -1285.909118767 (conv)
Lowest Freq. =    41.41 cm^-1
46
2n-INT2Ba (002_spiroB/c1/pbe0-d3.def2-TZVP)
C    -2.4169087    0.0205353    0.9619434
C    -2.9608214    0.4163309   -0.4018711
C    -0.8899576   -0.0461611    1.0246607
H    -2.7986475   -0.9754269    1.2156983
H    -2.7838416    0.7009664    1.7344786
C    -0.3378153   -0.9117613   -0.1294079
H    -0.6244965   -0.5696123    1.9497758
C    -2.4163442   -0.4930525   -1.4887838
H    -2.6961443    1.4541889   -0.6201449
H    -4.0535244    0.3714629   -0.3836147
C    -0.8938035   -0.5334475   -1.4990165
H    -0.7275769   -1.9156131    0.0878711
C     1.1760289   -1.0470169    0.0066176
H    -0.5729286   -1.2733167   -2.2329806
H    -0.4895748    0.4226796   -1.8375573
H    -2.7922824   -1.5113678   -1.3252834
H    -2.7846088   -0.1818596   -2.4702396
H     1.3564796   -1.4757929    0.9982021
O     1.7771925    0.2738375    0.0151003
C     1.9906929   -1.9002303   -0.9797704
C     1.3012569   -3.2506572   -1.1521560
C     3.3637685   -2.1297641   -0.3430968
C     2.2083681   -1.2283720   -2.3346911
H     1.1355974   -3.7376151   -0.1867530
H     1.9322289   -3.9118185   -1.7504921
H     0.3384401   -3.1644054   -1.6595420
H     1.2886259   -1.1064161   -2.9044279
H     2.8870447   -1.8414902   -2.9328146
H     2.6658453   -0.2458948   -2.2119216
H     3.2734644   -2.6466630    0.6158485
H     3.8798485   -1.1840619   -0.1701693
H     3.9858025   -2.7412493   -1.0007713
S     1.5464822    1.1139821    1.3368723
O     2.2563388    2.3316940    1.1441098
O     1.8140023    0.2902596    2.4786242
C    -0.2702802    1.3834229    1.2301842
C    -0.4815021    2.3812747    0.1436539
C    -0.9817417    3.6221755    0.3819696
```

```
H    -0.1623235    2.1136260   -0.8532871
C    -1.3474538    4.0383817    1.6730156
H    -1.0948647    4.3088397   -0.4495396
C    -1.1785012    3.1501532    2.7519740
H    -1.7401756    5.0329973    1.8379901
C    -0.6796895    1.9017197    2.5699299
H    -1.4565729    3.4657268    3.7514264
H    -0.5451267    1.2288117    3.4083865
```

2n-INT2Bb

```
E(PBE0-D3/def2-TZVP) = -1285.904893825 (conv)
Lowest Freq. =    40.27 cm^-1
46
2n-INT2Bb (002_spiroB/c2/pbe0-d3.def2-TZVP)
C    -2.1275122    0.8579397   -0.3575888
C    -2.8986556    0.0618715   -1.3985779
C    -0.6279207    0.6772675   -0.5547732
H    -2.3998608    0.5312575    0.6527299
H    -2.3852344    1.9180111   -0.4218800
C    -0.2534963   -0.8106099   -0.4091585
C    -2.4804694   -1.3990984   -1.4030772
H    -2.7083295    0.4953498   -2.3876656
H    -3.9741577    0.1499499   -1.2215681
C    -0.9708621   -1.5658557   -1.5264747
H    -0.6765557   -1.1710472    0.5377205
C     1.2706928   -0.9610757   -0.2716275
H    -0.7322803   -2.6282933   -1.4937723
H    -0.6293343   -1.1943021   -2.4988500
H    -2.8096111   -1.8713358   -0.4690531
H    -2.9808523   -1.9354534   -2.2141172
H     1.5152405   -0.7620657    0.7770292
O     1.8955513    0.0895047   -1.0462736
C     1.9982656   -2.2754465   -0.5895855
C     1.3555969   -3.3978804    0.2221762
C     3.4455214   -2.1134624   -0.1163668
C     2.0248029   -2.6101651   -2.0794190
H     1.3714448   -3.1654524    1.2910611
H     1.9180608   -4.3232761    0.0792244
H     0.3216563   -3.5916341   -0.0655452
H     1.0481920   -2.8833592   -2.4744869
H     2.6962962   -3.4560788   -2.2458269
H     2.4045995   -1.7656862   -2.6571287
H     3.4907669   -1.8570247    0.9451391
H     3.9585852   -1.3274084   -0.6711464
H     3.9904423   -3.0485724   -0.2645377
S     1.9598280    1.4786445   -0.2769604
O     2.2173809    2.4443207   -1.2951718
O     2.8350661    1.3608002    0.8472689
C     0.1839017    1.6492163    0.3390528
C    -0.1742729    3.0670432    0.0542340
C    -0.4367559    3.9664605    1.0362509
H    -0.1813292    3.3653317   -0.9867902
C    -0.4220642    3.5945310    2.3931104
H    -0.6713566    4.9896373    0.7651909
C    -0.1551507    2.2573899    2.7342451
H    -0.6275687    4.3256884    3.1639657
C     0.1229068    1.3232497    1.7876274
H    -0.1764686    1.9597448    3.7765620
H     0.3145802    0.3019864    2.0897340
H    -0.3942064    0.9649390   -1.5876369
```

2n-TS3Aa

```
E(PBE0-D3/def2-TZVP) = -1285.899083150 (conv)
Lowest Freq. =  -273.98 cm^-1
46
2n-TS3Aa (TS003A/c1/pbe0-d3.def2-TZVP)
C    -2.5086599   -1.5835349   -0.3720377
C    -3.0303181   -0.4301318    0.4705111
C    -0.9893985   -1.6669573   -0.3437304
H    -2.8343536   -1.4418342   -1.4103974
H    -2.9420197   -2.5303943   -0.0384238
C    -0.3428864   -0.3334384   -0.7164689
H    -0.6541171   -2.4385800   -1.0391350
C    -2.3849035    0.8821031    0.0560726
H    -2.8296714   -0.6211616    1.5296462
H    -4.1166350   -0.3516359    0.3724909
C    -0.8505957    0.8506288    0.1288722
H    -0.7085129   -0.0915103   -1.7222911
C     1.1889381   -0.4461650   -0.8068266
H    -2.6647092    1.1037696   -0.9807815
H    -2.7610163    1.7059682    0.6680319
H     1.5305750   -1.2690439   -0.1697792
O     1.8173207    0.7599753   -0.3236177
C     1.7706110   -0.6613800   -2.2168869
C     1.1544128   -1.9009963   -2.8574798
C     3.2771801   -0.8786968   -2.0834017
C     1.5129152    0.5523061   -3.1065491
H     1.3070963   -2.7909931   -2.2412870
H     1.6315450   -2.0824189   -3.8233957
H     0.0837921   -1.7843666   -3.0392512
H     0.4455780    0.7302504   -3.2603086
H     1.9602089    0.3881271   -4.0897178
H     1.9527021    1.4544606   -2.6800611
H     3.4932293   -1.7451766   -1.4525727
H     3.7660405   -0.0096720   -1.6425330
H     3.7190389   -1.0580769   -3.0664498
S     1.8217554    0.9532033    1.2650317
O     2.4332179    2.2327741    1.4712955
O     2.3748188   -0.2263653    1.8772560
C    -0.4078202    1.0190001    1.5888937
C    -0.5492696    2.3626890    2.0828758
C    -0.6308415    2.6386591    3.4215790
H    -0.5333484    3.1724392    1.3621729
C    -0.6512416    1.6016485    4.3571960
H    -0.6866958    3.6686150    3.7536173
C    -0.6193762    0.2796716    3.9140797
H    -0.7079664    1.8215173    5.4160189
C    -0.5430365   -0.0156699    2.5754073
H    -0.6613546   -0.5301951    4.6328985
H    -0.4987043   -1.0497793    2.2711120
H    -0.6440899   -1.9935466    0.6399846
H    -0.4894337    1.7539147   -0.3716600
```

2n-TS3Ab

```
E(PBE0-D3/def2-TZVP) = -1285.901521818 (conv)
Lowest Freq. =  -277.16 cm^-1
46
2n-TS3Ab (TS003A/c2/pbe0-d3.def2-TZVP)
C    -2.5289138   -1.7039610   -1.0332850
C    -3.2246079   -1.0973076    0.1655295
```

```
C    -1.0374836   -1.8345579   -0.7827914
H    -2.6983889   -1.0672951   -1.9102582
H    -2.9432324   -2.6866665   -1.2752265
C    -0.3357330   -0.5237198   -0.4179315
H    -0.5740434   -2.2470112   -1.6765976
C    -2.5981191    0.2432679    0.4828198
H    -3.1271133   -1.7676667    1.0279818
H    -4.2952665   -0.9751400   -0.0206294
C    -1.0856625    0.1645653    0.7494233
H    -0.3771016    0.1343671   -1.2938591
C     1.1450142   -0.7723333   -0.0871428
H    -0.9354039   -0.4555990    1.6429489
H    -2.7649319    0.9303602   -0.3555892
H    -3.0777930    0.7026660    1.3508919
H     1.2090037   -1.3436411    0.8458644
O     1.7694950    0.5115981    0.1502902
C     2.0618991   -1.4744974   -1.1231170
C     1.8948708   -2.9962443   -1.0761981
C     3.5194327   -1.2017139   -0.7309446
C     1.8260688   -0.9412880   -2.5324806
H     2.0318436   -3.3687505   -0.0574219
H     2.6658340   -3.4562134   -1.6988322
H     0.9337481   -3.3524692   -1.4379783
H     0.8301381   -1.1840100   -2.9096045
H     2.5533994   -1.3758063   -3.2224905
H     1.9482545    0.1440642   -2.5613079
H     3.7050981   -1.4779323    0.3103815
H     3.7881149   -0.1538875   -0.8517231
H     4.1779673   -1.8023871   -1.3624984
S     1.5167513    1.1814316    1.5784545
O     2.3388773    2.3554020    1.5859116
O     1.6637619    0.1799320    2.6080817
C    -0.6834431    1.5920407    1.1170535
C    -0.5301915    2.5751127    0.0980967
C    -0.5766099    3.9165237    0.3982827
H    -0.3163226    2.2572445   -0.9143452
C    -0.8513735    4.3467885    1.6943513
H    -0.4045652    4.6450497   -0.3853762
C    -1.1212075    3.4059068    2.6899037
H    -0.8746314    5.4047793    1.9238284
C    -1.0839832    2.0639611    2.4057663
H    -1.3711842    3.7357733    3.6914593
H    -1.2903308    1.3320797    3.1792120
H    -0.8719351   -2.5628150    0.0210962
```

2n-TS3Ba

```
E(PBE0-D3/def2-TZVP) = -1285.899233573 (conv)
Lowest Freq. =  -284.40 cm^-1
46
2n-TS3Ba (TS003B/c1/pbe0-d3.def2-TZVP)
C    -2.5121141   -0.2289415    0.9089419
C    -3.0678017    0.1983107   -0.4391393
C    -0.9850847   -0.0804423    0.9925992
H    -2.7564611   -1.2841641    1.0789818
H    -2.9798972    0.3397633    1.7167716
C    -0.3412734   -0.8797505   -0.1644018
H    -0.6606096   -0.5739657    1.9158023
C    -2.4158991   -0.5845585   -1.5673191
H    -2.9060080    1.2712420   -0.5814081
H    -4.1507145    0.0464948   -0.4552114
```

```
C    -0.8923940   -0.5170888   -1.5401658
H    -0.6805444   -1.9065749    0.0294441
C     1.1753838   -0.9603128   -0.0061540
H    -0.5091091   -1.2241400   -2.2763126
H    -0.5371466    0.4638588   -1.8609463
H    -2.7152747   -1.6369273   -1.4811110
H    -2.7844490   -0.2388294   -2.5370405
H     1.3514221   -1.3537214    1.0012431
O     1.7625419    0.3600750   -0.0475505
C     2.0119841   -1.8436066   -0.9524059
C     1.3350432   -3.2024061   -1.1056631
C     3.3723147   -2.0486820   -0.2808388
C     2.2563341   -1.2088647   -2.3208489
H     1.1383690   -3.6609623   -0.1321321
H     1.9895036   -3.8780854   -1.6611681
H     0.3902470   -3.1375838   -1.6492733
H     1.3481698   -1.1049995   -2.9127553
H     2.9481955   -1.8366902   -2.8879177
H     2.7080352   -0.2218319   -2.2160927
H     3.2644875   -2.5455612    0.6867980
H     3.8765915   -1.0951600   -0.1159962
H     4.0141606   -2.6683754   -0.9113090
S     1.6027784    1.2156547    1.2990893
O     2.2741760    2.4517655    1.0299546
O     1.9935290    0.4063155    2.4274267
C    -0.6510837    1.4020984    1.2086617
C    -0.7436090    2.3863794    0.1741307
C    -0.8782805    3.7214801    0.4718622
H    -0.6236483    2.0876122   -0.8558327
C    -1.0101157    4.1532978    1.7895184
H    -0.8853453    4.4451412   -0.3347374
C    -1.0333896    3.2102945    2.8194035
H    -1.1074942    5.2084842    2.0119564
C    -0.9008905    1.8747074    2.5414339
H    -1.1677949    3.5329426    3.8452662
H    -0.9168344    1.1463080    3.3444461
```

2n-TS3Bb

```
E(PBE0-D3/def2-TZVP) = -1285.900149534 (conv)
Lowest Freq. =  -276.65 cm^-1
46
2n-TS3Bb (TS003B/c2/pbe0-d3.def2-TZVP)
C    -2.5258548    0.9113013   -0.1909890
C    -3.2103512    0.1145298   -1.2884803
C    -1.0049151    0.8387570   -0.3331938
H    -2.8120481    0.5266484    0.7951898
H    -2.8455740    1.9562951   -0.2211797
C    -0.5373237   -0.6283072   -0.2429729
C    -2.7063161   -1.3176477   -1.3210334
H    -3.0067461    0.5930715   -2.2538613
H    -4.2951202    0.1359444   -1.1515579
C    -1.1869998   -1.3897731   -1.3967015
H    -0.9380296   -1.0535877    0.6871632
C     0.9909148   -0.7072733   -0.1136169
H    -0.8878908   -2.4368415   -1.3801275
H    -0.8356025   -0.9758097   -2.3484354
H    -3.0380038   -1.8366305   -0.4129373
H    -3.1456816   -1.8579752   -2.1641615
H     1.2331464   -0.4210181    0.9151386
O     1.5789973    0.2941835   -0.9746378
```

C	1.7521990	-2.0260135	-0.3308067
C	1.0981059	-3.1181030	0.5118982
C	3.1767262	-1.8091749	0.1871104
C	1.8470329	-2.4410142	-1.7981578
H	1.0555714	-2.8275693	1.5658551
H	1.6885180	-4.0350181	0.4484451
H	0.0844702	-3.3541848	0.1849372
H	0.8951347	-2.7616897	-2.2173153
H	2.5452727	-3.2767004	-1.8897918
H	2.2289067	-1.6197362	-2.4066643
H	3.1768626	-1.5151942	1.2397365
H	3.6880956	-1.0274887	-0.3757445
H	3.7519488	-2.7329162	0.0908609
S	1.6896985	1.7226719	-0.2459408
O	1.8292663	2.6703066	-1.3128168
O	2.6816469	1.6522638	0.7911929
C	-0.3453223	1.8193911	0.6284240
C	-0.5911332	3.2198528	0.3796843
C	-0.4476280	4.1548374	1.3683029
H	-0.8101037	3.5248019	-0.6370111
C	-0.1512687	3.7650461	2.6790285
H	-0.5767570	5.2050464	1.1344746
C	-0.0568502	2.4067046	2.9876060
H	-0.0307785	4.5101646	3.4553612
C	-0.1935777	1.4549297	2.0100381
H	0.1069642	2.0990013	4.0137811
H	-0.1586468	0.4065356	2.2784627
H	-0.7609557	1.1873824	-1.3445563

2o-PhRad

E(PBE0-D3/def2-TZVP) = -1287.067723955 (conv)
Lowest Freq. =   12.63 cm^-1
48
2o-PhRad (011/c2/pbe0-d3.def2-TZVP)

C	-0.9902818	-0.5142136	-0.9502745
C	-3.3064139	0.2689905	-0.3149725
C	-1.8733885	0.0612428	0.1442288
H	-0.9757265	0.1663869	-1.8068940
C	0.4332354	-0.7884493	-0.5101243
H	-1.4661976	1.0182820	0.4842611
H	-1.8582540	-0.6034096	1.0155136
H	-3.3139355	0.9277580	-1.1924712
H	0.4251848	-1.3877503	0.4045639
O	1.0328180	0.4988095	-0.1745312
C	1.3421048	-1.4797437	-1.5373723
C	0.7706093	-2.8650218	-1.8371207
C	2.7338749	-1.6528207	-0.9335800
C	1.4503431	-0.6712038	-2.8264835
H	0.6234466	-3.4422107	-0.9197060
H	1.4664844	-3.4200718	-2.4703792
H	-0.1830651	-2.8130743	-2.3646844
H	0.4916396	-0.6014748	-3.3442745
H	2.1589345	-1.1494931	-3.5071598
H	1.8063322	0.3410056	-2.6250821
H	2.6876579	-2.1759271	0.0259396
H	3.2241277	-0.6922801	-0.7736904
H	3.3624509	-2.2409620	-1.6062865
S	1.3065831	0.9447082	1.3100653
O	0.8855508	-0.0845257	2.2103094
O	0.8116019	2.2734202	1.4337410

```
C     3.0667197    1.0089781    1.3452826
C     3.7920741    0.1112666    2.1242860
C     5.1772192    0.1848814    2.1288473
H     3.2582495   -0.6250678    2.7131516
C     5.8413743    1.1353561    1.3652952
H     5.7451487   -0.5086578    2.7368210
C     5.1248748    2.0424311    0.5815780
H     6.9248113    1.1796700    1.3768145
C     3.7650721    1.9318448    0.6166456
H     5.6304844    2.7915996   -0.0160476
H    -1.4238739   -1.4552685   -1.3005566
H    -3.7275230   -0.6870353   -0.6513067
C    -4.1984422    0.8603078    0.7626990
H    -4.1924186    0.2021165    1.6404248
H    -3.7739452    1.8135788    1.1000644
C    -5.6318140    1.0795377    0.3085782
H    -5.6343882    1.7371012   -0.5685116
H    -6.0531725    0.1252654   -0.0285764
C    -6.5140299    1.6724340    1.3932903
H    -6.1308858    2.6414552    1.7247858
H    -6.5530707    1.0187016    2.2691166
H    -7.5381811    1.8215317    1.0437824
```

2o-TS1(1,8)a

```
E(PBE0-D3/def2-TZVP) = -1287.054539856 (conv)
Lowest Freq. =  -931.44 cm^-1
48
2o-TS1(1,8)a (TS005A/c2/pbe0-d3.def2-TZVP)
C     0.3421544    0.1371297    2.1678095
C     0.9509391    1.5270097    0.0914720
C    -0.0651814    1.2326172    1.1745973
H     0.0310919    0.4226582    3.1743301
C    -0.1857457   -1.2707319    1.9069943
H    -0.2108102    2.1617386    1.7401381
H    -1.0338932    1.0135083    0.7193892
H     1.9762917    1.3585472    0.4336761
H     0.5325442   -1.9925645    2.3074166
O    -0.2743550   -1.4822493    0.4714783
C    -1.5549268   -1.6387977    2.5017663
C    -1.4509304   -1.5908900    4.0252987
C    -1.8941986   -3.0698774    2.0879567
C    -2.6599211   -0.6982009    2.0332389
H    -0.6382216   -2.2261943    4.3884874
H    -2.3806333   -1.9565006    4.4670208
H    -1.2871752   -0.5792457    4.4014002
H    -2.4938601    0.3256494    2.3756695
H    -3.6207989   -1.0285443    2.4355052
H    -2.7382202   -0.6918764    0.9440777
H    -1.1088927   -3.7661165    2.3940827
H    -2.0111323   -3.1581539    1.0080227
H    -2.8276281   -3.3822358    2.5620332
S     0.9351972   -2.1889237   -0.2565533
O     0.7230050   -3.6016592   -0.2885409
O     2.1590055   -1.6838966    0.2881592
C     0.6474908   -1.5232156   -1.8558768
C     0.4526620   -2.3756667   -2.9348481
C     0.2297158   -1.8246362   -4.1881702
H     0.4700007   -3.4474384   -2.7777332
C     0.1896692   -0.4473071   -4.3515365
H     0.0785759   -2.4757981   -5.0405314
```

C	0.3821089	0.4014395	-3.2624805
H	0.0057498	-0.0254745	-5.3336268
C	0.6165895	-0.1585350	-2.0300946
H	0.3451952	1.4769170	-3.3956880
H	1.4328130	0.0853358	2.1924232
H	0.8324942	0.7066947	-0.8123004
C	0.7961778	2.8669215	-0.5871199
H	-0.1442715	2.8879570	-1.1532406
H	0.6922419	3.6474042	0.1798810
C	1.9596210	3.2231682	-1.4991851
H	2.8719870	3.2914581	-0.8968008
H	2.1343564	2.4066248	-2.2070647
C	1.7435626	4.5228120	-2.2534772
H	1.6039530	5.3605339	-1.5645768
H	0.8523735	4.4686237	-2.8857065
H	2.5932294	4.7599815	-2.8971726

2o-TS1(1,8)b

```
E(PBE0-D3/def2-TZVP) = -1287.055189953 (conv)
Lowest Freq. =  -887.89 cm^-1
48
2o-TS1(1,8)b (TS005B/c1/pbe0-d3.def2-TZVP)
C    -0.1008291    0.3673116    1.8027325
C     0.3829993    1.7602032   -0.3453186
C    -0.5406575    1.4587945    0.8195564
H    -0.4120451    0.6486890    2.8103572
C    -0.6111744   -1.0482312    1.5480310
H    -0.6377106    2.3927010    1.3885306
H    -1.5385072    1.2488416    0.4306255
H     0.2966498    0.8878141   -1.2011694
H     0.1175350   -1.7567405    1.9530187
O    -0.6912536   -1.2686822    0.1117260
C    -1.9737557   -1.4362463    2.1460533
C    -1.8678390   -1.3785649    3.6692552
C    -2.2881666   -2.8754986    1.7408736
C    -3.0986430   -0.5203507    1.6759477
H    -1.0427026   -1.9966117    4.0342015
H    -2.7896862   -1.7593568    4.1145772
H    -1.7225498   -0.3620387    4.0397789
H    -2.9594393    0.5056656    2.0235877
H    -4.0528648   -0.8755696    2.0728912
H    -3.1727989   -0.5120721    0.5864784
H    -1.4886673   -3.5559667    2.0454306
H    -2.4095451   -2.9717702    0.6621243
H    -3.2131104   -3.2022872    2.2217602
S     0.5035145   -2.0244439   -0.5890352
O     0.2537767   -3.4312592   -0.5996977
O     1.7357829   -1.5474628   -0.0349393
C     0.2699911   -1.3809386   -2.2052135
C     0.1664263   -2.2464049   -3.2867803
C     0.0263714   -1.7091557   -4.5580523
H     0.1913777   -3.3165886   -3.1198340
C    -0.0168584   -0.3336064   -4.7392075
H    -0.0539985   -2.3702143   -5.4124176
C     0.0803028    0.5271442   -3.6473113
H    -0.1296803    0.0752880   -5.7374081
C     0.2258898   -0.0196320   -2.3960609
H     0.0439668    1.6022250   -3.7880447
H     0.9887256    0.3295663    1.8310055
H    -0.0041487    2.6224196   -0.9001663
C     1.8533749    1.9253365   -0.0525625
H     1.9769560    2.6523649    0.7642058
H     2.2700845    0.9806646    0.3089203
C     2.6558349    2.3834663   -1.2594641
H     2.5234808    1.6576519   -2.0689144
H     2.2494158    3.3328212   -1.6280328
C     4.1348100    2.5398314   -0.9546902
H     4.5650622    1.5927721   -0.6187831
H     4.2992255    3.2765355   -0.1631869
H     4.6939335    2.8663374   -1.8342824
```

2o-TS1(1,7)a

```
E(PBE0-D3/def2-TZVP) = -1287.061856220 (conv)
Lowest Freq. =  -949.17 cm^-1
48
2o-TS1(1,7)a (TS004B/c3/pbe0-d3.def2-TZVP)
C    -0.0265505   -0.9614871   -1.0550254
C    -2.2755384   -0.0907761   -0.2313620
C    -0.8653041   -0.4995797    0.1111363
H     0.0331274   -0.1753759   -1.8141199
C     1.3739022   -1.3925265   -0.6670744
H    -0.3376155    0.5048664    0.5839557
H    -0.8269637   -1.2048919    0.9467222
H    -2.2507733    0.7786828   -0.9006841
H     1.3326856   -1.9921500    0.2458858
O     2.1366799   -0.1930354   -0.3634502
C     2.1656776   -2.1802272   -1.7211613
C     1.4462314   -3.5017409   -1.9872172
C     3.5516629   -2.4865539   -1.1566772
C     2.3029657   -1.3900267   -3.0184511
H     1.2449772   -4.0403510   -1.0567081
H     2.0737023   -4.1423891   -2.6107738
H     0.4998061   -3.3611554   -2.5131204
H     1.3330445   -1.2102182   -3.4881802
H     2.9161706   -1.9464255   -3.7313688
H     2.7840425   -0.4265342   -2.8403003
H     3.4794132   -3.0385316   -0.2152021
H     4.1200943   -1.5750953   -0.9690757
H     4.1149680   -3.0996332   -1.8639307
S     2.5323445    0.2065971    1.1108647
O     3.9280338    0.4986767    1.1131817
O     1.9980178   -0.7626223    2.0177291
C     1.6557031    1.7253586    1.2794985
C     2.3356612    2.8786565    1.6497391
C     1.6212387    4.0595262    1.7923477
H     3.4058163    2.8380150    1.8122030
C     0.2540730    4.0877503    1.5576469
H     2.1391259    4.9657783    2.0821764
C    -0.4216418    2.9287526    1.1788901
H    -0.2936114    5.0176110    1.6651368
C     0.2995681    1.7666194    1.0549636
H    -1.4888615    2.9542129    0.9877194
H    -0.5328126   -1.8114598   -1.5265543
H    -2.7541541   -0.8939055   -0.8096929
C    -3.1263289    0.2196246    0.9890010
H    -3.2112448   -0.6828440    1.6060527
H    -2.6134000    0.9579537    1.6162180
C    -4.5152370    0.7284523    0.6433231
H    -4.4247175    1.6339237    0.0316948
H    -5.0240404   -0.0099620    0.0130117
C    -5.3596953    1.0229368    1.8705626
H    -4.8841959    1.7775771    2.5033159
H    -5.4956960    0.1246031    2.4787865
H    -6.3503509    1.3933236    1.5983668
```

2o-TS1(1,7)b

```
E(PBE0-D3/def2-TZVP) = -1287.062709652 (conv)
Lowest Freq. =  -949.51 cm^-1
48
2o-TS1(1,7)b (TS004A/c3/pbe0-d3.def2-TZVP)
C    -0.4637553   -0.4707349   -1.7017679
C    -1.6316213   -0.9272039    0.5499908
C    -1.3381379    0.0332531   -0.5756154
H    -0.3959526    0.2871388   -2.4862402
C     0.9404738   -0.8651724   -1.2859074
H    -2.2556608    0.4807029   -0.9702125
H    -0.7845812    1.0077626   -0.0658810
H    -2.1025485   -1.8299744    0.1338522
H     0.9133055   -1.4591951   -0.3707945
O     1.6505478    0.3670283   -0.9802849
C     1.7672704   -1.6389916   -2.3235630
C     1.0954255   -2.9885023   -2.5727045
C     3.1589861   -1.8897158   -1.7457959
C     1.8887843   -0.8651366   -3.6320653
H     0.9291080   -3.5289571   -1.6364220
H     1.7379122   -3.6076478   -3.2026844
H     0.1363189   -2.8875998   -3.0844557
H     0.9189723   -0.7299592   -4.1164969
H     2.5314527   -1.4091681   -4.3283468
H     2.3312811    0.1186923   -3.4644782
H     3.0995135   -2.4200240   -0.7912067
H     3.7004775   -0.9578033   -1.5794214
H     3.7434610   -2.5023997   -2.4360226
S     2.1436770    0.7199761    0.4746715
O     3.5184663    1.0885480    0.3886252
O     1.7372323   -0.3192002    1.3724129
C     1.2052734    2.1751434    0.7964647
C     1.8373625    3.2986051    1.3156335
C     1.0743319    4.4187926    1.6112138
H     2.9091169    3.2842377    1.4725993
C    -0.2950415    4.4184623    1.3851141
H     1.5552778    5.3007613    2.0165727
C    -0.9215639    3.2924132    0.8549071
H    -0.8811280    5.3009379    1.6172174
C    -0.1514966    2.1903799    0.5746828
H    -1.9904778    3.2911825    0.6700728
H    -0.9492070   -1.3450803   -2.1500370
H    -0.6979334   -1.2543929    1.0175471
C    -2.5332721   -0.3335942    1.6190875
H    -2.0582075    0.5664109    2.0266624
H    -3.4757832   -0.0035755    1.1636122
C    -2.8308667   -1.2977899    2.7545429
H    -3.3025386   -2.2002792    2.3486830
H    -1.8852572   -1.6239778    3.2014665
C    -3.7208302   -0.6921701    3.8254420
H    -4.6838083   -0.3828007    3.4092653
H    -3.2542997    0.1920476    4.2683454
H    -3.9200591   -1.4014299    4.6317189
```

2o-TS1(1,6)a

```
E(TPSS-D3/def2-TZVP) = -1287.056547430 (conv)
Lowest Freq. = -1022.71 cm^-1
48
2o-TS1(1,6)a (TS006A/c1/pbe0-d3.def2-TZVP)
C     0.1829566   -0.8384313    0.6258183
C    -1.9072824   -0.4453148   -0.7318140
C    -0.4117443   -0.7193417   -0.7538980
H    -0.2497618   -1.6579133    1.2045541
C     1.6803031   -0.7762763    0.8131444
H    -0.2371244   -1.6486263   -1.3094945
H     0.1008779    0.0727475   -1.3082480
H    -2.4111953   -1.2145243   -0.1329512
H     1.8827208   -0.8434782    1.8855096
O     2.1238267    0.5412143    0.3765061
C     2.5787241   -1.8224807    0.1293266
C     2.0154767   -3.2066974    0.4386510
C     3.9765254   -1.7027960    0.7367097
C     2.6871327   -1.6225881   -1.3799731
H     1.9072522   -3.3621854    1.5159782
H     2.6934148   -3.9748661    0.0599827
H     1.0406883   -3.3632795   -0.0288635
H     1.7437279   -1.7991442   -1.8953804
H     3.4194585   -2.3264183   -1.7832281
H     3.0256563   -0.6134362   -1.6183388
H     3.9542760   -1.8509007    1.8190272
H     4.4106889   -0.7206770    0.5424976
H     4.6378284   -2.4559057    0.3017656
S     2.4167172    1.6708709    1.4346712
O     3.1824602    2.6553617    0.7480225
O     2.9141342    1.0741749    2.6354378
C     0.8166786    2.3374133    1.7740106
C     0.7139059    3.6841616    2.1083114
C    -0.5245376    4.1971684    2.4601018
H     1.5961735    4.3127239    2.0805524
C    -1.6479119    3.3811530    2.4763398
H    -0.6140883    5.2452814    2.7188388
C    -1.5454505    2.0371584    2.1282967
H    -2.6123058    3.7930391    2.7522892
C    -0.3071335    1.5455902    1.7896129
H    -2.4231019    1.4001311    2.1249248
H    -0.1696470    0.1672749    1.2407400
H    -2.0908118    0.5080118   -0.2248573
C    -2.5237083   -0.3998868   -2.1187089
H    -2.0050288    0.3569966   -2.7198892
H    -2.3525347   -1.3573294   -2.6264486
C    -4.0125697   -0.0965496   -2.1061838
H    -4.5288995   -0.8517192   -1.5021803
H    -4.1788727    0.8612680   -1.5998278
C    -4.6206882   -0.0510751   -3.4969144
H    -4.4952743   -1.0072116   -4.0125056
H    -4.1421934    0.7176547   -4.1099289
H    -5.6897395    0.1696575   -3.4619868
```

2o-TS1(1,6)b

```
E(TPSS-D3/def2-TZVP) = -1287.057616079 (conv)
Lowest Freq. =  -889.21 cm^-1
48
2o-TS1(1,6)b (TS006B/c1/pbe0-d3.def2-TZVP)
C     0.2514167   -0.5724118    0.0364775
C    -2.2516105   -1.0159324    0.1171158
C    -0.8521694   -1.5193075    0.4467087
H    -0.0791587    0.5209741    0.4675299
C     1.6185482   -0.8654220    0.6205819
H    -0.7782081   -1.6924934    1.5249918
H    -0.6971815   -2.4944621   -0.0326586
H    -2.9801030   -1.7527179    0.4721127
H     1.4997688   -1.4914835    1.5079403
O     2.2611925    0.3674311    1.0651459
C     2.6293421   -1.5400234   -0.3193215
C     2.0099730   -2.8291504   -0.8522927
C     3.8832563   -1.8830342    0.4819873
C     3.0039501   -0.6281017   -1.4837496
H     1.6972551   -3.4894821   -0.0387905
H     2.7458183   -3.3674708   -1.4538782
H     1.1434095   -2.6332941   -1.4876065
H     2.1379188   -0.3686851   -2.0971349
H     3.7226946   -1.1355759   -2.1315244
H     3.4606307    0.2964842   -1.1285825
H     3.6460235   -2.5519563    1.3138338
H     4.3507920   -0.9862796    0.8899508
H     4.6115909   -2.3869950   -0.1578874
S     1.8825044    1.0115302    2.4534565
O     3.0400644    1.7062288    2.9070503
O     1.2617239    0.0167570    3.2732538
C     0.6694420    2.2025721    1.9861719
C     0.6601398    3.4647429    2.5667362
C    -0.3368109    4.3579511    2.2019341
H     1.4301823    3.7346331    3.2795314
C    -1.3009620    4.0011529    1.2684549
H    -0.3560367    5.3461268    2.6453867
C    -1.2827146    2.7374452    0.6808142
H    -2.0705061    4.7121076    0.9885259
C    -0.2931821    1.8684312    1.0665376
H    -2.0273044    2.4586426   -0.0569869
H     0.2874563   -0.3896731   -1.0384199
H    -2.4442969   -0.1016085    0.6903074
C    -2.4995778   -0.7471532   -1.3579493
H    -1.8165565    0.0305113   -1.7192989
H    -2.2648686   -1.6484121   -1.9390018
C    -3.9274905   -0.3172061   -1.6510366
H    -4.6161919   -1.0966447   -1.3060683
H    -4.1626232    0.5740433   -1.0570408
C    -4.1719165   -0.0309137   -3.1220188
H    -3.9753364   -0.9164613   -3.7326470
H    -3.5169581    0.7675135   -3.4820899
H    -5.2033297    0.2770728   -3.3065525
```

2o-TS1(1,5)

```
E(TPSS-D3/def2-TZVP+COSMO) = -1287.054479377 (conv)
Lowest Freq. = -1060.34 cm^-1
48
2o-TS1(1,5) (TS009/c1c/pbe0-d3.def2-TZVP)
C    -0.4700040   -0.5989635   -0.0325199
C    -2.2366890   -0.3038144   -1.8000311
C    -0.7505249   -0.2463768   -1.4894664
H    -0.6351903   -1.6654005    0.1401828
C     0.9003121   -0.2517552    0.5012053
H    -0.2166107   -0.9256028   -2.1603807
H    -0.3758907    0.7599361   -1.6979748
H    -2.6251374   -1.3000442   -1.5525152
H     0.7932725   -0.3598974    1.7291709
O     1.2333255    1.1342535    0.3398478
C     2.1391125   -1.0606826    0.1110789
C     1.8278543   -2.5482452    0.2416030
C     3.2862280   -0.7070618    1.0596100
C     2.5820994   -0.7463900   -1.3206599
H     1.4484754   -2.7921266    1.2381330
H     2.7400317   -3.1273683    0.0825458
H     1.0933955   -2.8784545   -0.4953370
H     1.8444002   -1.0648203   -2.0565784
H     3.5153988   -1.2742177   -1.5323298
H     2.7599881    0.3218310   -1.4505224
H     3.0446334   -0.9666418    2.0926520
H     3.5182062    0.3580673    1.0215157
H     4.1836945   -1.2598363    0.7734821
S     0.5390389    2.2552125    1.2228073
O    -0.8669439    2.2629360    0.9708552
O     1.3077824    3.4332845    1.0077763
C     0.8258969    1.6373633    2.8461075
C     1.0839409    2.5106682    3.8964716
C     1.2507442    1.9801939    5.1661536
H     1.1574184    3.5757932    3.7122256
C     1.1739463    0.6087198    5.3775822
H     1.4508019    2.6429453    5.9993267
C     0.9342429   -0.2612785    4.3168956
H     1.3118977    0.2100199    6.3766607
C     0.7537317    0.2839349    3.0671837
H     0.8948601   -1.3323276    4.4807849
H    -1.2014803   -0.0745186    0.5870026
H    -2.7626667    0.4008648   -1.1456069
C    -2.5573746    0.0153183   -3.2496602
H    -2.1664652    1.0103457   -3.4953844
H    -2.0274801   -0.6876379   -3.9049657
C    -4.0438080   -0.0313781   -3.5624666
H    -4.4328699   -1.0255534   -3.3134952
H    -4.5702128    0.6717388   -2.9071193
C    -4.3557517    0.2906948   -5.0133889
H    -3.8653282   -0.4169035   -5.6878531
H    -4.0054310    1.2923441   -5.2774127
H    -5.4288707    0.2508318   -5.2131921
```

2o-INT1

```
E(PBE0-D3/def2-TZVP) = -1287.096925690 (conv)
Lowest Freq. =    11.12 cm^-1
48
2o-INT1 (012/c1/pbe0-d3.def2-TZVP)
```

```
C    -1.8606216   -0.1792937    0.3981845
C    -2.5837608    0.0890771    1.7083204
C    -0.6689398   -1.0472083    0.5356613
H    -1.5681510    0.7872280   -0.0467975
H    -2.5590811   -0.6200597   -0.3273980
C    -0.0465764   -1.7440434   -0.6171476
H    -0.1922823   -1.1362706    1.5079072
H    -2.9446857   -0.8574707    2.1274111
H    -0.1429386   -2.8313356   -0.4972734
C     1.4339635   -1.4349708   -0.7853450
H     1.9422241   -1.5145397    0.1790516
O     1.5192216   -0.0472359   -1.2091598
C     2.1973328   -2.2975385   -1.8039313
C     2.2157517   -3.7409702   -1.3032789
C     3.6387158   -1.7994153   -1.8937478
C     1.5503591   -2.2299547   -3.1834864
H     2.6199606   -3.8044590   -0.2889608
H     2.8536147   -4.3458431   -1.9517480
H     1.2232780   -4.1955702   -1.3056582
H     0.5466765   -2.6610986   -3.1847679
H     2.1506612   -2.7908485   -3.9038145
H     1.4842390   -1.1977446   -3.5330521
H     4.1176653   -1.7919554   -0.9114323
H     3.6922731   -0.7891447   -2.3019307
H     4.2160737   -2.4562930   -2.5486026
S     2.3322199    1.0406666   -0.4057954
O     3.0355538    1.7986607   -1.3871761
O     3.0253685    0.4178550    0.6797591
C     1.0540302    2.0565262    0.2501401
C     0.3680627    2.9110368   -0.6027596
C    -0.6350764    3.7133195   -0.0867386
H     0.6306359    2.9479937   -1.6525927
C    -0.9358176    3.6669477    1.2686481
H    -1.1789850    4.3828513   -0.7421378
C    -0.2344318    2.8186259    2.1119620
H    -1.7178105    4.3010505    1.6698238
C     0.7654984    2.0030847    1.6042929
H    -0.4645594    2.7914639    3.1703323
H     1.3303012    1.3367168    2.2432348
H    -0.5699651   -1.4856875   -1.5417139
H    -1.8659099    0.4894403    2.4331859
C    -3.7419415    1.0603569    1.5644654
H    -4.4766351    0.6475392    0.8636631
H    -3.3736190    1.9853134    1.1064908
C    -4.4160650    1.3777162    2.8872706
H    -5.2479676    2.0740841    2.7602672
H    -3.7083013    1.8290392    3.5887744
H    -4.8095586    0.4723578    3.3576002
```

2o-TS2a

```
E(PBE0-D3/def2-TZVP) = -1287.082526187 (conv)
Lowest Freq. =  -368.58 cm^-1
48
2o-TS2a (TS007A/c1/pbe0-d3.def2-TZVP)
C    -1.6721638    0.4181099    0.7194970
C    -2.5865876   -0.7763451    0.9985248
C    -0.2283561    0.0580325    0.7386864
H    -1.8700945    1.1888825    1.4700610
H    -1.9331340    0.8498962   -0.2531356
C     0.3047330   -0.6920164   -0.4343474
```

```
H     0.1717908   -0.2414871    1.7053276
H    -2.4249093   -1.5556912    0.2447706
H    -0.1786793   -1.6775659   -0.4695435
C     1.8028480   -0.8995773   -0.3907872
H     2.0840092   -1.3362137    0.5731873
O     2.4439894    0.3964708   -0.4750270
C     2.4120830   -1.7615729   -1.5034678
C     1.8275936   -3.1691805   -1.4069532
C     3.9212614   -1.8403217   -1.2797731
C     2.1306620   -1.1748268   -2.8834494
H     1.9468430   -3.5824269   -0.4013226
H     2.3494180   -3.8316864   -2.1011025
H     0.7669772   -3.1953734   -1.6649817
H     1.0620197   -1.1600929   -3.1097397
H     2.6195202   -1.7799865   -3.6504914
H     2.5155365   -0.1565311   -2.9631241
H     4.1501108   -2.2535449   -0.2935277
H     4.3857129   -0.8565412   -1.3491211
H     4.3772008   -2.4888129   -2.0314157
S     2.4207962    1.3475361    0.7832451
O     3.3588151    2.3732019    0.4742519
O     2.5657781    0.5724811    1.9779173
C     0.7876148    2.0342614    0.7384190
C     0.3652874    2.6295414   -0.4773783
C    -0.6190057    3.5905696   -0.4622138
H     0.8089487    2.3047434   -1.4101520
C    -1.1393229    4.0602095    0.7439969
H    -0.9687738    4.0084585   -1.3992678
C    -0.6410279    3.5645117    1.9486759
H    -1.9012590    4.8296168    0.7450237
C     0.3328770    2.5922136    1.9597088
H    -1.0080107    3.9599808    2.8888418
H     0.7449935    2.2210373    2.8899568
H     0.0219723   -0.1799595   -1.3605817
H    -2.3165701   -1.2227084    1.9628898
C    -4.0573303   -0.3931433    1.0123293
H    -4.3190982    0.0601186    0.0494796
H    -4.2169682    0.3857245    1.7663978
C    -4.9724325   -1.5725981    1.2903229
H    -6.0227706   -1.2732911    1.2957679
H    -4.7500294   -2.0227910    2.2616674
H    -4.8528689   -2.3513120    0.5319577
```

2o-TS2b

```
E(PBE0-D3/def2-TZVP) = -1287.079617640 (conv)
Lowest Freq. =  -416.64 cm^-1
48
2o-TS2b (TS007B/c1/pbe0-d3.def2-TZVP)
C    -0.7862781   -0.5918123    1.2258266
C    -2.0365337   -0.3299486    2.0512160
C    -0.7751895    0.1297604   -0.0886541
H    -0.6979735   -1.6717405    1.0358208
H     0.1034178   -0.3388203    1.8125837
C    -0.1810108   -0.5595860   -1.2740355
H    -1.6844647    0.6834408   -0.3076791
H    -2.1517268    0.7497878    2.1945761
H    -0.7036072   -1.5150509   -1.4130437
C     1.3038602   -0.8317830   -1.1228222
H     1.5113201   -1.2659660   -0.1397081
O     1.9835203    0.4459708   -1.1809233
```

C	1.9520841	-1.7288084	-2.1841185
C	1.3438013	-3.1254855	-2.0774509
C	3.4478918	-1.8226778	-1.8882212
C	1.7413360	-1.1731221	-3.5890365
H	1.4304854	-3.5207478	-1.0613229
H	1.8739313	-3.8085297	-2.7448557
H	0.2901065	-3.1422599	-2.3628936
H	0.6849727	-1.1609337	-3.8673636
H	2.2655161	-1.7960824	-4.3175006
H	2.1329214	-0.1575164	-3.6721397
H	3.6247692	-2.1954148	-0.8754997
H	3.9334814	-0.8509567	-1.9785907
H	3.9246102	-2.5112082	-2.5896919
S	1.9623324	1.3860581	0.0845740
O	2.8602794	2.4413396	-0.2416498
O	2.1659820	0.6063617	1.2678374
C	0.3051346	2.0123573	0.0933787
C	-0.1250224	2.6856125	-1.0850012
C	-1.1753842	3.5671596	-1.0172441
H	0.3557241	2.4632780	-2.0292256
C	-1.7498581	3.8990182	0.2128733
H	-1.5409216	4.0325340	-1.9253487
C	-1.2113745	3.3783471	1.3885525
H	-2.5725275	4.6018627	0.2559461
C	-0.1643039	2.4861989	1.3481310
H	-1.6056296	3.6927637	2.3481016
H	0.2782578	2.0965437	2.2558429
H	-0.3448397	0.0263053	-2.1810623
H	-2.9201081	-0.6556502	1.4885083
C	-2.0112128	-1.0213187	3.4031280
H	-1.1263700	-0.6888313	3.9577658
H	-1.8859099	-2.1004282	3.2561998
C	-3.2607444	-0.7584260	4.2248452
H	-3.2188188	-1.2613051	5.1933906
H	-4.1556807	-1.1116750	3.7050578
H	-3.3902458	0.3113853	4.4109270

2o-INT2a

```
E(PBE0-D3/def2-TZVP) = -1287.113142432 (conv)
Lowest Freq. =    34.34 cm^-1
48
2o-INT2a (013A/c1/pbe0-d3.def2-TZVP)
C    -1.7293783    0.3507040    0.7762013
C    -2.4026031   -0.9545915    1.1658282
C    -0.2059075    0.2818185    0.7284476
H    -2.0201651    1.1250784    1.4934171
H    -2.1040025    0.6868994   -0.1975726
C     0.2768059   -0.6115824   -0.4109593
H     0.1527236   -0.1413108    1.6742448
H    -2.1873608   -1.7351795    0.4274898
H    -0.1540638   -1.6036058   -0.2693495
C     1.7817600   -0.7583182   -0.4810929
H     2.1534884   -1.1821220    0.4581822
O     2.3693051    0.5631559   -0.6111930
C     2.3346772   -1.6007336   -1.6368653
C     1.7751018   -3.0165929   -1.5195247
C     3.8555454   -1.6567137   -1.5050818
C     1.9641929   -1.0058636   -2.9923983
H     1.9666706   -3.4405048   -0.5295389
H     2.2560976   -3.6643526   -2.2557752
H     0.6997052   -3.0507791   -1.7052857
H     0.8849778   -1.0135592   -3.1611867
H     2.4232305   -1.5928480   -3.7913180
H     2.3213706    0.0215254   -3.0817891
H     4.1500529   -2.0797678   -0.5407737
H     4.2977319   -0.6634880   -1.5871079
H     4.2770393   -2.2856616   -2.2926334
S     2.2039961    1.5197714    0.6379279
O     2.8523315    2.7333328    0.2772588
O     2.5834146    0.8151174    1.8266500
C     0.3777039    1.7290634    0.6636999
C    -0.0141606    2.4710711   -0.5660234
C    -0.5482722    3.7185951   -0.5172892
H     0.1635278    1.9915415   -1.5201363
C    -0.7677305    4.3810182    0.7042078
H    -0.8106631    4.2153987   -1.4446981
C    -0.4427910    3.7301539    1.9092044
H    -1.1838147    5.3797946    0.7172180
C     0.0843150    2.4794891    1.9184901
H    -0.6257273    4.2330948    2.8522481
H     0.3342476    1.9840963    2.8493084
H    -0.1070548   -0.2339557   -1.3628620
H    -1.9836057   -1.3099164    2.1156158
C    -3.9103538   -0.8172228    1.3031953
H    -4.3252343   -0.4521483    0.3568149
H    -4.1336907   -0.0453074    2.0481871
C    -4.5901233   -2.1177855    1.6927884
H    -5.6710675   -1.9937479    1.7865666
H    -4.2135297   -2.4867602    2.6507592
H    -4.4087128   -2.8963009    0.9465034
```

2o-INT2B

```
E(PBE0-D3/def2-TZVP) = -1287.106251729 (conv)
Lowest Freq. =    35.40 cm^-1
48
2o-INT2B (013B/c1/pbe0-d3.def2-TZVP)
C    -0.9543751   -0.5659383    1.1266587
C    -2.3147200   -0.3237694    1.7620968
C    -0.7084784    0.2821401   -0.1295464
H    -0.8895012   -1.6235970    0.8473082
H    -0.1610849   -0.4120762    1.8606678
C    -0.0442348   -0.5246377   -1.2476065
H    -1.6879199    0.5950661   -0.5071134
H    -2.4446948    0.7448347    1.9661336
H    -0.6176125   -1.4444925   -1.3770396
C     1.4007307   -0.8636836   -0.9539700
H     1.4861966   -1.3492859    0.0242565
O     2.1249636    0.3935008   -0.8742971
C     2.1320080   -1.7372214   -1.9787586
C     1.4421103   -3.0982910   -2.0344662
C     3.5708376   -1.9316697   -1.5041321
C     2.1333648   -1.0954870   -3.3629255
H     1.3725128   -3.5501871   -1.0408161
H     2.0179201   -3.7770012   -2.6674780
H     0.4358304   -3.0340913   -2.4532847
H     1.1249355   -1.0114428   -3.7743484
H     2.7186808   -1.7066196   -4.0538788
H     2.5796833   -0.0997635   -3.3338860
H     3.5977379   -2.3805765   -0.5074388
H     4.1063129   -0.9829861   -1.4607000
H     4.1042031   -2.5955675   -2.1883948
S     1.8143692    1.2978610    0.3852427
O     2.5833874    2.4803320    0.2008933
O     1.9685262    0.5162201    1.5764381
C     0.0259338    1.6495082    0.1333431
C    -0.0811810    2.5366071   -1.0625391
C    -0.5965835    3.7901580   -0.9954522
H     0.2769889    2.1478834   -2.0069085
C    -1.0374534    4.3438093    0.2203399
H    -0.6635844    4.3793054   -1.9032661
C    -0.9281712    3.5818586    1.3974443
H    -1.4382894    5.3482963    0.2522956
C    -0.4305619    2.3184368    1.3859986
H    -1.2461237    4.0114957    2.3409610
H    -0.3253351    1.7620995    2.3077676
H    -0.0950638    0.0168442   -2.1942221
H    -3.1002059   -0.5872618    1.0423932
C    -2.5185598   -1.1112394    3.0447353
H    -1.7382239   -0.8339416    3.7625825
H    -2.3731879   -2.1785100    2.8407545
C    -3.8870651   -0.8896194    3.6643795
H    -4.0084627   -1.4618207    4.5865935
H    -4.6835693   -1.1911782    2.9784693
H    -4.0429903    0.1656988    3.9047157
```

2o-TS3a

```
E(PBE0-D3/def2-TZVP) = -1287.103378560 (conv)
Lowest Freq. =  -280.60 cm^-1
48
2o-TS3a (TS008A/c1/pbe0-d3.def2-TZVP)
C    -1.8636904    0.2067701    0.8048965
C    -2.3319195   -1.1784292    1.2183896
C    -0.3421810    0.3589938    0.6797913
H    -2.2224782    0.9328562    1.5418310
H    -2.3286574    0.4922420   -0.1466143
C     0.1925691   -0.4520673   -0.5004056
H     0.1098491   -0.0359695    1.5980155
H    -2.0470363   -1.9242392    0.4682038
H    -0.2402720   -1.4508382   -0.4359718
C     1.6989661   -0.6155695   -0.5412862
H     2.0445776   -1.0307924    0.4123106
O     2.3254263    0.6801421   -0.6972879
C     2.2505215   -1.5030388   -1.6669376
C     1.6866936   -2.9133122   -1.5078303
C     3.7706837   -1.5644663   -1.5263136
C     1.8890398   -0.9504128   -3.0425810
H     1.8684982   -3.3044408   -0.5026126
H     2.1739087   -3.5856098   -2.2176157
H     0.6133330   -2.9547708   -1.7033278
H     0.8106238   -0.9637064   -3.2166737
H     2.3531312   -1.5607277   -3.8209248
H     4.0579632   -1.9494016   -0.5439836
H     4.2209211   -0.5790159   -1.6467894
H     4.1900409   -2.2287368   -2.2855692
S     2.2287183    1.6483647    0.5747990
O     2.8890589    2.8537452    0.1730407
O     2.6675530    0.9311742    1.7470205
C    -0.0209236    1.8517913    0.6394031
C    -0.2586222    2.6010313   -0.5534472
C    -0.3885878    3.9683287   -0.5151909
H    -0.2783770    2.0870085   -1.5059401
C    -0.3701988    4.6529336    0.6991629
H    -0.5163689    4.5182531   -1.4402860
C    -0.2588680    3.9355974    1.8928985
H    -0.4621841    5.7316326    0.7181228
C    -0.1327274    2.5705141    1.8747302
H    -0.2837368    4.4594648    2.8412856
H    -0.0440427    2.0130235    2.8006129
H    -0.1611937   -0.0234024   -1.4418122
H    -1.8232223   -1.4703794    2.1456557
C    -3.8363219   -1.2499695    1.4268828
H    -4.3419628   -0.9447214    0.5036835
H    -4.1288377   -0.5175226    2.1875566
C    -4.3108942   -2.6326686    1.8374540
H    -5.3926527   -2.6585035    1.9847266
H    -3.8404303   -2.9485391    2.7726381
H    -4.0613669   -3.3770136    1.0761802
H     2.2456776    0.0743979   -3.1598926
```

2o-TS3b

```
E(PBE0-D3/def2-TZVP) = -1287.097572518 (conv)
Lowest Freq. =  -316.92 cm^-1
48
2o-TS3b (TS008B/c1/pbe0-d3.def2-TZVP)
C    -0.9813968   -0.5809595    1.0766488
C    -2.3527009   -0.4939592    1.7301598
C    -0.8654305    0.2393077   -0.2131003
H    -0.7847379   -1.6321690    0.8390189
H    -0.2001315   -0.2947331    1.7858068
C    -0.0626329   -0.5023332   -1.2902301
H    -1.8812973    0.3249300   -0.6213381
H    -2.6405071    0.5542527    1.8685514
H    -0.5674976   -1.4562495   -1.4560240
C     1.3881880   -0.7697211   -0.9443551
H     1.4673668   -1.1922921    0.0631632
O     2.0927527    0.4961011   -0.9413438
C     2.1528388   -1.7001093   -1.8962910
C     1.5137268   -3.0862730   -1.8488624
C     3.5946172   -1.8098799   -1.4029164
C     2.1411915   -1.1681975   -3.3262073
H     1.4484573   -3.4593344   -0.8226478
H     2.1228763   -3.7912443   -2.4189775
H     0.5116168   -3.0969948   -2.2820425
H     1.1326993   -1.1517458   -3.7459761
H     2.7518846   -1.8094814   -3.9661503
H     2.5511431   -0.1575820   -3.3719497
H     3.6296054   -2.1733448   -0.3721786
H     4.1008226   -0.8450676   -1.4363621
H     4.1511239   -2.5117786   -2.0283975
S     1.7655978    1.4493480    0.3021006
O     2.4697573    2.6653677    0.0297409
O     2.0173503    0.7297936    1.5266897
C    -0.4478140    1.7050963   -0.0004196
C    -0.4577983    2.5582844   -1.1538385
C    -0.5317607    3.9221278   -1.0353647
H    -0.3566204    2.1127039   -2.1352379
C    -0.6792340    4.5221040    0.2160437
H    -0.4882666    4.5380252   -1.9260194
C    -0.7927253    3.7171479    1.3502904
H    -0.7266416    5.6002104    0.3035527
C    -0.7288484    2.3498896    1.2520470
H    -0.9438063    4.1732276    2.3217536
H    -0.7990979    1.7462916    2.1463497
H    -0.0995325    0.0369914   -2.2389209
H    -3.1003683   -0.9171679    1.0475625
C    -2.4220518   -1.2155483    3.0649593
H    -1.6835114   -0.7791250    3.7470342
H    -2.1209084   -2.2604003    2.9261174
C    -3.8011819   -1.1591125    3.6977580
H    -3.8248565   -1.6794656    4.6575775
H    -4.5509427   -1.6217966    3.0499802
H    -4.1113169   -0.1251346    3.8722454
```
